# Regioselective Radical Arene Amination for the Concise Synthesis of *ortho*-Phenylenediamines

**DOI:** 10.1021/jacs.1c05531

**Published:** 2021-06-15

**Authors:** James E. Gillespie, Charlotte Morrill, Robert J. Phipps

**Affiliations:** Yusuf Hamied Department of Chemistry, University of Cambridge, Lensfield Road, Cambridge CB2 1EW, U.K.

## Abstract

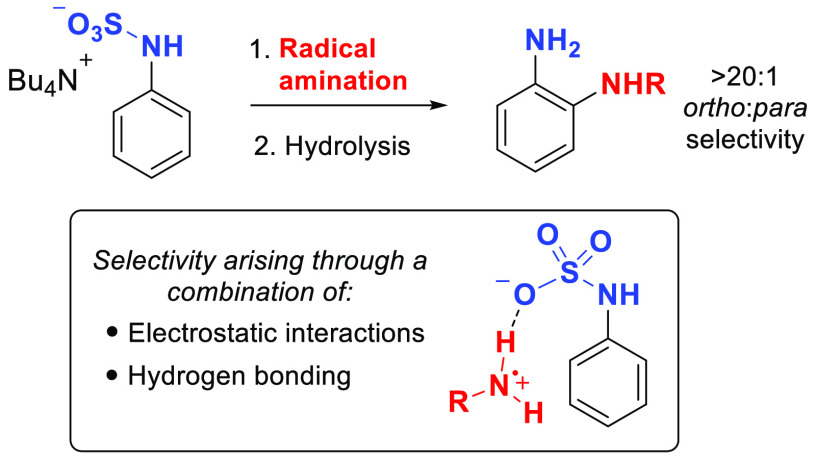

The formation of arene C–N bonds directly from C–H bonds is of great importance and there has been rapid recent development of methods for achieving this through radical mechanisms, often involving reactive *N*-centered radicals. A major challenge associated with these advances is that of regiocontrol, with mixtures of regioisomeric products obtained in most protocols, limiting broader utility. We have designed a system that utilizes attractive noncovalent interactions between an anionic substrate and an incoming radical cation in order to guide the latter to the arene *ortho* position. The anionic substrate takes the form of a sulfamate-protected aniline and telescoped cleavage of the sulfamate group after amination leads directly to *ortho*-phenylenediamines, key building blocks for a range of medicinally relevant diazoles. Our method can deliver both free amines and monoalkyl amines allowing access to unsymmetrical, selectively monoalkylated benzimidazoles and benzotriazoles. As well as providing concise access to valuable *ortho*-phenylenediamines, this work demonstrates the potential for utilizing noncovalent interactions to control positional selectivity in radical reactions.

Aromatic amines are ubiquitous in pharmaceuticals, agrochemicals, and natural products. Specifically, *o*-phenylenediamines are important intermediates for the synthesis of a variety of heterocycles such as benzimidazoles, 1,5-benzodiazepines, benzotriazoles, and quinoxalines, as found in numerous pharmaceuticals (Figure 1a).^1^ Classically, amines are installed onto aromatic rings via electrophilic nitration.^2^ However, the harsh conditions and formation of regioisomers limit applicability. Transition-metal-catalyzed cross-couplings have become the most established modern methods for arylamine synthesis, but require selective prefunctionalization of the aromatic substrate, incurring synthetic cost.^3^ Many recent advances have been made in directed transition-metal-catalyzed C–H amination of arenes.^4^ Several methods for *ortho*-selective C–H amination of aniline derivatives have been reported, generating variously *N*-substituted *o*-phenylenediamine derivatives, using Pd,^5^ Cu,^6^ Ru,^7^ Ir,^8^ and Co^9^ catalysis. While some protocols permit subsequent manipulations to obtain the free *o*-phenylenediamines, in practice there are limited means to obtain these extremely useful intermediates in a concise manner.

**Figure 1:**
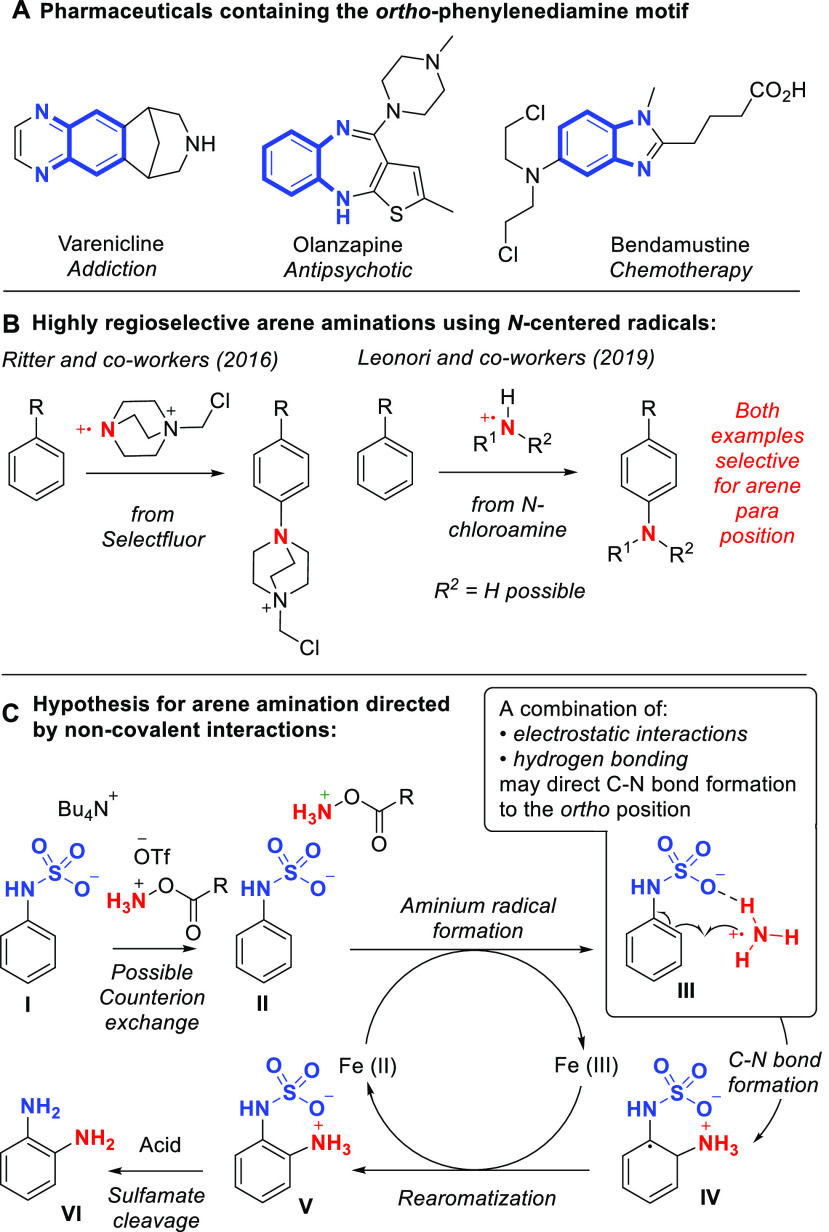
Background and hypothesis.

Mechanistically distinct to these methods is electrophilic amination proceeding via radical intermediates. While it has long been appreciated that electrophilic aminium radical cations react with aromatic systems,^10^ the forcing or inconvenient conditions traditionally required to produce them have hampered adoption. Recent advances have overcome these obstacles and have seen numerous new methods for arene amination utilizing *N*-centered radicals.^11^ Fragments such as imides,^12^ sulfonamides,^13^ amides,^14^ alkylamines,^15^ pyridiniums,^16^ 1,4-diazabicyclo[2.2.2] octane,^17^ and free amines^18^ have been variously incorporated onto arenes. The biggest barrier to widespread adoption of these methods is the challenge of positional selectivity; the majority of examples give rise to mixtures of regioisomers when given a choice and few studies have made headway in tackling this. Notable exceptions, from Ritter and co-workers^17^ and Leonori and co-workers,^15b^ have shown that careful tailoring of the structure of the aminium radical can result in high levels of *para*-selectivity (Figure 1b). A complementary approach to *para*-selective amination has been reported by Nicewicz and co-workers whereby an electron rich arene is oxidized and trapped with a nitrogen source.^19^ Strategies for achieving *ortho*-selective amination using radical approaches are largely undeveloped.^20^

In many of the aforementioned reactions, *N*-centered radical cations are proposed to be the key reactive species; to us their charged nature presented an exciting opportunity to utilize ion-pairing interactions between radical and substrate to exert control over regioselectivity in the C–N bond forming step. Furthermore, many aminium radicals bear multiple N–H bonds, which could feasibly act as hydrogen bond donors to interact with a suitable acceptor on the substrate. While noncovalent interactions, including electrostatic interactions, have been used to control regioselectivity in metal-catalyzed arene C–H functionalization, most extensively in iridium-catalyzed borylation,^21^ this approach remains largely unexplored in radical-based arene functionalization.^20^ We were drawn to the use of cationic N–O reagents as radical precursors, as utilized for arene amination independently by Morandi and co-workers^18a^ and Jiao and co-workers.^18b^ Here an iron catalyst mediates the redox events and the intermediacy of an unsubstituted aminium radical cation results in free amine products. We envisaged that facile conversion of aniline to sulfamate salt **I** (Figure 1c) would install an anionic group capable of engaging in attractive noncovalent interactions with the incoming aminium radical cation.^22^**I** may undergo ion exchange with the cationic radical precursor, although this step may not be essential (**I**–**II**). Importantly, once reduction of the N–O bond is accomplished (**II**–**III**), the approaching aminium radical should be directed to attack the proximal arene *ortho* position (**III**–**IV**) by the anionic sulfamate group of the substrate through a combination of electrostatic interactions and hydrogen bonding. Following oxidation and rearomatization (**IV**–**V**), treatment with acid would cleave the sulfamate resulting in the *ortho*-phenylenediamine product **VI**. A concern at the outset was that the published protocols utilize very polar solvent mixtures: MeCN/H_2_O^18a^ or TFE/H_2_O.^18b^ A subsequent detailed study from Ritter and co-workers showed that use of hexafluoroisopropanol (HFIP) increases reactivity, through proposed hydrogen bonding with the conjugate anions of various intermediates.^18c^ We reasoned that if both Coulombic electrostatic interactions and hydrogen bonding are working in tandem, these interactions may still be sufficient for useful levels of selectivity, even in relatively polar solvents.

We commenced our studies using the sulfamate salt derived from aniline (**1a**), aminating agent **2a** and FeBr_2_ as the catalyst (Table 1). In both MeCN/H_2_O and TFE/H_2_O, product was obtained in modest but encouraging yield and *ortho*:*para* selectivity was 4:1, close to the statistical ratio of 2:1 but showing a small bias toward the *ortho* position (Table 1, entries 1 and 2). In line with our hypothesis, removing the most polar component from these mixtures greatly improved selectivity as in both MeCN and TFE only the *ortho* isomer was observed (entries 3 and 4).

**Table 1:**
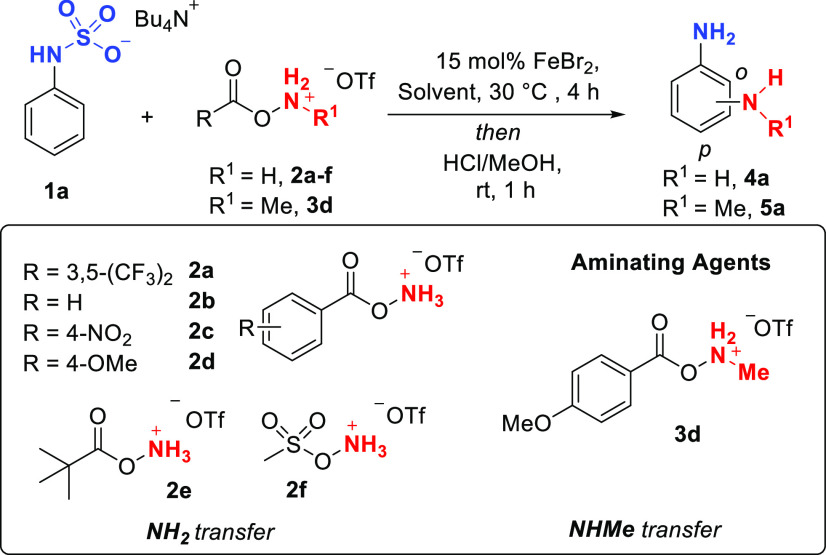
Optimization Studies^a^

entry	solvent (ε)	aminating agent	yield	selectivity (*o*:*p*)
1	CH_3_CN:H_2_O, 2:1	**2a**	28	4:1
2	TFE:H_2_O, 2:1	**2a**	35	4:1
3	CH_3_CN (38)	**2a**	38	>20:1
4	TFE (9)	**2a**	45	>20:1
5	DMA (38)	**2a**	16	7:1
6	EtOAc (6)	**2a**	40	>20:1
7	MeOH (33)	**2a**	21	9:1
8	*i*PrOH (18)	**2a**	13	>20:1
9	HFIP (16)	**2a**	47	>20:1
10	HFIP	**2b**	38	>20:1
11	HFIP	**2c**	40	>20:1
12	HFIP	**2d**	60 (57)	>20:1
13	HFIP	**2e**	38	>20:1
14	HFIP	**2f**	6	–
15^b^	HFIP	**2d**	<5	–
16	HFIP	**3d**	68 (61)	17:1

a Yields and ratios were determined by ^1^H NMR with internal standard. Yield in parentheses is isolated.

b No iron catalyst.

We then compared several aprotic solvents with MeCN, to probe selectivity trends. DMA has a similar dielectric constant to MeCN but exhibited reduced selectivity (7:1), most likely due to its high proficiency as a hydrogen bond acceptor, interrupting critical interactions (entry 5). Accordingly, switching to less polar EtOAc restored excellent selectivity, in line with our hypothesis (entry 6). For protic solvents, MeOH, of significantly higher dielectric constant than TFE, gave reduced selectivity (9:1, entry 7). Switching to less polar *i*PrOH returned the selectivity to >20:1, albeit in low yield (entry 8). Finally, HFIP was found to retain excellent (>20:1) regioselectivity and give the best product yield thus far (entry 9).^23^ We next evaluated a series of different aminating agents (entries 10–14) and found that the NMR yield could be increased to 60% by tuning the substitution on the aromatic ring, giving an isolated yield of 57% (entry 12). Product regioselectivity was unaffected by choice of aminating agent, in line with the proposed mechanism. In the absence of iron catalyst, only traces of product were observed (entry 15), although a more electron rich substrate gave some conversion at higher temperature, in line with observations of Morandi and co-workers in closely related systems (see SI).^15c^ Of several iron(II) sources evaluated, FeBr_2_ was optimal although several reaction components could feasibly ligate iron, making identification of the true active iron catalyst challenging. It is important to remember that while a multitude of ionic species may be present in solution, in addition to those explicitly depicted in Figure 1c, as long as the crucial interactions between substrate and incoming radical occur, then high selectivity should be achievable. Finally, we questioned whether an *N*-methylated aminating agent may enable transfer of NHMe, allowing access to selectively monoalkylated *o*-phenylenediamines.^15c^ Pleasingly, use of **3d** in place of **2d** gave the aminomethylated product with an *ortho*:*para* selectivity of 17:1 and in good isolated yield (entry 16).

First, the scope of NHMe transfer was evaluated and we were pleased to see high levels of *ortho* selectivity for a range of different aniline substrates (Scheme 1). Substrates with alkyl groups at the 2-position were well tolerated, giving good yields and excellent *ortho* selectivity (**5b**–**5d**), as were methyl and isopropyl at the 3-position (**5e**, **5f**). While *ortho* vs *para* selectivity was excellent, low regioselectivity (2.9:1) between the two distinct *ortho* positions was seen for **5e** but improved (5.7:1) with the bulkier isopropyl substituent (**5f**). An alkyne-containing substrate (**5g**), one bearing an alkyl group at the 4-position (**5h**) as well as multiple alkyl substituents on the ring were also well accommodated (**5i**–**5k**). Substrates bearing methoxy groups (**5l**, **5m**) and difluoromethoxy groups (**5n**) also worked well. In the cases where two *ortho* isomers were obtained, these could be separated on silica (**5l**, **5n**). Halides including Br, Cl, and F could be incorporated in various positions (**5o**–**5s**). Given that alkenes are known to undergo aminochlorination with related aminating agents,^24^ we were pleased that a substrate bearing an allyl substituent demonstrated excellent chemoselectivity (**5t**). Substrates bearing other arenes did not pose problems and only amination on the aniline-derived ring was observed (**5u**–**5w**). Finally, using different aminating agents we transferred several other *N*-alkyl groups including *N*-ethyl (**5x**), *N*-propyl (**5y**), *N*-propanenitrile (**5z**), and *N*-hexyl (**5aa**). Heterocyclic, polycyclic, and substrates bearing electron withdrawing groups, protected amines, and vinyl groups exhibited poor reactivity (see SI for details).

**Scheme 1:**
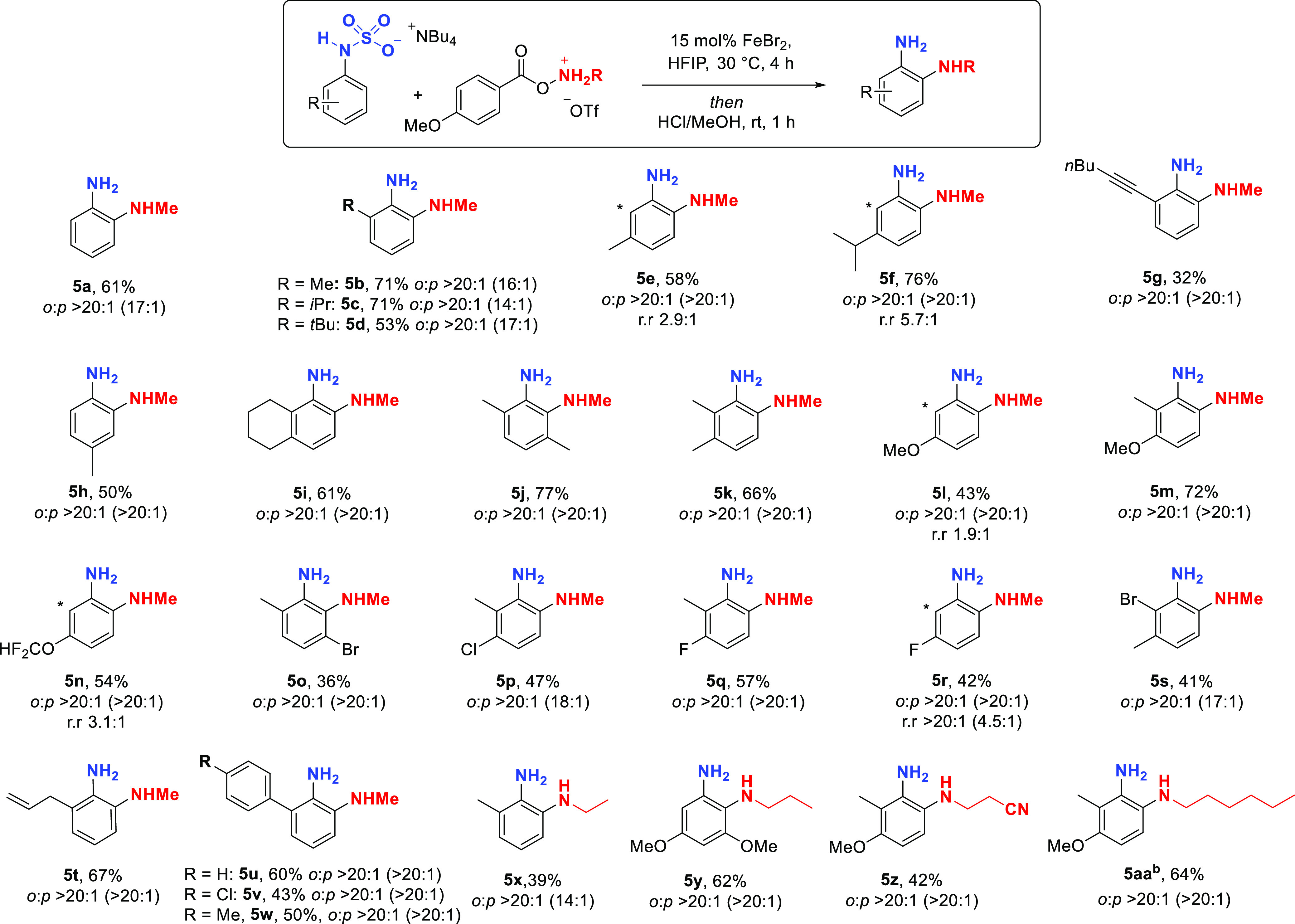
Scope of the *ortho*-Selective Amination for Transfer of NHMe Main *ortho*:*para* ratio quoted is after isolation, crude ratio in parentheses. Yields are isolated. If two ortho positions available, main regioisomeric ratio (r.r.) quoted after isolation, crude ratio shown in parentheses if different. Major regioisomer shown, minor indicated by (*). Product isolated as corresponding benzimidazole.

We next evaluated the scope of NH_2_ transfer (Scheme 2). Anilines bearing alkyl groups in the 2-position were well tolerated (**4b**, **4c**), giving the aminated products with excellent *ortho* selectivity (>20:1 in all cases by crude NMR and when isolated). Halogen substituents at the 3-position were readily incorporated (**4d**–**4g**) and the two *ortho* regioisomers were separable on silica. Several 2,3-disubstituted substrates were also effective (**4h**, **4i**). We were pleased to discover that *N*-alkylated aniline sulfamate salts also underwent the amination, delivering mono-*N*-alkylated *o*-phenylenediamines, with *N*-benzyl (**4j**), *N*-isopropyl (**4k**), and *N*-methyl (**4l**) all being compatible.

**Scheme 2:**
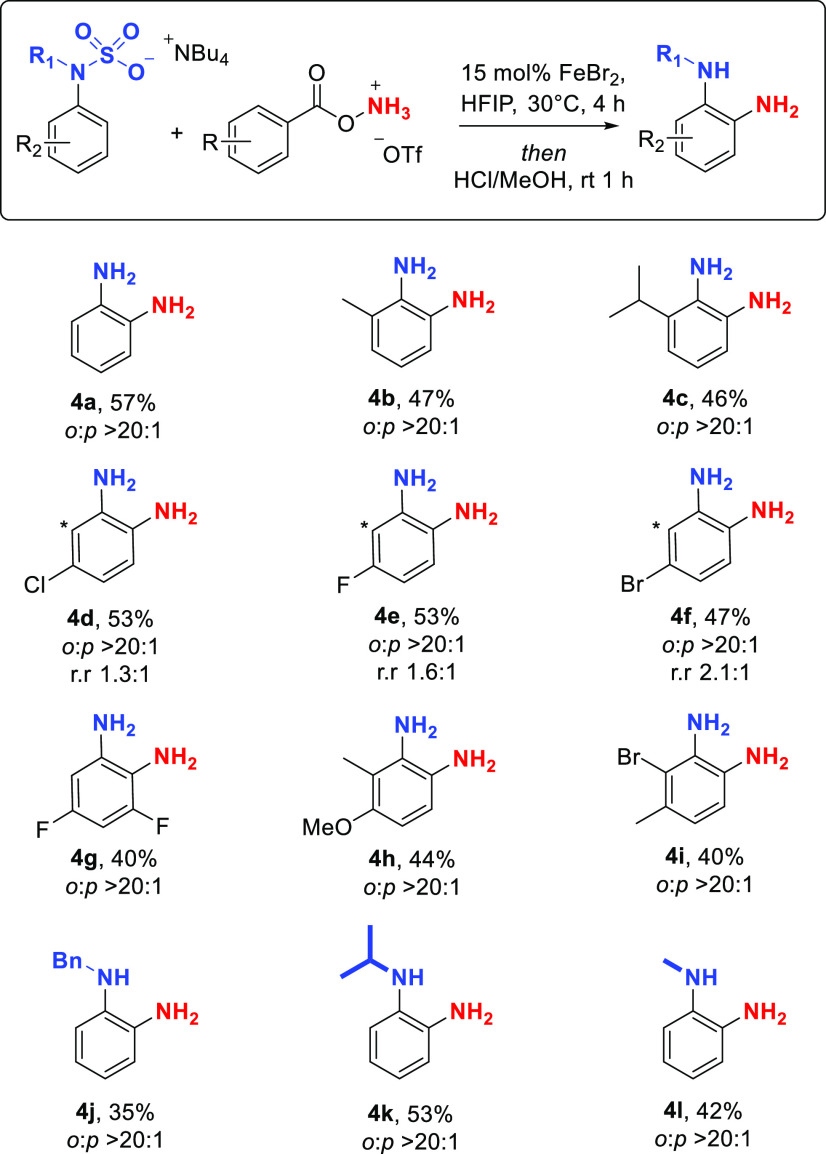
Scope of the *ortho*-Selective Amination for Transfer of NH_2_ *Ortho*:*para* ratio is that after isolation. In all cases, crude *ortho*:*para* ratio was >20:1.

Benzimidazoles and benzotriazoles are commonly synthesized from *o*-phenylenediamines and a great challenge of their chemistry is selective *N*-alkylation.^25,26^ We imagined exploiting our protocol to enable separate access to each isomer of nonsymmetrical *N*-methyl benzimidazoles and benzotriazoles. Telescoping the NHMe transfer to N–H sulfamate substrate **1k** with sulfamate cleavage and benzimidazole formation in one sequence worked extremely well (Scheme 3a). Conversely, by starting with *N*-methyl sulfamate **1ae** and performing NH_2_ transfer, the complementary alkylated regioisomer **6b** could be obtained (Scheme 3b). The same divergent strategy is applicable to benzotriazoles and either N-1 (**6c**) or N-3 (**6d**) methylated isomers could be selectively obtained (Scheme 3c, d). Here, direct alkylation would be even more challenging as N-2 is also liable to alkylation.^27^ We also telescoped our amination together with quinoxaline and benzodiazepine formation (Scheme 3e).

**Scheme 3:**
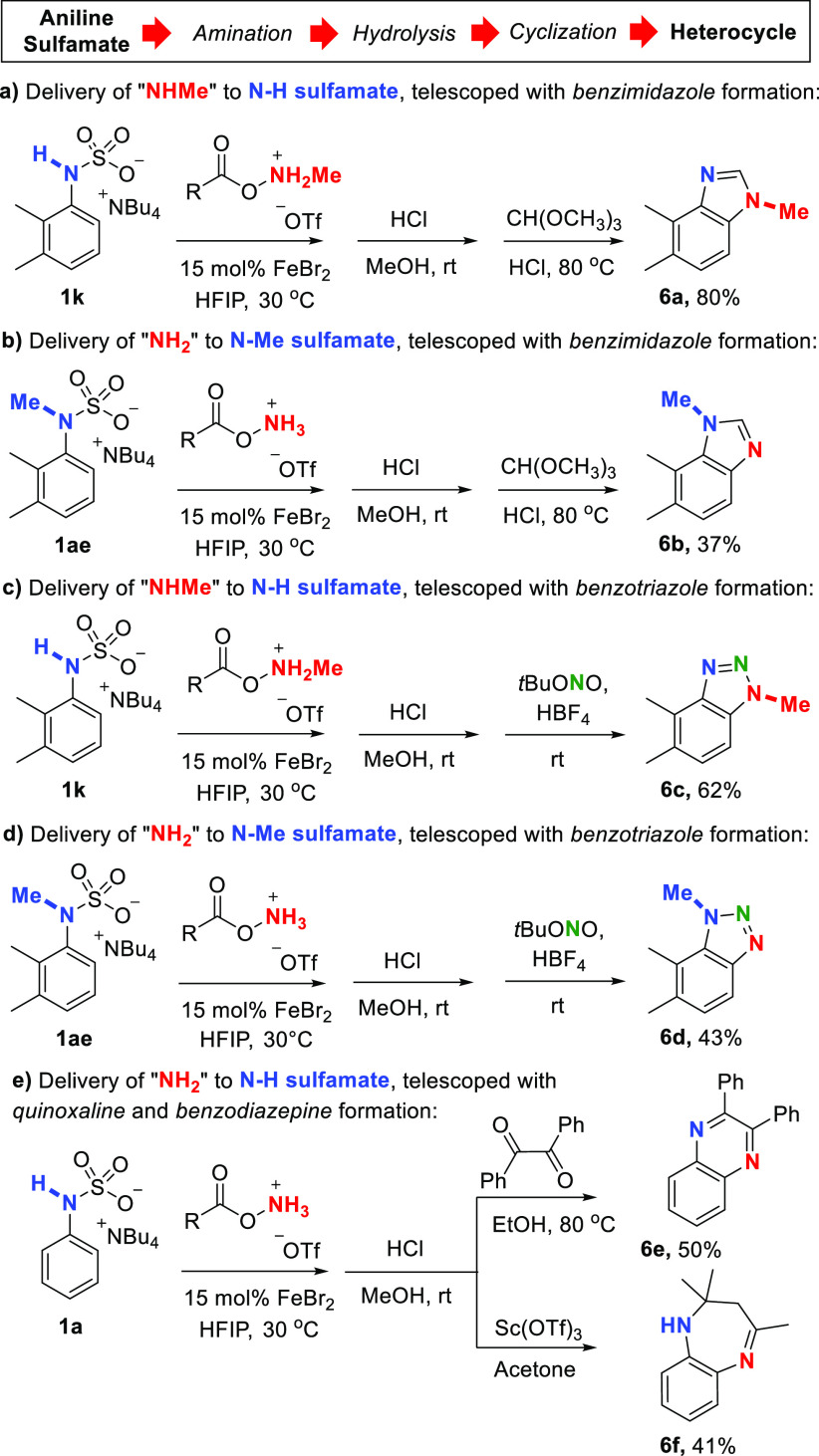
Telescoped Transformations to Heterocycles

To probe our hypothesis that attractive noncovalent interactions between the anionic substrate and the aminium radical cation are responsible for selectivity, we performed a control reaction with neutral sulfamate ester **7** (Figure 2a), which demonstrated that the anionic sulfamate is critical. To probe the effect in our optimal system of systematically increasing the dielectric constant of the solvent, we added varying amounts of water (ε = 80) to the HFIP (ε = 16) solvent. Selectivity quickly dropped off beyond 10% v/v and was essentially statistical at 50% v/v (Figure 2b). The dielectric constant of HFIP/H_2_O mixtures varies approximately linearly in relation to the volume of added water.^28^ Our observation that the relationship between water concentration and regioselectivity is nonlinear likely reflects that a combination of hydrogen bonding and electrostatic interactions are at play. Finally, we evaluated whether our strategy may be viable on a phenol-derived sulfate salt, to access 2-aminophenols (Figure 2c). While the reactivity of **8** was relatively low, crucially the selectivity was >20:1 for the *ortho* position. This provides further support for our hypothesis on the origin of selectivity. We anticipate that future developments to increase reactivity may enable this to become a synthetically useful process.

**Figure 2:**
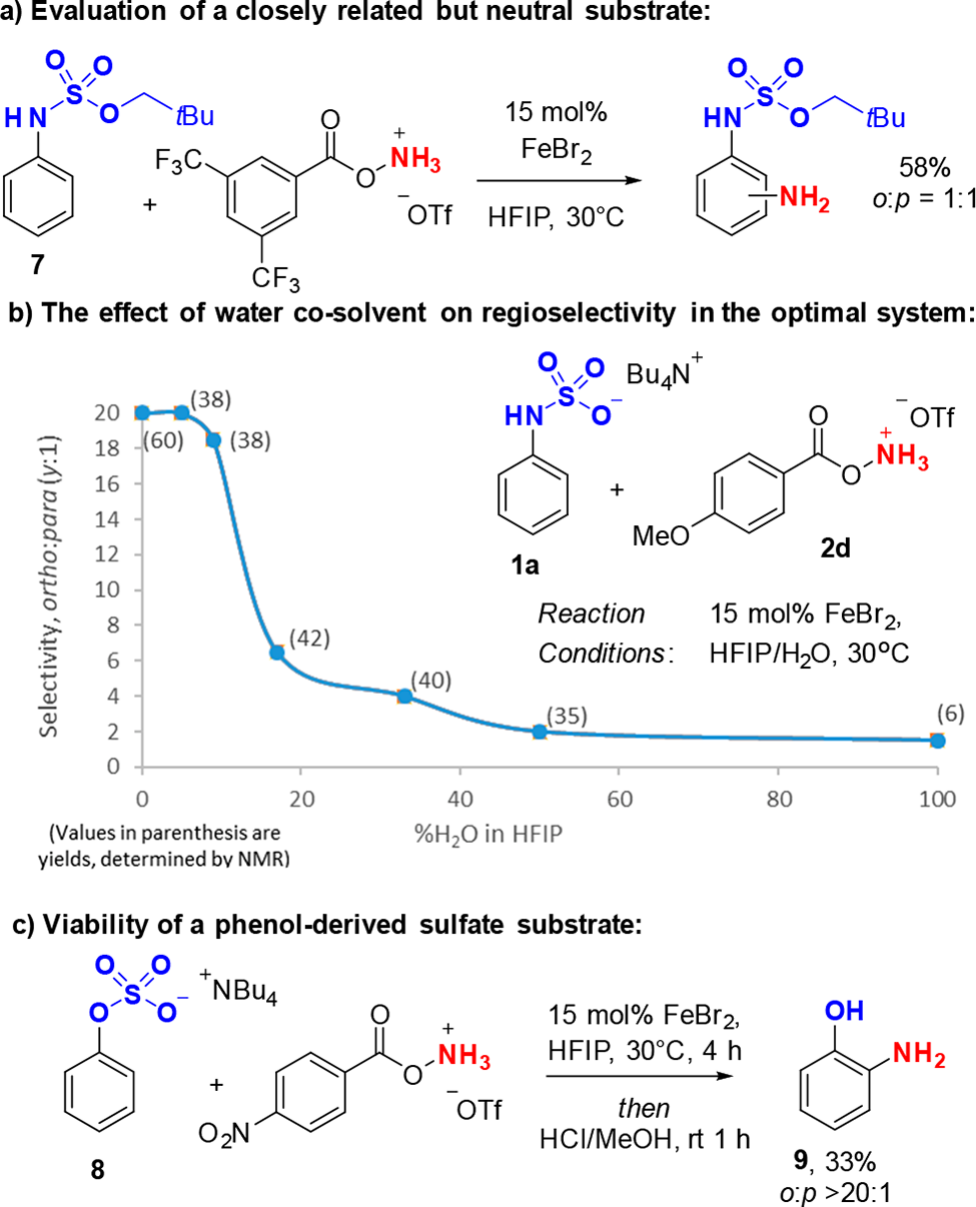
Experiments to probe the origin of selectivity and extension to phenols.

In conclusion, we have developed an *ortho*-selective radical amination of aniline-derived sulfamate salts which allows transfer of NH_2_ and alkylamine groups. Our method allows rapid conversion of anilines to a variety of diazines and triazines, and we envisage it will have particular utility where selective *N*-alkylation is required. We propose that the origin of selectivity is attractive noncovalent interactions between the anionic sulfamate substrate and cationic *N*-centered radical. While we anticipate that these results will have practical utility in heterocyclic chemistry, more broadly they demonstrate the potential of harnessing noncovalent interactions for controlling positional selectivity in radical reactions.

## Supporting Information Available

Additional optimization, full experimental details, and characterization data for compounds (PDF)
