# Supplementary material for: Regioselective Radical Arene Amination for the Concise Synthesis of ortho-Phenylenediamines
Source: J Am Chem Soc. 2021 Jun 15;143(25):9355–60. doi: 10.1021/jacs.1c05531 (PMC8251697; doi:10.1021/jacs.1c05531)

Regioselective Radical Arene Amination for the Concise Synthesis of *ortho*-Phenylenediamines

James E. Gillespie, Charlotte Morrill and Robert J. Phipps*

Yusuf Hamied Department of Chemistry, University of Cambridge, Lensfield Road, Cambridge, CB2 1EW, UK

*rjp71@cam.ac.uk

Contents

1. General Information S2
2. Additional Optimisation S3
3. General Procedures S8
4. Assignment of Product Regiochemistry S9
5. Limitations S10
6. Tetrabutylammonium Sulfamate Salt Synthesis S10
7. Synthesis of Aminating Agents S39
8. NHMe and NH-Alkyl Transfer Products S52
9. NH_2 Transfer Products S69
10. Product Functionalisations S76
11. Amination of a Neutral Substrate S81
12. Viability of a Phenol Derived Substrate S83
13. Effect of Water Concentration on Selectivity S90
14. References S90
15. NMR Spectra S93

1. General Information

NMR spectra: 1H NMR spectra were recorded on a 700 MHz TXO Cryoprobe spectrometer, a 600 MHz Bruker Avance DRX-600 spectrometer, a 400 MHz Avance III HD Smart probe spectrometer, and a 400 MHz QNP Cryoprobe spectrometer. Chemical shifts are reported in parts per million (ppm) and the spectra are calibrated to the resonance resulting from incomplete deuteration of the solvent ($CDCl_3$: 7.26 ppm, CD_3OD: 3.31 ppm, $(CD_3)_2SO$: 2.50 ppm, CD_3CN: 1.94 ppm). 13C NMR spectra were recorded with the same spectrometers with complete proton decoupling. Chemical shifts are reported in ppm with the solvent resonance as the internal standard ($^{13}CDCl_3$: 77.16 ppm, t; $^{13}CD_3OD$: 49.00, sept; $^{13}(CD_3)_2SO$: 39.52, sept; $^{13}CD_3CN$: 1.32, hept, 118.26, s). Data are reported as follows: chemical shift δ/ppmintegration (1H only), multiplicity (s = singlet, d = doublet, t = triplet, q = quartet, quin = quintet, sept = septet, br = broad, m = multiplet or combinations therof; 13C signals are singlets unless stated otherwise), coupling constants J in Hz. 1H-COSY, DEPT-135, HMQC and HMBC were used where appropriate to facilitate structural determination of regioisomers. 19 F NMR spectra were recorded on a 400 MHz Avance III HD Smart probe spectrometer and were proton decoupled.

High Resolution Mass Spectrometry (HRMS): Samples were recorded on a Waters Micromass LCT Premier or a Waters Xevo G2-S or a Waters Vion QTof spectrometer using a positive electrospray ionization (ESI+). The measured values are reported to 4 decimal places and are within ±5 ppm of the calculated value. The calculated values are based on the most abundant isotope

Chromatography: Analytical thin layer chromatography was performed using precoated Merck glass backed silica gel plates (Silicagel 60 F254). Visualisation was by ultraviolet fluorescence (λ = 254 nm) and/or staining potassium permanganate (KMnO4). Flash column chromatography was performed using silica gel 60 (0.040-0.063 µm) from Material Harvest.

Reagents: Unless stated otherwise were used as supplied from commercial sources without further purification. CH_2Cl_2, THF, Et_2O were purified by distillation on site under an inert atmosphere via the following processes: THF and Et_2O were pre-dried over sodium wire then distilled from calcium hydride and lithium aluminium hydride. CH_2Cl_2, *n*-hexane and toluene were distilled from calcium hydride.

2. Additional Optimisation

Table S1: Evaluation of reaction concentration

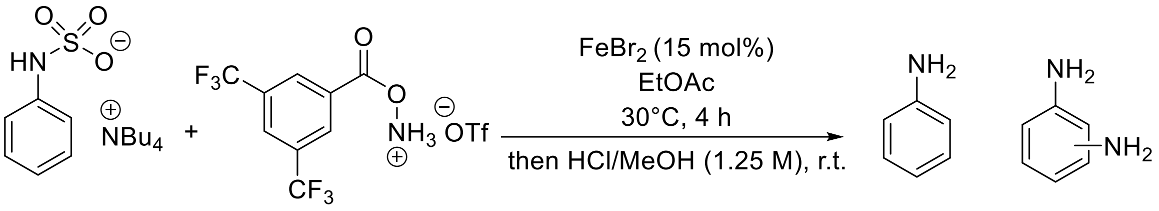

1a (1 equiv) **2a** (1 equiv) **4a**

Entry[a]	Concentration	NMR Yield (Aniline)[c]	NMR yield (4a)[c]	*ortho*:*para* (4a)[b]
1	0.5	45	40	>20:1
2	0.25	43	34	>20:1
3	0.1	40	23	>20:1
4	0.05	60	14	>20:1
5	0.25[d]	45	26	>20:1

[a] Reactions conducted on 0.1 mmol scale. [b] Regioisomer ratios were determined by ^{1}H NMR. [c] Yields were determined by ^{1}H NMR analysis of the crude reaction mixture using 1,2-dimethoxyethane as an internal standard. [d] Reaction time was 24 h.

Table S2: Evaluation of Fe(II) source

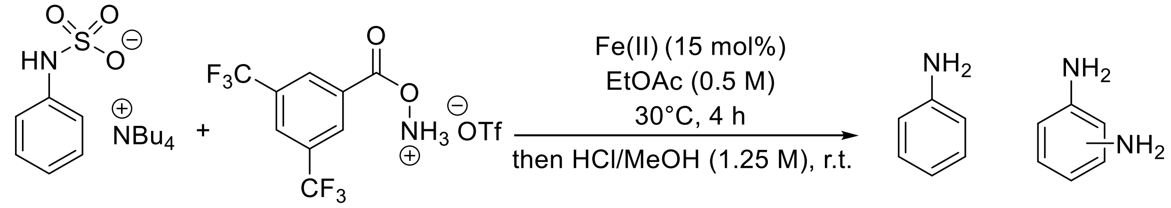

1a (1 equiv) **2a** (1 equiv) **4a**

Entry[a]	Fe(II) Source	NMR Yield (Aniline)[c]	NMR Yield (4a)[c]	*ortho*:*para* (4a)[b]
1	$FeCl_2$	79	0	-
2	$Fe(OAc)_2$	74	5	>20:1
3	$Fe(SO_4)_2{\cdot}7H_2O$	85	0	-
4	Ferrocene	33	38	>20:1
5	$FeBr_2$	45	40	>20:1
6	$FeBr_2$[d]	41	37	>20:1
7	$FeBr_2$[e]	37	37	>20:1

[a] Reactions conducted on 0.1mmol scale. [b] Regioisomer ratios were determined by ^{1}H NMR. [c] Yields were determined by ^{1}H NMR analysis of the crude reaction mixture using 1,2-dimethoxyethane as an internal standard. [d] Reaction run using anhydrous EtOAc under an argon atmosphere. [e] Reaction run using anhydrous, degassed EtOAc under an argon atmosphere.

Table S3: Solvent evaluation

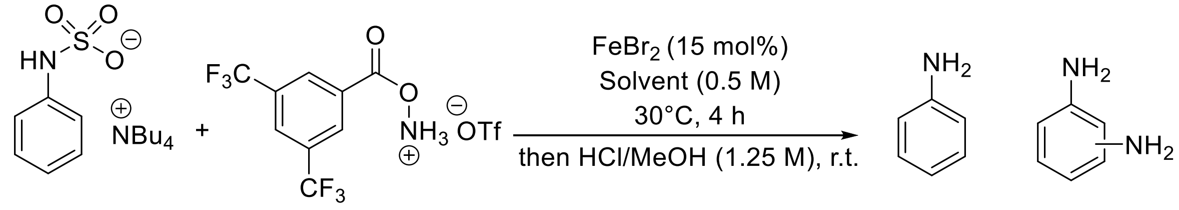

1a (1 equiv) **2a** (1 equiv) **4a**

Entry[a]	Solvent	NMR Yield (Aniline)[c]	NMR Yield (4a)[c]	*ortho*:*para* (4a)[b]
1	EtOAc	45	40	>20:1
2	TFE:H_2O (2:1)	34	35	4:1
3	MeCN:H_2O (2:1)	35	28	4:1
4	EtOAc:H_2O (2:1)	36	43	10:1
5	THF:H_2O (2:1)	64	8	4:1
6	DCM:H_2O (2:1)	15	20	19:1
7	DMA	46	16	7:1
8	TFE	18	45	>20:1
9	HFIP	21	47	>20:1

[a] Reactions conducted on 0.1 mmol scale. [b] Regioisomer ratios were determined by ^{1}H NMR. [c] Yields were determined by ^{1}H NMR analysis of the crude reaction mixture using 1,2-dimethoxyethane as an internal standard.

Table S4: Evaluation of aminating agents for NH_2 transfer

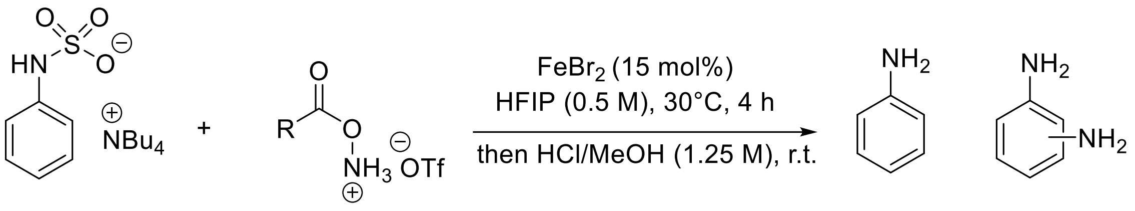

1a **2a-2f** **4a**

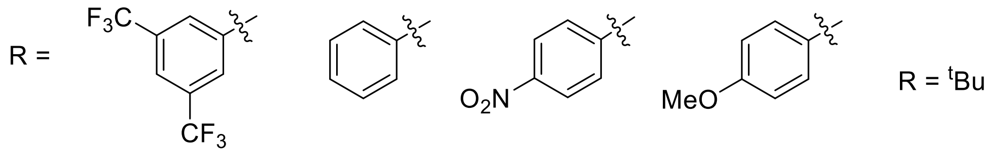

2a **2b** **2c** **2d** **2e**

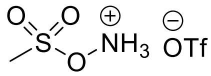

2f

Entry[a]	Aminating Agent	Equivalents of Aminating Agent	NMR Yield (Aniline)[c]	NMR Yield (4a)[c]	*ortho*:*para* (4a)[b]
1	**2a**	1	21	47	>20:1
2	**2b**	1	27	38	>20:1
3	**2c**	1	29	40	>20:1
4	**2d**	1	20	50	>20:1
5	**2d**	1.5	5	60	>20:1
6	**2d**	2	3	42	>20:1
7	**2e**	1	32	38	>20:1
8	**2f**	1	61	6	>20:1

[a] Reactions conducted on 0.1 mmol scale. [b] Regioisomer ratios were determined by ^1H NMR. [c] Yields were determined by ^1H NMR analysis of the crude reaction mixture using 1,2-dimethoxyethane as an internal standard.

Table S5: Evaluation of aminating agents for NHMe transfer

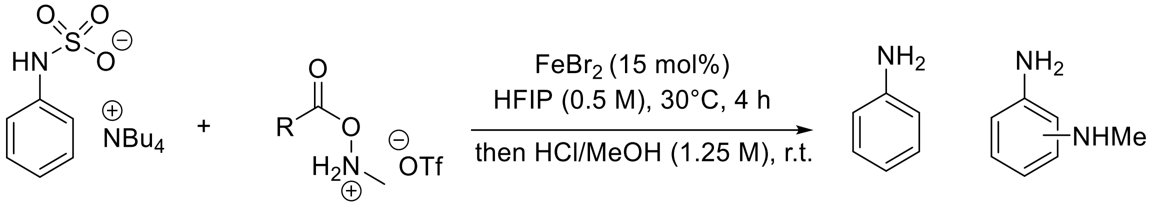

1a **3b-d** **5a**

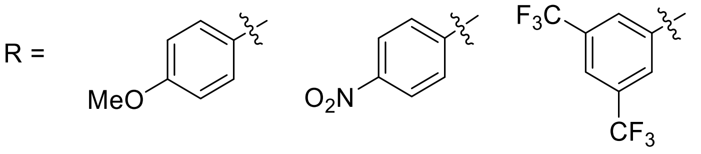

3d **3b** **3c**

Entry[a]	Aminating Agent	Equivalents of Aminating Agent	NMR Yield (Aniline)[c]	NMR Yield (5a)[c]	*ortho:para* (5a)[b]
1	**3d**	1	21	55	17:1
2	**3d**	1.5	7	68	17:1
3	**3b**	1	30	47	15:1
4	**3b**	1.5	10	52	12:1
5	**3c**	1	25	38	12:1
6	**3c**	1.5	10	45	10:1

[a] Reactions conducted on 0.1 mmol scale. [b] Regioisomer ratios were determined by ^{1}H NMR. [c] Yields were determined by ^{1}H NMR analysis of the crude reaction mixture using 1,2-dimethoxyethane as an internal standard.

Table S6: Iron free reactions at elevated temperature

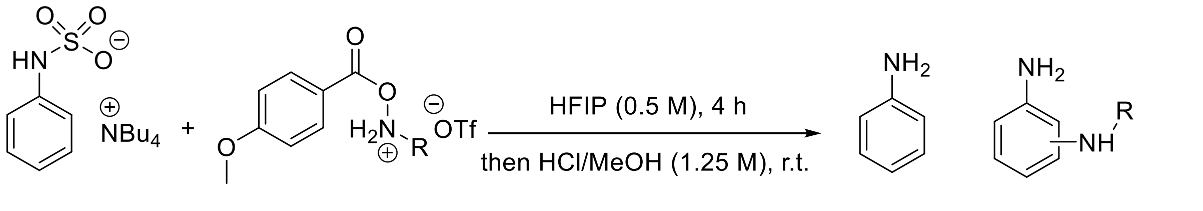

1a **2d (R = H) or 3d (R = Me)** **4a (R = H) or 5a (R = Me)**

(1.5 equiv)

Entry[a]	Aminating Agent	Temperature (℃)	NMR Yield (Aniline)[c]	NMR Yield (4a/5a)[c]	*ortho*:*para* (4a/5a)[b]
1	**2d**	60	67	<5	-
2	**2d**	80	75	<5	-
3	**3d**	60	61	0	-
4	**3d**	80	37	0	-

[a] Reactions conducted on 0.1 mmol scale. [b] Regioisomer ratios were determined by ^{1}H NMR. [c] Yields were determined by ^{1}H NMR analysis of the crude reaction mixture using 1,2-dimethoxyethane as an internal standard.

Table S7: Iron free reactions at elevated temperature with a more reactive substrate

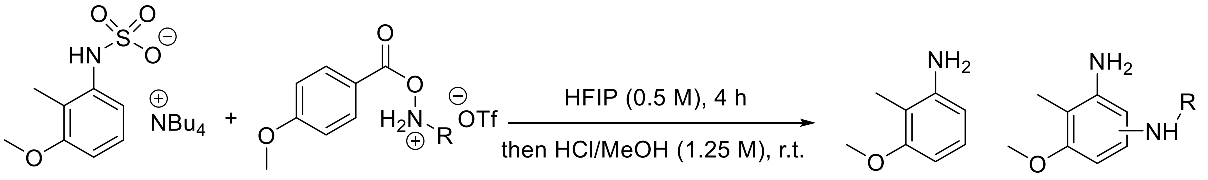

1l **2d (R = H) or 3d (R = Me)** **4h/5n**

(1 equiv) (1.5 equiv)

Entry[a]	Aminating Agent	Temperature (℃)	NMR Yield (Aniline)[c]	NMR Yield (4h/5n)[c]	*ortho*:*para* (4h/5n)[b]
1	**2d**	60	42	22	>20:1
2	**2d**	80	46	15	>20:1
3[d]	**3d**	60	-	-	-
4[d]	**3d**	80	-	-	-

[a] Reactions conducted on 0.1 mmol scale. [b] Regioisomer ratios were determined by ^{1}H NMR. [c] Yields were determined by ^{1}H NMR analysis of the crude reaction mixture using 1,2-dimethoxyethane as an internal standard. [d] ^{1}H NMR analysis revealed a complex mixture of products.

3. General Procedures

General Procedure 1 (GP-1): Synthesis of tetrabutylammonium sulfamate salts

Chlorosulfonic acid (1 equiv) was added dropwise *via* pipette to a stirred solution of the corresponding aniline (1 equiv) in CH_2Cl_2:NEt_3 (1.5:1, 0.36 M) at 0°C. The resulting suspension was stirred at room temperature for 1 h. Sodium hydroxide (10% aqueous solution) was then added and the aqueous layer was washed with CH_2Cl_2 three times and Et_2O once. Tetrabutylammonium hydrogensulfate (0.7-0.95 equiv) was added to the aqueous layer which was then extracted with CH_2Cl_2 three times. The combined organics were then washed with water five times, dried ($MgSO_4$), filtered and concentrated *in vacuo* to yield the tetrabutylammonium sulfamate salts which were used without further purification.

General Procedure 2 (GP-2): Synthesis of benzoyloxycarbamates

This procedure is adapted from those of Morandi,[1] Ritter[2] and Jiao.[3]

Acid chloride (1 or 1.1 equiv) was added dropwise or portion-wise to a stirred solution of *tert*-butyl *N*-hydroxycarbamate (1 equiv) and triethylamine (1.1 equiv) in Et_2O (0.2 M) or CH_2Cl_2 (0.5 M) at 0°C. The resulting suspension was stirred under argon at room temperature for 14 h. The organics were then washed with aqueous $NaHCO_3$, water and brine, dried ($MgSO_4$), filtered and concentrated *in vacuo*. The resulting crude benzoyloxycarbamate residue was either used directly in the next step or purified by silica gel chromatography.

General Procedure 3 (GP-3): Synthesis of NH_2 transfer and NHMe transfer reagents from benzoyloxycarbamates

This procedure is adapted from those of Morandi,[1] Ritter[2] and Jiao.[3]

Triflic acid (1.1 equiv) was added dropwise to a solution of the corresponding benzoyloxycarbamate (1 equiv) in Et_2O (0.2 M) at 0°C. The solution was then stirred at room temperature for 2 h. The resulting precipitate was then isolated by filtration and washed with Et_2O to give the final product which was used without further purification.

General Procedure 4 (GP-4): Amination of tetrabutylammonium sulfamate salts with NH_2 transfer reagents **2a-2d** or NHMe transfer reagent **3a**

A suspension of tetrabutylammonium sulfamate salt (1 equiv), NH_2 or NHMe transfer reagent (1 or 1.5 equiv) and $FeBr_2$ (0.015 equiv) in HFIP (0.5 M) was stirred at 30°C for 4 h. The solvent was then removed under a stream of air and HCl in MeOH (1.25 M, 5 equiv) was added. The resulting solution was then stirred at room temperature for 1 h. The solvent was removed under a stream of air and aqueous NaOH was added. The aqueous layer was then extracted with $CHCl_3$ three times. The combined organics were then dried ($MgSO_4$) and concentrated. The crude residue was then purified by silica gel chromatography to give the final product.

4. Assignment of Product Regiochemistry

In most cases, the coupling pattern in the ^1H NMR spectrum or comparison to literature data was sufficient to assign product regiochemistry. For fluorinated substrates, the coupling pattern in the ^{13}C NMR spectrum was also used to confirm product regiochemistry.

Where a 3-substituted or 2,3-disubstituted substrate was used, the expected coupling pattern is the same for both *ortho* and *para* regioisomers. In these cases, if the 3-substituent was a carbon, a strong HMBC signal to the *para* proton was observed, allowing the product to be assigned as *ortho*:

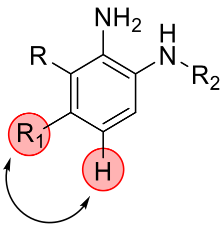

Strong HMBC when R_1 = C

If the 3-substituent was not a carbon, the products were converted into the corresponding benzimidazoles to confirm *ortho* regiochemistry:

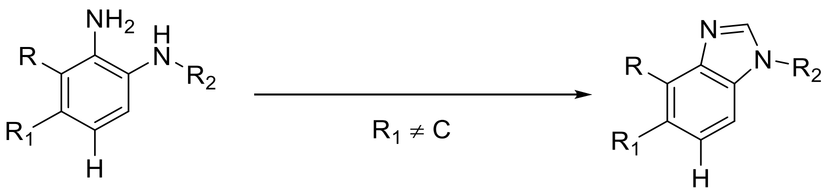

5. Limitations

The following substrates showed no conversion to the desired product after being subject to the NHMe or NH_2 transfer conditions:

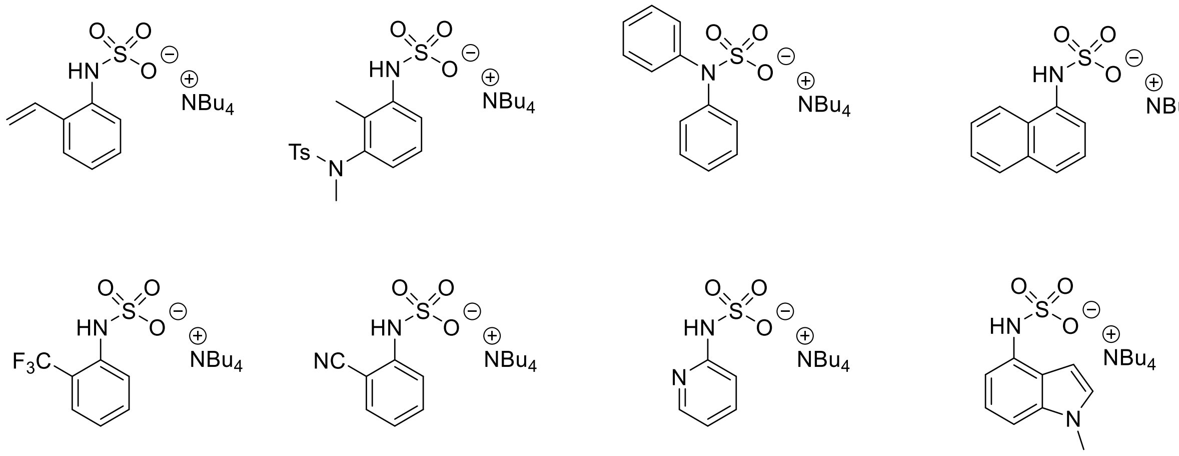

6. Tetrabutylammonium Sulfamate Salt Synthesis

Tetrabutylammonium phenylsulfamate (1a)

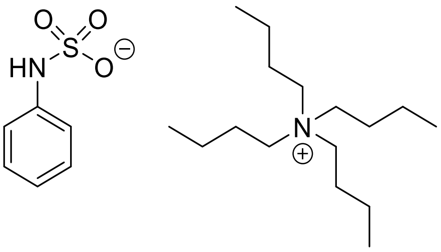

Aniline (1.40 g, 1.37 mL, 15.1 mmol, 1 equiv), chlorosulfonic acid (1.75 g, 0.98 mL, 15.0 mmol, 1 equiv) and tetrabutylammonium hydrogen sulfate (4.08 g, 12.0 mmol, 0.8 equiv) were subject to **GP-1** to give the title compound (2.30 g, 5.54 mmol, 46%) as a white solid.

^{1}H NMR (400 MHz, $CDCl_3$) δ 7.18 – 7.07 (m, 4H), 6.77 (tt, *J* = 5.6, 2.9 Hz, 1H), 6.50 (br s, 1H), 3.32 – 2.91 (m, 8H), 1.51 (tt, *J* = 8.1, 5.9 Hz, 8H), 1.34 (h, *J* = 7.3 Hz, 8H), 0.92 (t, *J* = 7.3 Hz, 12H); ^{13}C NMR (101 MHz, $CDCl_3$) δ 142.7, 128.6, 119.8, 117.0, 58.4, 23.9, 19.6, 13.67.

Data match reported values.[4]

Tetrabutylammonium *o*-tolylsulfamate (1b)

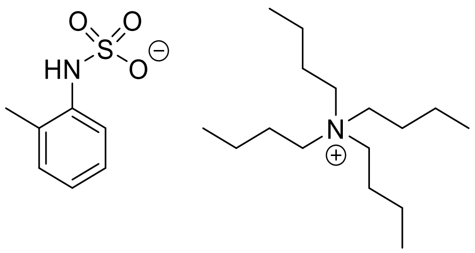

o-Toluidine (2.14 g, 2.12 mL, 20.0 mmol, 1 equiv), chlorosulfonic acid (2.34 g, 1.33 mL, 20.0 mmol, 1 equiv) and tetrabutylammonium hydrogen sulfate (4.76 g, 14.0 mmol, 0.7 equiv) were subject to **GP-1** to give the title compound (4.84 g, 11.3 mmol, 81%) as a white solid.

^{1}H NMR (400 MHz, $CDCl_3$) δ 7.65 (d, *J* = 8.1 Hz, 1H), 7.04 (t, *J* = 7.8 Hz, 1H), 6.98 (d, *J* = 7.5 Hz, 1H), 6.71 (t, *J* = 7.4 Hz, 1H), 5.93 (br s, 1H), 3.40 – 2.88 (m, 8H), 2.11 (s, 3H), 1.57 – 1.46 (m, 8H), 1.34 (h, *J* = 7.4 Hz, 8H), 0.93 (t, *J* = 7.2 Hz, 12H). ^{13}C NMR (101 MHz, $CDCl_3$) δ 140.8, 129.8, 126.6, 123.6, 119.8, 117.1, 58.4, 23.9, 19.6, 17.7, 13.7. HRMS (ESI) calcd for $[C_7H_8NO_3S]^-$: 186.0230. Found: 186.0234.

Tetrabutylammonium (2-isopropylphenyl)sulfamate (1c)

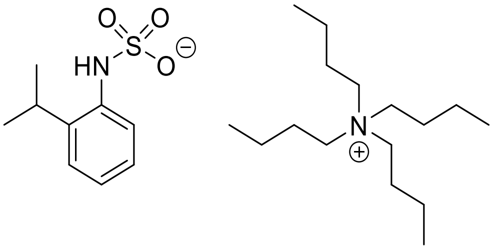

2-Isopropylaniline (2.02 g, 2.12 mL, 15.0 mmol, 1 equiv), chlorosulfonic acid (1.75 g, 0.98 mL, 15.0 mmol, 1 equiv) and tetrabutylammonium hydrogen sulfate (4.08 g, 12.0 mmol, 0.8 equiv) were subject to **GP-1** to give the title compound (3.65 g, 7.99 mmol, 67%) as a white solid.

^{1}H NMR (400 MHz, $CDCl_3$) δ 7.74 (d, *J* = 8.1 Hz, 1H), 7.13 (d, *J* = 7.8 Hz, 1H), 7.07 (t, *J* = 7.7 Hz, 1H), 6.85 (t, *J* = 7.5 Hz, 1H), 3.29 – 3.22 (m, 8H), 2.99 (hept, *J* = 7.1 Hz, 1H), 1.61 (h, *J* = 7.8 Hz, 8H), 1.41 (h, *J* = 7.4 Hz, 8H), 1.23 (d, *J* = 6.7 Hz, 6H), 0.99 (t, *J* = 7.3 Hz, 12H). ^{13}C NMR (101 MHz, $CDCl_3$) δ 139.4, 134.7, 126.1, 124.6, 120.5, 118.4, 58.7, 27.2, 24.0, 22.8, 19.7, 13.7.

Data match reported values.[4]

Tetrabutylammonium (2-*tert*-butylphenyl)sulfamate (1d)

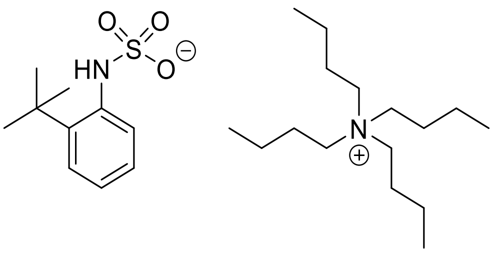

2-*tert*-Butylaniline (2.24 g, 2.33 mL, 15.0 mmol, 1 equiv), chlorosulfonic acid (1.75 g, 0.98 mL, 15.0 mmol, 1 equiv) and tetrabutylammonium hydrogen sulfate (4.08 g, 12.0 mmol, 0.8 equiv) were subject to **GP-1** to give the title compound (2.34 g, 4.97 mmol, 42%) as an off-white solid.

^{1}H NMR (400 MHz, $CDCl_3$) δ 7.76 (dd, *J* = 8.2, 1.4 Hz, 1H), 7.16 (dd, *J* = 8.0, 1.5 Hz, 1H), 7.06 – 6.95 (m, 1H), 6.73 (td, *J* = 7.6, 1.4 Hz, 1H), 6.29 (br s, 1H), 3.23 – 3.03 (m, 8H), 1.61 – 1.43 (m, 8H), 1.36 (s, 9H), 1.34 – 1.25 (m, 8H), 0.89 (t, *J* = 7.3 Hz, 12H). ^{13}C NMR (101 MHz, $CDCl_3$) δ 141.1, 135.2, 126.4, 125.6, 119.9, 119.2, 58.4, 33.9, 30.5, 23.9, 19.6, 13.6. HRMS (ESI) calcd for $[C_{10}H_{14}NO_3S]^-$: 228.0700. Found: 228.0697.

Tetrabutylammonium *m*-tolylsulfamate (1e)

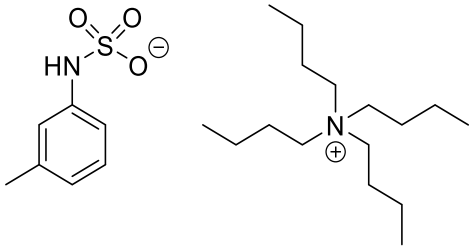

m-Toluidine (2.14 g, 2.14 mL, 20.0 mmol, 1 equiv), chlorosulfonic acid (2.34 g, 1.33 mL, 20.0 mmol, 1 equiv) and tetrabutylammonium hydrogen sulfate (4.76 g, 14.0 mmol, 0.7 equiv) were subject to **GP-1** to give the title compound (4.72 g, 11.0 mmol, 79%) as a white solid.

^{1}H NMR (400 MHz, $CDCl_3$) δ 7.06 – 6.88 (m, 3H), 6.60 (d, *J* = 7.3 Hz, 1H), 6.44 (br s, 1H), 3.24 – 3.09 (m, 8H), 2.23 (s, 3H), 1.52 (dt, *J* = 16.0, 8.0 Hz, 8H), 1.34 (h, *J* = 7.3 Hz, 8H), 0.97 – 0.85 (m, 12H). ^{13}C NMR (101 MHz, $CDCl_3$) δ 142.6, 138.1, 128.4, 120.8, 117.7, 114.3, 58.4, 23.9, 21.6, 19.6, 13.7. HRMS (ESI) calcd for $[C_7H_8NO_3S]^-$:186.0230. Found: 186.0221.

Tetrabutylammonium (3-isopropylphenyl)sulfamate (1f)

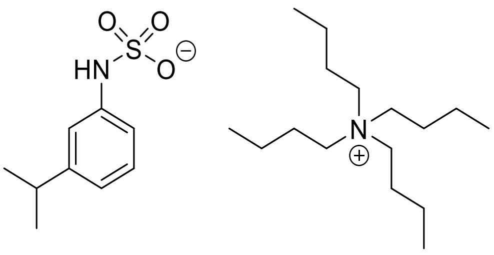

3-Isopropylaniline (2.02 g, 2.02 mL, 15.0 mmol, 1 equiv), chlorosulfonic acid (1.75 g, 0.98 mL, 15.0 mmol, 1 equiv) and tetrabutylammonium hydrogen sulfate (4.08 g, 12.0 mmol, 0.8 equiv) were subject to **GP-1** to give the title compound (3.88 g, 8.50 mmol, 71%) as a white solid.

^{1}H NMR (400 MHz, $CDCl_3$) δ 7.00 (m, 2H), 6.78 (s, 1H), 6.62 (d, *J* = 8.6 Hz, 1H), 6.16 (br s, 1H), 3.26 – 2.89 (m, 8H), 2.73 (h, *J* = 6.9 Hz, 1H), 1.46 (h, *J* = 7.8 Hz, 8H), 1.27 (h, *J* = 7.3 Hz, 8H), 1.13 (d, *J* = 6.8, 6H), 0.99 – 0.57 (m, 12H). ^{13}C NMR (101 MHz, $CDCl_3$) δ 149.2, 142.5, 128.4, 117.8, 115.4, 114.5, 58.2, 34.0, 23.8, 19.5, 13.6. HRMS (ESI) calcd for $[C_9H_{12}NO_3S]^-$: 214.0543. Found: 214.0549.

2-(Hex-1-yn-1-yl)aniline

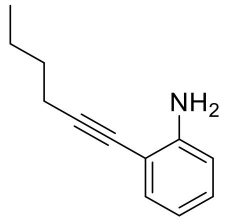

A solution of 2-iodoaniline (3.14 g, 14.3 mmol, 1 equiv), bis(triphenylphosphine)palladium(II) dichloride (211 mg, 0.300 mmol, 0.02 equiv) and copper(I) iodide (28.6 mg, 0.150 mmol, 0.01 equiv) in degassed triethylamine (30 mL) was stirred at 30℃ for 10 min. 1-Hexyne (2.28 mL, 1.64 g, 20.0 mmol, 1.4 equiv) was then added and the solution was stirred at 30℃ for a further 16 h. The reaction was then quenched with water (20 mL) and the aqueous layer was extracted with ethyl acetate (3 x 30 mL). The combined organics were then washed with brine, dried ($MgSO_4$), filtered and concentrated *in vacuo*. The crude residue was then purified by silica gel chromatography (eluent: Pet. Ether:EtOAc, 100:0 – 95:5) to give the title compound (2.16 g, 12.5 mmol, 87%) as an orange liquid.

^{1}H NMR (500 MHz, $CDCl_3$) δ 7.27 (dd, *J* = 7.6, 1.6 Hz, 1H), 7.14 – 7.05 (m, 1H), 6.78 – 6.58 (m, 2H), 4.18 (s, 2H), 2.50 (t, *J* = 7.1 Hz, 2H), 1.70 – 1.59 (m, 2H), 1.59 – 1.46 (m, 2H), 0.98 (t, *J* =

7.3 Hz, 3H). ^{13}C NMR (126 MHz, $CDCl_3$) δ 147.6, 132.0, 128.8, 117.9, 114.1, 109.0, 95.8, 77.0, 31.1, 22.1, 19.4, 13.7.

Data match literature values.[5]

Tetrabutylammonium (2-(hex-1-yn-1-yl)phenyl)sulfamate (1g)

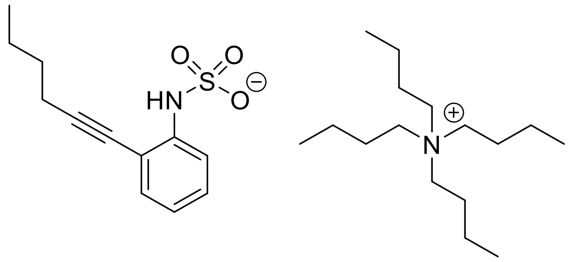

2-(Hex-1-yn-1-yl)aniline (1.21 g, 6.98 mmol, 1 equiv), chlorosulfonic acid (815 mg, 466 µL, 7.00 mmol, 1 equiv) and tetrabutylammonium hydrogen sulfate (2.12 g, 6.30 mmol, 0.9 equiv) were subject to **GP-1** to give the title compound (1.11 g, 2.24 mmol, 36%) as a yellow oil.

^{1}H NMR (700 MHz, MeOD-*d*$_4$) δ 7.60 (dd, *J* = 8.3, 1.2 Hz, 1H), 7.26 (dd, *J* = 7.7, 1.5 Hz, 1H), 7.21 (ddd, *J* = 8.6, 7.4, 1.6 Hz, 1H), 6.84 (td, *J* = 7.5, 1.1 Hz, 1H), 3.26 – 3.15 (m, 8H), 2.52 (t, *J* = 7.1 Hz, 2H), 1.65 (m, 8H), 1.65 (m, 2H), 1.55 (dt, *J* = 14.8, 7.3 Hz, 2H), 1.42 (h, *J* = 7.4 Hz, 8H), 1.03 (t, *J* = 7.4 Hz, 12H), 1.00 (t, *J* = 7.4 Hz, 3H).^{13}C NMR (176 MHz, MeOD-*d*$_4$) δ 142.2, 131.0, 128.2, 119.8, 115.5, 110.8, 96.2, 75.9, 58.1, 30.7, 23.4, 21.7, 19.3, 18.5, 12.6, 12.6. HRMS (ESI) calcd for $[C_{12}H_{14}NO_3S]^-$: 252.0700. Found: 252.0693.

Tetrabutylammonium *p*-tolylsulfamate (1h)

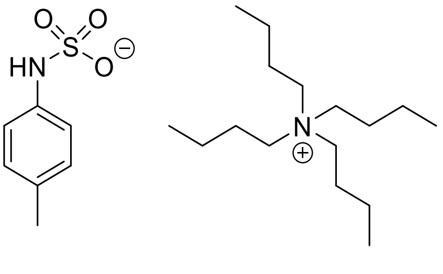

p-Toluidine (2.14 g, 20.0 mmol, 1 equiv), chlorosulfonic acid (2.34 g, 1.33 mL, 20.0 mmol, 1 equiv) and tetrabutylammonium hydrogen sulfate (4.76 g, 14.0 mmol, 0.7 equiv) were subject to **GP-1** to give the title compound (4.69 g, 11.0 mmol, 78%) as a white solid.

^{1}H NMR (400 MHz, $CDCl_3$ δ 6.94 (d, *J* = 8.4 Hz, 2H), 6.85 (d, *J* = 8.2 Hz, 2H), 6.11 (br s, 1H), 3.17 – 2.94 (m, 8H), 2.13 (s, 3H), 1.48 – 1.33 (m, 8H), 1.29 – 1.17 (m, 8H), 0.84 (t, *J* = 7.3 Hz, 12H). ^{13}C NMR (101 MHz, $CDCl_3$) δ 140.2, 129.0, 129.0, 117.3, 58.1, 23.7, 20.5, 19.5, 13.6. HRMS (ESI) calcd for $[C_7H_8NO_3S]^-$: 186.0230. Found: 186.0229.

Tetrabutylammonium (5,6,7,8-tetrahydronaphthalen-1-yl)sulfamate (1i)

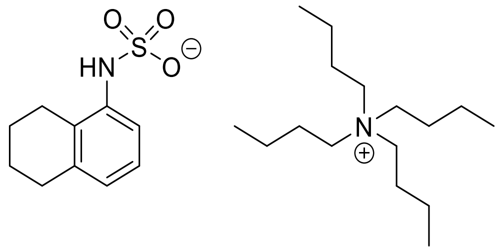

5,6,7,8-tetrahydro-1-naphthylamine (1.47 g, 10.0 mmol, 1 equiv), chlorosulfonic acid (1.32 g, 0.67 mL, 10.0 mmol, 1 equiv) and tetrabutylammonium hydrogen sulfate (2.72 g, 8.01 mmol, 0.8 equiv) were subject to **GP-1** to give the title compound (3.74 g, 7.98 mmol, 100%) as a white solid.

^{1}H NMR (400 MHz, $CDCl_3$) δ 7.45 (d, *J* = 8.0 Hz, 1H), 6.89 (t, *J* = 7.8 Hz, 1H), 6.51 (d, *J* = 7.5 Hz, 1H), 5.85 (br s, 1H), 3.17 – 2.96 (m, 8H), 2.64 (t, *J* = 6.1 Hz, 2H), 2.43 – 2.31 (m, 2H), 1.73 (m, 2H), 1.67 (m, 2H), 1.45 (q, *J* = 7.9 Hz, 8H), 1.28 (h, *J* = 7.4 Hz, 8H), 0.88 (t, *J* = 7.3 Hz, 12H). ^{13}C NMR (100 MHz, $CDCl_3$) δ 140.4, 137.0, 125.5, 122.6, 120.9, 114.2, 58.3, 30.1, 24., 23.8, 23.2, 22.7, 19.6, 13.7. HRMS (ESI) calcd for $[C_{10}H_{12}NO_3S]^-$: 226.0543. Found: 226.0541.

Tetrabutylammonium (2,5-dimethylphenyl)sulfamate (1j)

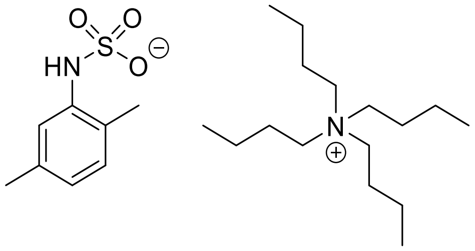

2,5-Dimethylaniline (1.21 g, 1.25 mL, 10.0 mmol, 1 equiv), chlorosulfonic acid (1.32 g, 0.67 mL, 10.0 mmol, 1 equiv) and tetrabutylammonium hydrogen sulfate (2.72 g, 8.01 mmol, 0.8 equiv) were subject to **GP-1** to give the title compound (2.93 g, 6.63 mmol, 83%) as a white solid.

^{1}H NMR (400 MHz, $CDCl_3$ δ 7.43 (d, *J* = 1.7 Hz, 1H), 6.81 (d, *J* = 7.6 Hz, 1H), 6.48 (dd, *J* = 7.2, 1.1 Hz, 1H), 5.80 (br s, 1H), 3.24 – 2.93 (m, 8H), 2.18 (s, 3H), 2.01 (s, 3H), 1.56 – 1.37 (m, 8H), 1.28 (h, *J* = 7.3 Hz, 8H), 0.88 (t, *J* = 7.3 Hz, 12H). ^{13}C NMR (101 MHz, $CDCl_3$) δ 140.5, 135.9, 129.5, 120.6, 120.4, 117.6, 58.2, 23.8, 21.5, 19.6, 17.3, 13.6. HRMS (ESI) calcd for $[C_8H_{10}NO_3S]^-$: 200.0387. Found: 200.0387.

Tetrabutylammonium (2,3-dimethylphenyl)sulfamate (1k)

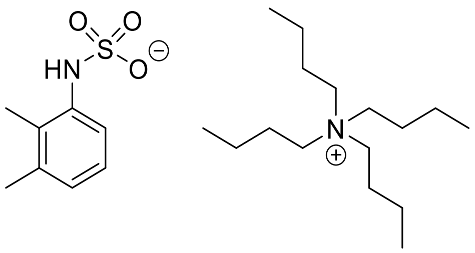

2,3-Dimethylaniline (1.82 g, 1.83 mL, 15.0 mmol, 1 equiv), chlorosulfonic acid (1.75 g, 0.98 mL, 15.0 mmol, 1 equiv) and tetrabutylammonium hydrogen sulfate (4.08 g, 12.0 mmol, 0.8 equiv) were subject to **GP-1** to give the title compound (3.99 g, 9.01 mmol, 75%) as a white solid.

^{1}H NMR (400 MHz, $CDCl_3$) δ 7.46 (d, *J* = 8.1 Hz, 1H), 6.85 (t, *J* = 7.8 Hz, 1H), 6.58 (d, *J* = 7.4 Hz, 1H), 5.76 (br s, 1H), 3.17 – 2.64 (m, 8H), 2.14 (s, 3H), 1.96 (s, 3H), 1.56 – 1.33 (m, 8H), 1.24 (h, *J* = 7.3 Hz, 8H), 0.85 (t, *J* = 7.3 Hz, 12H). ^{13}C NMR (101 MHz, $CDCl_3$) δ 140.6, 136.0, 125.5, 122.5, 122.1, 115.7, 58.1, 23.7, 20.8, 19.5, 13.6, 12.9. HRMS (ESI) calcd for $[C_8H_{10}NO_3S]^-$: 200.0387. Found: 200.0386.

Tetrabutylammonium (3-methoxyphenyl)sulfamate (1l)

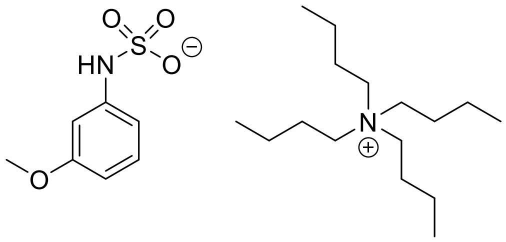

m-Anisidine (2.46 g, 2.24 mL, 20.0 mmol, 1 equiv), chlorosulfonic acid (2.34 g, 1.33 mL, 20.0 mmol, 1 equiv) and tetrabutylammonium hydrogen sulfate (4.76 g, 14.0 mmol, 0.7 equiv)

were subject to **GP-1** to give the title compound (4.08 g, 9.17 mmol, 66%) as an off-white solid.

^{1}H NMR (600 MHz, $CDCl_3$) δ 7.08 (t, *J* = 8.1 Hz, 1H), 6.82 (t, *J* = 2.3 Hz, 1H), 6.74 (dd, *J* = 8.0, 2.1, 1H), 6.41 (dd, *J* = 8.2, 2.5 Hz, 1H), 6.34 (br s, 1H), 3.77 (s, 3H), 3.30 – 3.18 (m, 8H), 1.61 (dq, *J* = 11.8, 8.0, 7.4 Hz, 8H), 1.42 (h, *J* = 7.4 Hz, 8H), 0.99 (t, *J* = 7.3 Hz, 12H). ^{13}C NMR (101 MHz, $CDCl_3$) δ 160.1, 144.0, 129.2, 109.7, 105.2, 103.0, 58.1, 55.0, 23.7, 19.5, 13.6. HRMS (ESI) calcd for $[C_7H_8NO_4S]^-$: 202.0180. Found: 202.0182.

Tetrabutylammonium (3-methoxy-2-methylphenyl)sulfamate (1m)

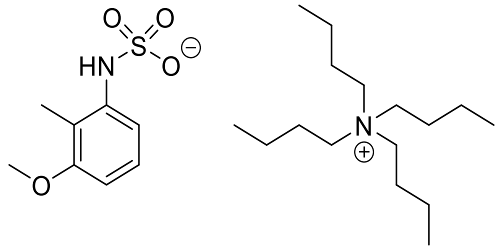

3-Methoxy-2-methylaniline (2.06 g, 15.0 mmol, 1 equiv), chlorosulfonic acid (1.75 g, 0.98 mL, 15.0 mmol, 1 equiv) and tetrabutylammonium hydrogen sulfate (4.08 g, 12.0 mmol, 0.8 equiv) were subject to **GP-1** to give the title compound (3.44 g, 7.50 mmol, 63%) as a white solid.

^{1}H NMR (400 MHz, $CDCl_3$) δ 7.25 (d, *J* = 8.3 Hz, 1H), 6.88 (t, *J* = 8.2 Hz, 1H), 6.28 (d, *J* = 8.1 Hz, 1H), 5.82 (br s, 1H), 3.64 (s, 3H), 3.13 – 2.83 (m, 8H), 1.88 (s, 3H), 1.44 – 1.32 (m, 8H), 1.22 (h, *J* = 7.3 Hz, 8H), 0.82 (t, *J* = 7.3 Hz, 12H). ^{13}C NMR (101 MHz, $CDCl_3$) δ 157.3, 141.7, 126.1, 111.5, 110.4, 102.5, 58.1, 55.5, 23.7, 19.5, 13.6, 8.9. HRMS (ESI) calcd for $[C_8H_{10}NO_4S]^-$: 216.0336. Found: 216.0342.

Tetrabutylammonium (3-difluoromethoxyphenyl)sulfamate (1n)

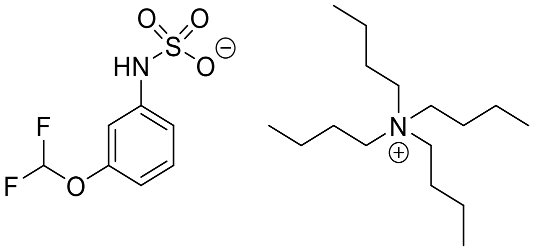

3-(Difluoromethoxy)aniline (2.39 g, 1.86 mL, 15.0 mmol, 1 equiv), chlorosulfonic acid (1.75 g, 0.98 mL, 15.0 mmol, 1 equiv) and tetrabutylammonium hydrogen sulfate (4.08 g, 12.0 mmol, 0.8 equiv) were subject to **GP-1** to give the title compound (3.48 g, 7.24 mmol, 60%) as a white solid.

^{1}H NMR (400 MHz, $CDCl_3$) δ 7.06 (t, *J* = 8.1 Hz, 1H), 7.00 (t, *J* = 2.2 Hz, 1H), 6.91 – 6.83 (m, 1H), 6.49 – 6.46 (m, 1H), 6.45 (t, *J* = 74.9 Hz, 1H), 3.25 – 2.83 (m, 8H), 1.60 – 1.39 (m, 8H), 1.30 (h, *J* = 7.3 Hz, 8H), 0.88 (t, *J* = 7.3 Hz, 12H). ^{13}C NMR (101 MHz, $CDCl_3$) δ 152.1 (t, *J* = 2.8 Hz), 144.6, 129.6, 116.4 (t, *J* = 256.9 Hz), 113.8, 109.5, 107.4, 58.3, 23.8, 19.5, 13.6. ^{19}F NMR (376 MHz, $CDCl_3$) δ -80.89. HRMS (ESI) calcd for $[C_7H_6F_2NO_3S]^-$: 237.9991. Found: 237.9994.

Tetrabutylammonium (5-bromo-2-methylphenyl)sulfamate (1o)

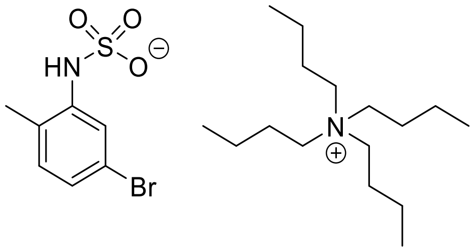

5-Bromo-2-methylaniline (2.79 g, 1.87 mL, 15.0 mmol, 1 equiv), chlorosulfonic acid (1.75 g, 0.98 mL, 15.0 mmol, 1 equiv) and tetrabutylammonium hydrogen sulfate (4.08 g, 12.0 mmol, 0.8 equiv) were subject to **GP-1** to give the title compound (3.88 g, 7.64 mmol, 64%) as a white solid.

^{1}H NMR (400 MHz, $CDCl_3$) δ 7.71 (s, 1H), 6.76 (d, *J* = 8.1 Hz, 1H), 6.71 (d, *J* = 8.2 Hz, 1H), 5.86 (br s, 1H), 3.28 – 2.64 (m, 8H), 1.94 (s, 3H), 1.51 – 1.35 (m, 8H), 1.23 (h, *J* = 7.5 Hz, 8H), 0.83 (t, *J* = 7.3 Hz, 12H). ^{13}C NMR (101 MHz, $CDCl_3$) δ 142.2, 131.0, 122.1, 122.0, 119.9, 118.8, 58.2, 23.7, 19.5, 17.2, 13.6. HRMS (ESI) calcd for $[C_7H_7BrNO_3S]^-$:263.9335. Found: 263.9333.

Tetrabutylammonium (3-chloro-2-methylphenyl)sulfamate (1p)

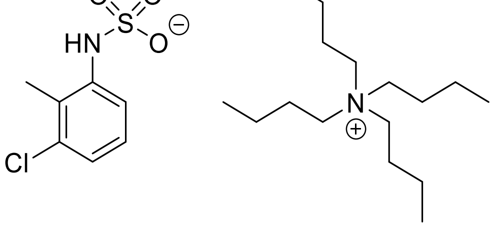

3-Chloro-2-methylaniline (2.11 g, 1.78 mL, 15.0 mmol, 1 equiv), chlorosulfonic acid (1.75 g, 0.98 mL, 15.0 mmol, 1 equiv) and tetrabutylammonium hydrogen sulfate (4.08 g, 12.0 mmol, 0.8 equiv) were subject to **GP-1** to give the title compound (3.14 g, 6.78 mmol, 56%) as a white solid.

^{1}H NMR (400 MHz, $CDCl_3$) δ 7.52 (dd, *J* = 8.2, 1.2 Hz, 1H), 6.89 (t, *J* = 8.1 Hz, 1H), 6.76 (dd, *J* = 8.0, 1.1 Hz, 1H), 5.66 (br s, 1H), 3.30 – 2.67 (m, 8H), 2.11 (s, 3H), 1.59 – 1.31 (m, 8H), 1.26 (h, *J* = 7.4 Hz, 8H), 0.85 (t, *J* = 7.3 Hz, 12H). ^{13}C NMR (101 MHz, $CDCl_3$) δ 142.3, 133.8, 126.7, 121.7, 120.6, 115.7, 58.3, 23.7, 19.5, 13.8, 13.6. HRMS (ESI) calcd for $[C_7H_7ClNO_3S]^-$: 219.9841. Found: 219.9839.

Tetrabutylammonium (3-fluoro-2-methylphenyl)sulfamate (1q)

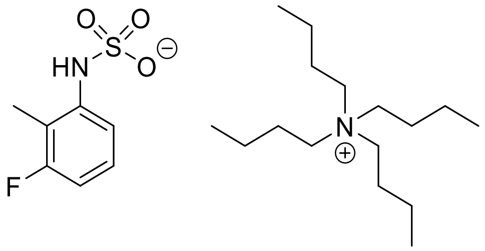

3-Fluoro-2-methylaniline (1.88 g, 1.70 mL, 15.0 mmol, 1 equiv), chlorosulfonic acid (1.75 g, 0.98 mL, 15.0 mmol, 1 equiv) and tetrabutylammonium hydrogen sulfate (4.08 g, 12.0 mmol, 0.8 equiv) were subject to **GP-1** to give the title compound (3.27 g, 7.32 mmol, 61%) as a white solid.

^{1}H NMR (400 MHz, $CDCl_3$) δ 7.33 (d, *J* = 8.2 Hz, 1H), 6.84 (td, *J* = 8.1, 6.5 Hz, 1H), 6.35 (t, *J* = 9.0 Hz, 1H), 5.87 (br s, 1H), 3.13 – 2.76 (m, 8H), 1.87 (d, *J* = 1.7 Hz, 3H), 1.46 – 1.30 (m, 8H), 1.19 (h, *J* = 7.3 Hz, 8H), 0.79 (t, *J* = 7.3 Hz, 12H). ^{13}C NMR (101 MHz, $CDCl_3$) δ 160.9 (d, *J* = 240.2 Hz), 142.4 (d, *J* = 6.3 Hz), 126.5 (d, *J* = 10.2 Hz), 112.3 (d, *J* = 2.7 Hz), 110.3 (d, *J* = 18.4 Hz), 106.0 (d, *J* = 23.2 Hz), 58.2, 23.7, 19.5, 13.5, 8.3 (d, *J* = 6.2 Hz). ^{19}F NMR (376 MHz, $CDCl_3$) δ -117.76. HRMS (ESI) calcd for $[C_7H_7FNO_3S]^-$: 204.0136 Found: 204.0134.

Tetrabutylammonium (3-fluorophenyl)sulfamate (1r)

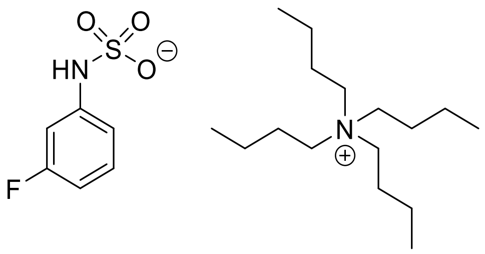

3-Fluoroaniline (1.11 g, 0.96 mL, 10.0 mmol, 1 equiv), chlorosulfonic acid (1.32 g, 0.67 mL, 10.0 mmol, 1 equiv) and tetrabutylammonium hydrogen sulfate (2.72 g, 8.0 mmol, 0.8 equiv) were subject to **GP-1** to give the title compound (1.75 g, 4.05 mmol, 51%) as a white solid.

^{1}H NMR (400 MHz, $CDCl_3$) δ 7.06 (br s, 1H), 6.98 – 6.89 (m, 2H), 6.68 (ddd, *J* = 8.1, 2.1, 0.9 Hz, 1H), 6.33 (tdd, *J* = 8.4, 2.5, 0.9 Hz, 1H), 3.24 – 2.81 (m, 8H), 1.48 – 1.32 (m, 8H), 1.22 (h, *J* = 7.3 Hz, 8H), 0.80 (t, *J* = 7.3 Hz, 12H). ^{13}C NMR (101 MHz, $CDCl_3$) δ 163.3 (d, *J* = 241.5 Hz), 144.7 (d, *J* = 11.1 Hz), 129.4 (d, *J* = 9.8 Hz), 112.2 (d, *J* = 2.4 Hz), 105.6 (d, *J* = 21.5 Hz), 103.5 (d, *J* = 25.6 Hz), 58.1, 23.7, 19.5, 13.5. ^{19}F NMR (376 MHz, $CDCl_3$) δ -113.59. HRMS (ESI) calcd for $[C_6H_5FNO_3S]^-$: 189.9980. Found: 189.9986.

Tetrabutylammonium (2-bromo-3-methylphenyl)sulfamate (1s)

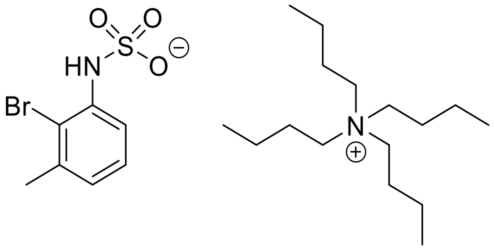

2-Bromo-3-methylaniline (2.38 g, 1.60 mL, 12.8 mmol, 1 equiv), chlorosulfonic acid (1.54 g, 0.86 mL, 12.8 mmol, 1 equiv) and tetrabutylammonium hydrogen sulfate (3.49 g, 10.3 mmol, 0.8 equiv) were subject to **GP-1** to give the title compound (2.82 g, 5.56 mmol, 54%) as a white solid.

^{1}H NMR (400 MHz, $CDCl_3$) δ 7.47 (d, *J* = 8.2 Hz, 1H), 6.87 (t, *J* = 7.8 Hz, 1H), 6.56 (br s, 1H), 6.53 (d, *J* = 7.5 Hz, 1H), 2.96 (m, 8H), 2.17 (s, 3H), 1.43 – 1.26 (m, 8H), 1.15 (h, *J* = 7.4 Hz, 8H), 0.76 (t, *J* = 7.3 Hz, 12H). ^{13}C NMR (101 MHz, $CDCl_3$) δ 140.2, 137.5, 127.2, 121.3, 114.7, 112.8, 58.0, 23.8, 23.6, 19.5, 13.5. HRMS (ESI) calcd for $[C_7H_7BrNO_3S]^-$:263.9335. Found: 263.9329.

***N*-Allylaniline**

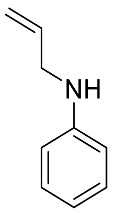

Allyl bromide (3.03 g, 2.16 mL, 25.0 mmol, 1 equiv) was added dropwise to a suspension of aniline (2.56 g, 2.51 mL, 27.5 mmol, 1.1 equiv) and K_2CO_3 (4.15 g, 30.0 mmol, 1.2 equiv) in DMF (50 mL) at 0°C. The reaction was allowed to warm to room temperature and was stirred for 20 h. The reaction was then diluted with aqueous NH_4Cl and the aqueous layer was extracted with ethyl acetate (3 x 50 mL). The combined organics were then dried ($MgSO_4$), filtered and concentrated *in vacuo.* The crude residue was then purified silica gel chromatography (eluent: Pet. Ether:EtOAc, 96:4) to give the title compound (1.37 g, 10.3 mmol, 41%) as a colourless liquid.

^{1}H NMR (400 MHz, $CDCl_3$) δ 7.24 – 7.12 (m, 1H), 6.73 (tt, *J* = 7.4, 1.1 Hz, 1H), 6.65 (d, *J* = 7.4 Hz, 2H), 5.97 (ddt, *J* = 17.1, 10.5, 5.4 Hz, 1H), 5.39 – 5.23 (m, 1H), 5.22 – 5.10 (m, 1H), 3.96 (br s, 1H), 3.79 (dt, *J* = 5.4, 1.6 Hz, 2H). ^{13}C NMR (101 MHz, $CDCl_3$) δ 147.8, 135.3, 129.2, 117.7, 116.4, 113.2, 46.7.

Data match literature values.[6]

2-Allylaniline

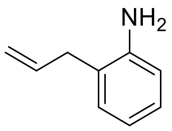

Boron trifluoride diethyl etherate (1.48 mL, 12.0 mmol, 1.2 equiv) was added dropwise to a stirred solution of *N*-allylaniline (1.33 g, 10.0 mmol, 1 equiv) in *p*-xylene (20 mL). The solution was then stirred at 180°C for 14 h. The reaction was then allowed to cool to room temperature and aqueous NaOH (2.5 M, 20 mL) was added. The aqueous layer was extracted with ethyl acetate (3 x 25 mL) and the combined organics were then dried ($MgSO_4$), filtered and concentrated in *vacuo*. The crude residue was purified by silica gel chromatography (eluent: Pet. Ether:EtOAc, 95:5 – 90:10) to give the title compound (604 mg, 4.53 mmol, 45%) as a pale yellow liquid.

^{1}H NMR (400 MHz, $CDCl_3$) δ 7.13 – 7.01 (m, 2H), 6.77 (td, *J* = 7.5, 1.1 Hz, 1H), 6.71 (dd, *J* = 7.8, 1.1 Hz, 1H), 5.97 (ddt, *J* = 16.5, 10.2, 6.2 Hz, 1H), 5.19 – 5.06 (m, 2H), 3.70 (br s, 2H), 3.33 (dt, *J* = 6.2, 1.7 Hz, 2H). ^{13}C NMR (101 MHz, $CDCl_3$) δ 144.6, 135.9, 130.2, 127.5, 124.1, 119.0, 116.1, 115.9, 36.5.

Data match literature values.[6]

Tetrabutylammonium (2-allylphenyl)sulfamate (1t)

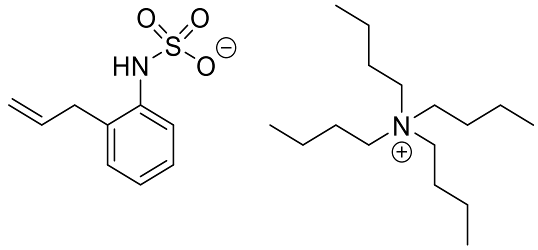

2-Allylaniline (532 mg, 3.99 mmol, 1 equiv), chlorosulfonic acid (500 mg, 0.28 mL, 4.29 mmol, 1.1 equiv) and tetrabutylammonium hydrogen sulfate (1.22 g, 3.59 mmol, 0.9 equiv) were subject to general **GP-1** to give the title compound (1.53 g, 3.36 mmol, 93%) as an off-white solid.

^{1}H NMR (400 MHz, DMSO-d_6) δ 7.40 (dd, *J* = 8.2, 1.2 Hz, 1H), 7.03 (td, *J* = 7.7, 1.6 Hz, 1H), 6.98 (dd, *J* = 7.6, 1.5 Hz, 1H), 6.76 (td, *J* = 7.4, 1.2 Hz, 1H), 6.35 (br s, 1H), 5.87 (ddt, *J* = 16.7, 10.1, 6.4 Hz, 1H), 5.09 – 4.91 (m, 2H), 3.24 (d, *J* = 6.4 Hz, 2H), 3.15 – 2.99 (m, 8H), 1.59 – 1.39 (m, 8H), 1.26 (h, *J* = 7.4 Hz, 8H), 0.87 (t, *J* = 7.3 Hz, 12H). HRMS (ESI) calcd for $[C_9H_{10}NO_3S]^-$: 212.0387. Found: 212.0386.

Tetrabutylammonium (2-biphenyl)sulfamate (1u)

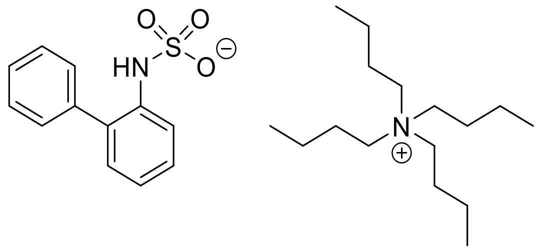

2-Aminobiphenyl (2.54 g, 15.0 mmol, 1 equiv), chlorosulfonic acid (1.75 g, 0.98 mL, 15.0 mmol, 1 equiv) and tetrabutylammonium hydrogen sulfate (4.08 g, 12.0 mmol, 0.8 equiv)

were subject to **GP-1** to give the title compound (4.37 g, 8.91 mmol, 74%) as an off-white solid.

^{1}H NMR (400 MHz, DMSO-d_6) δ 7.65 (d, *J* = 8.5 Hz, 1H), 7.49 (t, *J* = 7.5 Hz, 2H), 7.44 – 7.32 (m, 3H), 7.19 (td, *J* = 7.8, 1.6 Hz, 1H), 7.05 (dd, *J* = 7.5, 1.6 Hz, 1H), 6.86 (td, *J* = 7.4, 1.1 Hz, 1H), 5.94 (br s, 1H), 3.21 – 3.04 (m, 8H), 1.70 – 1.44 (m, 8H), 1.27 (h, *J* = 7.4 Hz), 0.89 (t, *J* = 7.3 Hz). ^{13}C NMR (101 MHz, DMSO-d_6) δ 140.3, 139.1, 129.8, 129.5, 129.4, 129.0, 128.2, 127.8, 120.0, 117.8, 58.0, 23.5, 19.6, 13.9. HRMS (ESI) calcd for $[C_{12}H_{10}NO_3S]^-$:248.0387. Found: 248.0383.

2-(4-Chlorophenyl)aniline

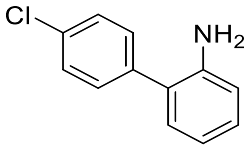

A solution of 2-iodoaniline (2.19 g, 10.0 mmol, 1 equiv), 4-chlorophenylboronic acid (3.12 g, 20.0 mmol, 2 equiv) and potassium carbonate (3.45 g, 25.0 mmol, 2.5 equiv) in acetone (20 mL) and water (24 mL) was heated to 65℃. A solution of $Pd(OAc)_2$ (22.5 mg, 100 µmol, 0.01 equiv) in acetone (4 mL) was then added and the resulting yellow solution was stirred at 65℃ for 20 h. The resulting black solution was allowed to cool to room temperature and the aqueous layer was extracted with ethyl acetate (3 x 40 mL). The combined organics were then washed with water (3x 30 mL), dried ($MgSO_4$), filtered and concentrated *in vacuo*. The crude residue was purified by silica gel chromatography (eluent: Pet. Ether:EtOAc, 98:2) to give the title compound (1.47 g, 7.22 mmol, 72%) as a yellow liquid.

^{1}H NMR (400 MHz, $CDCl_3$) δ 7.53 – 7.39 (m, 4H), 7.22 (t, *J* = 7.8, 1H), 7.14 (d, *J* = 7.6, 1H), 6.88 (t, *J* = 7.4, 1H), 6.80 (d, *J* = 8.0, 1H), 3.75 (br s, 2H). ^{13}C NMR (100 MHz, $CDCl_3$) δ 143.5, 138.0, 133.1, 130.5, 130.4, 129.0, 128.9, 126.3, 118.8, 115.8.

Data match literature values.[7]

Tetrabutylammonium (2-(4-chlorophenyl)phenyl)sulfamate (1v)

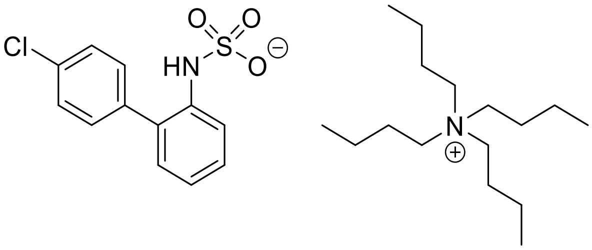

2-(4-Chlorophenyl)aniline (1.27 g, 6.23 mmol, 1 equiv), chlorosulfonic acid (728 mg, 0.42 mL, 6.25 mmol, 1 equiv) and tetrabutylammonium hydrogen sulfate (1.96 g, 5.77 mmol, 0.9 equiv) were subject to **GP-1** to give the title compound (2.76 g, 5.25 mmol, 91%) as a pink oil.

^{1}H NMR (400 MHz, DMSO-d_6) δ 7.60 (dd, *J* = 8.2, 1.1 Hz, 1H), 7.54 – 7.43 (m, 2H), 7.41 – 7.35 (m, 2H), 7.20 (td, *J* = 8.2, 1.6 Hz, 1H), 7.03 (dd, *J* = 7.6, 1.6 Hz, 1H), 6.88 (td, *J* = 7.4, 1.1 Hz, 1H), 5.93 (br s, 1H), 3.28 – 2.82 (m, 8H), 1.72 – 1.39 (m, 8H), 1.24 (h, *J* = 7.4 Hz, 8H), 0.86 (t, *J* = 7.3 Hz, 12H). ^{13}C NMR (101 MHz, DMSO-d_6) δ 139.8, 137.8, 132.7, 131.2, 129.9, 129.4, 128.6, 128.2, 120.7, 118.4, 58.0, 23.5, 19.5, 13.8. HRMS (ESI) calcd for $[C_{12}H_9ClNO_3S]^-$:281.9997. Found: 281.9997.

2-(4-Methylphenyl)aniline

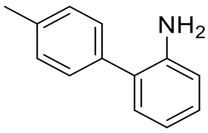

A suspension of 2-iodoaniline (2.19 g, 10.0 mmol, 1 equiv), 4-methylphenylboronic acid (2.04 g, 15.0 mmol, 1.5 equiv) and potassium carbonate (5.52 g, 40.0 mmol, 4 equiv) in DME (10 mL) and water (20 mL) was stirred at room temperature for 30 min. Bis(triphenylphosphine)palladium(II) dichloride (140 mg, 199 µmol, 0.02 equiv) and DME (10 mL) were then added. The resulting yellow suspension was stirred at 80 °C for 20 h. The resulting dark red suspension was allowed to cool to room temperature and the aqueous layer was extracted with ethyl acetate (3 x 40 mL). The combined organics were then dried ($MgSO_4$), filtered and concentrated *in vacuo*. The crude residue was then purified by silica gel chromatography (eluent: Pet. Ether:EtOAc, 99:1-96:4) to give the title compound (1.62 g, 8.84 mmol, 88%) as a yellow liquid.

^{1}H NMR (400 MHz, $CDCl_3$) δ 7.37 (d, *J* = 7.8 Hz, 2H), 7.27 (d, *J* = 7.7 Hz, 2H), 7.20 – 7.11 (m, 2H), 6.83 (t, *J* = 7.5 Hz, 1H), 6.77 (d, *J* = 7.9 Hz, 1H), 3.76 (br s, 2H), 2.42 (s, 3H). ^{13}C NMR (101 MHz, $CDCl_3$) δ 143.4, 136.8, 136.5, 130.4, 129.5, 128.9, 128.3, 127.6, 118.6, 115.5, 21.2.

Data match literature values.[7]

Tetrabutylammonium (2-(*p*-tolyl)phenyl)sulfamate (1w)

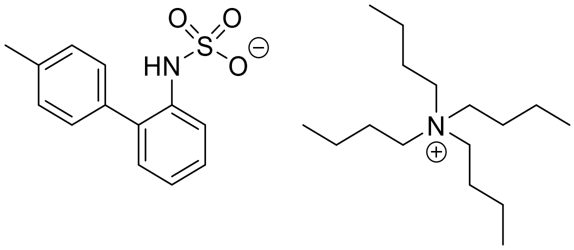

2-(4-Methylphenyl)aniline (1.27 g, 6.93 mmol, 1 equiv), chlorosulfonic acid (804 mg, 0.46 mL, 6.90 mmol, 1 equiv) and tetrabutylammonium hydrogen sulfate (1.88 g, 5.54 mmol, 0.8 equiv) were subject to **GP-1** to give the title compound (641 mg, 1.27 mmol, 23%) as a colourless oil.

^{1}H NMR (400 MHz, DMSO-*d*$_6$) δ 7.59 (d, *J* = 8.5 Hz, 1H), 7.28 (d, *J* = 7.9 Hz, 2H), 7.24 (d, *J* = 8.1 Hz, 2H), 7.16 (td, *J* = 7.7, 1.6 Hz, 1H), 7.02 (dd, *J* = 7.6, 1.6 Hz, 1H), 6.85 (td, *J* = 7.4, 1.1 Hz, 1H), 3.21 – 2.92 (m, 8H), 2.34 (s, 3H), 1.60 – 1.43 (m, 8H), 1.26 (h, *J* = 7.3 Hz, 8H), 0.88 (t, *J* = 7.3 Hz, 12H). ^{13}C NMR (101 MHz, DMSO-*d*$_6$) δ 140.1, 137.2, 136.0, 130.1, 129.8, 129.2, 129.1, 128.0, 120.2, 117.7, 58.0, 23.5, 21.2, 19.6, 13.9. HRMS (ESI) calcd for $[C_{13}H_{12}NO_3S]^-$:262.0543. Found: 262.0541.

Tetrabutylammonium (3,5-dimethoxyphenyl)sulfamate (1x)

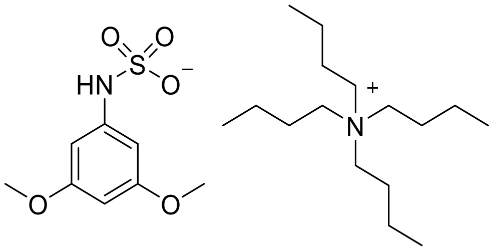

3,5-Dimethoxyaniline (2.30 g, 15.0 mmol, 1 equiv), chlorosulfonic acid (1.75 g, 0.98 mL, 15.0 mmol, 1 equiv) and tetrabutylammonium hydrogen sulfate (4.08 g, 12.0 mmol, 0.8 equiv)

were subject to general procedure 1 to give the title compound (4.32 g, 9.1 mmol, 76%) as a white solid.

^{1}H NMR (400 MHz, $CDCl_3$) δ 6.32 (t, *J* = 2.5 Hz, 2H), 5.91 (d, *J* = 2.3 Hz, 1H), 3.68 (s, 6H), 3.13 (m, 8H), 1.49 (m, 8H), 1.31 (m, 8H), 0.90 (m, 12H). ^{13}C NMR (101 MHz, $CDCl_3$) δ 161.0, 144.6, 95.8, 92.4, 58.3, 55.2, 23.80, 19.6, 13.6. HRMS (ESI) calcd for $[C_8H_{10}NO_5S]^-$: 232.0285. Found: 232.0283.

Tetrabutylammonium (3-chlorophenyl)sulfamate (1y)

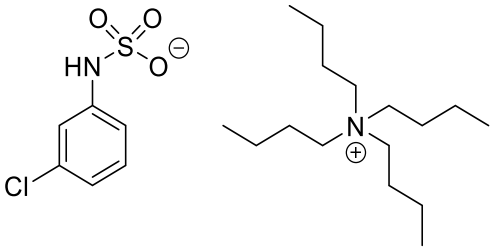

3-Chloroaniline (1.28 g, 1.07 mL 10.0 mmol, 1 equiv), chlorosulfonic acid (1.32 g, 0.67 mL, 10.0 mmol, 1 equiv) and tetrabutylammonium hydrogen sulfate (2.38 g, 7.01 mmol, 0.7 equiv) were subject to **GP-1** to give the title compound (1.73 g, 3.85 mmol, 55%) as a white solid.

^{1}H NMR (400 MHz, $CDCl_3$) δ 7.18 (t, *J* = 2.0 Hz, 1H), 7.14 (br s, 1H), 6.96 (t, *J* = 7.9 Hz, 1H), 6.89 (dd, *J* = 8.2, 2.2 Hz, 1H), 6.64 (dd, *J* = 7.8, 2.1 Hz, 1H), 3.15 – 2.70 (m, 8H), 1.57 – 1.32 (m, 8H), 1.25 (h, *J* = 7.4 Hz, 8H), 0.83 (t, *J* = 7.3 Hz, 12H). ^{13}C NMR (101 MHz, $CDCl_3$) δ 144.2, 134.0, 129.5, 119.2, 116.3, 114.89, 58.2, 23.7, 19.5, 13.6. HRMS (ESI) calcd for $[C_6H_5ClNO_3S]^-$: 205.9684. Found: 205.9686.

Tetrabutylammonium (3-bromophenyl)sulfamate (1z)

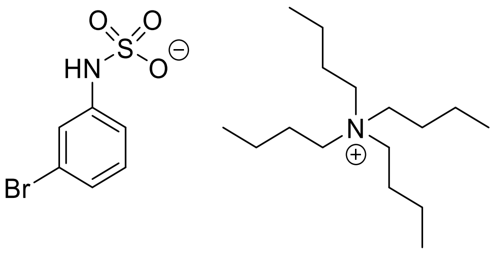

3-Bromoaniline (1.72 g, 1.01 mL 10.0 mmol, 1 equiv), chlorosulfonic acid (1.32 g, 0.67 mL, 10.0 mmol, 1 equiv) and tetrabutylammonium hydrogen sulfate (2.72 g, 8.01 mmol, 0.8 equiv) were subject to **GP-1** to give the title compound (2.77 g, 5.61 mmol, 56%) as a white solid.

^{1}H NMR (400 MHz, CDCl$_3$) δ 7.35 (s, 1H), 7.00 (d, *J* = 8.2 Hz, 1H), 6.94 (t, *J* = 7.9 Hz, 1H), 6.84 (d, *J* = 7.8 Hz, 1H), 3.24 – 2.98 (m, 8H), 1.56 – 1.38 (m, 8H), 1.29 (h, *J* = 7.3 Hz, 8H), 0.88 (t, *J* = 7.3 Hz, 12H). ^{13}C NMR (101 MHz, CDCl$_3$) δ 144.5, 129.9, 122.4, 122.2, 119.2, 115.4, 58.2, 23.76, 19.6, 13.6. HRMS (ESI) calcd for $[C_6H_5BrNO_3S]^-$: 249.9179. Found: 249.9177.

Tetrabutylammonium (3,5-difluorophenyl)sulfamate (1aa)

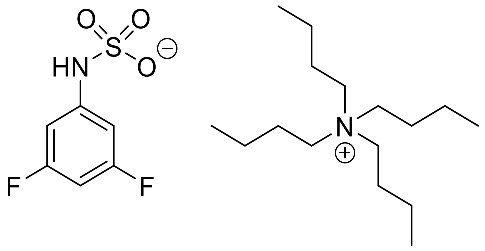

3,5-Difluoroaniline (1.94 g, 15.0 mmol, 1 equiv), chlorosulfonic acid (1.75 g, 0.98 mL, 15.0 mmol, 1 equiv) and tetrabutylammonium hydrogen sulfate (4.08 g, 12.0 mmol, 0.8 equiv) were subject to **GP-1** to give the title compound (1.40 g, 3.11 mmol, 26%) as a white solid.

^{1}H NMR (400 MHz, CDCl$_3$) δ 7.64 (br s, 1H), 6.87 – 6.47 (m, 2H), 6.15 (t, *J* = 9.2, 1H), 3.15 (m, 8H), 1.68 – 1.38 (m, 8H), 1.32 (h, *J* = 7.3 Hz, 8H), 0.90 (t, *J* = 7.3 Hz, 12H). ^{13}C NMR (101 MHz, CDCl$_3$) δ 163.4 (dd, *J* = 243.1, 15.3 Hz), 145.5 (t, *J* = 13.6 Hz), 99.9 – 98.6 (m), 94.0 (t, *J* = 26.0 Hz), 58.3, 23.8, 19.5, 13.5. ^{19}F NMR (376 MHz, CDCl$_3$) δ -112.17. HRMS (ESI) calcd for $[C_6H_4F_2NO_3S]^-$: 207.9885. Found: 207.9883.

Tetrabutylammonium *N*-benzyl(phenyl)sulfamate (1ab)

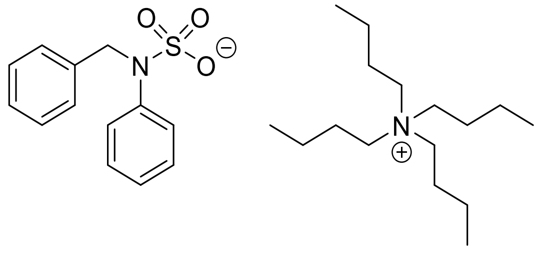

N-Benzylaniline (1.61 g, 2.59 mL, 15.0 mmol, 1 equiv), chlorosulfonic acid (1.75 g, 0.98 mL, 15.0 mmol, 1 equiv) and tetrabutylammonium hydrogen sulfate (4.08 g, 12.0 mmol, 0.8 equiv) were subject to **GP-1** to give the title compound (4.51 g, 8.93 mmol, 75%) as a white solid.

^{1}H NMR (400 MHz, $CDCl_3$) δ 7.43 – 7.30 (m, 4H), 7.17 (t, *J* = 7.4 Hz, 2H), 7.12 – 7.03 (m, 3H), 6.81 (t, *J* = 7.3 Hz, 1H), 4.95 (s, 2H), 3.58 – 2.58 (m, 8H), 1.47 (tt, *J* = 8.0, 5.9 Hz, 8H), 1.31 (h, *J* = 7.3 Hz, 8H), 0.92 (t, *J* = 7.3 Hz, 12H). ^{13}C NMR (100 MHz, $CDCl_3$) δ 145.3, 140.5, 127.9, 127.8, 127.6, 126.0, 122.5, 121.4, 58.3, 53.9, 23.8, 19.6, 13.7. HRMS (ESI) calcd for $[C_{13}H_{12}NO_3S]^-$: 262.0543. Found: 262.0546.

Tetrabutylammonium *N*-isopropyl(phenyl)sulfamate (1ac)

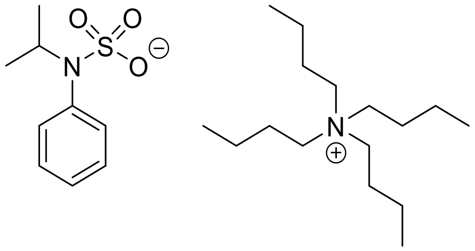

N-Isopropylaniline (2.03 g, 2.17 mL, 15.0 mmol, 1 equiv), chlorosulfonic acid (1.75 g, 0.98 mL, 15.0 mmol, 1 equiv) and tetrabutylammonium hydrogen sulfate (4.08 g, 12.0 mmol, 0.8 equiv) were subject to **GP-1** to give the title compound (2.08 g, 4.56 mmol, 38%) as a white solid.

^{1}H NMR (400 MHz, $CDCl_3$) δ 7.33 (dd, *J* = 8.4, 1.3 Hz, 2H), 7.09 (t, *J* = 8.4, 2H), 7.05 – 6.93 (m, 1H), 4.27 (hept, *J* = 6.7 Hz, 1H), 3.19 – 2.84 (m, 8H), 1.49 – 1.31 (m, 8H), 1.24 (h, *J* = 7.3 Hz, 8H), 0.97 (d, *J* = 6.8 Hz, 6H), 0.83 (t, *J* = 7.3 Hz, 12H). ^{13}C NMR (101 MHz, $CDCl_3$) δ 142.5, 130.7, 127.4, 125.0, 58.3, 50.1, 23.8, 22.0, 19.5, 13.6. HRMS (ESI) calcd for $[C_9H_{12}NO_3S]^-$: 214.0541. Found: 214.0543.

Tetrabutylammonium *N*-methyl(phenyl)sulfamate (1ad)

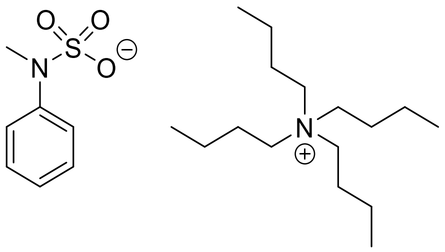

N-Methylaniline (1.61 g, 1.62 mL, 15.0 mmol, 1 equiv), chlorosulfonic acid (1.75 g, 0.98 mL, 15.0 mmol, 1 equiv) and tetrabutylammonium hydrogen sulfate (4.08 g, 12.0 mmol, 0.8 equiv) were subject to **GP-1** to give the title compound (3.04 g, 7.09 mmol, 69%) as a white solid.

^{1}H NMR (600 MHz, $CDCl_3$) δ 7.42 (dd, *J* = 8.7, 1.2 Hz, 2H), 7.23 – 7.07 (m, 2H), 6.89 (tt, *J* = 7.3, 1.2 Hz, 1H), 3.17 (s, 3H), 3.09 – 2.96 (m, 8H), 1.46 (m, 8H), 1.30 (h, *J* = 7.4 Hz, 8H), 0.90 (t, *J* = 7.4 Hz, 12H). ^{13}C NMR (151 MHz, $CDCl_3$) δ 147.1, 127.8, 122.8, 121.9, 58.2, 37.9, 23.8, 19.6, 13.7. HRMS (ESI) calcd for $[C_7H_8NO_3S]^-$: 186.0230. Found: 186.0224.

***tert*-Butyl (2,3-dimethylphenyl)carbamate**

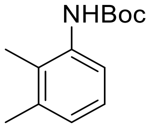

Di-*tert*-butyl dicarbonate (5.45 g, 25.0 mmol, 1 equiv) was added to a solution of 2,3-Dimethylaniline (3.07 g, 3.1 mL, 25.4 mmol, 1 equiv) and potassium carbonate (6.90 g. 50.0 mmol, 2 equiv) in THF:H_2O (1:1, 50 mL). The reaction was stirred at room temperature for 6 h. Water (20 mL) was then added and the aqueous layer was extracted with CH_2Cl_2 (3 x 50 mL). The combined organics were then dried ($MgSO_4$), filtered and concentrated *in vacuo*. The crude residue was then purified by silica gel chromatography (eluent: Pet. Ether:EtOAc, 100:0 – 95:5) to give the title compound (3.89 g, 17.6 mmol, 70%) as a white solid.

^{1}H NMR (400 MHz, $CDCl_3$) δ 7.53 (d, *J* = 7.6 Hz, 1H), 7.09 (t, *J* = 7.8 Hz, 1H), 6.95 (d, *J* = 7.5 Hz, 1H), 6.31 (br s, 1H), 2.30 (s, 3H), 2.16 (s, 3H), 1.54 (s, 9H). ^{13}C NMR (101 MHz, $CDCl_3$) δ 153.5, 137.1, 135.9, 127.6, 126.1, 125.8, 120.4, 80.2, 28.4, 20.7, 13.5.

Data match literature values.[8]

***N*,2,3-Trimethylaniline**

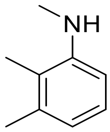

Sodium hydride (60% in mineral oil, 576 mg, 14.4 mmol, 1.2 equiv) was added to a stirred solution of *tert*-butyl (2,3-dimethylphenyl)carbamate (2.66 g, 12.0 mmol, 1 equiv) in dry DMF

(24 mL). The reaction was stirred at 0℃ for 30 min. Methyl iodide (0.89 mL, 2.03 g, 14.3 mmol, 2 equiv) was then added dropwise at 0℃. The reaction was allowed to warm to room temperature and was stirred for a further 16 h. The reaction was then quenched with water (20 mL) and the aqueous layer was extracted with CH_2Cl_2 (3 x 50 mL). The combined organics were then washed with water (5 x 50 mL), dried ($MgSO_4$), filtered and concentrated *in vacuo.* The resulting residue was then dissolved in CH_2Cl_2 (60 mL). Trifluoroacetic acid (21 mL) was then added and the reaction was stirred at room temperature for 1 h. The reaction was quenched with aqueous sodium carbonate (100 mL) and the aqueous layer was extracted with CH_2Cl_2 (3 x 50 mL). The combined organics were then dried ($MgSO_4$), filtered and concentrated *in vacuo*. The crude reasidue was then purified by silica gel chromatography (eluent: Pet. Ether:EtOAc, 98:2 – 93:7) to give the title compound (1.21 g, 8.94 mmol, 74%) as a colourless liquid.

^{1}H NMR (400 MHz, $CDCl_3$) δ 7.09 (t, *J* = 7.8 Hz, 1H), 6.64 (d, *J* = 7.6 Hz, 1H), 6.56 (d, *J* = 8.1 Hz, 1H), 3.65 (br s, 1H), 2.92 (s, 3H), 2.32 (s, 3H), 2.08 (s, 3H). ^{13}C NMR (101 MHz, $CDCl_3$) δ 147.2, 136.4, 126.3, 120.3, 119.3, 107.4, 31.2, 20.7, 12.4. HRMS (ESI) calcd for $[C_9H_{13}N + H]^+$: 136.1121. Found: 136.1117.

Tetrabutylammonium *N*-methyl-(2,3-dimethylphenyl)sulfamate (1ae)

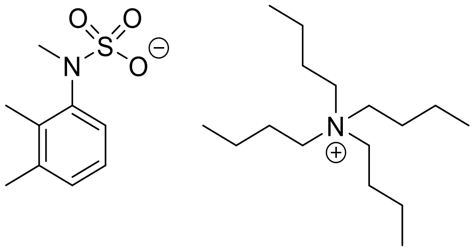

N,2,3-Trimethylaniline (1.08 g, 7.99 mmol, 1 equiv), chlorosulfonic acid (932 mg, 0.53 mL, 8.00 mmol, 1 equiv) and tetrabutylammonium hydrogen sulfate (2.44 g, 7.19 mmol, 0.9 equiv) were subject to **GP-1** to give the title compound (2.03 g, 4.44 mmol, 61%) as a white solid.

^{1}H NMR (400 MHz, $CDCl_3$) δ 7.50 (dd, *J* = 7.8, 1.4 Hz, 1H), 6.94 (t, *J* = 7.6 Hz, 1H), 6.88 (dd, *J* = 7.6, 1.4 Hz, 1H), 3.14 – 3.04 (m, 8H), 3.01 (s, 3H), 2.29 (s, 3H), 2.19 (s, 3H), 1.56 – 1.41 (m, 8H), 1.32 (h, *J* = 7.4 Hz, 8H), 0.92 (t, *J* = 7.3 Hz, 12H). ^{13}C NMR (101 MHz, $CDCl_3$) δ 146.7, 136.8,

136.5, 126.8, 124.8, 123.9, 58.3, 39.8, 23.9, 20.6, 19.6, 14.4, 13.7. HRMS (ESI) calcd for $[C_9H_{12}NO_3S]^-$:214.0543. Found: 214.0544.

1-Nitro-2-vinylbenzene

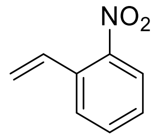

Potassium *tert*-butoxide (3.82 g, 34.0 mmol, 1.7 equiv) was added portion-wise to a stirred suspension of methyltriphenylphosphonium bromide (12.1 g, 33.8 mmol, 1.7 equiv) in THF (40 mL) at 0°C. The resulting yellow suspension was stirred at room temperature for 30 min. The reaction was then cooled to 0°C and 2-nitrobenzaldehyde (3.02 g, 20.0 mmol, 1 equiv) was added. The reaction was then stirred at room temperature for 5 h. The reaction was then quenched with water (40 mL) and the aqueous layer was extracted with ethyl acetate (3 x 50 mL). The combined organics were then dried ($MgSO_4$), filtered and concentrated *in vacuo*. The crude residue was then purified by silica gel chromatography (eluent: Pet. Ether:EtOAc, 98:2) to give the title compound (2.21 g, 14.8 mmol, 74%) as a colourless oil.

^{1}H NMR (500 MHz, $CDCl_3$) δ 7.95 (dd, *J* = 8.2, 1.3 Hz, 1H), 7.65 (dd, *J* = 7.9, 1.6 Hz, 1H), 7.61 (td, *J* = 7.6, 0.8 Hz, 1H), 7.47 – 7.40 (m, 1H), 7.20 (dd, *J* = 17.3, 11.0 Hz, 1H), 5.77 (dd, *J* = 17.3, 0.9 Hz, 1H), 5.51 (dd, *J* = 11.0, 0.9 Hz, 1H). ^{13}C NMR (126 MHz, $CDCl_3$) δ 147.9, 133.4, 133.1, 132.5, 128.5, 128.4, 124.4, 119.0.

Data match literature values.[9]

2-Vinylaniline

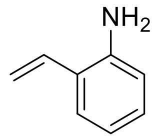

Powdered zinc (3.14 g, 48.0 mmol, 6 equiv) as added to a solution of 1-nitro-2-vinylbenzene (1.19 g, 7.98 mmol, 1 equiv) in acetic acid (16 mL). The resulting suspension was stirred at room temperature for 3 h. The reaction was then quenched with saturated aqueous $NaHCO_3$ and the aqueous layer was extracted with ether (3 x 30 mL). The combined organics were

then washed with brine, dried ($MgSO_4$), filtered and concentrated *in vacuo*. The crude residue was then purified by silica gel chromatography (eluent: Pet. Ether:EtOAc, 97:3 – 93:7) to give the title compound (407 mg, 3.42 mmol, 43%) as a red liquid.

^{1}H NMR (400 MHz, $CDCl_3$) δ 7.32 (dd, *J* = 7.7, 1.5 Hz, 1H), 7.12 (td, *J* = 7.7, 1.6 Hz, 1H), 6.86 – 6.76 (m, 2H), 6.73 (dd, *J* = 7.9, 1.1 Hz, 1H), 5.66 (dd, *J* = 17.4, 1.4 Hz, 1H), 5.35 (dd, *J* = 11.0, 1.4 Hz, 1H), 3.99 (s, 2H). ^{13}C NMR (101 MHz, $CDCl_3$) δ 143.3, 132.7, 128.7, 127.3, 124.3, 119.1, 116.2, 115.8.

Data match literature values.[10]

Tetrabutylammonium (2-vinylphenyl)sulfamate

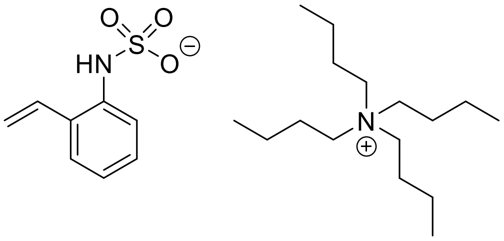

2-Vinylaniline (356 mg, 2.99 mmol, 1 equiv), chlorosulfonic acid (199 µL, 348 mg, 2.99 mmol, 1 equiv) and tetrabutylammonium hydrogen sulfate (968 mg, 2.85mmol, 0.95 equiv) were subject to **GP-1** to give the title compound (1.12 g, 2.54 mmol, 89%) as a yellow solid.

^{1}H NMR (400 MHz, MeOD-*d*$_4$) δ 7.63 (dd, *J* = 8.2, 1.1 Hz, 1H), 7.42 (dd, *J* = 7.7, 1.5 Hz, 1H), 7.21 (td, *J* = 7.8, 1.5 Hz, 1H), 7.10 – 6.89 (m, 2H), 5.67 (dd, *J* = 17.4, 1.5 Hz, 1H), 5.34 (dd, *J* = 11.1, 1.5 Hz, 1H), 3.22 – 3.11 (m, 10H), 1.69 – 1.50 (m, 11H), 1.39 (h, *J* = 7.4 Hz, 10H), 1.01 (t, *J* = 7.4 Hz, 14H). ^{13}C NMR (101 MHz, MeOD-*d*$_4$) δ 138.6, 132.8, 127.9, 127.9, 126.1, 121.6, 119.9, 115.1, 58.0, 23.4, 19.3, 12.8. HRMS (ESI) calcd for $[C_8H_8NO_3S]^-$: 198.0230. Found: 198.0224.

***N*-(3-Amino-2-methylphenyl)-4-methylbenzenesulfonamide**

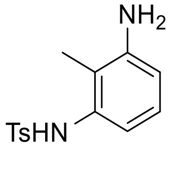

Triethylamine (4.15 mL, 30.0 mmol, 2 equiv) was added to a stirred solution of 2,6-diaminotoluene (5.49 g, 45.0 mmol, 3 equiv) and *p*-toluenesulfonyl chloride (2.87 g, 15.0

mmol, 1 equiv) at 0°C. The reaction was then stirred at room temperature for 16 h. The reaction was then quenched with aqueous $NaHCO_3$ and the aqueous layer was extracted with CH_2Cl_2 (3 x 50 mL). The combined organics were then dried ($MgSO_4$), filtered and concentrated *in vacuo*. The crude residue was then purified by silica gel chromatography (eluent: Pet. Ether:EtOAc, 80:20 – 50:50) to give the title compound (2.43 g, 8.80 mmol, 59%) as a white solid.

^{1}H NMR (700 MHz, $CDCl_3$) δ 7.63 (d, *J* = 8.3 Hz, 2H), 7.24 (d, *J* = 8.0 Hz, 2H), 6.91 (t, *J* = 7.9 Hz, 1H), 6.61 – 6.52 (m, 2H), 6.33 (s, 1H), 2.42 (s, 3H), 1.93 (s, 3H). ^{13}C NMR (176 MHz, $CDCl_3$) δ 145.5, 143.6, 136.7, 134.7, 129.5, 127.3, 126.6, 118.1, 116.4, 113.8, 21.5, 11.5. HRMS (ESI) calcd for $[C_{14}H_{16}N_2O_2S + H]^+$: 277.1005. Found: 277.1012.

_N_-(3-Amino-2-methylphenyl)-_N_,4-dimethylbenzenesulfonamide

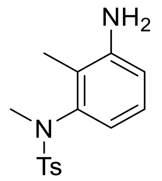

Under an atmosphere of nitrogen, sodium hydride (60% in mineral oil, 144 mg, 3.60 mmol, 1.2 equiv) was added to a solution of *N*-(3-Amino-2-methylphenyl)-4-methylbenzenesulfonamide (828 mg, 3.00 mmol, 1 equiv) in anhydrous DMF (6 mL) at 0°C. The reaction was stirred at 0°C for 30 min. Methyl iodide (205 µL, 467 mg, 3.29 mmol, 1.1 equiv) was then added and the reaction was stirred at room temperature for 15 h. The reaction was then quenched with water. The aqueous layer was then extracted with ethyl acetate (3 x 10 mL). The combined organics were then washed with water (5 x 50 mL) and brine (2 x 50 mL), dried ($MgSO_4$), filtered and concentrated *in vacuo*. The crude residue was then purified by silica gel chromatography (eluent: Pet. Ether:EtOAc, 95:5 – 80:20) to give the title compound (638 mg, 2.20 mmol, 73%) as a white solid.

^{1}H NMR (500 MHz, $CDCl_3$) δ 7.62 (d, *J* = 8.3 Hz, 1H), 7.31 (d, *J* = 8.7 Hz, 2H), 6.89 (t, *J* = 7.9 Hz, 1H), 6.70 (dd, *J* = 7.9, 1.2 Hz, 1H), 6.08 (dd, *J* = 7.9, 1.2 Hz, 1H), 4.22 (s, 2H), 3.13 (d, *J* = 0.8 Hz, 3H), 2.47 (s, 3H), 2.26 (s, 3H). ^{13}C NMR (126 MHz, $CDCl_3$) δ 145.3, 143.3, 141.1, 135.0, 129.3, 128.1, 126.3, 123.9, 117.3, 115.1, 39.1, 21.6, 12.6. HRMS (ESI) calcd for $[C_{15}H_{18}N_2O_2S + H]^+$: 291.1162. Found: 291.1172.

Tetrabutylammonium (3-((N,4-dimethylphenyl)sulfonamido)-2-methylphenyl)sulfamate

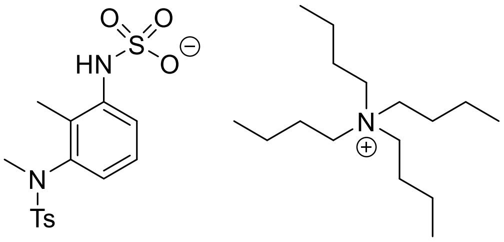

N-(3-Amino-2-methylphenyl)-*N*,4-dimethylbenzenesulfonamide (581 mg, 2.00 mmol, 1 equiv), chlorosulfonic acid (133 µL, 233 mg, 2.00 mmol, 1 equiv) and tetrabutylammonium hydrogen sulfate (645 mg, 1.90 mmol, 0.95 equiv) were subject to **GP-1** to give the title compound (864 mg, 1.41 mmol, 74%) as a white solid.

^{1}H NMR (400 MHz, MeOD-*d*$_4$) δ 7.58 (dd, *J* = 8.3, 2.0 Hz, 3H), 7.41 (d, *J* = 8.1 Hz, 2H), 6.99 (t, *J* = 8.1 Hz, 1H), 6.29 – 6.16 (m, 1H), 3.29 – 3.20 (m, 8H), 3.11 (s, 3H), 2.47 (s, 3H), 2.26 (s, 3H), 1.72 – 1.61 (m, 8H), 1.41 (h, *J* = 7.4 Hz, 8H), 1.02 (t, *J* = 7.3 Hz, 12H). ^{13}C NMR (101 MHz, MeOD-*d*$_4$) δ 143.9, 141.1, 140.5, 134.9, 129.3, 127.8, 127.2, 125.6, 119.7, 118.6, 58.1, 38.3, 23.4, 20.2, 19.3, 12.6, 11.7. HRMS (ESI) calcd for $[C_{15}H_{18}NO_5S_2]^-$: 369.0584. Found: 369.0579.

Tetrabutylammonium diphenylsulfamate

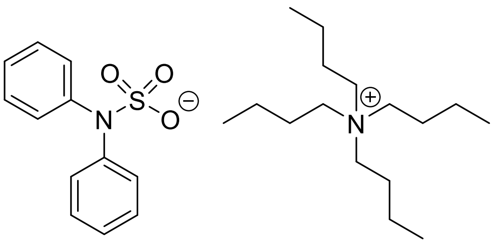

Diphenylamine (2.54 g, 15.0 mmol 1 equiv), chlorosulfonic acid (3.00 mL, 5,25 g, 45.1 mmol, 3 equiv) and tetrabutylammonium hydrogen sulfate (4.58 g, 13.5 mmol, 0.9 equiv) were subject to **GP-1** to give the title compound (743 mg, 1.51 mmol, 11%) as a yellow oil.

^{1}H NMR (400 MHz, MeOD-*d*$_4$) δ 7.22 (t, *J* = 7.7 Hz, 4H), 7.10 (d, *J* = 8.0 Hz, 4H), 6.84 (t, *J* = 7.3 Hz, 2H), 3.25 – 3.17 (m, 8H), 1.70 – 1.61 (m, 8H), 1.41 (h, *J* = 7.4 Hz, 8H), 1.02 (t, *J* = 7.3 Hz, 12H). ^{13}C NMR (101 MHz, MeOD-*d*$_4$) δ 143.8, 128.8, 119.7, 116.9, 58.1, 23.4, 19.3, 12.6. HRMS (ESI) calcd for $[C_{12}H_{10}NO_3S]^-$: 248.0387. Found: 248.0381.

Tetrabutylammonium naphthalen-1-ylsulfamate

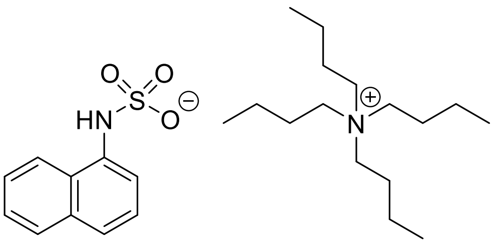

1-Naphthylamine (1.43 g, 10.0 mmol, 1 equiv), chlorosulfonic acid (1.32 g, 0.67 mL, 10.0 mmol, 1 equiv) and tetrabutylammonium hydrogen sulfate (2.72 g, 8.01 mmol, 0.8 equiv) were subject to general procedure 1 to give the title compound (2.37 g, 5.10 mmol, 64%) as an off-white solid.

^{1}H NMR (400 MHz, $CDCl_3$) δ 7.82 – 7.63 (m, 3H), 7.40 – 7.21 (m, 4H), 6.64 (s, 1H), 3.67 – 2.13 (m, 8H), 1.30 (p, *J* = 8.0, 7.5 Hz, 8H), 1.16 (h, *J* = 7.3 Hz, 8H), 0.80 (t, *J* = 7.3 Hz, 12H).^{13}C NMR (101 MHz, $CDCl_3$) δ 137.7, 134.0, 128.4, 126.4, 125.3, 124.9, 124.4, 120.4, 120.1, 112.9, 58.0, 23.6, 19.5, 13.6. HRMS (ESI) calcd for $[C_{10}H_8NO_3S]^-$: 222.0230. Found: 222.0226.

Tetrabutylammonium (2-(trifluoromethyl)phenyl)sulfamate

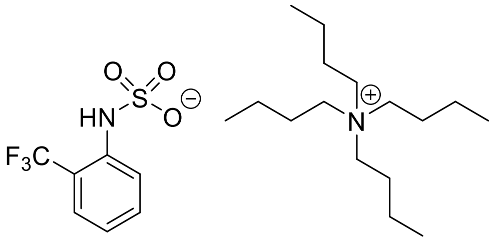

2-(Trifluoromethyl)aniline (1.89 mL, 2.42 g, 15.0 mmol, 1 equiv), chlorosulfonic acid (1.75 g, 999 µL, 15.0 mmol, 1 equiv) and tetrabutylammonium hydrogen sulfate (4.08 g, 12.0 mmol, 0.8 equiv) were subject to general procedure 1 to give the title compound (1.46 g, 3.02 mmol, 25%) as an off-white solid.

^{1}H NMR (700 MHz, $CDCl_3$) δ 7.89 (d, *J* = 8.4 Hz, 1H), 7.36 (d, *J* = 7.9 Hz, 1H), 7.32 (t, *J* = 8.0 Hz, 1H), 6.79 (t, *J* = 7.6 Hz, 1H), 6.58 (s, 1H), 3.18 – 3.06 (m, 8H), 1.51 (p, *J* = 7.9 Hz, 8H), 1.29 (h, *J* = 7.4 Hz, 8H), 0.88 (t, *J* = 7.3 Hz, 12H). ^{13}C NMR (176 MHz, $CDCl_3$) δ 140.7, 132.6, 125.8 – 125.1

(m), 124.8 (q, *J* = 260.5 Hz), 118.5, 118.1, 114.3 (q, *J* = 29.0 Hz), 58.5, 23.8, 19.5, 13.4.^{19}F NMR (376 MHz, $CDCl_3$) δ -62.16.

Data match literature values.[4]

Tetrabutylammonium (2-cyanophenyl)sulfamate

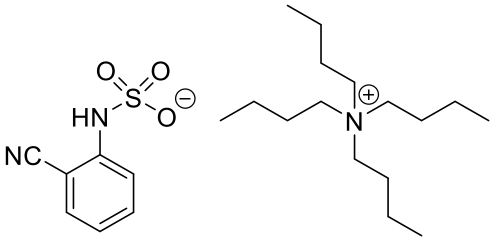

2-Aminobenzonitrile (1.18 g, 10.0 mmol, 1 equiv), chlorosulfonic acid (1.32 g, 0.67 mL, 10.0 mmol, 1 equiv) and tetrabutylammonium hydrogen sulfate (2.72 g, 8.0 mmol, 0.8 equiv) were subject to general procedure 1 to give the title compound (0.72 g, 1.6 mmol, 20%) as an off-white solid.

^{1}H NMR (400 MHz, $CDCl_3$) δ 7.82 (d, *J* = 8.6, 1H), 7.42 – 7.35 (m, 1H), 7.34 (dd, *J* = 7.8, 1.5 Hz, 1H), 6.83 – 6.74 (m, 2H), 3.44 – 2.88 (m, 8H), 1.73 – 1.48 (m, 8H), 1.36 (h, *J* = 7.4 Hz, 8H), 0.94 (t, *J* = 7.3 Hz, 12H). ^{13}C NMR (101 MHz, $CDCl_3$) δ 145.6, 133.9, 131.8, 119.2, 117.5, 116.7, 97.3, 58.6, 23.8, 19.6, 13.6. HRMS (ESI) calcd for $[C_7H_5N_2O_3S]^-$: 197.0026. Found: 197.0024.

Tetrabutylammonium pyridin-2-ylsulfamate

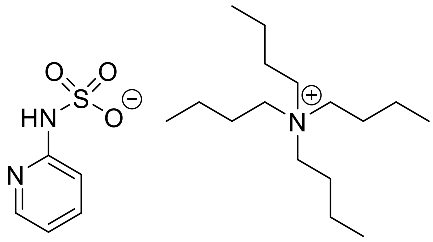

2-Aminopyridine (470 mg, 4.99 mmol, 1 equiv), chlorosulfonic acid (583 mg, 333 µL, 5.00 mmol, 1 equiv) and tetrabutylammonium hydrogen sulfate (1.36 g, 4.01 mmol, 0.8 equiv) were subject to general procedure 1 to give the title compound (349 mg, 0.842 mmol, 21%) as a white solid.

^{1}H NMR (700 MHz, MeOD-d_4) δ 8.18 – 8.07 (m, 1H), 7.69 (t, *J* = 8.1 Hz, 1H), 7.44 (d, *J* = 8.6 Hz, 1H), 6.99 – 6.83 (m, 1H), 3.34 – 3.11 (m, 8H), 1.77 – 1.58 (m, 8H), 1.53 – 1.31 (m, 8H), 1.19 – 0.81 (m, 12H). ^{13}C NMR (101 MHz, $CDCl_3$-*d*) δ 155.2, 147.4, 137.6, 115.2, 110.7, 58.4, 23.8, 19.6, 13.7. HRMS (ESI) calcd for $[C_5H_5N_2O_3S]^-$: 173.0026. Found: 173.0020.

1-Methyl-4-nitro-1H-indole

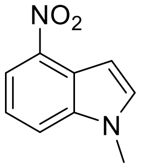

Sodium hydride (60% in mineral oil, 720 mg, 18.0 mmol, 1.2 equiv) was added to a stirred solution of 4-nitroindole (2.43 g, 15.0 mmol, 1 equiv) in anhydrous DMF (30 mL) at 0°C. The reaction was stirred at 0°C for 30 min. Methyl iodide (2.34 g, 1.03 mL, 16.5 mmol, 1.1 equiv) was then added and the reaction was stirred at room temperature for 14 h. The reaction was then quenched with water (30 mL). The aqueous layer was then extracted with CH_2Cl_2 (3 x 50 mL). The combined organics were washed with water (3 x 50 ml) and brine (3 x 50 mL), dried ($MgSO_4$), filtered and concentrated *in vacuo*. The crude product was purified by silica gel chromatography (eluent: Pet. Ether:EtOAc, 80:20) to give the title compound as a yellow solid (2.51 g, 14.2 mmol, 95%).

^{1}H NMR (700 MHz, $CDCl_3$) δ 8.10 (d, *J* = 7.9 Hz, 1H), 7.62 (d, *J* = 8.0 Hz, 1H), 7.29 (d, *J* = 3.2 Hz, 1H), 7.25 (t, *J* = 8.0 Hz, 1H), 7.18 (d, *J* = 3.0 Hz, 1H), 3.87 (s, 3H).^{13}C NMR (176 MHz, $CDCl_3$) δ 140.3, 138.8, 133.3, 122.5, 120.3, 117.3, 116.1, 101.6, 33.3.

Data match literature values.[11]

1-Methyl-1H-indol-4-amine

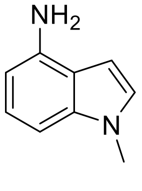

Iron powder (896 mg, 16.0 mmol, 2 equiv) was added portion-wise to a refluxing solution of 1-methyl-4-nitro-1H-indole (1.41 g, 8.00 mmol, 1 equiv) in concentrated HCl (1 mL) and EtOH (20 mL). The reaction was heated under reflux for 20 h and then quenched with saturated $NaHCO_3$. The reaction was then filtered and the filtrate concentrated *in vacuo*. The resulting residue was then dissolved in EtOAc (30 mL) and the organic layer was washed with water (3 x20 mL), dried ($MgSO_4$), filtered and concentrated *in vacuo*. The crude product was purified by silica gel chromatography (eluent: Pet. Ether:EtOAc, 80:20) to give the title compound (164 mg, 1.12 mmol, 14%) as a red oil.

^{1}H NMR (500 MHz, $CDCl_3$) δ 7.12 – 7.05 (m, 1H), 6.99 (dd, *J* = 3.3, 0.9 Hz, 1H), 6.83 (dt, *J* = 8.2, 0.9 Hz, 1H), 6.48 – 6.39 (m, 2H), 3.95 (s, 2H), 3.78 (d, *J* = 0.7 Hz, 3H). ^{13}C NMR (126 MHz, $CDCl_3$) δ 139.5, 137.8, 127.0, 122.8, 117.8, 103.8, 100.5, 97.2, 33.1.

Data match literature values.[12]

Tetrabutylammonium (1-methyl-1H-indol-4-yl)sulfamate

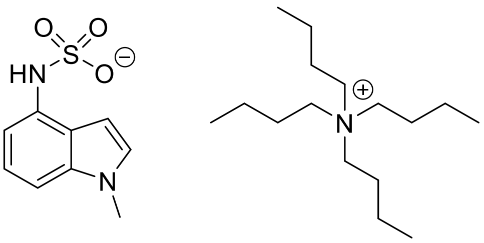

1-Methyl-1H-indol-4-amine (161 mg, 1.10 mmol, 1 equiv), chlorosulfonic acid (128 mg, 73 µL, 1.10 mmol, 1 equiv) and tetrabutylammonium hydrogen sulfate (373 mg, 1.10 mmol, 1 equiv) were subject to general procedure 1 to give the title compound (378 mg, 0.807 mmol, 73%) as a red solid.

^{1}H NMR (400 MHz, MeOD-d_4) δ 7.22 (d, *J* = 7.6 Hz, 1H), 7.14 – 7.02 (m, 2H), 6.97 (d, *J* = 8.1 Hz, 1H), 6.60 (d, *J* = 3.1 Hz, 1H), 3.76 (s, 3H), 3.20 – 3.06 (m, 8H), 1.57 (p, *J* = 7.8 Hz, 8H), 1.37 (h, *J* = 7.4 Hz, 8H), 1.00 (t, *J* = 7.3 Hz, 12H). ^{13}C NMR (101 MHz, MeOD-d_4) δ 137.6, 133.9, 127.1, 121.7, 119.4, 106.5, 102.3, 97.4, 57.9, 31.6, 23.3, 12.6. HRMS (ESI) calcd for $[C_9H_9N_2O_3S]^-$: 225.0339. Found: 225.0333.

7. Synthesis of Aminating Agents

tert-Butyl ((3,5-bis(trifluoromethyl)benzoyl)oxy)carbamate

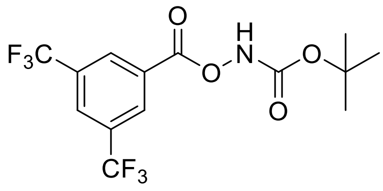

(3,5-Bis(trifluoromethyl)benzoyl) chloride (6.09 g, 3.99 mL, 22.0 mmol, 1.1 equiv), *tert*-butyl *N*-hydroxycarbamate (2.66 g, 20.0 mmol, 1 equiv) and triethylamine (2.22 g, 3.06 mL, 22.0 mmol, 1.1 equiv) was subject to **GP-2**. The crude product was purified by silica gel chromatography (eluent: Hexane:EtOAc, 96:4-93:7) to give the title compound as a white solid (5.13 g, 18.8 mmol, 94%).

^{1}H NMR (400 MHz, $CDCl_3$) δ 8.55 (s, 2H), 8.37 (br s, 1H), 8.15 (s, 1H), 1.54 (s, 9H). ^{13}C NMR (101 MHz, $CDCl_3$) δ 163.6, 155.2, 132.6 (q, J = 34.3 Hz), 130.0 (q, J = 3.9 Hz), 129.3, 127.6 – 127.2 (m), 122.6 (q, J = 273.0 Hz), 84.1, 27.9. ^{19}F NMR (376 MHz, $CDCl_3$) δ -63.20. HRMS (ESI) calcd for $[C_{14}H_{13}F_6NO_4 + Na]^+$: 396.0641. Found: 396.0632.

_O_-(3,5-Bis(trifluoromethyl)benzoyl)hydroxylammonium trifluoromethanesulfonate (2a)

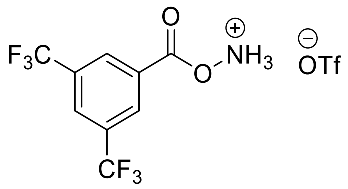

tert-Butyl ((3,5-bis(trifluoromethyl)benzoyl)oxy)carbamate (3.55 g, 13.0 mmol, 1 equiv) and triflic acid (2.15 g, 1.26 mL, 14.3 mmol, 1.1 equiv) were subject to **GP-3** to give the title compound as a white solid (5.21 g, 12.3 mmol, 95%).

^{1}H NMR (400 MHz, CD_3CN) δ 9.68 (br s, 3H), 8.55 (s, 2H), 8.39 (s, 1H,). ^{13}C NMR (101 MHz, CD_3CN) δ 160.7, 132.2 (q, J = 34.5 Hz), 130.9 – 130.1 (m), 128.9 (p, J = 3.7 Hz), 127.2, 122.7 (q, J = 272.3 Hz), 120.4 (q, J = 319.0 Hz). ^{19}F NMR (376 MHz, MeOD-d_4) δ -64.60, -80.14. HRMS (ESI) calcd for $[C_9H_5F_6NO_2 + H]^+$: 274.0297. Found: 274.0291.

_O_-Benzoylhydroxylammonium trifluoromethanesulfonate (2b)

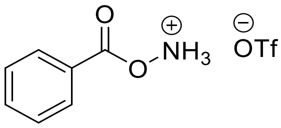

Benzoyl chloride (3.10 g, 2.58 mL, 22.0 mmol, 1.1 equiv), *tert*-butyl *N*-hydroxycarbamate (2.66 g, 20.0 mmol, 1 equiv) and triethylamine (2.22 g, 3.06 mL, 22.0 mmol, 1.1 equiv) was subject to **GP-2**. Crude *tert*-Butyl (benzoyloxy)carbamate was used directly in the next step.

Crude *tert*-Butyl (benzoyloxy)carbamate (1.19 g, 5.02 mmol, approx 1 equiv) and triflic acid (830 mg, 0.52 mL, 5.53 mmol, 1.1 equiv) were subject to **GP-3** to give the title compound as a white solid (1.15 g, 4.00 mmol, 80%).

^{1}H NMR (400 MHz, MeOD) δ 8.07 (d, *J* = 7.0 Hz, 2H), 7.76 (t, *J* = 7.5 Hz, 1H), 7.59 (t, *J* = 7.8 Hz, 2H). ^{13}C NMR (101 MHz, MeOD) δ 163.1, 135.1, 129., 129.0, 124.7, 120.4 (q, *J* = 318.4 Hz). ^{19}F NMR (376 MHz, MeOD) δ -80.14. HRMS (ESI) calcd for $[C_7H_8N_2O_2 + H]^+$: 138.0550. Found: 138.0554.

tert-Butyl ((4-nitrobenzoyl)oxy)carbamate

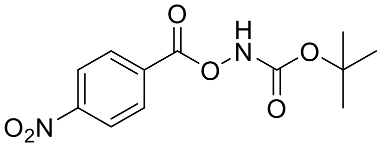

4-Nitrobenzoyl chloride (4.08 g, 22.0 mmol, 1.1 equiv), *tert*-butyl *N*-hydroxycarbamate (2.66 g, 20.0 mmol, 1 equiv) and triethylamine (2.22 g, 3.06 mL, 22.0 mmol, 1.1 equiv) was subject to **GP-2**. The crude product was purified by silica gel chromatography (eluent: Hexane:EtOAc, 90:10) to give the title compound as a white solid (3.89 g, 13.8 mmol, 69%).

^{1}H NMR (400 MHz, $CDCl_3$) δ 8.33 (d, *J* = 8.6 Hz, 2H), 8.27 (d, *J* = 9.3 Hz, 3H), 1.51 (s, 9H). ^{13}C NMR (101 MHz, $CDCl_3$) δ 164.3, 155.2, 151.1, 132.4, 131.1, 123.8, 84.0, 28.0.

Data match literature values.[13]

O-(4-Nitrobenzoyl)hydroxylammonium trifluoromethanesulfonate (2c)

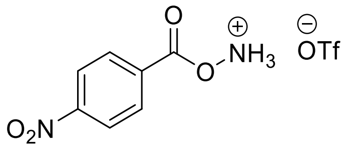

tert-Butyl (4-nitrobenzoyl)oxy)carbamate (850 mg, 3.01 mmol, 1 equiv) and triflic acid (500 mg, 0.32 mL, 3.33 mmol, 1,1 equiv) were subject to **GP-3** to give the title compound as a white solid (850 mg, 2.56 mmol, 86%).

^{1}H NMR (400 MHz, CD_3CN) δ 10.49 (br s, 3H), 8.33 (d, *J* = 8.9 Hz, 2H), 8.22 (d, *J* = 8.9 Hz, 2H). ^{13}C NMR (101 MHz, CD_3CN) δ 161.5, 151.8, 131.4, 129.7, 124.2, 120.3 (q, *J* = 318.8 Hz), 117.6. ^{19}F NMR (376 MHz, DMSO-d_6) δ -77.74. HRMS (ESI) calcd for $[C_7H_6N_2O_4 + H]^+$: 183.0400. Found: 183.0405.

***O*-(4-Methoxybenzoyl)hydroxylammonium trifluoromethanesulfonate (2d)**

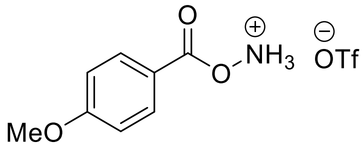

4-Methoxybenzoyl chloride (3.74 g, 2.97 mL, 22.0 mmol, 1.1 equiv), *tert*-butyl *N*-hydroxycarbamate (2.66 g, 20.0 mmol, 1 equiv) and triethylamine (2.22 g, 3.06 mL, 22.0 mmo, 1.1 equiv) was subject to **GP-2**. The crude product was then dissolved in CH_2Cl_2 (50 mL) and the resulting solution was cooled to 0℃. Triflic acid (3.00 g, 1.76 mL, 20.0 mmol, 1 equiv) was then added dropwise. The reaction was allowed to warm to room temperature and was stirred for 1 h. The resulting precipitate was then isolated by filtration to give the title compound as a white solid (4.23 g, 13.3 mmol, 67% over two steps).

^{1}H NMR (400 MHz, MeOD) δ 8.13 – 7.91 (m, 2H), 7.19 – 6.98 (m, 2H), 3.90 (s, 3H). ^{13}C NMR (101 MHz, MeOD) δ 165.5, 162.7, 132.0, 120.4 (q, *J* = 318.4 Hz) 116.3, 114.3, 54.9. ^{19}F NMR (376 MHz, DMSO-d_6) δ -77.74. HRMS (ESI) calcd for $[C_8H_{10}NO_3 + K]^+$: 207.0300. Found: 207.0292.

***tert*-Butyl (pivaloyloxy)carbamate**

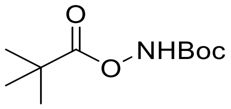

A stirred solution of pivalic anhydride (4.09 g, 4.46 mL, 22.0 mmol, 2.2 equiv), *tert*-butyl *N*-hydroxycarbamate (1.33 g, 10.0 mmol, 1 equiv) in dry $CHCl_3$ (25 mL) was heated under reflux for 72 h. The reaction was allowed to cool to room temperature and aqueous $NaHCO_3$ was added. The aqueous layer was then extracted with CH_2Cl_2 (3 x 30 mL). The combined organics were then washed with aqueous $NaHCO_3$ (2 x 20 mL), dried ($MgSO_4$), filtered and concentrated *in vacuo*. The crude product was purified by silica gel chromatography (eluent: Hexane:EtOAc, 95:5) to give the title compound as a white solid (1.66 g, 7.65 mmol, 76%).

^{1}H NMR (400 MHz, $CDCl_3$) δ 7.83 (br s, 1H), 1.47 (s, 9H), 1.28 (s, 9H).^{13}C NMR (101 MHz, $CDCl_3$) δ 177.7, 155.7, 83.0, 38.1, 28.0, 26.9.

Data match literature values.[13]

***O*-Pivaloylhydroxylammonium trifluoromethanesulfonate (2e)**

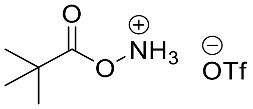

tert-Butyl (pivaloyloxy)carbamate (1.50 g, 6.91 mmol, 1 equiv) and triflic acid (1.04 g, 0.61 mL, 6.93 mmol, 1 equiv) were subject to **GP-3** to give the title compound as a white solid (1.48 g, 5.54 mmol, 80%).

^{1}H NMR (400 MHz, CD_3CN) δ 1.31 (s, 9H). ^{13}C NMR (101 MHz, CD_3CN) δ 174.2, 120.4 (q, J = 319.0 Hz), 38.1, 25.8. ^{19}F NMR (376 MHz, CD_3CN) δ -80.39.

Data match literature values.[14]

***tert*-Butyl ((methylsulfonyl)oxy)carbamate**

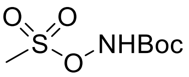

The title compound was prepared according to a literature procedure.[2]

O-(Methylsulfonyl)hydroxylammonium trifluoromethanesulfonate (2f)

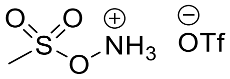

The title compound was prepared according to a literature procedure.[2]

tert-Butyl (((4-nitrophenyl)sulfonyl)oxy)carbamate

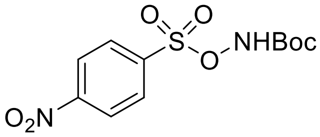

4-Nitrobenzenesulfonyl chloride (2.44 g, 11.0 mmol, 1.1 equiv), tert-butyl N-hydroxycarbamate (1.33 g, 10.0 mmol, 1 equiv) and triethylamine (1.5 mL, 11.0 mmol, 1.1 equiv) was subject to GP-2. The crude product was purified by recrystallisation from Pet. Ether/EtOAc to yield the title compound as yellow crystals (2.40 g, 7.54 mmol, 75%).

^{1}H NMR (400 MHz, $CDCl_3$) δ 8.43 (d, J = 8.6 Hz, 2H), 8.24 (d, J = 8.6 Hz, 2H), 7.72 (br s, 1H), 1.34 (s, 9H); ^{13}C NMR (101 MHz, $CDCl_3$) δ 153.7, 151.2, 139.3, 131.1, 124.0, 84.8, 27.8.

O-((4-Nitrophenyl)sulfonyl)hydroxylammonium trifluoromethanesulfonate (2g)

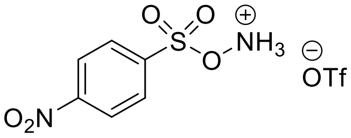

tert-Butyl (((4-nitrophenyl)sulfonyl)oxy)carbamate (2.40 g, 7.54 mmol, 1 equiv) and triflic acid (0.73 mL, 8.29 mmol, 1.1 equiv) were subject to GP-3 to give the title compound as a yellow solid (2.20 g, 5.97 mmol, 81%).

^{1}H NMR (400 MHz, DMSO-d^6) δ 9.18 (br s, 3H), 8.21 (d, J = 8.3 Hz, 2H), 7.84 (d, J = 8.3 Hz, 2H); ^{13}C NMR (101 MHz, DMSO-d^6) δ 154.4, 147.8, 127.4, 123.9, 121.1 (q, J = 322.2 Hz); ^{19}F NMR (376 MHz, DMSO-d^6) δ -78.7.

Data match literature values.[3]

tert-Butyl hydroxy(methyl)carbamate

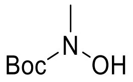

Triethylamine (6.9 mL, 49.9 mmol, 2 equiv) and di-*tert*-butyl dicarbonate (5.45 g, 25.0 mmol, 1 equiv) were added sequentially to a stirred suspension of *N*-methylhydroxylamine hydrochloride (2.09 g, 25.0 mmol, 1 equiv) in dry CH_2Cl_2 (25 mL) at 0°C. The reaction was stirred under argon at room temperature for 16 h. Water (50 mL) was then added and the aqueous layer was then extracted with CH_2Cl_2 (3 x 50 mL). The combined organics were then washed with water (3 x 50 mL), dried ($MgSO_4$), filtered and concentrated *in vacuo* to give crude *tert*-butyl hydroxy(methyl)carbamate as a colourless oil. Crude *tert*-butyl hydroxy(methyl)carbamate was subsequently used without further purification.

tert-Butyl ((4-methoxybenzoyl)oxy)(methyl)carbamate

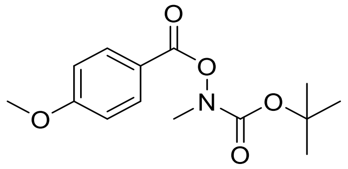

Dry CH_2Cl_2 (50 mL) was added to crude *tert*-butyl hydroxy(methyl)carbamate followed by triethylamine (3.5 mL, 25.3 mmol, 1 equiv) and 4-methoxybenzoyl chloride (4.27 g, 25.0 mmol, 1 equiv). The reaction was then stirred under argon at room temperature for 4 h. The reaction was then quenched with aqueous $NaHCO_3$ and the aqueous layer was extracted with CH_2Cl_2 (3 x 50 mL). The combined organics were then dried ($MgSO_4$), filtered and concentrated *in vacuo*. The crude residue was then purified by silica gel chromatography (eluent: Pet. Ether:EtOAc, 98:2-92:8) to give the title compound (5.19 g, 18.5 mmol, 74%) as a colourless oil.

^{1}H NMR (400 MHz, $CDCl_3$) δ 8.03 (d, *J* = 8.8 Hz, 2H), 6.94 (d, *J* = 8.8 Hz, 2H), 3.87 (s, 3H), 3.33 (s, 3H), 1.46 (s, 9H). ^{13}C NMR (101 MHz, $CDCl_3$) δ 164.4, 164.0, 155.4, 132.0, 119.8, 113.9, 82.2, 55.5, 37.9, 28.1.

Data match literature values.[15]

O-(4-Methoxybenzoyl)-N-methylhydroxylammonium trifluoromethanesulfonate (3d)

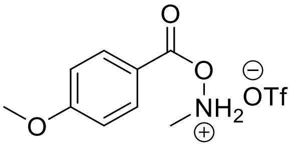

tert-Butyl ((4-methoxybenzoyl)oxy)(methyl)carbamate (2.00 g, 7.11 mmol, 1 equiv) and triflic acid (1.17 g, 0.69 mL, 7.82 mmol, 1.1 equiv) were subject to **GP-3** to give the title compound as a white solid (2.00 g, 6.04 mmol, 85%).

^{1}H NMR (400 MHz, CD_3CN) δ 10.98 (br s, 2H), 8.00 (d, J = 9.0 Hz, 2H), 7.13 – 7.03 (m, 2H), 3.89 (s, 3H), 3.27 (s, 3H). ^{13}C NMR (101 MHz, CD_3CN) δ 165.7, 162.3, 132.5, 120.6 (q, J = 319.3 Hz), 115.6, 114.7, 65.3, 55.7, 36.3, 14.6. ^{19}F NMR (376 MHz, CD_3CN) δ -79.35. HRMS (ESI) calcd for $[C_9H_{11}NO_3 + H]^+$: 182.0812. Found: 182.0808.

***tert*-Butyl methyl((4-nitrobenzoyl)oxy)carbamate**

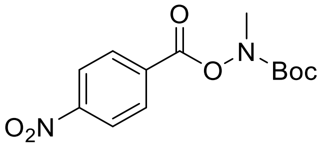

Dry CH_2Cl_2 (20 mL) was added to crude *tert*-butyl hydroxy(methyl)carbamate (1.47 g, 10.0 mmol, 1 equiv) followed by triethylamine (1.38 mL, 10.0 mmol, 1 equiv) and 4-nitrobenzoyl chloride (1.86 g, 10.0 mmol, 1 equiv). The reaction was then stirred under argon at room temperature for 2 h. The reaction was then quenched with aqueous $NaHCO_3$ and the aqueous layer was extracted with CH_2Cl_2 (3 x 50 mL). The combined organics were then dried ($MgSO_4$), filtered and concentrated *in vacuo*. The crude residue was then purified by silica gel chromatography (eluent: Pet. Ether:EtOAc, 90:10) to give the title compound (1.42 g, 4.79 mmol, 48%) as a white solid.

^{1}H NMR (400 MHz, $CDCl_3$) δ 8.31 (d, J = 8.5 Hz, 2H), 8.24 (d, J = 8.5 Hz, 2H), 3.35 (s, 3H), 1.46 (s, 9H). ^{13}C NMR (101 MHz, $CDCl_3$) δ 163.0, 155.1, 1510, 133.1, 131.0, 123.7, 82.9, 38.2, 28.0. HRMS (ESI) calcd for $[C_{13}H_{16}N_2O_6 + Na]^+$: 319.0901. Found: 319.0895.

_N_-Methyl-_O_-(4-nitrobenzoyl)hydroxylammonium trifluoromethanesulfonate (3b)

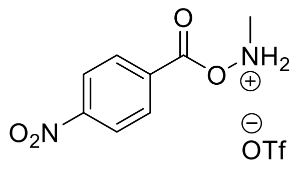

tert-Butyl methyl((4-nitrobenzoyl)oxy)carbamate (1.20 g, 4.05 mmol, 1 equiv) and triflic acid (595 mg, 0.35 mL, 3.97 mmol, 1 equiv) were subject to **GP-3** to give the title compound as a white solid (1.36 g, 3.93 mmol, 99%).

^{1}H NMR (400 MHz, CD_3CN) δ 8.37 (d, *J* = 8.9 Hz, 2H), 8.25 (d, *J* = 8.9 Hz, 2H), 7.82 (br s, 2H), 3.30 (s, 3H). ^{13}C NMR (101 MHz, CD_3CN) δ 161.5, 151.9, 131.4, 129.7, 124.2, 120.6 (q, *J* = 319.4 Hz), 117.4, 36.3. ^{19}F NMR (376 MHz, CD_3CN) δ -80.32. HRMS (ESI) calcd for $[C_8H_8N_2O_4 + H]^+$: 197.0562. Found: 197.0557.

***tert*-Butyl ((3,5-bis(trifluoromethyl)benzoyl)oxy)(methyl)carbamate**

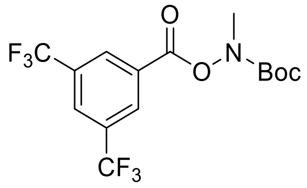

Dry CH_2Cl_2 (20 mL) was added to crude *tert*-butyl hydroxy(methyl)carbamate (1.47 g, 10.0 mmol, 1 equiv) followed by triethylamine (1.38 mL, 10.0 mmol, 1 equiv) and 3,5-(bistrifluoromethyl)benzoyl chloride (2.77 g, 1.81 mL, 10.0 mmol, 1 equiv). The reaction was then stirred under argon at room temperature for 2 h. The reaction was then quenched with aqueous $NaHCO_3$ and the aqueous layer was extracted with CH_2Cl_2 (3 x 50 mL). The combined organics were then dried ($MgSO_4$), filtered and concentrated *in vacuo*. The crude residue was then purified by silica gel chromatography (eluent: Pet. Ether:EtOAc, 99:1-95:5) to give the title compound (2.80 g, 7.23 mmol, 72%) as a white solid.

^{1}H NMR (400 MHz, $CDCl_3$) δ 8.51 (s, 2H), 8.11 (s, 1H), 3.37 (s, 3H), 1.48 (s, 9H). ^{13}C NMR (101 MHz, $CDCl_3$) δ 162.2, 155.0, 132.5 (q, *J* = 34.3 Hz), 123.0, 129.9 (q, *J* = 3.9 Hz), 127.1 (hept, *J* = 3.7 Hz), 122.6 (q, *J* = 272.9 Hz), 83.0, 38.2, 27.9. ^{19}F NMR (376 MHz, $CDCl_3$) δ -63.29. HRMS (ESI) calcd for $[C_{10}H_8F_6NO_2 + H]^+$: 288.0454. Found: 288.0462.

O-(3,5-bis(trifluoromethyl)benzoyl)-N-methylhydroxylammonium trifluoromethanesulfonate (3c)

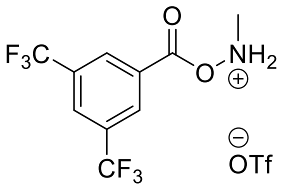

tert-Butyl ((3,5-(bistrifluoromethyl)benzoyl)oxy)(methyl)carbamate (1.52 g, 3.92 mmol, 1 equiv) and triflic acid (578 mg, 0.34 mL, 3.85 mmol, 1 equiv) were subject to **GP-3** to give the title compound as a white solid (1.52 g, 3.48 mmol, 90%).

^{1}H NMR (400 MHz, CD_3CN) δ 10.96 (br s, 2H), 8.57 (s, 2H), 8.39 (s, 1H), 3.36 (s, 3H). ^{13}C NMR (101 MHz, CD_3CN) δ 160.7, 132.2 (q, *J* = 34.5 Hz), 131.0 – 129.8 (m), 128.9 (hept, *J* = 3.6 Hz), 127.1, 122.7 (q, *J* = 272.3 Hz), 120.4 (q, *J* = 318.9 Hz), 36.3. ^{19}F NMR (376 MHz, CD_3CN) δ -64.58, -80.33. HRMS (ESI) calcd for $[C_{10}H_7F_6NO_2 + H]^+$: 288.0454. Found: 288.0454.

***tert*-Butyl ((4-methoxybenzoyl)oxy)carbamate**

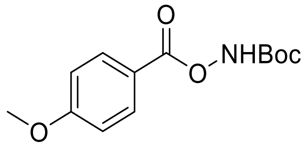

4-Methoxybenzoyl chloride (11.1 g, 65.0 mmol), *tert*-butyl *N*-hydroxycarbamate (8.65 g, 65.0 mmol) and triethylamine (8.99 mL, 65.0 mmol) was subject to **GP-2** to give the title compound (15.7 g, 58.7 mmol, 90%) which was used without further purification.

^{1}H NMR (400 MHz, $CDCl_3$) δ 8.25 (br s, 1H), 8.03 – 8.01 (m, 2H), 6.93 – 6.90 (m, 2H), 3.84 (s, 3H), 1.48 (s, 9H). ^{13}C NMR (101 MHz, $CDCl_3$) δ 165.8, 164.2, 155.8, 132.1, 119.1, 113.9, 83.1, 55.5, 28.0.

Data match literature values.[13]

***tert*-Butyl ethyl((4-methoxybenzoyl)oxy)carbamate**

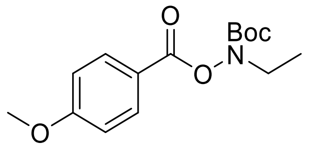

Under an atmosphere of nitrogen, sodium hydride (60% in mineral oil, 720 mg, 18.0 mmol, 1.2 equiv) was added to a stirred solution of *tert*-butyl ((4-methoxybenzoyl)oxy)carbamate (4.01 g, 15.0 mmol, 1 equiv) in dry DMF (30 mL). The reaction was stirred at 0°C for 30 min, then ethyl iodide (2.87 g, 1.48 mL, 18.4 mmol, 1.2 equiv) was added. The reaction was allowed to warm to room temperature and was stirred for 18 h. The reaction was then quenched with water (20 mL) and the aqueous layer was extracted with EtOAc (3 x 100 mL). The combined organic layers were then washed with water (3 x 100 mL) and brine (3 x 100 mL), dried ($MgSO_4$), filtered and concentrated *in vacuo*. The crude residue was then purified by silica gel chromatography (eluent: Pet. Ether:EtOAc, 98:2 – 93:7) to give the title compound (2.70 g, 9.14 mmol, 61%) as a colourless oil.

^{1}H NMR (400 MHz, $CDCl_3$) δ 8.04 (d, *J* = 8.9 Hz, 2H), 6.94 (d, *J* = 8.9 Hz, 2H), 3.87 (s, 3H), 3.73 (q, *J* = 7.1 Hz, 2H), 1.46 (s, 9H), 1.23 (t, *J* = 7.1 Hz, 3H). ^{13}C NMR (101 MHz, $CDCl_3$) δ 164.4, 164.0, 155.0, 132.0, 119.9, 113.9, 82.1, 55.5, 45.6, 28.2, 12.3. HRMS (ESI) calcd for $[C_{15}H_{21}NO_5 + Na]^+$: 318.1312. Found: 318.1310.

_N_-Ethyl-O-(4-methoxybenzoyl)hydroxylammonium trifluoromethanesulfonate (3e)

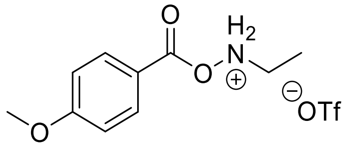

tert-Butyl ethyl((4-methoxybenzoyl)oxy)carbamate (2.07 mg, 7.01 mmol, 1 equiv) and triflic acid (1.05 g, 0.62 mL, 7.03 mmol, 1 equiv) were subject to **GP-3** to give the title compound as a white solid (2.22 g, 6.42 mmol, 91%).

^{1}H NMR (400 MHz, CD_3CN) δ 8.03 (d, *J* = 9.0 Hz, 2H), 7.10 (d, *J* = 9.0 Hz, 2H), 3.90 (s, 3H), 3.64 (q, *J* = 7.2 Hz, 2H), 1.41 (t, *J* = 7.3 Hz, 3H). ^{13}C NMR (101 MHz, CD_3CN) δ 165.7, 162.5, 132.5, 120.8 (q, *J* = 320.2 Hz), 115.6, 114.8, 55.7, 46.3, 8.1. ^{19}F NMR (376 MHz, CD_3CN) δ -80.33. HRMS (ESI) calcd for $[C_{10}H_{13}NO_3 + H]^+$: 196.0968. Found: 196.0967.

tert-Butyl ((4-methoxybenzoyl)oxy)(propyl)carbamate

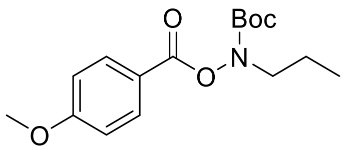

Under an atmosphere of nitrogen, sodium hydride (60% in mineral oil, 240 mg, 6.00 mmol, 1.2 equiv) was added to a stirred solution of *tert*-butyl ((4-methoxybenzoyl)oxy)carbamate (1.34 g, 5.01 mmol, 1 equiv) in dry DMF (10 mL). The reaction was stirred at 0°C for 30 min, then propyl iodide (1.02 g, 0.59 mL, 6.00 mmol, 1.2 equiv) was added. The reaction was allowed to warm to room temperature and was stirred for 18 h. The reaction was then quenched with water (20 mL) and the aqueous layer was extracted with EtOAc (3 x 50 mL). The combined organic layers were then washed with water (3 x 50 mL) and brine (3 x 50 mL), dried ($MgSO_4$), filtered and concentrated *in vacuo*. The crude residue was then purified by silica gel chromatography (eluent: Pet. Ether:EtOAc, 98:2 – 93:7) to give the title compound (960 mg, 3.10 mmol, 62%) as a colourless oil.

^{1}H NMR (400 MHz, $CDCl_3$) δ 8.08 – 7.97 (m, 2H), 7.00 – 6.86 (m, 2H), 3.87 (s, 3H), 3.64 (t, *J* = 7.1 Hz, 2H), 1.66 (h, *J* = 7.3 Hz, 2H), 1.45 (s, 9H), 0.96 (t, *J* = 7.4 Hz, 3H). ^{13}C NMR (101 MHz, $CDCl_3$) δ 164.3, 164.0, 155.0, 132.0, 119.9, 113.9, 82.0, 55.5, 52.2, 28.1, 20.6, 11.2. HRMS (ESI) calcd for $[C_{16}H_{23}NO_5 + Na]^+$: 332.1468. Found: 332.1464.

***O*-(4-Methoxybenzoyl)-*N*-propylhydroxylammonium trifluoromethanesulfonate (3f)**

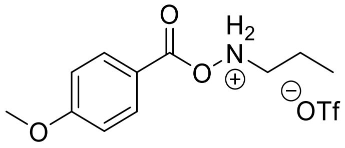

tert-Butyl propyl((4-methoxybenzoyl)oxy)carbamate (804 mg, 2.60 mmol, 1 equiv) and triflic acid (391 mg, 0.23 mL, 2.61 mmol, 1 equiv) were subject to **GP-3** to give the title compound as a white solid (862 mg, 2.40 mmol, 87%).

^{1}H NMR (400 MHz, CD_3CN) δ 8.01 (d, *J* = 9.0 Hz, 2H), 7.09 (d, *J* = 9.0 Hz, 2H), 3.90 (s, 3H), 3.65 – 3.47 (m, 2H), 1.92 – 1.81 (m, 2H), 1.04 (t, *J* = 7.5 Hz, 3H). ^{13}C NMR (101 MHz, CD_3CN) δ

165.7, 162.4, 132.5, 120.6 (q, J = 319.5 Hz), 115.6, 114.8, 55.7, 52.0, 16.9, 10.0. ^{19}F NMR (376 MHz, CD_3CN) δ -79.36. HRMS (ESI) calcd for $[C_{11}H_{15}NO_3 + H]^+$: 210.1125. Found: 210.1126.

tert-Butyl (2-cyanoethyl)((4-methoxybenzoyl)oxy)carbamate

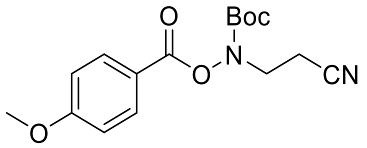

Aqueous potassium hydroxide (672 mg, 12.0 mmol, 1.2 equiv, 50% w/v) and tetrabutylammonium bromide (322 mg, 0.999 mmol, 0.1 equiv) were sequentially added to a stirred solution of *tert*-butyl ((4-methoxybenzoyl)oxy)carbamate (2.67 g, 10.0 mmol, 1 equiv) in toluene (15 mL). Acrylonitrile (530 mg, 0.65 mL, 10.0 mmol, 1 equiv) was then added dropwise and the reaction stirred at room temperature for 20 h. Any remaining acrylonitrile was then removed under a stream of air and water added (50 mL). The aqueous layer was extracted with EtOAc (3 x 50 mL) and the combined organics were dried ($MgSO_4$), filtered and concentrated *in vacuo*. The crude residue was then purified by silica gel chromatography (eluent: Pet. Ether:EtOAc, 92:8 – 80:20) to give the title compound (1.42 g, 4.43 mmol, 44%) as a colourless oil.

^{1}H NMR (400 MHz, $CDCl_3$) δ 8.02 (d, J = 8.9 Hz, 2H), 6.95 (d, J = 9.0 Hz, 2H), 3.98 (t, J = 6.8 Hz, 2H), 3.87 (s, 3H), 2.72 (t, J = 7.5, 2H), 1.45 (s, 9H). ^{13}C NMR (101 MHz, $CDCl_3$) δ 164.3, 153.9, 132.2, 119.0, 117.4, 114.1, 83.3, 55.5, 46.5, 28.0, 16.7. HRMS (ESI) calcd for $[C_{16}H_{20}N_2O_5 + Na]^+$: 343.1264. Found: 343.1270.

***N*-(2-Cyanoethyl)-*O*-(4-methoxybenzoyl)hydroxylammonium trifluoromethanesulfonate (3g)**

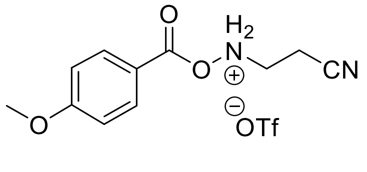

tert-Butyl hexyl((4-methoxybenzoyl)oxy)carbamate (1.42 g, 4.43 mmol, 1 equiv) and triflic acid (663 mg, 0.39 mL, 4.42 mmol, 1 equiv) were subject to **GP-3** to give the title compound as a white solid (1.25 g, 3.38 mmol, 76%).

^{1}H NMR (400 MHz, CD_3CN) δ 10.59 (br s, 2H), 8.09 (d, *J* = 9.1 Hz, 2H), 7.10 (d, *J* = 7.6 Hz, 2H), 4.00 (t, *J* = 6.3 Hz, 2H), 3.94 – 3.87 (s, 3H), 3.15 (t, *J* = 6.5 Hz, 2H).^{13}C NMR (101 MHz, CD_3CN) δ 165.8, 162.3, 132.8, 120.4 (q, *J* = 319.0 Hz), 116.9, 115.3, 114.8, 55.8, 46.2, 13.3. ^{19}F NMR (376 MHz, CD_3CN) δ -79.37. HRMS (ESI) calcd for $[C_{11}H_{12}N_2O_3 + H]^+$: 221.0921. Found: 221.0917.

tert-Butyl hexyl((4-methoxybenzoyl)oxy)carbamate

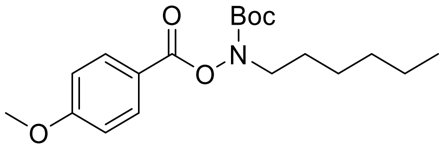

Under an atmosphere of nitrogen, sodium hydride (60% in mineral oil, 480 mg, 12.0 mmol, 1.2 equiv) was added to a stirred solution of *tert*-butyl ((4-methoxybenzoyl)oxy)carbamate (2.67 g, 10.0 mmol, 1 equiv) in dry DMF (20 mL). The reaction was stirred at 0℃ for 30 min, then hexyl iodide (1.77 mL, 2.54 g, 12.4 mmol, 1.2 equiv) was added. The reaction was allowed to warm to room temperature and was stirred for 18 h. The reaction was then quenched with water (20 mL) and the aqueous layer was extracted with EtOAc (3 x 100 mL). The combined organic layers were then washed with water (3 x 100 mL) and brine (3 x 100 mL), dried ($MgSO_4$), filtered and concentrated *in vacuo*. The crude residue was then purified by silica gel chromatography (eluent: Pet. Ether:EtOAc, 98:2 – 93:7) to give the title compound (1.88 g, 5.35 mmol, 54%) as a colourless oil.

^{1}H NMR (400 MHz, $CDCl_3$) δ 8.03 (d, *J* = 8.8 Hz, 2H), 6.94 (d, *J* = 8.8 Hz, 2H), 3.88 (s, 3H), 3.67 (t, *J* = 7.2 Hz, 2H), 1.63 (p, *J* = 7.3 Hz, 2H), 1.46 (s, 9H), 1.42 – 1.25 (m, 6H), 0.96 – 0.77 (m, 3H). ^{13}C NMR (101 MHz, $CDCl_3$) δ 164.3, 164.0, 155.0, 132.0, 119.9, 113.9, 82.0, 55.5, 50.6, 31.4, 28.2, 27.2, 26.3, 22.5, 14.0. HRMS (ESI) calcd for $[C_{19}H_{29}NO_5 + Na]^+$: 374.1943. Found: 374.1939.

***N*-Hexyl-O-(4-methoxybenzoyl)hydroxylammonium trifluoromethanesulfonate (3h)**

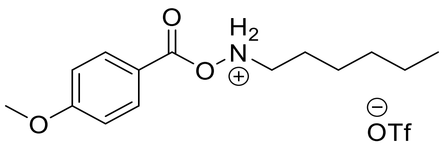

tert-Butyl hexyl((4-methoxybenzoyl)oxy)carbamate (1.41 g, 4.01 mmol, 1 equiv) and triflic acid (595 mg, 0.35 mL, 3.97 mmol, 1 equiv) were subject to **GP-3** to give the title compound as a white solid (1.41 g, 3.51 mmol, 88%).

^{1}H NMR (400 MHz, CD_3CN) δ 9.92 (br s, 2H), 8.02 (d, *J* = 8.9 Hz, 1H), 7.10 (d, *J* = 9.0 Hz, 1H), 3.90 (s, 3H), 3.66 – 3.49 (m, 2H), 1.90 – 1.76 (m, 2H), 1.49 – 1.38 (m, 2H), 1.33 (dq, *J* = 7.2, 3.6 Hz, 4H), 0.95 – 0.85 (m, 3H). ^{13}C NMR (101 MHz, CD_3CN) δ 165.7, 162.4, 132.5, 120.6 (q, *J* = 319.6 Hz), 115.6, 114.8, 55.7, 50.6, 30.8, 25.5, 23.1, 22.0, 13.2. ^{19}F NMR (376 MHz, CD_3CN) δ -79.35. HRMS (ESI) calcd for $[C_{14}H_{21}NO_3 + H]^+$: 252.1594. Found: 252.1593.

8. NHMe and NH-Alkyl Transfer Products

_N_-Methylbenzene-1,2-diamine (5a)

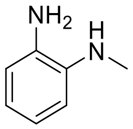

Tetrabutylammonium phenylsulfamate **1a** (207 mg, 0.499 mmol, 1 equiv), **3d** (248 mg, 0.749 mmol, 1.5 equiv), $FeBr_2$ (16.2 mg, 0.0751 mmol, 0.15 equiv) were subject to **GP-4**. Purification by silica gel chromatography (eluent: Pet. Ether:EtOAc, 90:10 – 65:35) gave the title compound (37.2 mg, 0.304 mmol, 61%, *o*:*p* >20:1) as a red oil.

Note: NMR analysis of the crude reaction mixture suggested an *o*:*p* ratio of 17:1. However, the minor isomer was unable to be isolated by column chromatography to confirm this.

^{1}H NMR (400 MHz, $CDCl_3$) δ 6.89 – 6.81 (m, 1H), 6.76 – 6.65 (m, 3H), 3.53 (br s, 3H), 2.87 (s, 3H). ^{13}C NMR (101 MHz, $CDCl_3$) δ 138.4, 134.2, 120.8, 118.8, 116.4, 111.4, 31.1.

Data match literature values.[16]

_N_1,3-dimethylbenzene-1,2-diamine (5b)

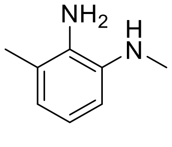

Tetrabutylammonium *o*-tolylsulfamate **1b** (214 mg, 0.499 mmol, 1 equiv), **3d** (248 mg, 0.749 mmol, 1.5 equiv), $FeBr_2$ (16.2 mg, 0.0751 mmol, 0.15 equiv) were subject **GP-4**. Purification by silica gel chromatography (eluent: Pet. Ether:EtOAc, 90:10 – 70:30) gave the title compound (48.6 mg, 0.357 mmol, 71%, *o*:*p* >20:1) as a red oil.

Note: NMR analysis of the crude reaction mixture suggested an *o*:*p* ratio of 16:1. However, the minor isomer was unable to be isolated by column chromatography to confirm this.

^{1}H NMR (400 MHz, $CDCl_3$) δ 6.78 (t, *J* = 7.7 Hz, 1H), 6.65 (d, *J* = 8.2 Hz, 1H), 6.61 (dd, *J* = 7.9, 1.4 Hz, 1H), 3.35 (br s, 3H), 2.87 (s, 3H), 2.22 (s, 3H). ^{13}C NMR (101 MHz, $CDCl_3$) δ 137.9, 132.8, 122.7, 120.9, 119.6, 109.6, 31.4, 17.5. HRMS (ESI) calcd for $[C_8H_{12}N_2 + Na]^+$: 159.0893. Found: 159.0892

3-Isopropyl-*N*1-methylbenzene-1,2-diamine (5c)

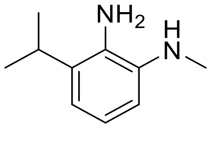

Tetrabutylammonium (2-isopropylphenyl)sulfamate **1c** (228 mg, 0.499 mmol, 1 equiv), **3d** (248 mg, 0.749 mmol, 1.5 equiv), $FeBr_2$ (16.2 mg, 0.0751 mmol, 0.15 equiv) were subject to **GP-4**. Purification by silica gel chromatography (eluent: Pet. Ether:EtOAc, 95:5 – 75:25) gave the title compound (58.7 mg, 0.357 mmol, 71%, *o*:*p* >20:1) as a yellow oil.

Note: NMR analysis of the crude reaction mixture suggested an *o*:*p* ratio of 14:1. However, the minor isomer was unable to be isolated by column chromatography to confirm this.

^{1}H NMR (400 MHz, $CDCl_3$) δ 6.88 (t, *J* = 7.8 Hz, 1H), 6.76 (dd, *J* = 7.8, 1.3 Hz, 1H), 6.62 (dd, *J* = 7.8, 1.3 Hz, 1H), 3.37 (br s, 3H), 2.99 (hept, *J* = 6.8 Hz, 1H) 2.88 (s, 3H) 1.29 (d, *J* = 6.8 Hz, 6H). ^{13}C NMR (101 MHz, $CDCl_3$) δ 138.5, 133.1, 131.5, 119.9, 115.6, 109.2, 31.4, 28.0, 22.6. HRMS (ESI) calcd for $[C_{10}H_{16}N_2 + H]^+$: 165.1386. Found: 165.1384.

3-*tert*-Buytl-*N*1-methylbenzene-1,2-diamine (5d)

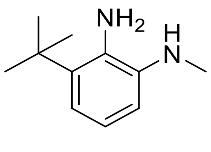

Tetrabutylammonium (2-*tert*-butylphenyl)sulfamate **1d** (236 mg, 0.501 mmol, 1 equiv), **3d** (248 mg, 0.749 mmol, 1.5 equiv), $FeBr_2$ (16.2 mg, 0.0751 mmol, 0.15 equiv) were subject to **GP-4**. Purification by silica gel chromatography was performed twice (eluent: Pet. Ether:EtOAc, 95:5 – 70:30 then Pet. Ether:CH_2Cl_2 40:60 – 0:100) to give the title compound (47.5 mg, 0.266 mmol, 53%, *o*:*p* >20:1) as a yellow oil.

Note: NMR analysis of the crude reaction mixture suggested an *o*:*p* ratio of 17:1. However, the minor isomer was unable to be isolated by column chromatography to confirm this.

^1H NMR (400 MHz, $CDCl_3$) δ 6.91 – 6.78 (m, 2H), 6.69 (dd, *J* = 7.5, 1.6 Hz, 1H), 3.67 (br s, 3H), 2.88 (s, 3H), 1.46 (s, 9H). ^{13}C NMR (101 MHz, $CDCl_3$) δ 138.9, 134.3, 133.3, 119.3, 117.2, 110.2, 34.2, 31.6, 30.1. HRMS (ESI) calcd for $[C_{11}H_{17}N_2 + H]^+$: 178.1465. Found: 178.1461.

N1,6-Dimethylbenzene-1,2-diamine (5e-i) and N1,4-dimethylbenzene-1,2-diamine (5e-ii)

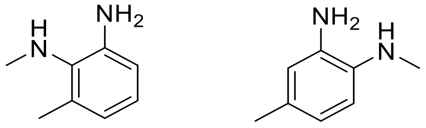

5e-i **5e-ii**

Tetrabutylammonium *m*-tolylsulfamate **1e** (214 mg, 0.499 mmol, 1 equiv), **3d** (248 mg, 0.749 mmol, 1.5 equiv), $FeBr_2$ (16.2 mg, 0.0751 mmol, 0.15 equiv) were subject to **GP-4**. Purification by silica gel chromatography (eluent: Pet. Ether:EtOAc, 95:5 – 75:25) gave an inseparable mixture of the title compounds (39.5 mg, 0.290 mmol, 58%, r.r 2.9:1, *o*:*p* >20:1) as a red oil. The NMR spectra for each isomer was deconvoluted from the mixture:

N1,6-Dimethylbenzene-1,2-diamine (5e-i)

^1H NMR (700 MHz, DMSO-d_6) δ 6.62 (td, *J* = 7.9, 2.4 Hz, 1H), 6.48 (d, *J* = 7.5 Hz, 1H), 6.36 (d, *J* = 8.4 Hz, 2H), 3.36 (br s, 3H), 2.54 (s, 3H), 2.18 (s, 3H). ^{13}C NMR (176 MHz, DMSO-d_6) δ 142.7, 135.1, 131.3, 123.2, 119.1, 113.2, 34.3, 18.1.

N1,4-Dimethylbenzene-1,2-diamine (5e-ii)

^1H NMR (700 MHz, DMSO-d_6) δ 6.38 (s, 1H), 6.34 (d, *J* = 8.0 Hz, 1H), 6.28 (dd, *J* = 7.9, 2.4 Hz, 1H), 3.36 (br s, 3H), 2.68 (s, 3H), 2.10 (s, 3H). ^{13}C NMR (176 MHz, DMSO-d_6) δ 135.6, 135.4, 125.5, 118.3, 115.3, 109.8, 30.9, 20.9.

HRMS (ESI) calcd for $[C_8H_{12}N_2 + H]^+$: 137.1073. Found: 137.1076.

6-Isopropyl-*N*1-methylbenzene-1,2-diamine (5f-i) and 4-isopropyl-*N*1-methylbenzene-1,2-diamine (5f-ii)

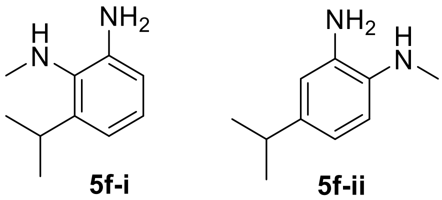

5f-i **5f-ii**

Tetrabutylammonium (3-isopropylphenyl)sulfamate **1f** (228 mg, 0.499 mmol, 1 equiv), **3d** (248 mg, 0.749 mmol, 1.5 equiv), $FeBr_2$ (16.2 mg, 0.0751 mmol, 0.15 equiv) were subject to **GP-4**. Purification by silica gel chromatography (eluent: Pet. Ether:EtOAc, 95:5 – 75:25) gave an inseparable mixture of the title compounds (62.7 mg, 0.382 mmol, 76%, r.r 5.7:1, *o*:*p* >20:1) as a red oil. The NMR spectra of each isomer was deconvoluted from the mixture:

6-Isopropyl-*N*1-methylbenzene-1,2-diamine (5f-i)

^{1}H NMR (400 MHz, DMSO-d_6) δ 6.73 (t, *J* = 7.8 Hz, 1H), 6.48 (d, *J* = 7.9 Hz, 2H), 3.35 (br s, 3H), 3.27 – 3.20 (m, 1H), 2.51 (s, 3H), 1.21 – 1.00 (m, 6H). ^{13}C NMR (101 MHz, DMSO-d_6) δ 143.2, 142.7, 133.5, 124.0, 114.0, 112.5, 35.5, 27.0, 24.7.

4-Isopropyl-*N*1-methylbenzene-1,2-diamine (5f-ii)

^{1}H NMR (400 MHz, DMSO-d_6) δ 6.45 (s, 1H), 6.40 (dd, *J* = 7.9, 1.9 Hz, 1H), 6.31 (d, *J* = 7.9 Hz, 1H), 3.35 (br s, 3H), 2.68 (s, 3H), 2.67 – 2.60 (m, 1H), 1.21 – 1.00 (m, 6H). ^{13}C NMR (101 MHz, DMSO-d_6) δ 137.3, 135.7, 135.5, 115.6, 112.6, 109.7, 33.2, 31.0, 24.8.

HRMS (ESI) calcd for $[C_{10}H_{16}N_2 + H]^+$: 165.1386. Found: 165.1387.

3-(Hex-1-yn-1-yl)-N1-methylbenzene-1,2-diamine (5g)

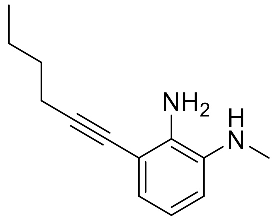

Tetrabutylammonium (2-(hex-1-yn-1-yl)phenyl)sulfamate **1g** (247 mg, 0.499mmol, 1 equiv), **3d** (248 mg, 0.749 mmol, 1.5 equiv), $FeBr_2$ (16.2 mg, 0.0751 mmol, 0.15 equiv) were subject to **GP-4**. Purification by silica gel chromatography (eluent: Pet. Ether:EtOAc, 95:5 – 85:15) gave the title compound (32.7 mg, 0.162 mmol, 32%, *o*:*p* >20:1) as an orange oil.

^{1}H NMR (400 MHz, $CDCl_3$) δ 6.85 (d, *J* = 7.7 Hz, 1H), 6.77 (t, *J* = 7.8 Hz, 1H), 6.63 (d, *J* = 7.8 Hz, 1H), 3.65 (s, 3H), 2.88 (s, 3H), 2.51 (t, *J* = 7.1 Hz, 2H), 1.65 (p, *J* = 7.2 Hz, 2H), 1.53 (h, *J* = 7.4 Hz, 2H), 0.99 (t, *J* = 7.3 Hz, 3H). ^{13}C NMR (101 MHz, $CDCl_3$) δ 137.9, 136.3, 121.6, 119.5, 110.6, 110.0, 95.3, 77.2, 31.1, 31.1, 22.1, 19.4, 13.7. HRMS (ESI) calcd for $[C_{13}H_{18}N_2 + H]^+$: 203.1543. Found: 203.1544.

***N*1,5-Dimethylbenzene-1,2-diamine (5h)**

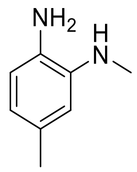

Tetrabutylammonium *p*-tolylsulfamate **1h** (214 mg, 0.499 mmol, 1 equiv), **3d** (248 mg, 0.749 mmol, 1.5 equiv), $FeBr_2$ (16.2 mg, 0.0751 mmol, 0.15 equiv) were subject to **GP-4**. Purification by silica gel chromatography (eluent: Pet. Ether:EtOAc, 90:10 – 70:30) gave the title compound (35.7 mg, 0.262 mmol, 52%, *o*:*p* >20:1) as a red oil.

^{1}H NMR (700 MHz, $CDCl_3$) δ 6.65 (d, *J* = 8.0 Hz, 1H), 6.62 – 6.43 (m, 2H), 3.38 (br s, 3H), 2.89 (s, 3H), 2.31 (s, 3H). ^{13}C NMR (176 MHz, $CDCl_3$) δ 138.9, 131.3, 130.4, 118.7, 116.7, 112.1, 31.1, 21.2. HRMS (ESI) calcd for $[C_8H_{12}N_2 + H]^+$: 137.1073. Found: 137.1076.

***N*2-Methyl-5,6,7,8-tetrahydronaphthalene-1,2-diamine (5i)**

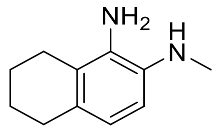

Tetrabutylammonium (5,6,7,8-tetrahydronaphthalen-1-yl)sulfamate **1i** (234 mg, 0.499 mmol, 1 equiv), **3d** (248 mg, 0.749 mmol, 1.5 equiv), $FeBr_2$ (16.2 mg, 0.0751 mmol, 0.15 equiv) were

subject to **GP-4**. Purification by silica gel chromatography (eluent: Pet. Ether (40-60):EtOAc, 90:10 – 65:35) gave the title compound (58.8 mg, 0.334 mmol, 67%, *o*:*p* >20:1) as a yellow oil.

^{1}H NMR (400 MHz, MeOD-d_4) δ 6.51 (d, *J* = 8.3 Hz, 1H), 6.48 (d, *J* = 8.5 Hz, 1H), 2.79 (s, 3H), 2.66 (t, *J* = 6.3 Hz, 2H), 2.51 (t, *J* = 6.6 Hz, 2H), 1.84 (p, *J* = 6.3 Hz, 2H), 1.77 – 1.62 (m, 2H). ^{13}C NMR (101 MHz, MeOD-d_4) δ 134.7, 132.3, 127.9, 121.7, 119.4, 109.8, 30.6, 29.3, 24.0, 23.2, 23.0. HRMS (ESI) calcd for $[C_{11}H_{16}N_2 + H]^+$: 177.1386. Found: 177.1383.

_N_1,3,6-Trimethylbenzene-1,2-diamine (5j)

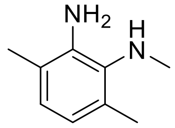

Tetrabutylammonium (2,5-dimethylphenyl)sulfamate **1j** (133 mg, 0.300 mmol, 1 equiv), **3d** (149 mg, 0.450 mmol, 1.5 equiv), $FeBr_2$ (9.7 mg, 0.0450 mmol, 0.15 equiv) were subject to **GP-4**. Purification by silica gel chromatography (eluent: Pet. Ether:EtOAc, 90:10 – 70:30) gave the title compound (34.6 mg, 0.230 mmol, 77%, *o*:*p* >20:1) as a yellow oil.

^{1}H NMR (400 MHz, $CDCl_3$) δ 6.75 (d, *J* = 7.6 Hz, 1H), 6.54 (d, *J* = 7.6 Hz, 1H), 3.64 (br s, 3H), 2.71 (s, 3H), 2.27 (s, 3H), 2.16 (s, 3H). ^{13}C NMR (101 MHz, $CDCl_3$) δ 139.9, 133.8, 128.7, 125.5, 120.8, 119.5, 34.2, 17.5, 17.2. HRMS (ESI) calcd for $[C_9H_{14}N_2 + H]^+$: 151.1230. Found: 151.1234.

_N_1,3,4-Trimethylbenzene-1,2-diamine (5k)

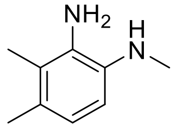

Tetrabutylammonium (2,3-dimethylphenyl)sulfamate **1k** (133 mg, 0.300 mmol, 1 equiv), **3d** (149 mg, 0.450 mmol, 1.5 equiv), $FeBr_2$ (9.7 mg, 0.0450 mmol, 0.15 equiv) were subject to **GP-4**. Purification by silica gel chromatography (eluent: Pet. Ether:EtOAc, 90:10 – 70:30) gave the title compound (29.9 mg, 0.199 mmol, 66%, *o*:*p* >20:1) as an orange oil.

^{1}H NMR (400 MHz, $CDCl_3$) δ 6.68 (d, *J* = 8.0 Hz, 1H), 6.56 (d, *J* = 8.0 H, 1H), 3.54 (br s, 3H,), 2.86 (s, 3H), 2.25 (s, 3H), 2.13 (s, 3H). ^{13}C NMR (101 MHz, $CDCl_3$) δ 135.6, 133.6, 127.7, 121.2, 120.7, 109.7, 31.7, 20.1, 13.2. HRMS (ESI) calcd for $[C_9H_{14}N_2 + H]^+$: 151.1230. Found: 151.1227.

6-Methoxy-N1-methylbenzene-1,2-diamine (5l-i), 4-methoxy-N1-methylbenzene-1,2-diamine (5l-ii) and 5-methoxy-1-methyl-1H-benzo[d]imidazole (5l-iii)

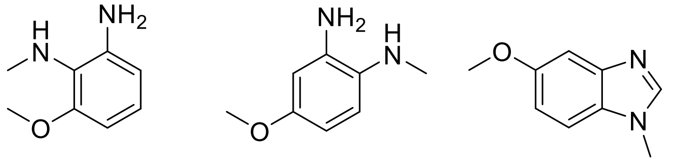

5l-i **5l-ii** **5l-iii**

Tetrabutylammonium (3-methoxyphenyl)sulfamate **1l** (222 mg, 0.499 mmol, 1 equiv), **3d** (248 mg, 0.749 mmol, 1.5 equiv), $FeBr_2$ (16.2 mg, 0.0751 mmol, 0.15 equiv) were subject to **GP-4**. Purification by silica gel chromatography (eluent: Pet. Ether:EtOAc, 90:10 – 50:50) gave the title compounds **5l-i** (11.5 mg, 0.0756 mmol, 15%, *o*:*p* >20:1) and **5l-ii** (21.5 mg, 0.141 mmol, 28%, *o*:*p* >20:1) with a total yield of 43% and a regioisomer ratio of 1.9:1.

6-Methoxy-*N*1-methylbenzene-1,2-diamine (5l-i)

^1H NMR (400 MHz, $CDCl_3$) δ 6.84 (t, *J* = 8.1 Hz, 1H), 6.39 (dd, *J* = 8.1, 1.2 Hz, 1H), 6.34 (dd, *J* = 8.2, 1.2 Hz, 1H), 3.81 (s, 3H), 3.67 (br s, 3H), 2.68 (s, 3H). ^{13}C NMR (101 MHz, $CDCl_3$) δ 153.2, 141.8, 125.1, 123.7, 109.0, 101.1, 55.7, 34.5. HRMS (ESI) calcd for $[C_8H_{12}N_2O + H]^+$: 153.1022. Found: 153.1026.

4-Methoxy-*N*1-methylbenzene-1,2-diamine (5l-ii)

^1H NMR (400 MHz, MeOD-d_4) δ 6.57 (dd, *J* = 8.5, 1.7 Hz, 1H), 6.37 (s, 1H), 6.28 (d, *J* = 8.6 Hz, 1H), 3.68 (s, 3H), 2.75 (s, 3H). ^{13}C NMR (101 MHz, MeOD-d_4) δ 153.8, 136.9, 131.4, 113.0, 103.2, 102.6, 54.5, 30.9. HRMS (ESI) calcd for $[C_8H_{12}N_2O + H]^+$: 153.1022. Found: 153.1028.

To confirm the *ortho* regioselectivity, the title compound **5l-ii** was converted to the corresponding benzimidazole. To achieve this trimethyl orthoformate (1 mL) and concentrated hydrochloric acid (2 drops) were added to 4-methoxy-*N*1-methylbenzene-1,2-diamine **5l-ii** (21.5 mg, 0.141 mmol) and the reaction was stirred at 80°C for 1 h. The reaction was concentrated under a stream of nitrogen and aqueous Na_2CO_3 was then added to the resulting residue. The organics were extracted with chloroform three times, dried ($MgSO_4$), filtered and concentrated *in vacuo*. Purification by silica gel chromatography (eluent:

EtOAc:MeOH, 98:2-96:4) gave the title compound **5l-iii** (19.6 mg, 0.121 mmol, 86%) as a yellow solid.

^{1}H NMR (400 MHz, $CDCl_3$) δ 7.30 – 7.20 (m, 2H), 6.97 (dd, *J* = 8.8, 2.3 Hz, 1H), 3.86 (s, 3H), 3.82 (s, 3H). ^{13}C NMR (101 MHz, $CDCl_3$) δ 156.3, 143.8, 143.4, 129.0, 113.4, 109.8, 101.9, 55.8, 31.3. HRMS (ESI) calcd for $[C_9H_{10}N_2O + H]^+$: 163.0866. Found: 163.0870.

4-Methoxy-*N*1,3-dimethylbenzene-1,2-diamine (5m-i) and 5-methoxy-1,4-dimethyl-1H-benzo[d]imidazole (5m-ii)

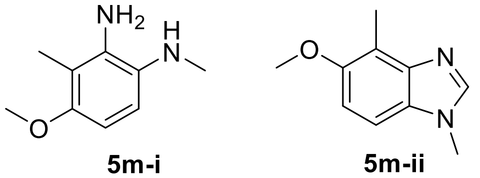

5m-i **5m-ii**

Tetrabutylammonium (3-methoxy-2-methylphenyl)sulfamate **1m** (229 mg, 0.499 mmol, 1 equiv), **3d** (248 mg, 0.749 mmol, 1.5 equiv), $FeBr_2$ (16.2 mg, 0.0751 mmol, 0.15 equiv) were subject to **GP-4**. Purification by silica gel chromatography (eluent: Pet. Ether:EtOAc, 95:5 – 65:35) gave the title compound **5l-i** (59.8 mg, 0.360 mmol, 72%, *o*:*p* >20:1) as a red oil.

^{1}H NMR (400 MHz, $CDCl_3$) δ 6.60 (d, *J* = 8.6 Hz, 1H), 6.41 (d, *J* = 8.3 Hz, 1H), 3.81 (s, 3H), 3.39 (br s, 3H), 2.86 (s, 3H), 2.13 (s, 3H). ^{13}C NMR (101 MHz, $CDCl_3$) δ 152.1, 135.5, 131.5, 111.4, 110.2, 101.4, 56.1, 32.1, 9.4. HRMS (ESI) calcd for $[C_9H_{14}N_2O + H]^+$: 167.1178. Found: 167.1179.

To confirm the *ortho* regiochemistry, the corresponding benzimidazole was synthesised. To achieve this, tetrabutylammonium (3-methoxy-2-methylphenyl)sulfamate **1m** (229 mg, 0.499 mmol, 1 equiv), **3d** (248 mg, 0.75 mmol, 1.5 equiv), $FeBr_2$ (16.2 mg, 0.075 mmol, 0.15 equiv) were subject to **GP-4**. To remove NBu_4OTf, the crude residue was then filtered through a silica plug (eluent Pet. Ether (40-60):EtOAc, 40:60) and the filtrate was concentrated *in vacuo*. Trimethyl orthoformate (1 mL) and concentrated hydrochloric acid (2 drops) were then added to the resulting residue and the reaction was stirred at 80°C for 2 h. The reaction was then concentrated under a stream of air and aqueous Na_2CO_3 was added to the resulting residue. The organics were then extracted with chloroform three time, dried ($MgSO_4$) filtered and concentrated *in vacuo*. The crude residue was then purified by silica gel chromatography

(eluent: EtOAc:MeOH 100:0 – 98:2) to give the title compound **5m-ii** (52.0 mg, 0.295 mmol, 59%) as a yellow solid.

^{1}H NMR (400 MHz, $CDCl_3$) δ 7.82 (s, 1H), 7.15 (d, *J* = 8.7 Hz, 1H), 7.00 (d, *J* = 8.7 Hz, 1H), 3.89 (s, 3H), 3.79 (s, 3H), 2.57 (s, 3H). ^{13}C NMR (101 MHz, $CDCl_3$) δ 153.2, 144.1, 143.6, 129.6, 117.8, 109.4, 106.0, 57.5, 31.1, 10.0. HRMS (ESI) calcd for $[C_{10}H_{12}N_2O + H]^+$: 177.1022. Found: 177.1018.

6-Difluoromethoxy-N1-methylbenzene-1,2-diamine (5n-i), 4-difluoromethoxy-N1-methylbenzene-1,2-diamine (5n-ii) and 5-(difluoromethoxy)-1-methyl-1H-benzo[d]imidazole (5n-iii)

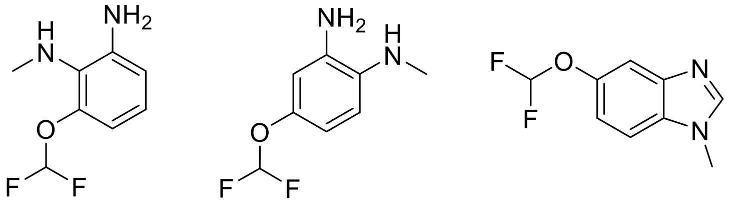

5n-i **5n-ii** **5n-iii**

Tetrabutylammonium (3-difluoromethoxyphenyl)sulfamate **1n** (240 mg, 0.499 mmol, 1 equiv), **3d** (248 mg, 0.749 mmol, 1.5 equiv), $FeBr_2$ (16.2 mg, 0.0751 mmol, 0.15 equiv) were subject to **GP-4**. Purification by silica gel chromatography (eluent: Pet. Ether:EtOAc, 95:5 – 70:30) gave the title compounds **5n-i** (12.2 mg, 0.0648 mmol, 13%, *o*:*p* >20:1) and **5n-ii** (38.4 mg, 0.204 mmol, 41%, *o*:*p* >20:1) with a total yield of 54% and a regioisomer ratio of 3.1:1.

6-Difluoromethoxy-*N*1-methylbenzene-1,2-diamine (5n-i)

^{1}H NMR (400 MHz, $CDCl_3$) δ 6.84 (t, *J* = 8.1 Hz, 1H), 6.57 (dd, *J* = 8.1, 1.3 Hz, 1H), 6.50 (dd, *J* = 8.1, 1.1 Hz, 1H), 6.50 (t, *J* = 74.4 Hz, 1H), 3.75 (br s, 3H), 2.71 (s, 3H). ^{13}C NMR (101 MHz, $CDCl_3$) δ 145.3 (t, *J* = 2.5 Hz), 142.7, 127.9, 123.8, 116.7 (t, *J* = 258.8 Hz), 112.8, 108.5, 34.3. ^{19}F NMR (376 MHz, $CDCl_3$) δ -80.14. HRMS (ESI) calcd for $[C_8H_{10}F_2N_2O + H]^+$: 189.0834. Found: 189.0835

4-Difluoromethoxy-*N*1-methylbenzene-1,2-diamine (5n-ii)

^{1}H NMR (400 MHz, $CDCl_3$) δ 6.60 (s, 2H), 6.53 (s, 1H), 6.39 (t, *J* = 75.1 Hz, 1H), 3.48 (br s, 3H), 2.85 (s, 3H). ^{13}C NMR (101 MHz, $CDCl_3$) δ 144.1 (t, *J* = 2.9 Hz), 135.8, 135.8, 116.6 (t, *J* = 258.1

Hz), 111.8, 111.0, 108.3, 31.3. ^{19}F NMR (376 MHz, $CDCl_3$) δ -79.81. HRMS (ESI) calcd for $[C_8H_{10}F_2N_2O + H]^+$: 189.0834. Found: 189.0836.

To confirm the *ortho* regioselectivity, the title compound **5n-ii** was converted to the corresponding benzimidazole. To achieve this trimethyl orthoformate (1 mL) and concentrated hydrochloric acid (2 drops) were added to 4-difluoromethoxy-*N*1-methylbenzene-1,2-diamine **5m-ii** (38.4 mg, 0.204 mmol) and the reaction was stirred at 80°C for 1 h. The reaction was concentrated under a stream of nitrogen and aqueous Na_2CO_3 was then added to the resulting residue. The organics were extracted with chloroform three times, dried ($MgSO_4$), filtered and concentrated *in vacuo*. Purification by silica gel chromatography (eluent: EtOAc:MeOH, 99:1) gave the title compound **5n-iii** (27.6 mg, 0.139 mmol, 70%) as a brown oil.

^{1}H NMR (400 MHz, $CDCl_3$) δ 7.92 (s, 1H), 7.58 (d, *J* = 2.2 Hz, 1H), 7.36 (d, *J* = 8.7 Hz, 1H), 7.16 (dd, *J* = 8.7, 2.2 Hz, 1H), 6.54 (t, *J* = 74.4 Hz, 1H), 3.86 (s, 3H). ^{13}C NMR (101 MHz, $CDCl_3$) δ 146.9 (t, *J* = 3.0 Hz), 144.9, 144.0, 132.3, 116.5, 116.4 (t, *J* = 268.1 Hz), 111.3, 110.0, 31.3. ^{19}F NMR (376 MHz, $CDCl_3$) δ -81.26. HRMS (ESI) calcd for $[C_9H_8F_2N_2O + H]^+$: 199.0677. Found: 199.0677.

6-Bromo-*N*1,3-dimethylbenzene-1,2-diamine (5o)

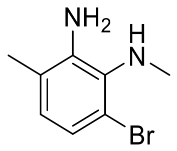

Tetrabutylammonium (5-bromo-2-methylphenyl)sulfamate **1o** (254 mg, 0.500 mmol, 1 equiv), **3d** (248 mg, 0.749 mmol, 1.5 equiv), $FeBr_2$ (16.2 mg, 0.0751 mmol, 0.15 equiv) were subject to **GP-4**. Purification by silica gel chromatography (eluent: Pet. Ether:EtOAc, 95:5 – 80:20 then Pet. Ether (40-60):CH_2Cl_2 50:50– 0:100) gave the title compound (39.0 mg, 0.181 mmol, 36%, *o*:*p* >20:1) as a red oil.

^{1}H NMR (400 MHz, $CDCl_3$) δ 6.86 (d, *J* = 8.1 Hz, 1H), 6.69 (d, *J* = 8.1 Hz, 1H), 3.85 (br s, 3H), 2.68 (s, 3H), 2.13 (s, 3H). ^{13}C NMR (101 MHz, $CDCl_3$) δ 141.05, 133.20, 126.66, 121.96, 120.70, 117.20, 33.89, 17.35. HRMS (ESI) calcd for $[C_8H_{11}N_2Br + H]^+$: 215.0178. Found: 215.0179.

4-Chloro-*N*1,3-dimethylbenzene-1,2-diamine (5p-i) and 5-chloro-1,4-dimethyl-1H-benzo[d]imidazole (5p-ii)

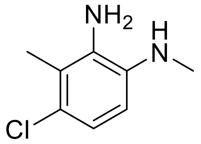
5p-i

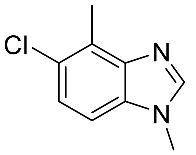
5p-ii

Tetrabutylammonium (3-chloro-2-methylphenyl)sulfamate **1o** (232 mg, 0.500 mmol, 1 equiv), **3d** (248 mg, 0.749 mmol, 1.5 equiv), $FeBr_2$ (16.2 mg, 0.0751 mmol, 0.15 equiv) were subject to **GP-4**. Purification by silica gel chromatography (eluent: Pet. Ether:EtOAc, 95:5 – 75:25) gave the title compound (40.1 mg, 0.235 mmol, 47%, *o*:*p* >20:1) as a red solid.

Note: NMR analysis of the crude reaction mixture suggested an *o*:*p* ratio of 18:1. However, the minor isomer was unable to be isolated by column chromatography to confirm this.

^{1}H NMR (400 MHz, $CDCl_3$) δ 6.86 (d, *J* = 8.5 Hz, 1H), 6.52 (d, *J* = 8.5 Hz, 1H), 3.41 (br s, 3H), 2.85 (s, 3H), 2.27 (s, 3H). ^{13}C NMR (101 MHz, $CDCl_3$) δ 136.2, 134.3, 125.0, 120.2, 119.8, 110.2, 31.4, 14.1. HRMS (ESI) calcd for $[C_8H_{11}ClN_2 + H]^+$: 171.0684. Found: 171.0681.

To confirm the *ortho* regioselectivity the title compound **5p-i** was converted to the corresponding benzimidazole. To achieve this trimethyl orthoformate (1 mL) and concentrated hydrochloric acid (2 drops) were added to 4-chloro-*N*1,3-dimethylbenzene-1,2-diamine **5o-i** (40.1 mg, 0.235 mmol) and the reaction was stirred at 80°C for 1 h. The reaction was concentrated under a stream of nitrogen and aqueous Na_2CO_3 was then added to the resulting residue. The organics were extracted with chloroform three times, dried ($MgSO_4$), filtered and concentrated *in vacuo*. Purification by silica gel chromatography (eluent: EtOAc:MeOH, 99:1) gave the title compound **5p-ii** (25.2 mg, 0.140 mmol, 58%) as a white solid.

^{1}H NMR (400 MHz, $CDCl_3$) δ 7.85 (s, 1H), 7.29 (d, *J* = 8.5 Hz, 1H), 7.13 (d, *J* = 8.6 Hz, 1H), 3.81 (s, 3H), 2.69 (s, 3H). ^{13}C NMR (101 MHz, $CDCl_3$) δ 143.8, 143.6, 132.7, 128.2, 127.5, 123.9, 107.4, 31.2, 14.0. HRMS (ESI) calcd for $[C_9H_9ClN_2 + H]^+$: 181.0527. Found: 181.0526.

4-Fluoro-*N*1,3-dimethylbenzene-1,2-diamine (5q)

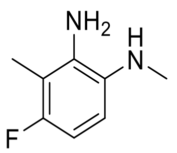

Tetrabutylammonium (3-fluoro-2-methylphenyl)sulfamate **1q** (223 mg, 0.499 mmol, 1 equiv), **3d** (248 mg, 0.749 mmol, 1.5 equiv), $FeBr_2$ (16.2 mg, 0.0751 mmol, 0.15 equiv) were subject to **GP-4**. Purification by silica gel chromatography (eluent: Pet. Ether:EtOAc, 90:10 – 60:40) gave the title compound (44.2 mg, 0.287 mmol, 57%, *o*:*p* >20:1) as a red solid.

^{1}H NMR (400 MHz, MeOD-d_4) δ 6.45 (dd, *J* = 8.7, 5.4 Hz), 6.37 (t, *J* = 9.0 Hz, 1H), 2.76 (s, 3H), 2.06 (d, *J* = 1.7 Hz, 3H).^{13}C NMR (101 MHz, MeOD-d_4) δ 155.7 (d, *J* = 231.5 Hz), 135.0 (d, *J* = 6.2 Hz), 132.9 (d, *J* = 2.0 Hz), 109.4 (d, *J* = 9.9 Hz), 109.0 (d, *J* = 20.1 Hz), 103.3 (d, *J* = 24.1 Hz), 30.6, 7.5 (d, *J* = 5.8 Hz). ^{19}F NMR (376 MHz, MeOD-d_4) δ -131.14. HRMS (ESI) calcd for $[C_8H_{11}N_2F + H]^+$: 155.0979. Found: 155.0978.

4-Fluoro-*N*1-methylbenzene-1,2-diamine (5r)

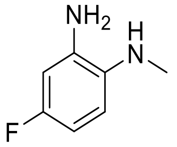

Tetrabutylammonium (3-fluorophenyl)sulfamate **1r** (216 mg, 0.499 mmol, 1 equiv), **3d** (248 mg, 0.749 mmol, 1.5 equiv), $FeBr_2$ (16.2 mg, 0.0751 mmol, 0.15 equiv) were subject to **GP-4**. Purification by silica gel chromatography (eluent: Pet. Ether:EtOAc, 90:10 – 70:30) gave the title compound (29.5 mg, 0.210 mmol, 42%, *o*:*p* >20:1) as a red oil.

Note: It's suspected that there was a 4.5:1 regioisomer ratio of 4-fluoro-*N*1-methylbenzene-1,2-diamine and 6-fluoro-*N*1-methylbenzene-1,2-diamine in the crude NMR however only the major regioisomer could be isolated after column chromatography.

^{1}H NMR (400 MHz, $CDCl_3$) δ 6.58 (dd, *J* = 8.6, 5.4 Hz, 1H), 6.51 (td, *J* = 8.7, 2.8 Hz, 1H), 6.46 (dd, *J* = 9.7, 2.8 Hz, 1H), 3.37 (br s, 3H), 2.83 (s, 3H). ^{13}C NMR (101 MHz, $CDCl_3$) δ 157.1 (d, *J* = 235.3 Hz), 136.3 (d, *J* = 10.1 Hz), 134.0 (d, *J* = 2.0 Hz), 112.2 (d, *J* = 9.3 Hz), 105.4 (d, *J* = 21.7 Hz), 103.1 (d, *J* = 25.7 Hz), 31.6. ^{19}F NMR (376 MHz, $CDCl_3$) δ -125.35. HRMS (ESI) calcd for $[C_7H_9N_2F + H]^+$: 141.0823. Found: 141.0826.

3-Bromo-*N*1,4-dimethylbenzene-1,2-diamine (5s)

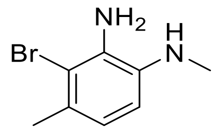

Tetrabutylammonium (2-Bromo-3-methylphenyl)sulfamate **1s** (254 mg, 0.500 mmol, 1 equiv), **3d** (248 mg, 0.749 mmol, 1.5 equiv), $FeBr_2$ (16.2 mg, 0.0751 mmol, 0.15 equiv) were subject to **GP-4**. Purification by silica gel chromatography was performed twice (eluent: Pet. Ether:EtOAc, 95:5 – 65:35 then Pet. Ether:CH_2Cl_2 40:60– 0:100) to give the title compound (43.7 mg, 0.203 mmol, 41%, *o*:*p* >20:1) as a yellow oil.

Note: NMR analysis of the crude reaction mixture suggested an *o*:*p* ratio of 17:1. However, the minor isomer was unable to be isolated by column chromatography to confirm this.

^{1}H NMR (700 MHz, $CDCl_3$) δ 6.74 (d, *J* = 7.9 Hz, 1H), 6.58 (d, *J* = 8.0 Hz, 1H), 3.69 (br s, 3H), 2.87 (s, 3H), 2.37 (s, 3H). ^{13}C NMR (176 MHz, $CDCl_3$) δ 136.7, 133.2, 128.0, 120.8, 113.7, 110.1, 31.5, 22.9. $[C_8H_{11}N_2Br + H]^+$: 215.0178. Found: 215.0177.

3-Allyl-*N*1-methylbenzene-1,2-diamine (5t)

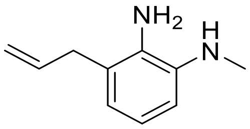

Tetrabutylammonium (2-allylphenyl)sulfamate **1t** (228 mg, 0.501 mmol, 1 equiv), **3d** (248 mg, 0.749 mmol, 1.5 equiv), $FeBr_2$ (16.2 mg, 0.0751 mmol, 0.15 equiv) were subject to **GP-4**. Purification by silica gel chromatography (eluent: Pet. Ether:EtOAc, 95:5 – 75:25) gave the title compound (54.4 mg, 0.335 mmol, 67%, *o*:*p* >20:1) as a yellow liquid.

^{1}H NMR (400 MHz, $CDCl_3$) δ 6.83 (t, *J* = 7.7 Hz, 1H), 6.69 – 6.56 (m, 2H), 5.97 (ddt, *J* = 16.6, 10.4, 6.2 Hz, 1H), 5.17 – 5.04 (m, 2H), 3.50 (br s, 3H), 3.36 (dt, *J* = 6.2, 1.7 Hz, 2H), 2.88 (s, 3H). ^{13}C NMR (101 MHz, $CDCl_3$) δ 138.3, 136.1, 133.1, 124.5, 120.6, 119.9, 115.9, 110.2, 36.7, 31.4. HRMS (ESI) calcd for $[C_{10}H_{13}N_2 + H]^+$: 162.1151. Found: 162.1146.

3-Phenyl-*N*1-methylbenzene-1,2-diamine (5u)

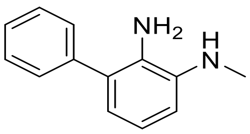

Tetrabutylammonium (2-biphenyl)sulfamate **1u** (246 mg, 0.501 mmol, 1 equiv), **3d** (248 mg, 0.749 mmol, 1.5 equiv), $FeBr_2$ (16.2 mg, 0.0751 mmol, 0.15 equiv) were subject to **GP-4**. Purification by silica gel chromatography was performed twice (eluent: Pet. Ether:CH_2Cl_2, 40:60 – 0:100 then Pet. Ether:EtOAc 95:5– 70:30) gave the title compound (59.9 mg, 0.302 mmol, 60%, *o*:*p* >20:1) as a red oil.

^{1}H NMR (400 MHz, $CDCl_3$) δ 7.54 – 7.44 (m, 4H), 7.43 – 7.32 (m, 1H), 6.95 (t, *J* = 7.8 Hz, 1H), 6.80 – 6.70 (m, 2H), 3.55 (br s, 3H), 2.94 (s, 3H). ^{13}C NMR (101 MHz, $CDCl_3$) δ 139.9, 138.6, 131.7, 129.3, 128.9, 128.8, 127.1, 120.4, 119.9, 110.3, 31.3. HRMS (ESI) calcd for $[C_{13}H_{14}N_2 + H]^+$: 199.1230. Found: 199.1221.

3-(*p*-Chlorophenyl)-*N*1-methylbenzene-1,2-diamine (5v)

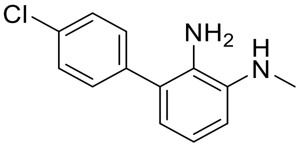

Tetrabutylammonium (2-(4-chlorophenyl)phenyl)sulfamate **1v** (262 mg, 0.499 mmol, 1 equiv), **3d** (248 mg, 0.749 mmol, 1.5 equiv), $FeBr_2$ (16.2 mg, 0.0751 mmol, 0.15 equiv) were subject to **GP-4**. Purification by silica gel chromatography (eluent: Pet. Ether:CH_2Cl_2 40:60–0:100) gave the title compound (50.2 mg, 0.216 mmol, 43%, *o*:*p* >20:1) as a red solid.

^{1}H NMR (400 MHz, $CDCl_3$) δ 7.43 (d, *J* = 8.4 Hz, 2H), 7.38 (d, *J* = 8.5 Hz, 2H), 6.91 (t, *J* = 7.8 Hz, 1H), 6.73 (dd, *J* = 8.0, 1.3 Hz, 1H), 6.69 (dd, *J* = 7.7, 1.3 Hz, 1H), 3.51 (br s, 3H), 2.92 (s, 3H). ^{13}C NMR (101 MHz, $CDCl_3$) δ 138.4, 138.2, 133.1, 131.7, 130.6, 129.0, 127.5, 120.3, 120.0, 110.7, 31.3. HRMS (ESI) calcd for $[C_{13}H_{13}ClN_2 + H]^+$: 233.0840. Found: 233.0838.

3-(*p*-Tolyl)-*N*1-methylbenzene-1,2-diamine (5w)

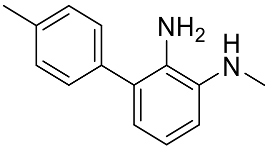

Tetrabutylammonium (2-(*p*-tolyl)phenyl)sulfamate **1w** (152 mg, 0.301 mmol, 1 equiv), **3d** (149 mg, 0.450 mmol, 1.5 equiv), $FeBr_2$ (9.7 mg, 0.0450 mmol, 0.15 equiv) were subject to **GP-4**. Purification by silica gel chromatography was performed twice (eluent: Pet. Ether:EtOAc, 95:5 – 75:25 then Pet. Ether:CH_2Cl_2 40:60– 0:100) to give the title compound (31.9 mg, 0.150 mmol, 50%, *o*:*p* >20:1) as a yellow solid.

^{1}H NMR (400 MHz, $CDCl_3$) δ 7.35 (d, *J* = 7.7 Hz, 2H), 7.29 (d, *J* = 7.9 Hz, 2H), 6.92 (t, *J* = 7.7 Hz, 1H), 6.85 – 6.71 (m, 2H), 3.75 (s, 3H), 2.96 (br s, 3H), 2.44 (s, 3H). ^{13}C NMR (101 MHz, $CDCl_3$) δ 137.4, 136.9, 136.7, 132.2, 129.5, 129.1, 129.1, 121.2, 119.8, 111.1, 31.6, 21.2. HRMS (ESI) calcd for $[C_{14}H_{16}N_2 + H]^+$: 213.1386. Found: 213.1387.

_N_1-Ethyl-3-methylbenzene-1,2-diamine (5x)

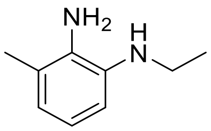

Tetrabutylammonium *o*-tolylsulfamate **1b** (214 mg, 0.499 mmol, 1 equiv), **3e** (259 mg, 0.750 mmol, 1 equiv), $FeBr_2$ (16.2 mg, 0.0751 mmol, 0.15 equiv) were subject to **GP-4**. Purification by silica gel chromatography (eluent: Pet. Ether:EtOAc, 95:5 – 70:30) gave the title compound (29.4 mg, 0.196 mmol, 39%, *o*:*p* >20:1) as a red solid.

Note: NMR analysis of the crude reaction mixture suggested an *o*:*p* ratio of 14:1. However, the minor isomer was unable to be isolated by column chromatography to confirm this.

^{1}H NMR (400 MHz, $CDCl_3$) δ 6.74 (t, *J* = 7.7 Hz, 1H), 6.66 – 6.59 (m, 2H), 3.34 (br s, 3H), 3.16 (q, *J* = 7.1 Hz, 2H), 2.21 (s, 3H), 1.31 (t, *J* = 7.1 Hz, 3H). ^{13}C NMR (101 MHz, $CDCl_3$) δ 136.8, 132.9, 122.9, 121.0, 119.5, 110.6, 39.2, 17.6, 15.0. HRMS (ESI) calcd for $[C_9H_{14}N_2 + H]^+$: 151.1230. Found: 151.1229.

4,6-Dimethoxy-_N_1-propylbenzene-1,2-diamine (5y)

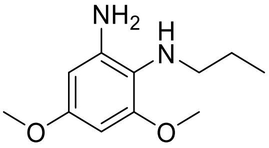

Tetrabutylammonium (3,5-dimethoxyphenyl)sulfamate **1x** (188 mg, 0.396 mmol, 1 equiv), **3f** (216 mg, 0.601 mmol, 1.5 equiv), $FeBr_2$ (13.0 mg, 0.0603 mmol, 0.15 equiv) were subject to **GP-4**. Purification by silica gel chromatography (eluent: Pet. Ether:EtOAc, 90:10 – 50:50) gave the title compound (52.1 mg, 0.248 mmol, 62%, *o*:*p* >20:1) as a colourless oil.

^{1}H NMR (400 MHz, $CDCl_3$) δ 5.95 – 5.91 (m, 2H), 4.31 (br s, 3H), 3.79 (s, 3H), 3.73 (s, 3H), 2.96 – 2.78 (m, 2H), 1.61 (h, *J* = 7.4 Hz, 2H), 0.96 (t, *J* = 7.4 Hz, 3H). ^{13}C NMR (101 MHz, $CDCl_3$) δ 157.7, 154.6, 142.9, 114.6, 93.3, 89.2, 55.7, 55.3, 50.5, 22.9, 11.5. HRMS (ESI) calcd for $[C_{11}H_{18}N_2O_2 + H]^+$: 211.1441. Found: 211.1439.

3-((2-Amino-4-methoxy-3-methylphenyl)amino)propanenitrile (5z-i) and 3-(5-methoxy-4-methyl-1H-benzo[d]imidazol-1-yl)propanenitrile (5z-ii)

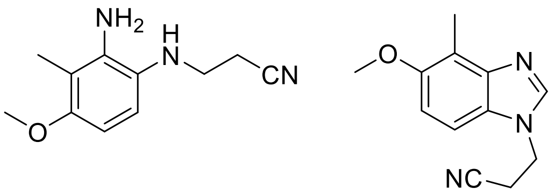

5z-i **5z-ii**

Tetrabutylammonium (3-methoxy-2-methylphenyl)sulfamate **1m** (229 mg, 0.499 mmol, 1 equiv), **3g** (277 mg, 0.748 mmol, 1.5 equiv), $FeBr_2$ (16.2 mg, 0.0751 mmol, 0.15 equiv) were subject to **GP-4**. Purification by silica gel chromatography (eluent: CH_2Cl_2:EtOAc, 100:0 – 85:15) gave the title compound **5y-i** (42.8 mg, 0.208 mmol, 42%, *o*:*p* >20:1) as an orange solid.

^{1}H NMR (400 MHz, $CDCl_3$) δ 6.64 (d, *J* = 8.6 Hz, 1H), 6.31 (d, *J* = 8.5 Hz, 1H), 3.78 (s, 3H), 3.67 (br s, 3H), 3.37 (t, *J* = 6.5 Hz, 2H), 2.60 (t, *J* = 6.5 Hz, 2H), 2.08 (s, 3H). ^{13}C NMR (101 MHz, $CDCl_3$) δ 153.9, 138.3, 126.6, 118.6, 115.4, 111.7, 100.8, 55.8, 42.1, 18.3, 9.5. HRMS (ESI) calcd for $[C_{11}H_{15}N_3O + H]^+$: 206.1288. Found: 206.1285.

To confirm the *ortho* regioselectivity the title compound **5z-i** was converted to the corresponding benzimidazole. To achieve this trimethyl orthoformate (1 mL) and concentrated hydrochloric acid (2 drops) were added to 3-((2-amino-4-methoxy-3-methylphenyl)amino)propanenitrile **5z-i** (42.8 mg, 0.208 mmol) and the reaction was stirred at 80°C for 1 h. The reaction was concentrated under a stream of nitrogen and aqueous Na_2CO_3 was then added to the resulting residue. The organics were extracted with chloroform

three times, dried ($MgSO_4$), filtered and concentrated *in vacuo*. Purification by silica gel chromatography (eluent: EtOAc:MeOH, 98:2) gave the title compound **5z-ii** (25.3 mg, 0.118 mmol, 57%) as a white solid.

^{1}H NMR (400 MHz, $CDCl_3$) δ 8.06 (s, 1H), 7.15 (d, *J* = 8.7 Hz, 1H), 7.00 (d, *J* = 8.7 Hz, 1H), 4.47 (t, *J* = 6.7 Hz, 2H), 3.88 (s, 3H), 2.89 (t, *J* = 6.7 Hz, 2H), 2.54 (s, 3H). ^{13}C NMR (101 MHz, $CDCl_3$) δ 153.8, 143.7, 142.6, 127.6, 118.3, 116.5, 109.7, 105.7, 57.2, 40.8, 19.2, 10.1. [$C_{12}H_{13}N_3O$ + H]$^+$: 216.1131. Found: 216.1135.

1-Hexyl-5-methoxy-4-methyl-1H-benzo[d]imidazole (5aa)

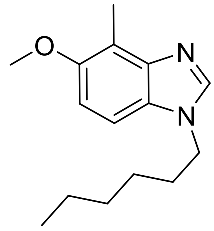

Tetrabutylammonium (3-methoxy-2-methylphenyl)sulfamate **1m** (115 mg, 0.251 mmol, 1 equiv), **3h** (156 mg, 0.376 mmol, 1.5 equiv), $FeBr_2$ (8.1 mg, 0.0376 mmol, 0.15 equiv) were subject to **GP-4**. For ease of purification, the crude product was converted to the corresponding benzimidazole. To the crude residue, trimethylorthoformate (0.5 mL) and concentrated hydrochloric acid (2 drops) were added and the reaction was stirred at 80°C for 1 h. The reaction was concentrated under a stream of nitrogen and aqueous Na_2CO_3 was then added to the resulting residue. The organics were extracted with chloroform three times, dried ($MgSO_4$), filtered and concentrated *in vacuo* Purification by silica gel chromatography (eluent: Pet. Ether:EtOAc, 70:30 – 40:60) gave the title compound (41.5 mg, 0.159 mmol, 64%) as a yellow oil.

^{1}H NMR (400 MHz, $CDCl_3$) δ 7.91 (s, 1H), 7.15 (d, *J* = 8.8 Hz, 1H), 6.97 (d, *J* = 8.8 Hz, 1H), 4.11 (t, *J* = 7.1 Hz, 2H), 3.88 (s, 3H), 2.56 (s, 3H), 1.85 (p, *J* = 7.2 Hz, 2H), 1.36 – 1.22 (m, 6H), 0.94 – 0.77 (m, 3H). ^{13}C NMR (101 MHz, $CDCl_3$) δ 153.2, 143.7, 142.9, 128.6, 117.7, 109.3, 106.4, 57.4, 45.2, 31.2, 29.7, 26.4, 22.5, 13.9, 10.0. HRMS (ESI) calcd for [$C_{15}H_{22}N_2O$ + H]$^+$: 247.1805. Found: 247.1814.

9. NH_2 Transfer Products

Benzene-1,2-diamine (4a)

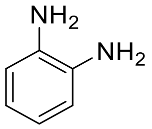

Tetrabutylammonium phenylsulfamate **1a** (207 mg, 0.50 mmol, 1 equiv), **2d** (238 mg, 0.750 mmol, 1.5 equiv), $FeBr_2$ (16.2 mg, 0.0751 mmol, 0.15 equiv) were subject to **GP-4**. Purification by silica gel chromatography (eluent: Pet. Ether:EtOAc, 80:20 – 50:50) gave the title compound (30.8 mg, 0.285 mmol, 57%, *o*:*p* >20:1) as a yellow solid.

^1H NMR (400 MHz, $CDCl_3$) δ 6.80 – 6.61 (m, 4H), 3.44 (br s, 4H). ^{13}C NMR (101 MHz, $CDCl_3$) δ 134.7, 120.3, 116.7.

Data match literature values.[17]

3-Methylbenzene-1,2-diamine (4b)

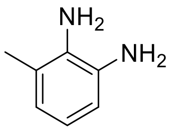

Tetrabutylammonium *o*-tolylsulfamate **1b** (214 mg, 0.499 mmol, 1 equiv), **2c** (249 mg, 0.750 mmol, 1 equiv), $FeBr_2$ (16.2 mg, 0.0751 mmol, 0.15 equiv) were subject to **GP-4**. Purification by silica gel chromatography (eluent: Pet. Ether:EtOAc, 80:20 – 50:50) gave the title compound (28.4 mg, 0.232 mmol, 47%, *o*:*p* >20:1) as a red oil.

^1H NMR (400 MHz, $CDCl_3$) δ 6.67 – 6.61 (m, 3H), 3.38 (br s, 4H), 2.20 (s, 3H). ^{13}C NMR (101 MHz, $CDCl_3$) δ 133.9, 133.4, 123.3, 122.1, 119.1, 115.1, 17.4.

Data match literature values.[18]

3-Isopropylbenzene-1,2-diamine (4c-i) and 4-isopropyl-1H-benzo[d]imidazole (4c-ii)

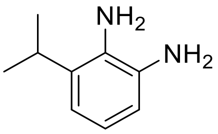

4c-i

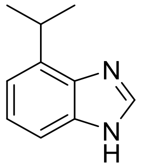

4c-ii

Tetrabutylammonium 2-isopropyl(phenyl)sulfamate **1c** (228 mg, 0.499 mmol, 1 equiv), **2a** (317 mg, 0.749 mmol, 1.5 equiv), $FeBr_2$ (16.2 mg, 0.0751 mmol, 0.15 equiv) were subject to **GP-4**. Purification by silica gel chromatography (eluent: Pet. Ether:EtOAc, 85:15 – 50:50) gave the title compound **4c-i** (34.2 mg, 0.228mmol, 46%, *o*:*p* >20:1) as a red oil.

^{1}H NMR (400 MHz, MeOD-d_4) δ 6.69 – 6.54 (m, 3H), 3.01 (hept, *J* = 6.8 Hz, 1H), 1.21 (d, *J* = 6.8 Hz, 6H). ^{13}C NMR (101 MHz, MeOD-d_4) δ 133.8, 131.6, 118.9, 116.3, 114.5, 27.2, 21.7. HRMS (ESI) calcd for $[C_9H_{14}N_2 + H]^+$: 151.1230. Found: 151.1224.

Note: Only 5 aromatic carbons could be seen. Therefore, to confirm the isolation of the title compound, it was converted into the corresponding benzimidazole.

Trimethyl orthoformate (1 mL) and concentrated hydrochloric acid (2 drops) were added to 3-isopropylbenzene-1,2-diamine **4c-i** (34.2 mg, 0.228 mmol) and the reaction was stirred at 80°C for 1 h. The reaction was concentrated under a stream of nitrogen and aqueous Na_2CO_3 was then added to the resulting residue. The organics were extracted with chloroform three times, dried ($MgSO_4$), filtered and concentrated *in vacuo*. Purification by silica gel chromatography (eluent: EtOAc) gave the title compound **4c-ii** (13.0 mg, 0.0811 mmol, 35%) as a yellow oil.

^{1}H NMR (400 MHz, $CDCl_3$) δ 8.90 (s, 1H), 8.81 (br s, 1H), 7.58 (d, *J* = 8.1 Hz, 1H), 7.33 (t, *J* = 7.9 Hz, 1H), 7.25 (d, *J* = 7.6 Hz, 1H), 3.63 (hept, *J* = 7.0 Hz, 1H), 1.40 (d, *J* = 6.9 Hz, 6H). ^{13}C NMR (101 MHz, $CDCl_3$) δ 139.7, 136.5, 134.5, 133.3, 124.6, 120.3, 112.4, 29.6, 22.9. $[C_{10}H_{12}N_2 + H]^+$: 161.1073. Found: 161.1081.

Note: ^{1}H NMR analysis showed a 3:1 mixture of product tautomers. Only peaks for the major tautomer are reported.

3-Chlorobenzene-1,2-diamine (4d-i) and 4-chlorobenzene-1,2-diamine (4d-ii)

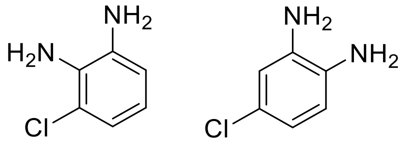

4d-i **4d-ii**

Tetrabutylammonium (3-chlorophenyl)sulfamate **1y** (225 mg, 0.501 mmol, 1 equiv), **2a** (317 mg, 0.749 mmol, 1.5 equiv), (16.2 mg, 0.0751 mmol, 0.15 equiv) were subject to **GP-4**. Purification by silica gel chromatography (eluent: Pet. Ether:EtOAc, 80:20 – 50:50) gave the title compound **4d-i** (16.3 mg, 0.114 mmol, 23%, *o*:*p* >20:1) and **4d-ii** (21.3 mg, 0.149 mmol, 30%, *o*:*p* >20:1) with a total yield of 53% and a regioisomer ratio of 1.3:1.

3-Chlorobenzene-1,2-diamine (4d-i)

^{1}H NMR (400 MHz, $CDCl_3$) δ 6.82 (dd, *J* = 6.2, 3.2 Hz, 1H), 6.67 – 6.57 (m, 2H), 3.55 (br s, 4H). ^{13}C NMR (101 MHz, $CDCl_3$) δ 135.5, 132.0, 120.9, 120.4, 119.6, 114.9.

Data match literature values.[19]

4-Chlorobenzene-1,2-diamine (4d-ii)

^{1}H NMR (400 MHz, $CDCl_3$) δ 6.73 – 6.66 (m, 2H), 6.63 (d, *J* = 8.0 Hz, 1H), 3.36 (br s, 4H). ^{13}C NMR (101 MHz, $CDCl_3$) δ 136.1, 133.0, 124.8, 119.6, 117.6, 116.3.

Data match literature values. [20]

3-Fluorobenzene-1,2-diamine (4e-i) and 4-fluorobenzene-1,2-diamine (4e-ii)

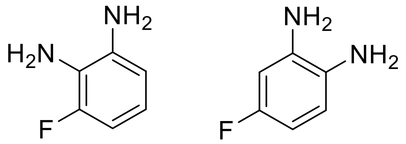

4e-i **4e-ii**

Tetrabutylammonium (3-fluorophenyl)sulfamate **1r** (216 mg, 0.499 mmol, 1 equiv), **2c** (249 mg, 0.750 mmol, 1.5 equiv), $FeBr_2$ (16.2 mg, 0.0751 mmol, 0.15 equiv) were subject to **GP-4**. Purification by silica gel chromatography (eluent: Pet. Ether:EtOAc, 85:15 – 30:70) gave the title compound **4e-i** (13.0 mg, 0.103 mmol, 21%, *o*:*p* >20:1) and **4e-ii** (20.4 mg, 0.162 mmol, 32%, *o*:*p* >20:1) with a total yield of 53% and a regioisomer ratio of 1.6:1.

3-Fluorobenzene-1,2-diamine (4e-i)

^{1}H NMR (400 MHz, $CDCl_3$) δ 6.63 (td, *J* = 8.0, 6.0 Hz, 1H), 6.59 – 6.52 (m, 1H), 6.49 (dt, *J* = 7.8, 1.3 Hz, 1H), 3.20 (br s, 4H). ^{13}C NMR (101 MHz, $CDCl_3$) δ 153.1 (d, *J* = 235.7 Hz), 137.0 (d, *J* = 5.5 Hz), 122.4 (d, *J* = 15.7 Hz), 119.4 (d, *J* = 9.3 Hz), 111.8 (d, *J* = 2.4 Hz), 106.4 (d, *J* = 20.0 Hz). ^{19}F NMR (376 MHz, $CDCl_3$) δ -134.52.

Data match literature values.[21]

4-Fluorobenzene-1,2-diamine (4e-ii)

^{1}H NMR (400 MHz, $CDCl_3$) δ 6.62 (dd, *J* = 8.4, 5.5 Hz, 1H), 6.44 (dd, *J* = 9.8, 2.7 Hz, 1H), 6.38 (td, *J* = 8.5, 2.8 Hz, 1H), 3.24 (br s, 4H). ^{13}C NMR (101 MHz, $CDCl_3$) δ 157.8 (d, *J* = 236.2 Hz), 136.8 (d, *J* = 10.2 Hz), 129.7 (d, *J* = 2.3 Hz), 117.7 (d, *J* = 9.3 Hz), 105.3 (d, *J* = 22.2 Hz), 103.2 (d, *J* = 25.5 Hz). ^{19}F NMR (376 MHz, $CDCl_3$) δ -123.63.

Data match literature values.[20]

3-Bromobenzene-1,2-diamine (4f-i) and 4-Bromobenzene-1,2-diamine (4f-ii)

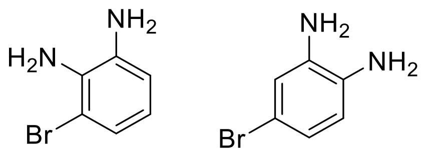

Tetrabutylammonium (3-bromophenyl)sulfamate **1z** (247 mg, 0.501 mmol, 1 equiv), **2b** (215 mg, 0.749 mmol, 1.5 equiv), (16.2 mg, 0.0751 mmol, 0.15 equiv) were subject to **GP-4**. Purification by silica gel chromatography (eluent: Pet. Ether:EtOAc, 85:15 – 50:50) gave the title compound **4f-i** (14.1 mg, 0.0754 mmol, 15%, *o*:*p* >20:1) and **4f-ii** (29.9 mg, 0.160 mmol, 32%, *o*:*p* >20:1) with a total yield of 47% and a regioisomer ratio of 2.1:1.

3-Bromobenzene-1,2-diamine (4f-i)

^{1}H NMR (400 MHz, $CDCl_3$) δ 7.00 (d, *J* = 8.0 Hz, 1H) 6.67 (d, *J* = 7.6 Hz, 1H), 6.59 (t, *J* = 7.8 Hz, 1H), 3.66 (br s, 4H). ^{13}C NMR (101 MHz, $CDCl_3$) δ 135.4, 133.3, 123.6, 120.3, 115.6, 111.2. HRMS (ESI) calcd for $[C_6H_7BrN_2 + H]^+$: 186.9865. Found: 186.9863.

4-Bromobenzene-1,2-diamine (4f-ii)

^{1}H NMR (400 MHz, $CDCl_3$) δ 6.85 (s, 1H), 6.82 (d, *J* = 8.3 Hz, 1H), 6.59 (d, *J* = 7.5 Hz, 1H), 3.26 (br s, 4H). ^{13}C NMR (101 MHz, $CDCl_3$) δ 136.4, 133.6, 122.6, 119.1, 117.9, 112.0.

Data match literature values.[22]

3,5-Difluorobenzene-1,2-diamine (4g)

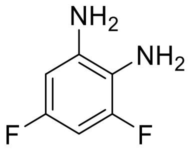

Tetrabutylammonium (3,5-difluorophenyl)sulfamate **1aa** (226 mg, 0.502 mmol, 1 equiv), **2c** (249 mg, 0.750 mmol, 1.5 equiv), $FeBr_2$ (16.2 mg, 0.0751 mmol, 0.15 equiv) were subject to **GP-4**. Purification by silica gel chromatography (eluent: Pet. Ether:EtOAc, 80:20 – 50:50) gave the title compound (29.1 mg, 0.202 mmol, 40%, *o*:*p* >20:1) as red oil.

^{1}H NMR (400 MHz, $CDCl_3$) δ 6.52 – 5.96 (m, 2H), 3.25 (br s, 4H). ^{13}C NMR (101 MHz, $CDCl_3$) δ 156.8 (dd, *J* = 237.8, 14.9 Hz), 153.0 (dd, *J* = 236.8, 14.8 Hz), 138.7 (dd, *J* = 12.4, 7.1 Hz), 117.3 (dd, *J* = 16.3, 3.5 Hz), 98.4 (dd, *J* = 25.4, 2.9 Hz), 93.9 (dd, *J* = 26.6, 25.0 Hz). ^{19}F NMR (376 MHz, $CDCl_3$) δ -121.57, -130.43. HRMS (ESI) calcd for $[C_6H_6N_2F_2 + H]^+$: 145.0572. Found: 145.0573.

4-Methoxy-3-methylbenzene-1,2-diamine (4h-i) and 5-methoxy-4-methyl-1H-benzo[d]imidazole (4h-ii)

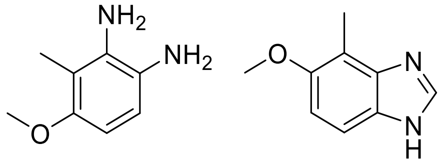

4h-i **4h-ii**

Tetrabutylammonium (3-methoxy-2-methylphenyl)sulfamate **1m** (229 mg, 0.499 mmol, 1 equiv), **2c** (249 mg, 0.750 mmol, 1.5 equiv), $FeBr_2$ (16.2 mg, 0.0751 mmol, 0.15 equiv) were subject to **GP-4**. Purification by silica gel chromatography (eluent: Pet. Ether:EtOAc, 80:20 – 40:60) gave the title compound **4h-i** (33.6 mg, 0.221 mmol, 44%, *o*:*p* >20:1) as a brown solid.

^{1}H NMR (400 MHz, $CDCl_3$) δ 6.57 (d, *J* = 8.4 Hz, 1H), 6.26 (d, *J* = 8.4 Hz, 1H), 3.76 (s, 3H), 3.34 (br s, 4H), 2.08 (s, 3H). ^{13}C NMR (101 MHz, $CDCl_3$) δ 152.5, 135.9, 127.0, 111.5, 101.3, 56.0, 9.4. HRMS (ESI) calcd for $[C_8H_{12}N_2O + Na]^+$: 175.0842. Found: 175.0834.

To confirm the *ortho* regioselectivity the title compound **4h-i** was converted to the corresponding benzimidazole. To achieve this trimethyl orthoformate (1 mL) and concentrated hydrochloric acid (2 drops) were added to 4-methoxy-3-methylbenzene-1,2-diamine **4h-i** (33.6 mg, 0.221 mmol) and the reaction was stirred at 80℃ for 1 h. The reaction was concentrated under a stream of nitrogen and aqueous Na_2CO_3 was then added to the resulting residue. The organics were extracted with chloroform three times, dried ($MgSO_4$), filtered and concentrated *in vacuo*. Purification by silica gel chromatography (eluent: EtOAc:MeOH, 98:2) gave the title compound **4h-ii** (19.4 mg, 0.119 mmol, 55%) as a yellow solid.

^{1}H NMR (400 MHz, $CDCl_3$) δ 8.03 (s, 1H), 7.46 (d, *J* = 8.9 Hz, 1H), 6.96 (d, *J* = 8.8 Hz, 1H), 3.89 (s, 3H), 2.46 (s, 3H). ^{13}C NMR (101 MHz, $CDCl_3$) δ 153.7, 140.4, 137.3, 133.1, 113.2, 112.0, 108.8, 57.1, 10.5. HRMS (ESI) calcd for $[C_9H_{10}N_2O + H]^+$: 163.0866. Found: 163.0864.

Note: ^{1}H NMR analysis showed an 8:1 mixture of product tautomers. Only peaks for the major tautomer reported.

3-Bromo-4-methylbenzene-1,2-diamine (4i)

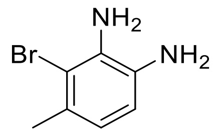

Tetrabutylammonium (2-bromo-3-methylphenyl)sulfamate **1s** (203 mg, 0.400 mmol, 1 equiv), **2d** (199 mg, 0.599 mmol, 1.5 equiv), $FeBr_2$ (13.0 mg, 0.0603 mmol, 0.15 equiv) were subject to **GP-4**. Purification by silica gel chromatography (eluent: Pet. Ether:EtOAc, 80:20 – 40:60) gave the title compound (32.2 mg, 0.160 mmol, 40%, *o*:*p* >20:1) as an orange solid.

^{1}H NMR (400 MHz, MeOD-d_4) δ 6.63 (d, *J* = 7.9 Hz, 1H), 6.52 (d, *J* = 7.9 Hz 1H), 2.25 (s, 3H). ^{13}C NMR (101 MHz, MeOD-d_4) δ 133.6, 131.0, 129.0, 119.5, 115.5, 112.4, 21.8. HRMS (ESI) calcd for $[C_7H_9BrN_2 + H]^+$: 201.0022. Found: 201.0019.

N-Benzylbenzene-1,2-diamine (4j)

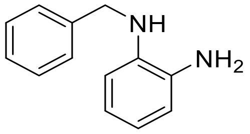

Tetrabutylammonium *N*-benzyl(phenyl)sulfamate **1ab** (252 mg, 0.499 mmol, 1 equiv), **2a** (317 mg, 0.749 mmol, 1.5 equiv), $FeBr_2$ (16.2 mg, 0.0751 mmol, 0.15 equiv) were subject to **GP-4**. Purification by silica gel chromatography (eluent: Pet. Ether:EtOAc, 90:10 – 60:40) gave the title compound (34.6 mg, 0.174 mmol, 35%, *o*:*p* >20:1) as a red oil.

^{1}H NMR (700 MHz, $CDCl_3$) δ 7.44 (d, *J* = 7.5 Hz, 2H), 7.38 (t, *J* = 7.4 Hz, 2H), 7.31 (t, *J* = 7.4 Hz, 1H), 6.83 (td, *J* = 7.4, 1.8 Hz, 1H), 6.80 – 6.76 (m, 2H), 6.74 (d, *J* = 7.9 Hz, 1H), 4.36 (s, 2H), 3.75 (br s, 3H). ^{13}C NMR (176 MHz, $CDCl_3$) δ 138.7, 136.4, 134.5, 128.6, 128.1, 127.4, 120.73, 119.7, 116.8, 113.1, 48.9.

Data match literature values.[23]

N-Isopropylbenzene-1,2-diamine (4k)

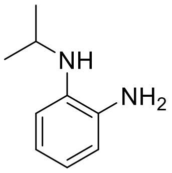

Tetrabutylammonium *N*-isopropyl(phenyl)sulfamate **1ac** (228 mg, 0.499 mmol, 1 equiv), **2c** (166 mg, 0.500 mmol, 1 equiv), $FeBr_2$ (16.2 mg, 0.0751 mmol, 0.15 equiv) were subject to **GP-4**. Purification by silica gel chromatography (eluent: Pet. Ether:EtOAc, 95:5 – 65:35) gave the title compound (39.9 mg, 0.266 mmol, 53%, *o*:*p* >20:1) as a red oil.

^{1}H NMR (400 MHz, $CDCl_3$) δ 6.87 – 6.77 (m, 1H), 6.77 – 6.64 (m, 3H), 3.62 (hept, *J* = 6.2 Hz, 1H), 3.41 (br s, 3H), 1.25 (d, *J* = 6.3 Hz, 6H). ^{13}C NMR (101 MHz, $CDCl_3$) δ 136.1, 134.7, 120.6, 118.9, 116.8, 113.4, 44.6, 23.0.

Data match literature values.[24]

N-Methylbenzene-1,2-diamine (4l)

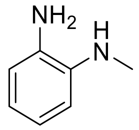

Tetrabutylammonium *N*-methyl(phenyl)sulfamate **1ad** (207 mg, 0.499 mmol, 1 equiv), **2d** (238 mg, 0.750 mmol, 1.5 equiv), $FeBr_2$ (16.2 mg, 0.0751 mmol, 0.15 equiv) were subject to **GP-4**. Purification by silica gel chromatography (eluent: Pet. Ether:EtOAc, 90:10 – 60:40) gave the title compound (25.7 mg, 0.210 mmol, 42%, *o*:*p* >20:1) as a brown oil.

^{1}H NMR (400 MHz, $CDCl_3$) δ 6.94 – 6.83 (m, 1H), 6.82 – 6.68 (m, 3H), 3.87 (br s, 3H), 2.91 (s, 3H). ^{13}C NMR (101 MHz, $CDCl_3$) δ 137.2, 134.7, 120.7, 119.8, 116.6, 112.5, 31.5.

Data match literature values.[16]

10.Product Functionalisations

1,4,5-Trimethyl-1H-benzo[d]imidazole (6a)

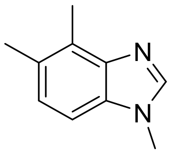

A suspension of tetrabutylammonium (2,3-dimethylphenyl)sulfamate **1k** (221 mg, 0.499 mmol, 1 equiv), **2c** (248 mg, 0.749 mmol, 1.5 equiv) and $FeBr_2$ (16.2 mg, 0.0751 mmol, 0.15 equiv) in HFIP (1 mL) was stirred at 30°C for 4 h. The reaction was concentrated under a stream of air and HCl/MeOH (1.25 M, 2 mL) added. The resulting solution was stirred at room temperature for 1 h. The reaction was concentrated under a stream of air and aqueous NaOH was added. The aqueous layer was then extracted with chloroform three times. The combined organics were then dried ($MgSO_4$), filtered and concentrated *in vacuo*. Trimethyl orthoformate (1 mL) was added to the resulting residue followed by concentrated hydrochloric acid (2 drops). The resulting solution was then stirred at 80°C for 2 h. The reaction was then concentrated under a stream of air and aqueous Na_2CO_3 was added. The aqueous layer was then extracted with chloroform three times and the combined organics were dried ($MgSO_4$), filtered and concentrated *in vacuo*. The crude residue was then purified

by silica gel chromatography (eluent: Pet. Ether:EtOAc, 40:60 – 25:75) to give the title compound (64.2 mg, 0.401 mmol, 80%) as a white solid.

^{1}H NMR (400 MHz, CD_3CN) δ 7.93 (s, 1H), 7.20 (d, *J* = 8.2 Hz, 1H), 7.12 (d, *J* = 8.2 Hz, 1H), 3.77 (s, 3H), 2.50 (s, 3H), 2.36 (s, 3H). ^{13}C NMR (101 MHz, CD_3CN) δ 143.1, 142.4, 132.6, 129.4, 126.8, 125.0, 106.8, 30.6, 18.3, 12.4. HRMS (ESI) calcd for $[C_{10}H_{12}N_2 + H]^+$: 161.1073. Found: 161.1074.

1,6,7-Trimethyl-1H-benzo[d]imidazole (6b)

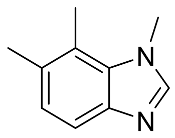

A suspension of tetrabutylammonium *N*-methyl-(2,3-dimethylphenyl)sulfamate **1ae** (229 mg, 0.501 mmol, 1 equiv), **2c** (249 mg, 0.751 mmol, 1.5 equiv) and $FeBr_2$ (16.2 mg, 0.075 mmol, 0.15 equiv) in HFIP (1 mL) was stirred at 30°C for 4 h. The reaction was concentrated under a stream of air and HCl/MeOH (1.25 M, 2 mL) added. The resulting solution was stirred at room temperature for 1 h. The reaction was concentrated under a stream of air and aqueous NaOH was added. The aqueous layer was then extracted with chloroform three times. The combined organics were then dried ($MgSO_4$), filtered and concentrated *in vacuo*. To remove NBu_4OTf, the resulting residue was then filtered through a plug of silica (eluent: Pet. Ether:EtOAc, 40:60. The filtrate was then concentrated *in vacuo*. Trimethyl orthoformate (1 mL) was added to the resulting residue followed by concentrated hydrochloric acid (2 drops). The resulting solution was then stirred at 80°C for 2 h. The reaction was then concentrated under a stream of air and aqueous Na_2CO_3 was added. The aqueous layer was then extracted with chloroform three times and the combined organics were dried ($MgSO_4$), filtered and concentrated *in vacuo*. The crude residue was then purified by silica gel chromatography (eluent: EtOAc:MeOH, 100:0 – 97:3) to give the title compound (29.4 mg, 0.184 mmol, 37%) as a white solid.

^{1}H NMR (400 MHz, $CDCl_3$) δ 7.95 (s, 1H), 7.54 (d, *J* = 8.2 Hz, 1H), 7.11 (d, *J* = 8.1 Hz, 1H), 4.09 (s, 3H), 2.63 (s, 3H), 2.41 (s, 3H). ^{13}C NMR (101 MHz, $CDCl_3$) δ 144.4, 141.7, 133.3, 131.7, 125.5, 119.7, 116.9, 35.0, 19.9, 14.0. HRMS (ESI) calcd for $[C_{10}H_{12}N_2 + H]^+$: 161.1073. Found: 161.1070.

1,4,5-Trimethyl-1H-benzo[d][1,2,3]triazole (6c)

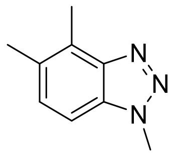

A suspension of tetrabutylammonium (2,3-dimethylphenyl)sulfamate **1k** (221 mg, 0.499 mmol, 1 equiv), **2c** (248 mg, 0.749 mmol, 1.5 equiv) and $FeBr_2$ (16.2 mg, 0.0751 mmol, 0.15 equiv) in HFIP (1 mL) was stirred at 30℃ for 4 h. The reaction was concentrated under a stream of air and HCl/MeOH (1.25 M, 1.0 mL) added. The resulting solution was stirred at room temperature for 1 h. The reaction was concentrated under a stream of air and aqueous NaOH was added. The aqueous layer was then extracted with chloroform three times. The combined organics were then dried ($MgSO_4$), filtered and concentrated *in vacuo*. The resulting residue was then filtered through a plug of silica (eluent: CH_2Cl_2:MeOH, 95:5) and the filtrate was concentrated *in vacuo*. Dry acetonitrile (0.5 mL) was then added to the resulting residue followed by tetrafluoroboric acid (48% wt in H_2O, 132 µL, 1.01 mmol, 2 equiv) and *tert*-butylnitrite (88 µL, 0.743 mmol, 1.5 equiv). The resulting solution was stirred at room temperature for 14 h. The reaction was quenched with aqueous Na_2CO_3 and the aqueous layer was extracted with chloroform three times. The combined organics were dried ($MgSO_4$), filtered and concentrated *in vacuo*. The crude residue was then purified by silica gel chromatography (eluent: Pet. Ether:EtOAc, 95:5 – 70:30) to give the title compound (49.9 mg, 0.309 mmol, 62%) as a yellow oil.

^1H NMR (400 MHz, $CDCl_3$) δ 7.27 (d, *J* = 8.4 Hz, 1H), 7.19 (d, *J* = 8.5 Hz, 1H), 4.23 (s, 3H), 2.69 (s, 3H), 2.39 (s, 3H). ^{13}C NMR (101 MHz, $CDCl_3$) δ 146.6, 132.0, 131.0, 130.1, 127.8, 105.6, 34.2, 18.7, 13.4. HRMS (ESI) calcd for $[C_9H_{11}N_3 + H]^+$: 162.1026. Found: 162.1031.

1,6,7-Trimethyl-1H-benzo[d][1,2,3]triazole (6d)

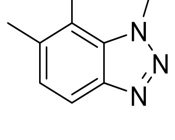

A suspension of tetrabutylammonium *N*-methyl-(2,3-dimethylphenyl)sulfamate **1ae** (228 mg, 0.499 mmol, 1 equiv), **2c** (249 mg, 0.750 mmol, 1.5 equiv) and $FeBr_2$ (16.2 mg, 0.0751 mmol,

0.15 equiv) in HFIP (0.5 mL) was stirred at 30°C for 4 h. The reaction was concentrated under a stream of air and HCl/MeOH (1.25 M, 1.0 mL) added. The resulting solution was stirred at room temperature for 1 h. The reaction was concentrated under a stream of air and aqueous NaOH was added. The aqueous layer was then extracted with chloroform three times. The combined organics were then dried ($MgSO_4$), filtered and concentrated *in vacuo*. The resulting residue was then filtered through a plug of silica (eluent: CH_2Cl_2:MeOH, 95:5) and the filtrate was concentrated *in vacuo*. Dry acetonitrile (0.5 mL) was then added to the resulting residue followed by tetrafluoroboric acid (48% wt in H_2O, 132 µL, 1.01 mmol, 2 equiv) and *tert*-butylnitrite (88 µL, 0.743 mmol, 1.5 equiv). The resulting solution was stirred at room temperature for 14 h. The reaction was quenched with aqueous Na_2CO_3 and the aqueous layer was extracted with chloroform three times. The combined organics were dried ($MgSO_4$), filtered and concentrated *in vacuo*. The crude residue was then purified by silica gel chromatography (eluent: Pet. Ether:EtOAc, 95:5 – 70:30) to give the title compound (34.7 mg, 0.215 mmol, 62%) as a yellow oil.

^1H NMR (400 MHz, $CDCl_3$) δ 7.72 (d, *J* = 8.5 Hz, 1H), 7.13 (d, *J* = 8.5 Hz, 1H), 4.46 (s, 2H), 2.62 (s, 3H), 2.41 (s, 3H). ^{13}C NMR (101 MHz, $CDCl_3$) δ 145.5, 135.5, 133.4, 127.5, 118.1, 116.7, 37.4, 19.7, 14.0. HRMS (ESI) calcd for $[C_9H_{11}N_3 + H]^+$: 162.1026. Found: 162.1031.

2,3-Diphenylquinoxaline (6e)

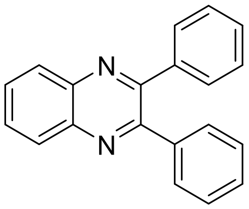

A suspension of tetrabutylammonium phenyl sulfamate **1a** (124 mg, 0.304 mmol, 1 equiv), **2d** (143 mg, 0.451 mmol, 1.5 equiv) and $FeBr_2$ (9.8 mg, 0.0450 mmol, 0.15 equiv) in HFIP (0.6 mL) was stirred at 30°C for 4 h. The reaction was concentrated under a stream of air and HCl/MeOH (1.25 M, 1.2 mL) added. The resulting solution was stirred at room temperature for 1 h. The reaction was concentrated under a stream of air and aqueous NaOH was added. The aqueous layer was then extracted with chloroform three times. The combined organics were then dried ($MgSO_4$), filtered and concentrated *in vacuo*. Ethanol (0.6 mL) was then added to the resulting residue followed by benzil (126 mg, 0.60 mmol, 2 equiv). The resulting

solution was then stirred at 80°C for 16 h. The reaction was then concentrated under a stream of air and aqueous Na_2CO_3 added. The aqueous layer was extracted with chloroform three times and the combined organics were then dried ($MgSO_4$), filtered and concentrated *in vacuo*. The crude residue was then purified by silica gel chromatography (eluent: Pet. Ether:CH_2Cl_2, 80:20 – 0:100) to give the title compound (41.3 mg, 0.146 mmol, 49%) as a white solid.

^{1}H NMR (400 MHz, $CDCl_3$) δ 8.21 (dd, *J* = 6.4, 3.4 Hz, 2H), 7.78 (dd, *J* = 6.4, 3.4 Hz, 2H), 7.56 – 7.50 (m, 4H), 7.40 – 7.30 (m, 6H).^{13}C NMR (101 MHz, $CDCl_3$) δ 153.4, 141.1, 138.9, 130.1, 129.9, 129.1, 128.9, 128.3.

Data matches literature values.[25]

2,2,4-Trimethyl-2,3-dihydro-1H-benzo[b][1,4]diazepine (6f)

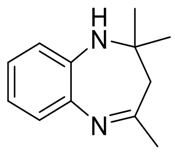

Tetrabutylammonium phenylsulfamate **1a** (207 mg, 0.499 mmol, 1 equiv), **2d** (238 mg, 0.750 mmol, 1.5 equiv), $FeBr_2$ (16.2 mg, 0.0751 mmol, 0.15 equiv) in HFIP (1 mL) was stirred at 30°C for 4 h. The reaction was concentrated under a stream of air and HCl/MeOH (1.25 M, 1.2 mL) added. The resulting solution was stirred at room temperature for 1 h. The reaction was concentrated under a stream of air and aqueous NaOH was added. The aqueous layer was then extracted with chloroform three times. The combined organics were then dried ($MgSO_4$), filtered and concentrated *in vacuo*. The resulting residue was then filtered through a plug of silica (eluent: CH_2Cl_2:MeOH, 95:5) and the filtrate was concentrated *in vacuo*. Acetone (0.5 mL) was then added to the resulting residue followed by Sc(OTf)$_3$ (49.0 mg, 98.5 µmol, 0.1 equiv). The resulting solution as stirred at room temperature for 14 h. The reaction was quenched with aqueous Na_2CO_3 and the aqueous layer was extracted with chloroform three times. The combined organics were dried ($MgSO_4$), filtered and concentrated *in vacuo*. The crude residue was then purified by silica gel chromatography (eluent: Pet. Ether:EtOAc, 90:10 – 40:60) to give the title compound (38.4 mg, 0.204 mmol, 41%) as a yellow solid.

^{1}H NMR (400 MHz, $CDCl_3$) δ 7.19 – 7.06 (m, 1H), 7.00 – 6.94 (m, 2H), 6.84 – 6.61 (m, 1H), 3.04 (br s, 1H), 2.37 (s, 3H), 2.23 (s, 2H), 1.33 (s, 6H). ^{13}C NMR (101 MHz, $CDCl_3$) δ 172.6, 140.4, 138.0, 126.8, 125.6, 122.1, 121.7, 68.3, 45.2, 30.5, 29.8.

Data matches literature values.[26]

11.Amination of a Neutral Substrate

Sodium phenylsulfamate

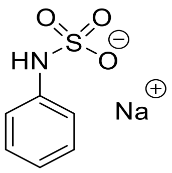

Trimethylamine sulfur trioxide complex (13.9 g, 99.9 mmol, 2 equiv) was added to a stirred solution of aniline (4.56 mL, 4.65 g, 50.0 mmol, 1 equiv) and tributylamine (23.8 mL, 18.6 g, 100 mmol, 2 equiv) in anhydrous CH_3CN (100 mL). The reaction was stirred at 30℃ for 3 h. The reaction was then concentrated in vacuo and ethanol (100 mL) was added to the resulting residue. The resulting suspension was filtered and the filtrate was concentrated *in vacuo*. The resulting residue was then dissolved in EtOAc (100 mL) and the organic layer was washed with water (3 x 50 ml), dried ($MgSO_4$), filtered and concentrated *in vacuo* to give a white solid. The white solid dissolved in CH_3CN (1.25 L) and sodium iodide (11.3 g, 75.4 mmol, 1.5 equiv) was added. The reaction was stirred vigorously at room temperature for 1 h. The resulting precipitate was then collected by filtration and washed with CH_3CN to give the title compound (5.62 g, 28.8 mmol, 58%) as a white solid.

^{1}H NMR (400 MHz, D_2O) δ 7.31 (t, *J* = 7.9 Hz, 2H), 7.14 (d, *J* = 7.8 Hz, 2H), 7.06 (t, *J* = 7.2 Hz, 1H).^{13}C NMR (101 MHz, D_2O) δ 139.8, 129.4, 123.1, 119.2.

Neopentyl phenylsulfamate (7)

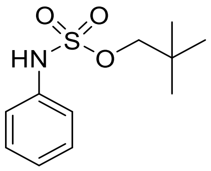

A suspension of sodium phenyl sulfamate (5.00 g, 25.6 mmol, 1 equiv) and phosphorous pentachloride (5.40 g, 26.0 mmol, 1 equiv) in anhydrous toluene (80 mL) was stirred at 80℃ for 18 h. The suspension was then filtered and the filtrate was concentrated *in vacuo*. The resulting residue was dissolved in anhydrous toluene (10 mL) and was added to a stirred suspension of neopentyl alcohol (2.30 g, 26.1 mmol, 1 equiv), Na_2CO_3 (7.80 g, 73.6 mmol, 3 equiv) and benzyltriethylammonium chloride (590 mg, 2.59 mmol, 0.1 equiv) in anhydrous toluene (20 mL) at 0℃. The reaction was stirred at room temperature for 5 h. The reaction was then filtered and the filtrate was concentrated *in vacuo*. The crude residue was then purified by silica gel chromatography (eluent: Pet. Ether:EtOAc, 80:20) to give the title compound (2.05 g, 8.43 mmol, 33%) as a yellow oil.

^{1}H NMR (400 MHz, $CDCl_3$) δ 7.36 (t, *J* = 7.5 Hz, 2H), 7.25 – 7.12 (m, 3H), 7.06 (s, 1H), 3.86 (s, 2H), 0.93 (s, 9H). ^{13}C NMR (101 MHz, $CDCl_3$) δ 136.4, 129.5, 124.8, 119.5, 80.8, 31.6, 26.0. HRMS (ESI) calcd for $[C_{11}H_{16}NO_3S]^-$: 242.0856. Found: 242.0851.

Neopentyl (aminophenyl)sulfamate

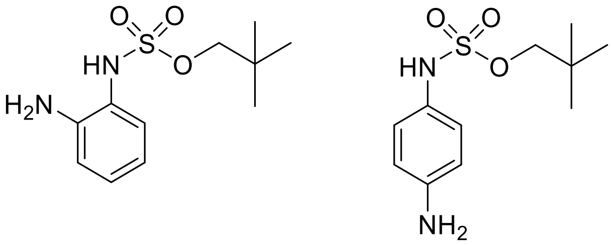

A suspension of neopentyl phenylsulfamate (122 mg, 0.50 mmol, 1 equiv), **2a** (212 mg, 0.50 mmol, 1 equiv) and $FeBr_2$ (16.3 mg, 0.075 mmol, 0.15 equiv) in HFIP (1.0 mL) was stirred at 30 °C for 4 h. Triethylamine (400 μL) was added and the solvent was removed under a stream of air. The crude residue was purified by silica gel column chromatography (Pet. Ether:EtOAc 85:15 – 70:30) to give the *ortho* (39.3 mg, 0.152 mmol, yellow oil) and *para* (35.0 mg, 0.135 mmol, white solid) isomers of the title product as separate isolated samples (overall yield 74.3 mg, 0.288 mmol, 58%), with an *o*:*p* ratio of 1.1:1.

Ortho isomer: ^{1}H NMR (400 MHz, $CDCl_3$) δ 7.23 (d, J = 7.8 Hz, 1H), 7.13 (t, J = 7.8 Hz, 1H), 6.85 – 6.76 (m, 2H), 3.88 (s, 2H), 0.97 (s, 9H). ^{13}C NMR (101 MHz, CDCl3) δ 142.6, 128.8, 127.6,

121.6, 119.5, 117.6, 80.7, 31.8, 26.1. HRMS (ESI) calcd for $[C_{11}H_{18}N_2O_3S + H]^+$: 259.1111. Found: 259.1121.

Para isomer: ^{1}H NMR (400 MHz, $CDCl_3$) δ 7.06 (d, J = 6.7 Hz, 2H), 6.67 (d, J = 6.7 Hz, 2H), 6.43 (br s, 1H), 3.84 (s, 2H), 0.96 (s, 9H). ^{13}C NMR (101 MHz, CDCl3) δ 144.9, 126.7, 124.7, 115.7, 80.5, 31.7, 26.1. HRMS (ESI) calcd for $[C_{11}H_{18}N_2O_3S + H]^+$: 259.1111. Found: 259.1120.

12.Viability of a Phenol Derived Substrate

As detailed in the manuscript (Figure 2c), the amination reaction was shown to be viable using a phenol-derived sulfate substrate (**8**). In this case, the yield of the reaction was found to be lower than for the corresponding aniline substrates, although the high level of regioselectivity for functionalization at the *ortho* position (*o:p* >20:1) were maintained. Exploration of reaction variables for this substrate is described below.

Optimisation

General procedure for screening reactions: A suspension of tetrabutylammonium sulfate salt, aminating agent and Fe(II) source was stirred under air. Following reaction completion, solvent was removed under a stream of air and HCl/MeOH (1.25 M, 5 equiv) was added. The resulting solution was then stirred at room temperature for 1 h. The reaction was quenched by addition of NEt_3 (20 equiv) and the solvent was removed under a stream of air. The crude reaction mixture was filtered through a silica plug with the aid of CH_2Cl_2/MeOH (9:1) or petroleum ether/EtOAc (1:1), concentrated and analysed by ^{1}H NMR.

Following promising results from initial investigations, optimisation commenced using aminating agent **2g**.

Table S8: Evaluation of Fe(II) source

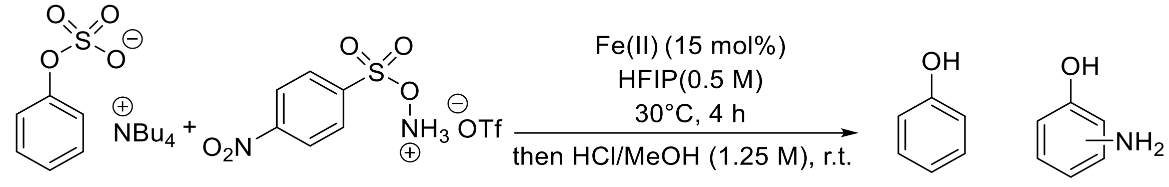

8 (1 equiv) **2g** (1 equiv) **9**

Entry[a]	Fe(II) source	NMR yield (phenol)[b]	NMR yield (9)	*ortho:para* (9)[c]
1	$FeBr_2$	28	35	>20:1
2	$FeSO_4.7H_2O$	30	13	>20:1
3	$FeCl_2$	20	28	>20:1
4	$Fe(OAc)_2$	18	18	>20:1
5	Ferrocene	10	19	>20:1

[a] Reactions conducted on 0.1 mmol scale. [b] Yields were determined by ^{1}H NMR analysis of the crude reaction mixture using 1,2-dimethoxyethane as an internal standard. [c] Regioisomeric ratios were determined by ^{1}H NMR.

Table S9: Evaluation of reaction solvent

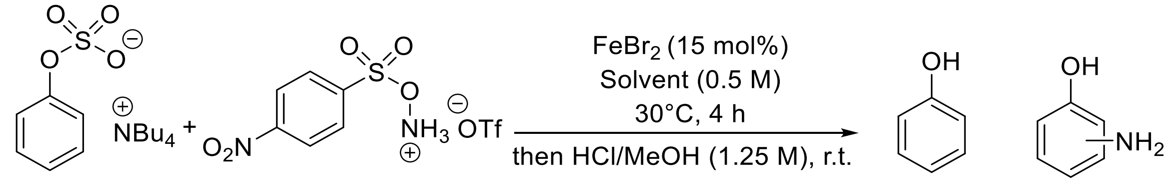

8 (1 equiv) **2g** (1 equiv) **9**

Entry[a]	Solvent	NMR yield (phenol)[b]	NMR yield (9)	*ortho:para* (9)[c]
1	HFIP	28	35	>20:1
2	TFE	21	14	>20:1
3	MeOH	49	0	-
4	EtOAc	43	<5	-
5	THF	71	0	-
6	MeCN	54	0	-

[a] Reactions conducted on 0.1 mmol scale. [b] Yields were determined by ^{1}H NMR analysis of the crude reaction mixture using 1,2-dimethoxyethane as an internal standard. [c] Regioisomeric ratios were determined by ^{1}H NMR.

Table S10: Evaluation of reaction concentration

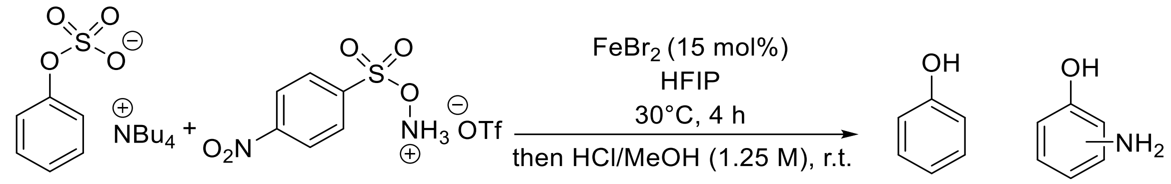

8 (1 equiv) 2g (1 equiv) 9

Entry[a]	Concentration	NMR yield (phenol)[b]	NMR yield (9)	*ortho:para* (9)[c]
1	0.5	28	35	>20:1
2	0.2	26	22	>20:1
3	0.1	23	14	>20:1
4	0.05	49	<5	-

[a] Reactions conducted on 0.1 mmol scale. [b] Yields were determined by ^{1}H NMR analysis of the crude reaction mixture using 1,2-dimethoxyethane as an internal standard. [c] Regioisomeric ratios were determined by ^{1}H NMR

Table S11: Evaluation of reaction time

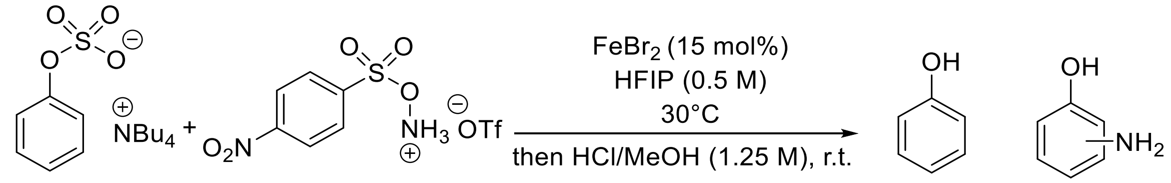

8 (1 equiv) 2g (1 equiv) 9

Entry[a]	Reaction time (h)	NMR yield (phenol)[b]	NMR yield (9)	*ortho:para* (9)[c]
1	0.5	55	<5	-
2	1	50	8	>20:1
3	2	52	10	>20:1
4	4	30	35	>20:1
5	16	8	30	>20:1

[a] Reactions conducted on 0.1 mmol scale. [b] Yields were determined by ^{1}H NMR analysis of the crude reaction mixture using 1,2-dimethoxyethane as an internal standard. [c] Regioisomeric ratios were determined by ^{1}H NMR.

Table S12: Evaluation of aminating agents

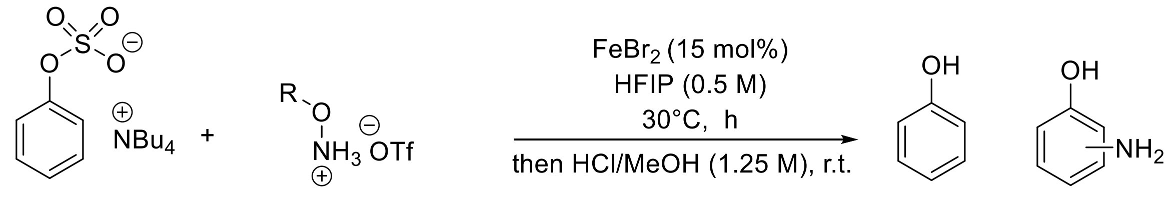

8

(1 equiv)

9

R =

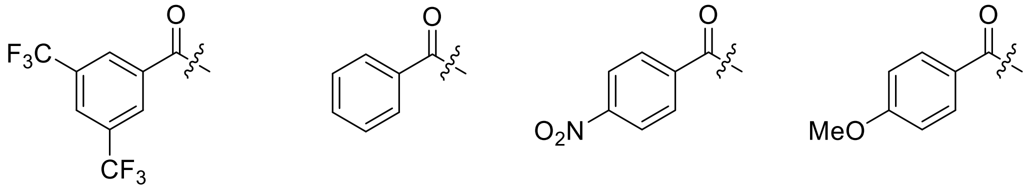

2a 2b 2c 2d

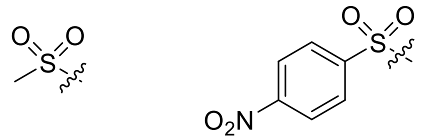

2f 2g

Entry[a]	Aminating agent	Equivalents of aminating agent	NMR yield (phenol)[b]	NMR yield (9)	*ortho:para* (9)[c]
1	**2a**	1	46	22	>20:1
2	**2a**	1.5	49	<5	-
3	**2b**	1	23	26	>20:1
4	**2b**	1.5	20	26	>20:1
5	**2c**	1	6	36	>20:1
6	**2c**	1.5	10	33	>20:1
7	**2d**	1	64	0	-
8	**2d**	1.5	55	0	-
9	**2f**	1	15	14	>20:1
10	**2f**	1.5	16	28	>20:1
11	**2g**	1	30	35	>20:1
12	**2g**	1.5	15	30	>20:1

[a] Reactions conducted on 0.1 mmol scale. [b] Yields were determined by ^{1}H NMR analysis of the crude reaction mixture using 1,2-dimethoxyethane as an internal standard. [c] Regioisomeric ratios were determined by ^{1}H NMR.

Evaluation of phenol

The origin of selectivity was probed by using phenol as a substrate for the amination reaction. Although unreactive under the optimised $FeBr_2$-catalysed conditions, reactivity could be achieved using conditions analogous to those reported by Ritter *et al.*[2] The amination product of the reaction was obtained as a statistical mixture of *ortho* and *para* isomers, providing further support for our hypothesis that the observed regioselectivity of the amination can be attributed to attractive non-covalent interactions between the substrate and the aminium radical cation. Under the same reaction conditions, sulfate salt **8** exhibited low reactivity (10% NMR yield) but high selectivity (*o:p* >20:1).

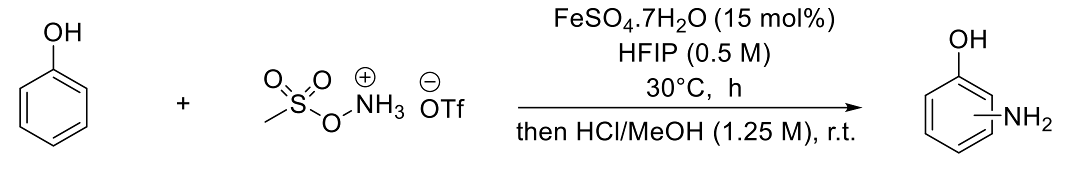

(1 equiv) **2f** (1 equiv) **9** 40% *o:p* 2.1:1

A suspension of phenol (47 mg, 0.50 mmol, 1 equiv), **2f** (131 mg, 0.50 mmol, 1 equiv) and $FeSO_4.7H_2O$ (1.4 mg, 0.005 mmol, 0.01 equiv) in HFIP (1.5 mL) was stirred at 60 °C for 1 h. The reaction was cooled to rt, triethylamine (400 uL) was added and the solvent was removed under a stream of air. The crude residue was purified by silica gel column chromatography (Pet. Ether/EtOAc 80:20 – 70:30) to yield the *ortho* (15.0 mg, brown solid) and *para* (7.0 mg, brown solid) isomers of the title products as separate isolated samples (overall yield 22.0 mg, 0.202 mmol, 40%), with an *o:p* ratio of 2.1:1.

2-Aminophenol: ^{1}H NMR (400 MHz, DMSO-*d*$_6$) δ 8.92 (br s, 1H), 6.65 (d, *J* = 7.7 Hz, 1H), 6.59 (d, *J* = 7.7 Hz, 1H), 6.55 (t, *J* = 7.4 Hz, 1H), 6.40 (t, *J* = 7.4 Hz, 1H), 4.47 (br s, 1H). ^{13}C NMR (101 MHz, DMSO-*d*$_6$) δ 144.4, 137.0, 120.0, 116.9, 114.9, 114.8.

Data match reported values.[28]

4-Aminophenol: 1H NMR (400 MHz, DMSO-d_6) δ 8.32 (br s, 1H), 6.48 (d, J = 7.6 Hz, 2H), 6.42 (d, J = 7.6 Hz, 2H), 4.38 (br s, 2H). 13C NMR (101 MHz, DMSO-d_6) δ 148.7, 141.2, 116.0, 115.7.

Data match reported values.[29]

Tetrabutylammonium phenyl sulfate (8)

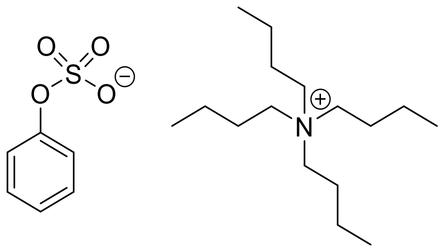

Phenol (0.94 g, 10.0 mmol, 1 equiv), chlorosulfonic acid (0.66 mL, 10 mmol, 1 equiv) and tetrabutylammonium hydrogen sulfate (1.36 g, 8 mmol, 0.8 equiv) were subjected to **GP-1** to give the title compound as a white solid (2.75 g, 6.61 mmol, 66%).

^{1}H NMR (600 MHz, $CDCl_3$) δ 7.40 – 7.39 (m, 2H), 7.27 (dd, *J* = 8.6, 7.4 Hz, 2H), 7.06 (tt, *J* = 7.4, 1.2 Hz, 1H), 3.24 – 3.18 (m, 8H), 1.60 (tdd, *J* = 8.0, 6.1, 4.4 Hz, 8H), 1.41 (h, *J* = 7.4 Hz, 8H), 0.99 (t, *J* = 7.4 Hz, 12H). ^{13}C NMR (101 MHz, $CDCl_3$) δ 153.7, 128.8, 123.4, 121.2, 58.8, 24.0, 19.7, 13.8.

Data match reported values.[4]

2-Aminophenol (9)

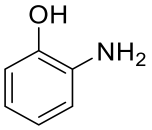

Tetrabutylammonium phenyl sulfate **8** (208 mg, 0.50 mmol, 1 equiv), **2c** (166 mg, 0.50 mmol, 1 equiv) and $FeBr_2$ (16.2 mg, 0.0751 mmol, 0.15 equiv) were subject to **GP-4**. Purification by silica gel column chromatography (Pet. Ether:EtOAc 80:20 – 70:30) yielded the title compound (18.2 mg, 0.0167 mmol, 33%) as a brown solid.

^{1}H NMR (400 MHz, DMSO-d_6) δ 8.92 (br s, 1H), 6.65 (d, J = 7.7 Hz, 1H), 6.59 (d, J = 7.7 Hz, 1H), 6.55 (t, J = 7.4 Hz, 1H), 6.40 (t, J = 7.4 Hz, 1H), 4.47 (br s, 1H). ^{13}C NMR (101 MHz, DMSO-d_6) δ 144.4, 137.0, 120.0, 116.9, 114.9, 114.8.

Data match reported values.[28]

13.Effect of Water Concentration on Selectivity

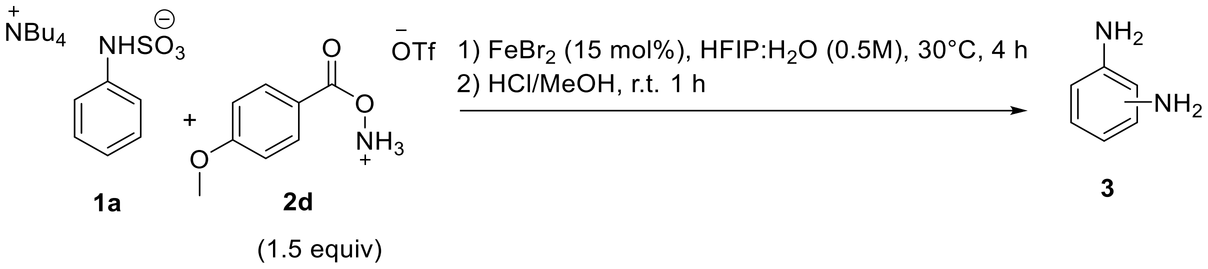

Entry[a]	HFIP:H_2O ratio	*o*:*p* (3)[b]	*o:p (3) -rpt*[b]	*Average o:p*[b]	Average NMR yield
1	20:1	>20:1	>20:1	>20:1	38
2	10:1	18:1	19:1	18.5:1	38
3	5:1	6:1	7:1	6.5:1	42
4	2:1	4:1	4:1	4:1	40
5	1:1	2:1	2:1	2:1	35

[a] Reactions conducted on 0.1 mmol scale. [b] Regioisomer ratios were determined by ^{1}H NMR.

14.References

[1] L. Legnani, G. P. Cerai, B. Morandi, *ACS Catal.* **2016**, *6*, 8162–8165.

[2] E. M. D'Amato, J. Börgel, T. Ritter, *Chem. Sci.* **2019**, *10*, 2424–2428.

[3] J. Liu, K. Wu, T. Shen, Y. Liang, M. Zou, Y. Zhu, X. Li, X. Li, N. Jiao, *Chem. - A Eur. J.* **2017**, *23*, 563–567.

[4] M. T. Mihai, B. D. Williams, R. J. Phipps, *J. Am. Chem. Soc.* **2019**, *141*, 15477–15482.

[5] R. Yanada, K. Hashimoto, R. Tokizane, Y. Miwa, H. Minami, K. Yanada, *J. Org. Chem.* **2008**, *73*, 5135–5138.

[6] S. Fu, H. Yang, G. Li, Y. Deng, H. Jiang, W. Zeng, *Org. Lett.* **2015**, *17*, 1018–1021.

[7] Z. Liang, L. Ju, Y. Xie, L. Huang, Y. Zhang, *Chem. - A Eur. J.* **2012**, *18*, 15816–15821.

[8] Q. Dai, P. Li, N. Ma, C. Hu, *Org. Lett.* **2016**, *18*, 5560–5563.

[9] S. Llona-Minguez, M. Desroses, A. Ghassemian, S. A. Jacques, L. Eriksson, R. Isacksson, T. Koolmeister, P. Stenmark, M. Scobie, T. Helleday, *Chem. - A Eur. J.* **2015**, *21*, 7394–7398.

[10] R. A. Green, J. F. Hartwig, *Org. Lett.* **2014**, *16*, 4388–4391.

[11] J. Bergman, P. Sand, *Tetrahedron* **1990**, *46*, 6085–6112.

[12] O. Halaiev, M. Garazd, A. Gzella, R. Lesyk, *Tetrahedron Lett.* **2017**, *58*, 1324–1325.

[13] P. Patel, S. Chang, *Org. Lett.* **2014**, *16*, 3328–3331.

[14] S. Chatterjee, S. Makai, B. Morandi, *Angew. Chemie - Int. Ed.* **2021**, *60*, 758–765.

[15] C. S. Beshara, A. Hall, R. L. Jenkins, K. L. Jones, T. C. Jones, N. M. Killeen, P. H. Taylor, S. P. Thomas, N. C. O. Tomkinson, *Org. Lett.* **2005**, *7*, 5729–5732.

[16] H. Kohatsu, S. Kamo, S. Tomoshige, K. Kuramochi, *Org. Lett.* **2019**, *21*, 7311–7314.

[17] H. J. Xu, Y. F. Liang, Z. Y. Cai, H. X. Qi, C. Y. Yang, Y. S. Feng, *J. Org. Chem.* **2011**, *76*, 2296–2300.

[18] L. Wang, Z. Yang, M. Yang, R. Zhang, C. Kuai, X. Cui, *Org. Biomol. Chem.* **2017**, *15*, 8302–8307.

[19] H. Goker, M. Alp, Z. Ates-Alagoz, S. Yildiz, *J. Heterocycl. Chem.* **2009**, *46*, 936–948.

[20] Y. Zhou, H. Zhou, S. Liu, D. Pi, G. Shen, *Tetrahedron* **2017**, *73*, 3898–3904.

[21] K. Kawamoto, S. Uchiyama, *Chem. Lett.* **2012**, *41*, 1451–1452.

[22] A. H. Romero, H. Cerecetto, *European J. Org. Chem.* **2020**, *2020*, 1853–1865.

[23] P. Chaudhary, S. Gupta, N. Muniyappan, S. Sabiah, J. Kandasamy, *J. Org. Chem.* **2019**, *84*, 104–119.

[24] W. C. Chen, Y. C. Hsu, C. Y. Lee, G. P. A. Yap, T. G. Ong, *Organometallics* **2013**, *32*, 2435–2442.

[25] Y. Su, J. L. Petersen, T. L. Gregg, X. Shi, *Org. Lett.* **2015**, *17*, 1208–1211.

[26] J. Wang, N. Li, R. Qiu, X. Zhang, X. Xu, S. F. Yin, *J. Organomet. Chem.* **2015**, *785*, 61–67.

[27] S. W. Youn, T. Y. Ko, Y. H. Jang, *Angew. Chemie - Int. Ed.* **2017**, *56*, 6636–6640.

[28] R. J. Rahaim, R. E. Maleczka, *Org. Lett.* **2005**, *7*, 5087–5090.

[29] Y. Motoyama, K. Kamo, H. Nagashima, *Org. Lett.* **2009**, *11*, 1345–1348.

15. NMR Spectra

Tetrabutylammonium Sulfamate Salts

^{1}H NMR of tetrabutylammonium phenylsulfamate **1a** in $CDCl_3$

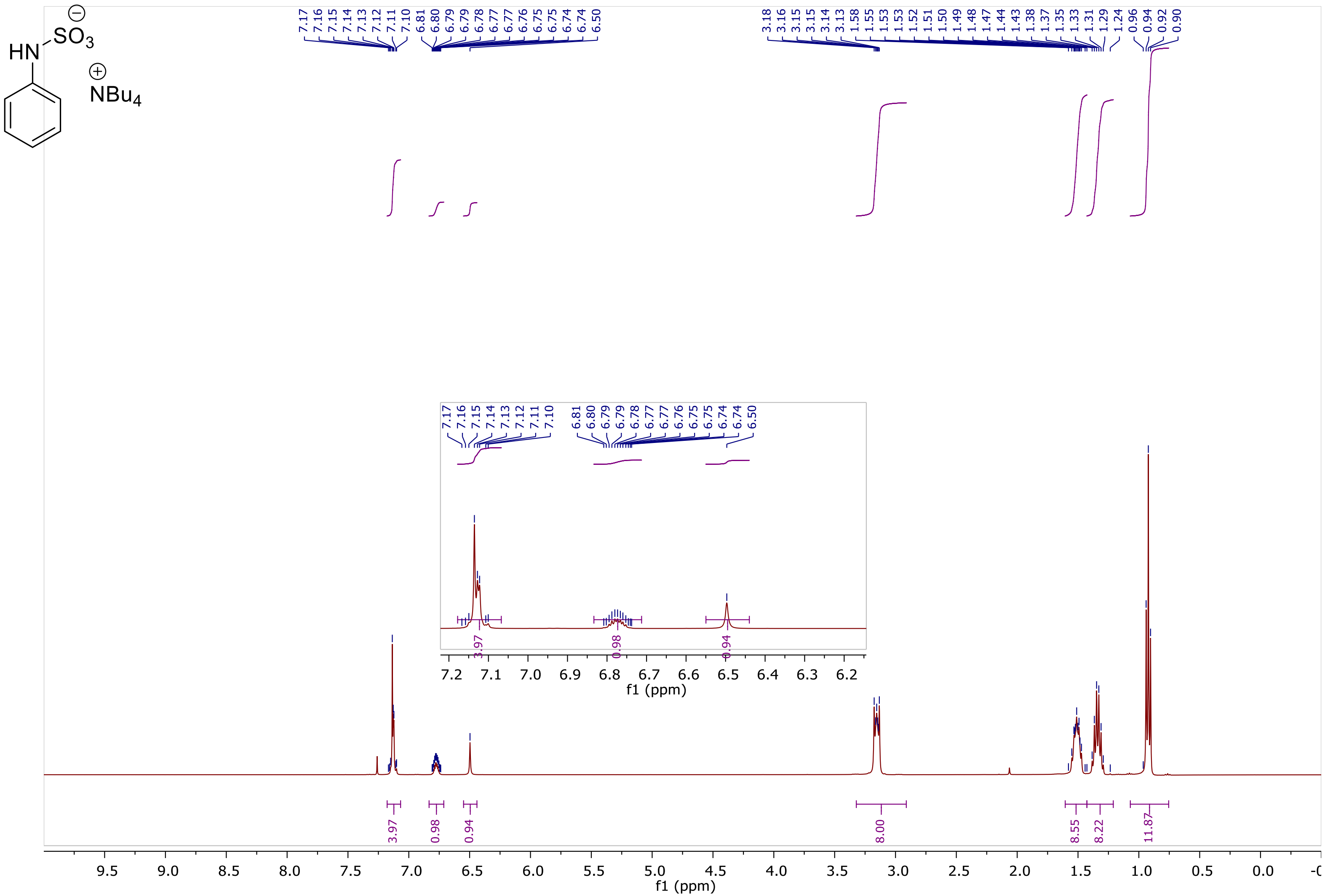

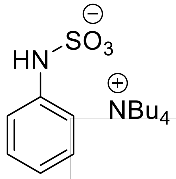

^{13}C NMR of tetrabutylammonium phenylsulfamate **1a** in $CDCl_3$

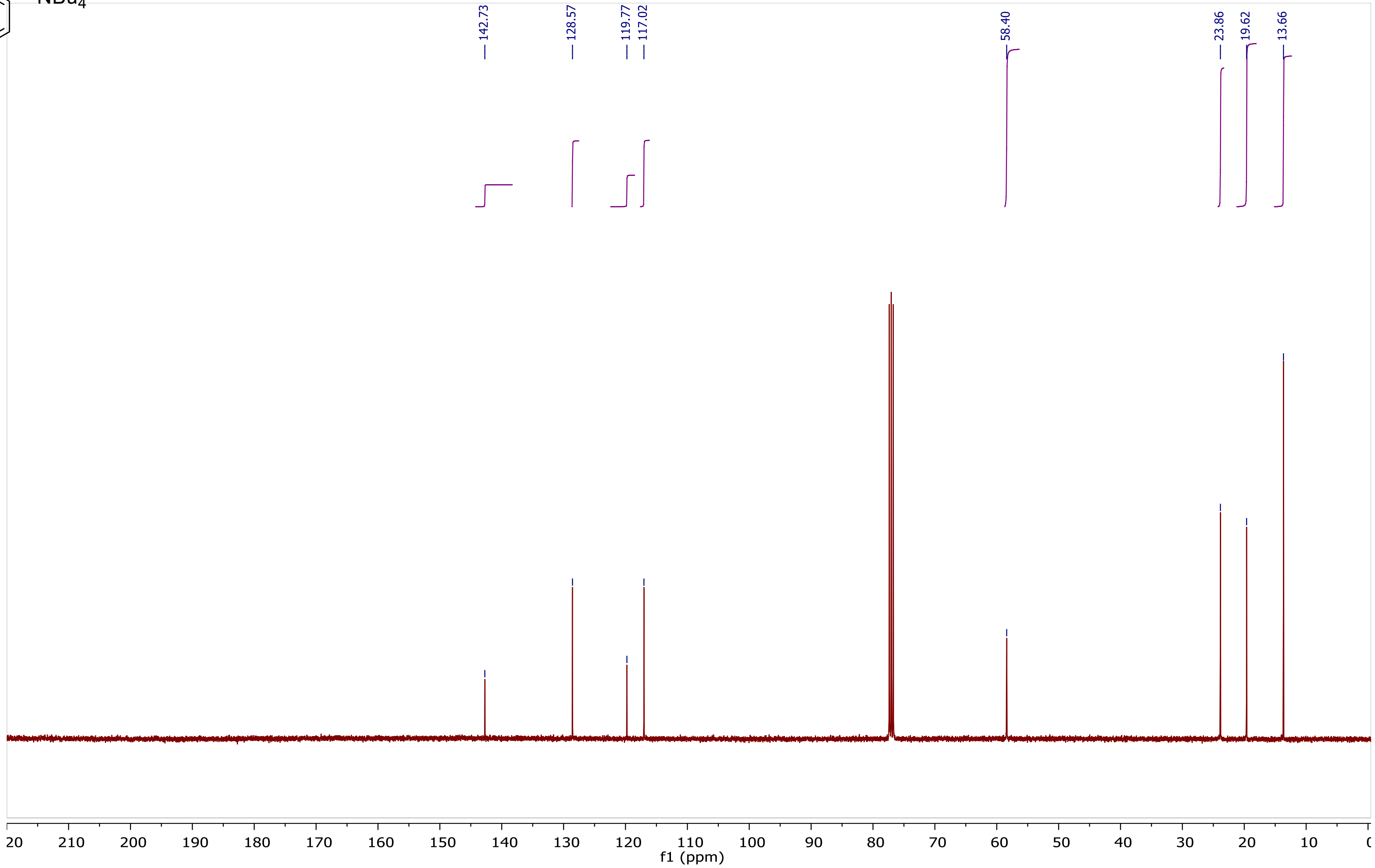

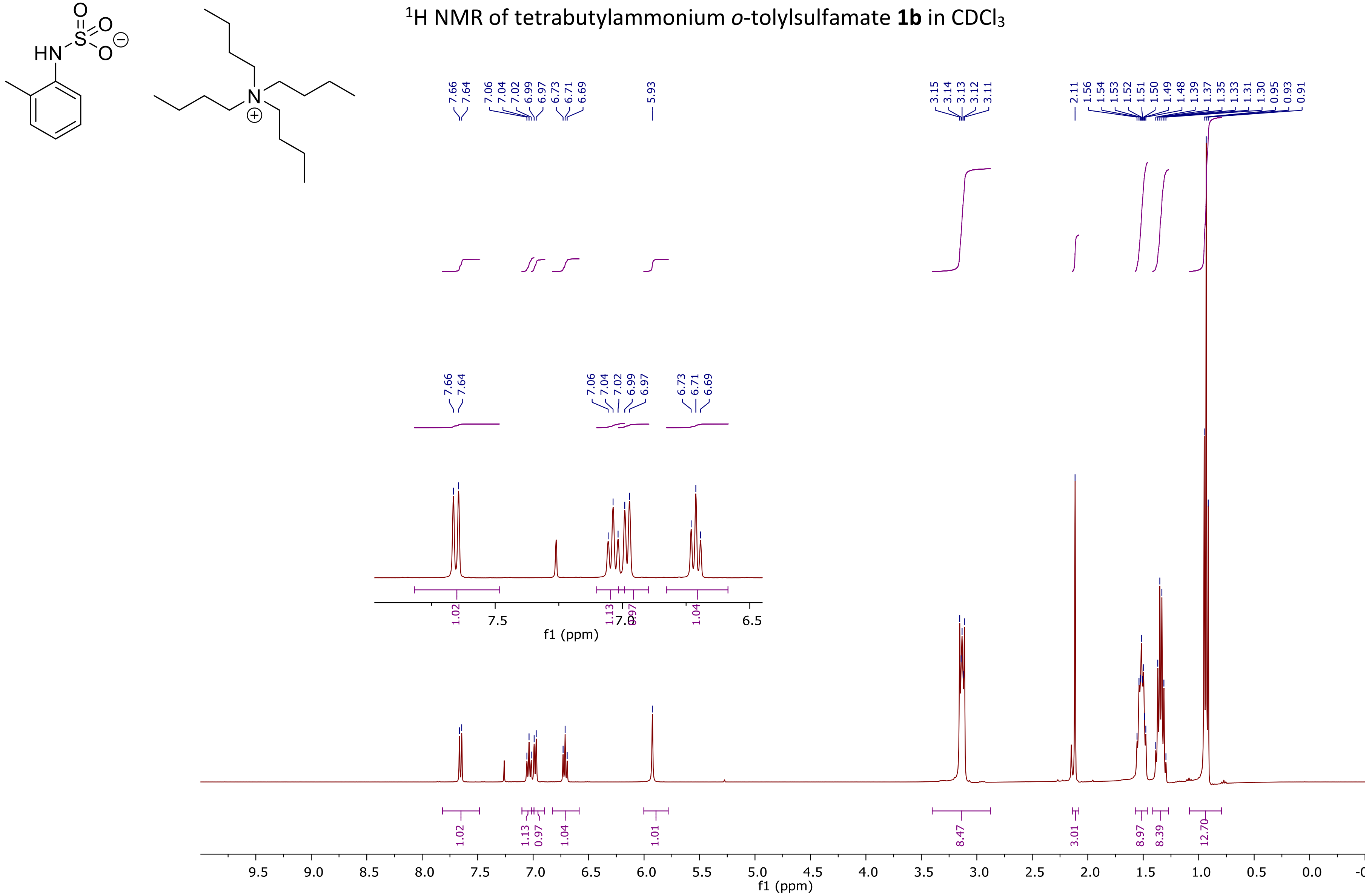

1H NMR of tetrabutylammonium o-tolylsulfamate 1b in CDCl3
f1 (ppm)

^{13}C NMR of tetrabutylammonium *o*-tolylsulfamate **1b** in $CDCl_3$

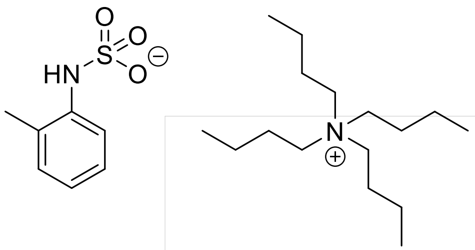

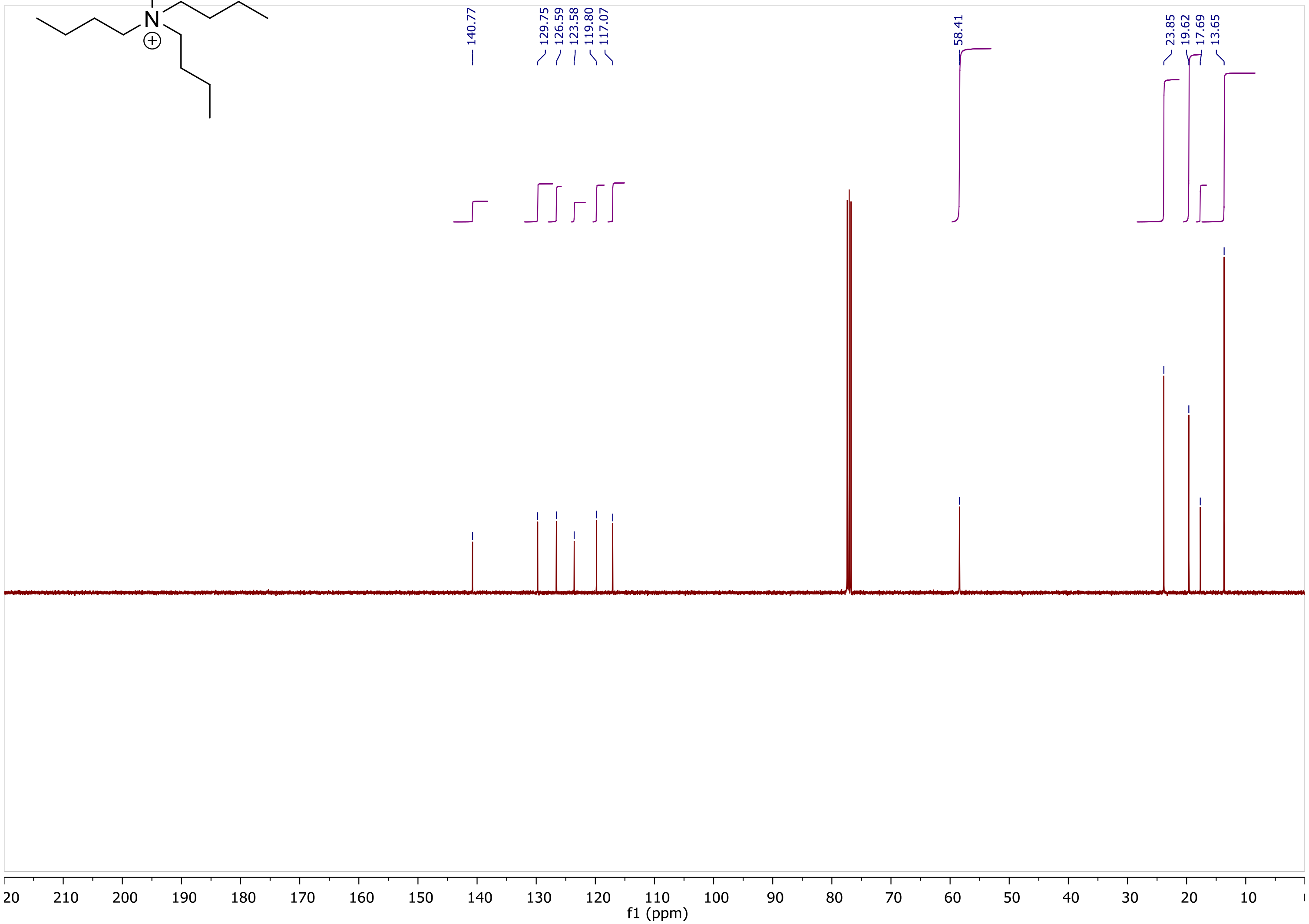

^{1}H NMR of tetrabutylammonium (2-isopropylphenyl)sulfamate **1c** in $CDCl_3$

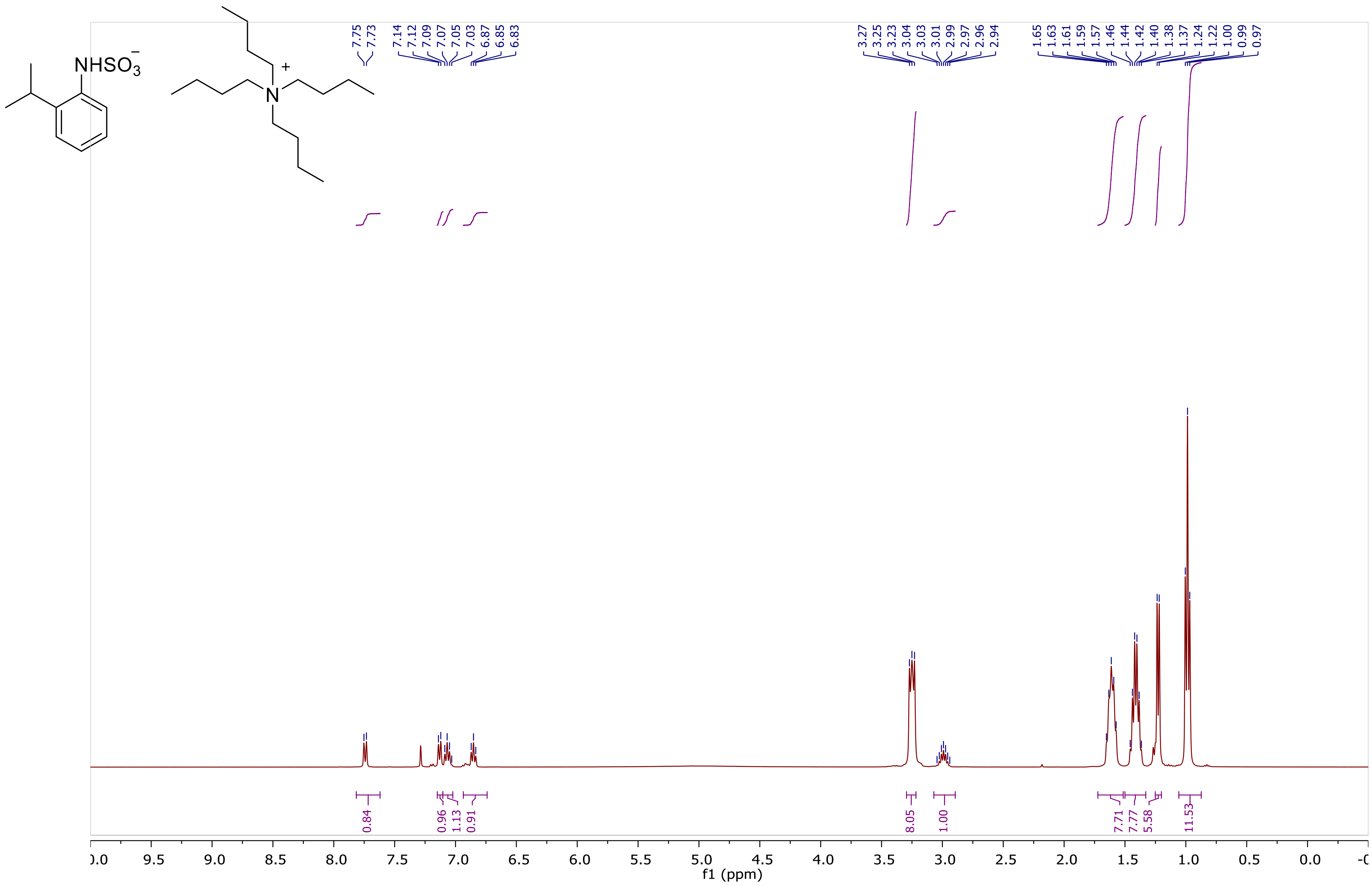

^{13}C NMR of tetrabutylammonium (2-isopropylphenyl)sulfamate **1c** in $CDCl_3$

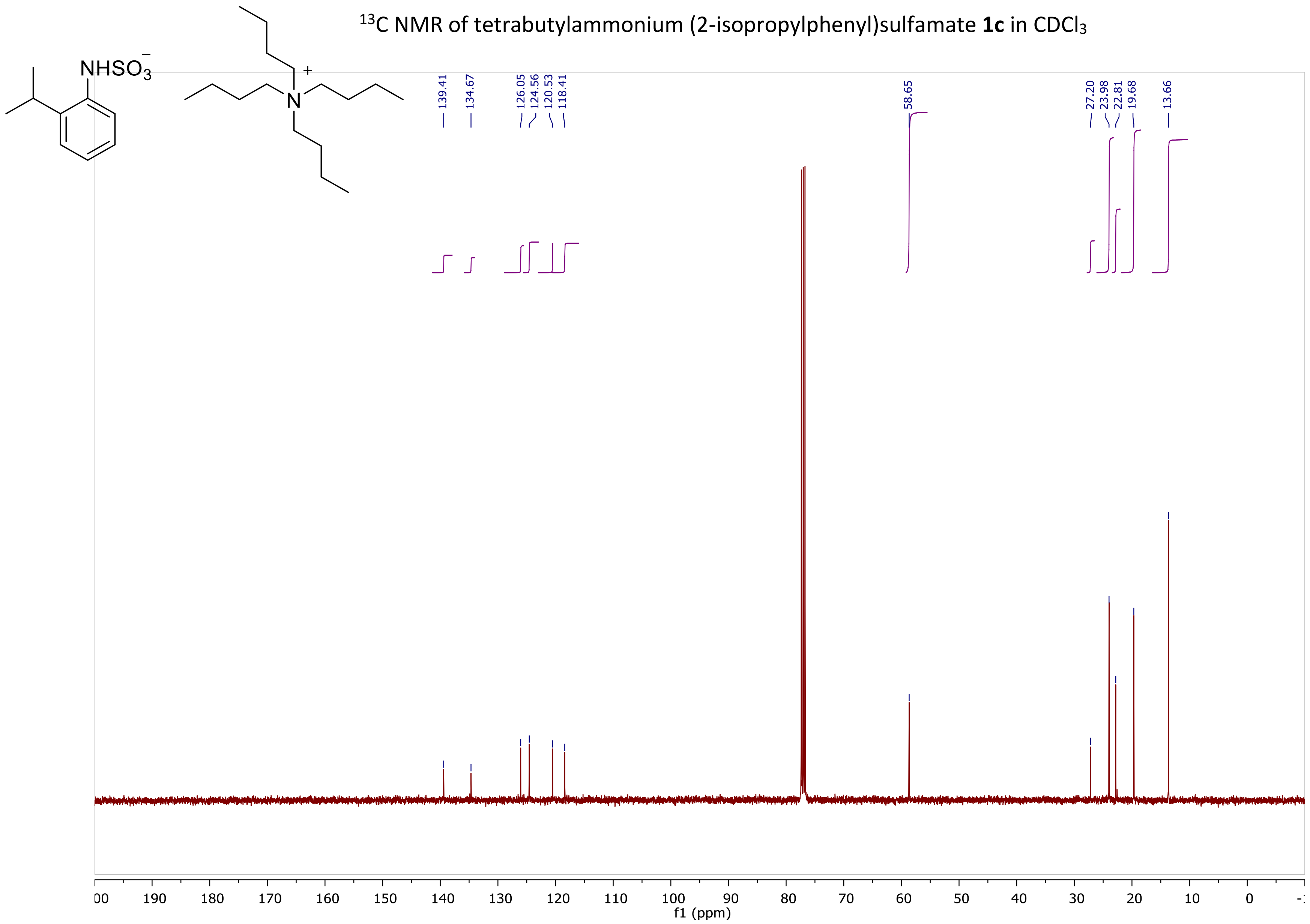

^{1}H NMR of tetrabutylammonium (2-*tert*-butylphenyl)sulfamate **1d** in $CDCl_3$

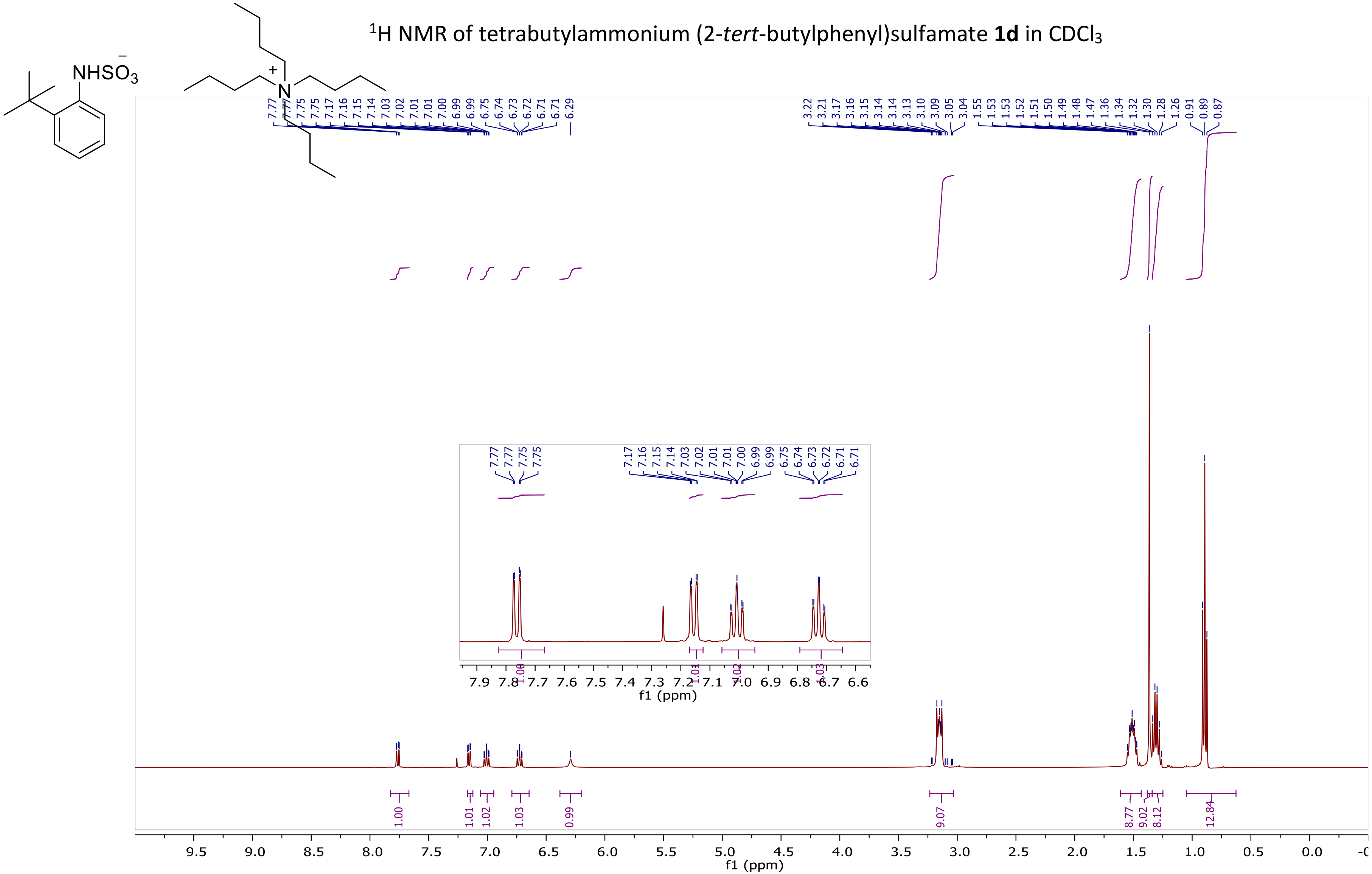

^{13}C NMR of tetrabutylammonium (2-*tert*-butylphenyl)sulfamate **1d** in $CDCl_3$

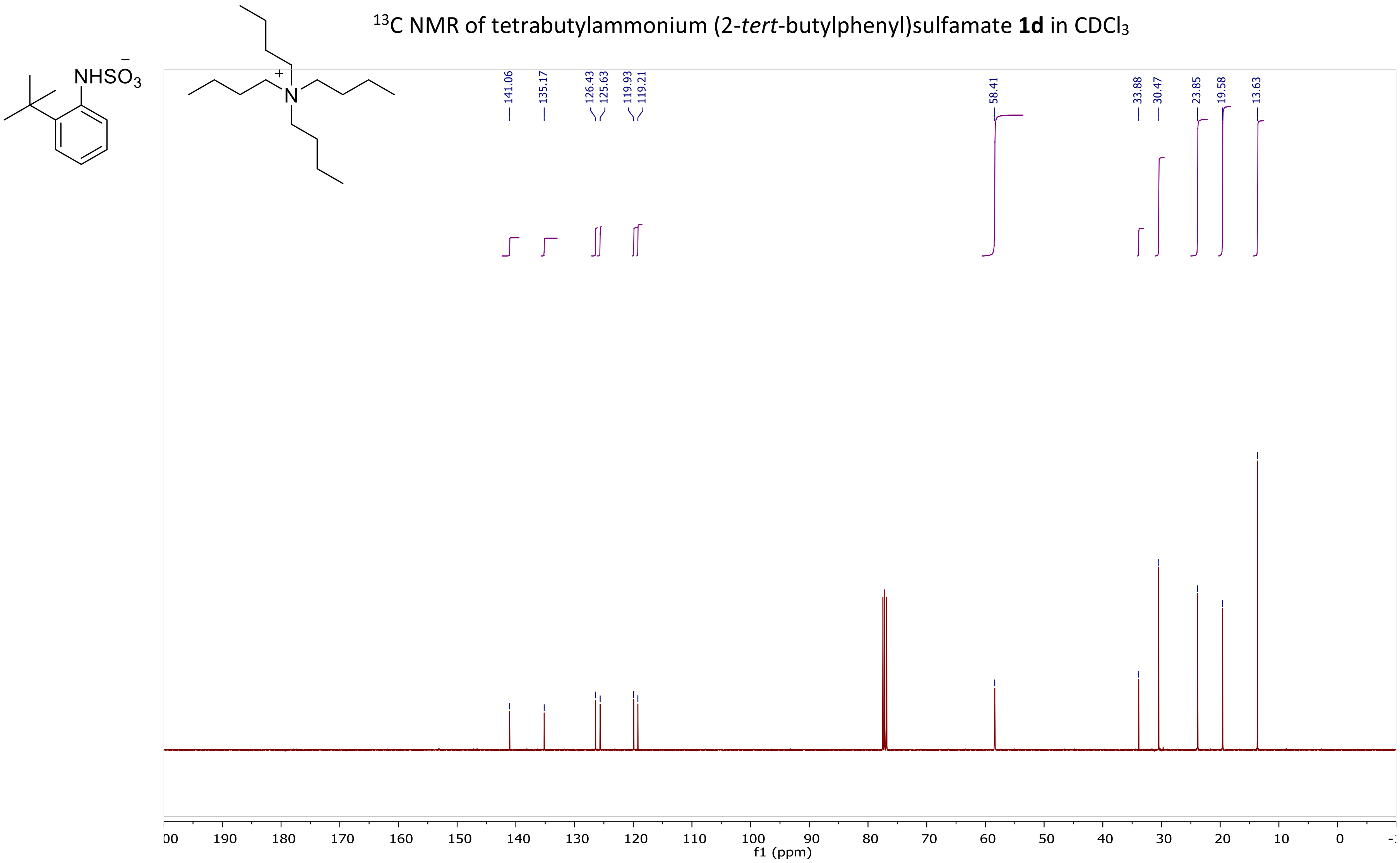

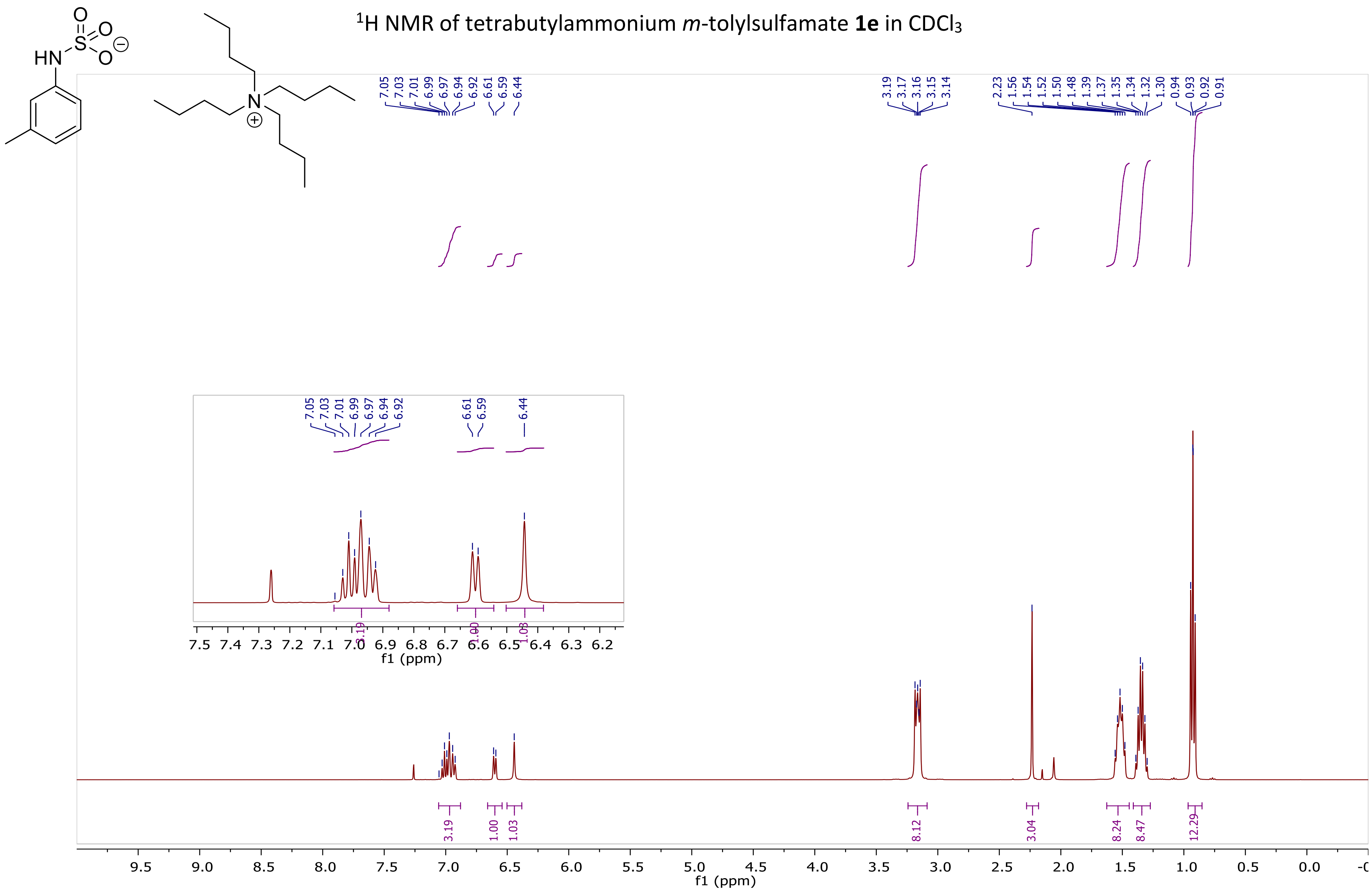

^{1}H NMR of tetrabutylammonium *m*-tolylsulfamate **1e** in $CDCl_3$
7.05 7.03 7.01 6.99 6.97 6.94 6.92 6.61 6.59 6.44
3.19 3.17 3.16 3.15 3.14
2.23 1.56 1.54 1.52 1.50 1.48 1.39 1.37 1.35 1.34 1.32 1.30 0.94 0.93 0.92 0.91
3.19 1.00 1.03 8.12 3.04 8.24 8.47 12.29
f1 (ppm)

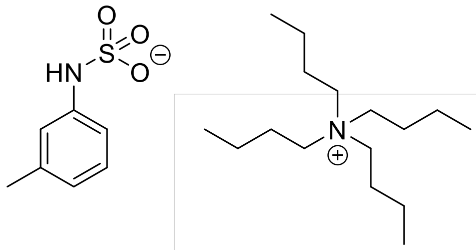

^{13}C NMR of tetrabutylammonium *m*-tolylsulfamate **1e** in $CDCl_3$

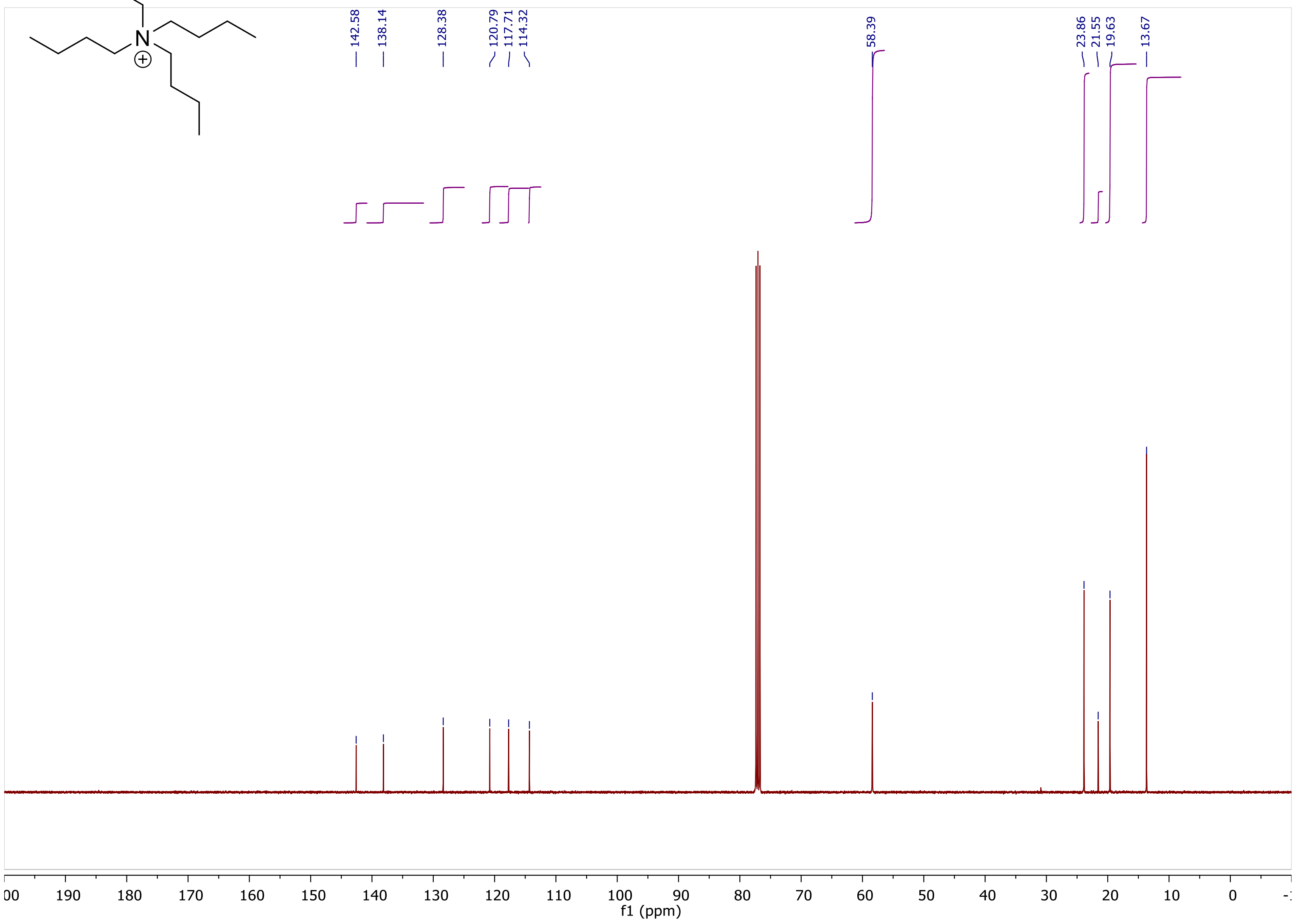

^{1}H NMR of tetrabutylammonium (3-isopropylphenyl)sulfamate **1f** in $CDCl_3$

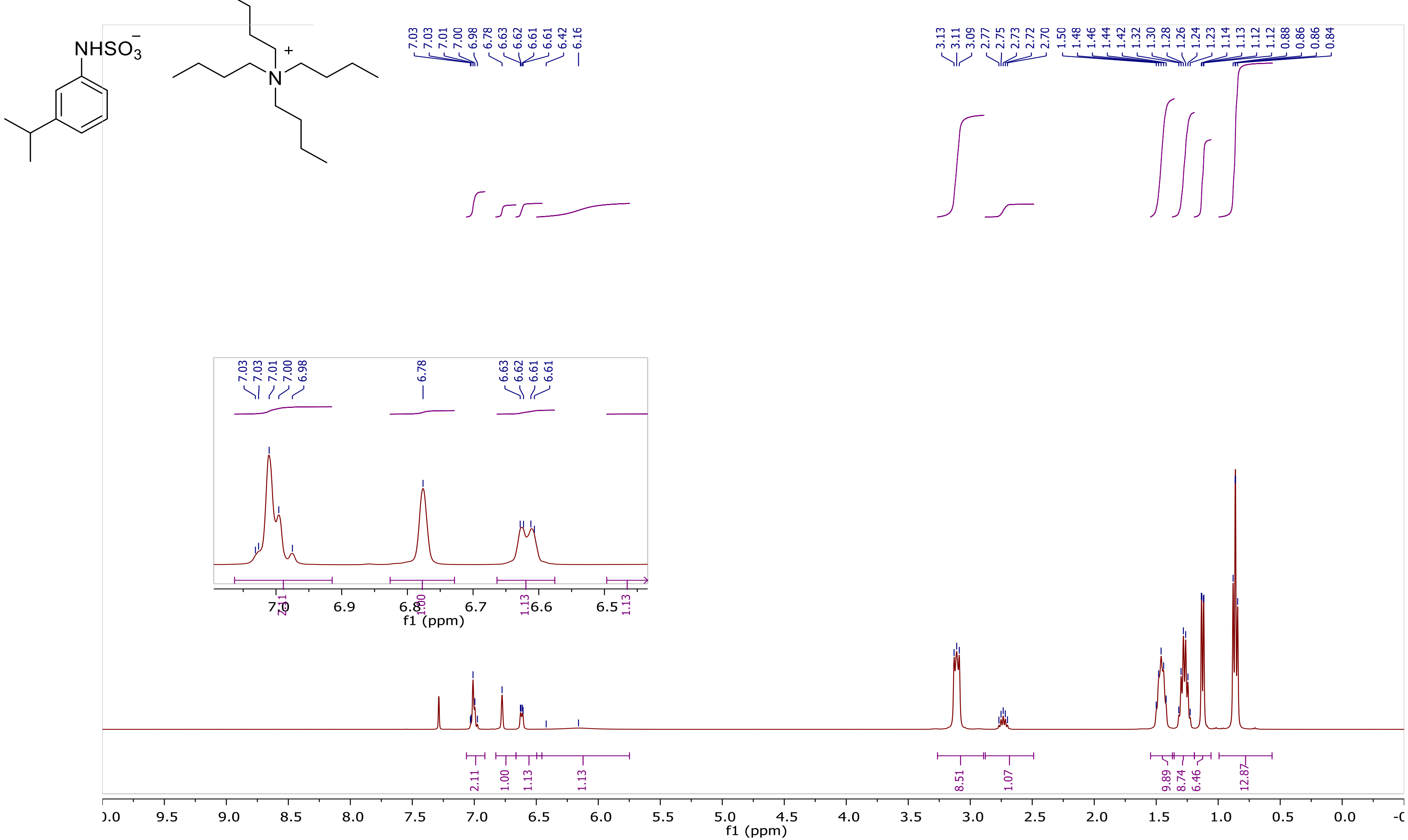

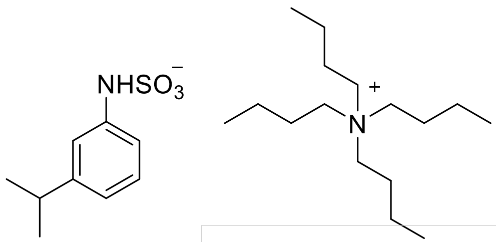

^{13}C NMR of tetrabutylammonium (3-isopropylphenyl)sulfamate **1f** in $CDCl_3$

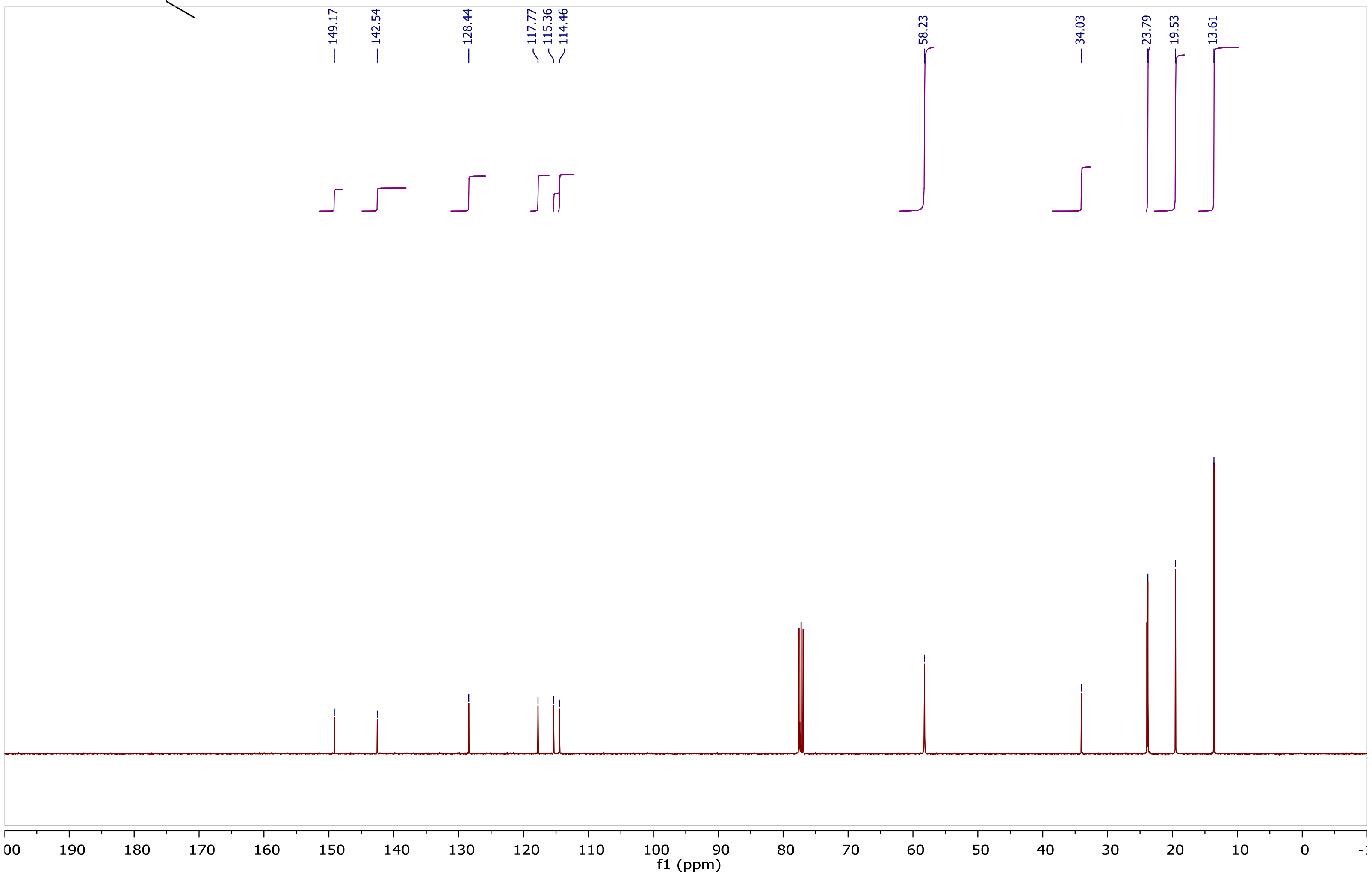

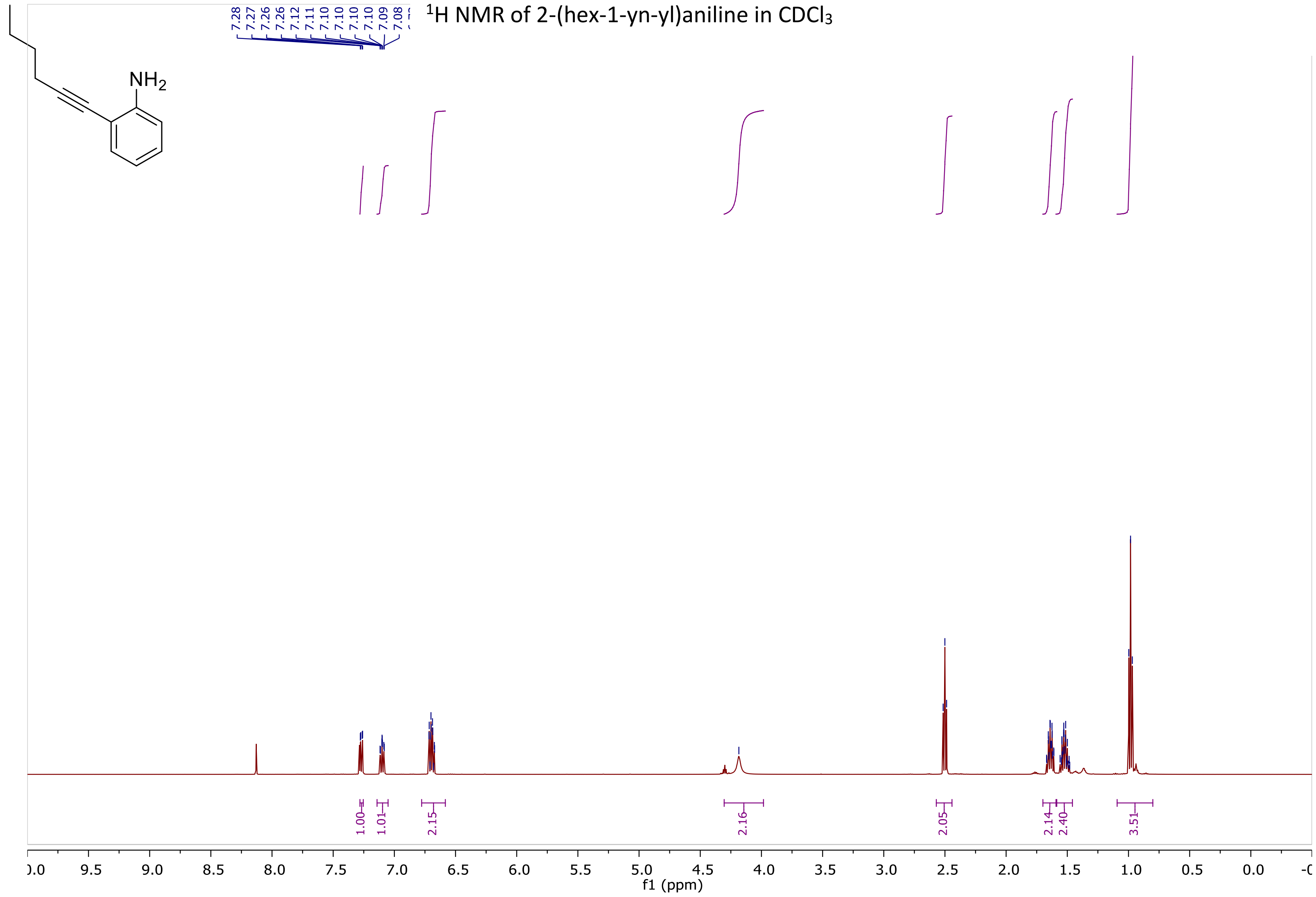

NH2
^{1}H NMR of 2-(hex-1-yn-yl)aniline in CDCl3
7.28
7.27
7.26
7.26
7.12
7.11
7.10
7.10
7.10
7.10
7.09
7.08
1.00
1.01
2.15
2.16
2.05
2.14
2.40
3.51
9.5
9.0
8.5
8.0
7.5
7.0
6.5
6.0
5.5
5.0
4.5
4.0
3.5
3.0
2.5
2.0
1.5
1.0
0.5
0.0
f1 (ppm)

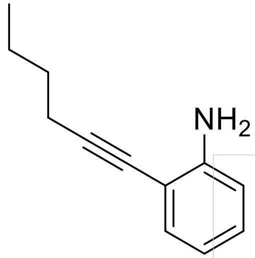

^{13}C NMR of 2-(hex-1-yn-yl)aniline in $CDCl_3$

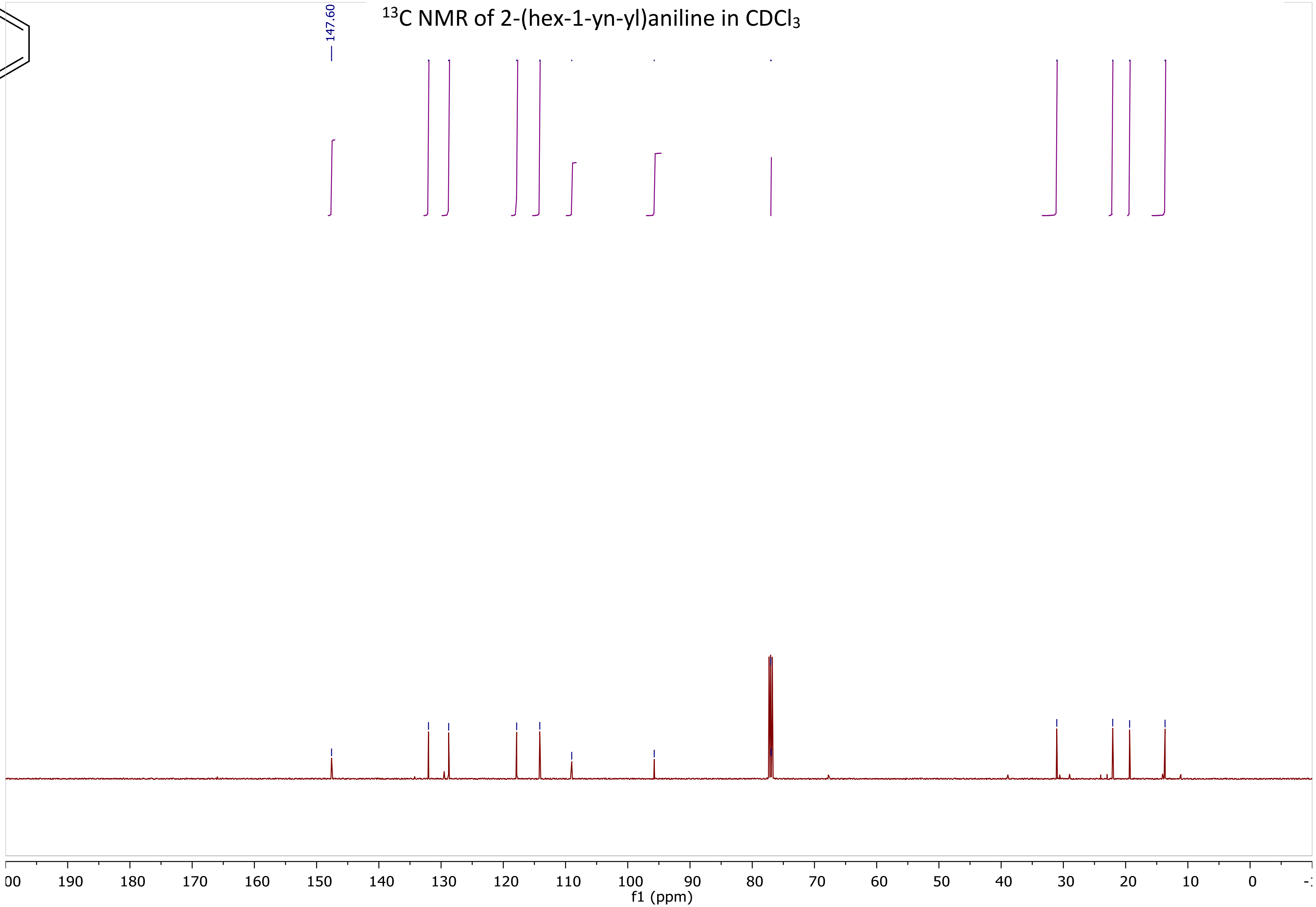

^{1}H NMR of tetrabutylammonium (2-(hex-1-yn-1-yl)phenyl)sulfamate **1g** in MeOD-d4

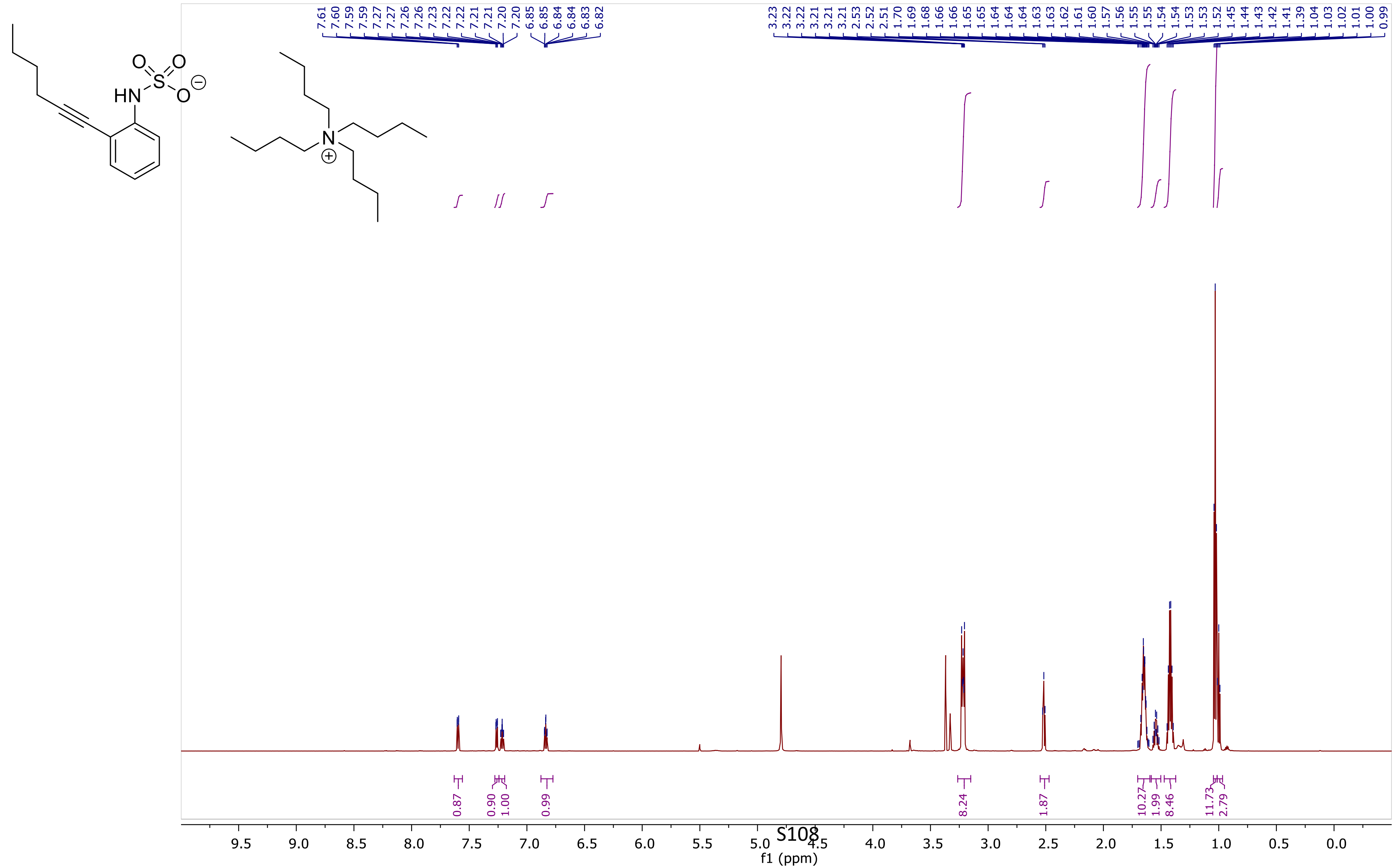

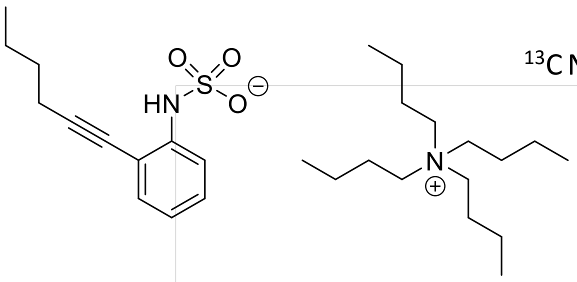

^{13}C NMR of tetrabutylammonium (2-(hex-1-yn-1-yl)phenyl)sulfamate **1g** in MeOD-d4

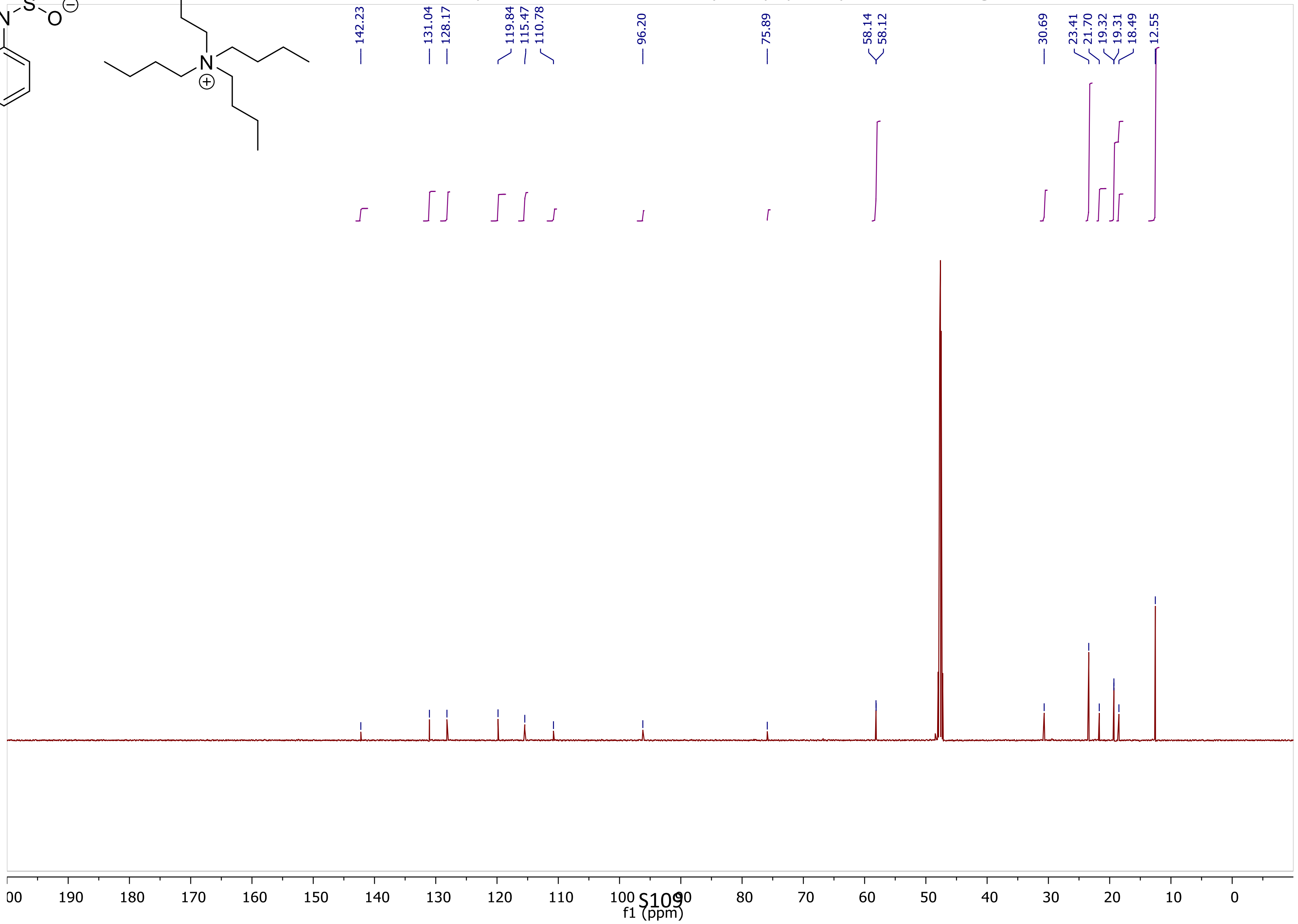

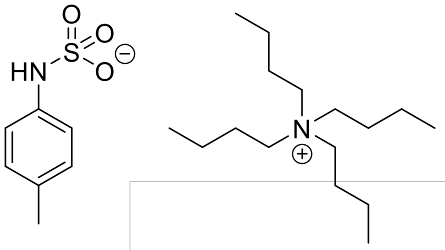

^{1}H NMR of tetrabutylammonium *p*-tolylsulfamate **1h** in $CDCl_3$

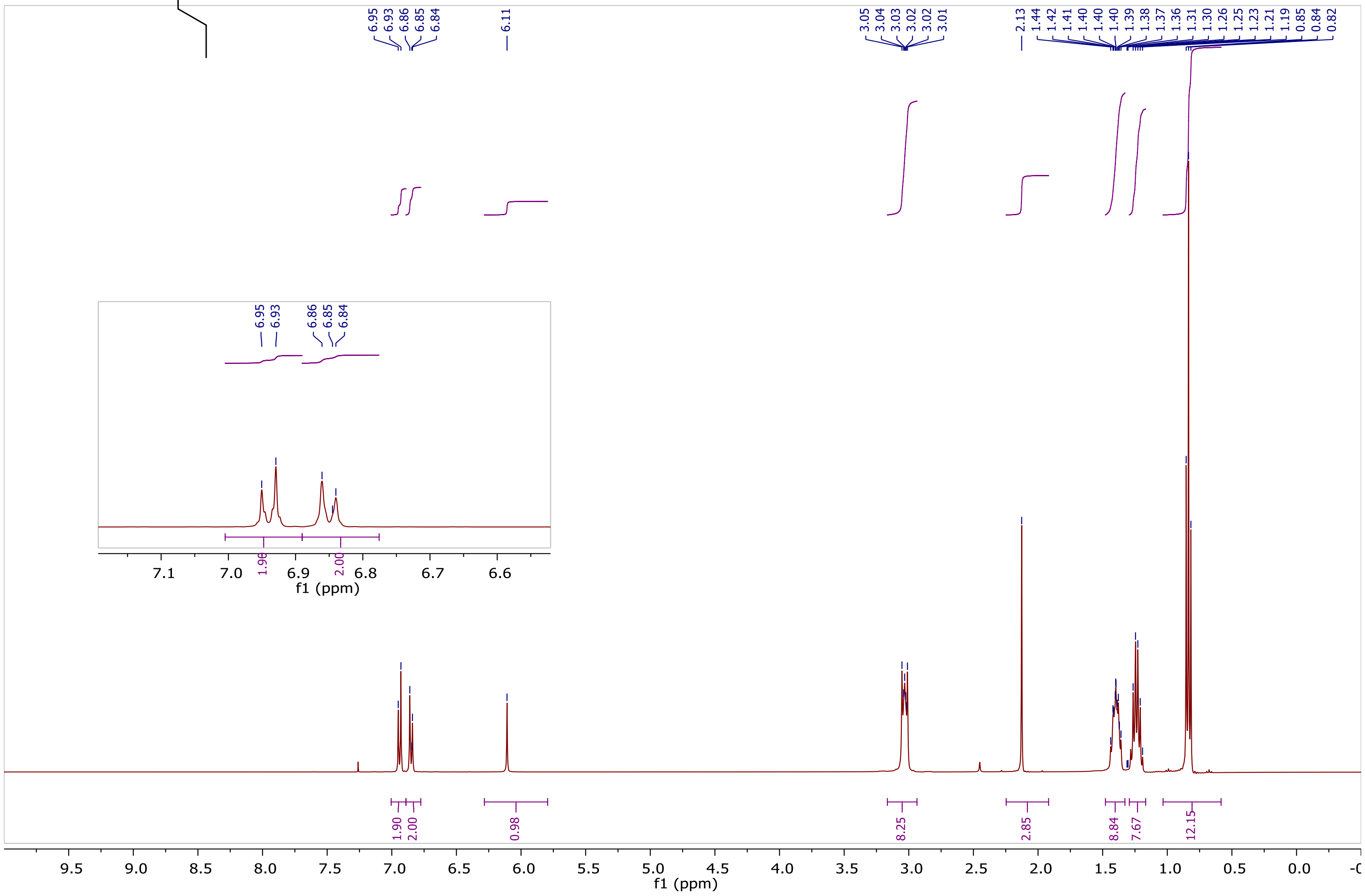

^{13}C NMR of tetrabutylammonium *p*-tolylsulfamate **1h** in $CDCl_3$

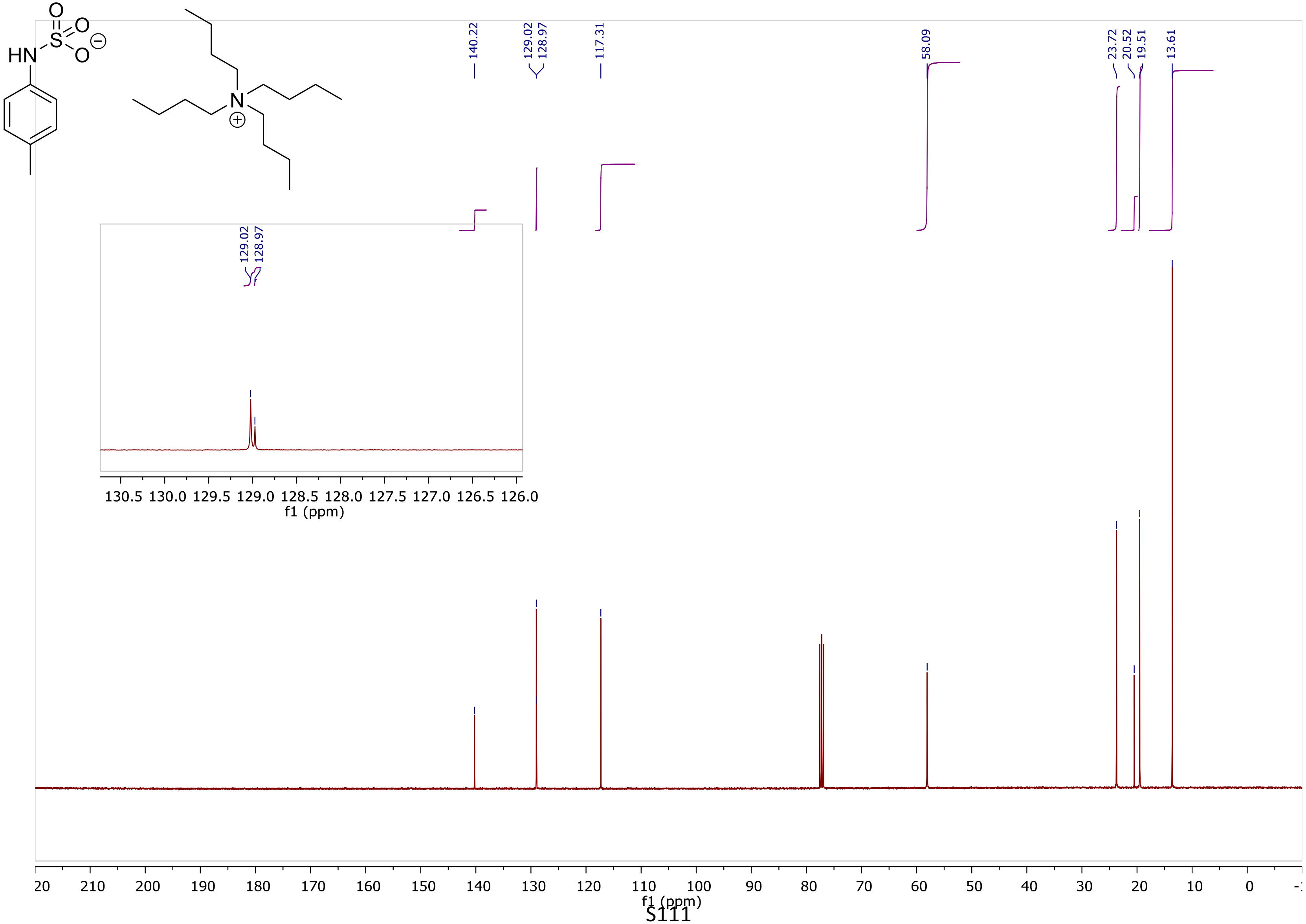

^{1}H NMR of tetrabutylammonium (5,6,7,8-tetrahydronapthalen-1-yl)sulfamate **1i** in $CDCl_3$

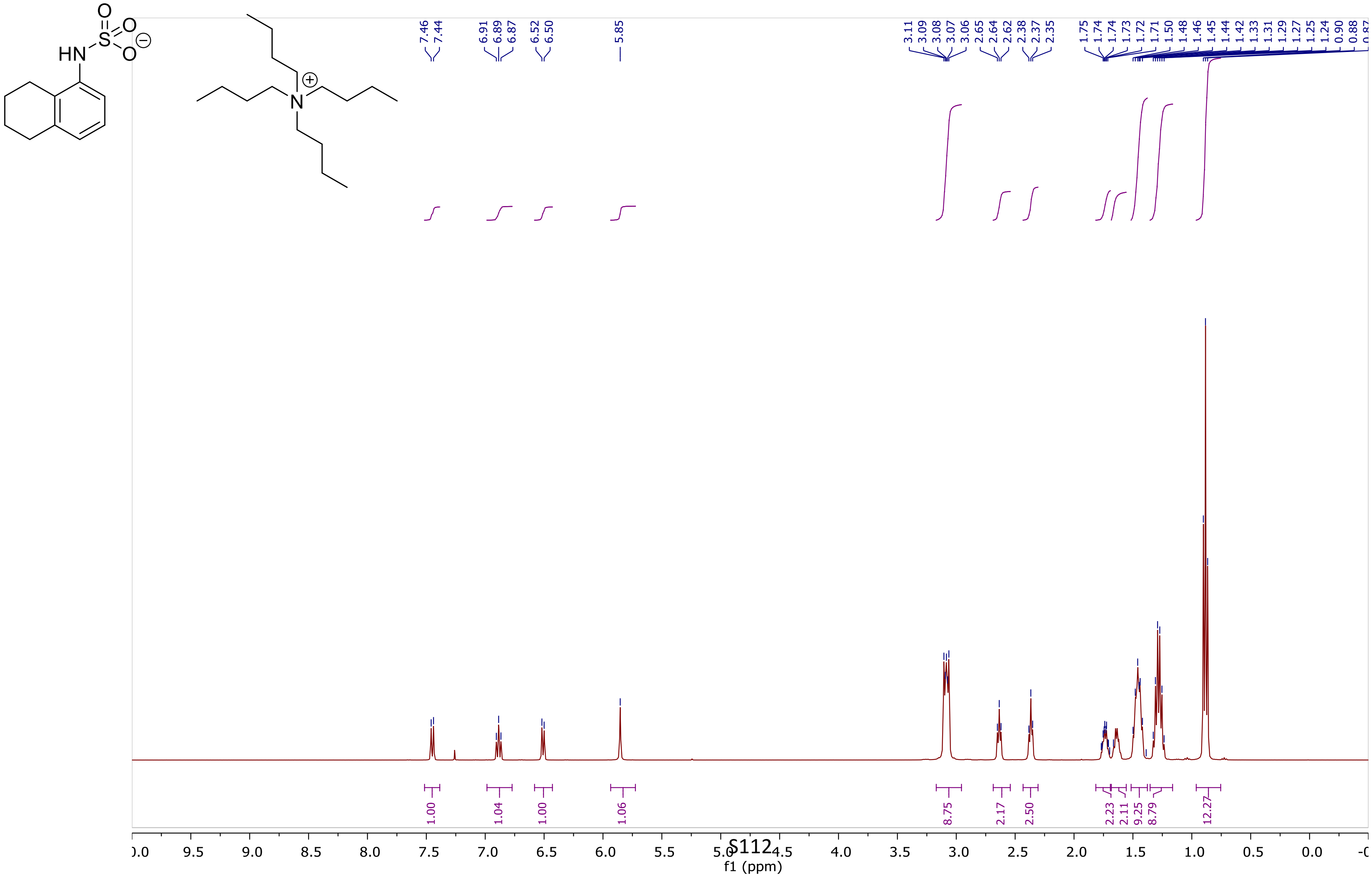

^{13}C NMR of tetrabutylammonium (5,6,7,8-tetrahydronapthalen-1-yl)sulfamate **1i** in $CDCl_3$

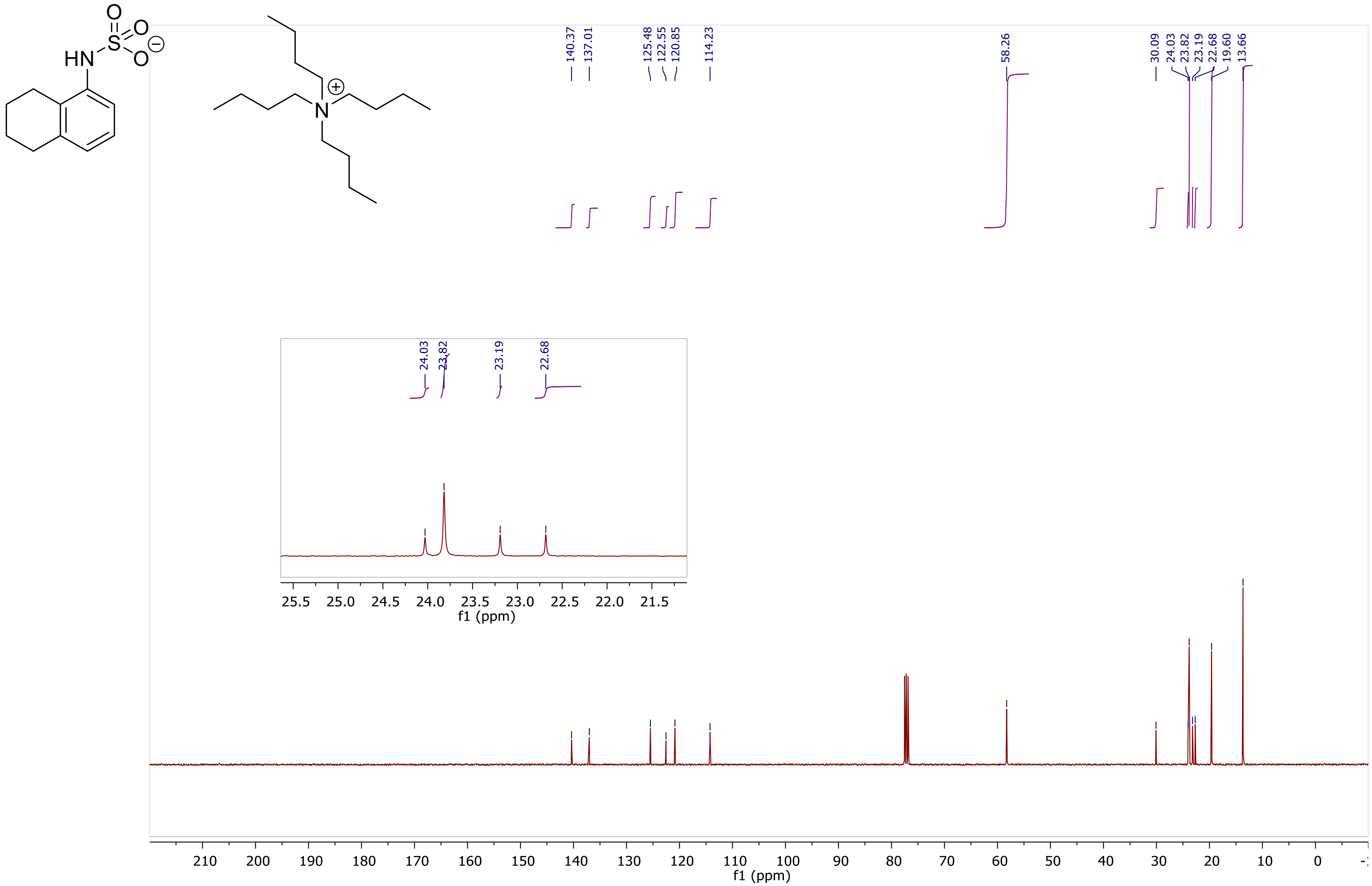

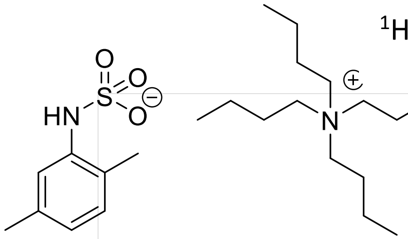

^{1}H NMR of tetrabutylammonium (2,5-dimethylphenyl)sulfamate **1j** in $CDCl_3$

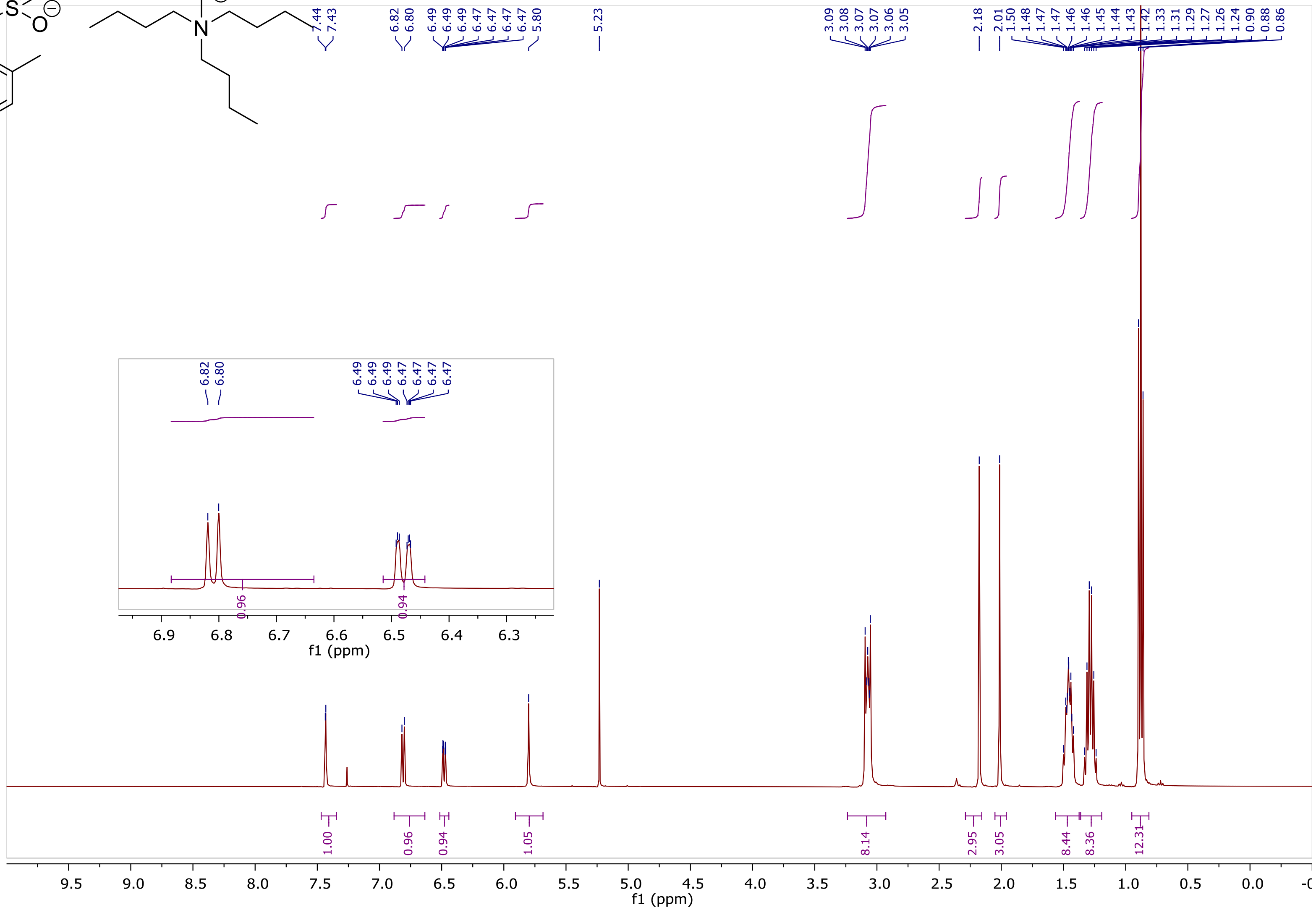

^{13}C NMR of tetrabutylammonium (2,5-dimethylphenyl)sulfamate **1j** in $CDCl_3$

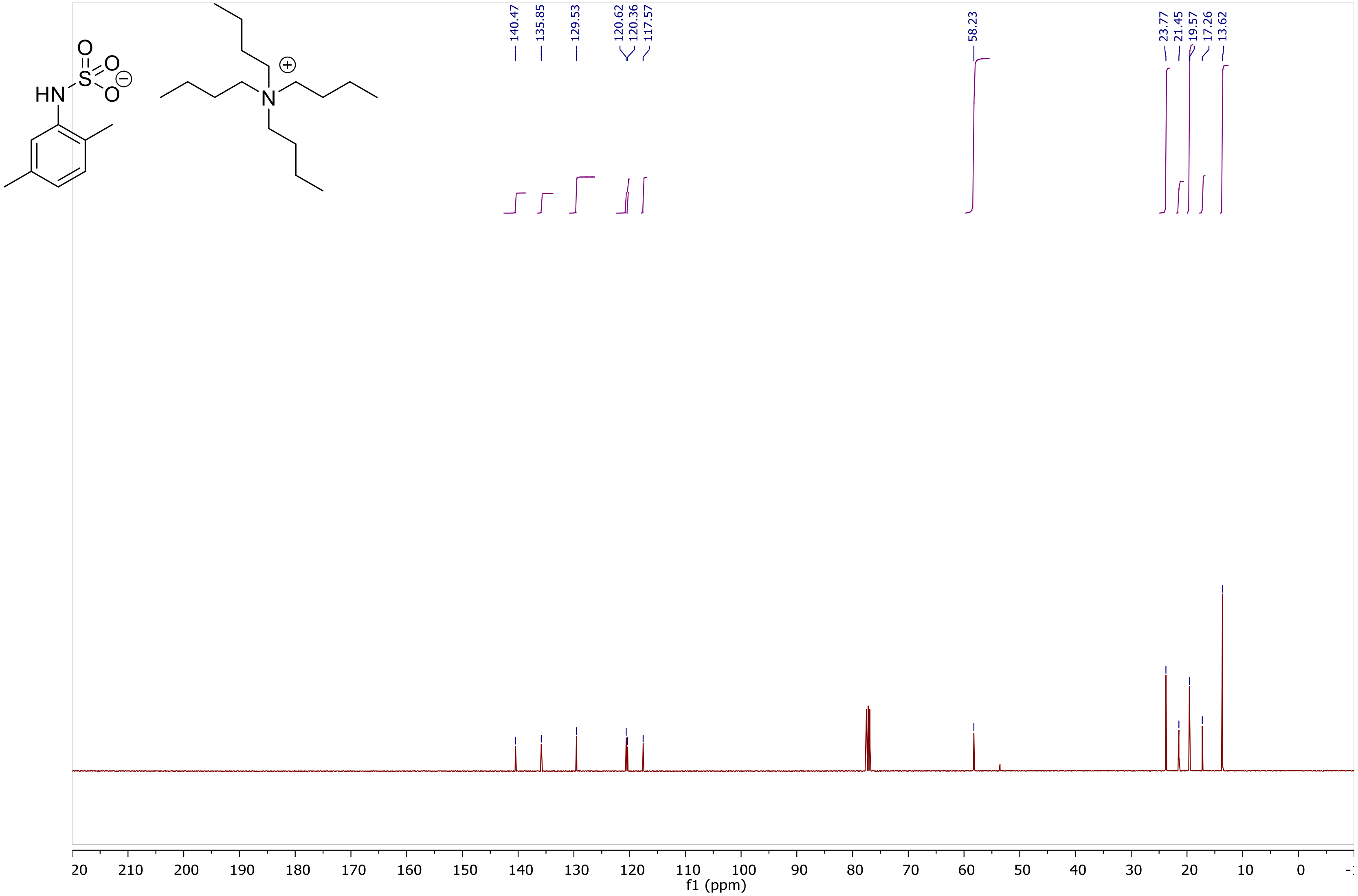

^{1}H NMR of tetrabutylammonium (2,3-dimethylphenyl)sulfamate **1k** in $CDCl_3$

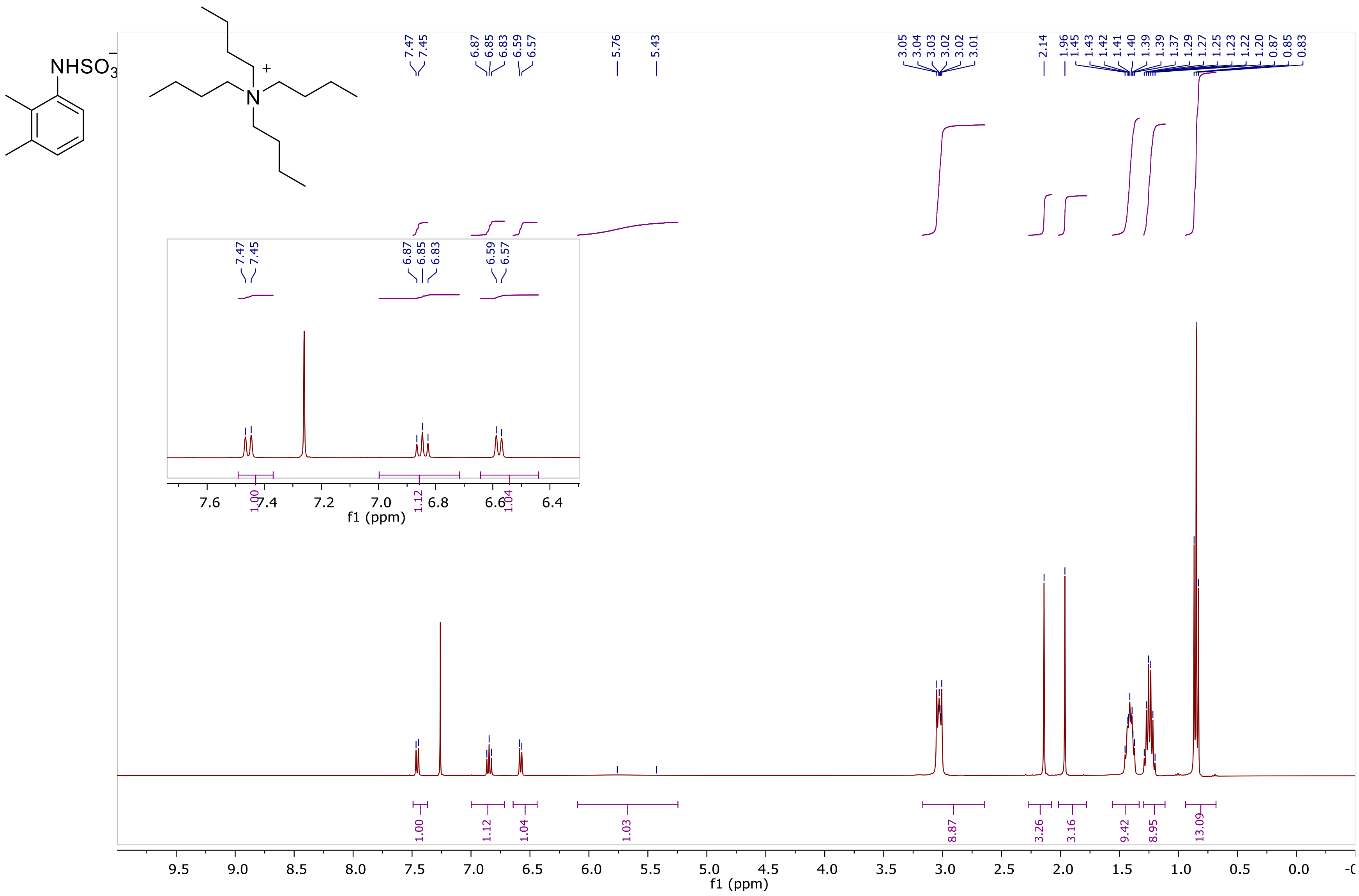

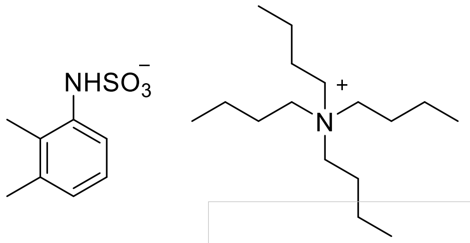

^{13}C NMR of tetrabutylammonium (2,3-dimethylphenyl)sulfamate **1k** in $CDCl_3$

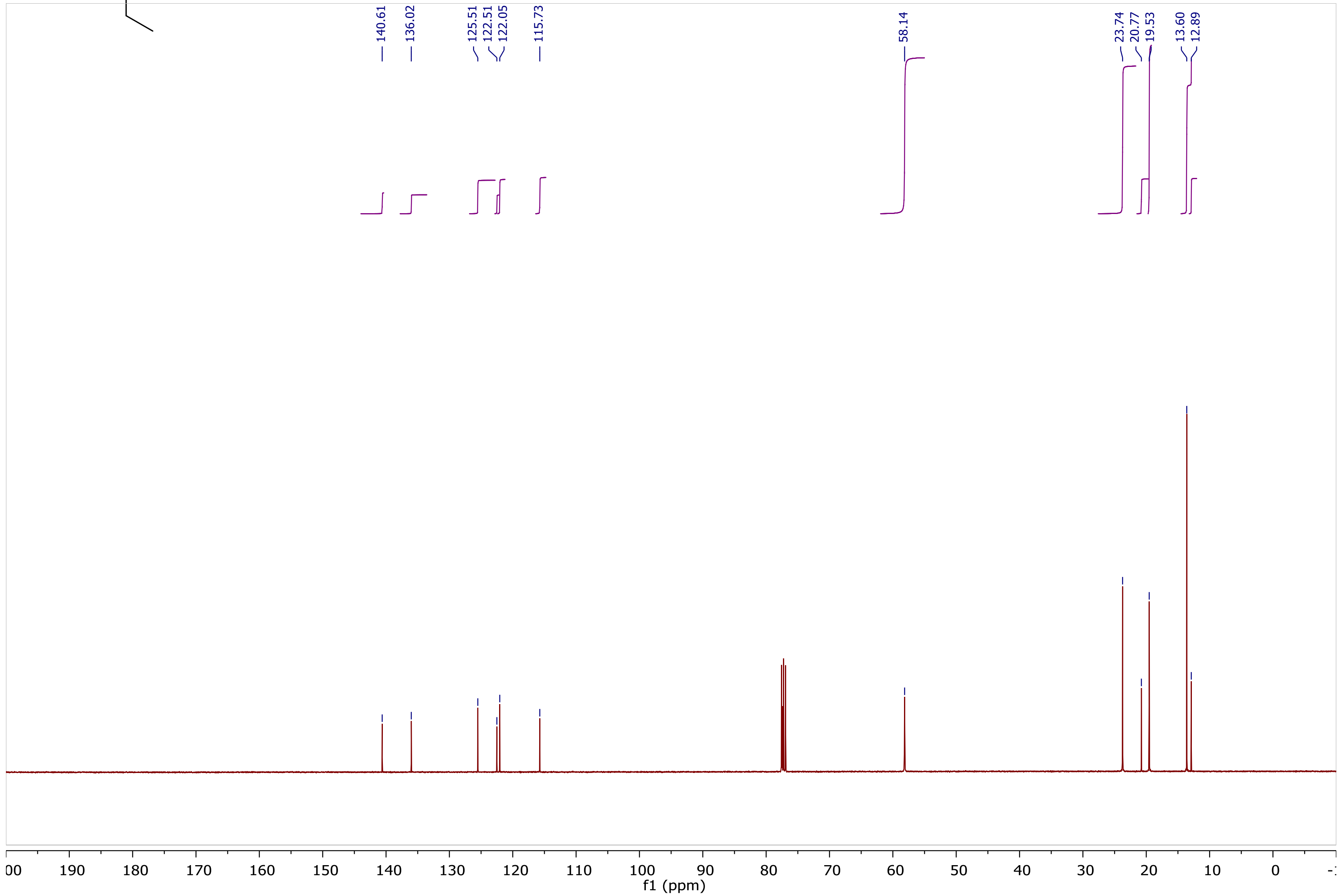

^{1}H NMR of tetrabutylammonium (3-methoxyphenyl)sulfamate **1l** in $CDCl_3$

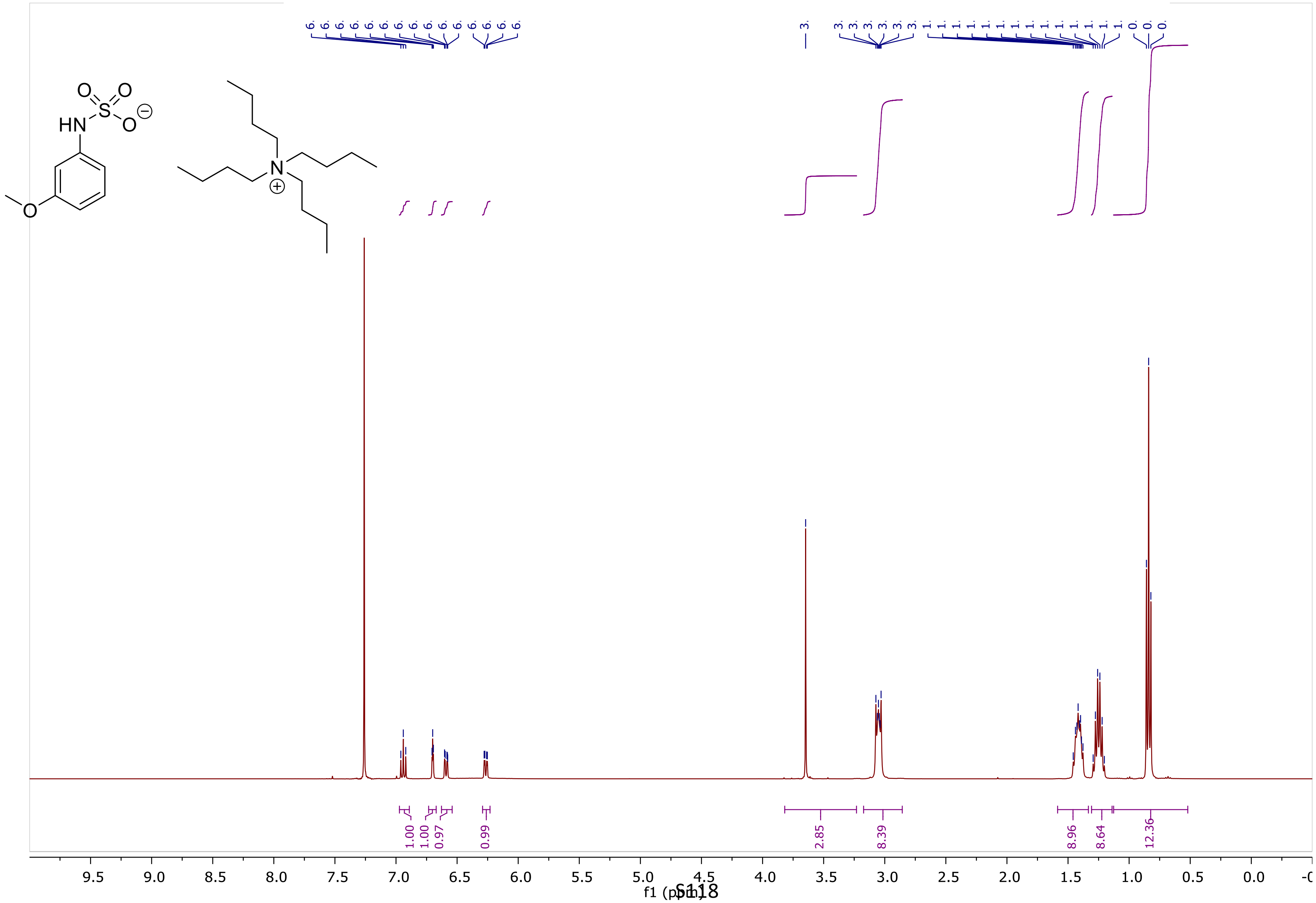

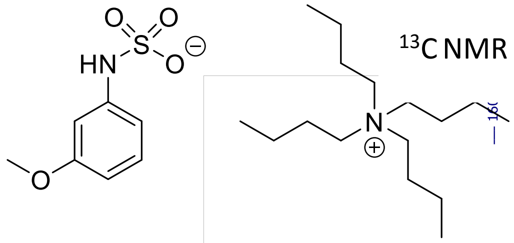

^{13}C NMR of tetrabutylammonium (3-methoxyphenyl)sulfamate **1l** in $CDCl_3$

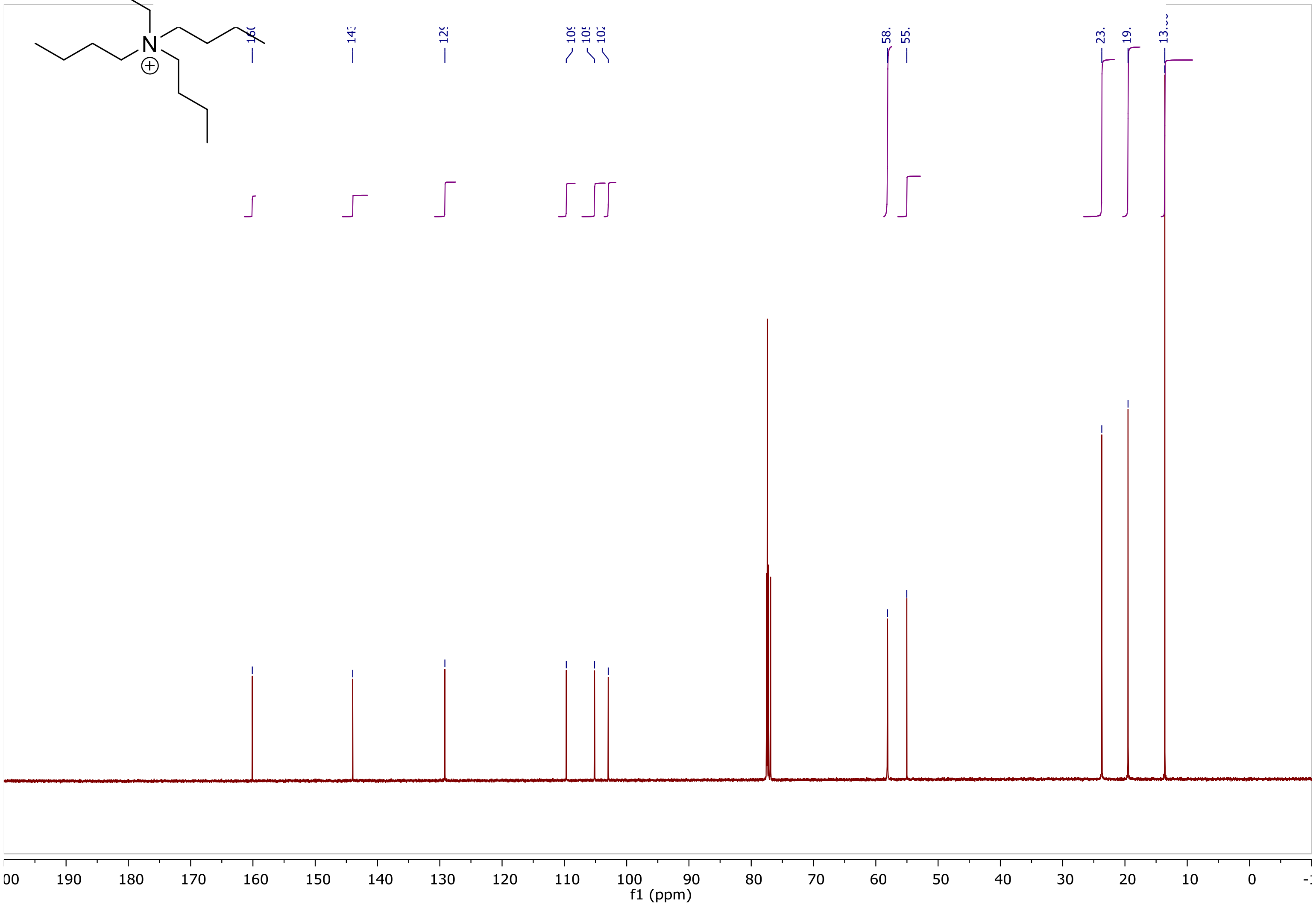

^{1}H NMR of tetrabutylammonium (3-methoxy-2-methylphenyl)sulfamate **1m** in $CDCl_3$

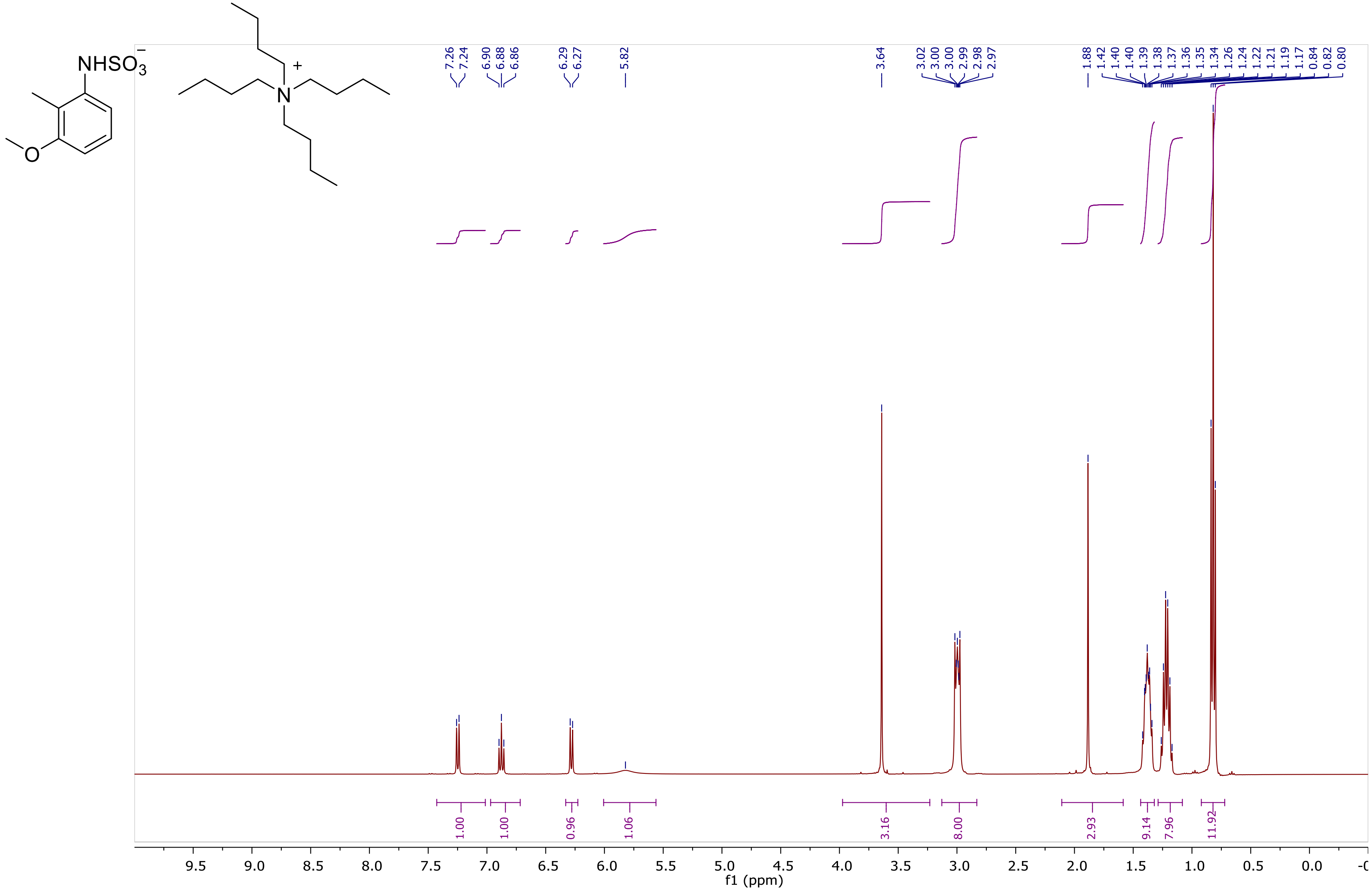

^{13}C NMR of tetrabutylammonium (3-methoxy-2-methylphenyl)sulfamate **1m** in $CDCl_3$

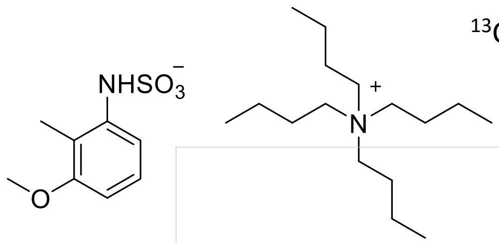

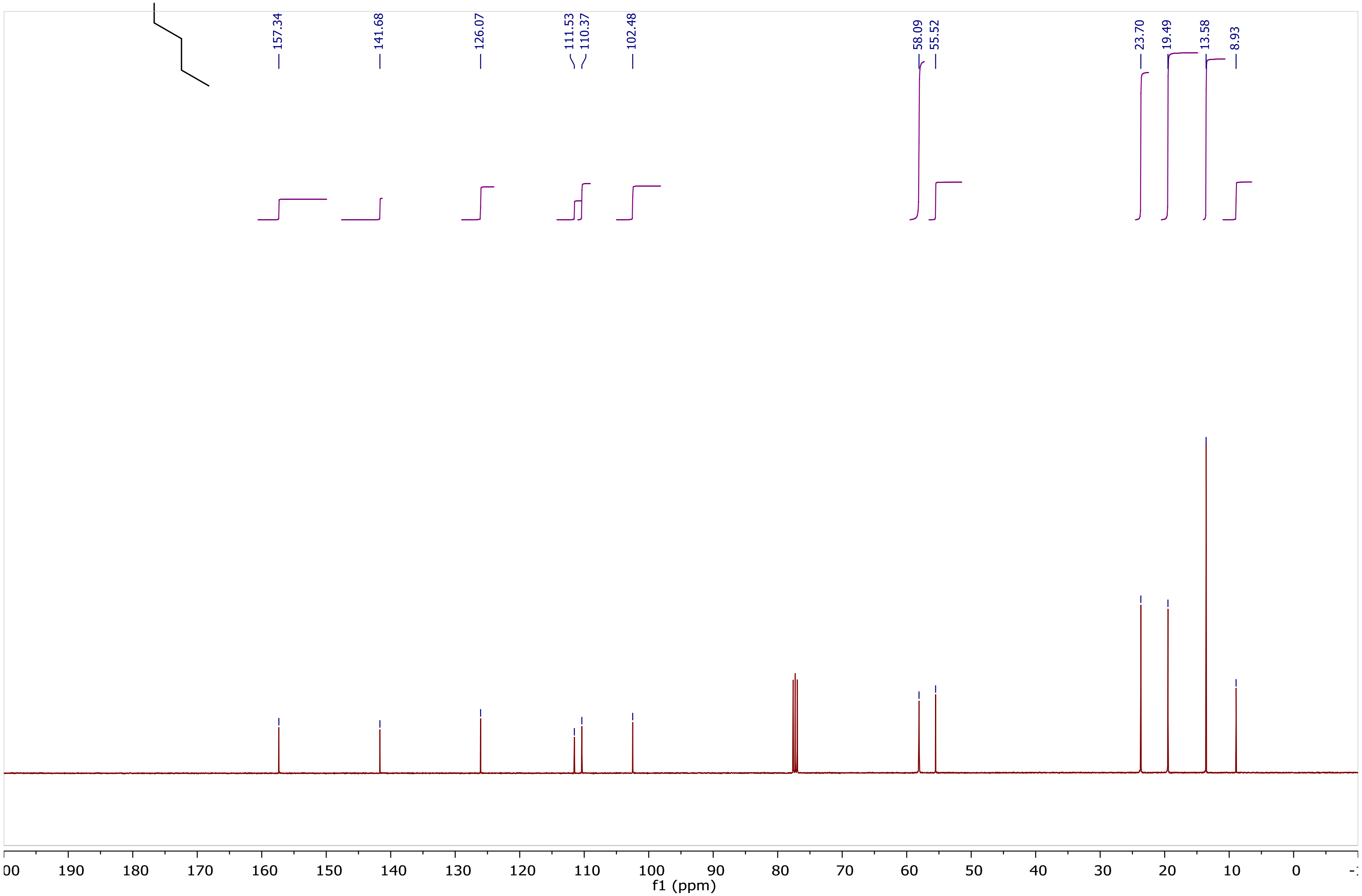

^1H NMR of tetrabutylammonium (3-difluoromethoxyphenyl)sulfamate **1n** in $CDCl_3$

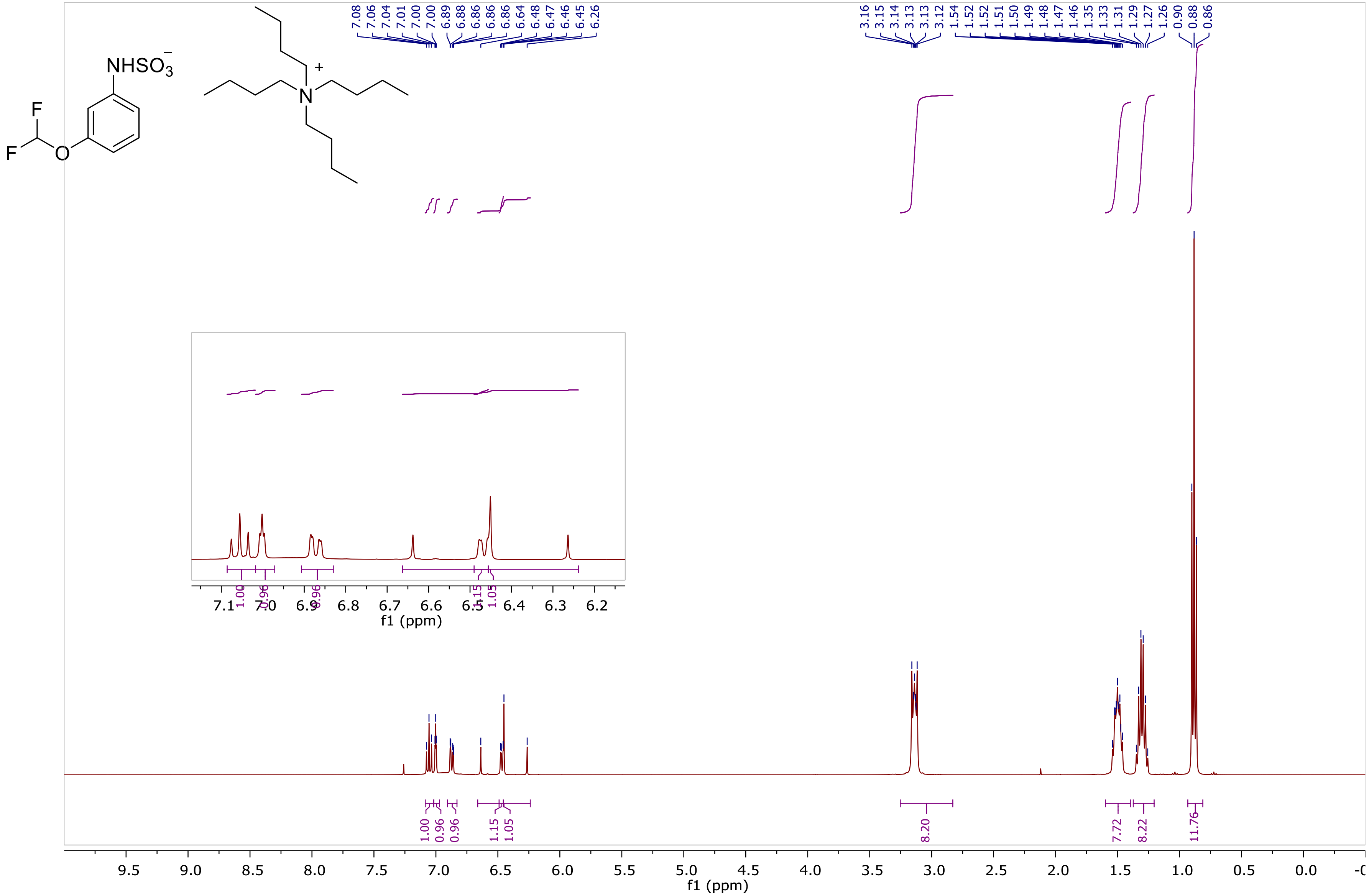

^{13}C NMR of tetrabutylammonium (3-difluoromethoxyphenyl)sulfamate **1n** in $CDCl_3$

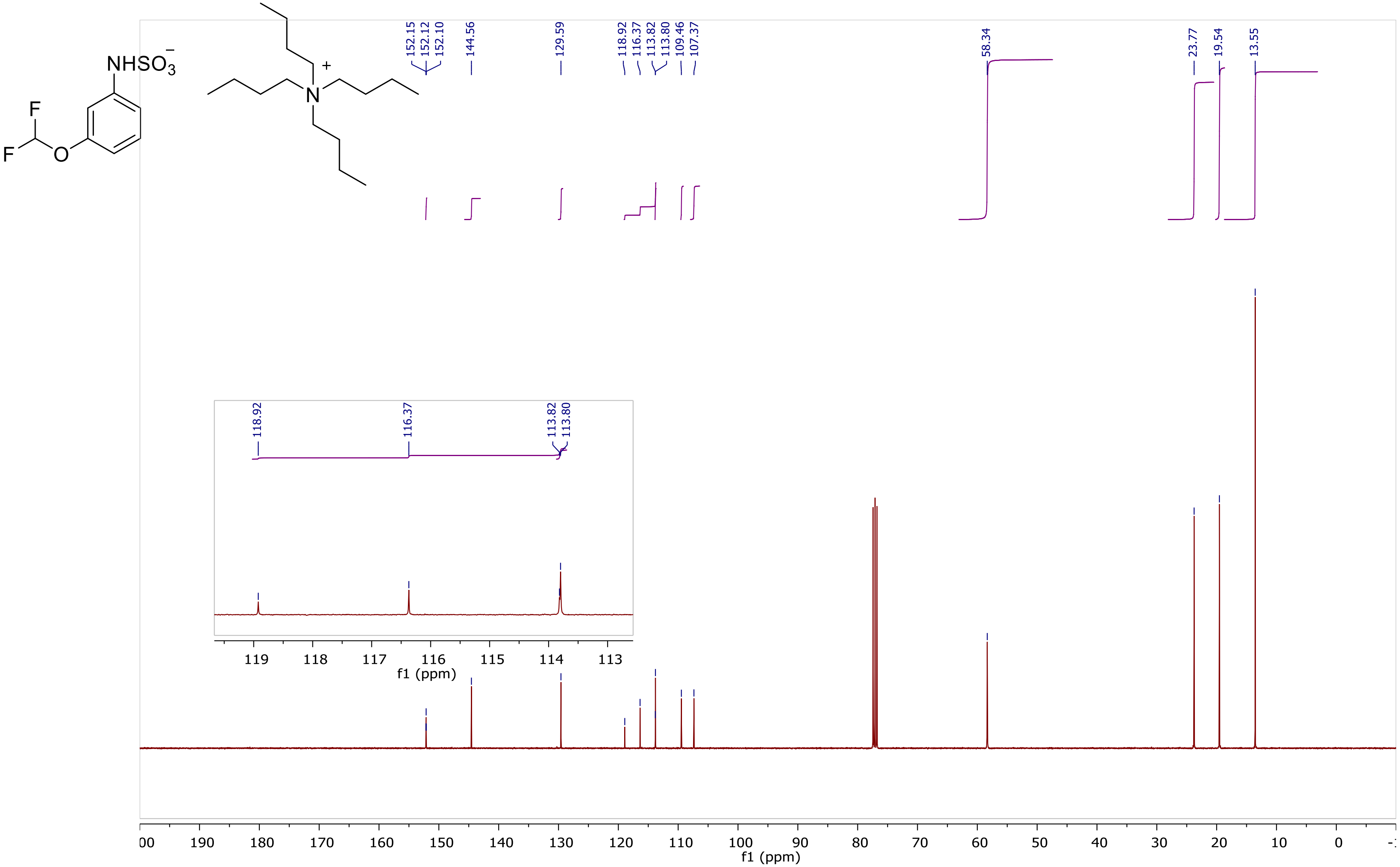

^{19}F NMR of tetrabutylammonium (3-difluoromethoxyphenyl)sulfamate **1n** in $CDCl_3$

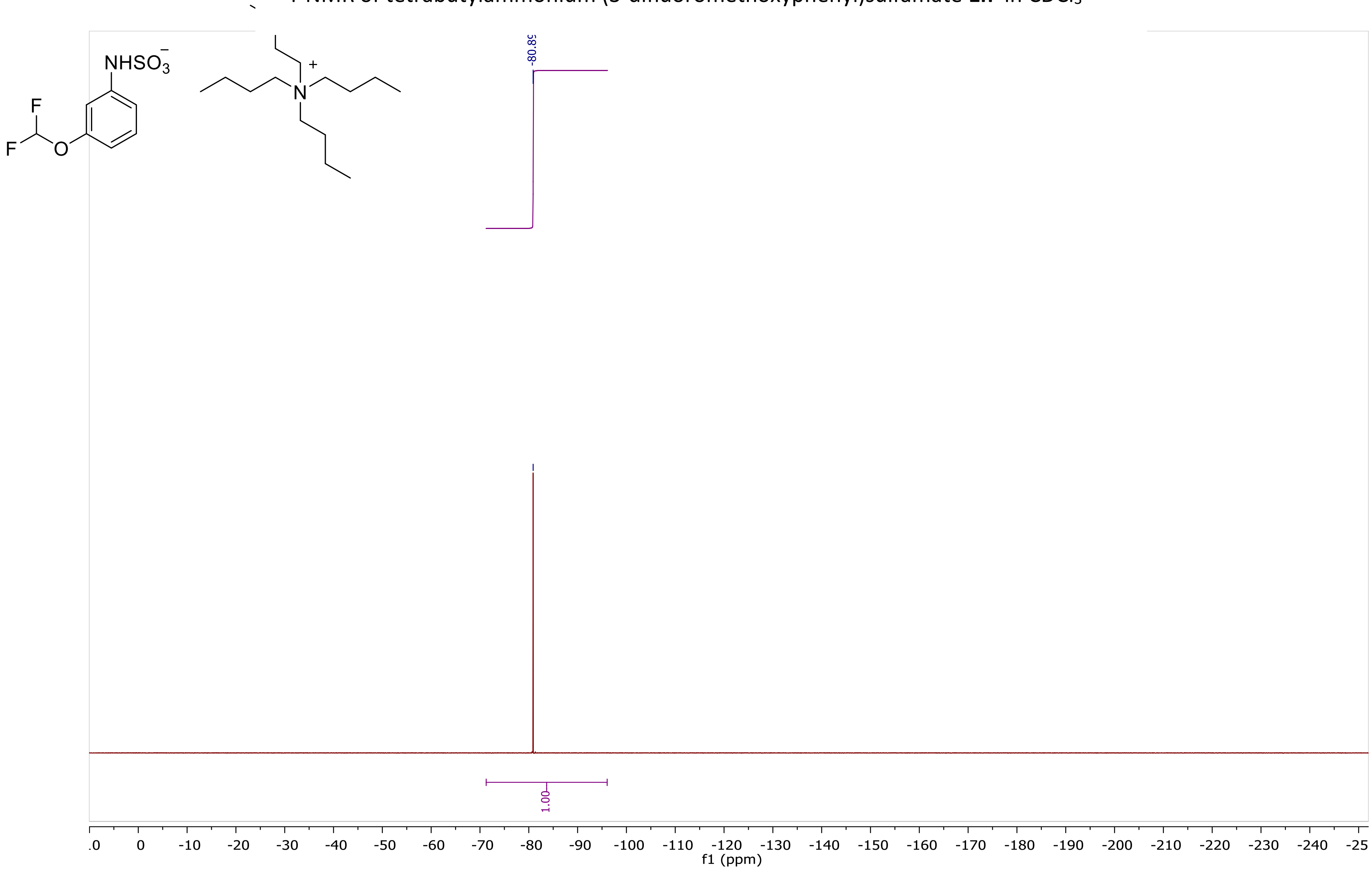

^{1}H NMR of tetrabutylammonium (5-bromo-2-methylphenyl)sulfamate **1o** in $CDCl_3$

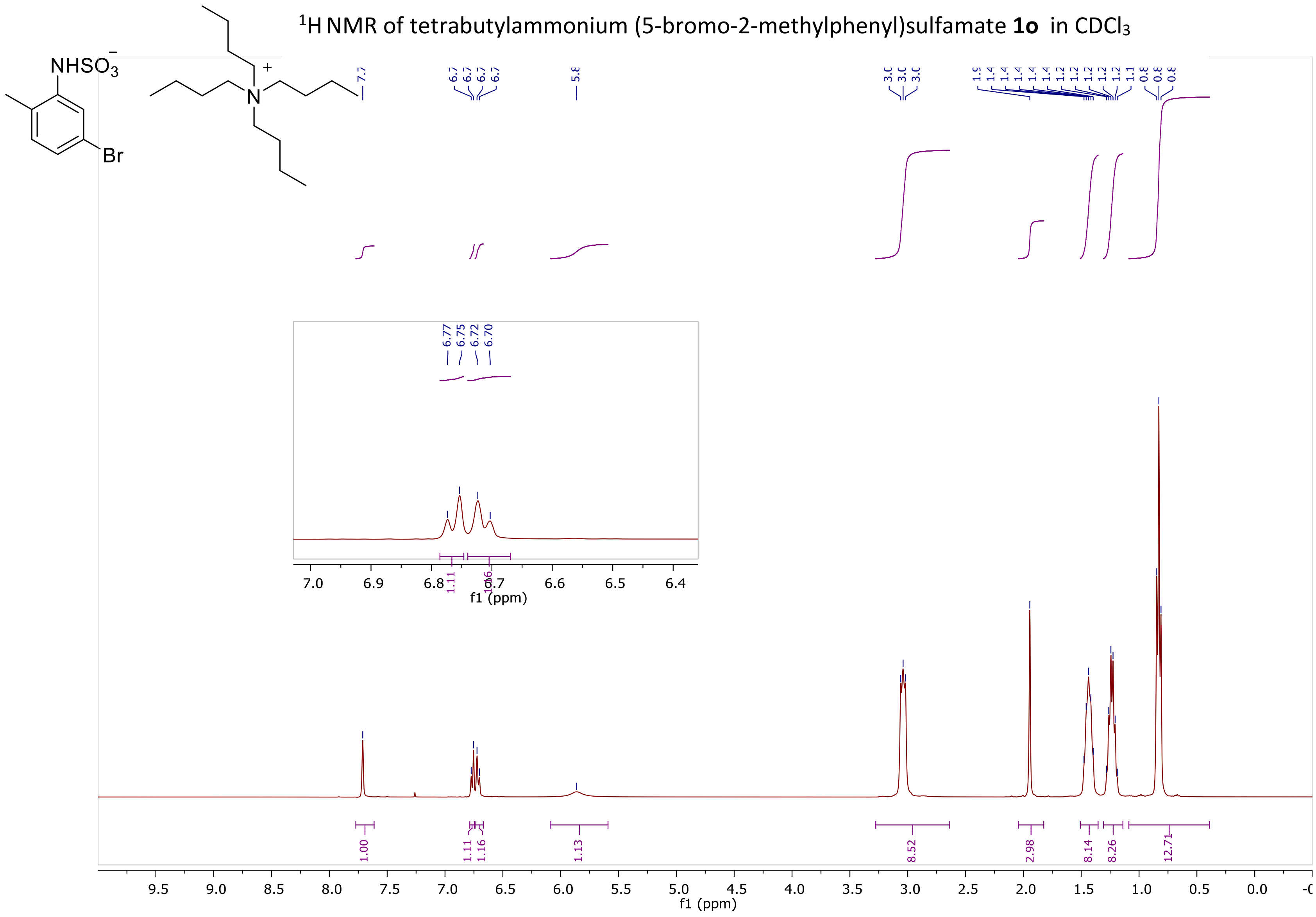

^{13}C NMR of tetrabutylammonium (5-bromo-2-methylphenyl)sulfamate **1o** in $CDCl_3$

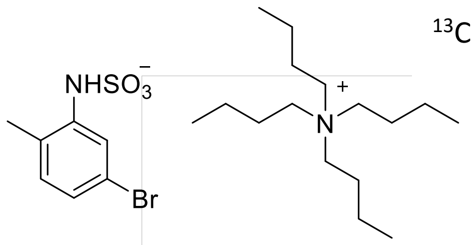

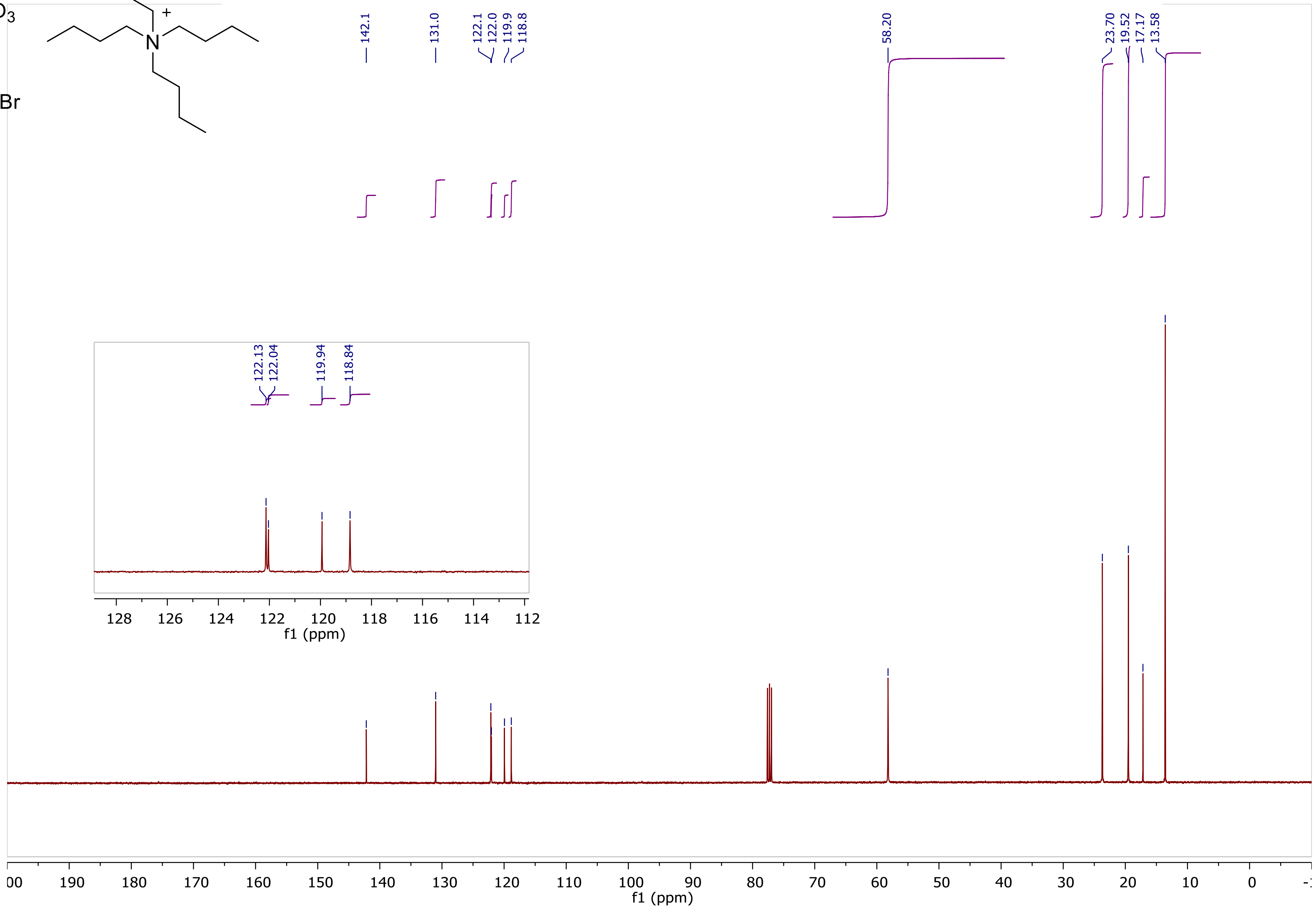

^{1}H NMR of tetrabutylammonium (3-chloro-2-methylphenyl)sulfamate **1p** in $CDCl_3$

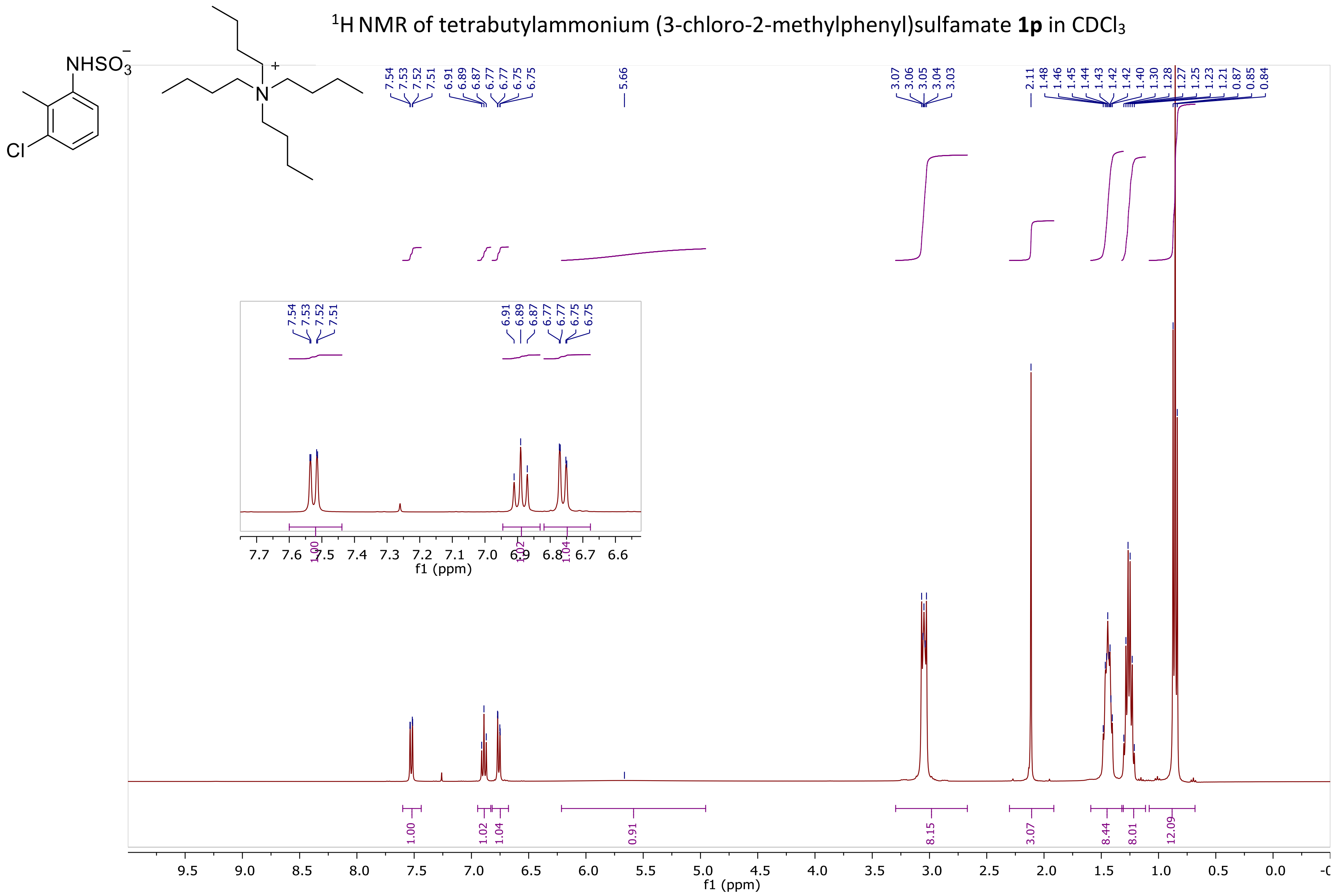

^{13}C NMR of tetrabutylammonium (3-chloro-2-methylphenyl)sulfamate **1p** in $CDCl_3$

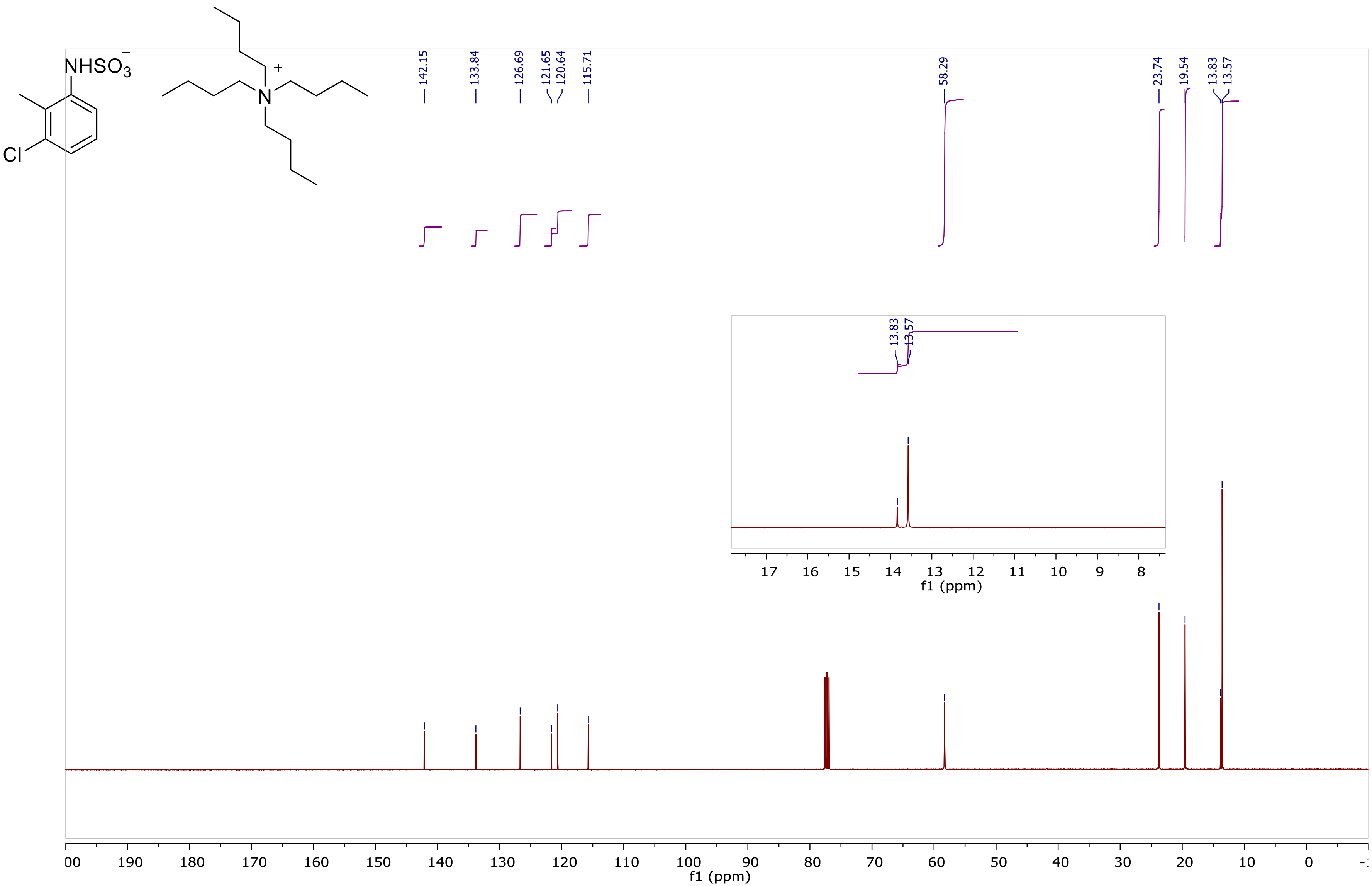

^{1}H NMR of tetrabutylammonium (3-fluoro-2-methylphenyl)sulfamate **1q** in $CDCl_3$

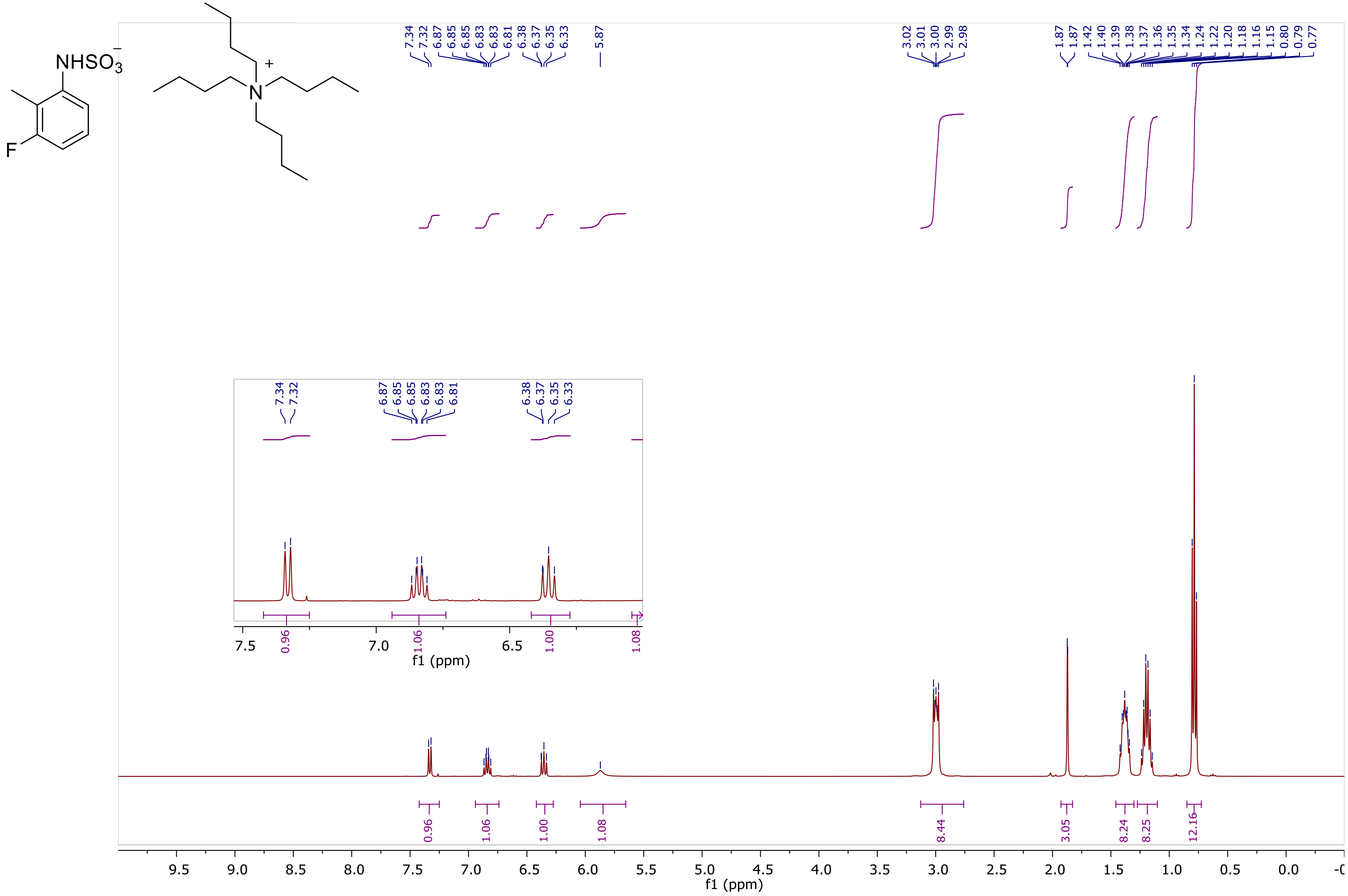

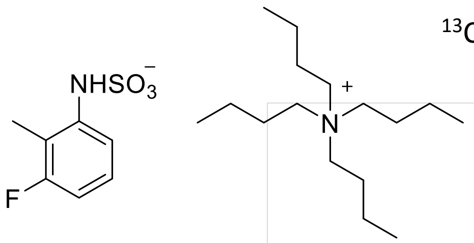

^{13}C NMR of tetrabutylammonium (3-fluoro-2-methylphenyl)sulfamate **1q** in $CDCl_3$

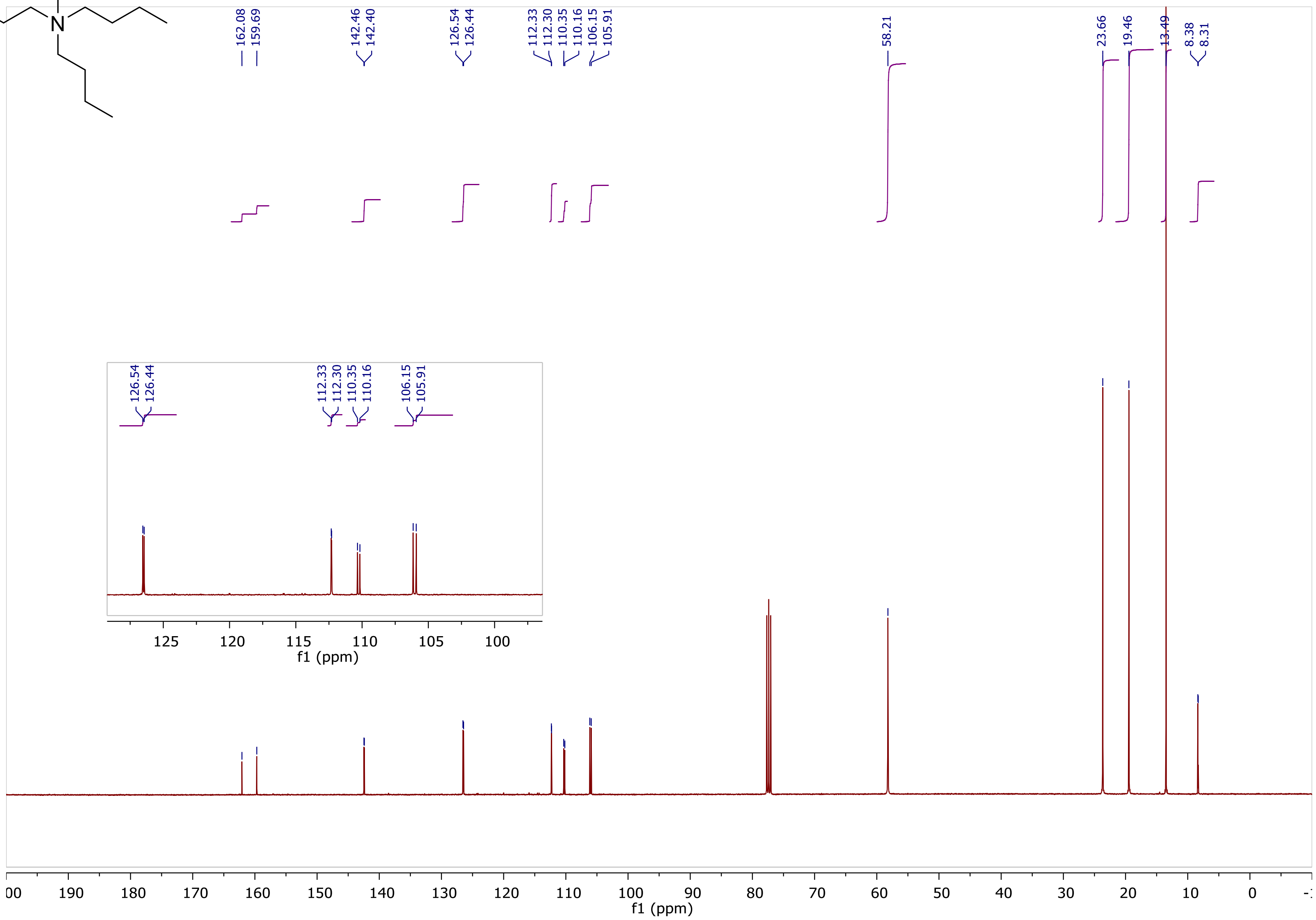

^{19}F NMR of tetrabutylammonium (3-fluoro-2-methylphenyl)sulfamate **1q** in $CDCl_3$

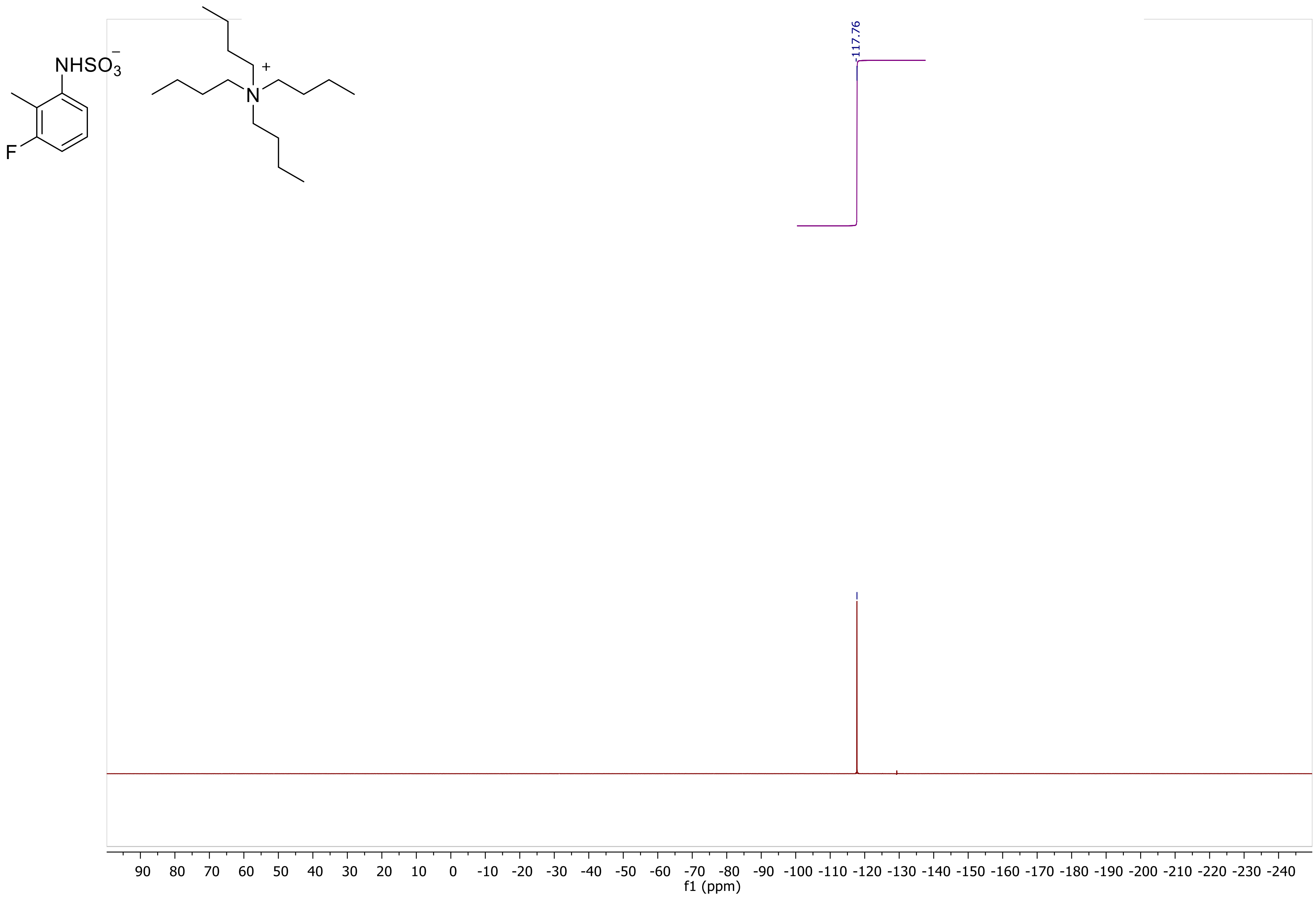

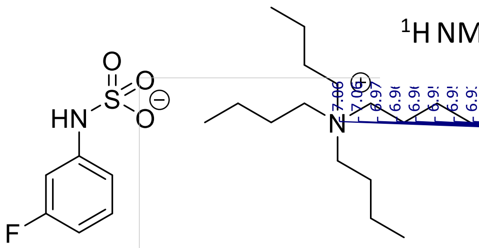

^{1}H NMR of tetrabutylammonium (3-fluorophenyl)sulfamate **1r** in $CDCl_3$

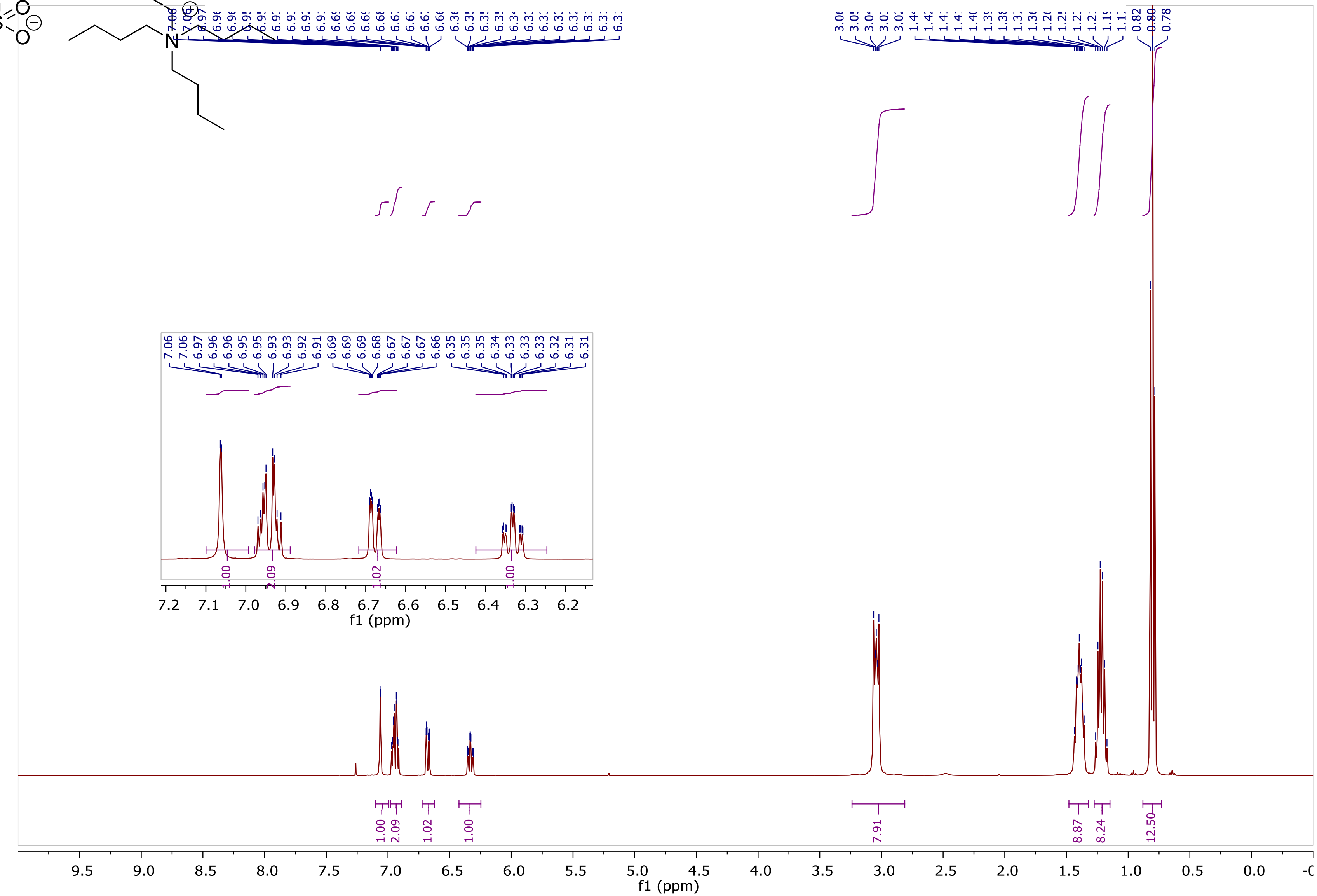

^{13}C NMR of tetrabutylammonium (3-fluorophenyl)sulfamate **1r** in $CDCl_3$

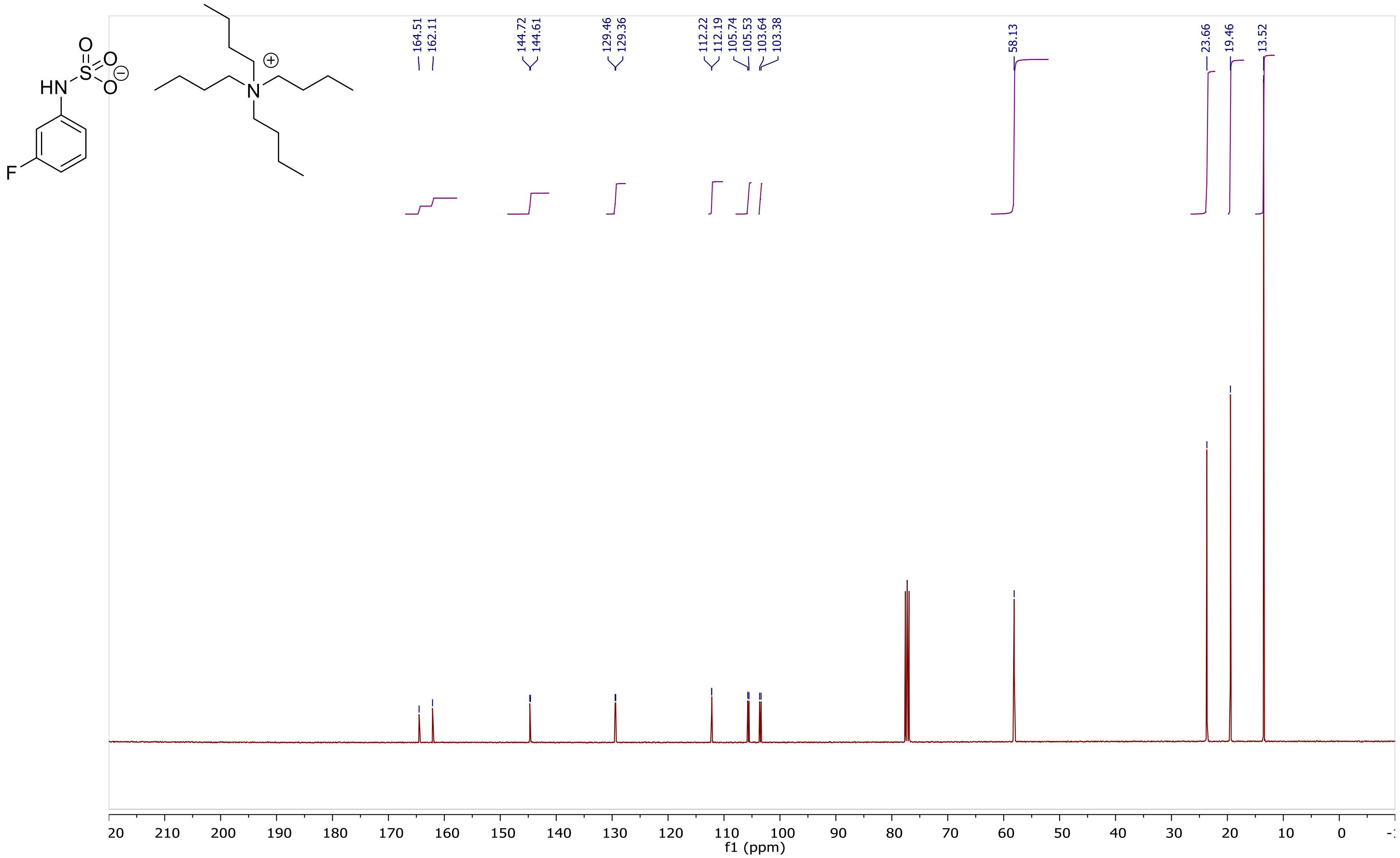

^{19}F NMR of tetrabutylammonium (3-fluorophenyl)sulfamate **1r** in $CDCl_3$

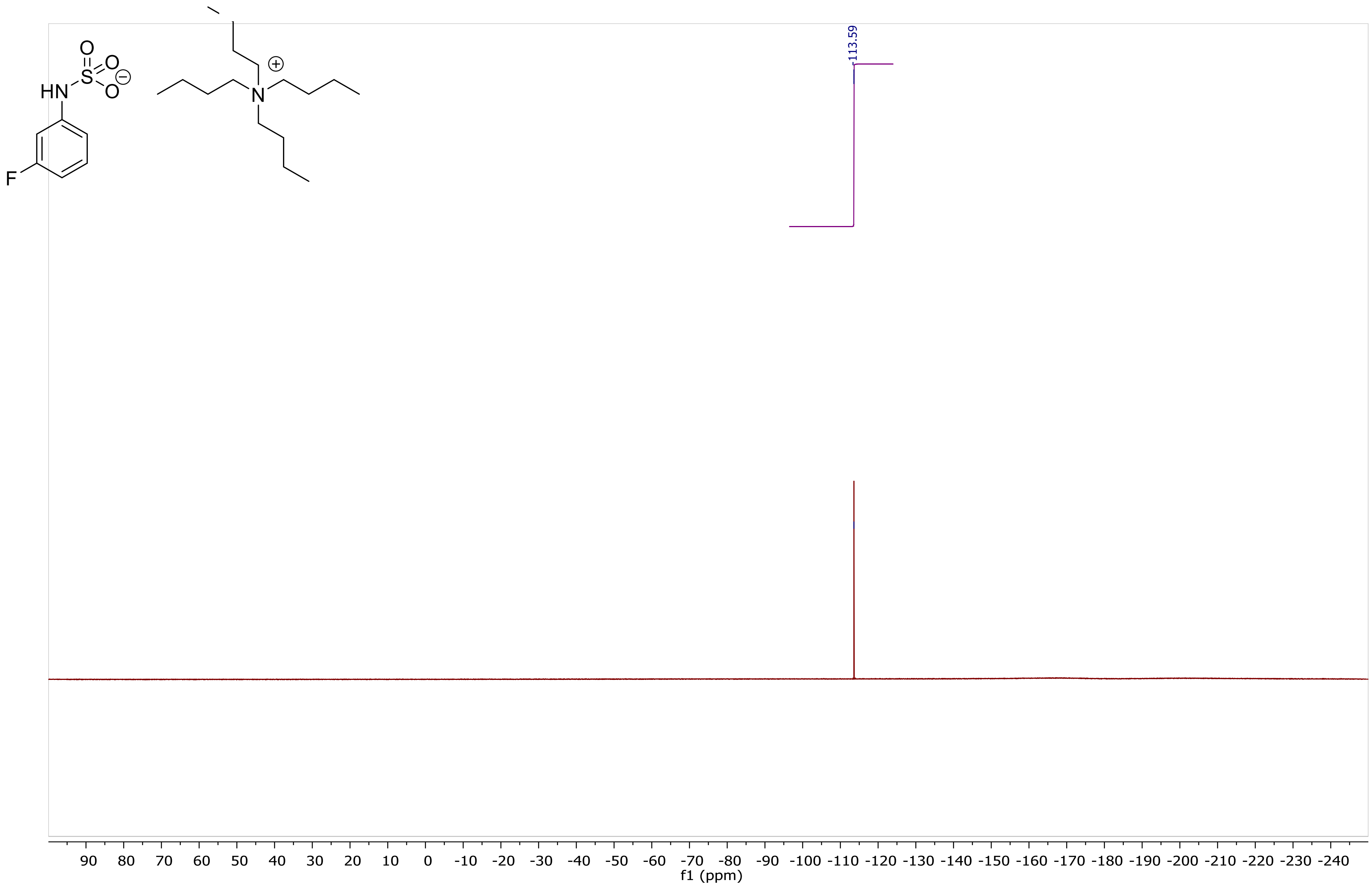

^{1}H NMR of tetrabutylammonium (2-bromo-3-methylphenyl)sulfamate **1s** in $CDCl_3$

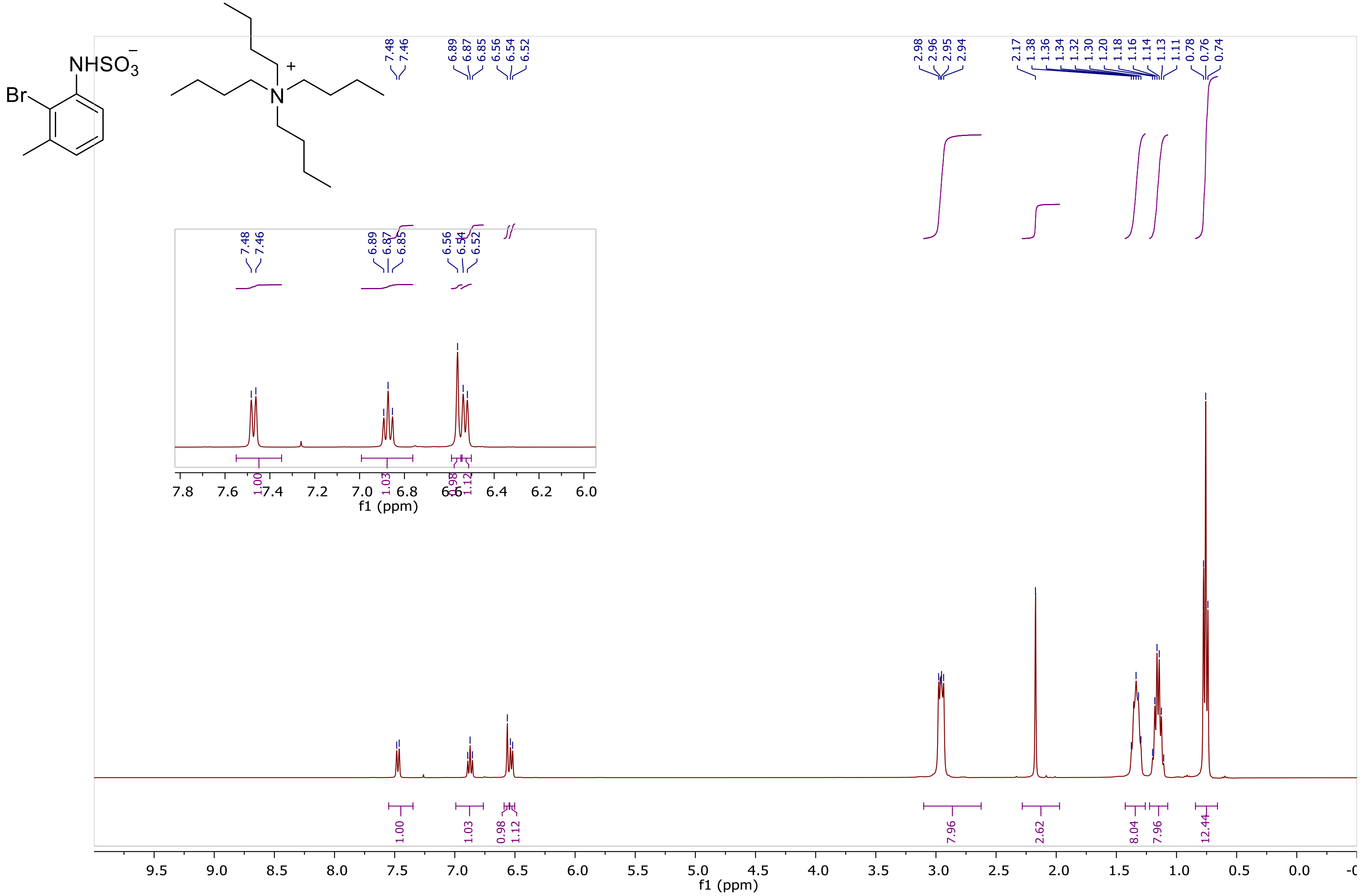

^{13}C NMR of tetrabutylammonium (2-bromo-3-methylphenyl)sulfamate **1s** in $CDCl_3$

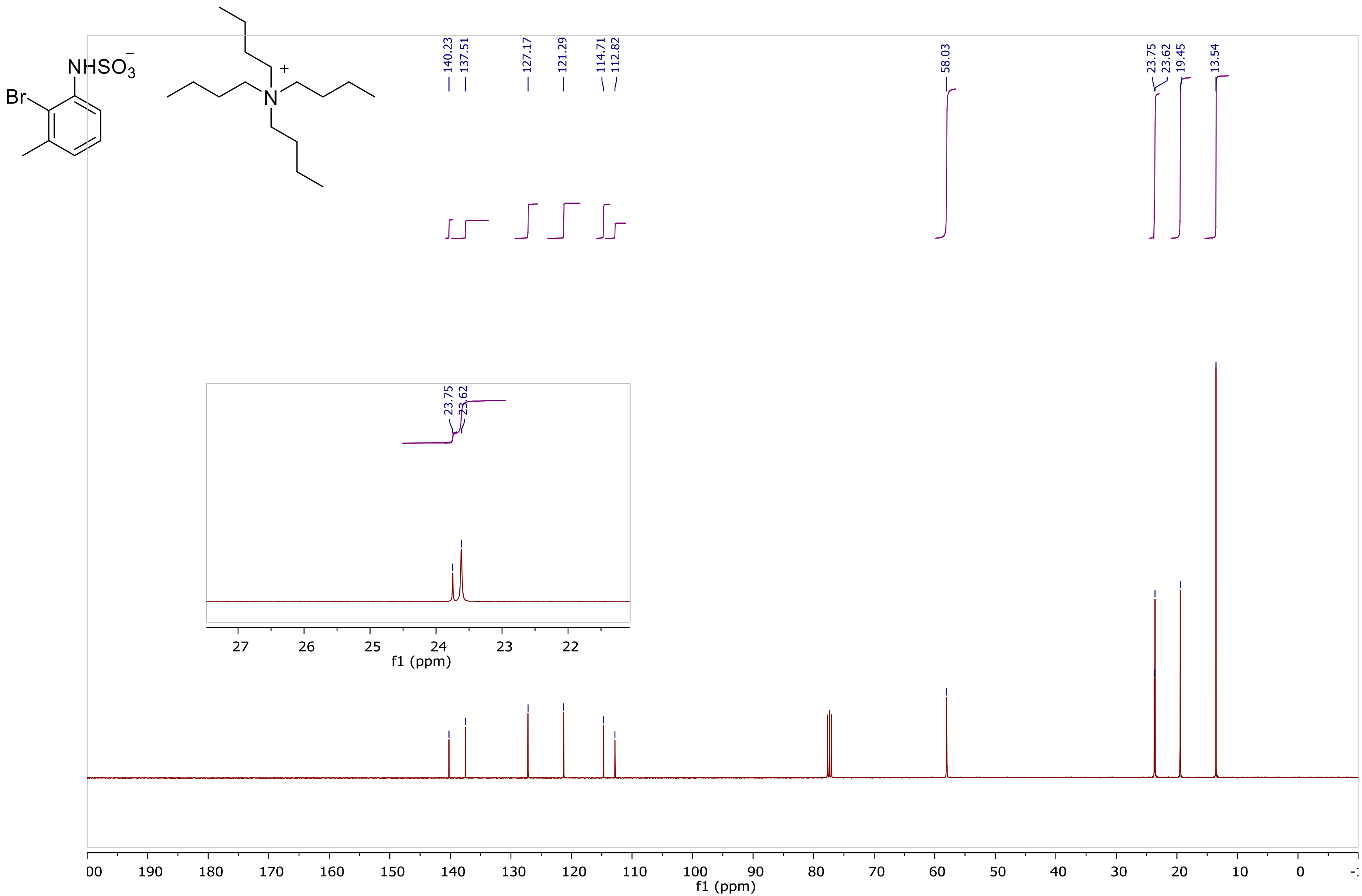

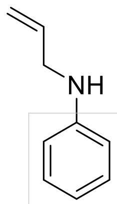

^{1}H NMR of *N*-allylaniline in $CDCl_3$

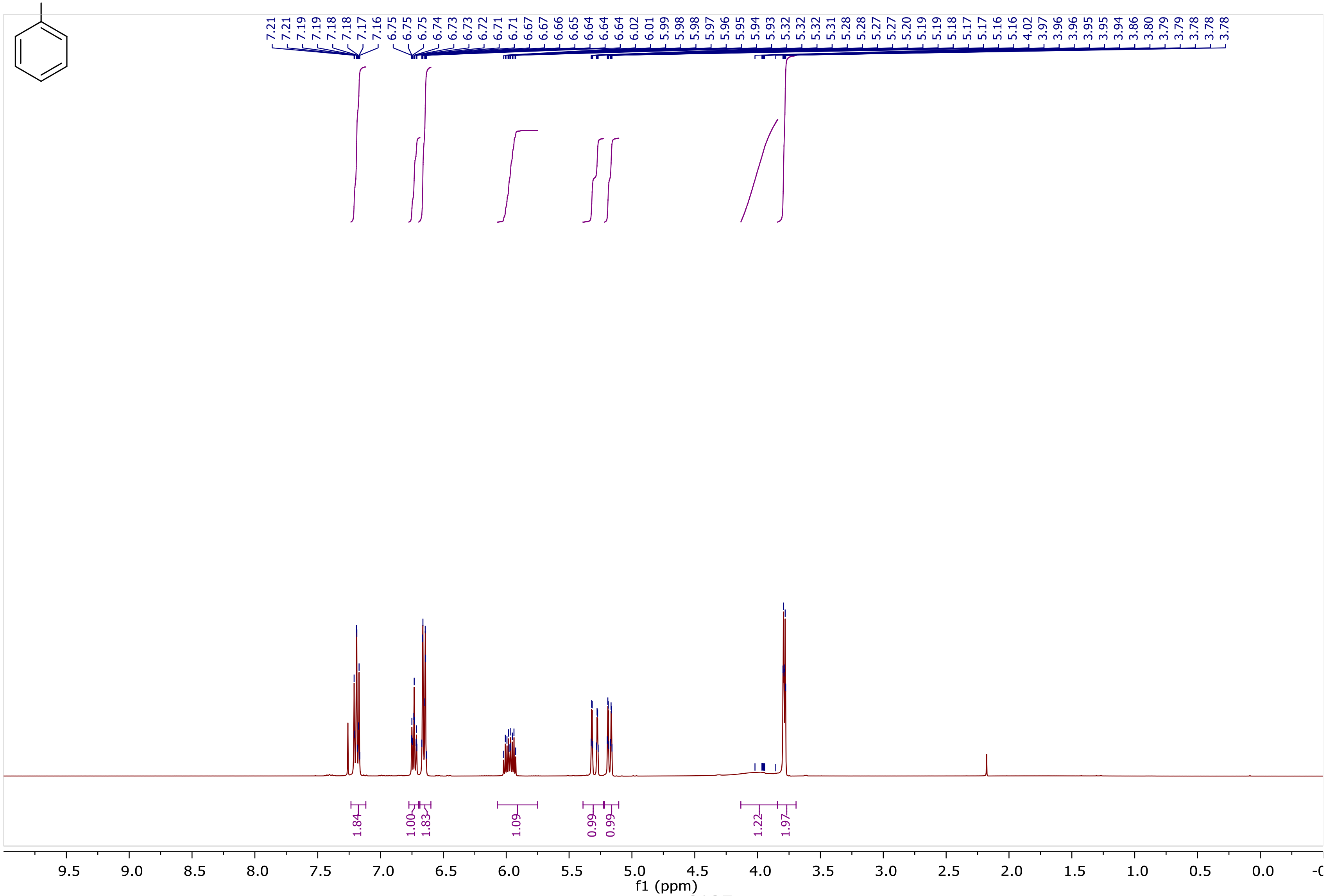

^{13}C NMR of *N*-allylaniline in $CDCl_3$

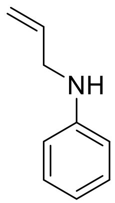

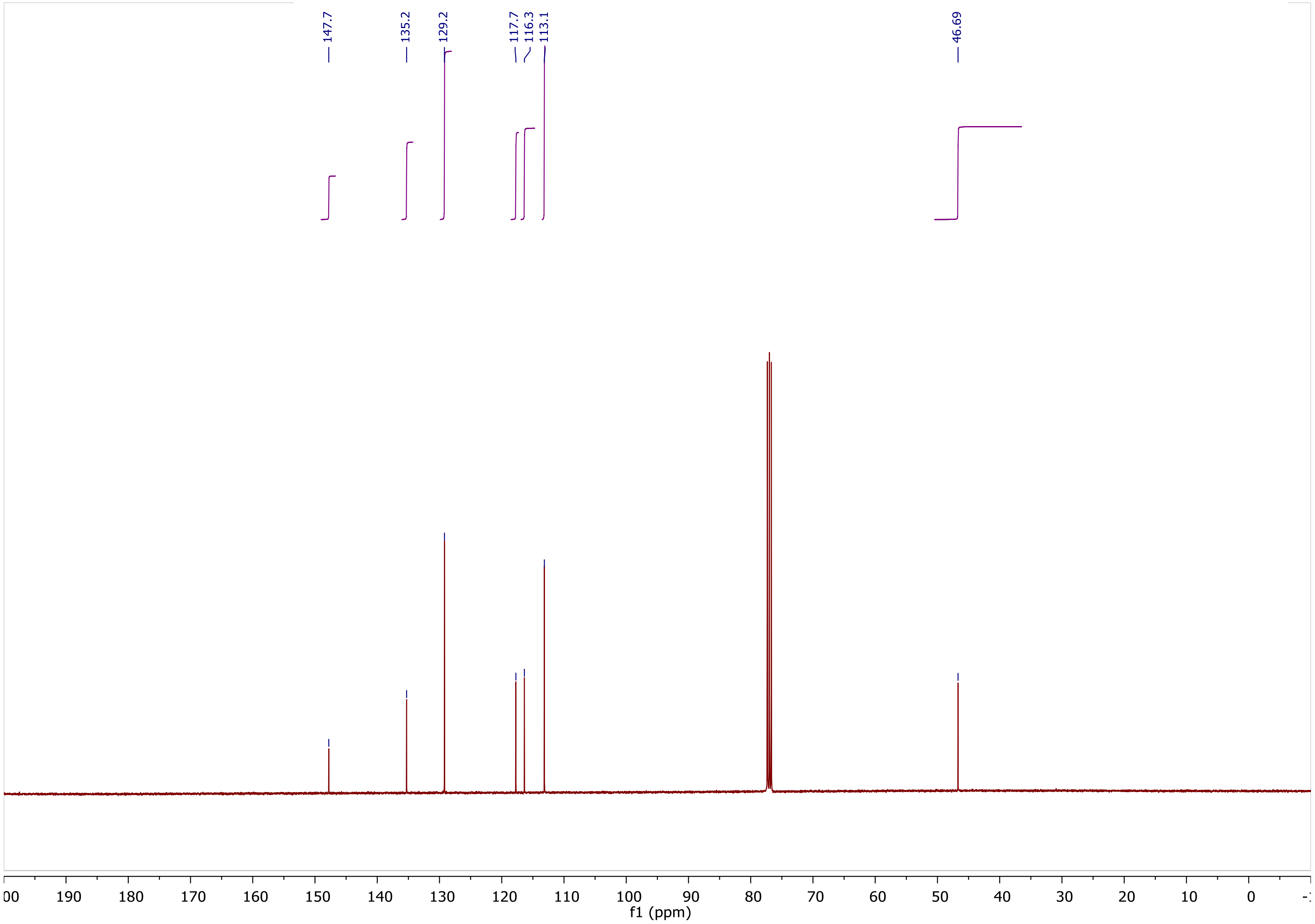

^{1}H NMR of 2-allylaniline in $CDCl_3$

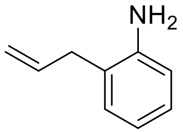

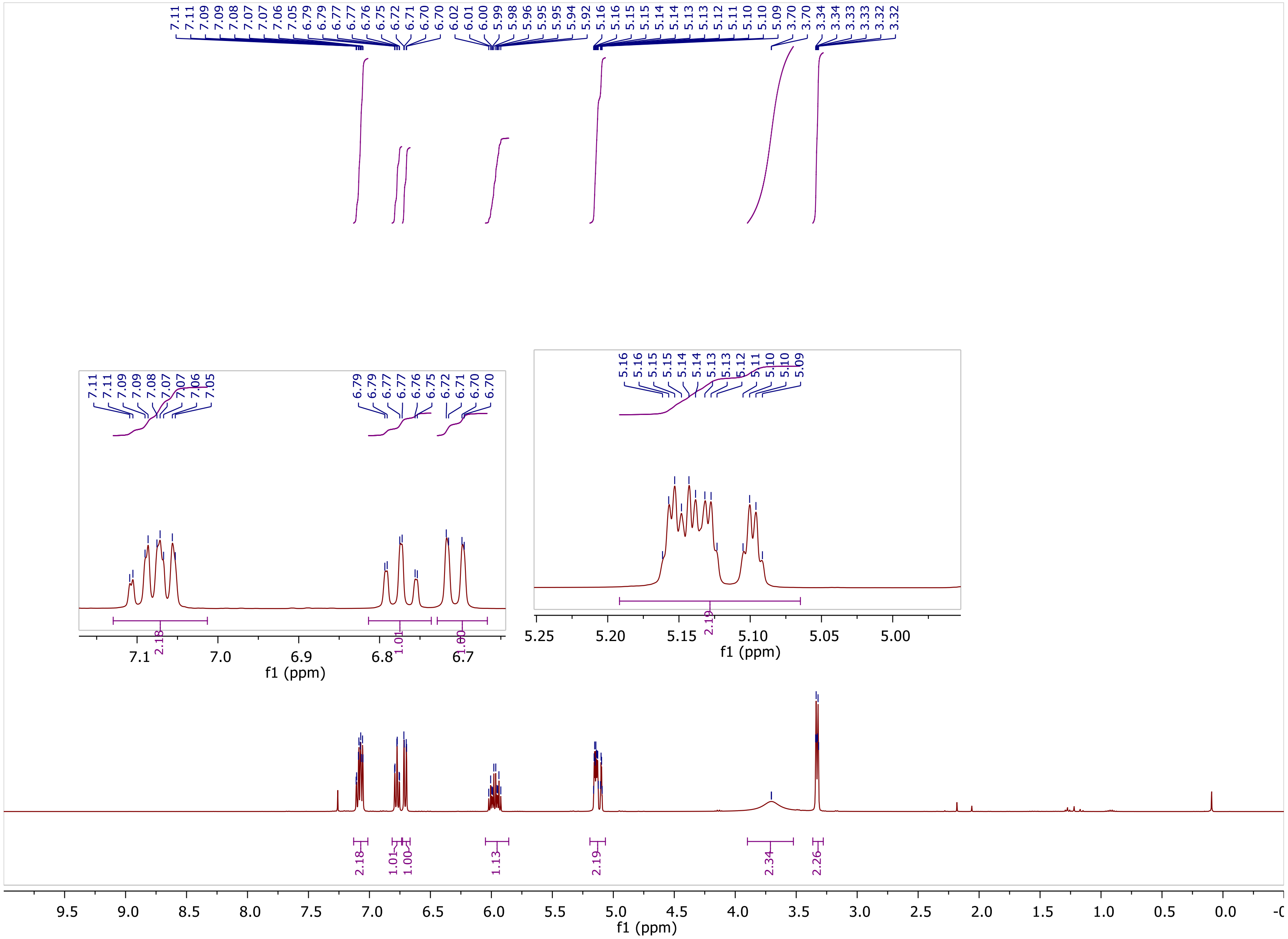

^{13}C NMR of 2-allylaniline in $CDCl_3$

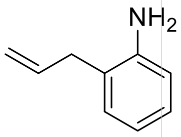

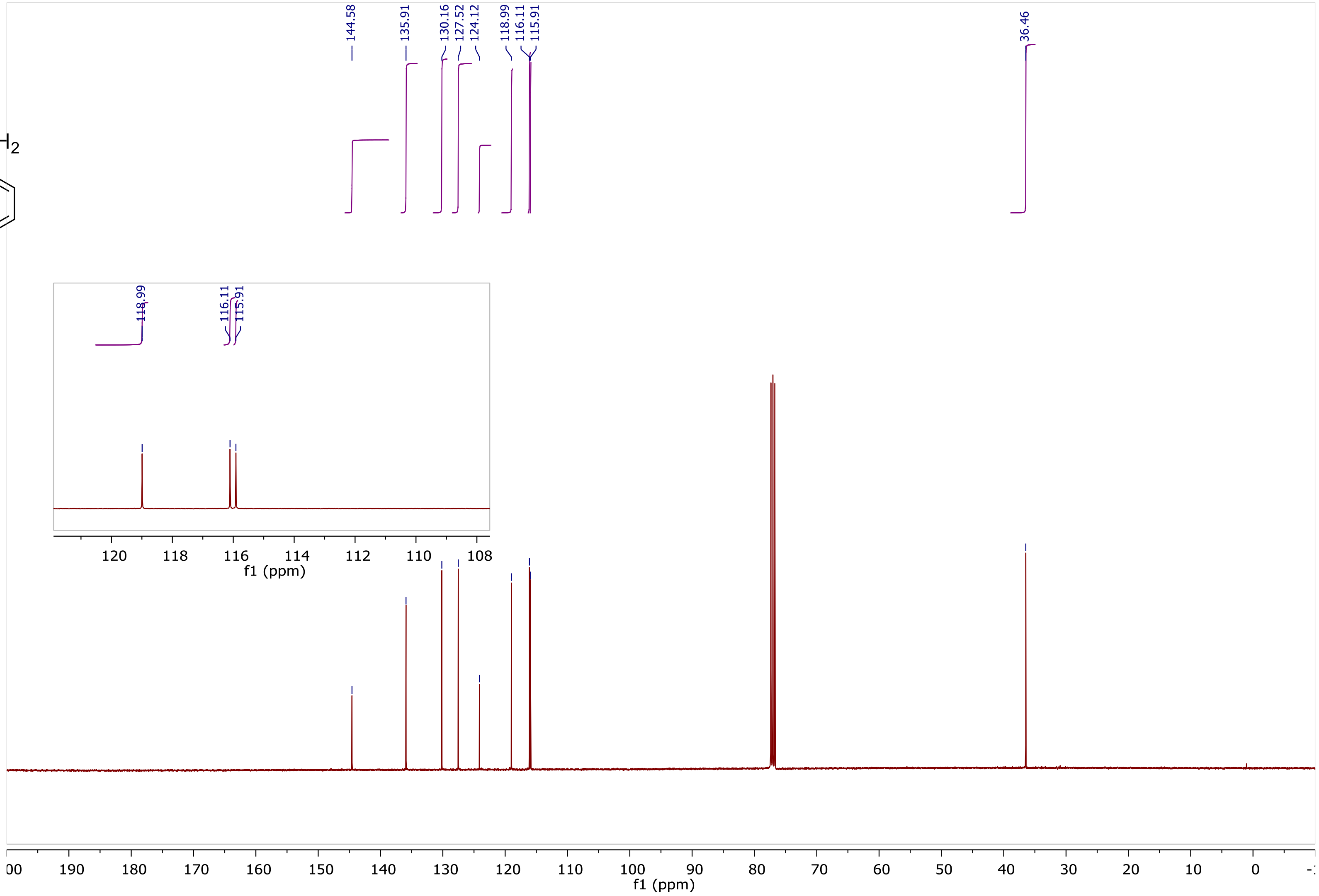

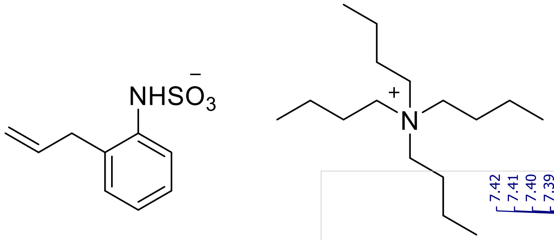

^{1}H NMR of tetrabutylammonium (2-allylphenyl)sulfamate **1t** in MeOD-*d*[4]

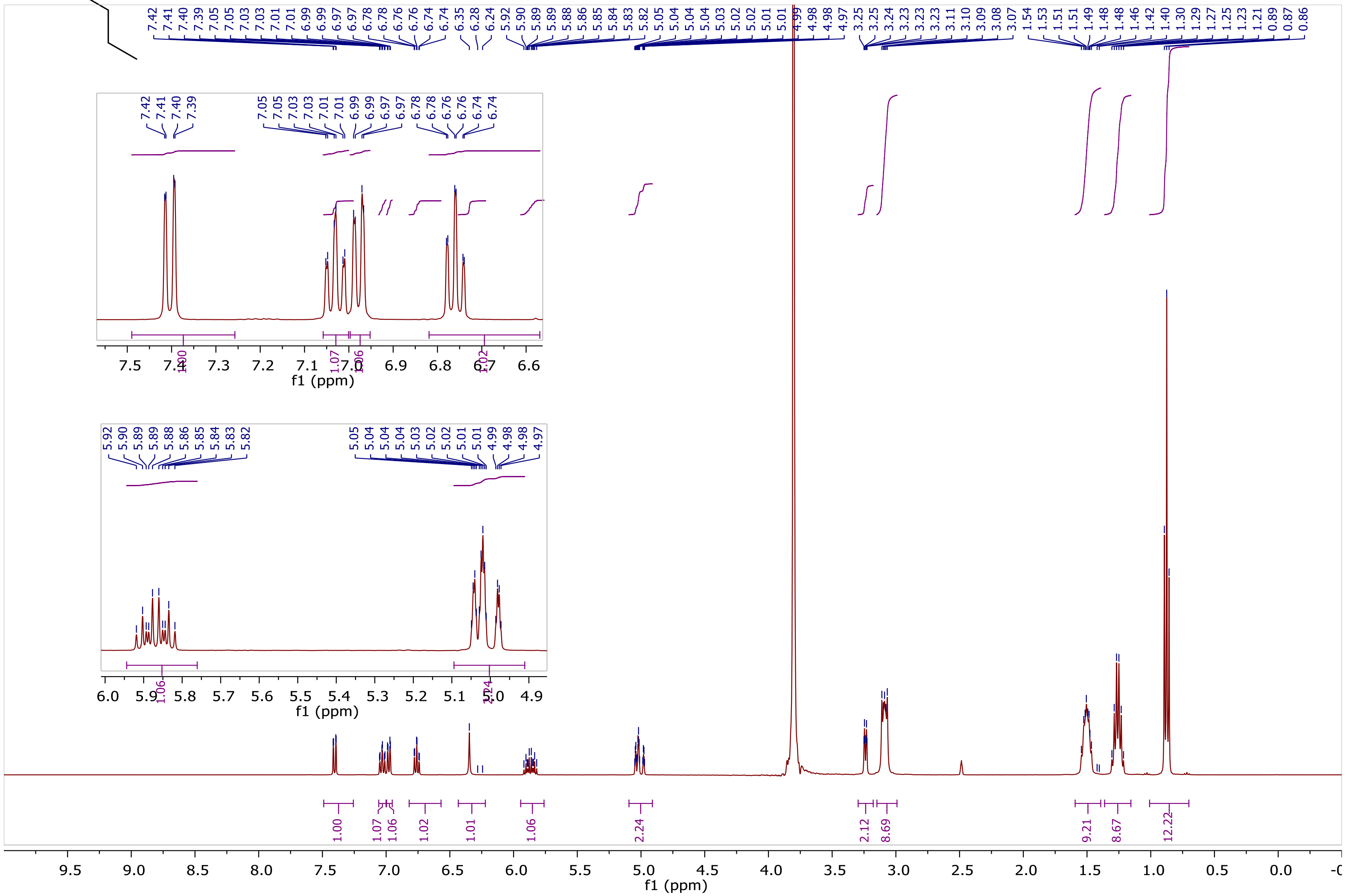

^{13}C NMR of tetrabutylammonium (2-allylphenyl)sulfamate **1t** in MeOD-*d*^{4}

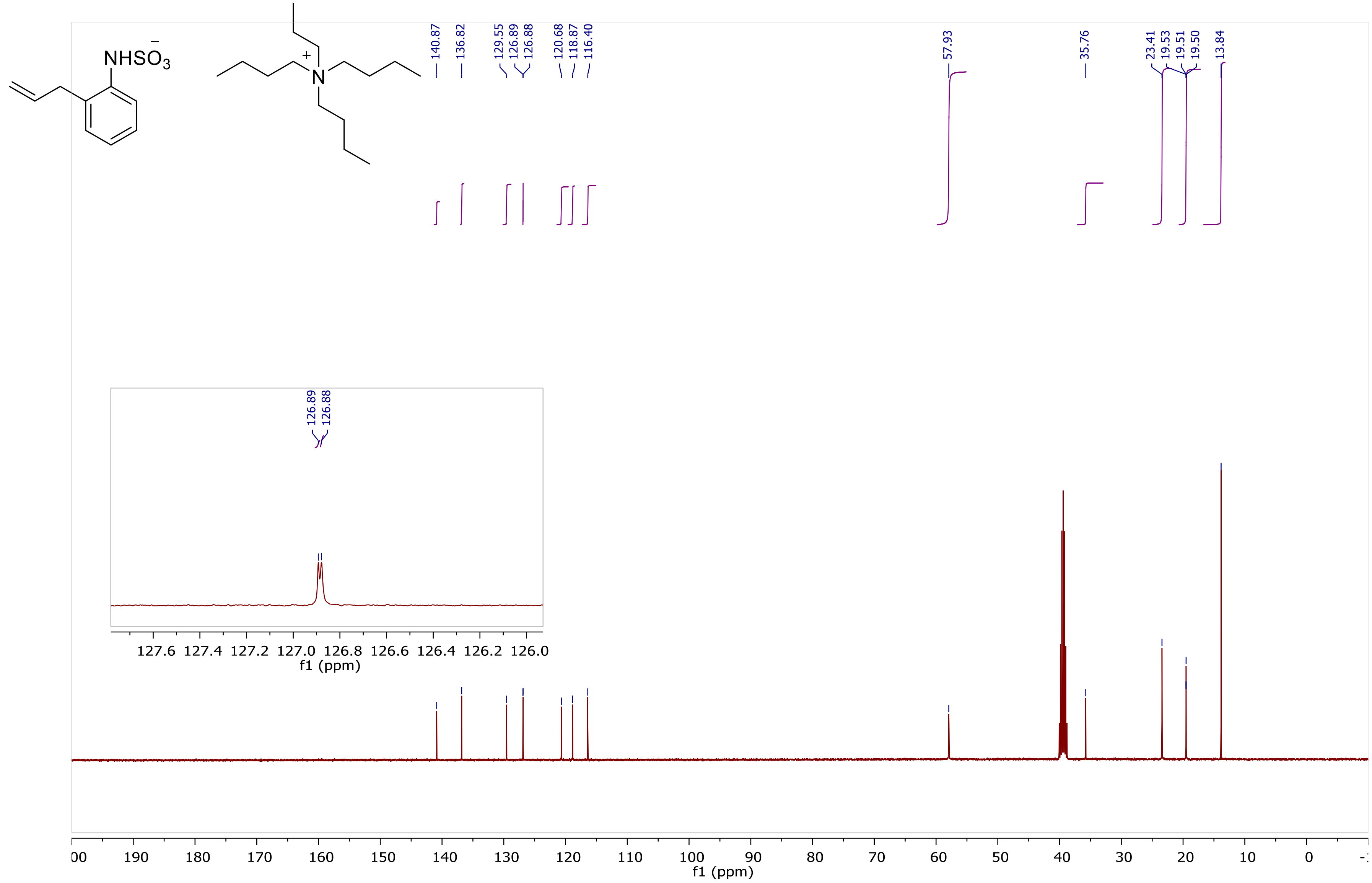

^{1}H NMR of tetrabutylammonium (2-biphenyl)sulfamate **1u** in MeOD-d^{4}

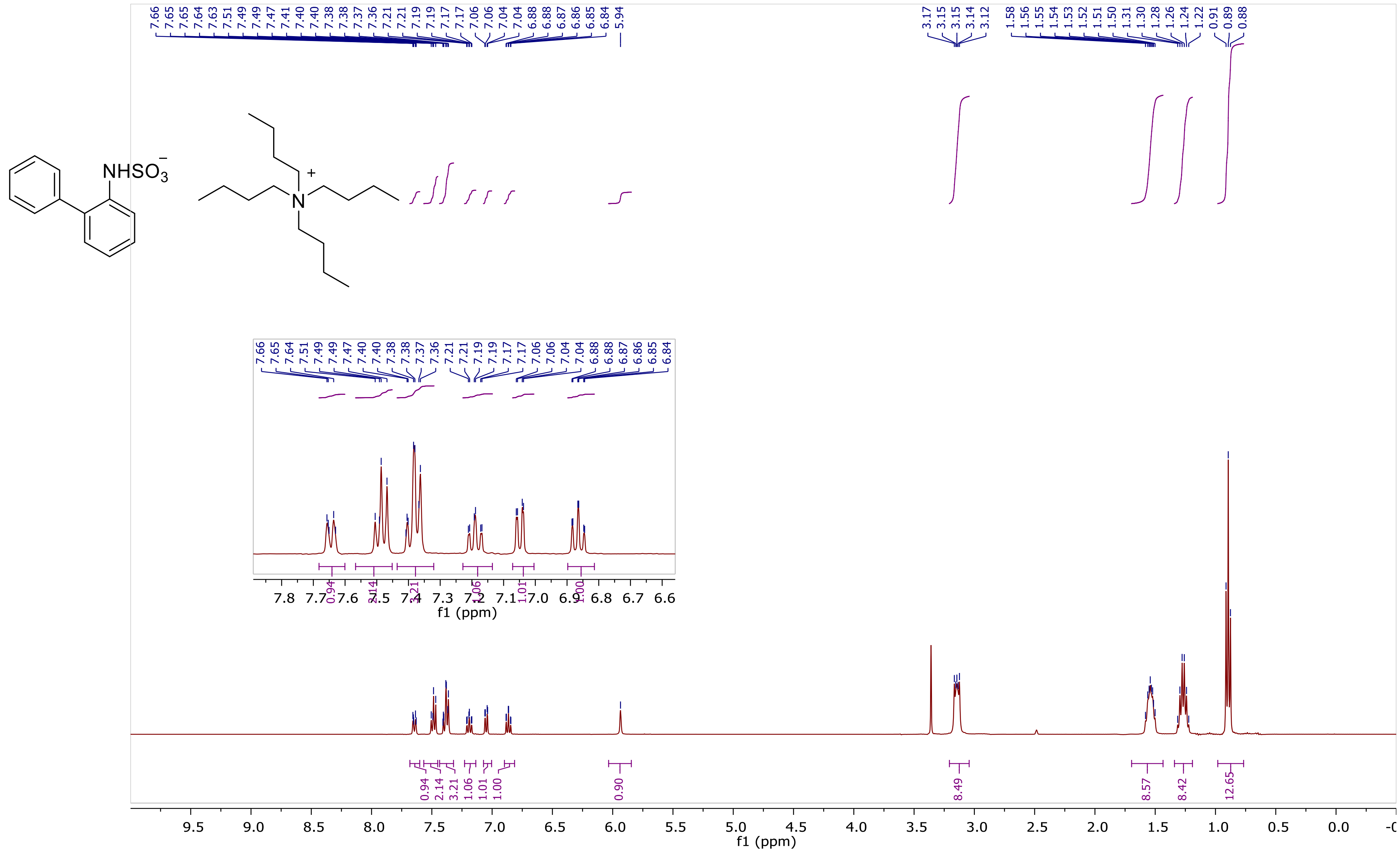

^{13}C NMR of tetrabutylammonium (2-biphenyl)sulfamate **1u** in MeOD-d^4

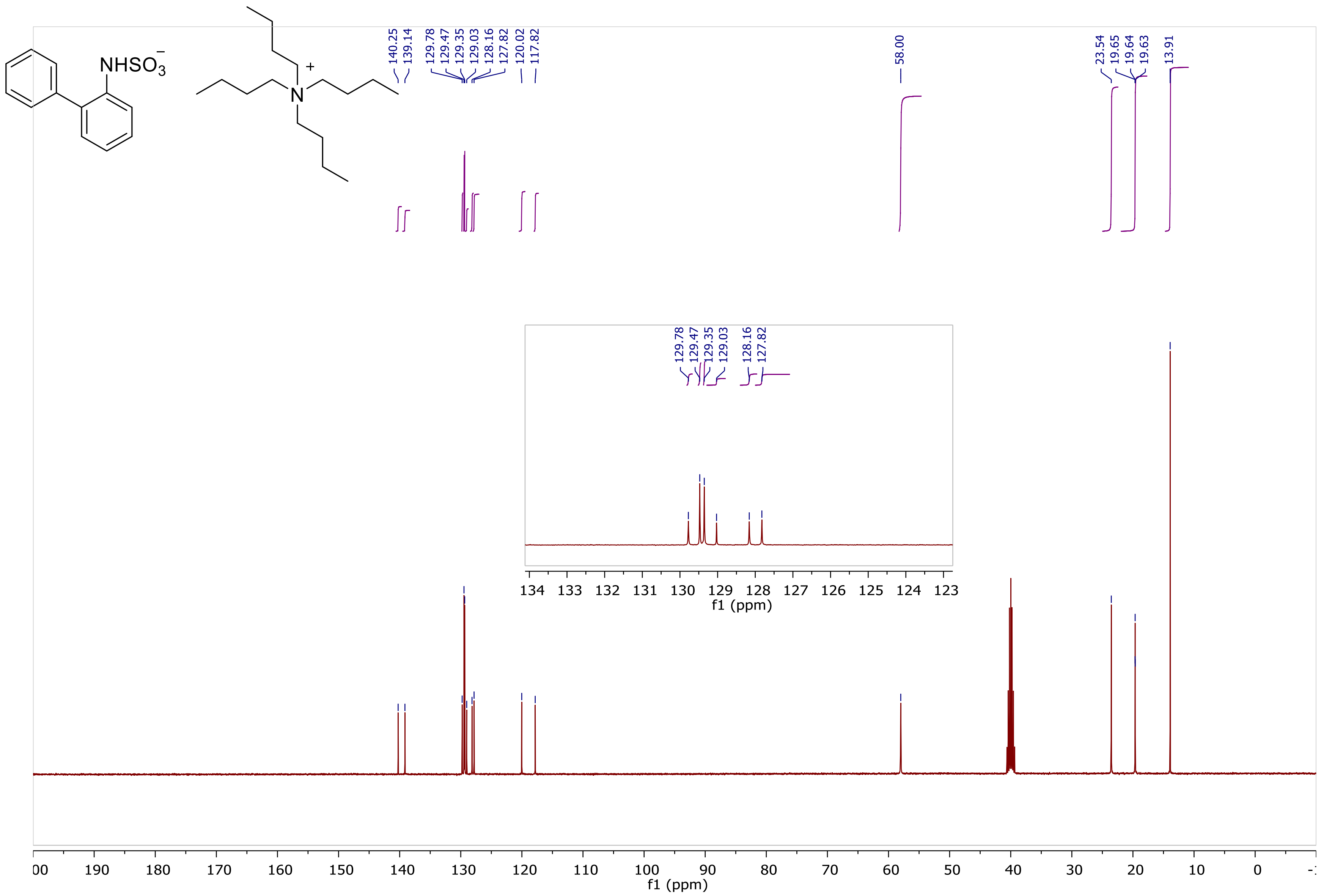

^{1}H NMR of 2-(4-chlorophenyl)aniline in $CDCl_3$

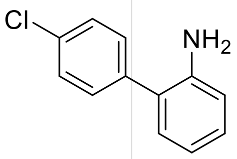

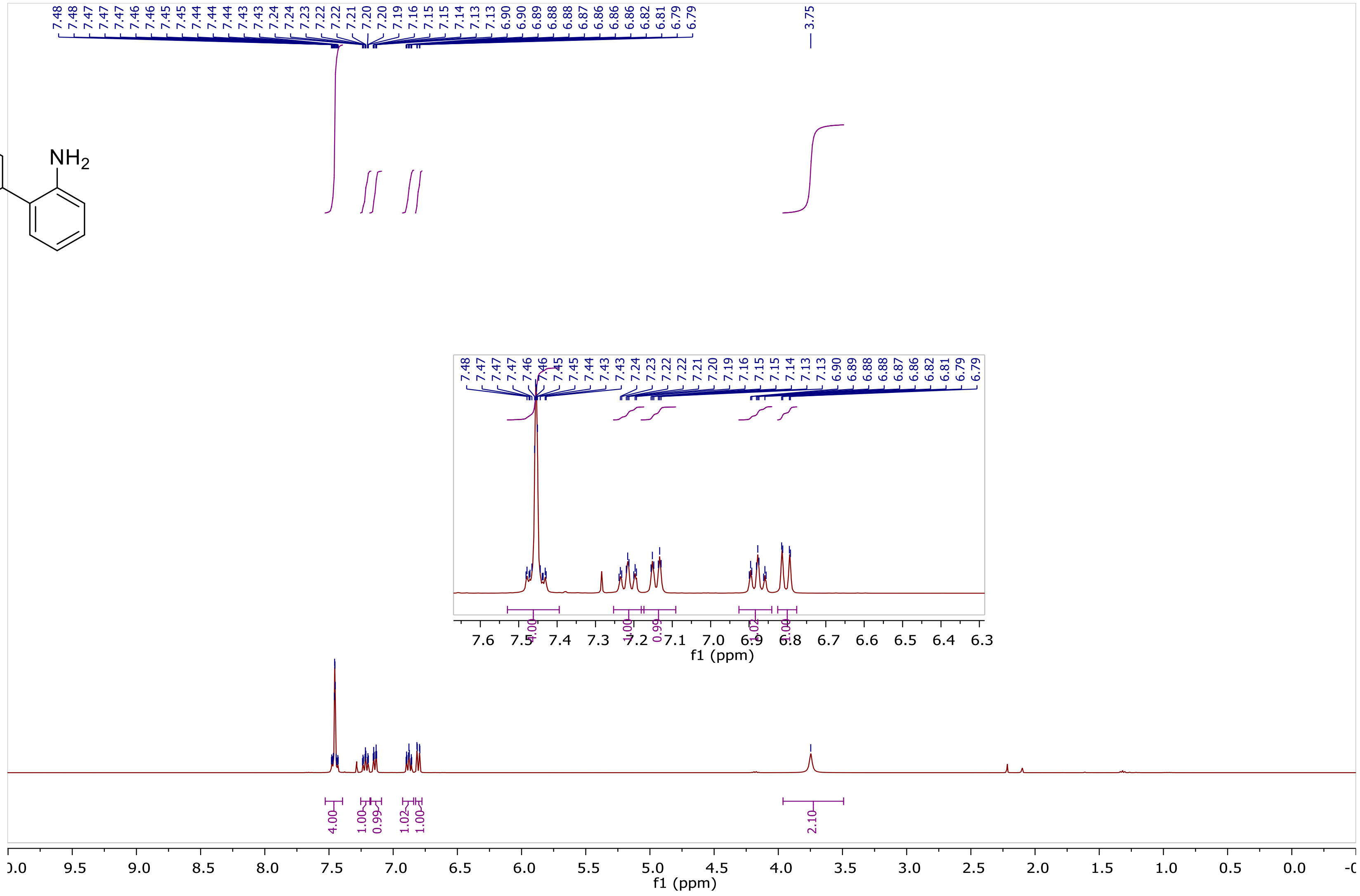

^{13}C NMR of 2-(4-chlorophenyl)aniline in $CDCl_3$

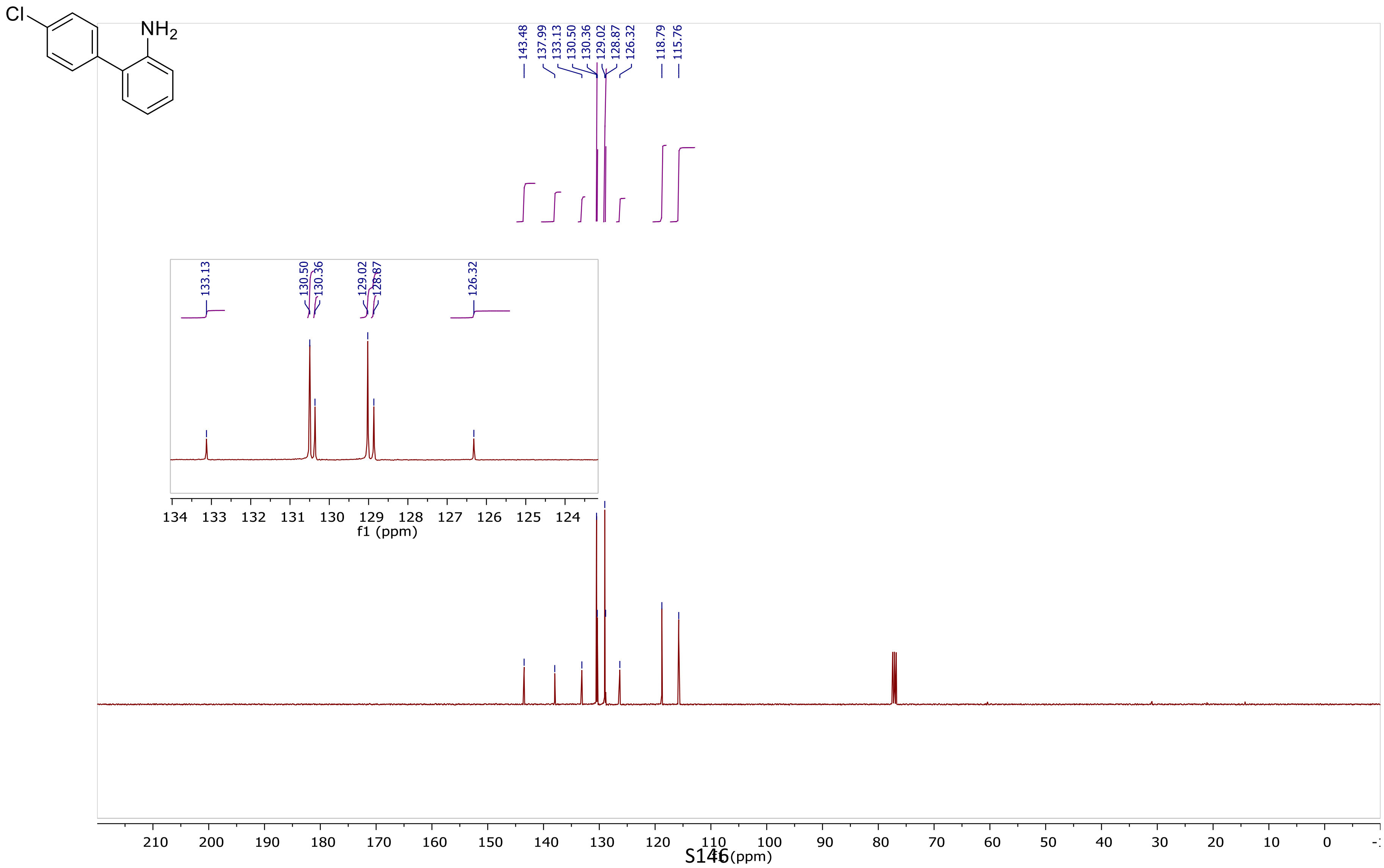

^{1}H NMR of tetrabutylammonium (2-(4-chlorophenyl)phenyl)sulfamate **1v** in MeOD-*d*[4]

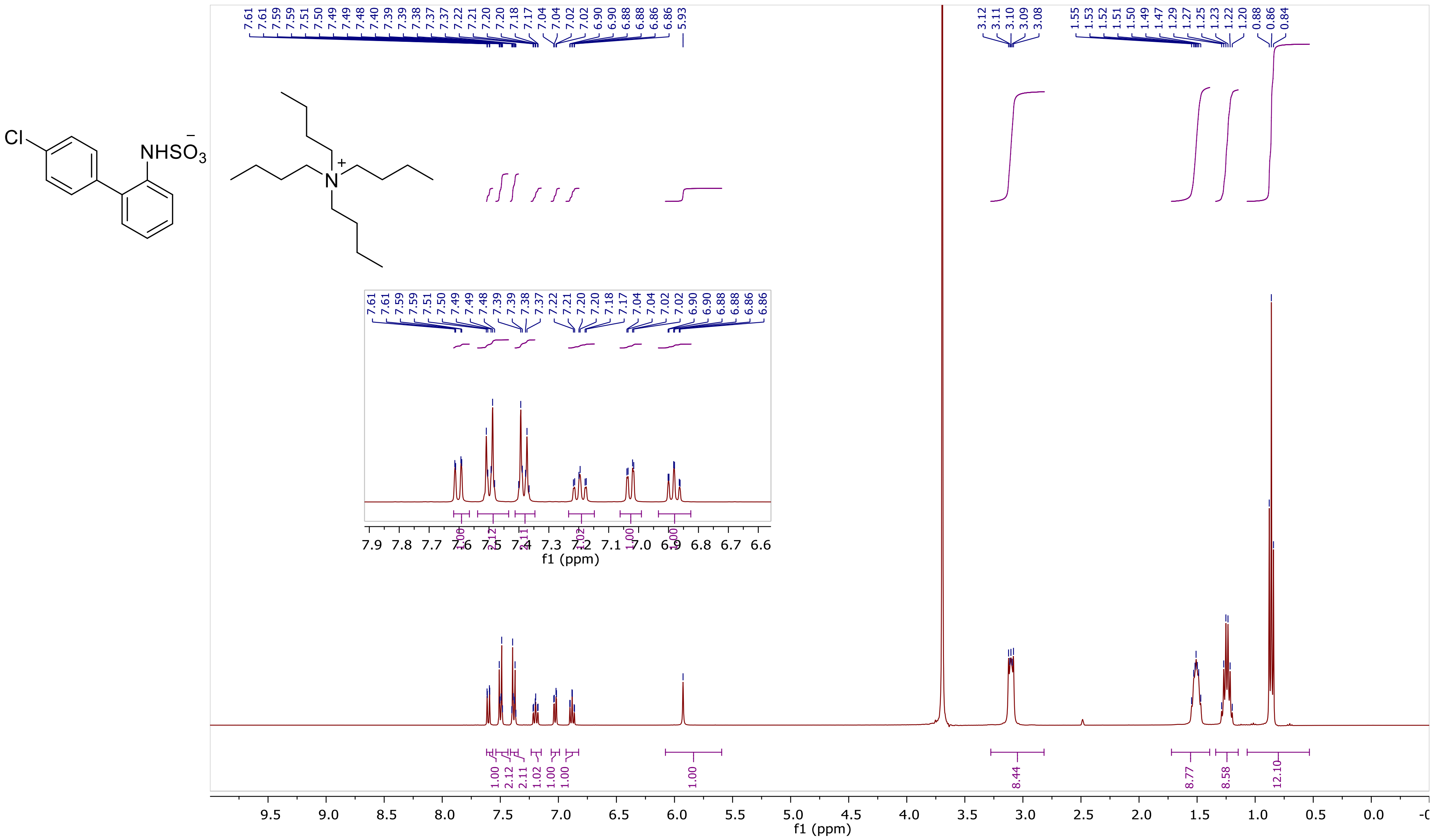

^{13}C NMR of tetrabutylammonium (2-(4-chlorophenyl)phenyl)sulfamate **1v** in MeOD-d^4

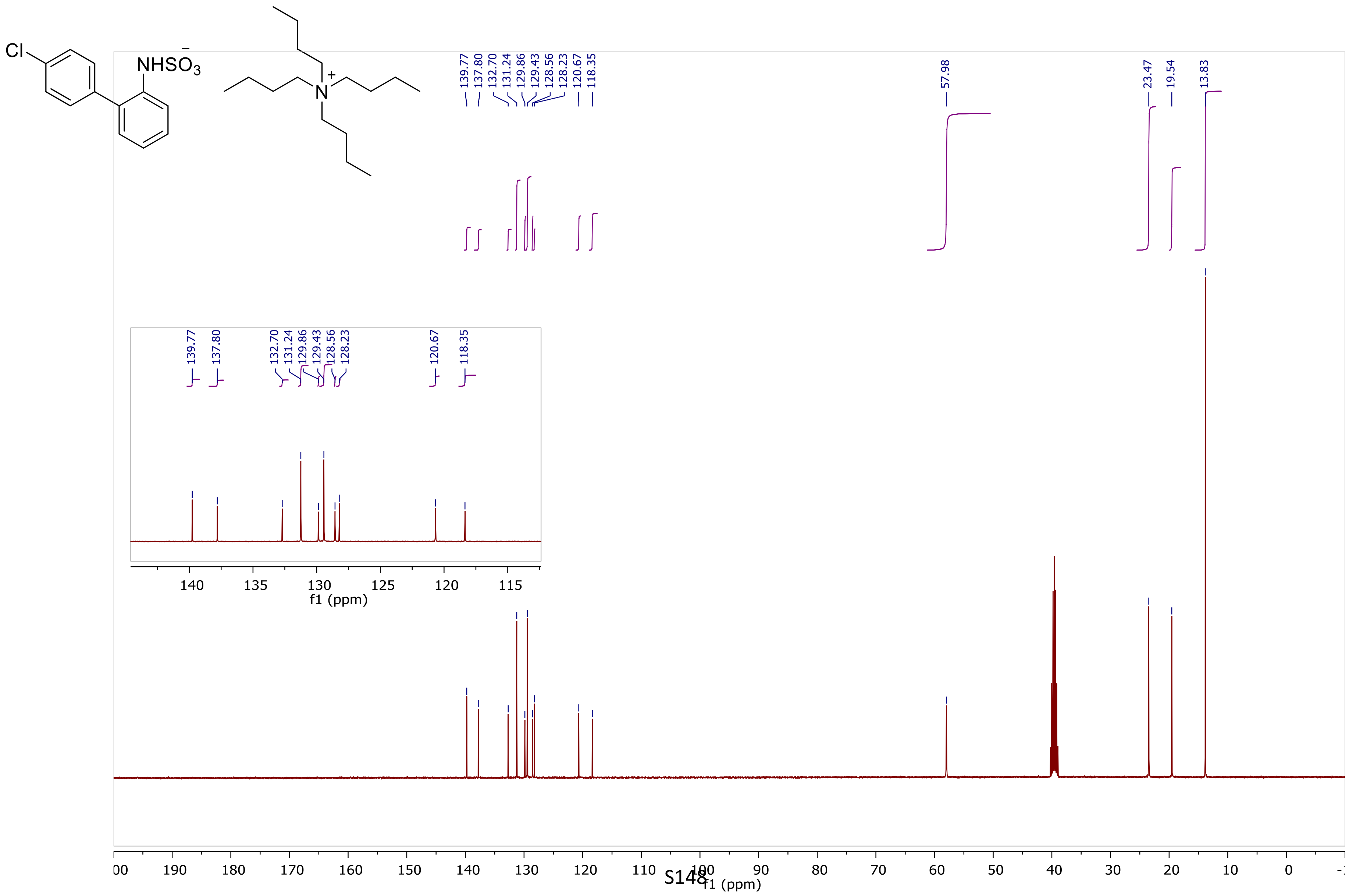

^{1}H NMR of 2-(*p*-tolyl)aniline in $CDCl_3$

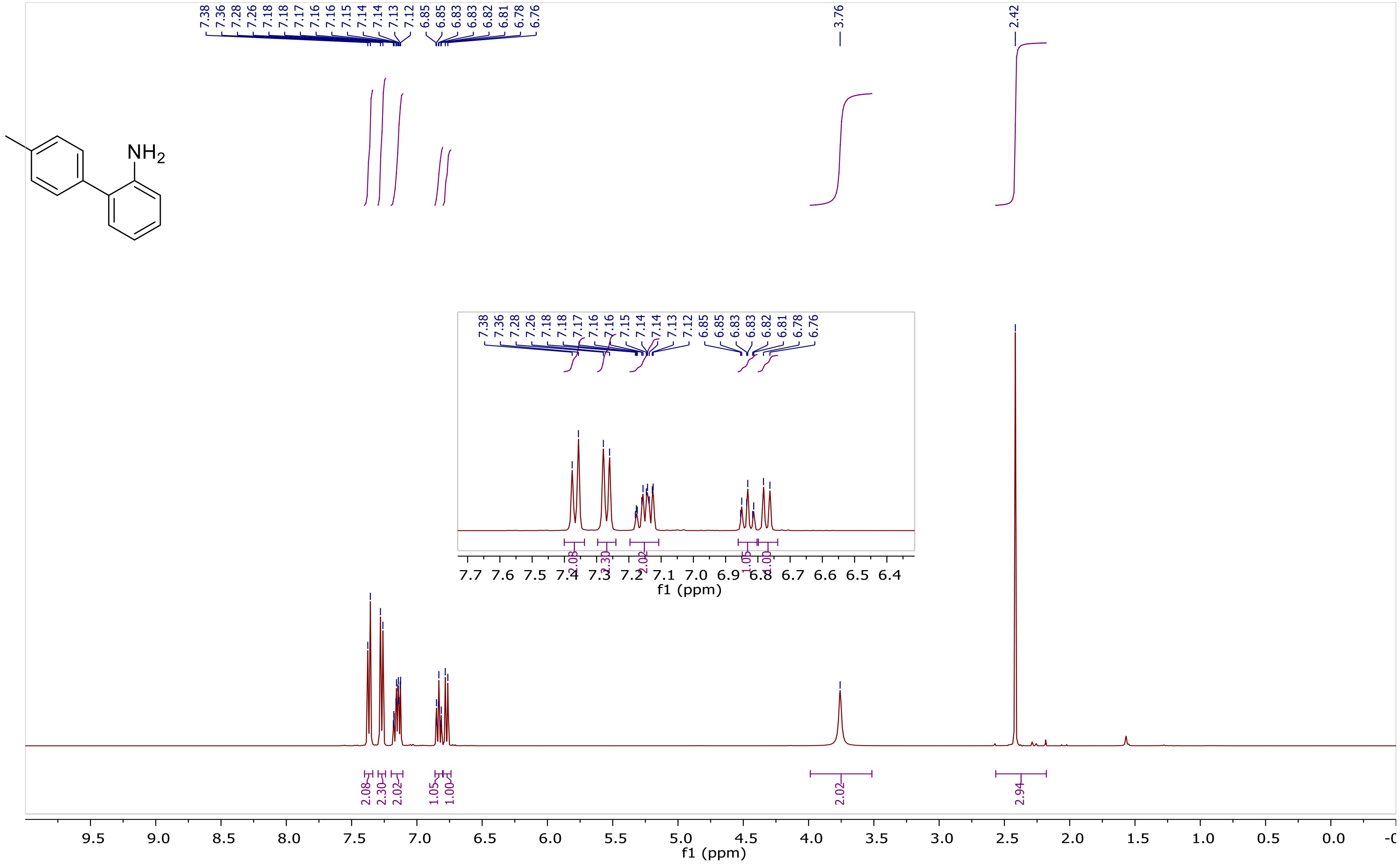

^{13}C NMR of 2-(*p*-tolyl)aniline in $CDCl_3$

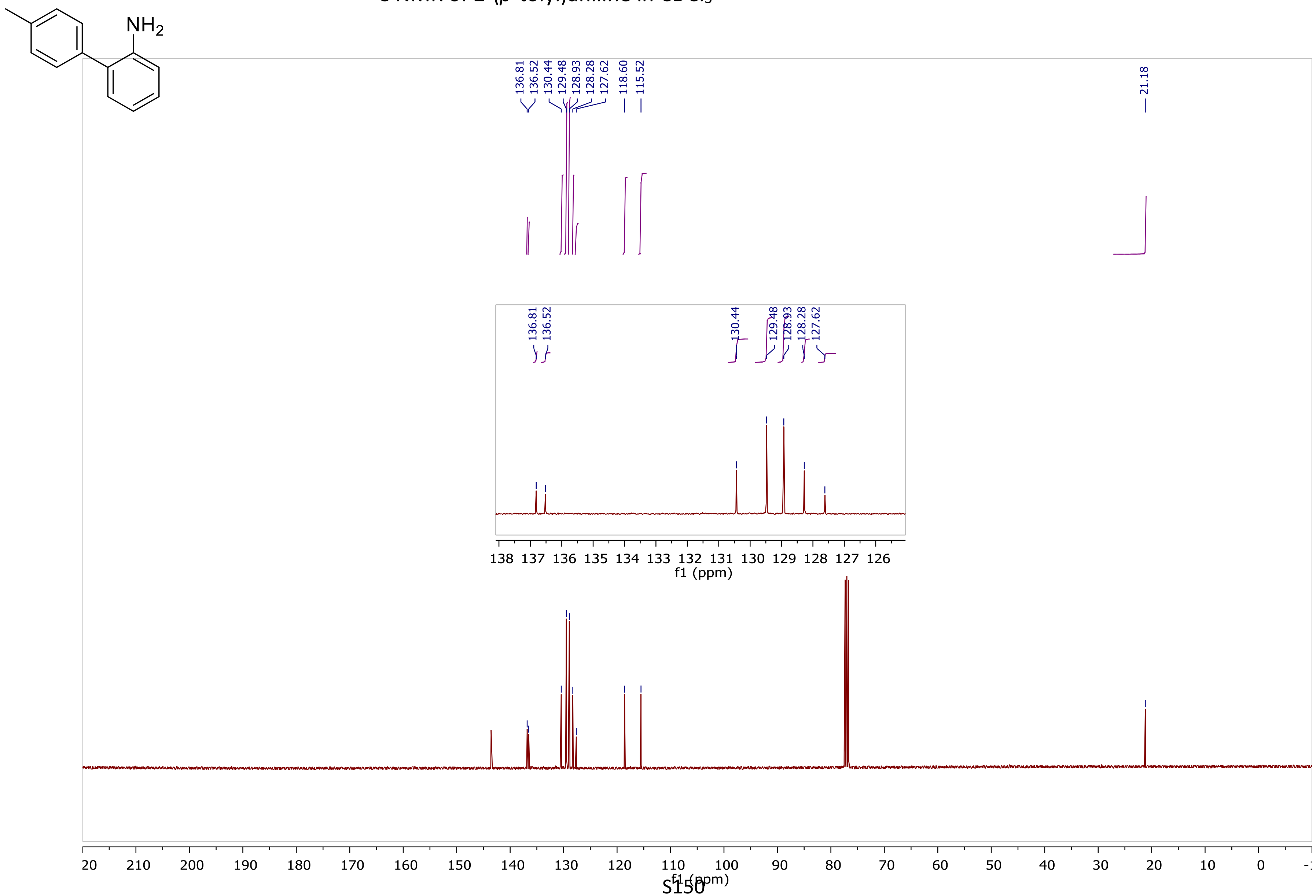

^{1}H NMR of tetrabutylammonium (2-(*p*-tolyl)phenyl)sulfamate **1w** in MeOD-*d*[4]

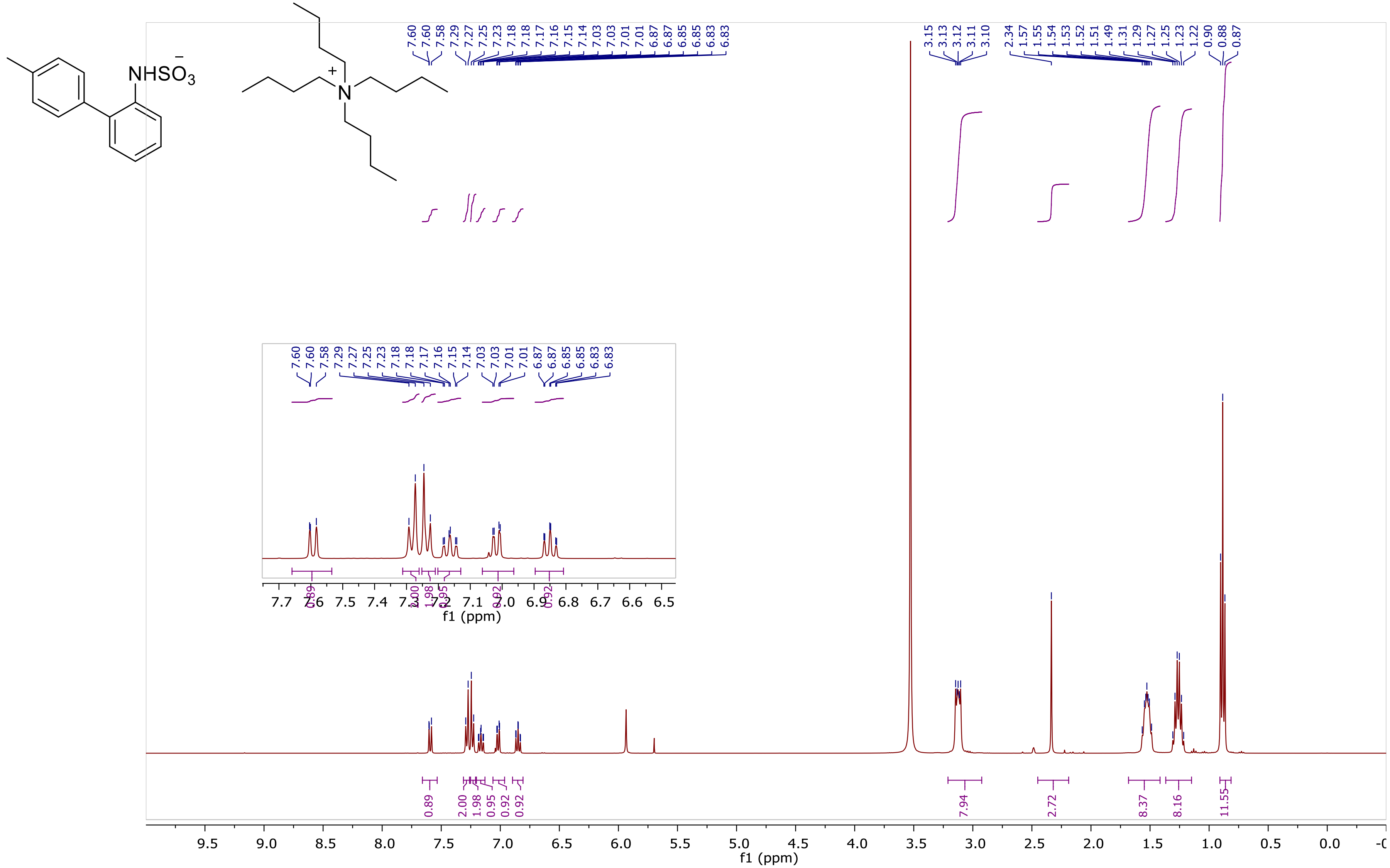

^{13}C NMR of tetrabuytlammonium (2-(*p*-tolyl)phenyl)sulfamate **1w** in MeOD-d^4

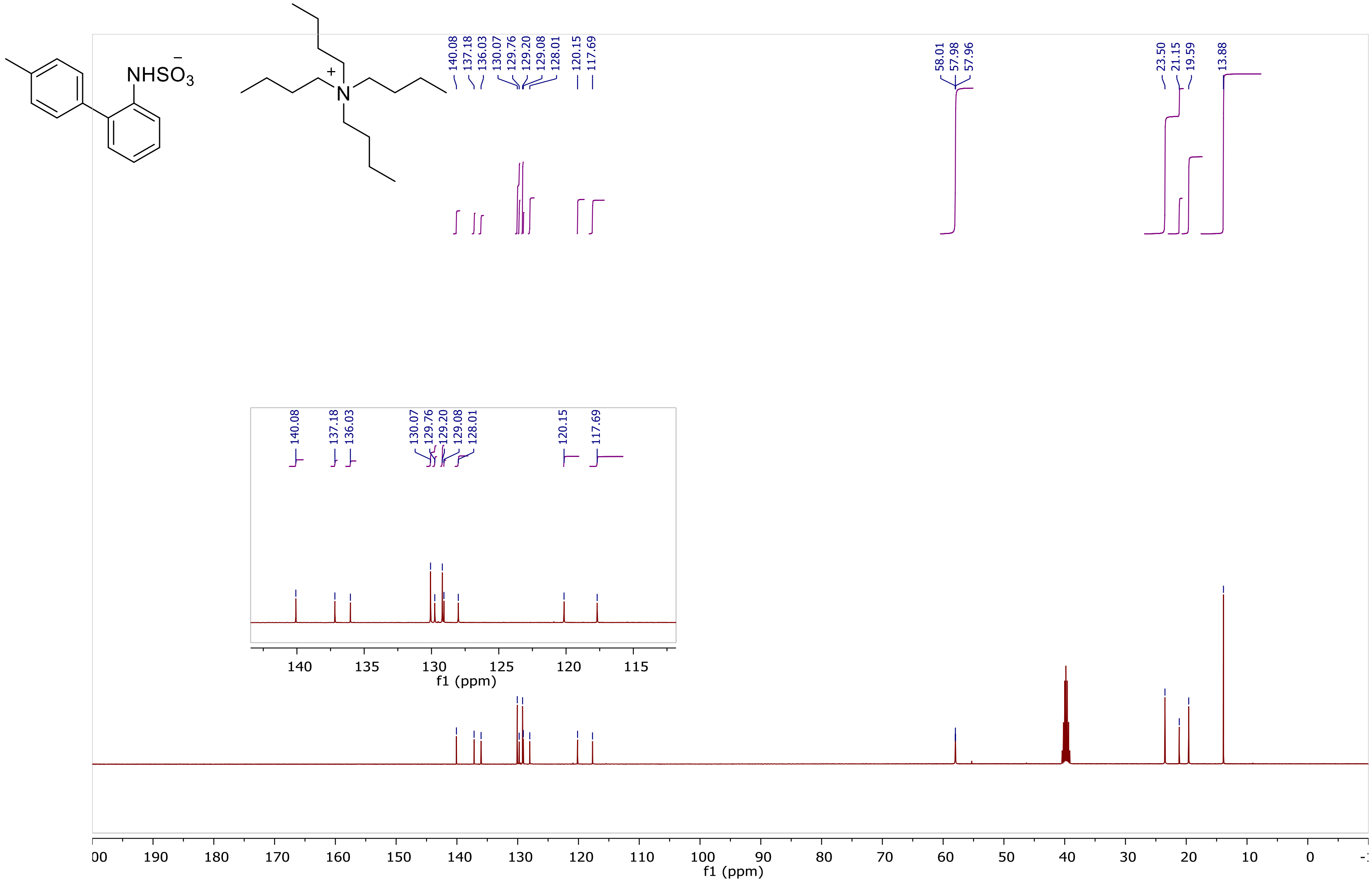

^{1}H NMR of tetrabutylammonium (3,5-dimethoxyphenyl)sulfamate **1x** in $CDCl_3$

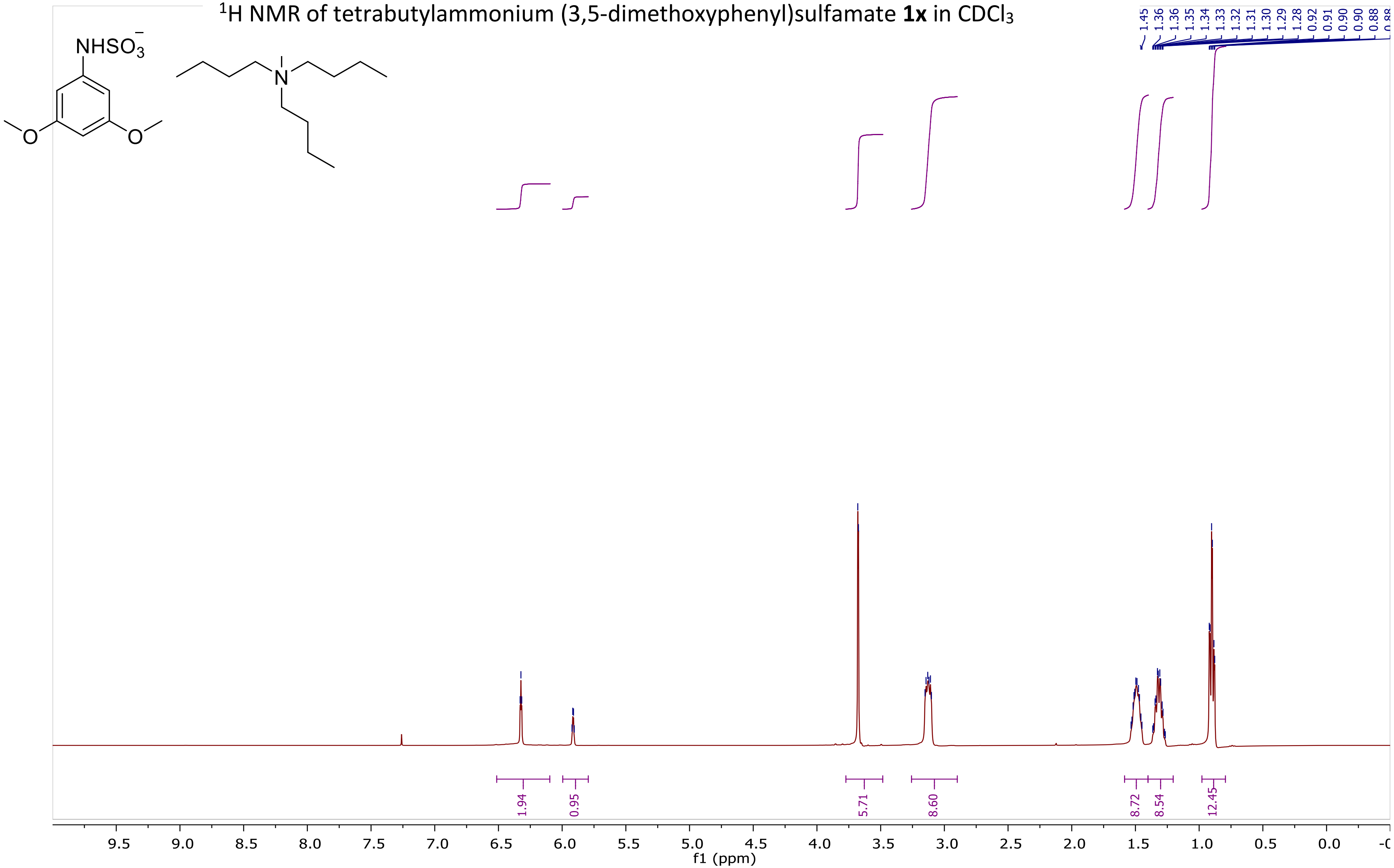

^{1}H NMR of tetrabutylammonium (3,5-dimethoxyphenyl)sulfamate **1x** in $CDCl_3$

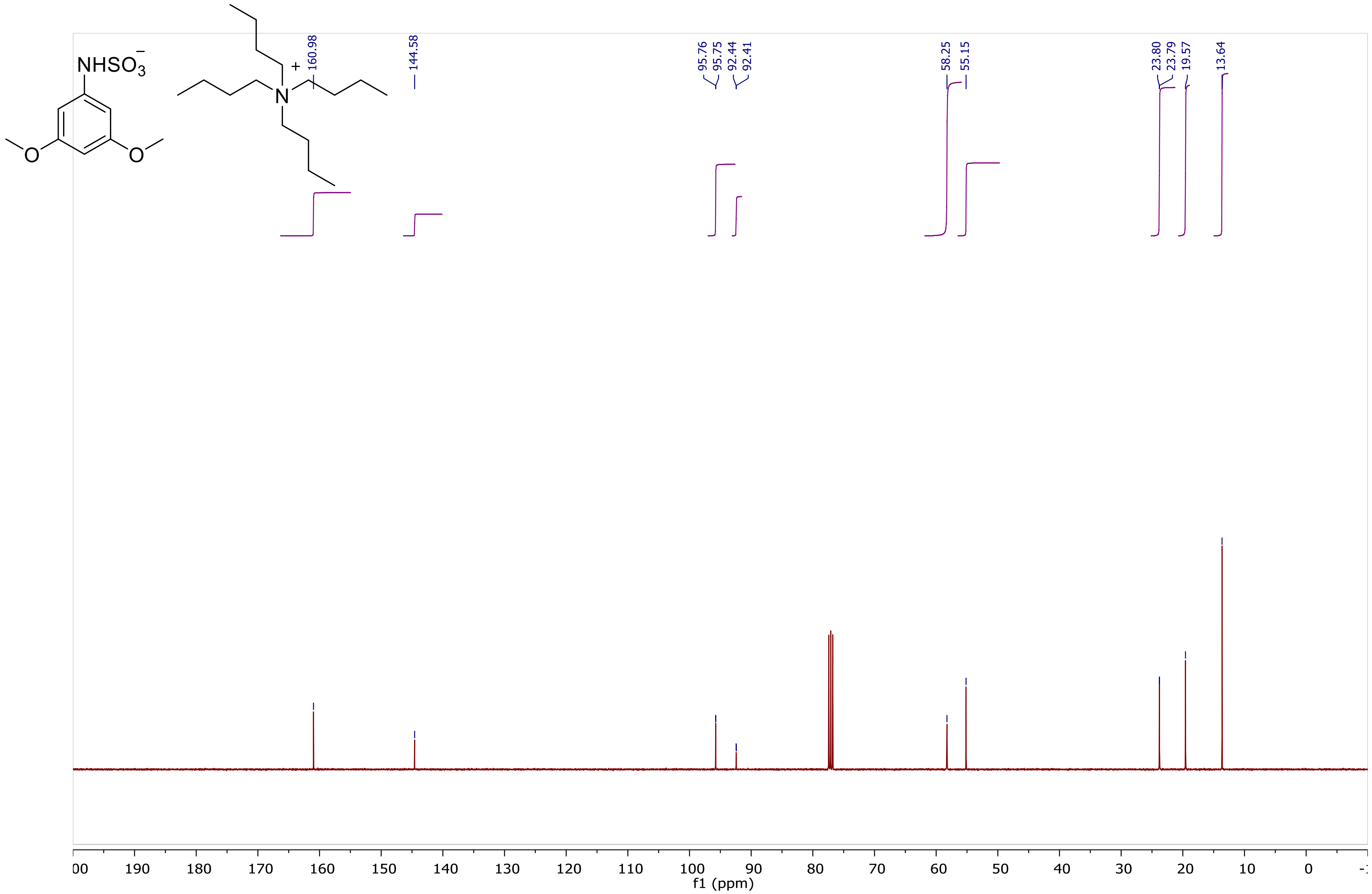

^{1}H NMR of tetrabutylammonium (3-chlorophenyl)sulfamate **1y** in $CDCl_3$

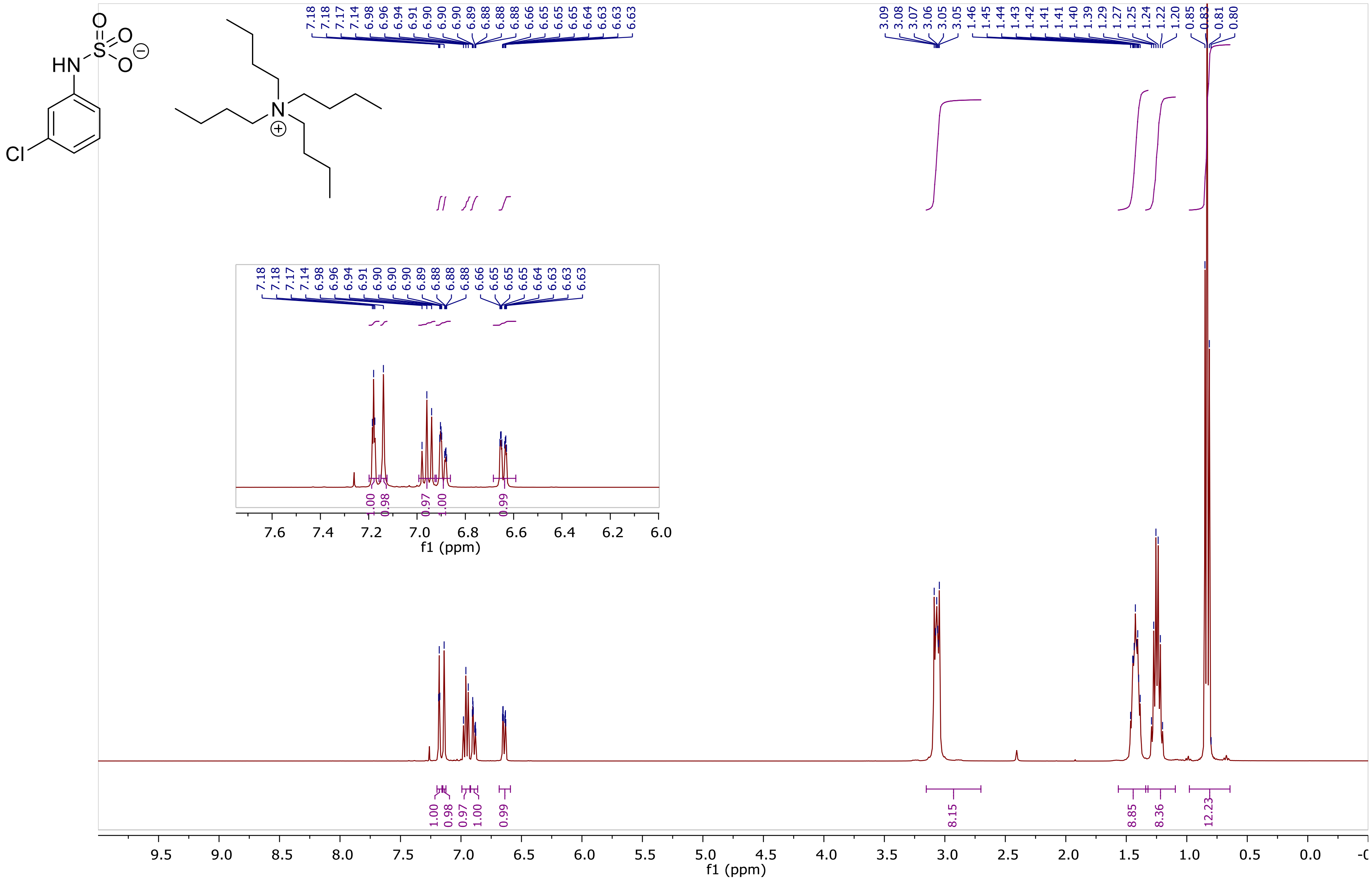

^{13}C NMR of tetrabutylammonium (3-chlorophenyl)sulfamate **1y** in $CDCl_3$

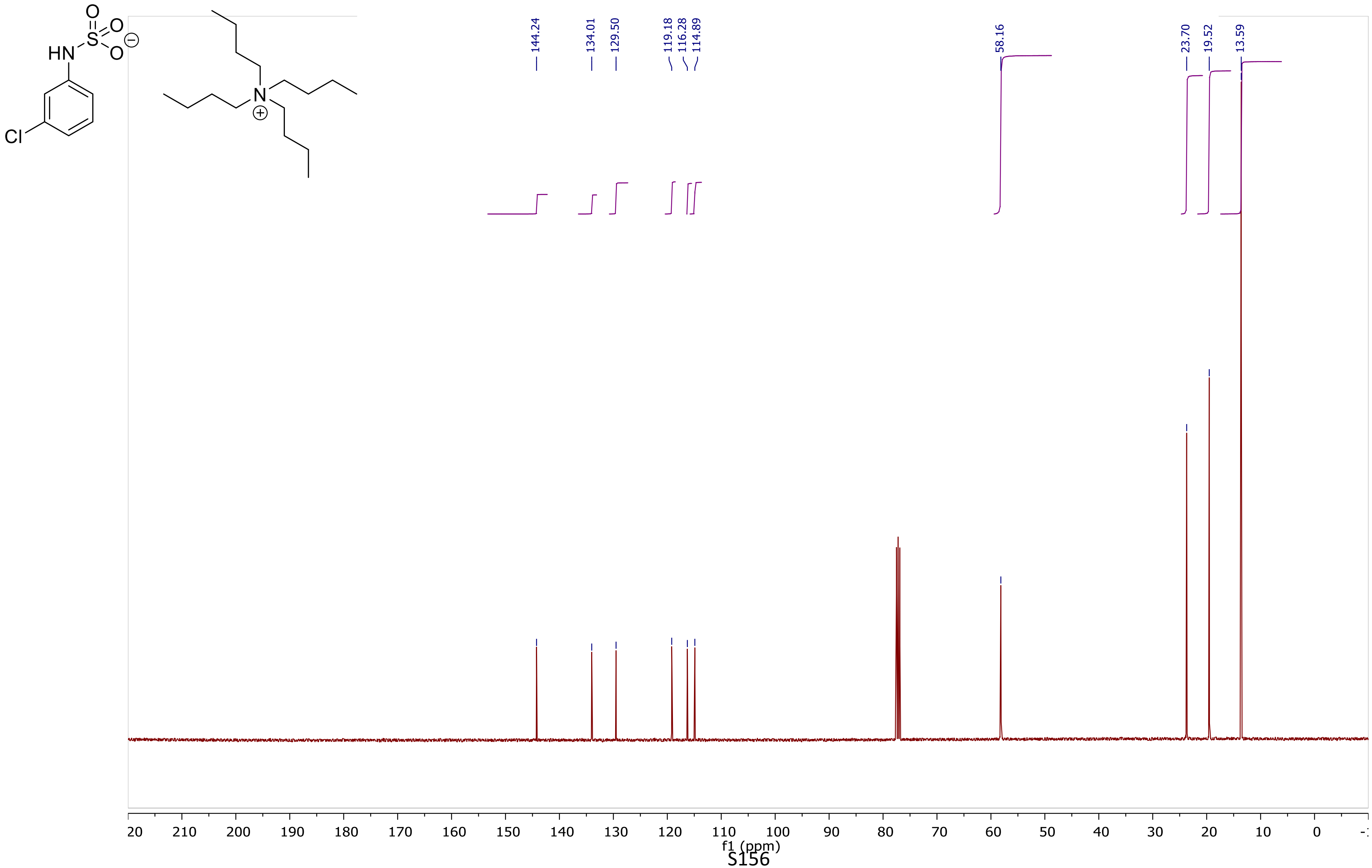

^{1}H NMR of tetrabutylammonium (3-bromophenyl)sulfamate **1z** in $CDCl_3$

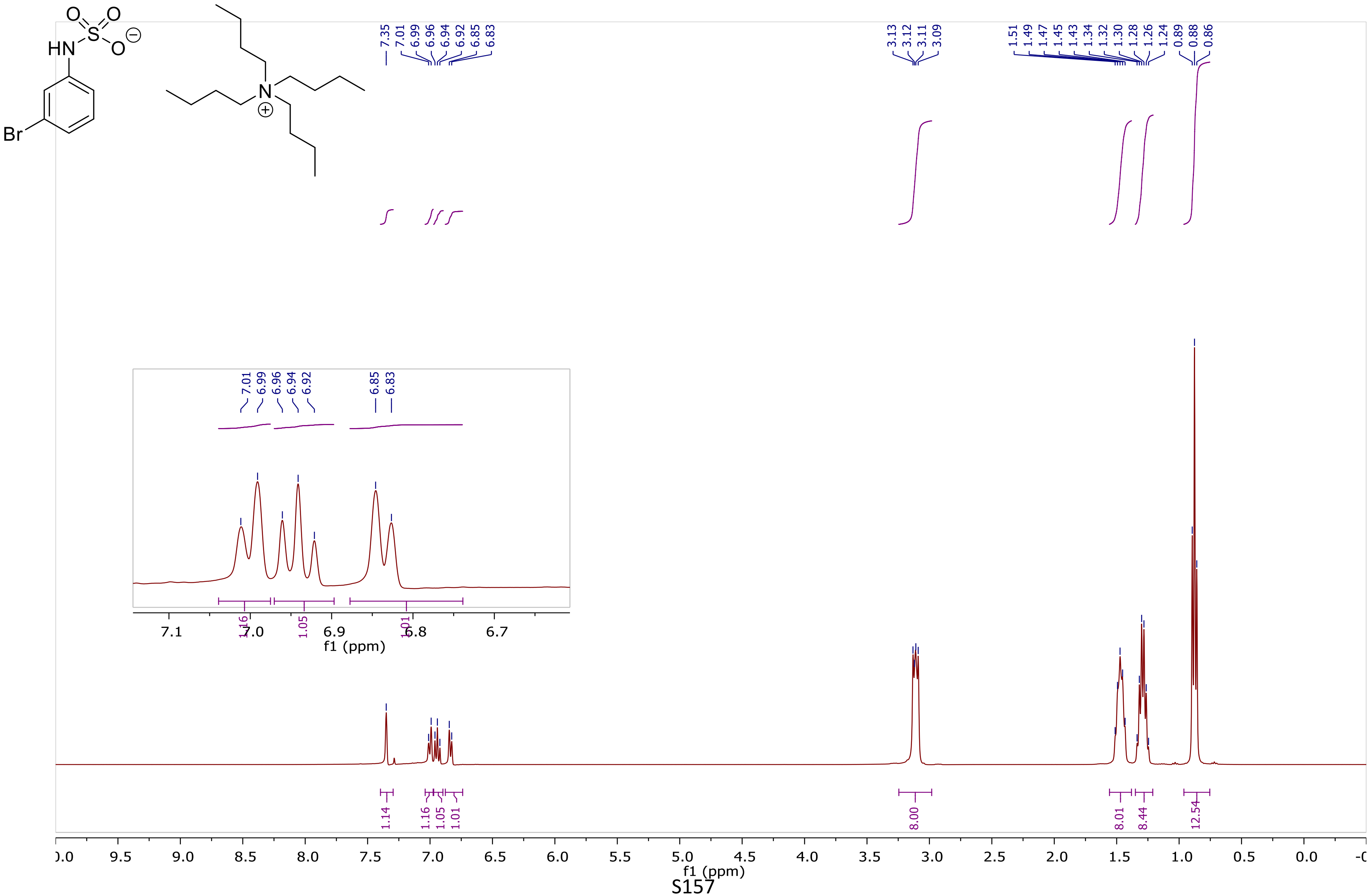

^{13}C NMR of tetrabutylammonium (3-bromophenyl)sulfamate **1z** in $CDCl_3$

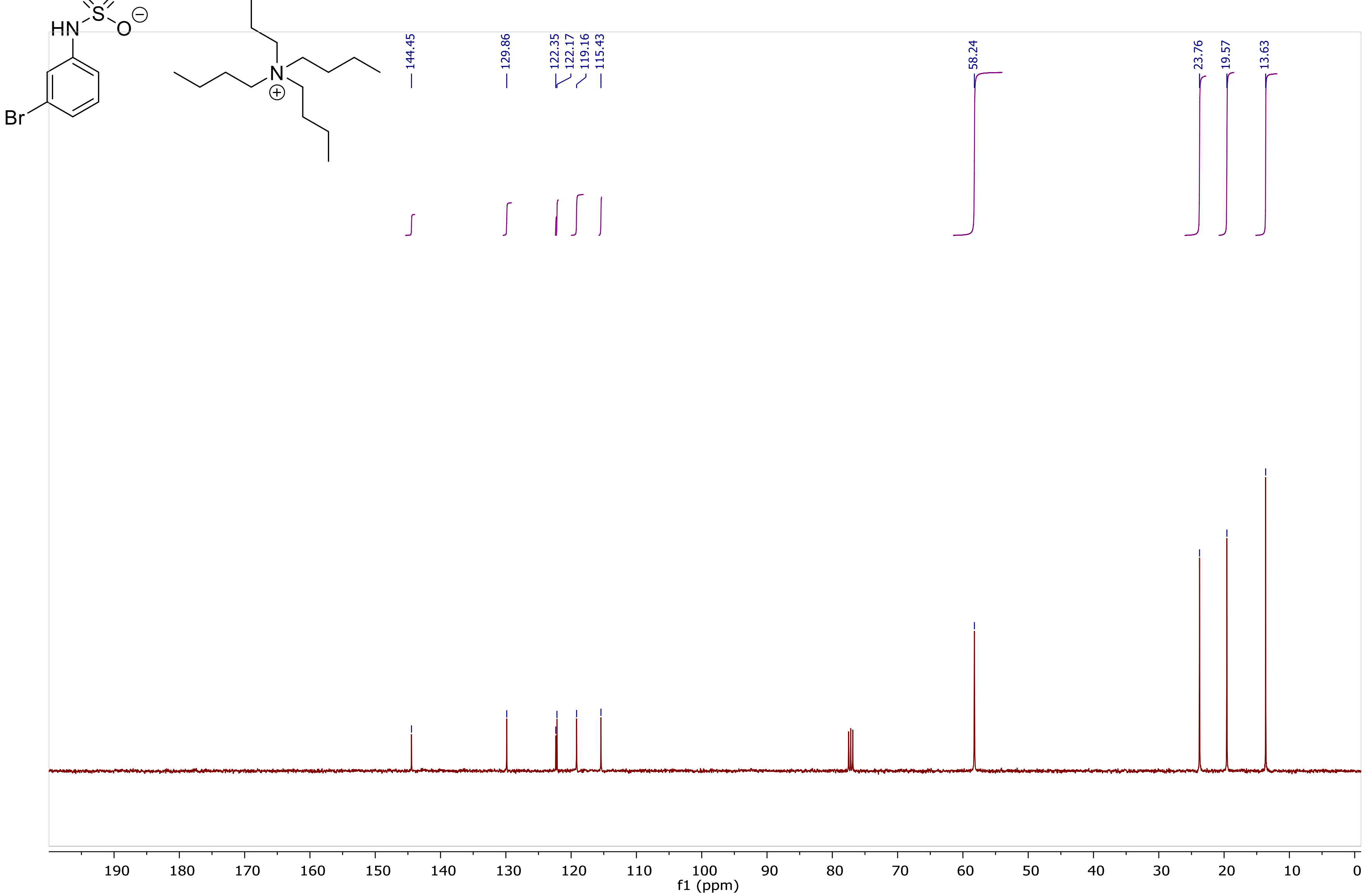

^{1}H NMR of tetrabutylammonium (3,5-difluorophenyl)sulfamate **1aa** in $CDCl_3$

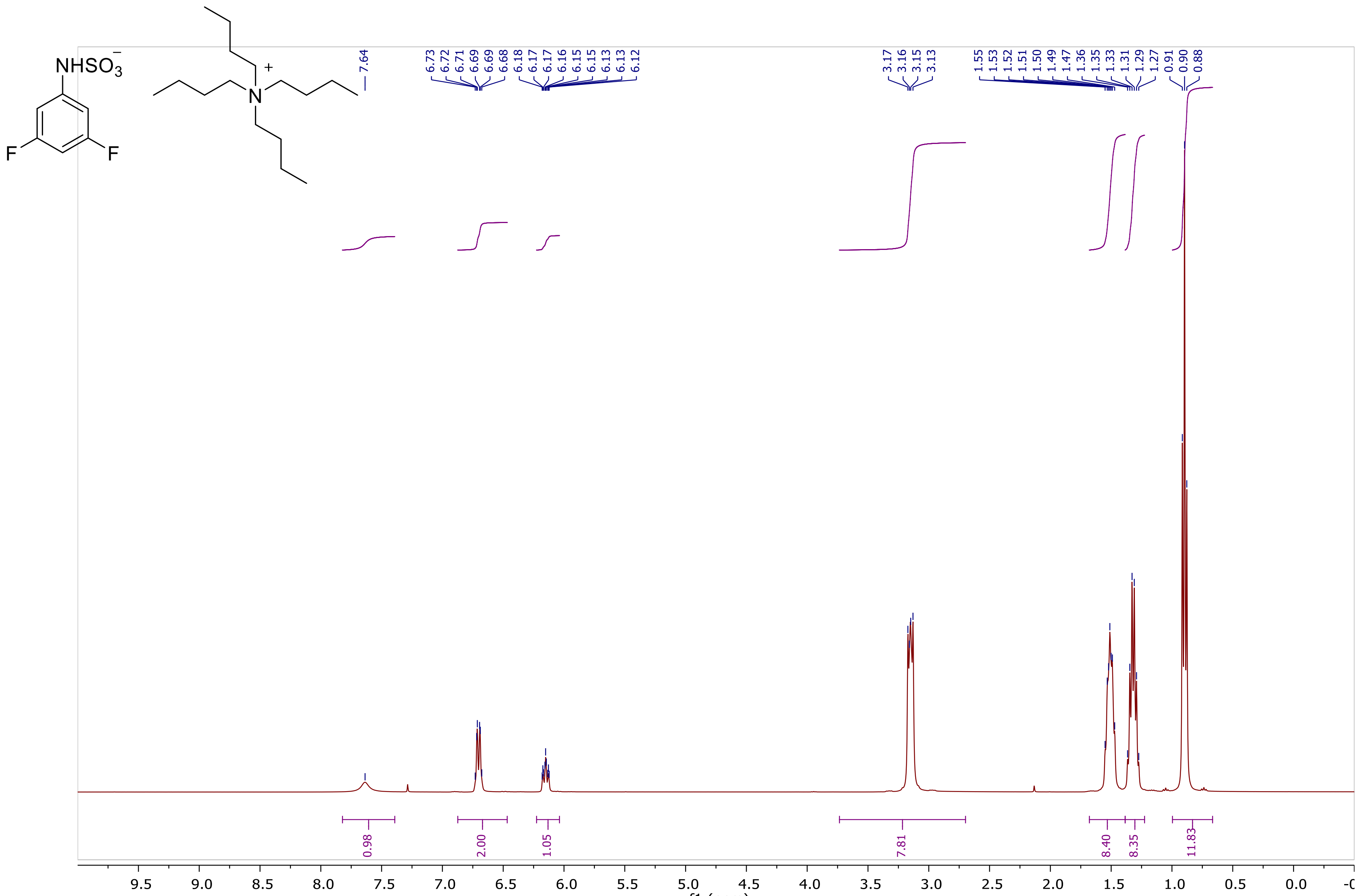

^{13}C NMR of tetrabutylammonium (3,5-difluorophenyl)sulfamate **1aa** in $CDCl_3$

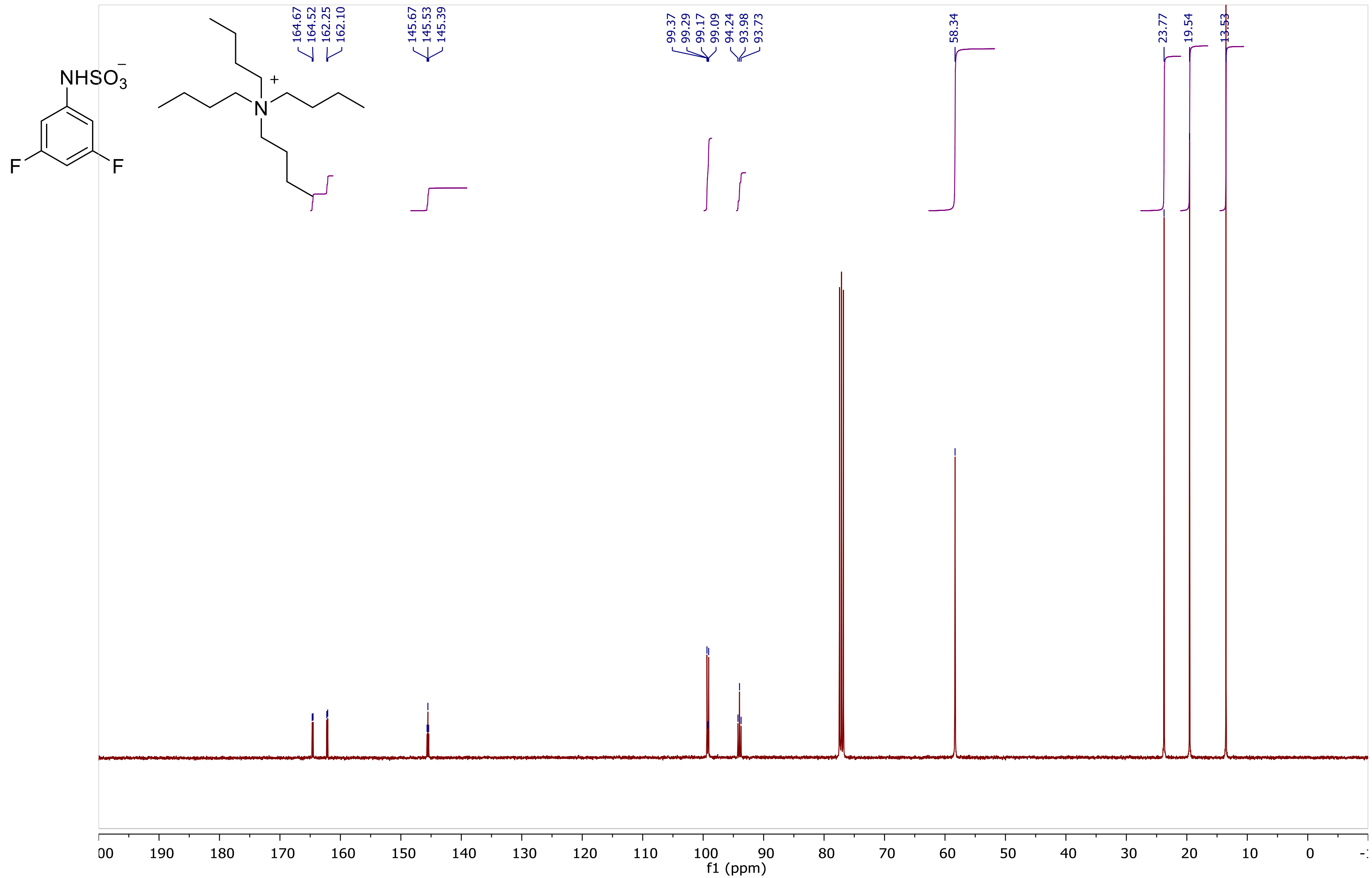

^{19}F NMR of tetrabutylammonium (3,5-difluorophenyl)sulfamate **1aa** in $CDCl_3$

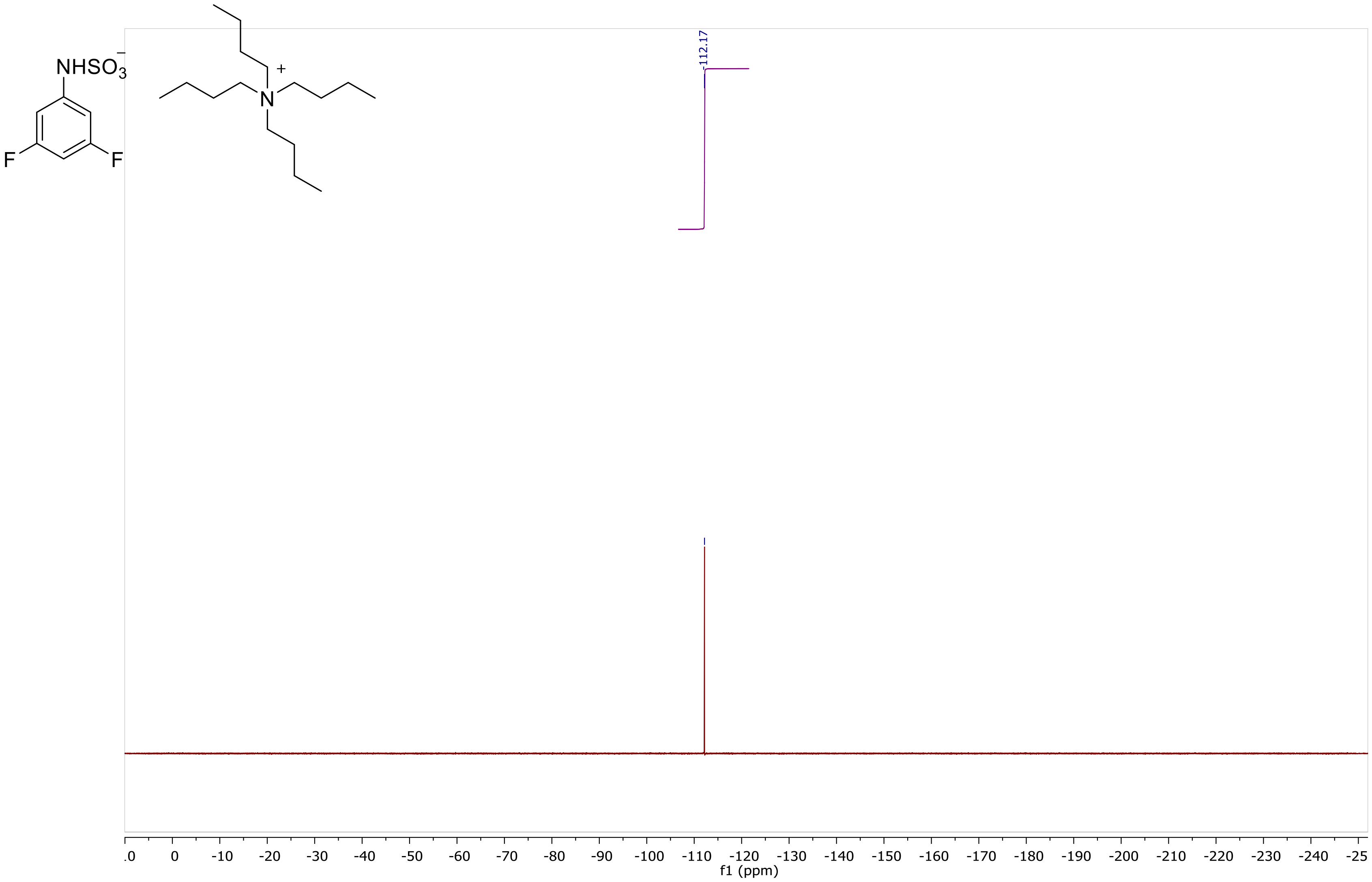

^{1}H NMR of tetrabutylammonium ((*N*-benzyl)phenyl)sulfamate **1ab** in $CDCl_3$

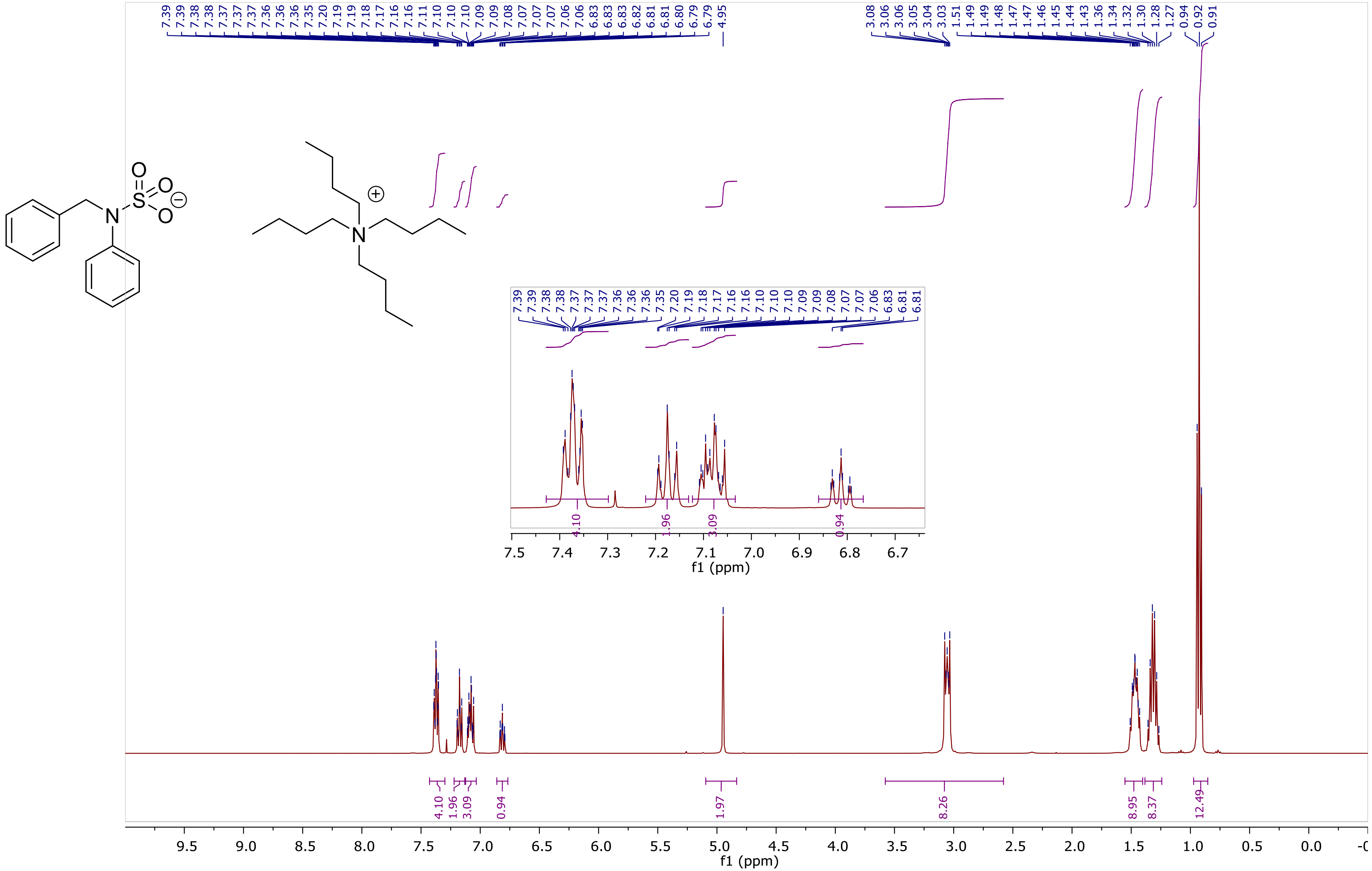

^{13}C NMR of tetrabutylammonium ((*N*-benzyl)phenyl)sulfamate **1ab** in $CDCl_3$

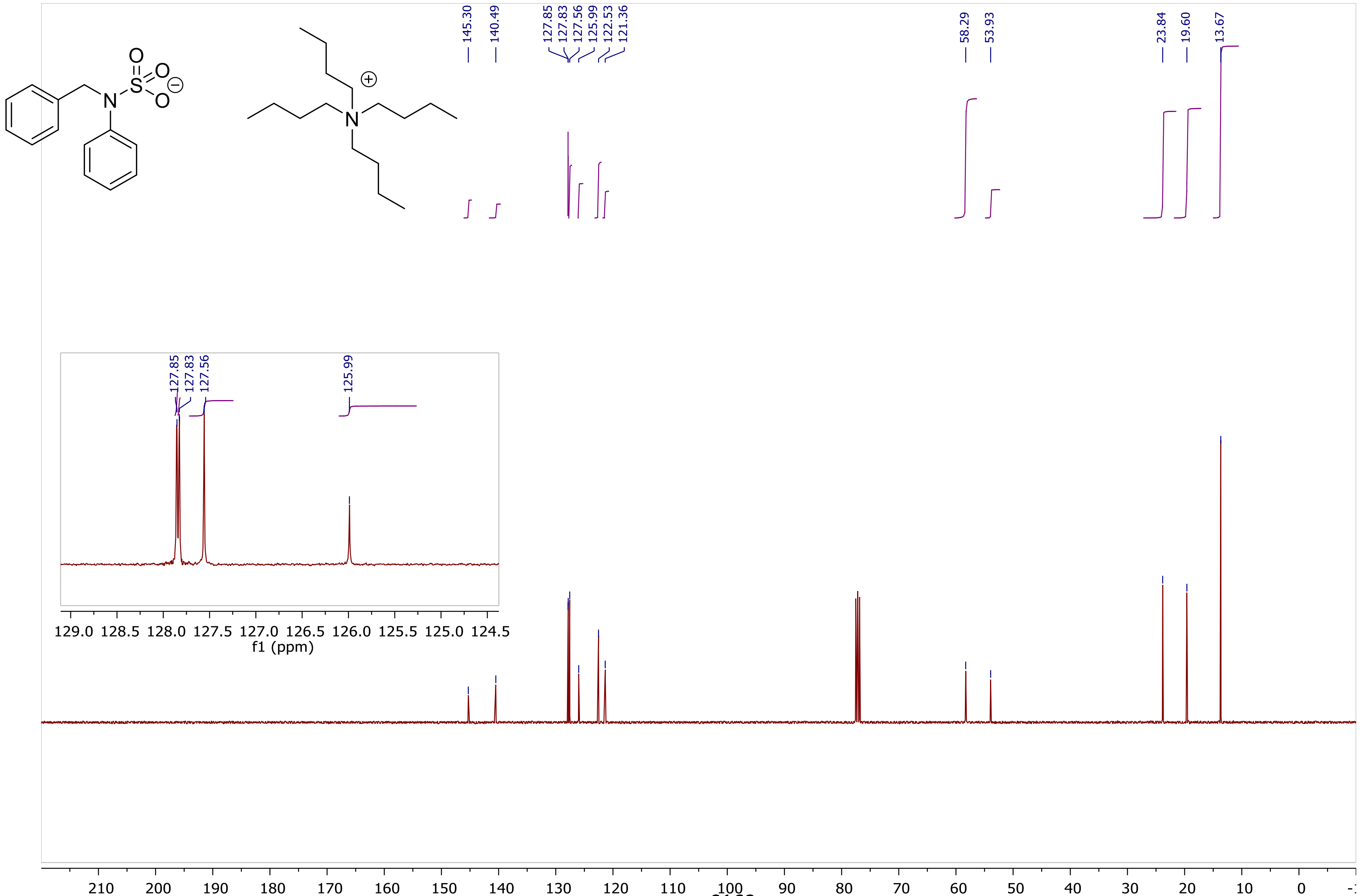

^{1}H NMR of tetrabutylammonium ((*N*-isopropyl)phenyl)sulfamate **1ac** in $CDCl_3$

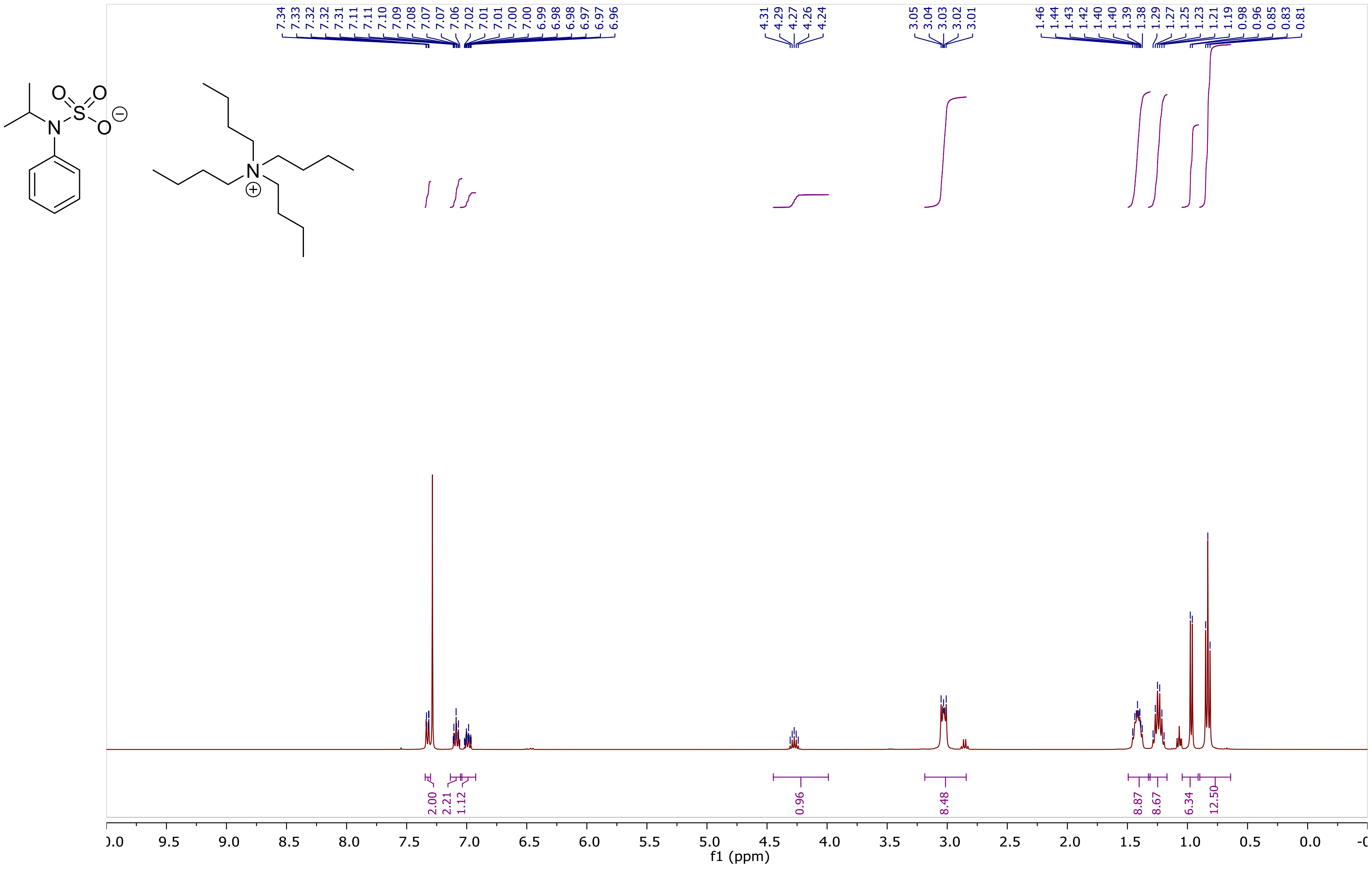

^{13}C NMR of tetrabutylammonium ((*N*-isopropyl)phenyl)sulfamate **1ac** in $CDCl_3$

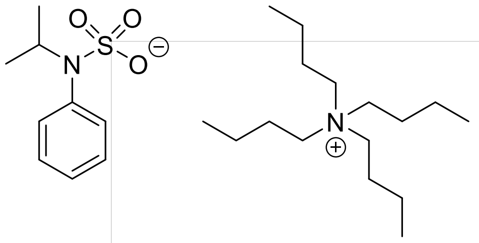

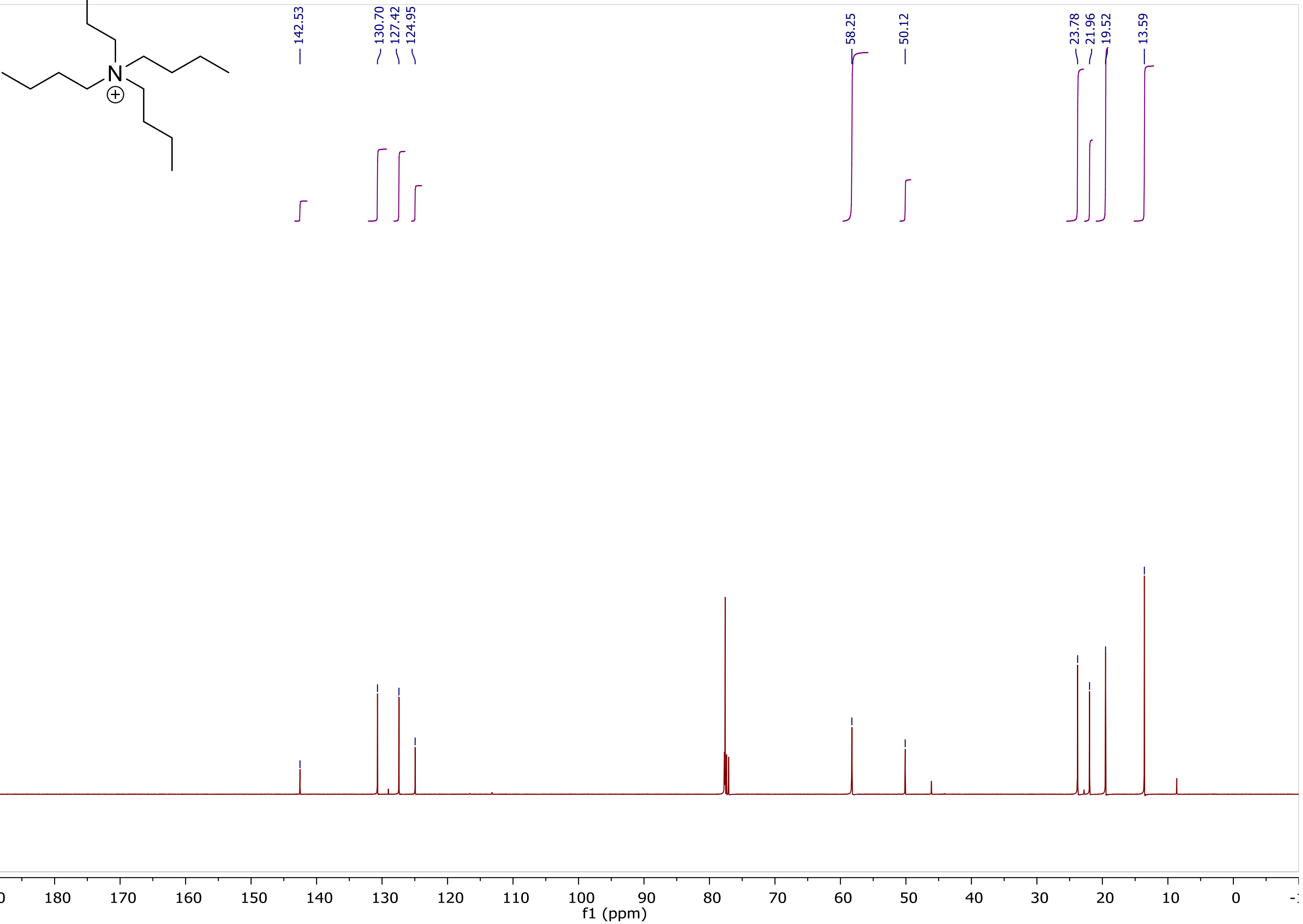

^{1}H NMR of tetrabutylammonium ((*N*-methyll)phenyl)sulfamate **1ad** in $CDCl_3$

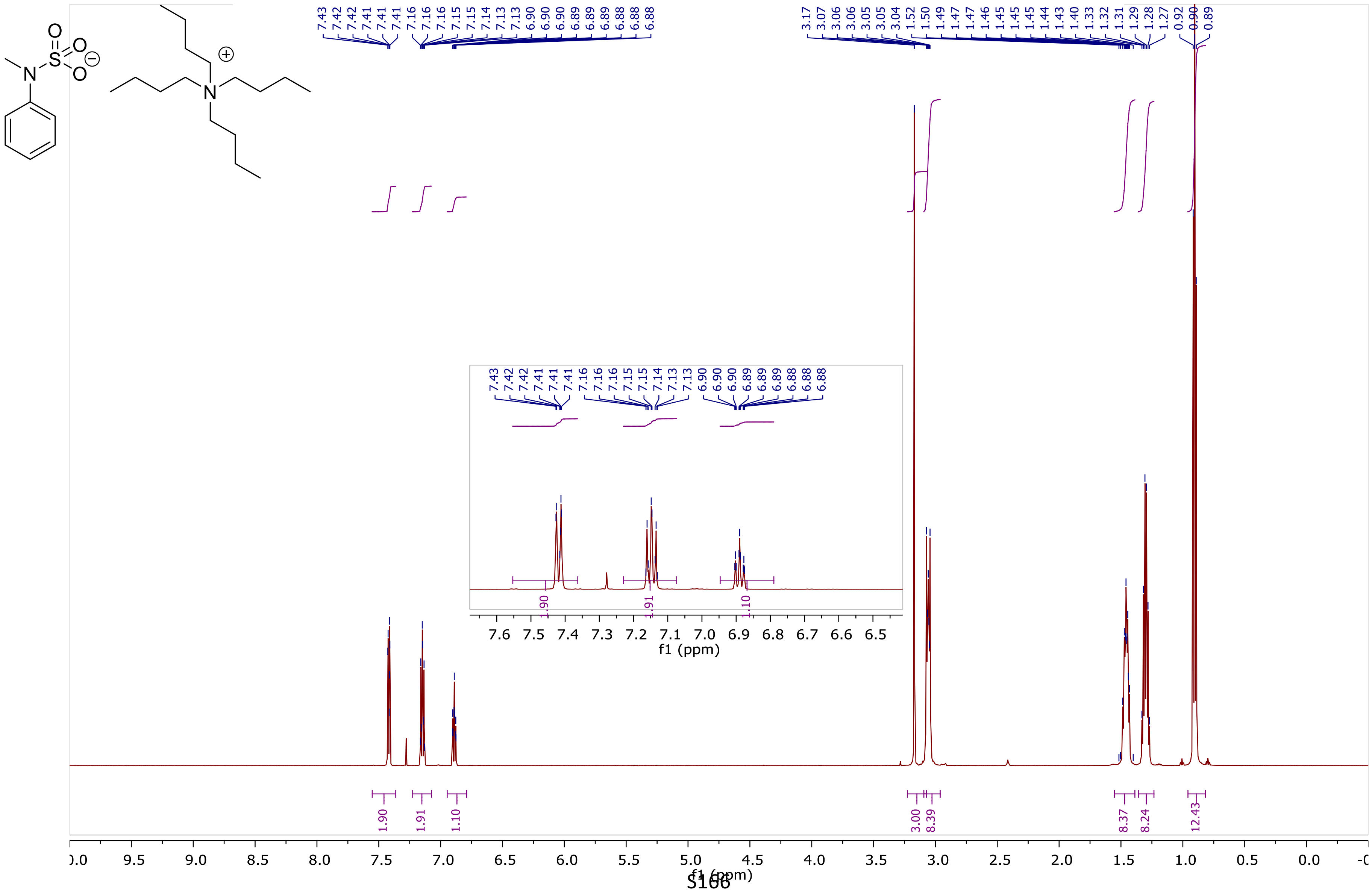

^{13}C NMR of tetrabutylammonium ((*N*-methyl)phenyl)sulfamate **1ad** in $CDCl_3$

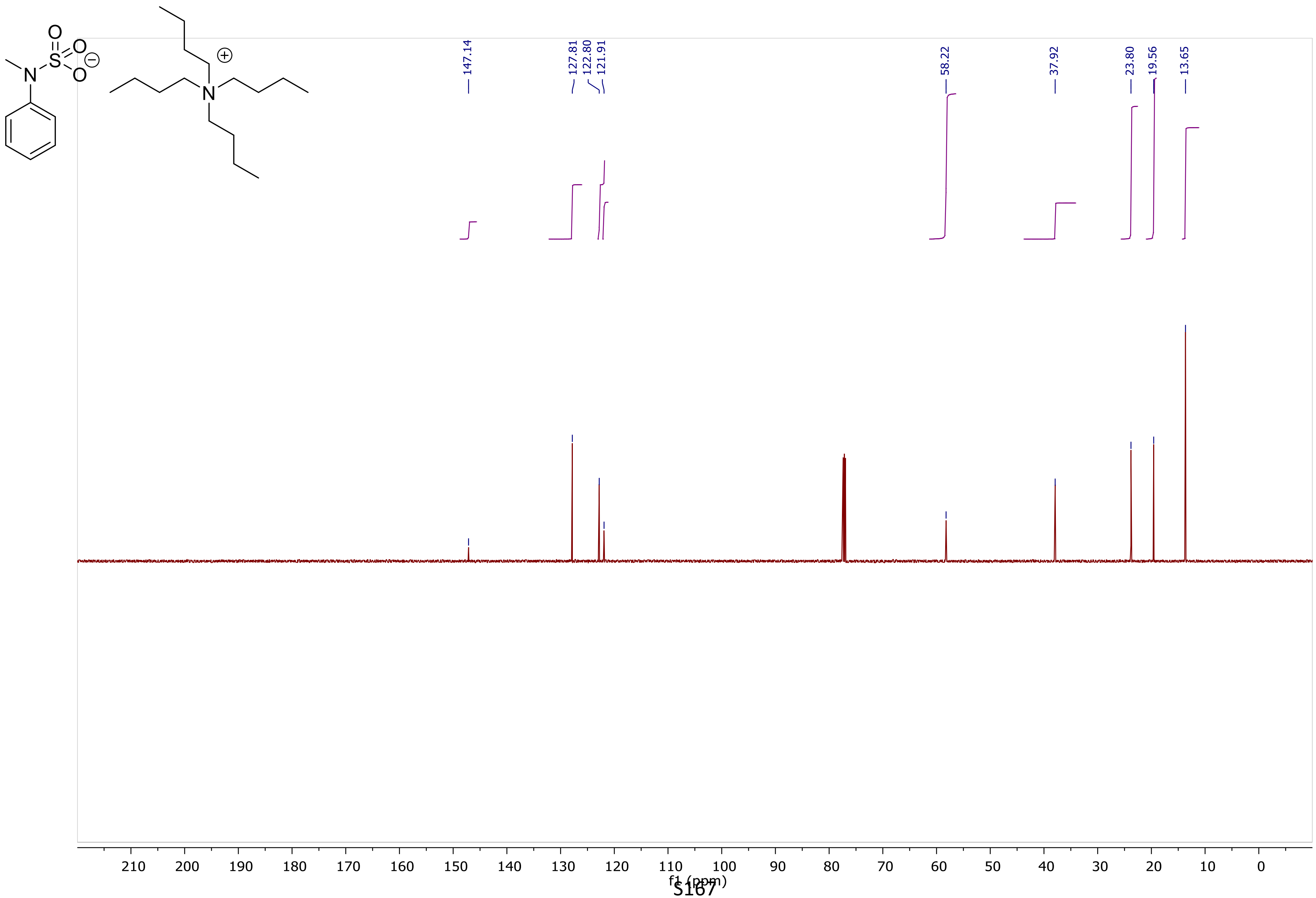

^{1}H NMR of *tert*-butyl (2,3-dimethylphenyl)carbamate in $CDCl_3$

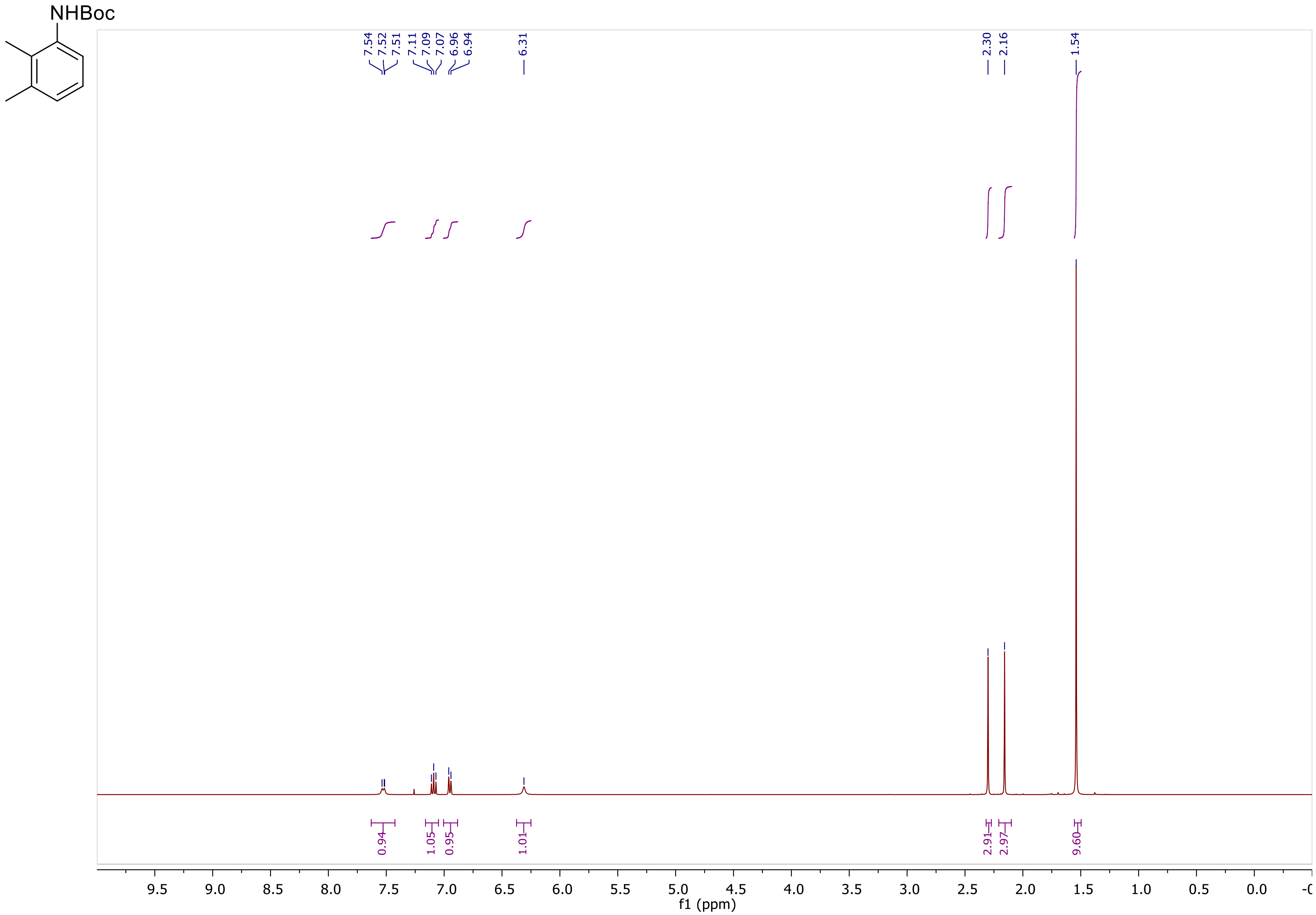

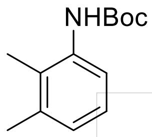

^{13}C NMR of *tert*-butyl (2,3-dimethylphenyl)carbamate in $CDCl_3$

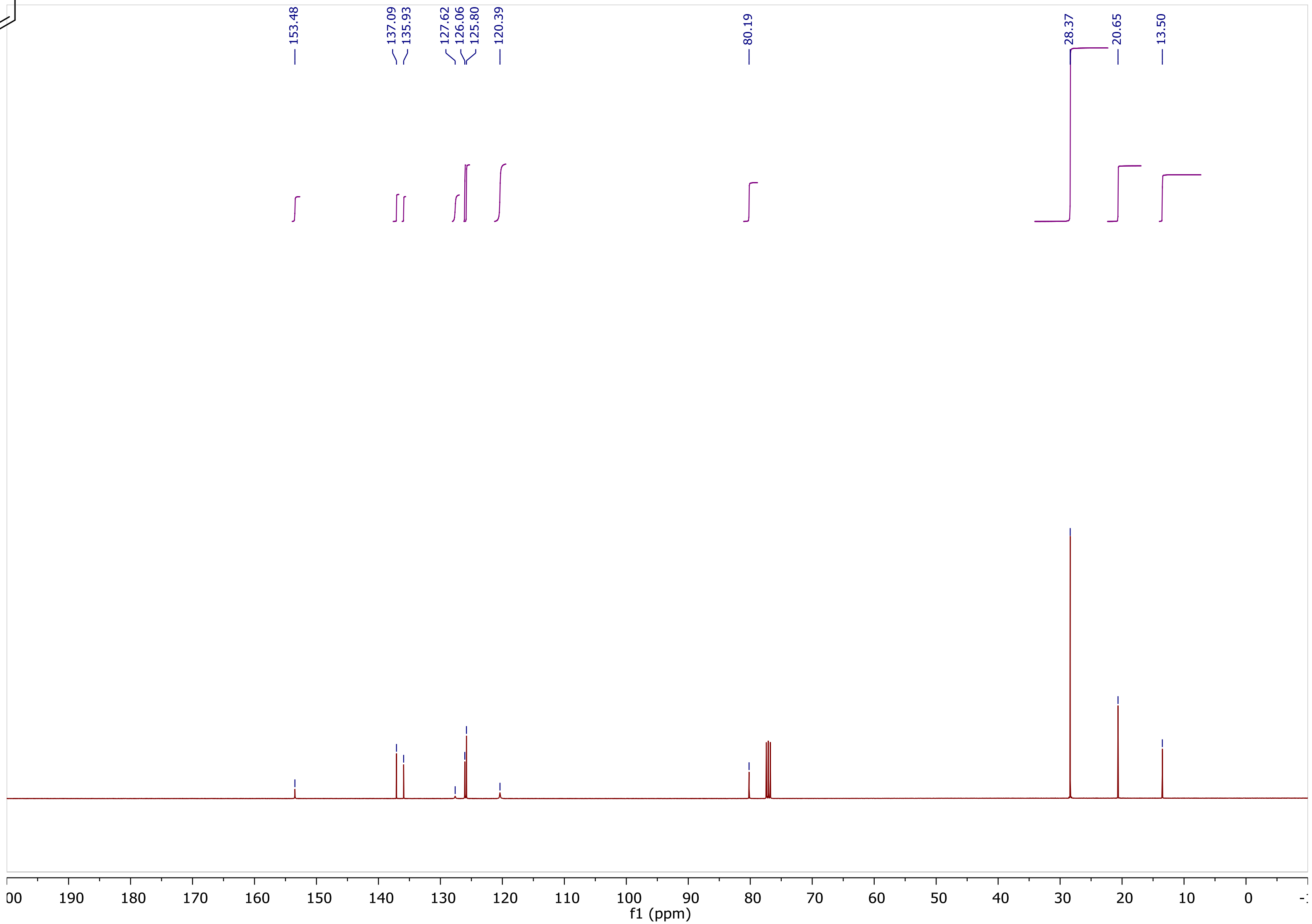

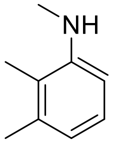

^{1}H NMR of *N*,2,3 trimethylaniline in $CDCl_3$

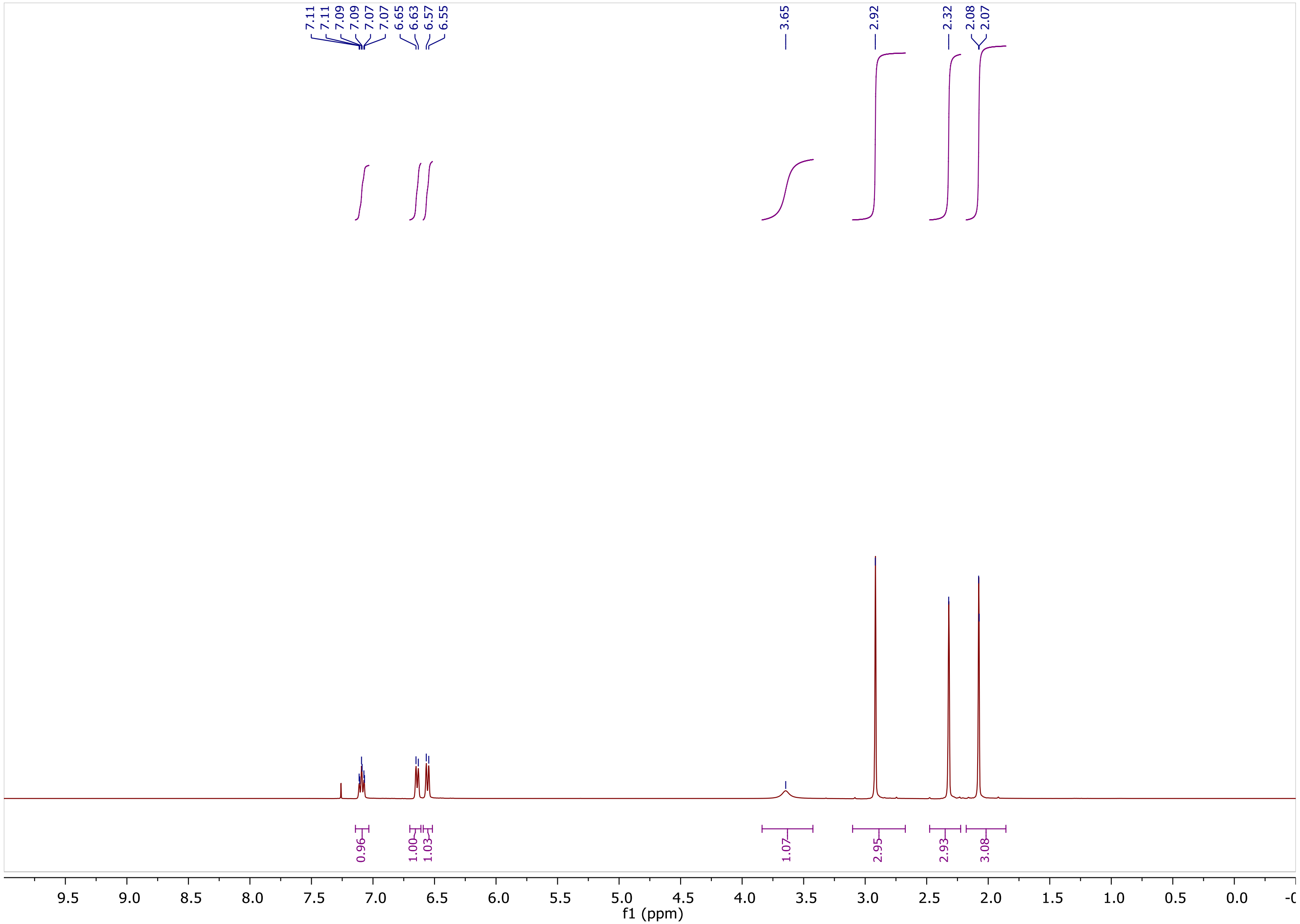

^{1}H NMR of *N*,2,3 trimethylaniline in $CDCl_3$

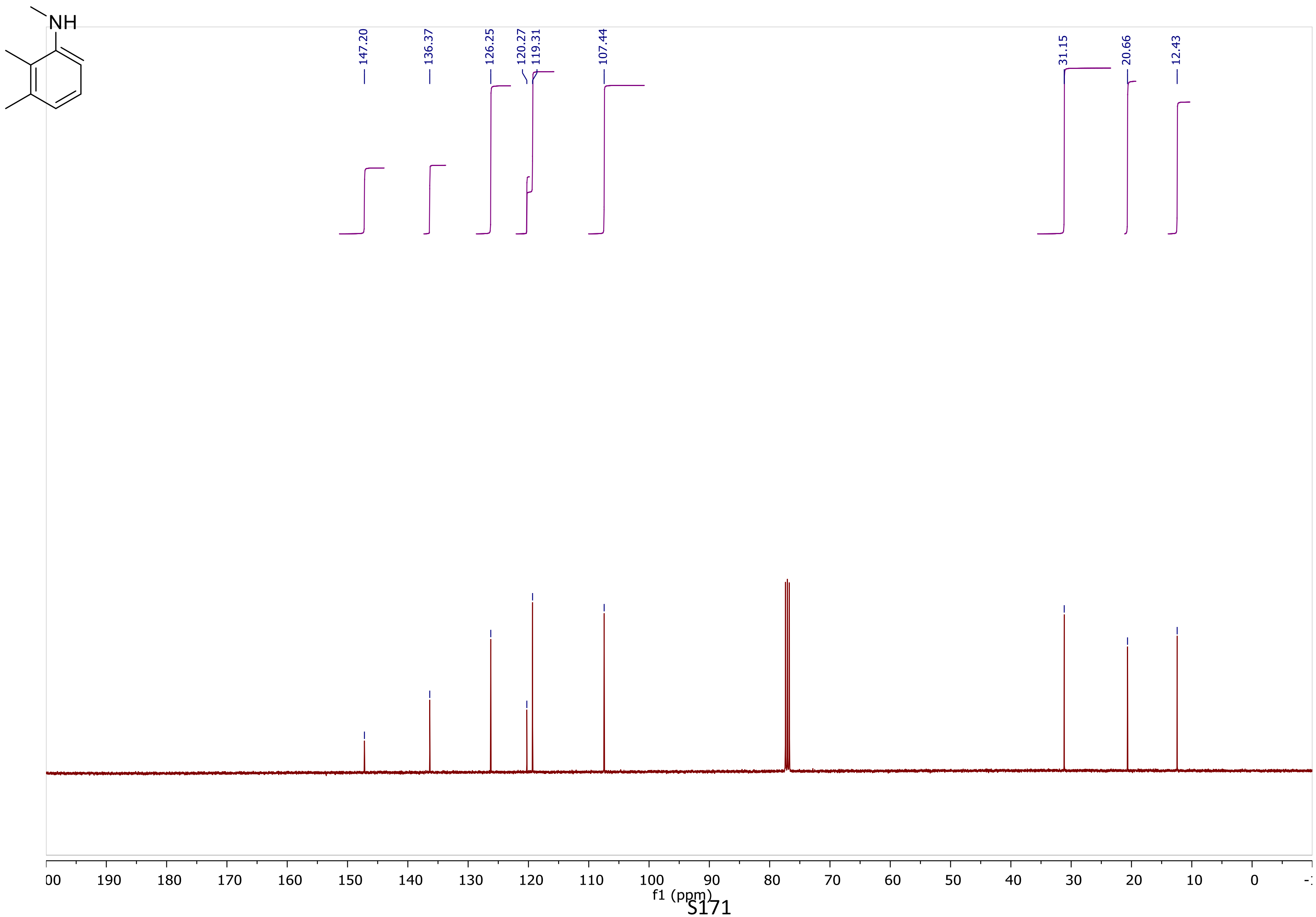

^{1}H NMR of tetrabutylammonium *N*-methyl(2,3-dimethyl)phenylsulfamate **1ae** in $CDCl_3$

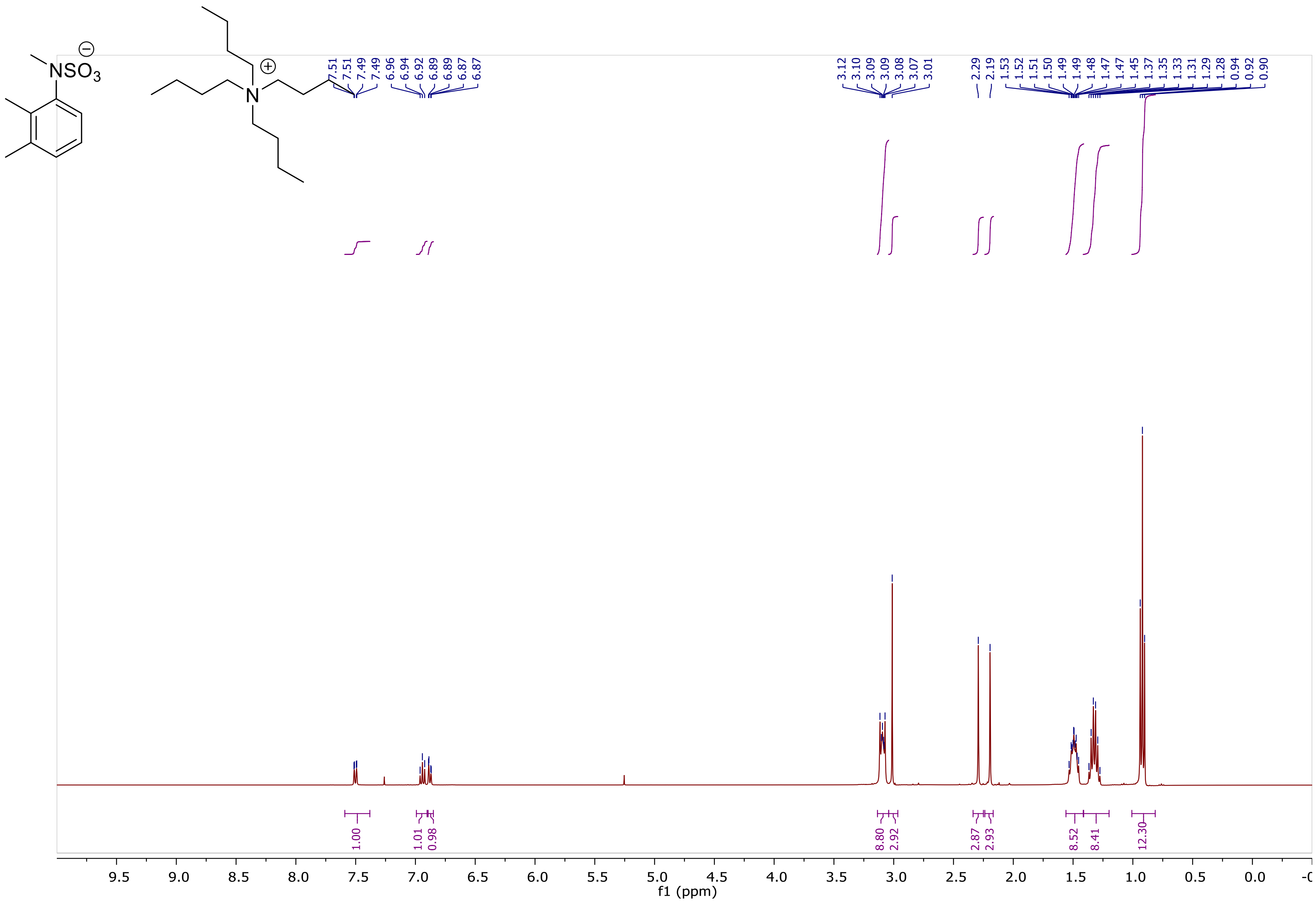

^{13}C NMR of tetrabutylammonium *N*-methyl(2,3-dimethyl)phenylsulfamate **1ae** in $CDCl_3$

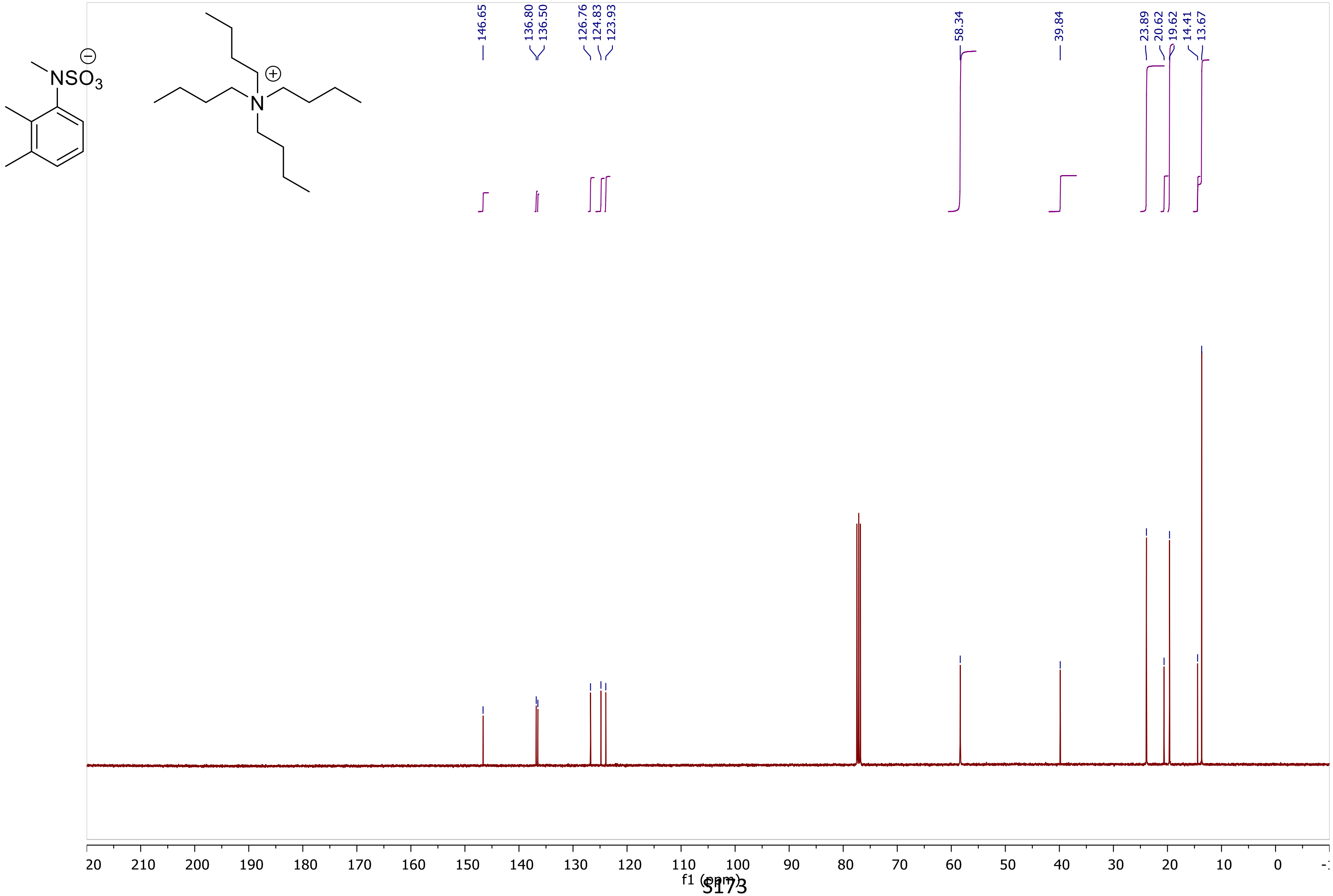

^1H NMR of 1-nitro-2-vinylbenzene in $CDCl_3$

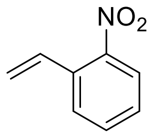

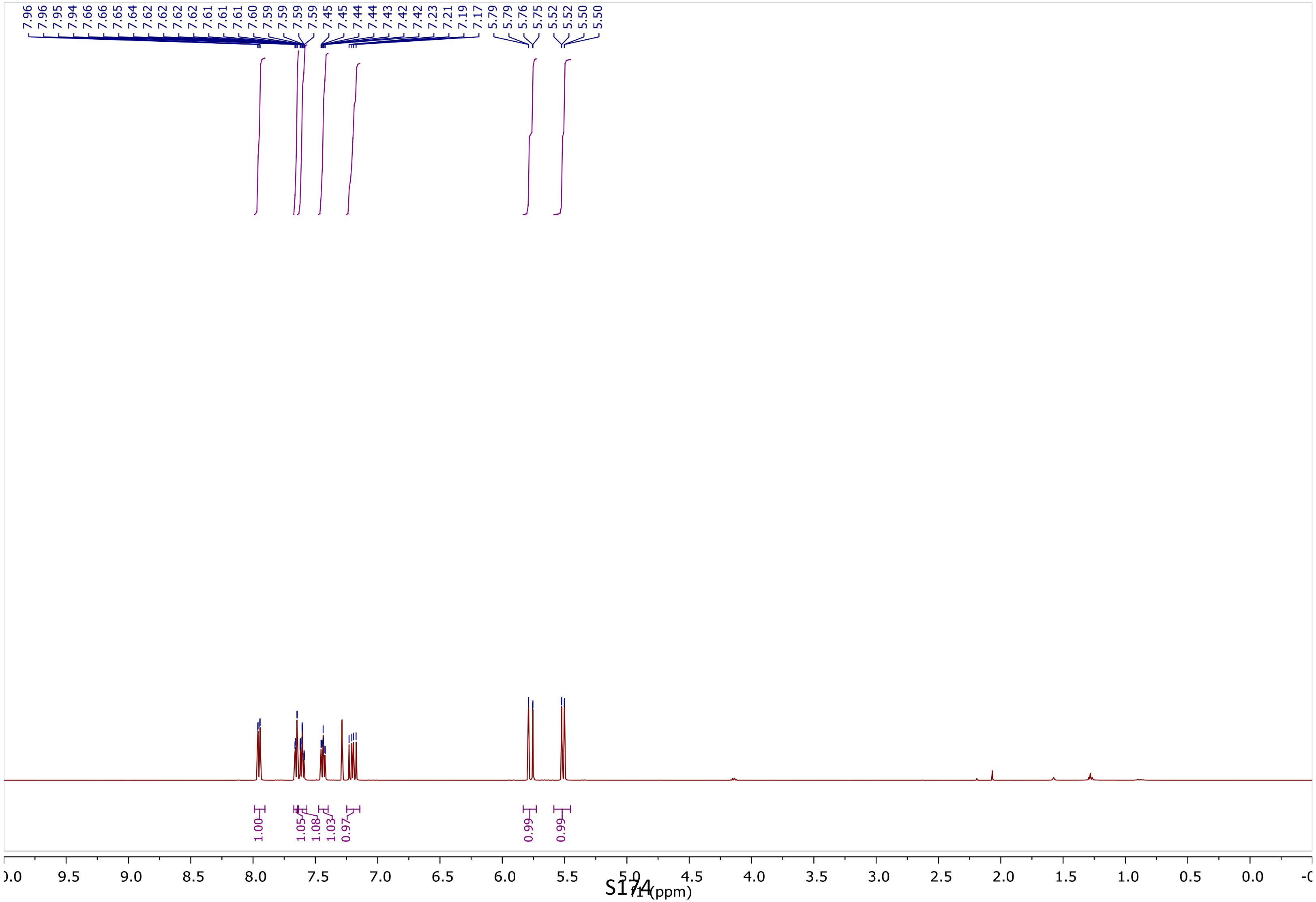

^{13}C NMR of 1-nitro-2-vinylbenzene in $CDCl_3$

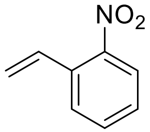

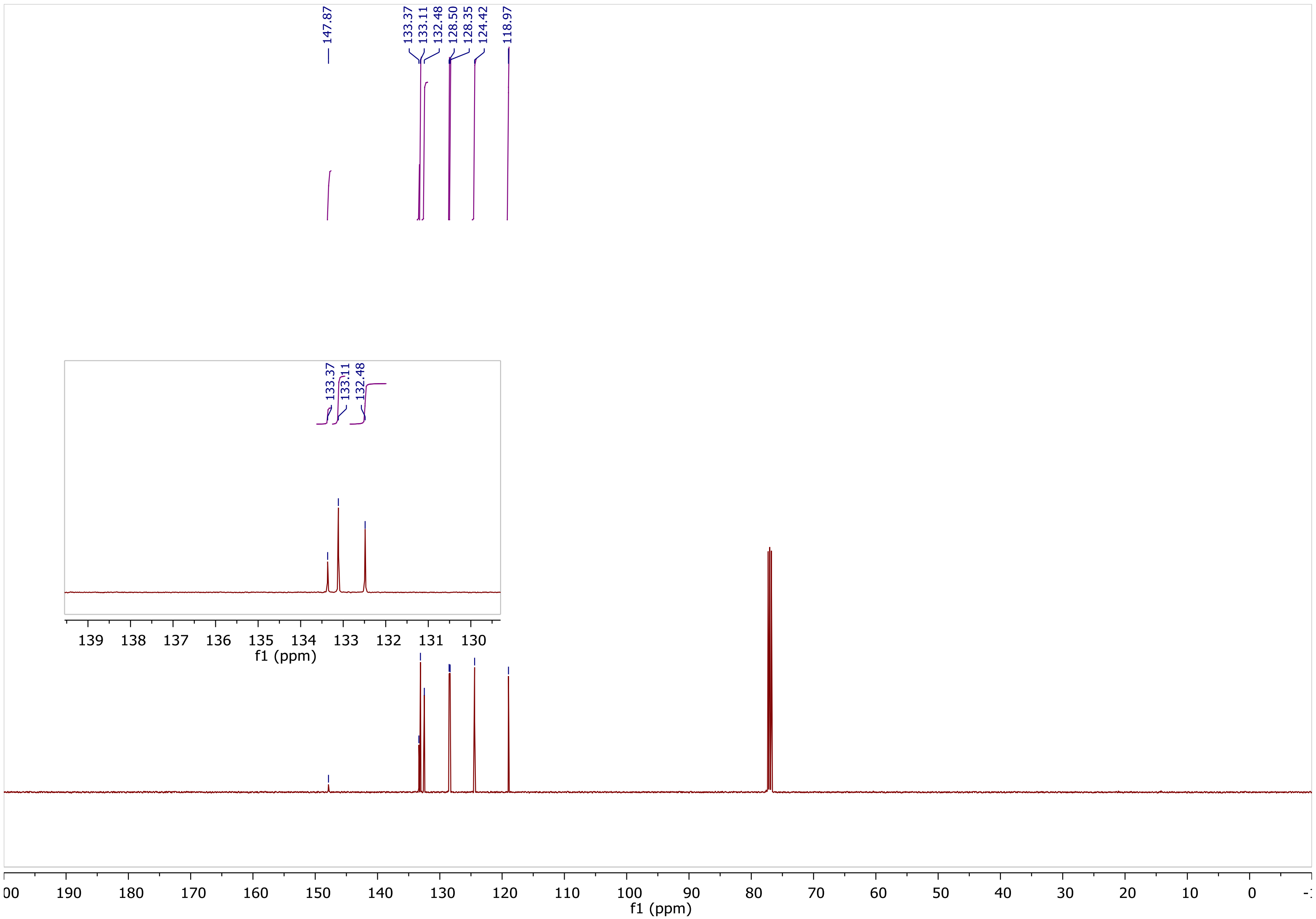

^{1}H NMR of 2-vinylaniline in $CDCl_3$

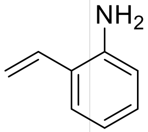

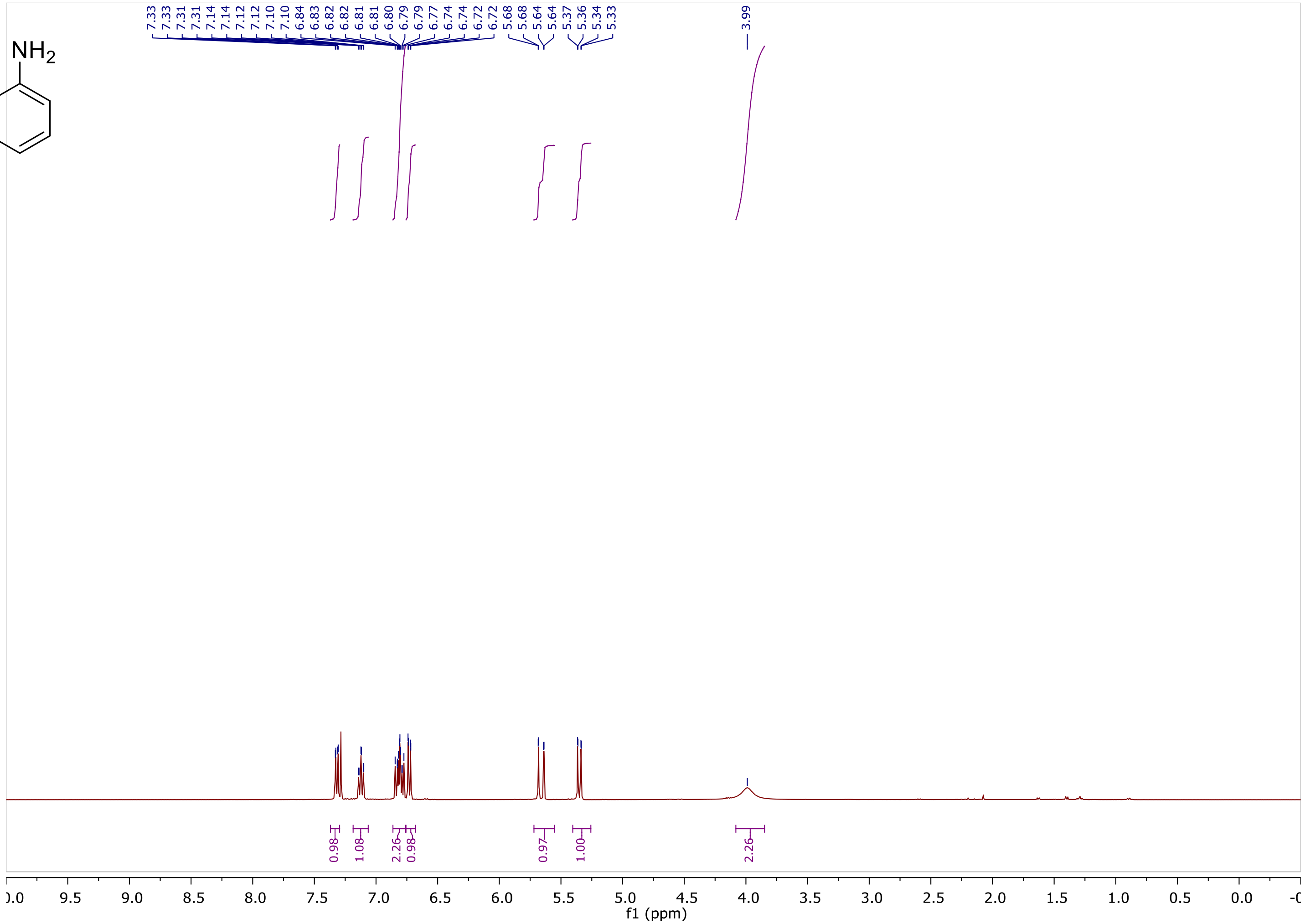

^{13}C NMR of 2-vinylaniline in $CDCl_3$

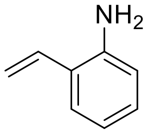

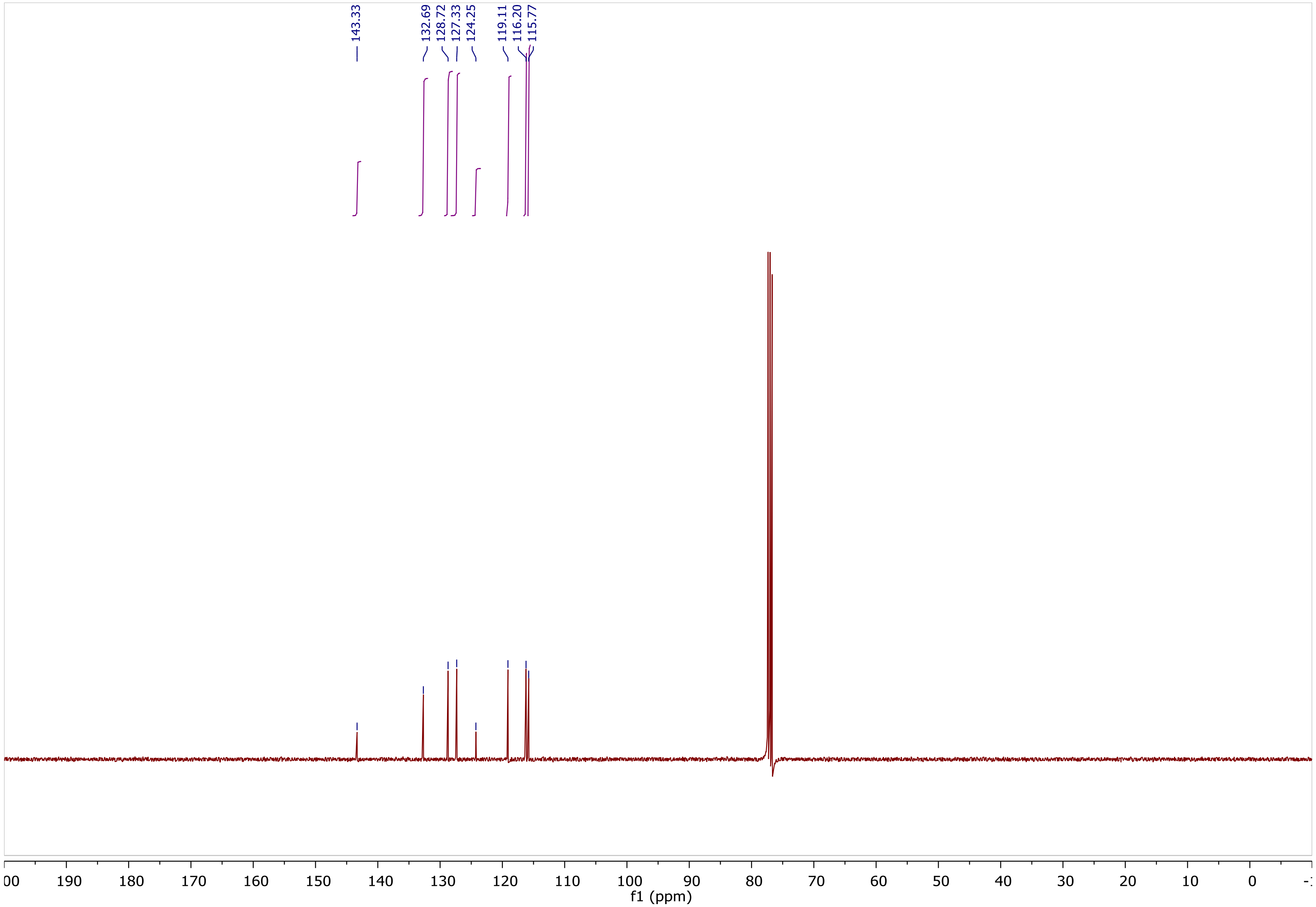

^{1}H NMR of tetrabutylammonium (2-vinylphenyl)sulfamate in MeOD-d4

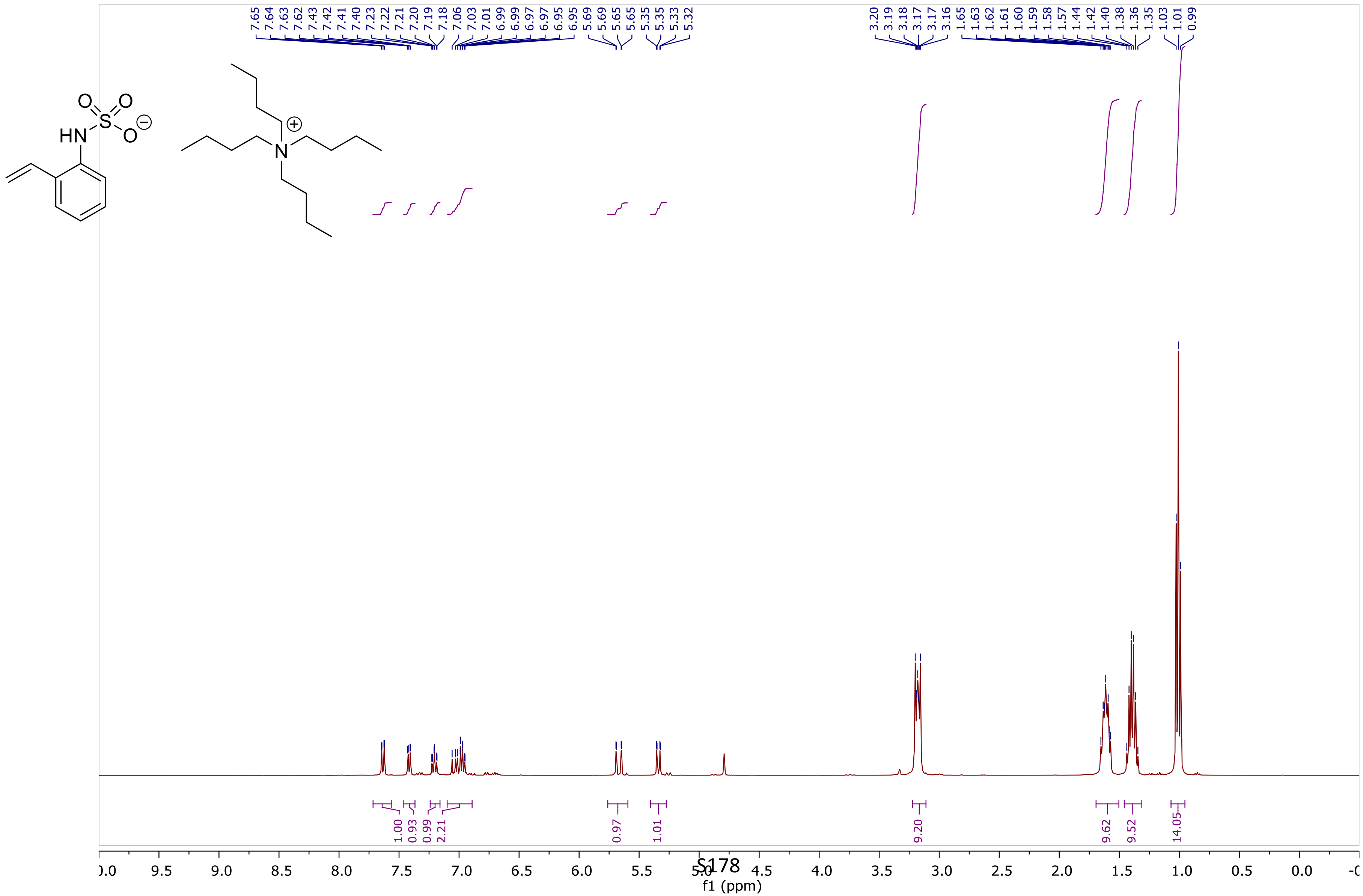

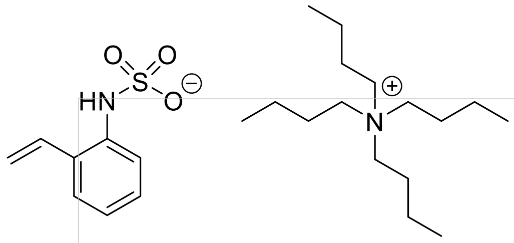

^{13}C NMR of tetrabutylammonium (2-vinylphenyl)sulfamate in MeOD-d4

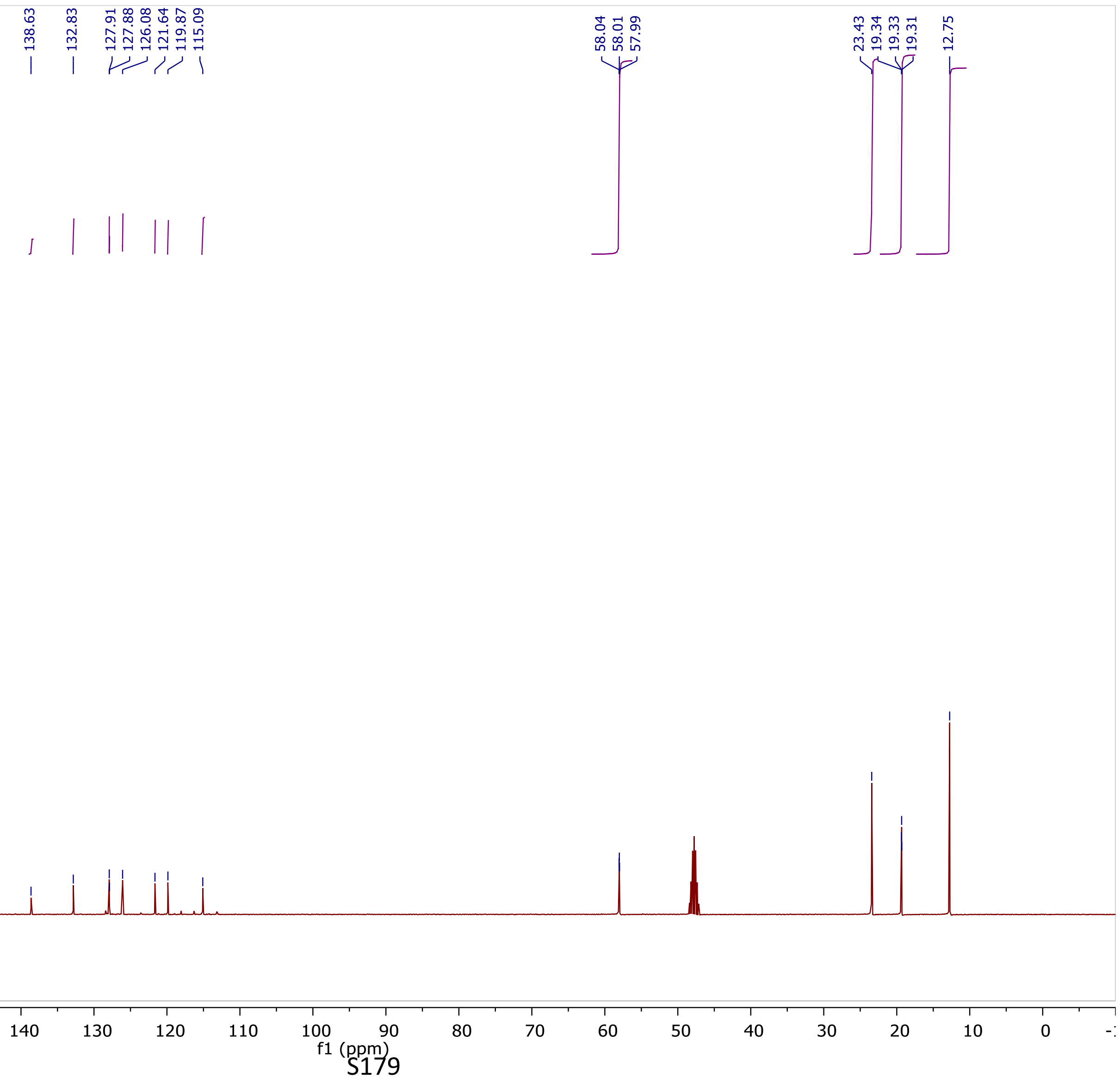

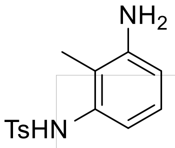

^{1}H NMR of N-(3-amino-2-methylphenyl)-4-methylbenzenesulfonamide in $CDCl_3$

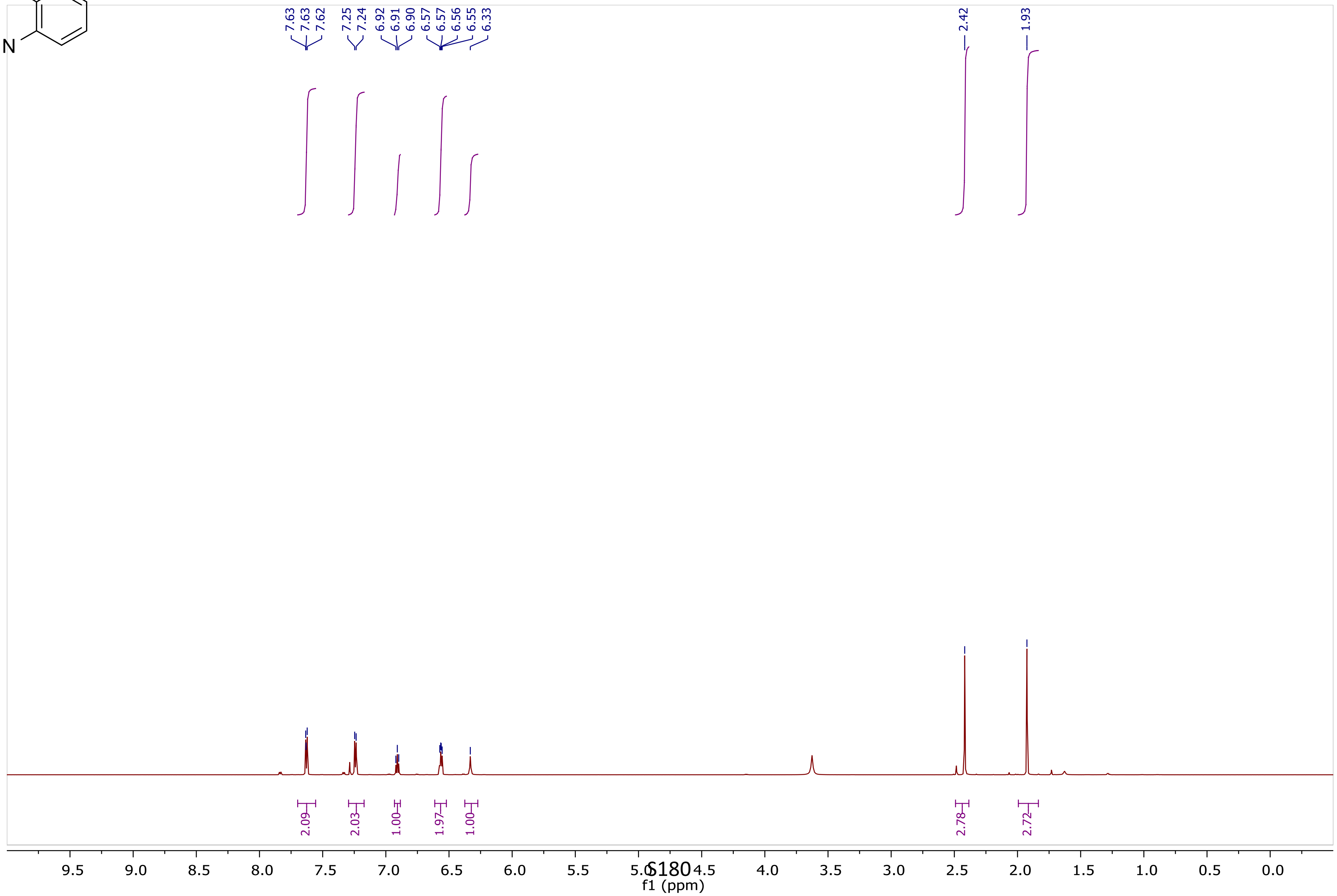

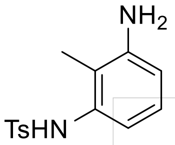

^{13}C NMR of N-(3-amino-2-methylphenyl)-4-methylbenzenesulfonamide in $CDCl_3$

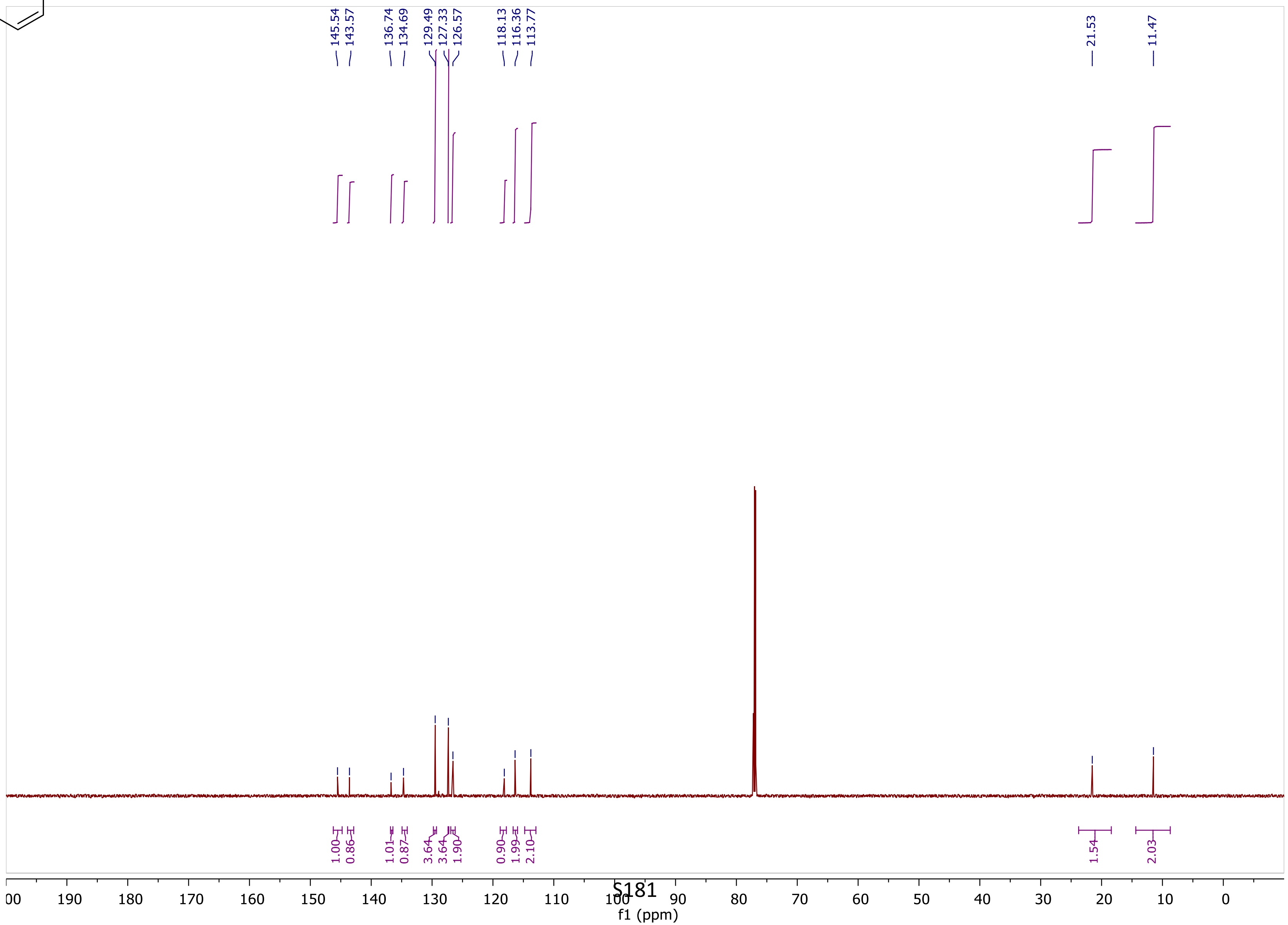

^{1}H NMR of *N*-(3-amino-2-methylphenyl)-*N*,4-dimethylbenzenesulfonamide in $CDCl_3$

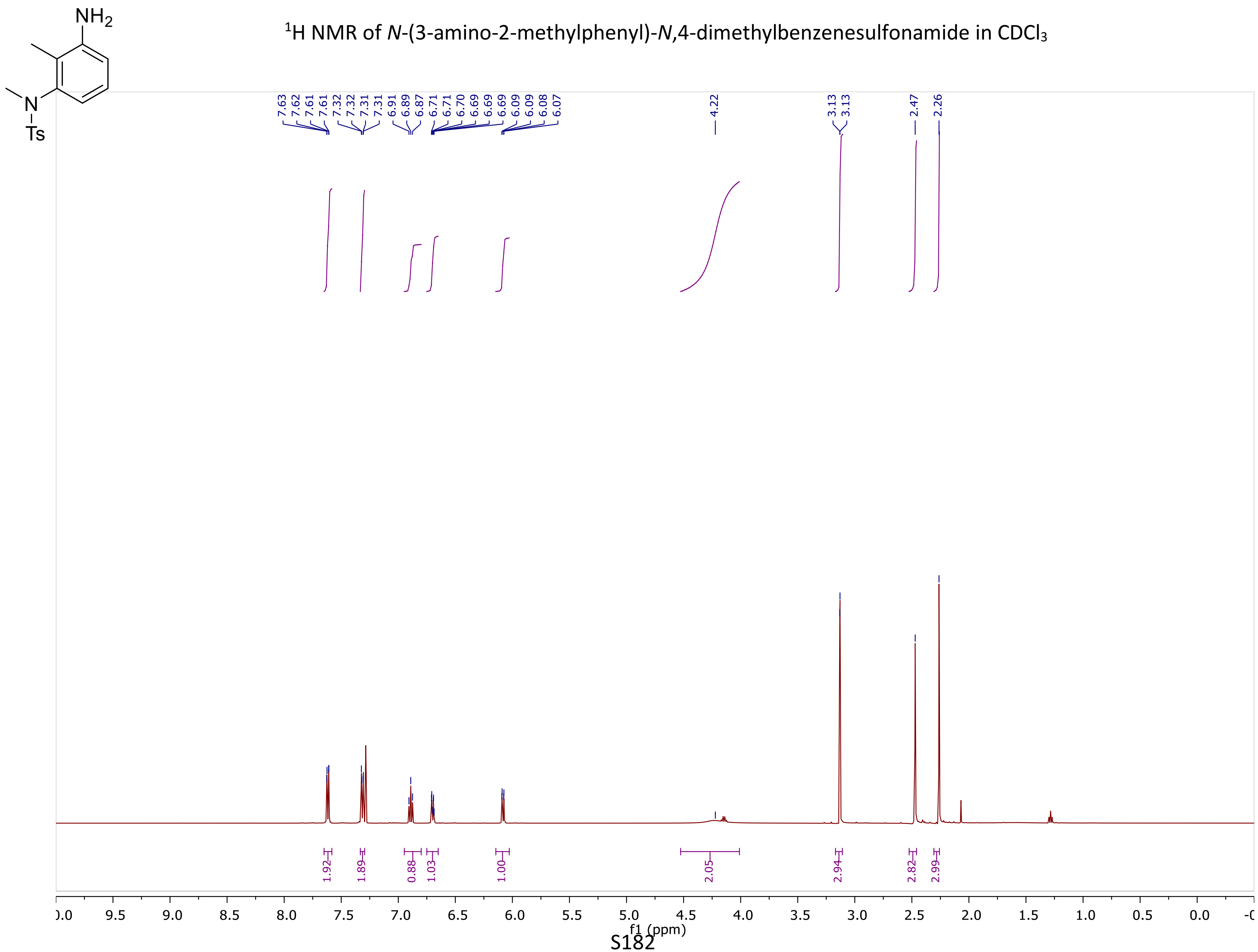

^{13}C NMR of *N*-(3-amino-2-methylphenyl)-*N*,4-dimethylbenzenesulfonamide in $CDCl_3$

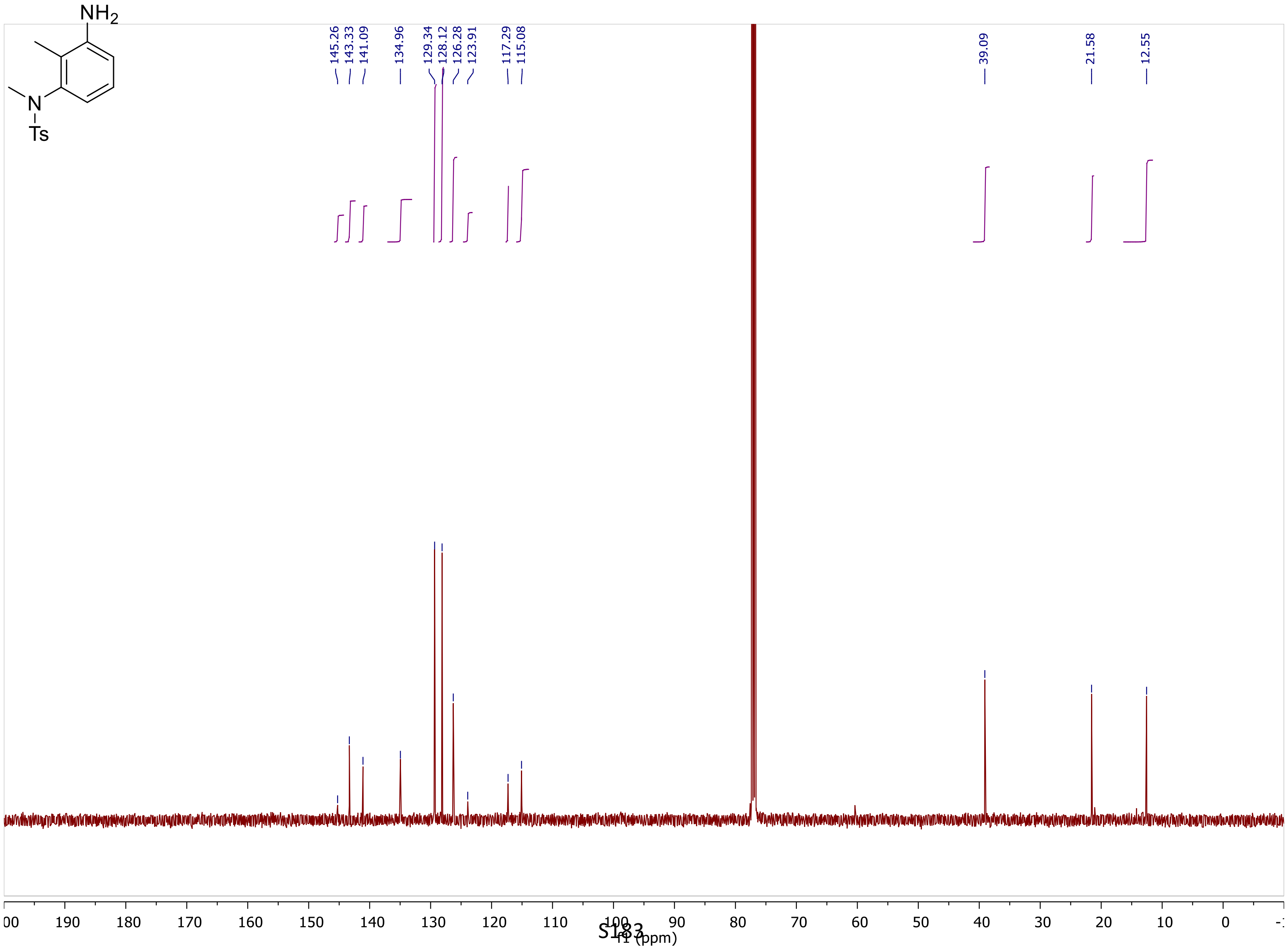

^{1}H NMR of tetrabutylammonium (3-((N,4-dimethylphenyl)sulfonamido)-2-methylphenyl)sulfamate in MeOD-d4

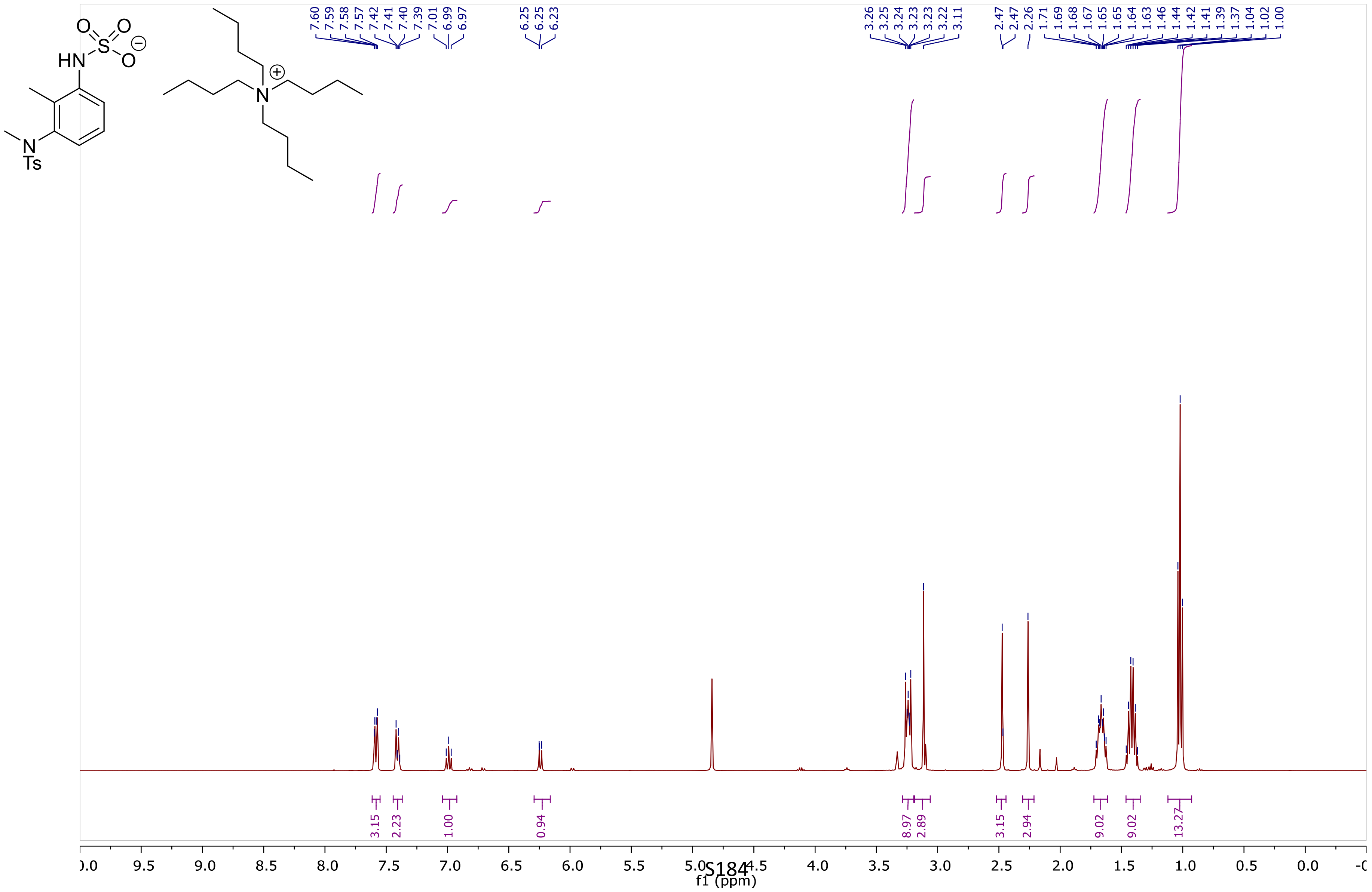

^{13}C NMR of tetrabutylammonium (3-((N,4-dimethylphenyl)sulfonamido)-2-methylphenyl)sulfamate in MeOD-d4

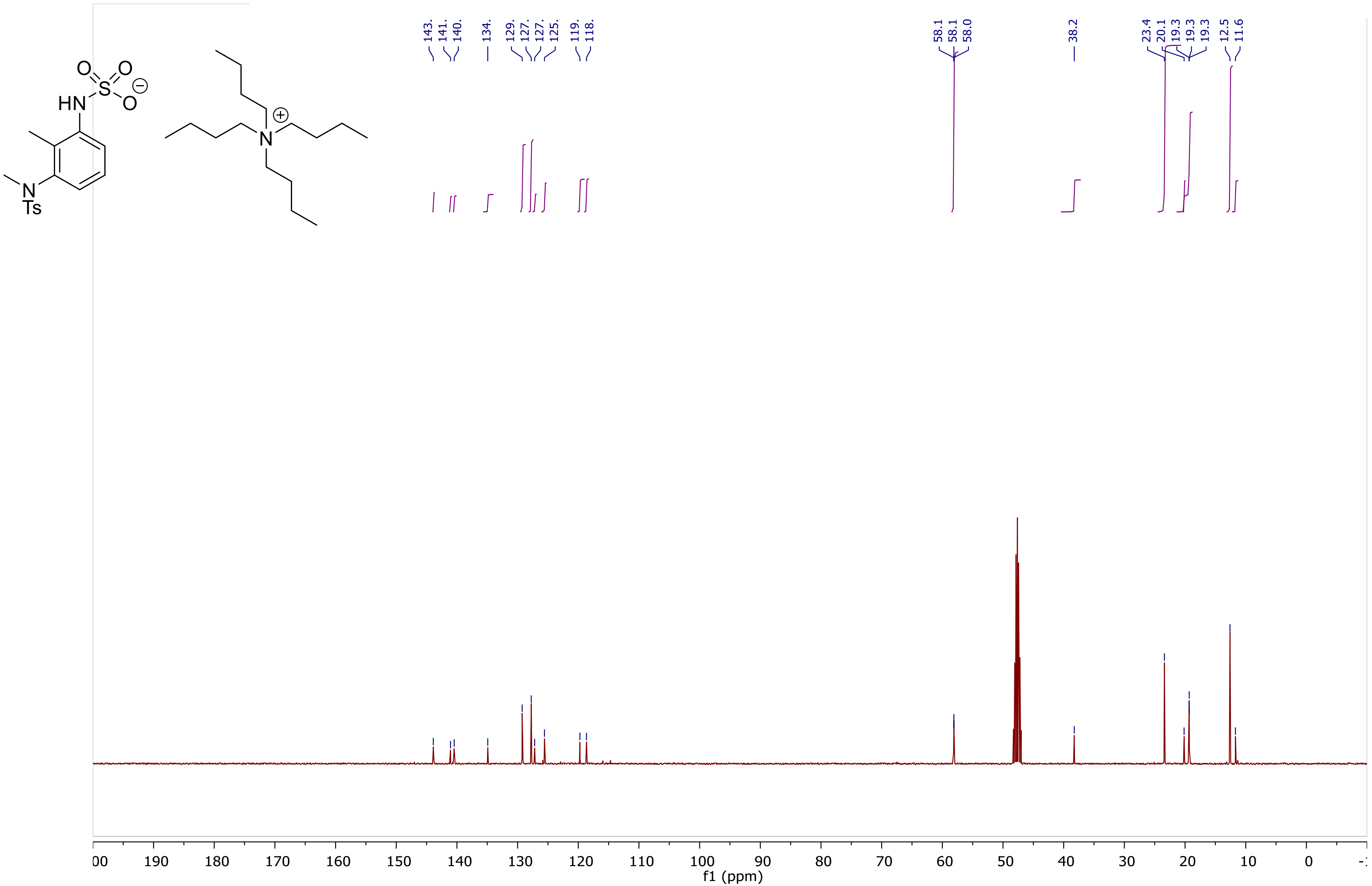

^{1}H NMR of tetrabutylammonium diphenylsulfamate in $CDCl_3$

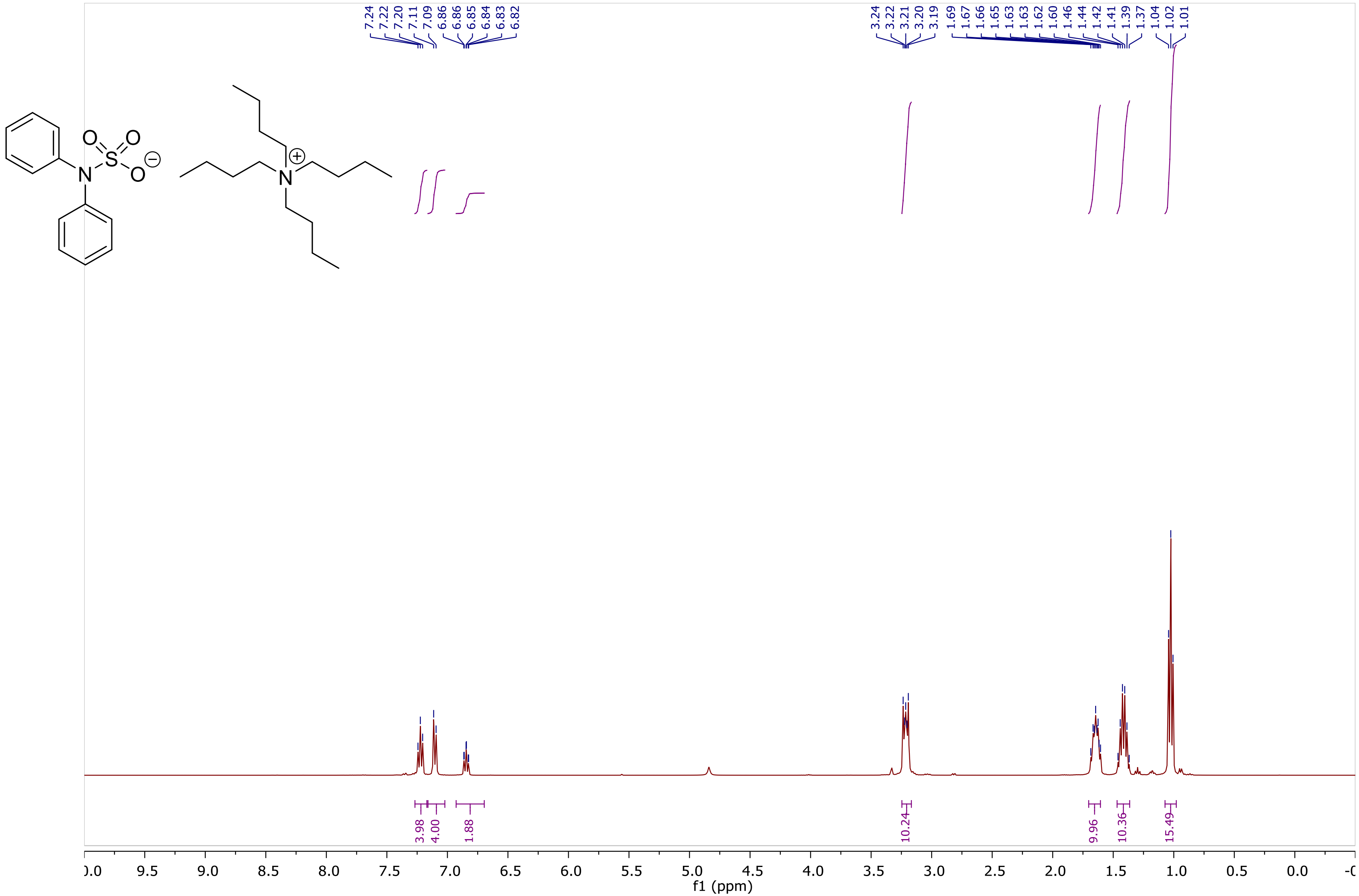

^{13}C NMR of tetrabutylammonium diphenylsulfamate in $CDCl_3$

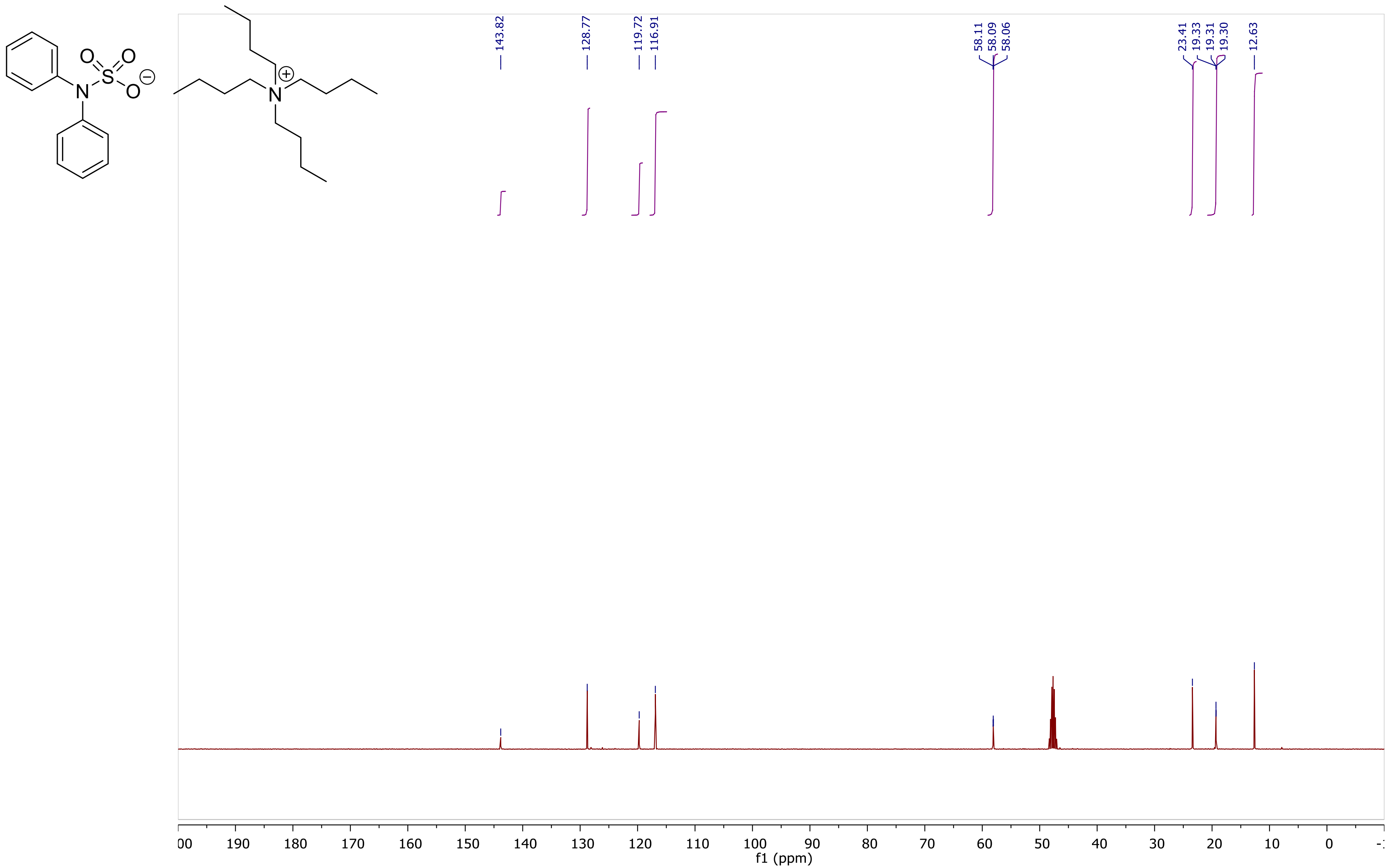

^{1}H NMR of tetrabutylammonium naphthalen-1-ylsulfamate in $CDCl_3$

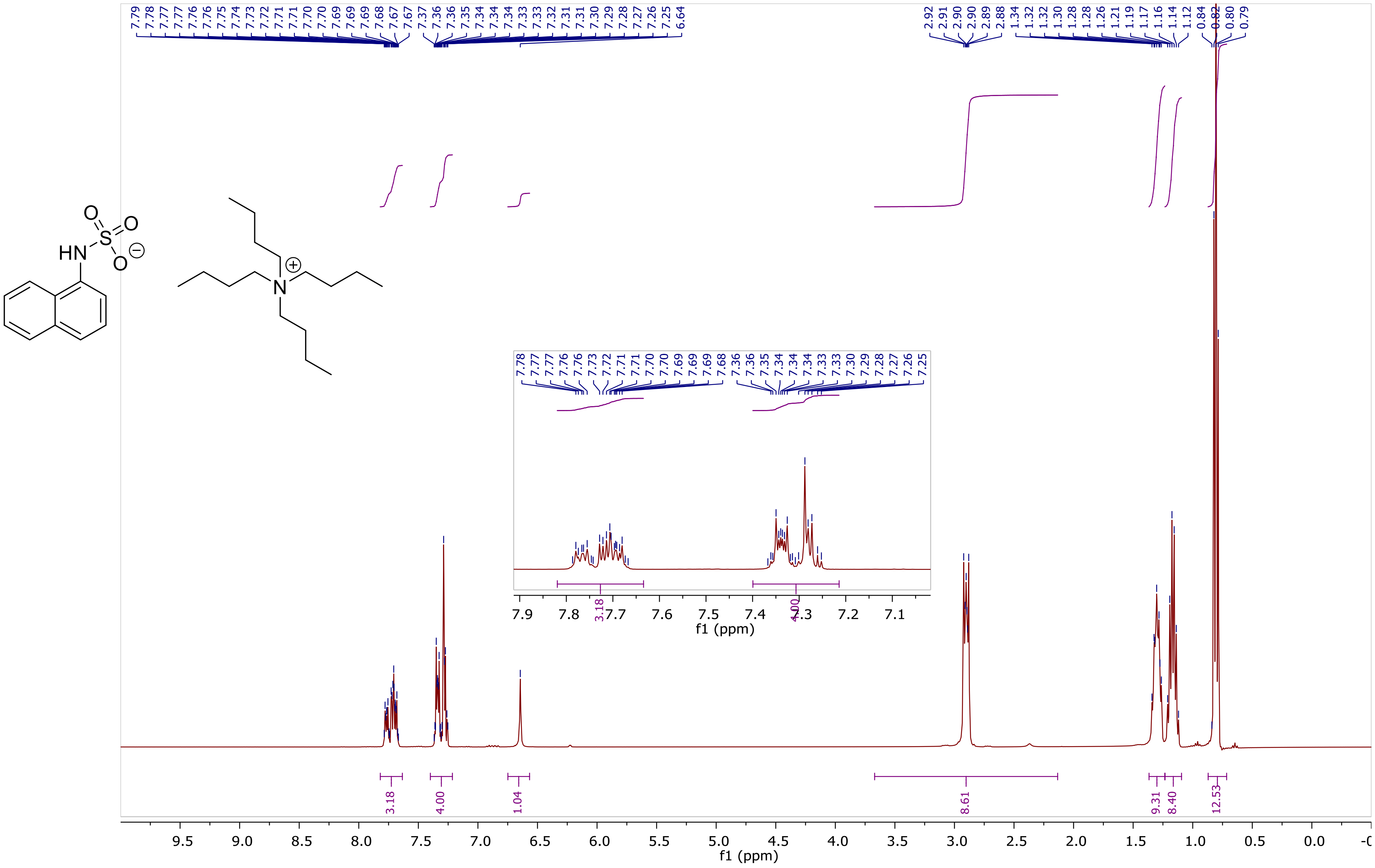

^{13}C NMR of tetrabutylammonium naphthalen-1-ylsulfamate in $CDCl_3$

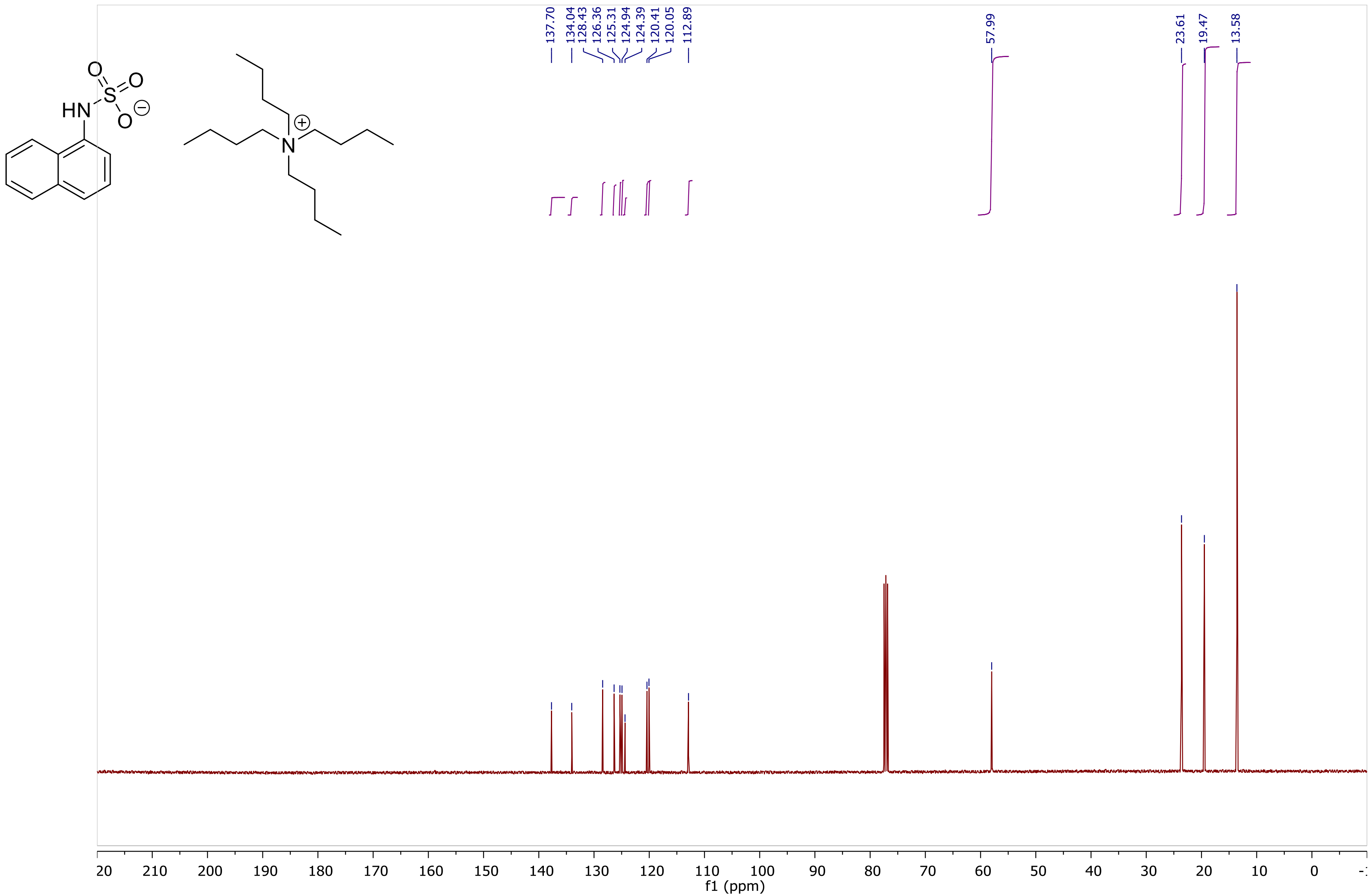

^{1}H NMR of tetrabutylammonium (2-(trifluoromethyl)phenyl)sulfamate in $CDCl_3$

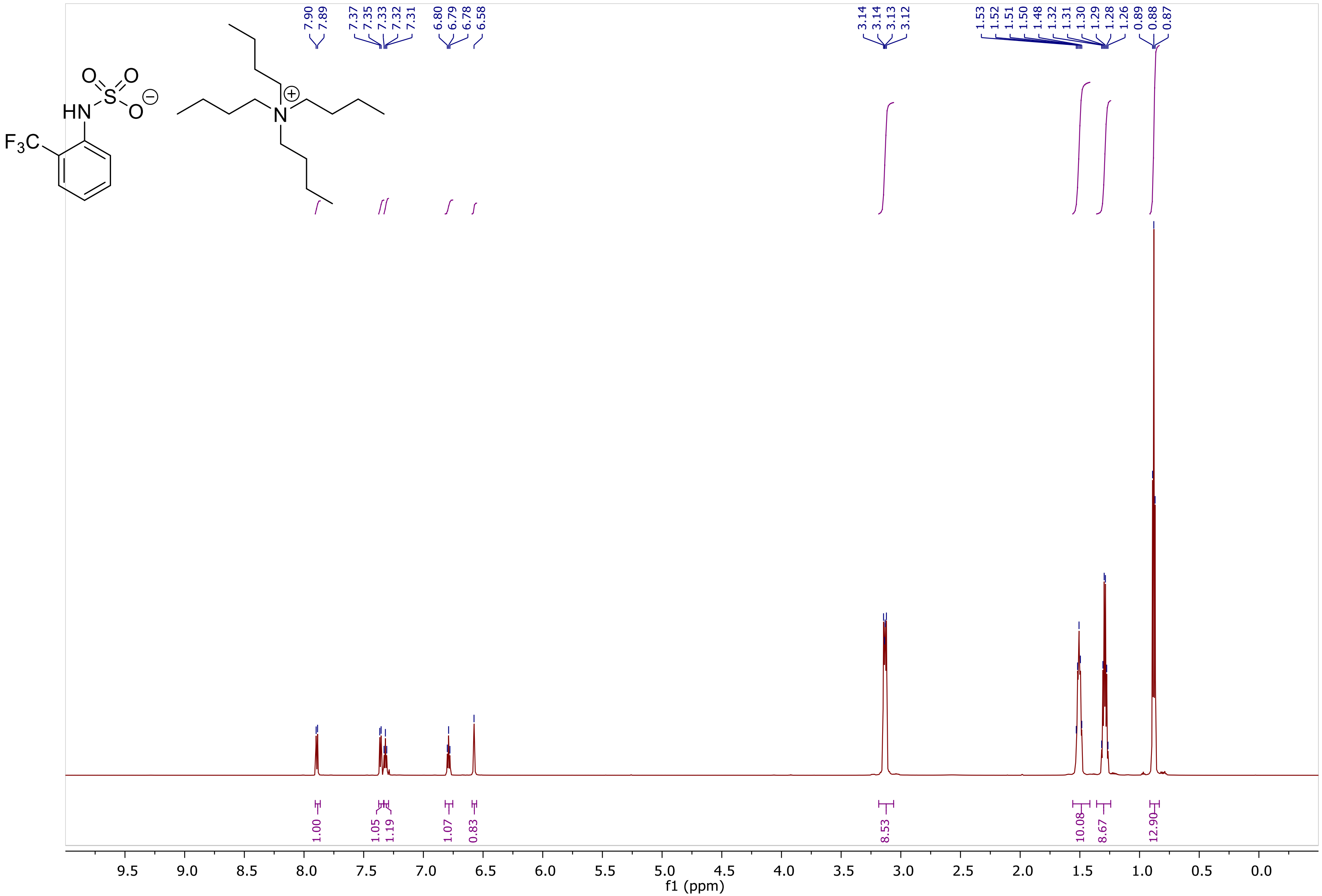

^{13}C NMR of tetrabutylammonium (2-(trifluoromethyl)phenyl)sulfamate in $CDCl_3$

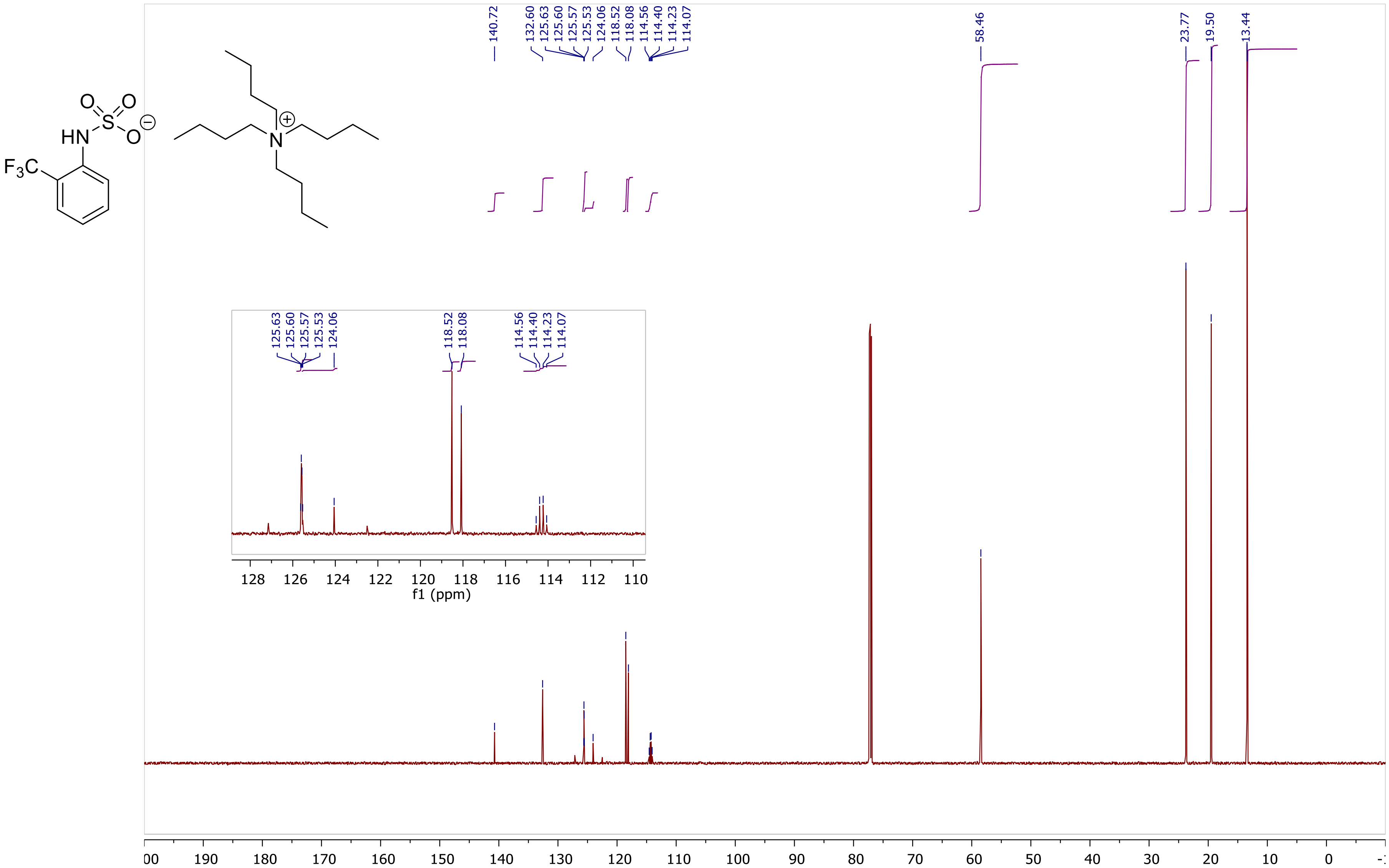

^{19}F NMR of tetrabutylammonium (2-(trifluoromethyl)phenyl)sulfamate in $CDCl_3$

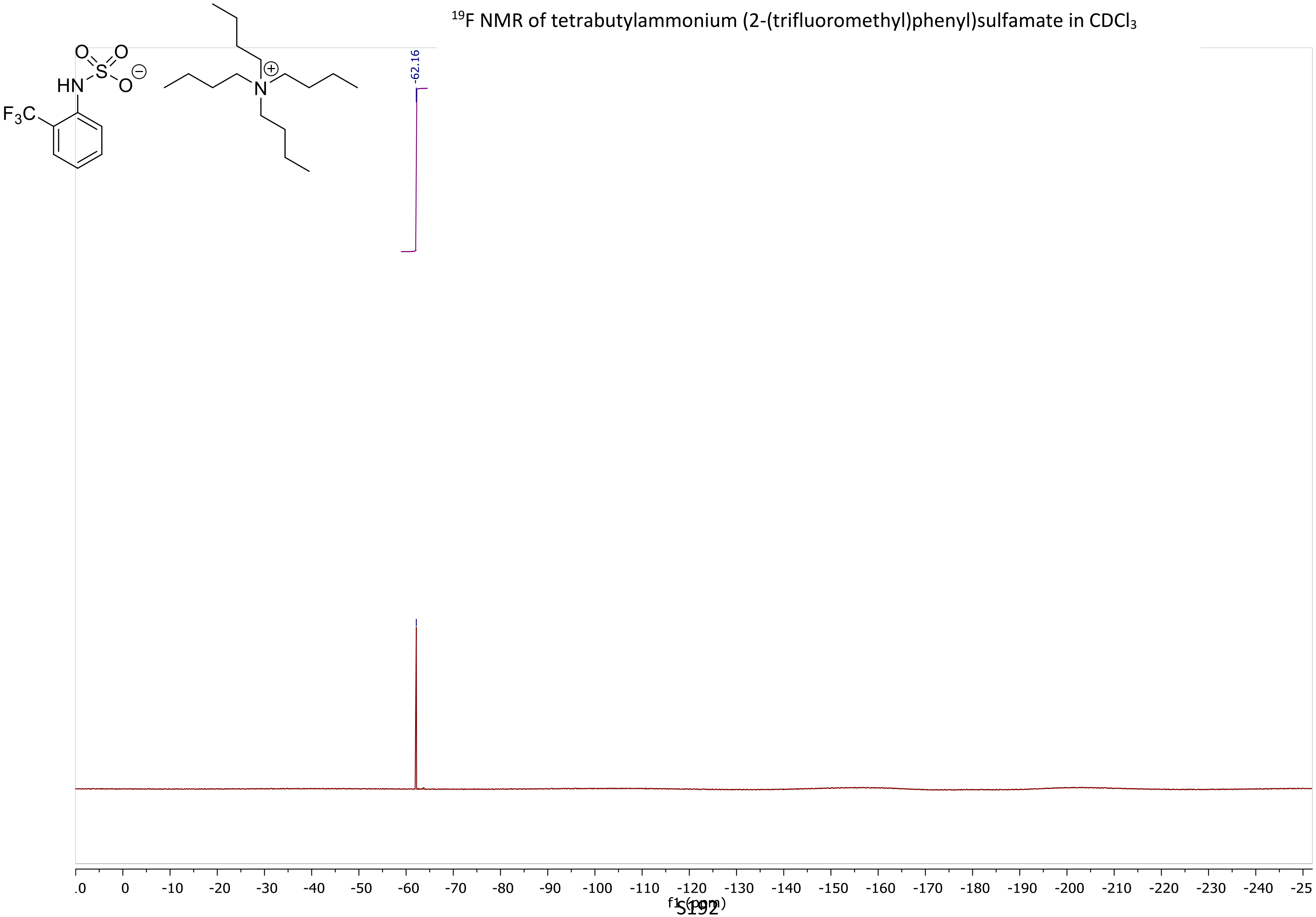

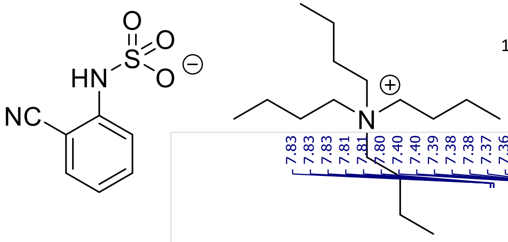

^{1}H NMR of tetrabutylammonium (2-cyanophenyl)sulfamate in $CDCl_3$

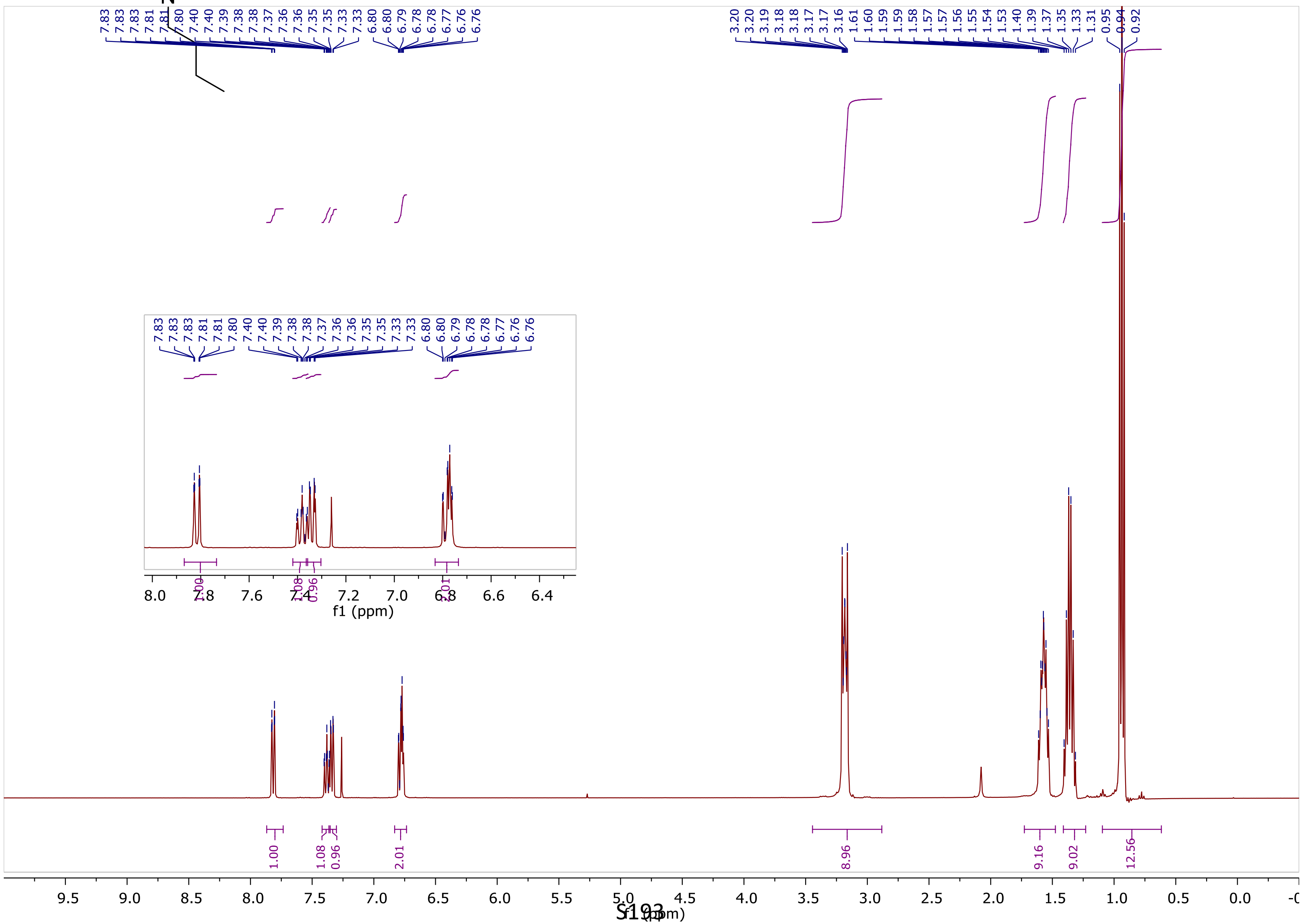

^{13}C NMR of tetrabutylammonium (2-cyanophenyl)sulfamate in $CDCl_3$

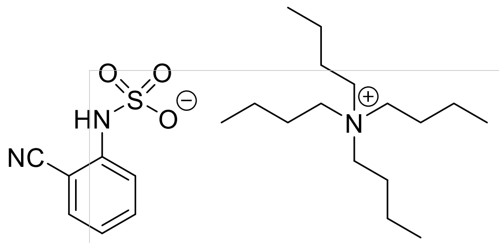

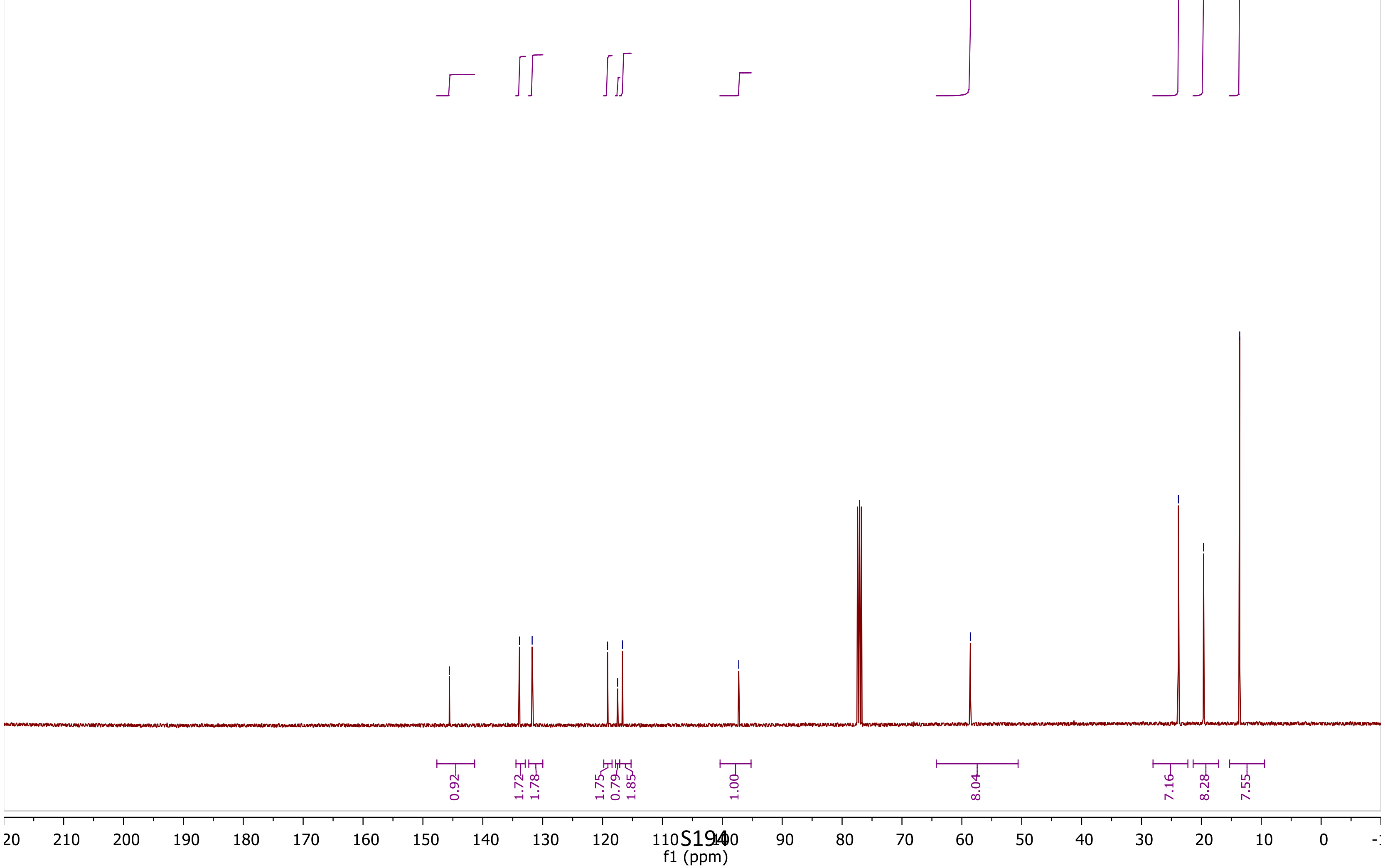

^{1}H NMR of tetrabutylammonium pyridine-2-ylsulfamate in MeOD-d4

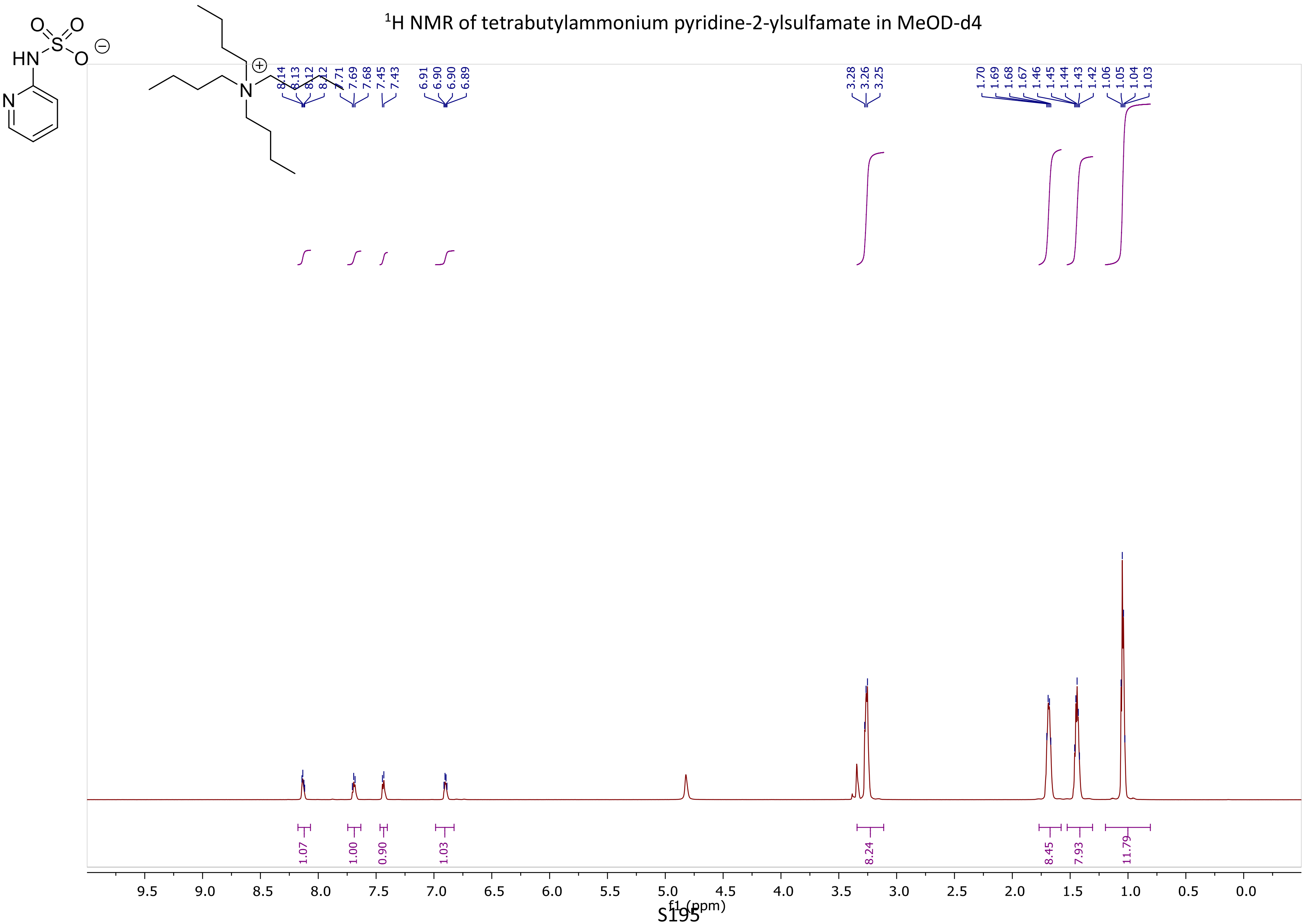

^{13}C NMR of tetrabutylammonium pyridine-2-ylsulfamate in $CDCl_3$

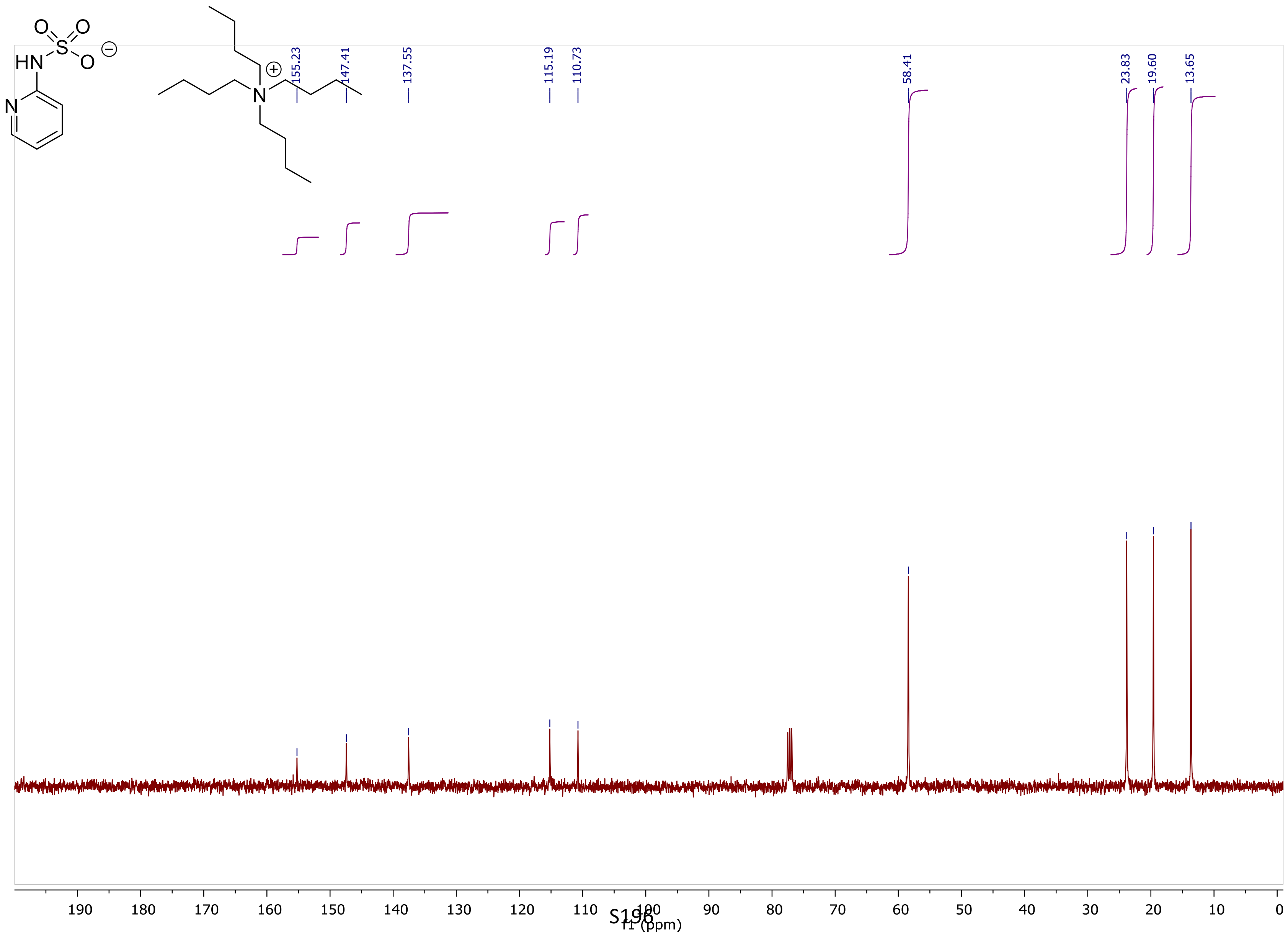

^{1}H NMR of 1-methyl-4-nitro-1H-indole in $CDCl_3$

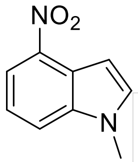

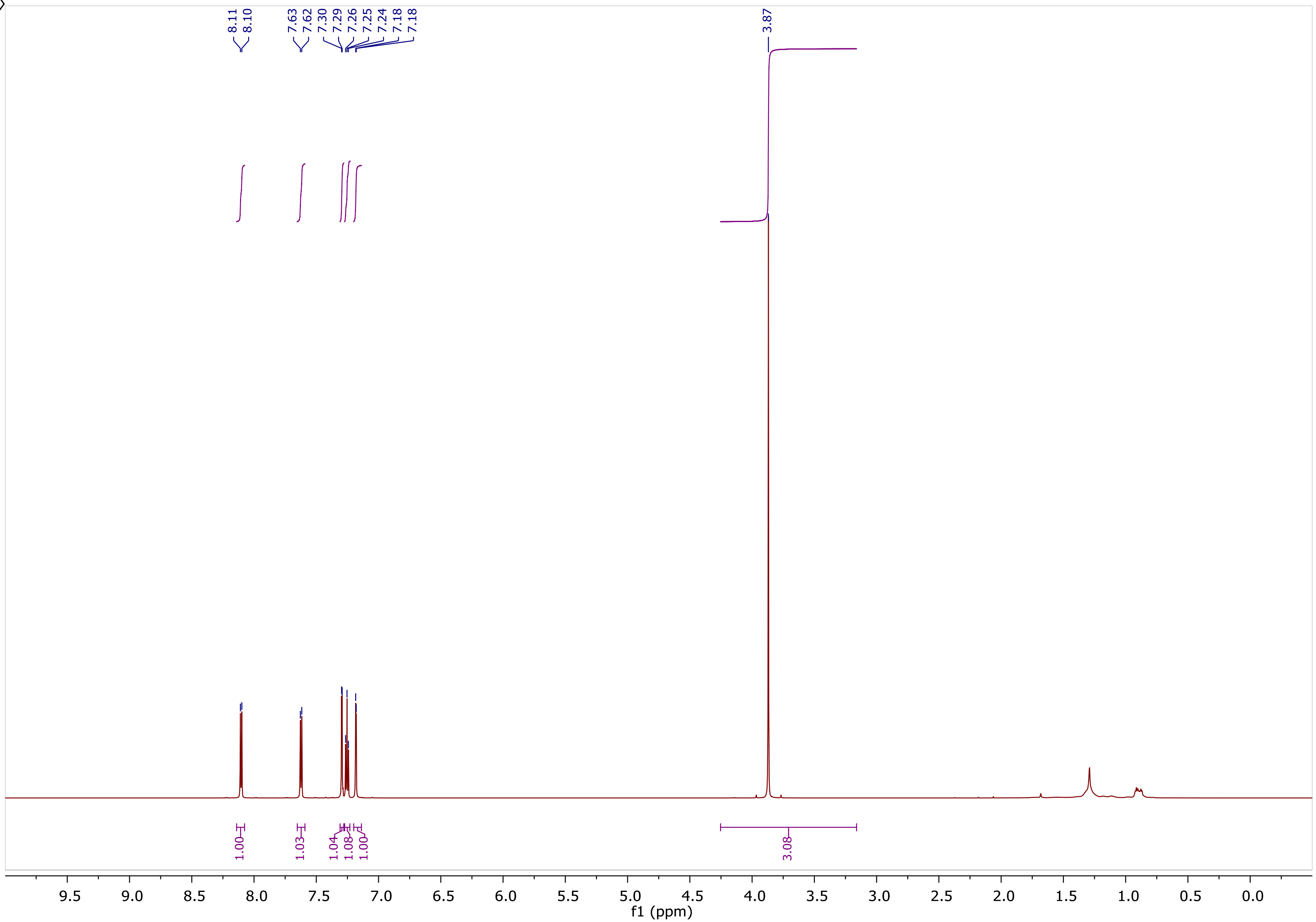

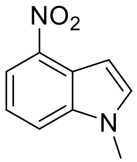

^{13}C NMR of 1-methyl-4-nitro-1H-indole in $CDCl_3$

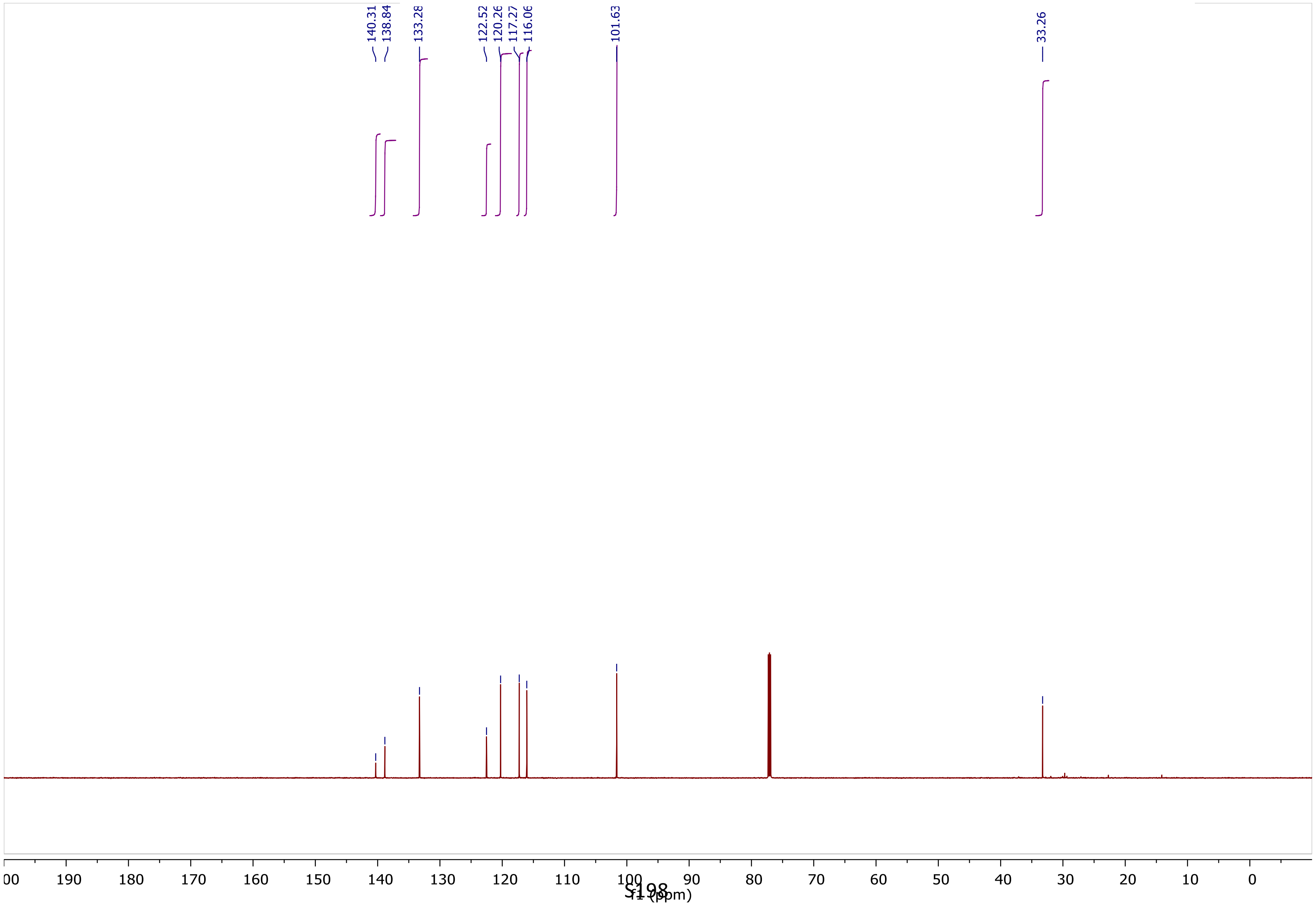

^{1}H NMR of 1-methyl-1H-indol-4-amine in $CDCl_3$

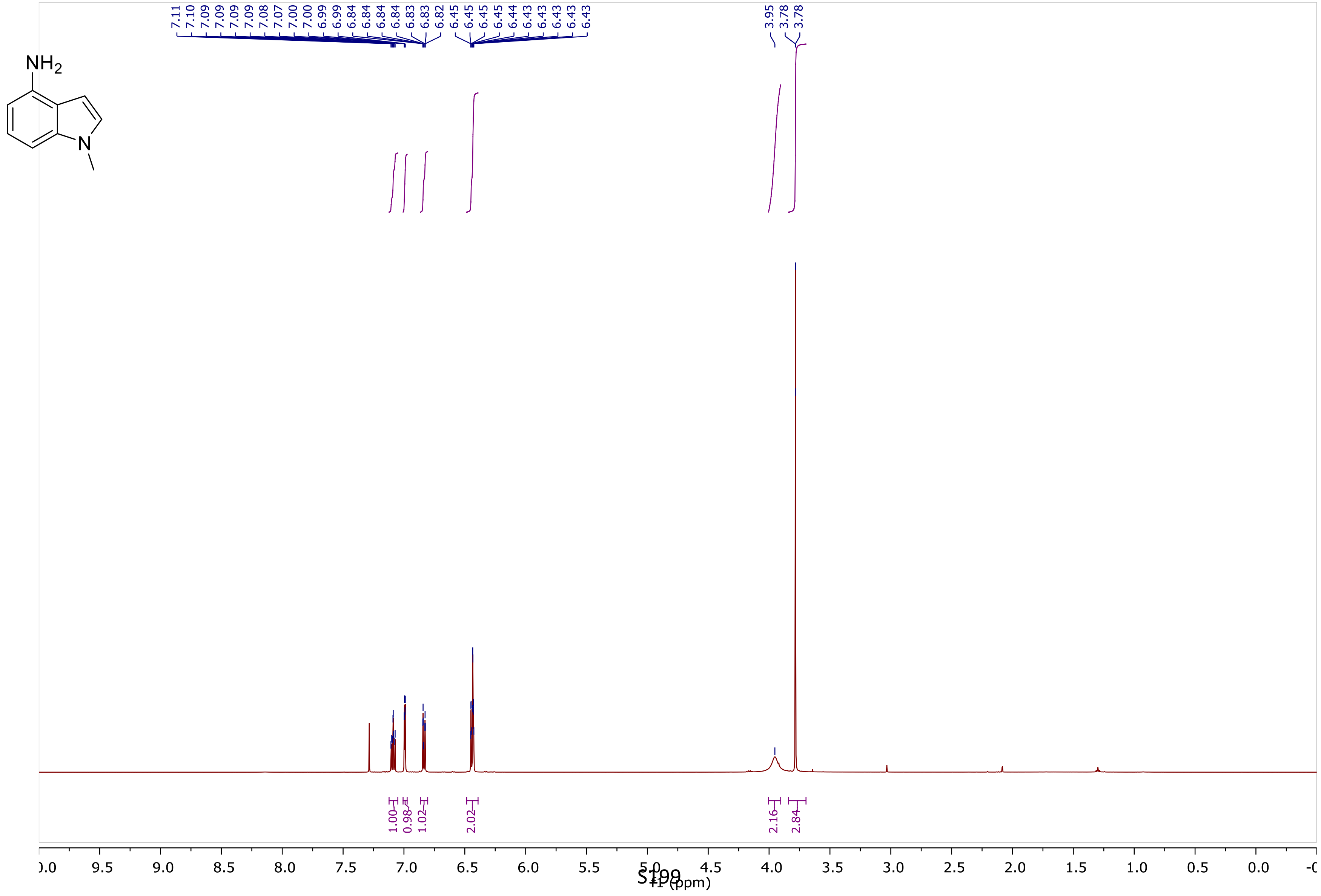

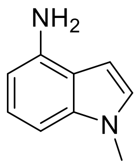

^{13}C NMR of 1-methyl-1H-indol-4-amine in $CDCl_3$

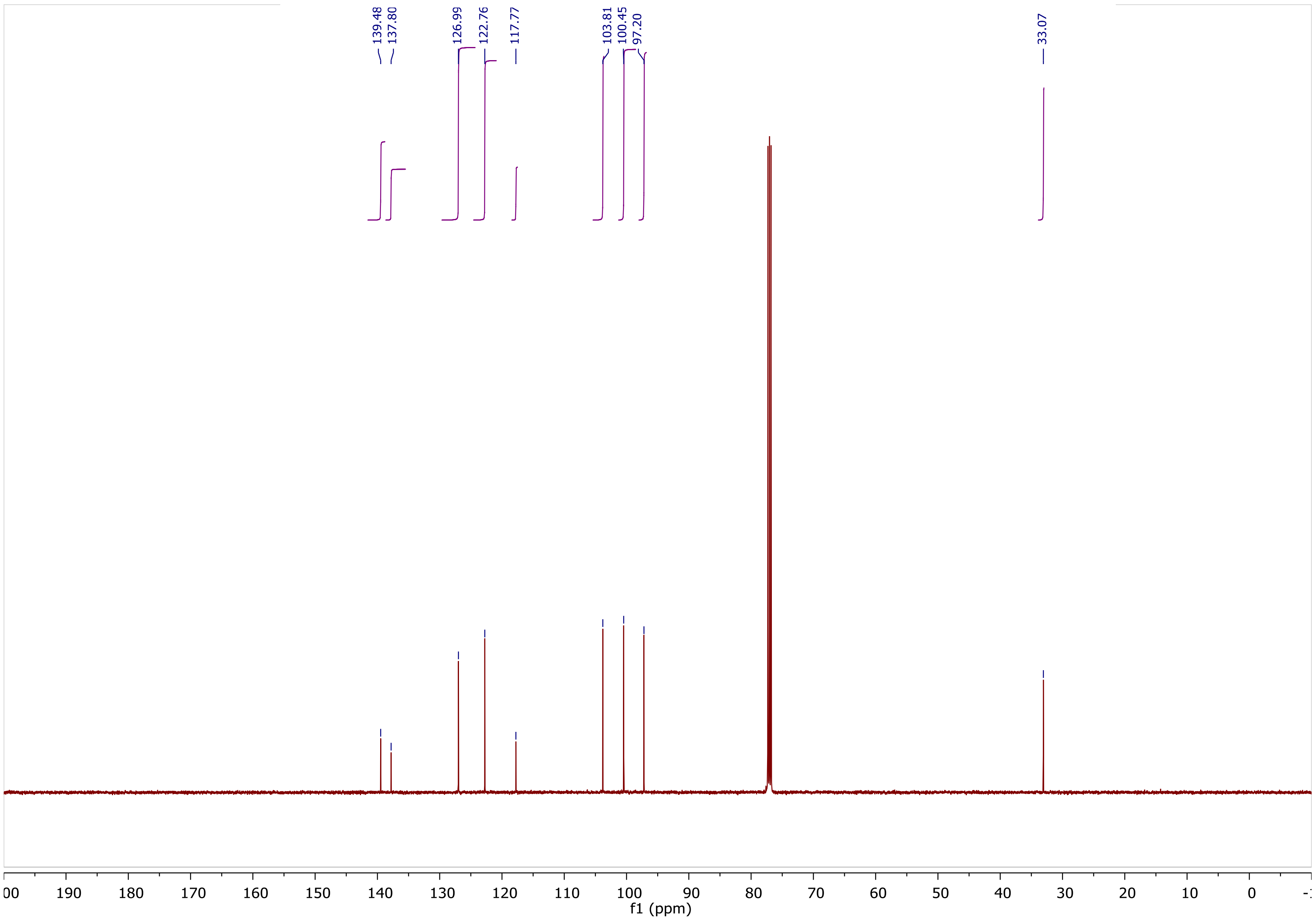

^{1}H NMR of tetrabutylammonium (1-methyl-1H-indol-4-yl)sulfamate in MeOD-d4

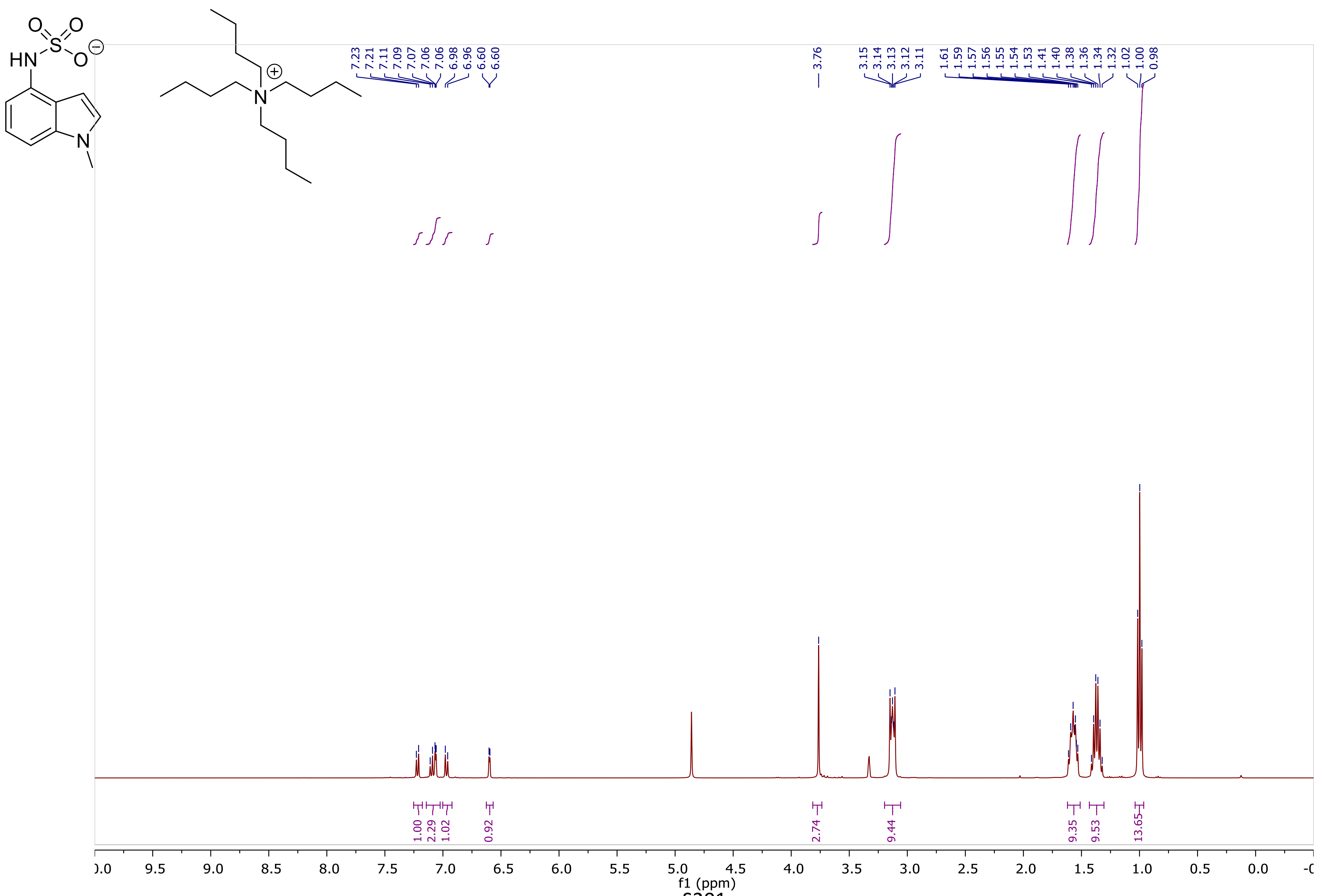

^{13}C NMR of tetrabutylammonium (1-methyl-1H-indol-4-yl)sulfamate in MeOD-d4

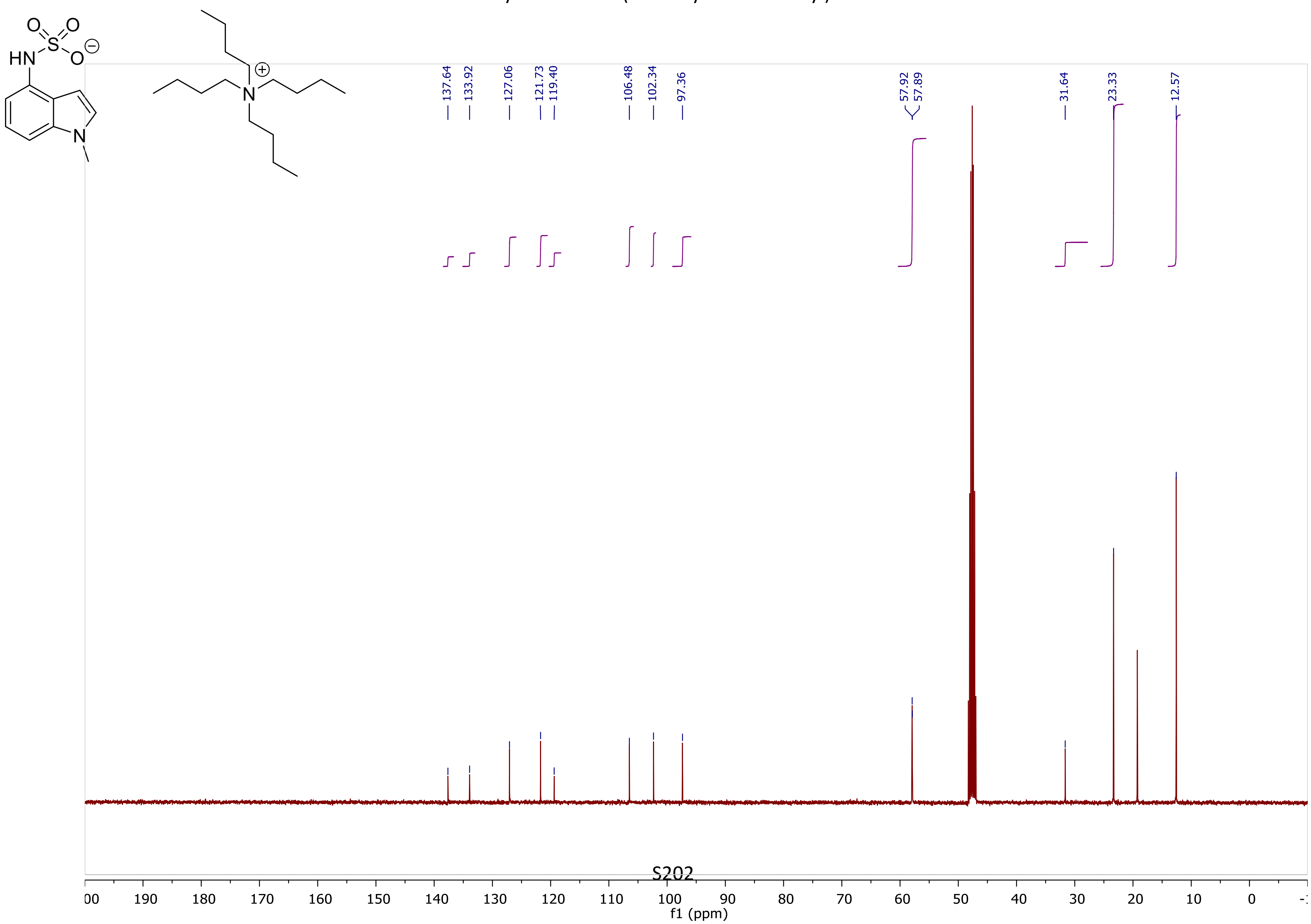

Aminating Agents

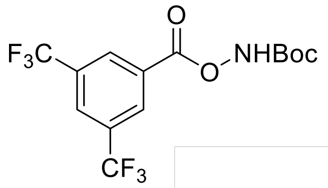

^{1}H NMR of *tert*-butyl ((3,5-bis(trifluoromethyl)benzoyl)oxy)carbamate in $CDCl_3$

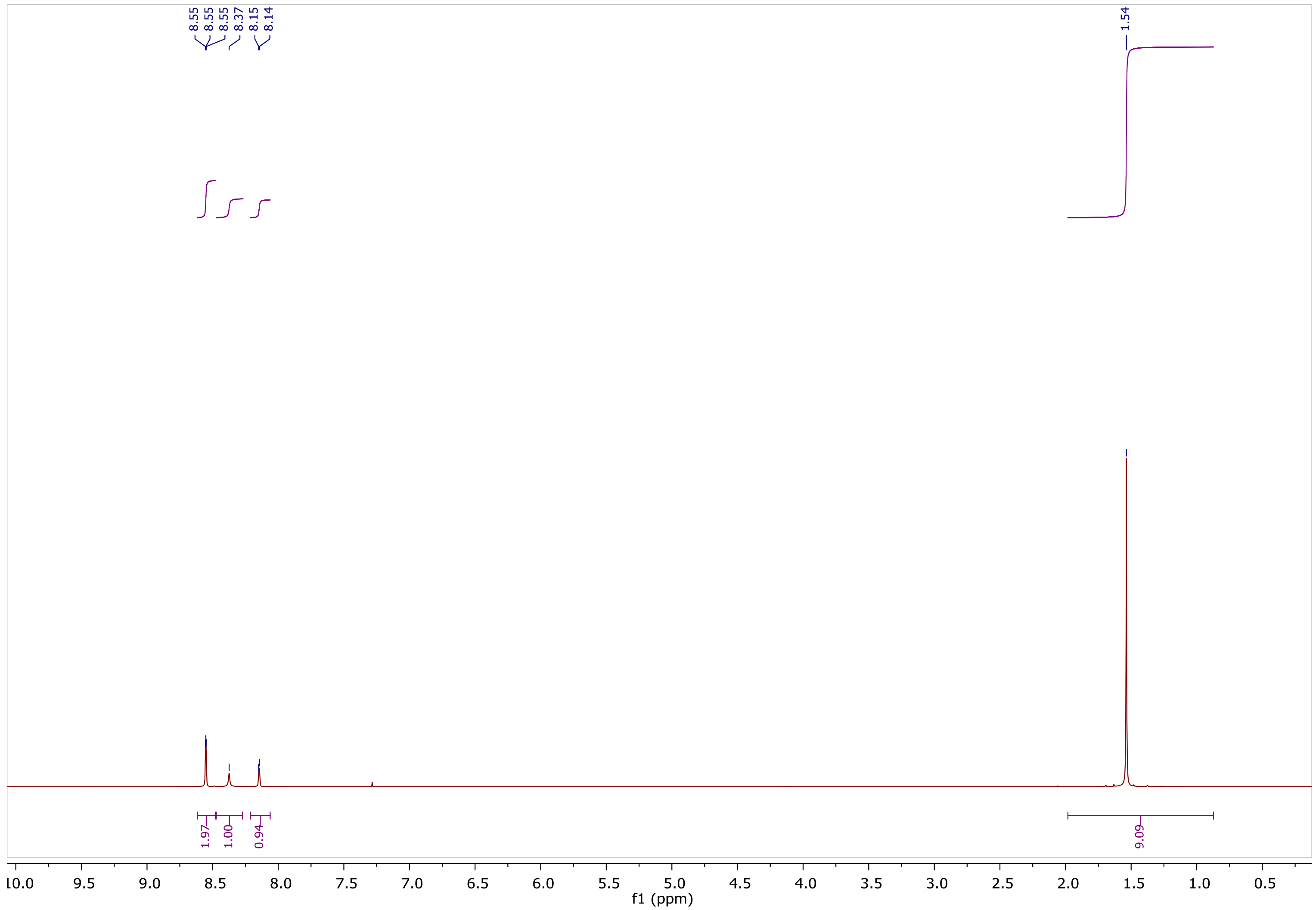

^{13}C NMR of *tert*-butyl ((3,5-bis(trifluoromethyl)benzoyl)oxy)carbamate in $CDCl_3$

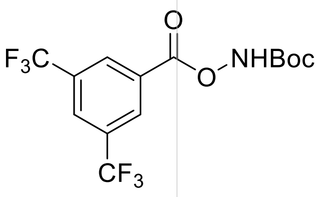

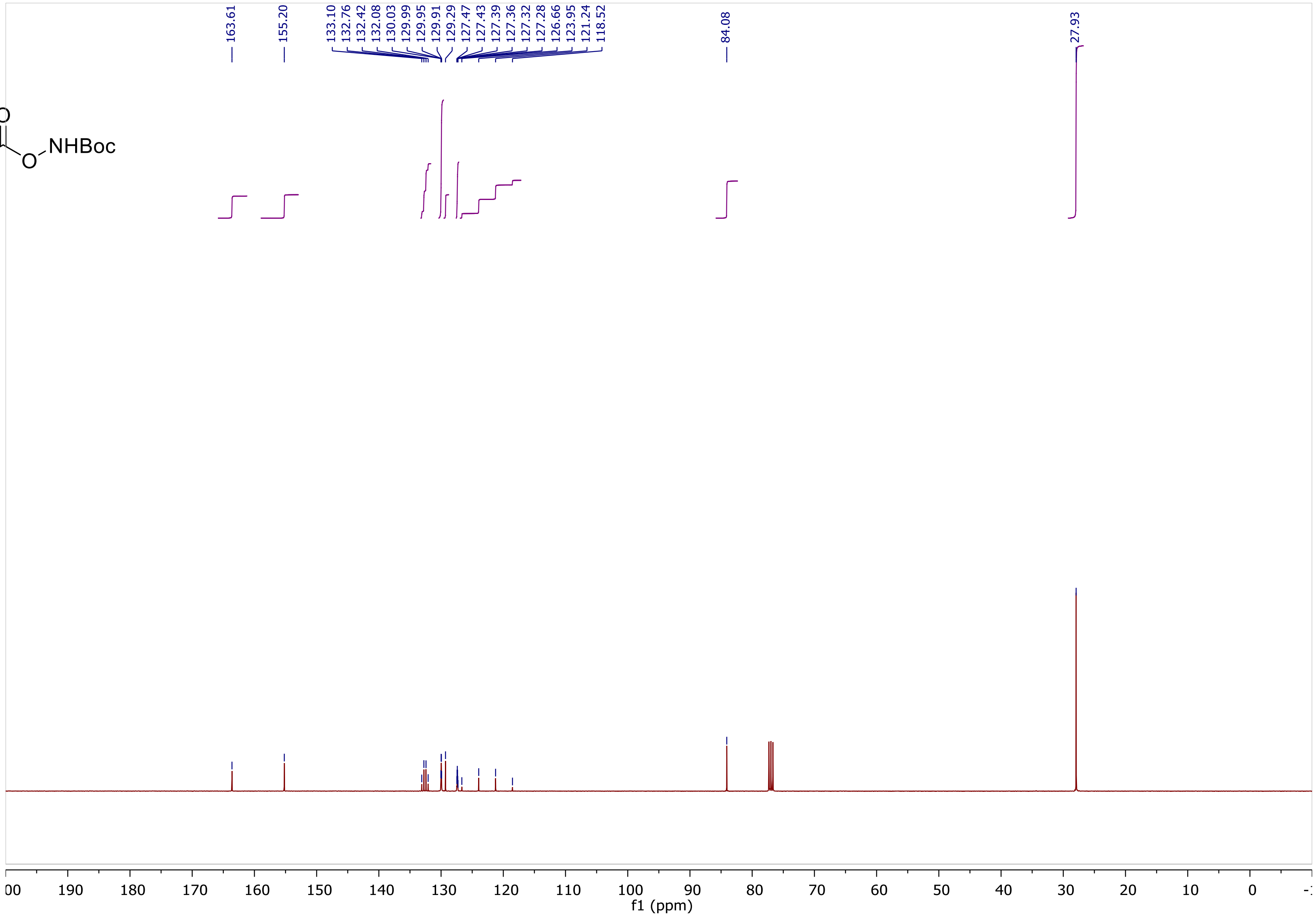

^{19}F NMR of *tert*-butyl ((3,5-bis(trifluoromethyl)benzoyl)oxy)carbamate in $CDCl_3$

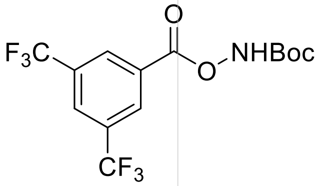

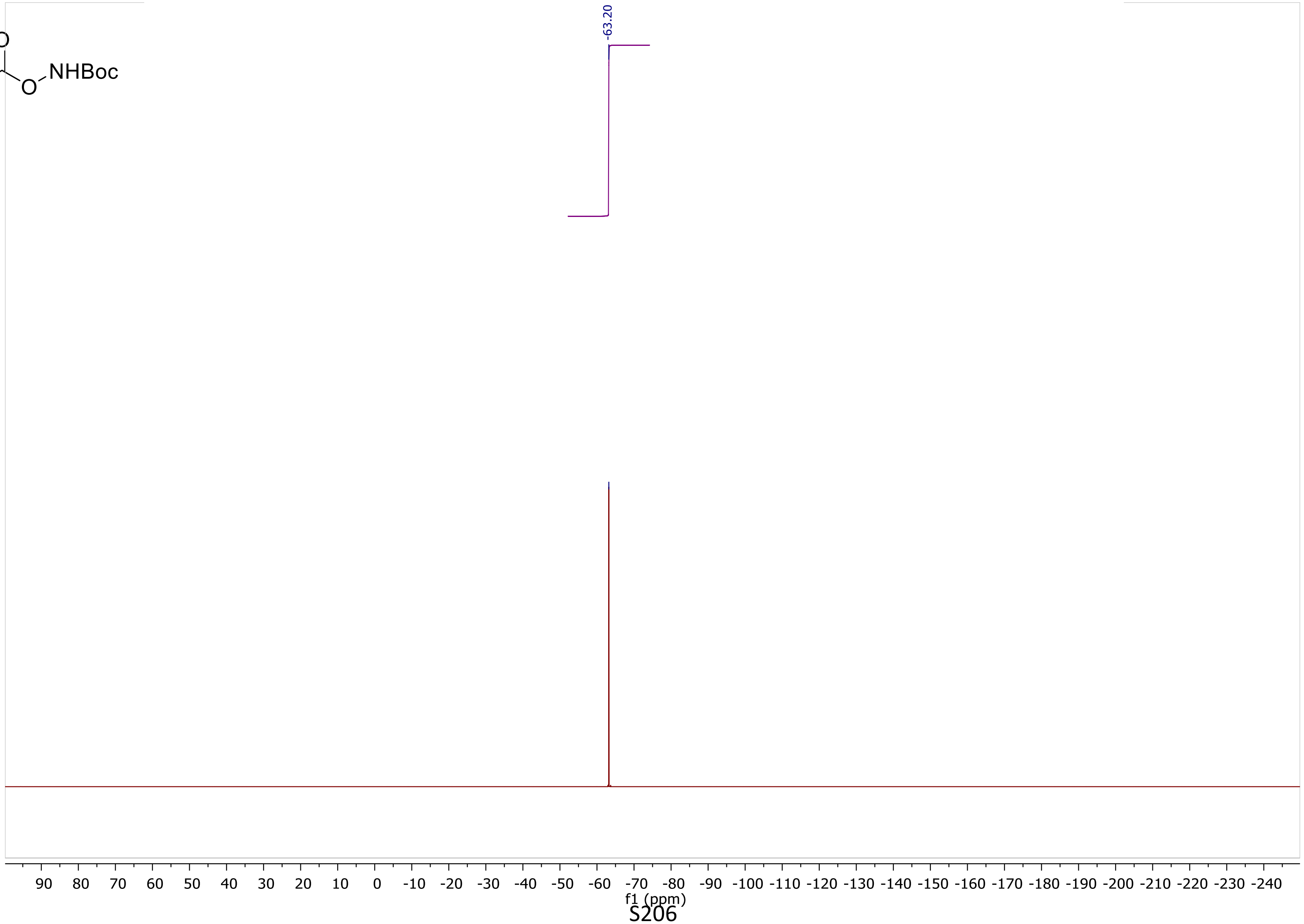

^{1}H NMR of *O*-(3,5-bis(trifluoromethyl)benzoyl)hydroxylammonium trifluoromethanesulfonate **2a** in CD_3CN

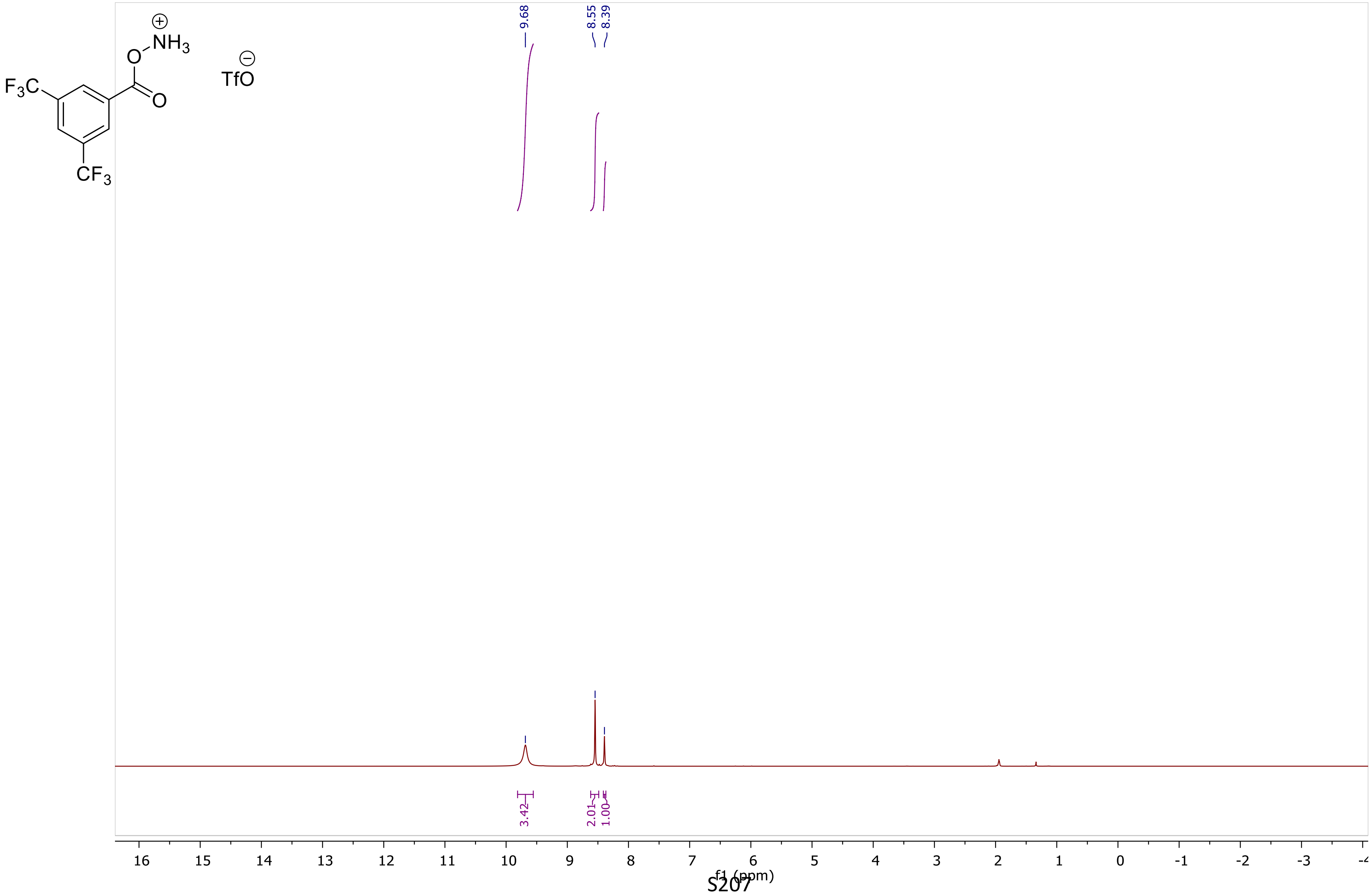

^{13}C NMR of *O*-(3,5-bis(trifluoromethyl)benzoyl)hydroxylammonium trifluoromethanesulfonate **2a** in CD_3CN

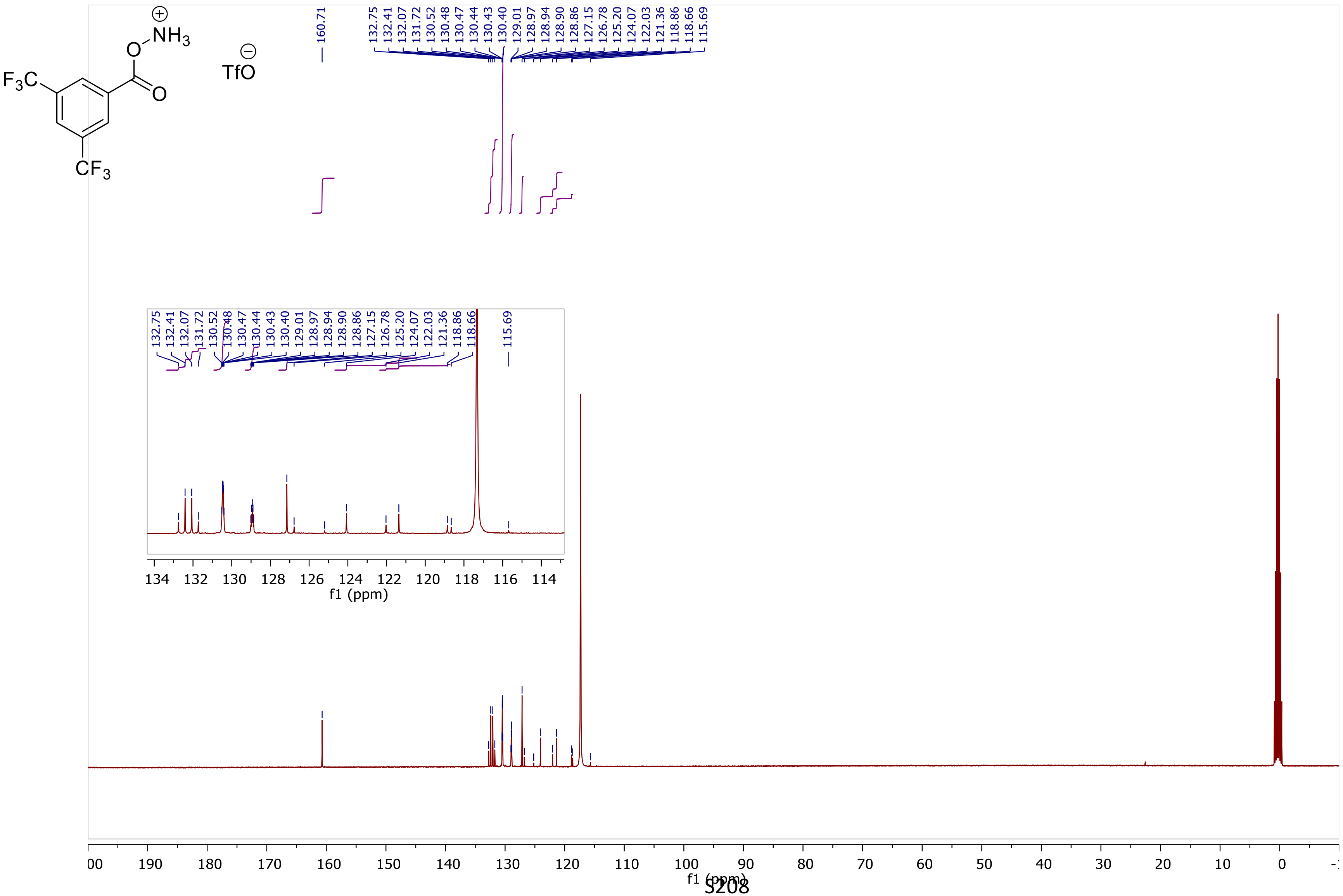

^{19}F NMR of *O*-(3,5-bis(trifluoromethyl)benzoyl)hydroxylammonium trifluoromethanesulfonate **2a** in MeOD-d[4]

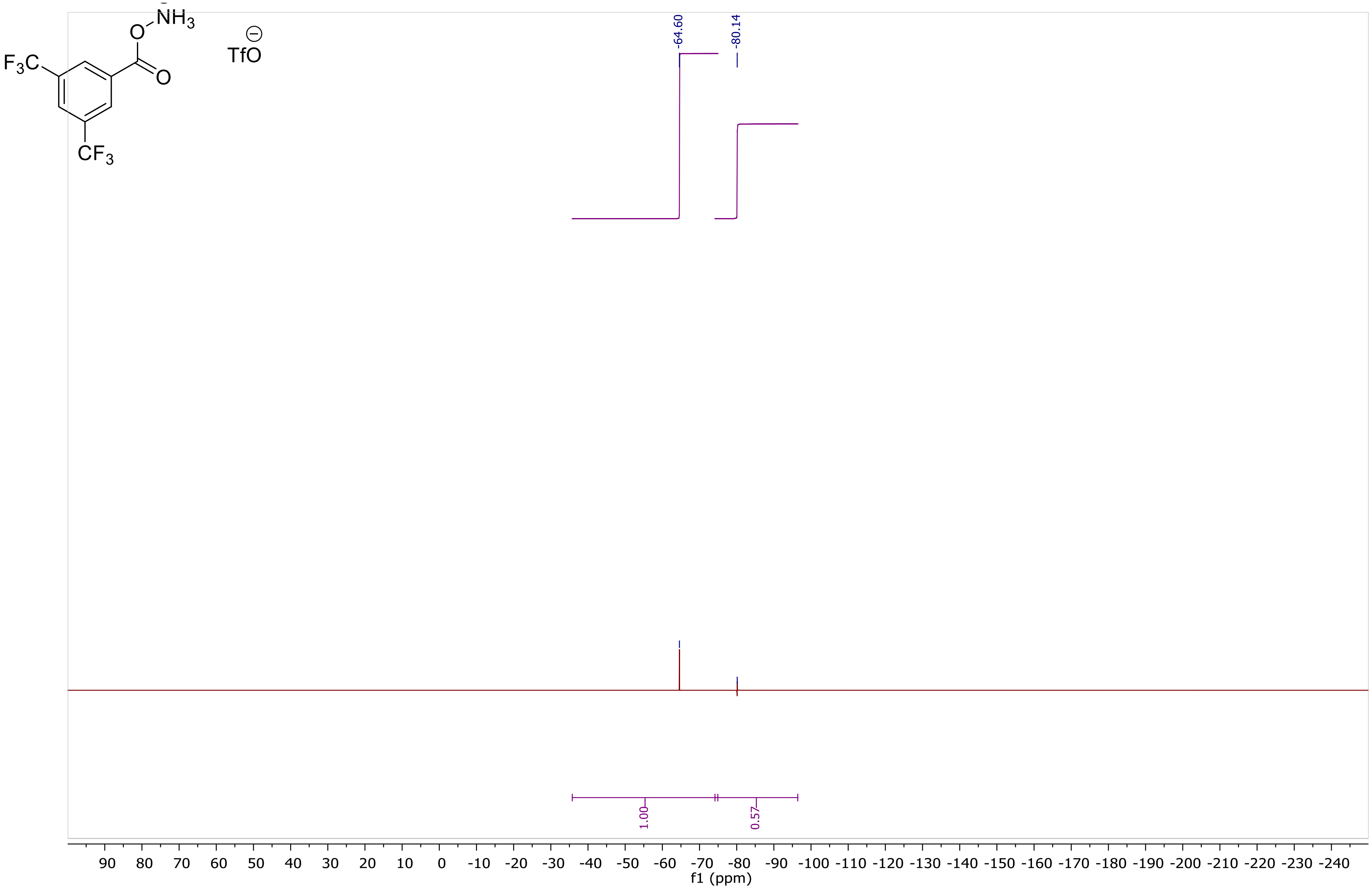

^{1}H NMR of *O*-benzoylhydroxylammonium trifluoromethanesulfonate **2b** in MeOD-d^4

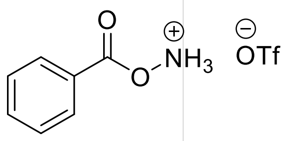

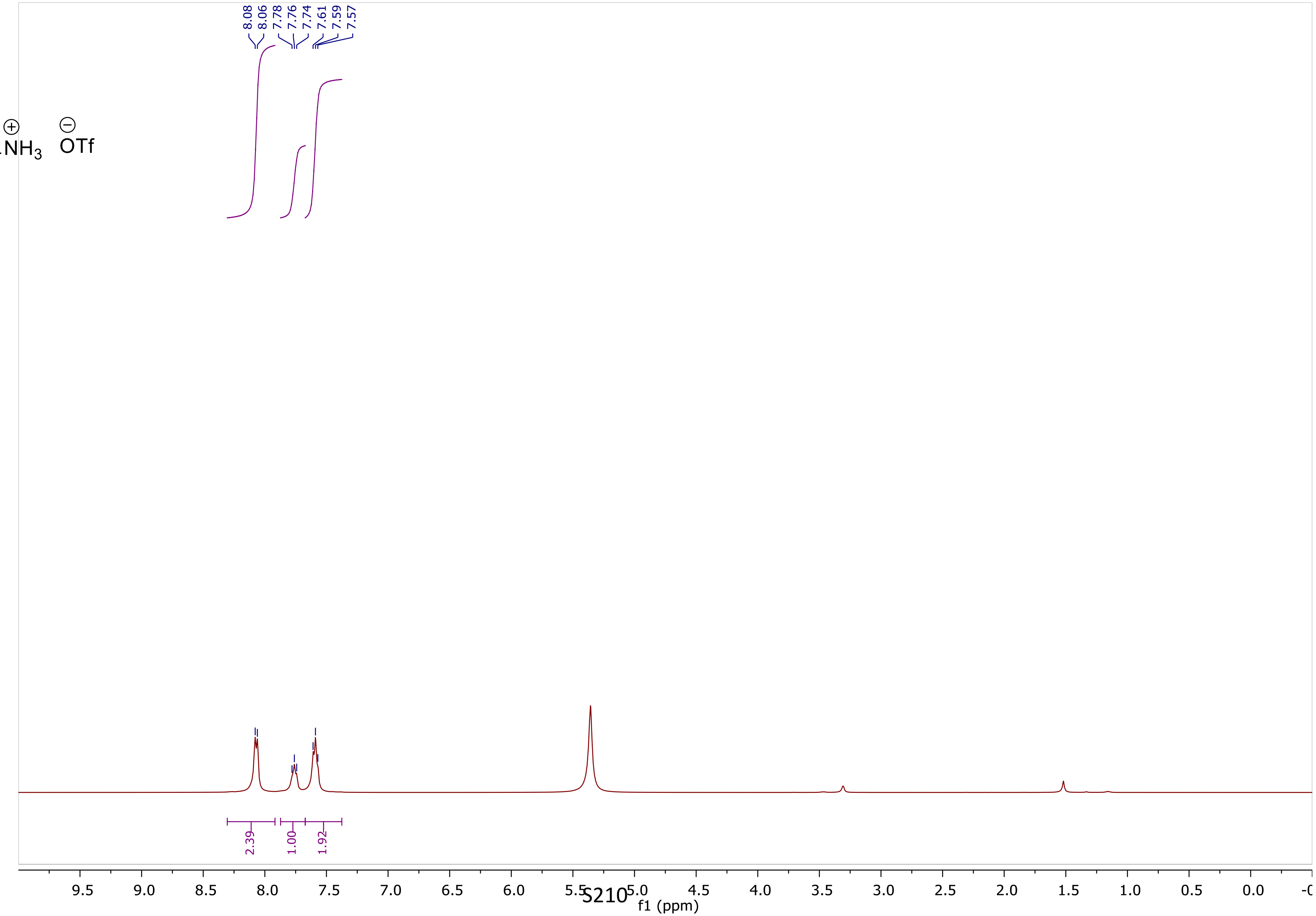

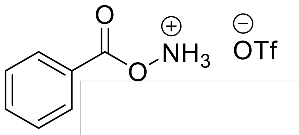

^{13}C NMR of *O*-benzoylhydroxylammonium trifluoromethanesulfonate **2b** in MeOD-*d*[4]

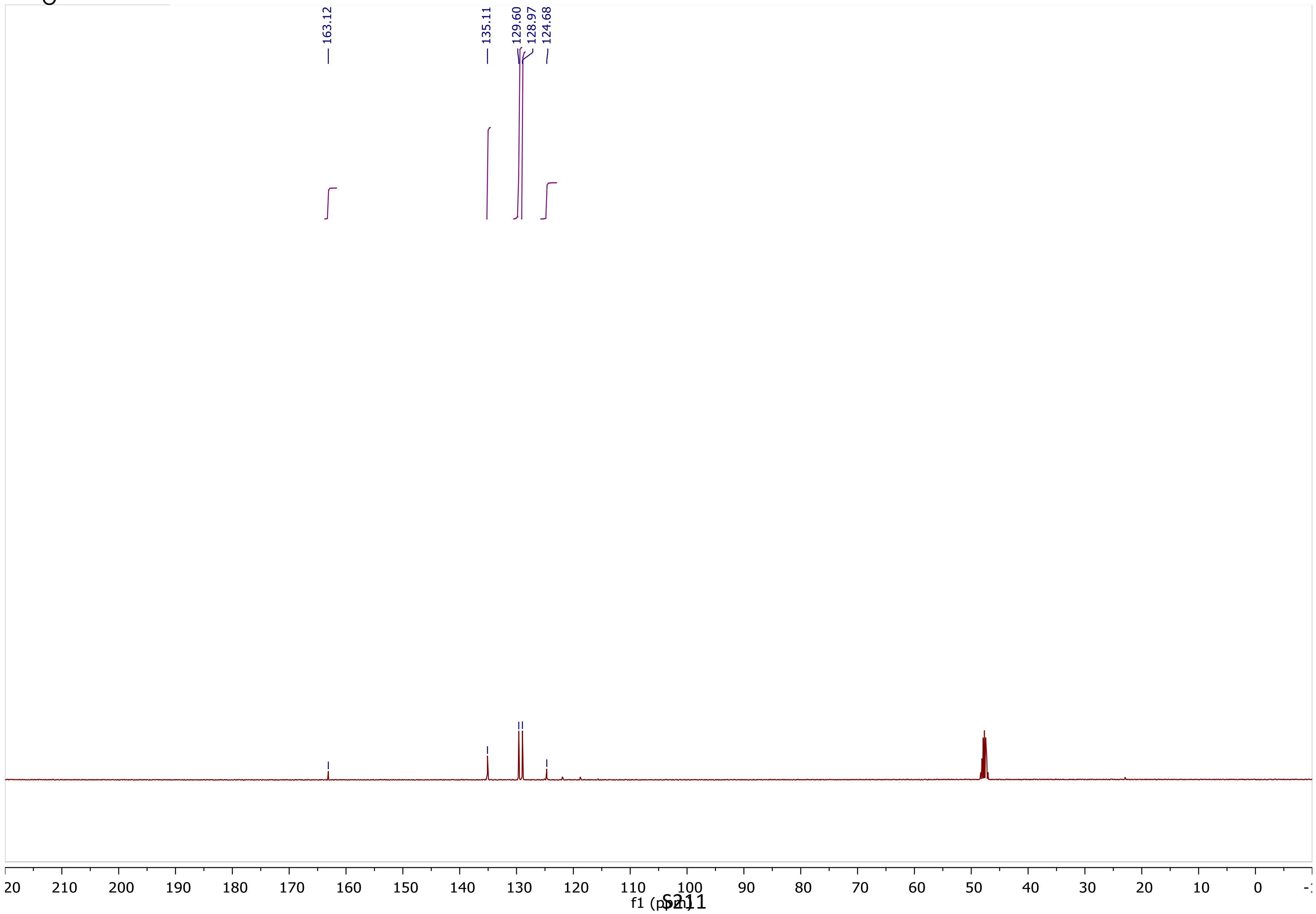

^{19}F NMR of *O*-benzoylhydroxylammonium trifluoromethanesulfonate **2b** in MeOD-*d*^{4}

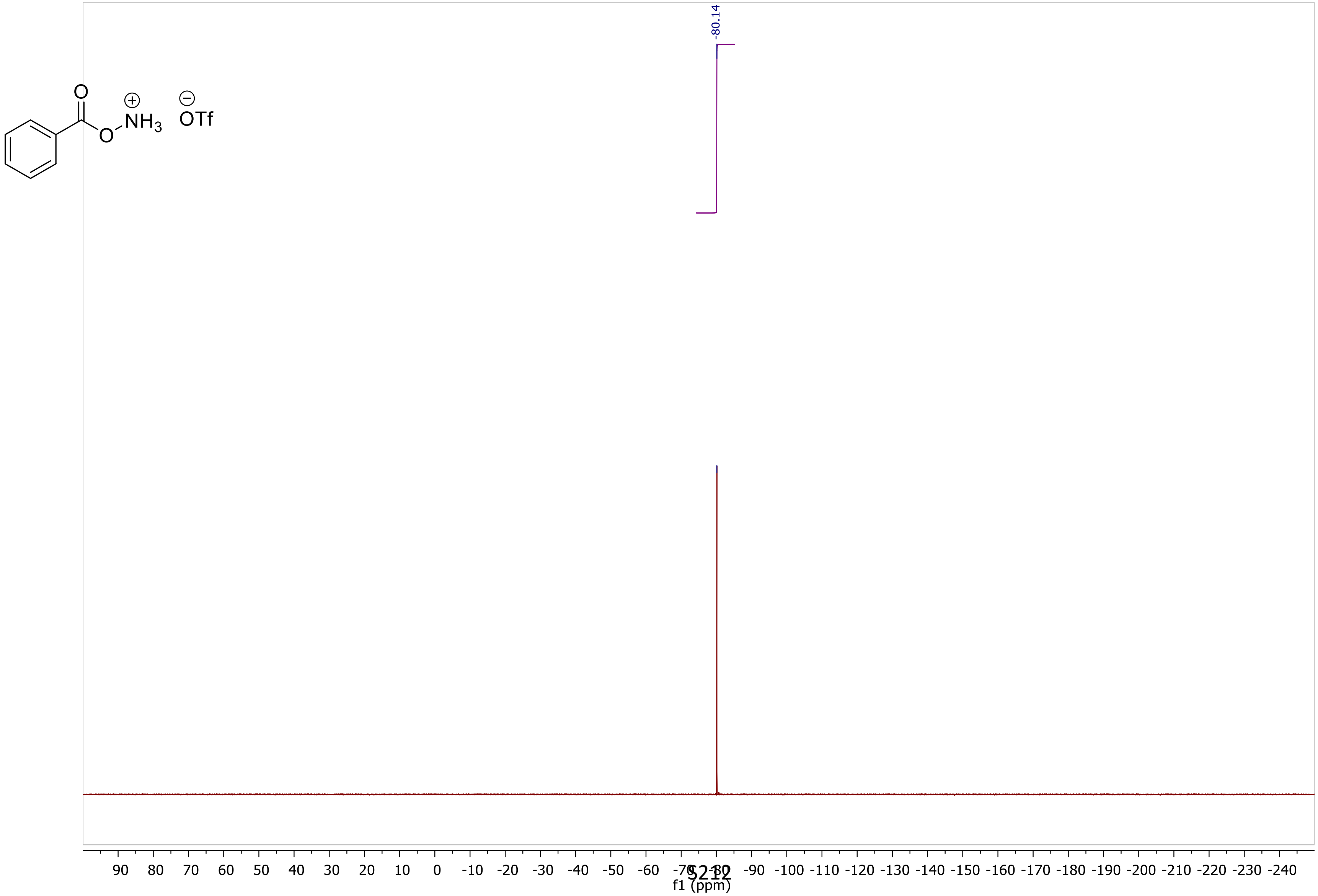

^{1}H NMR of *tert*-butyl (4-nitrobenzoyl)oxy)carbamate in $CDCl_3$

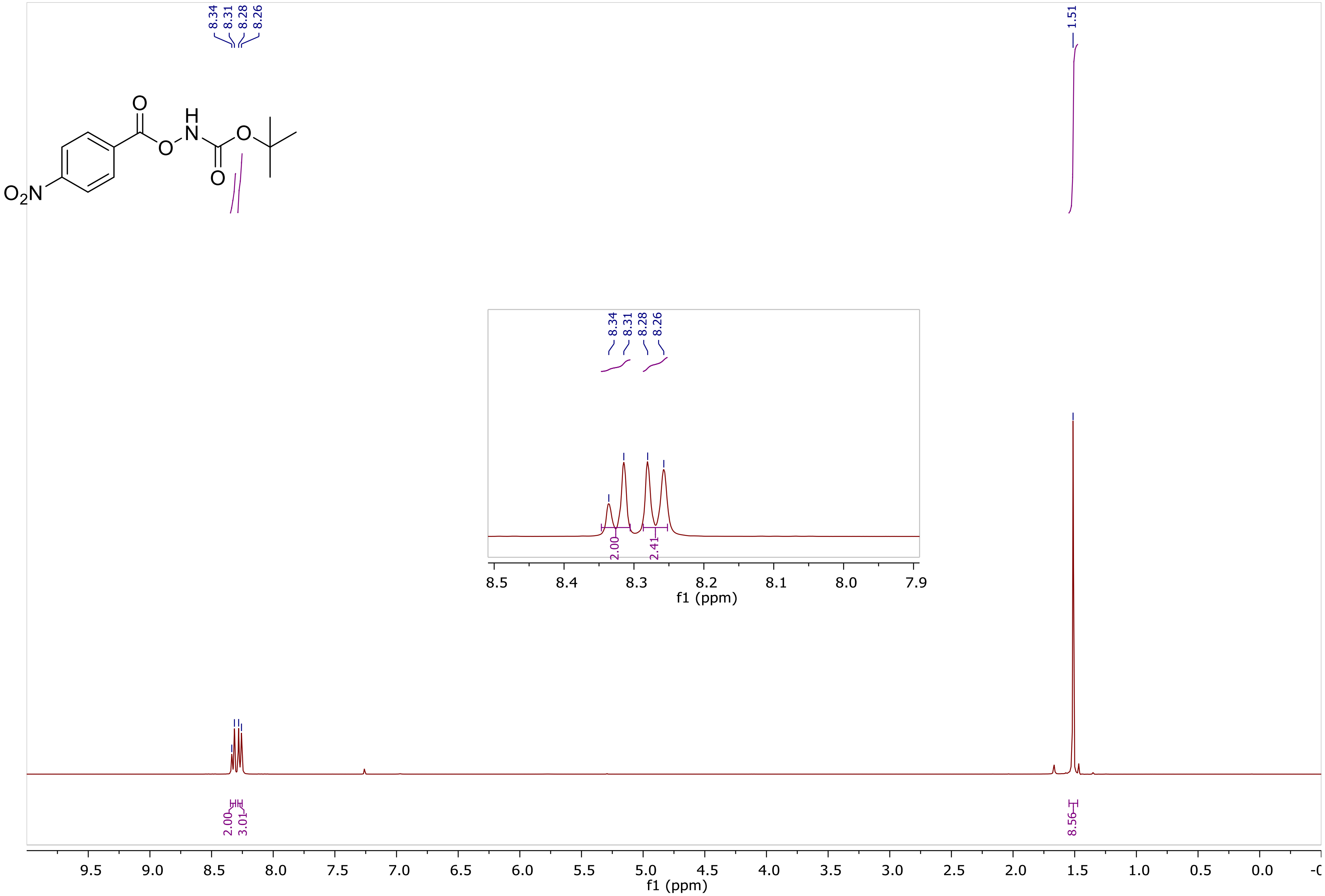

^{13}C NMR of *tert*-butyl (4-nitrobenzoyl)oxy)carbamate in $CDCl_3$

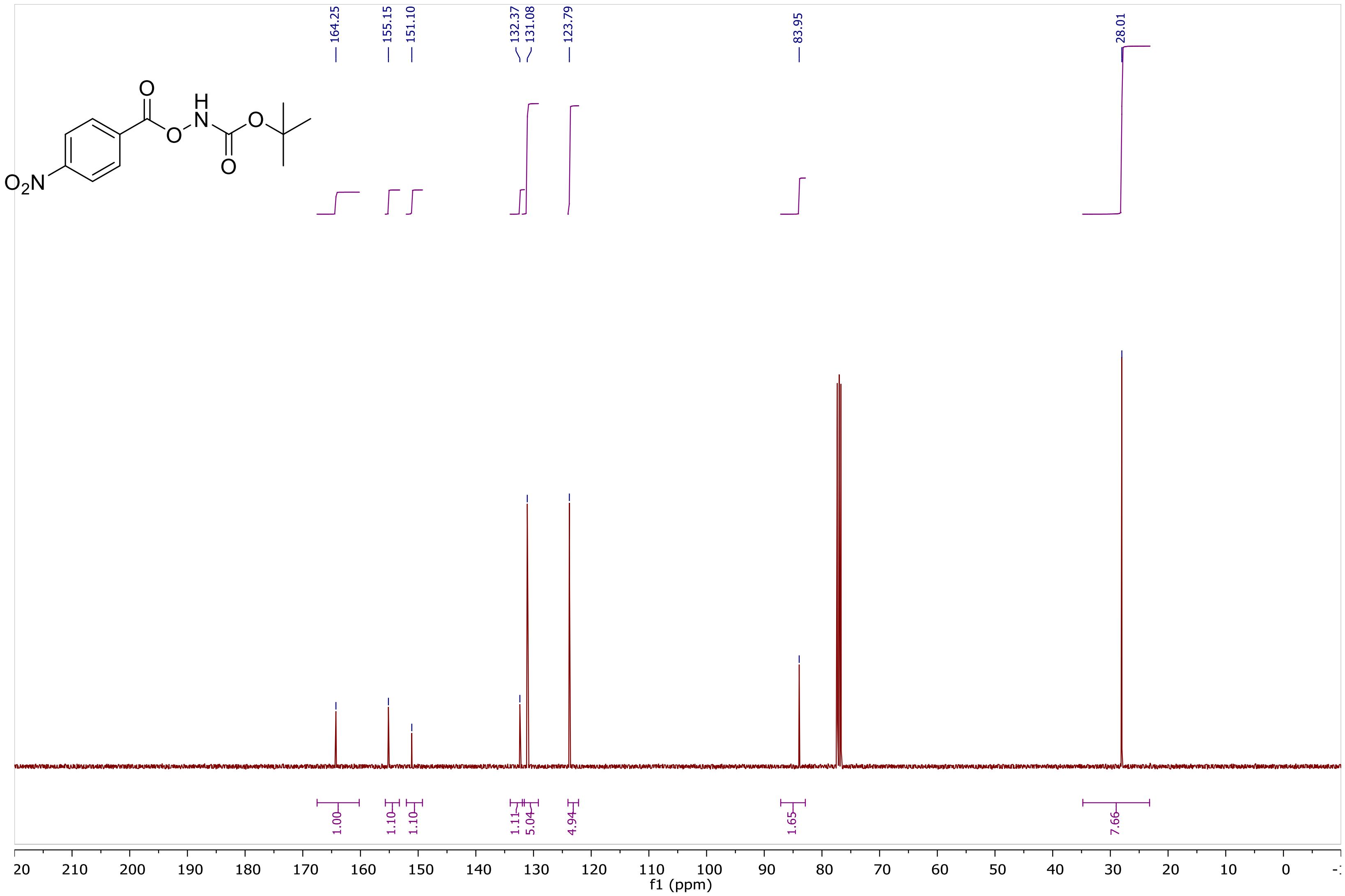

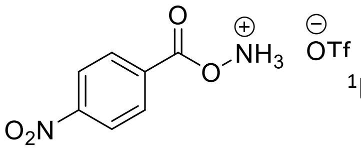

^{1}H NMR of *O*-(4-nitrobenzoyl)hydroxylammonium trifluoromethanesulfonate **2c** in CD_3CN

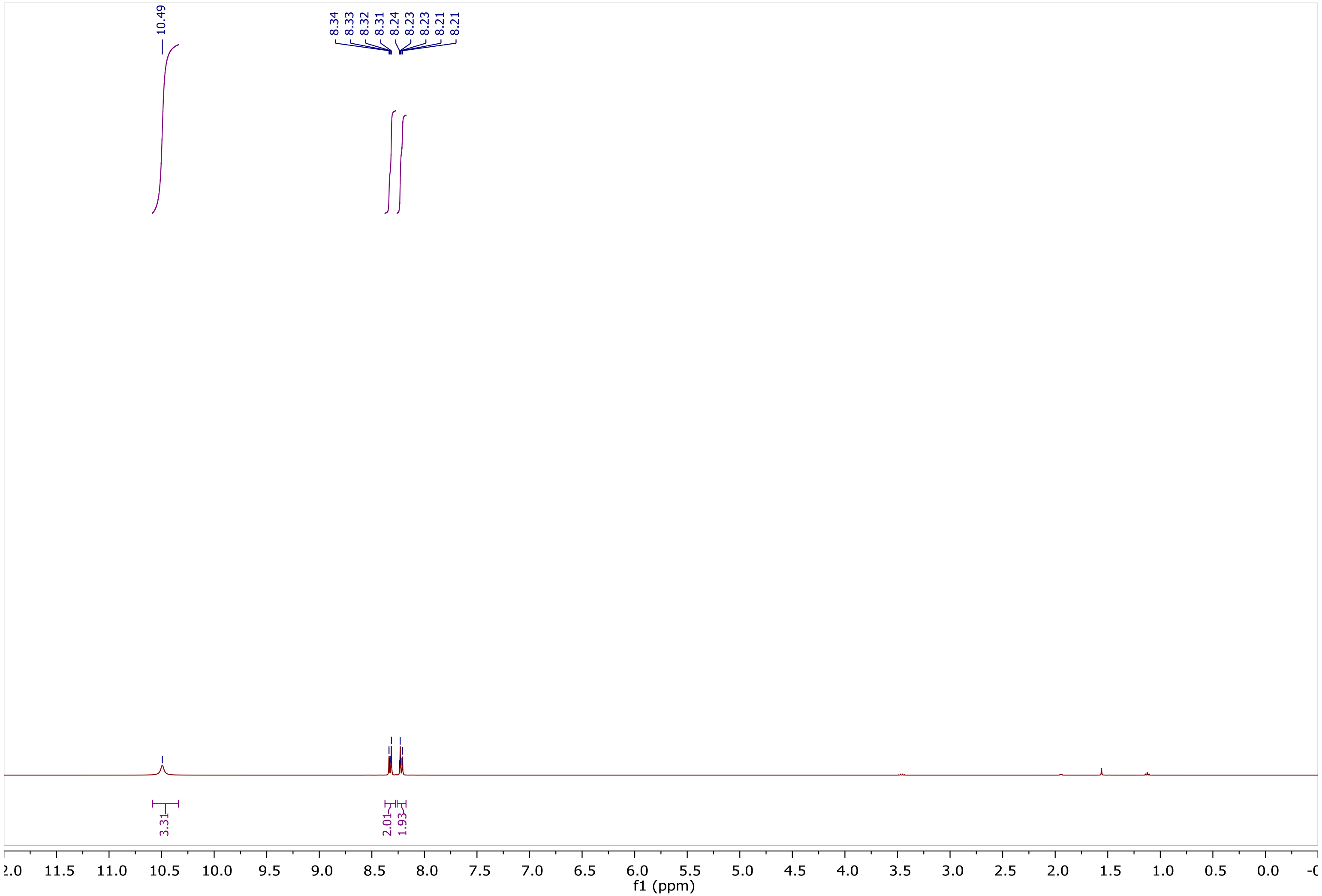

^{13}C NMR of *O*-(4-nitrobenzoyl)hydroxylammonium trifluoromethanesulfonate **2c** in CD_3CN

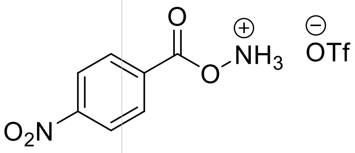

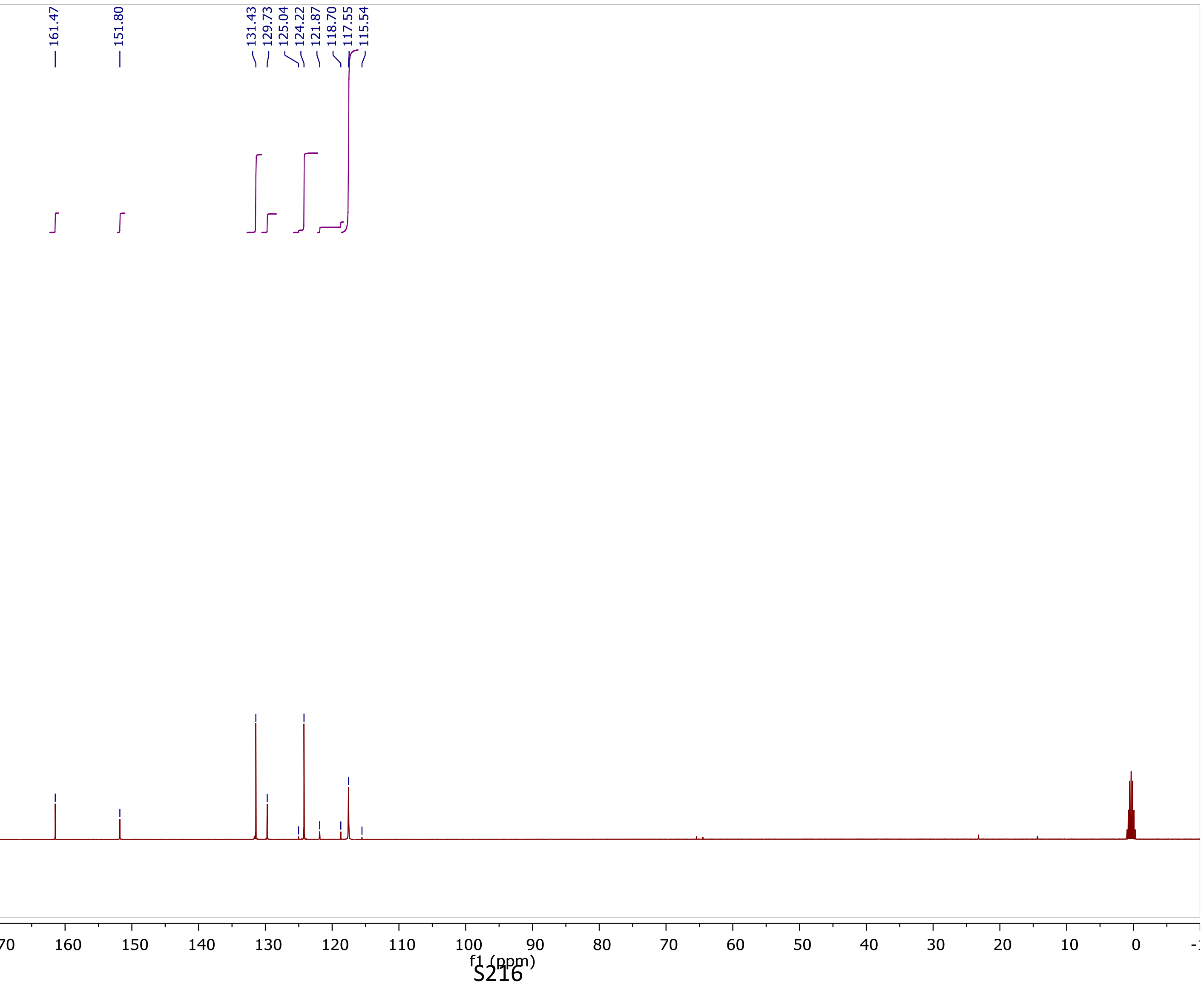

^{19}F NMR of *O*-(4-nitrobenzoyl)hydroxylammonium trifluoromethanesulfonate **2c** in DMSO-d^{6}

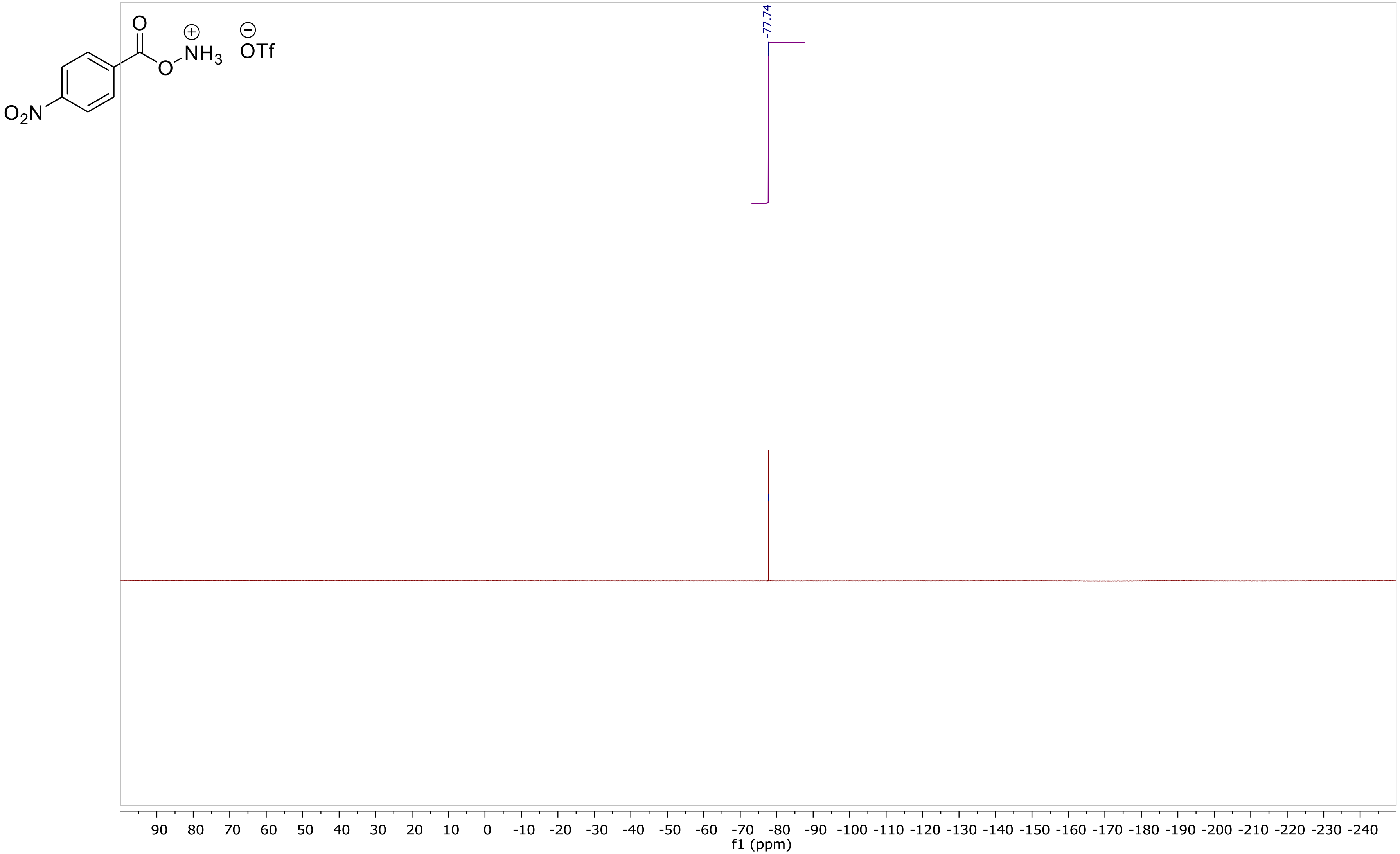

^{1}H NMR of *O*-(4-methoxybenzoyl)hydroxylammonium trifluoromethanesulfonate **2d** in MeOD-d[4]

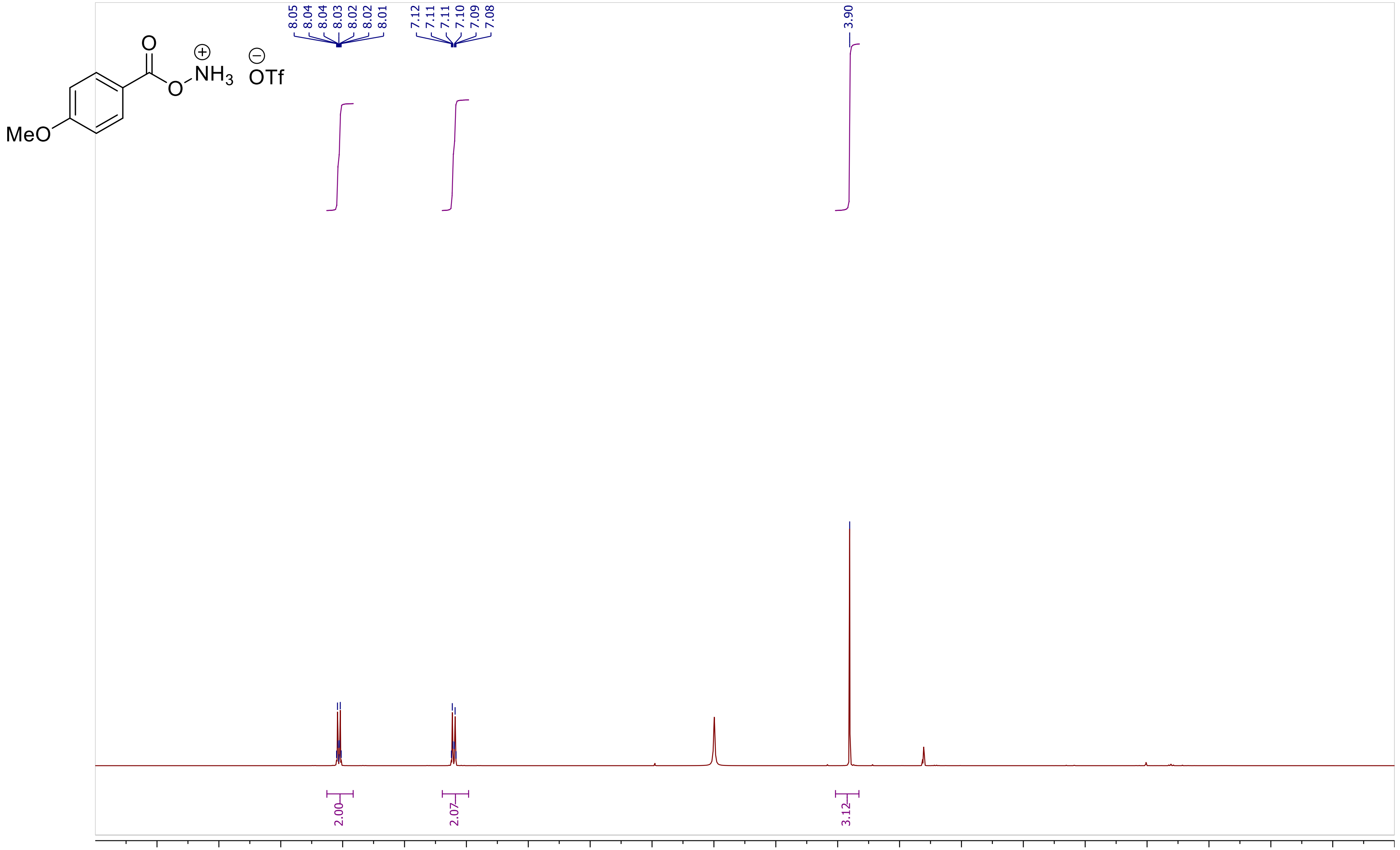

^{1}H NMR of *O*-(4-methoxybenzoyl)hydroxylammonium trifluoromethanesulfonate in **2d** MeOD-d[4]

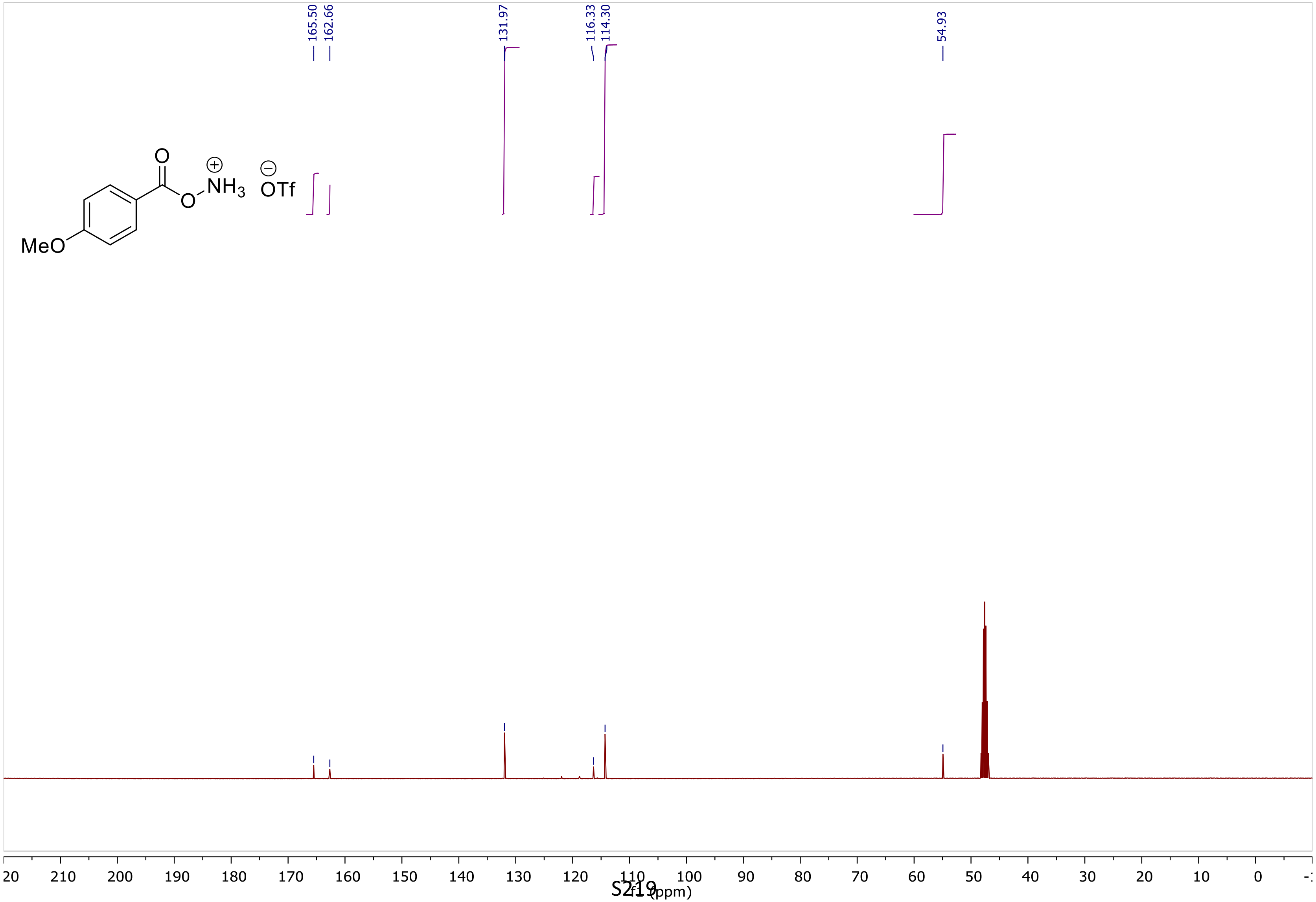

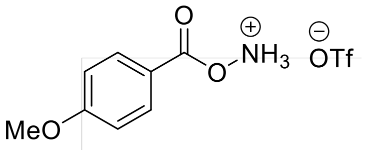

^{19}F NMR of *O*-(4-methoxybenzoyl)hydroxylammonium trifluoromethanesulfonate **2d** in DMSO-d^6

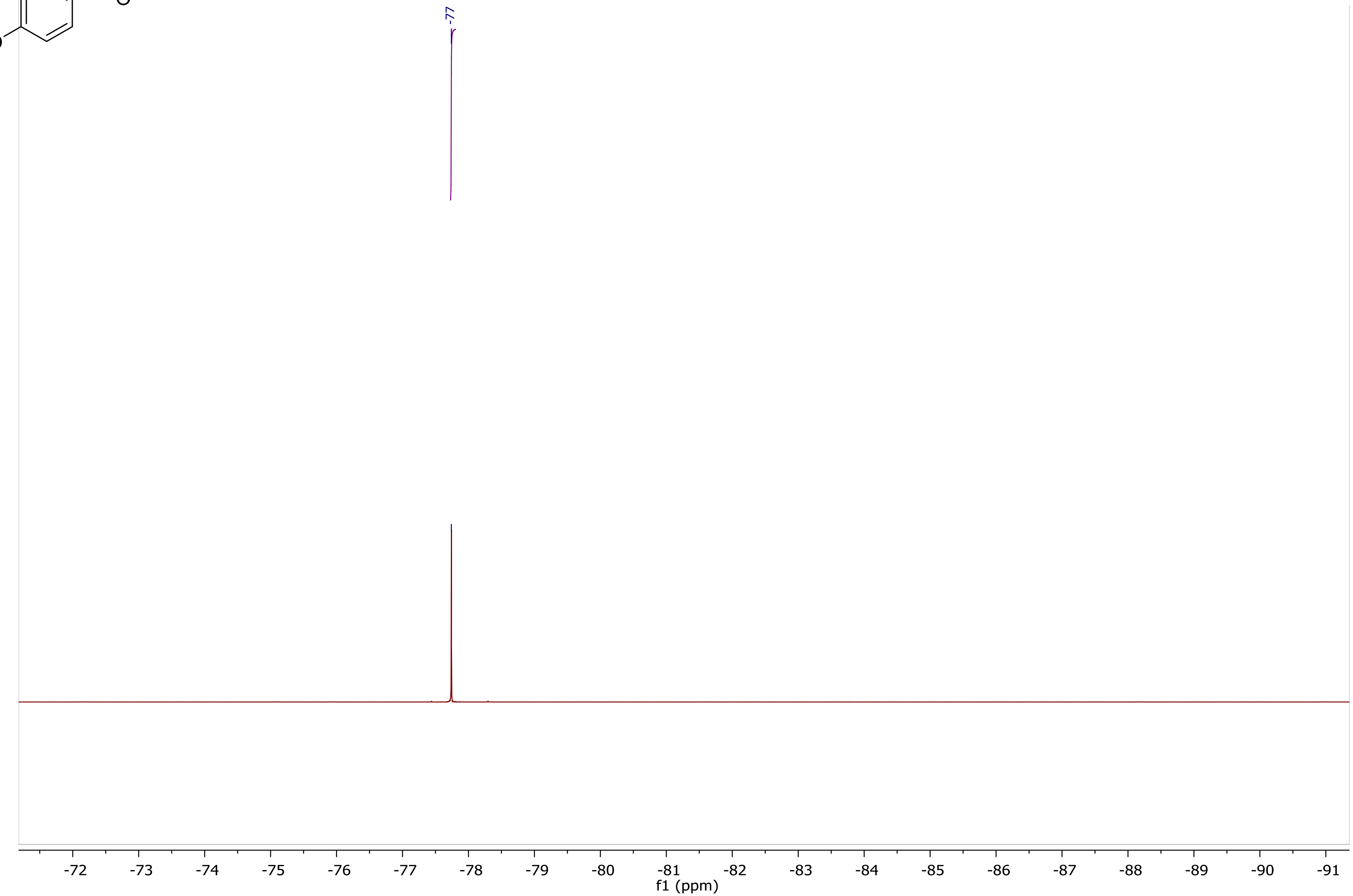

^{1}H NMR of *tert*-butyl (pivaloyloxy)carbamate in $CDCl_3$

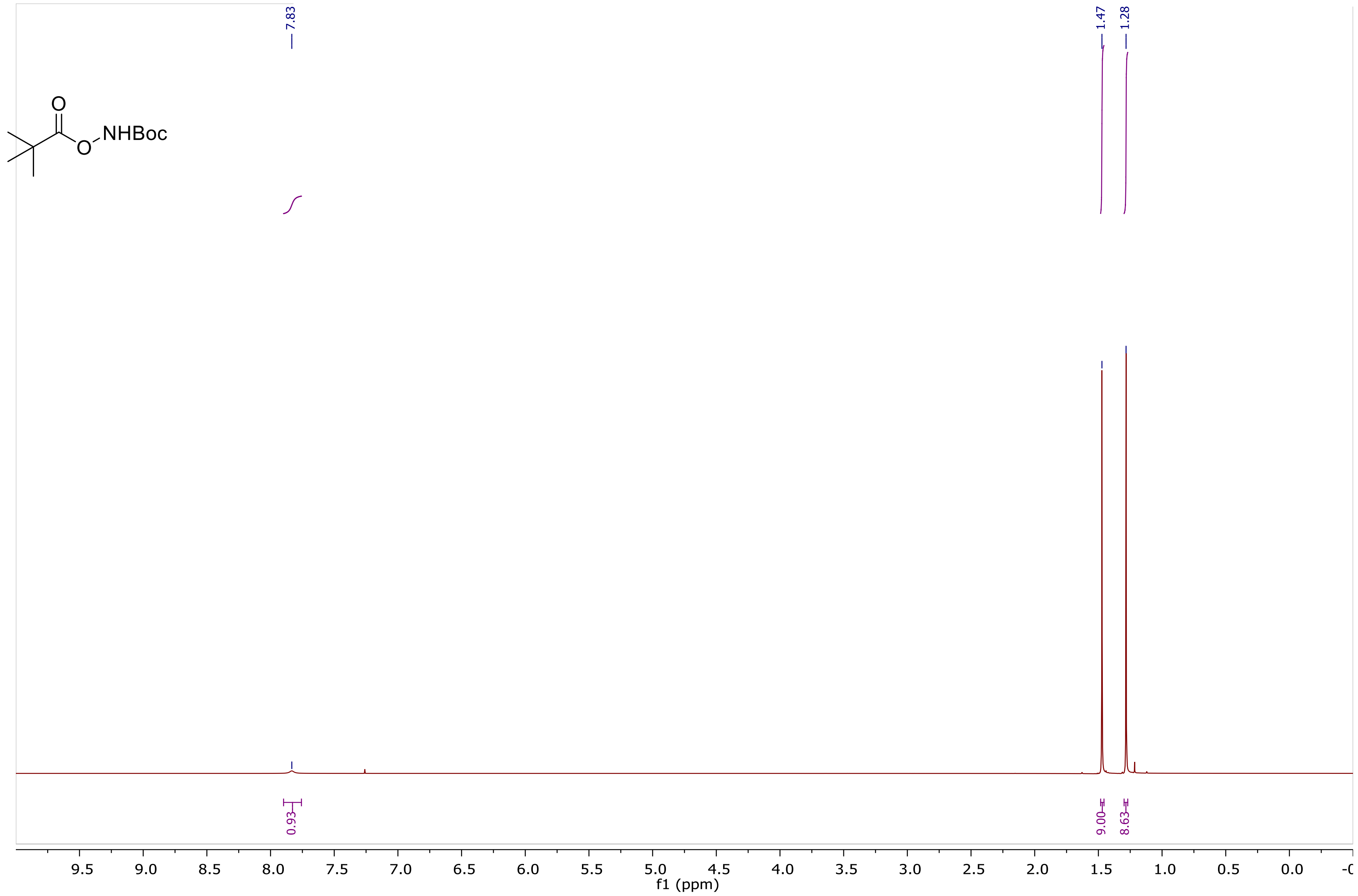

^{13}C NMR of *tert*-butyl (pivaloyloxy)carbamate in $CDCl_3$

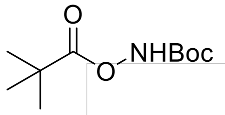

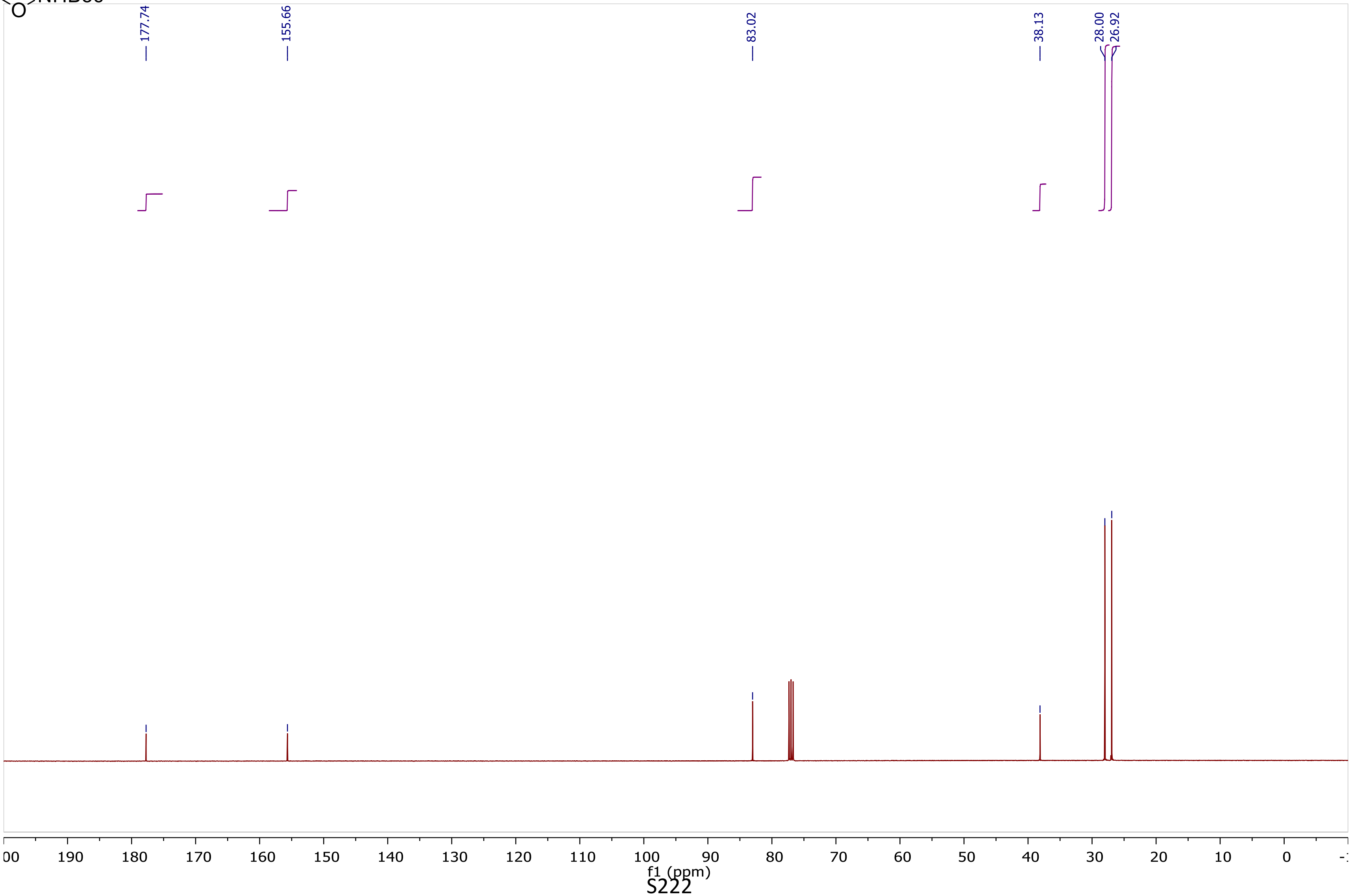

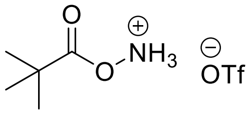

^{1}H NMR of *O*-pivaloylhydroxylammonium trifluoromethanesulfonate **2e** in CD_3CN

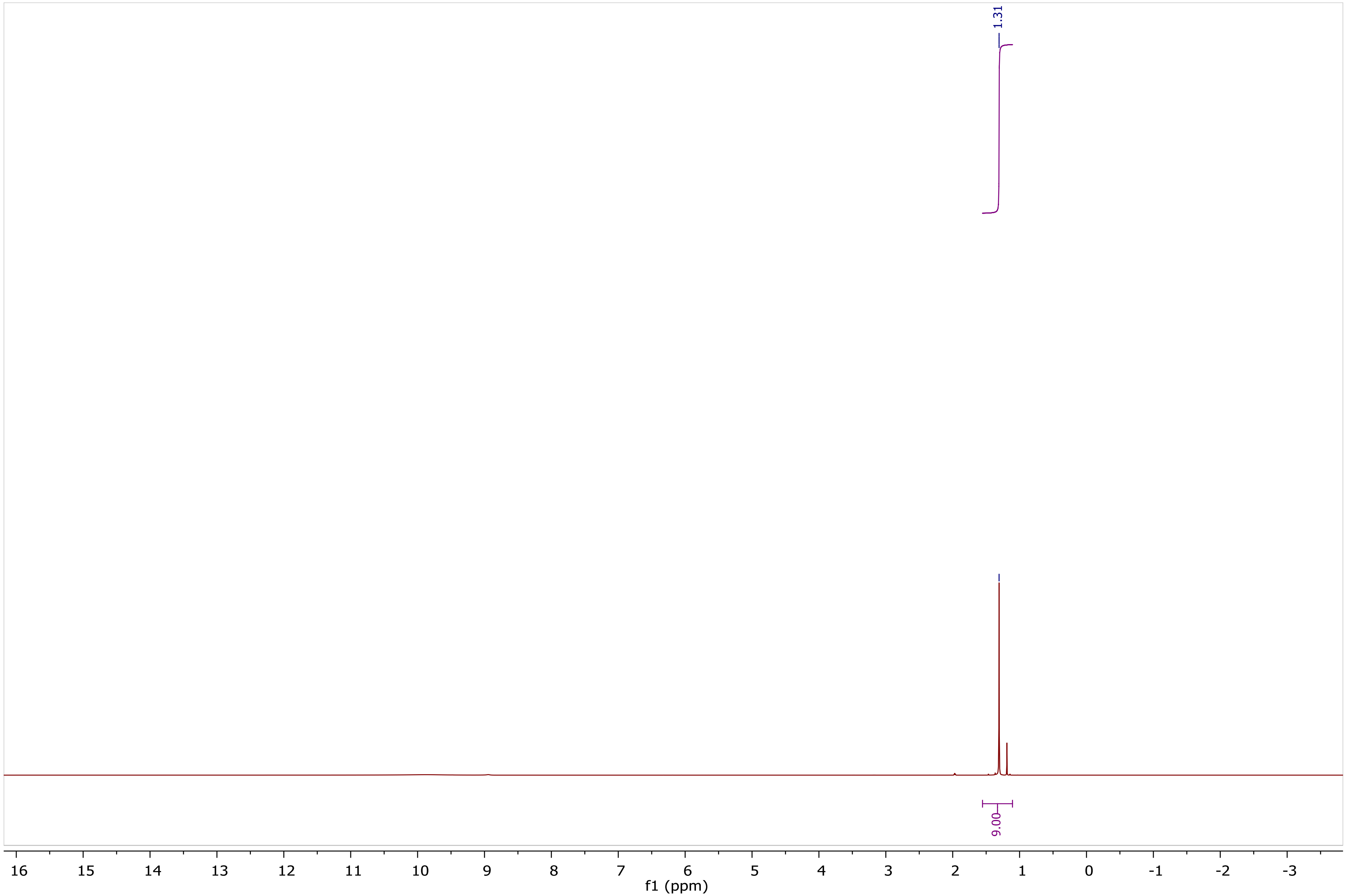

^{13}C NMR of *O*-pivaloylhydroxylammonium trifluoromethanesulfonate **2e** in CD_3CN

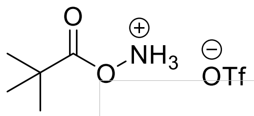

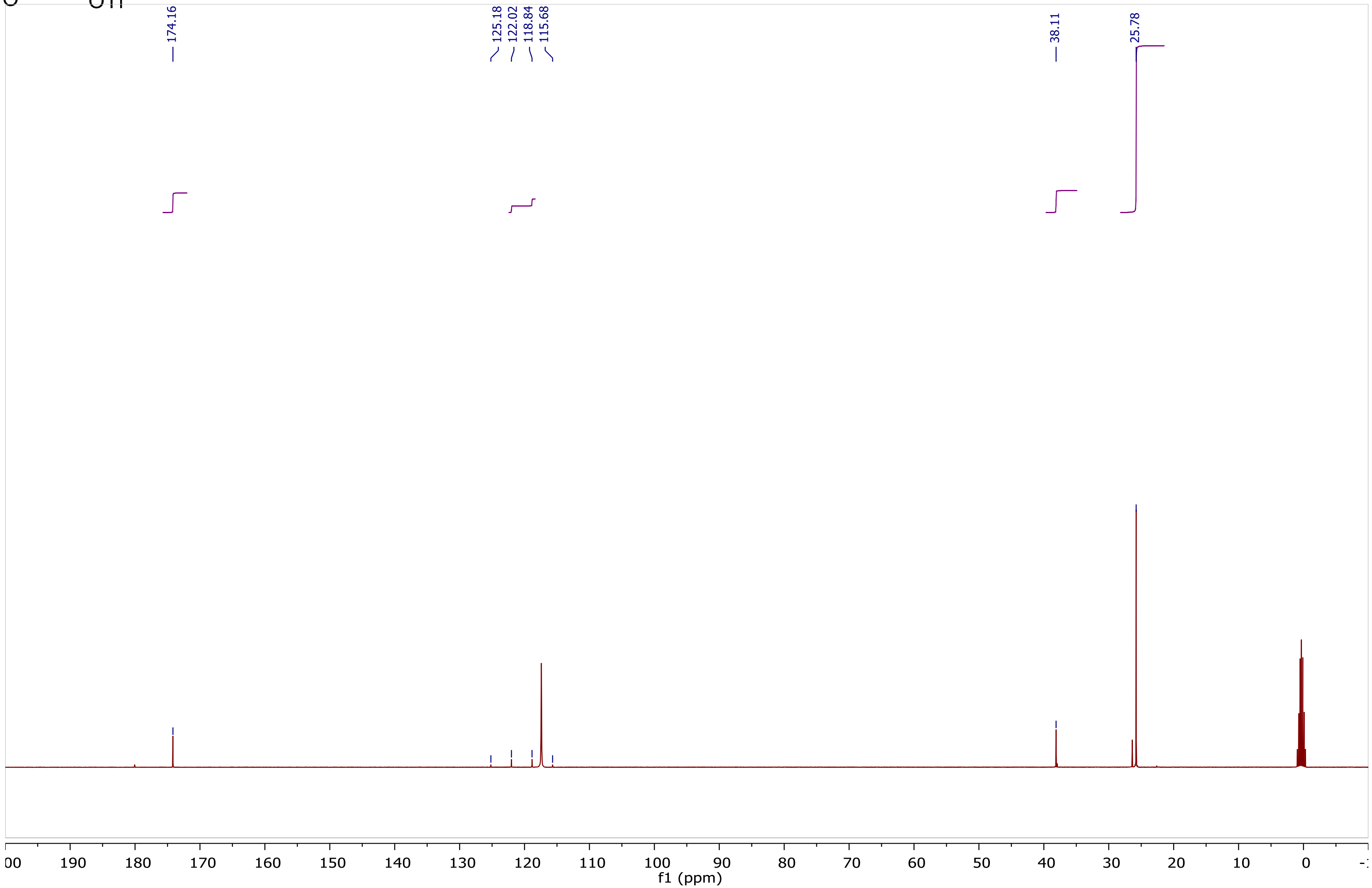

^{19}F NMR of *O*-pivaloylhydroxylammonium trifluoromethanesulfonate **2e** in CD_3CN

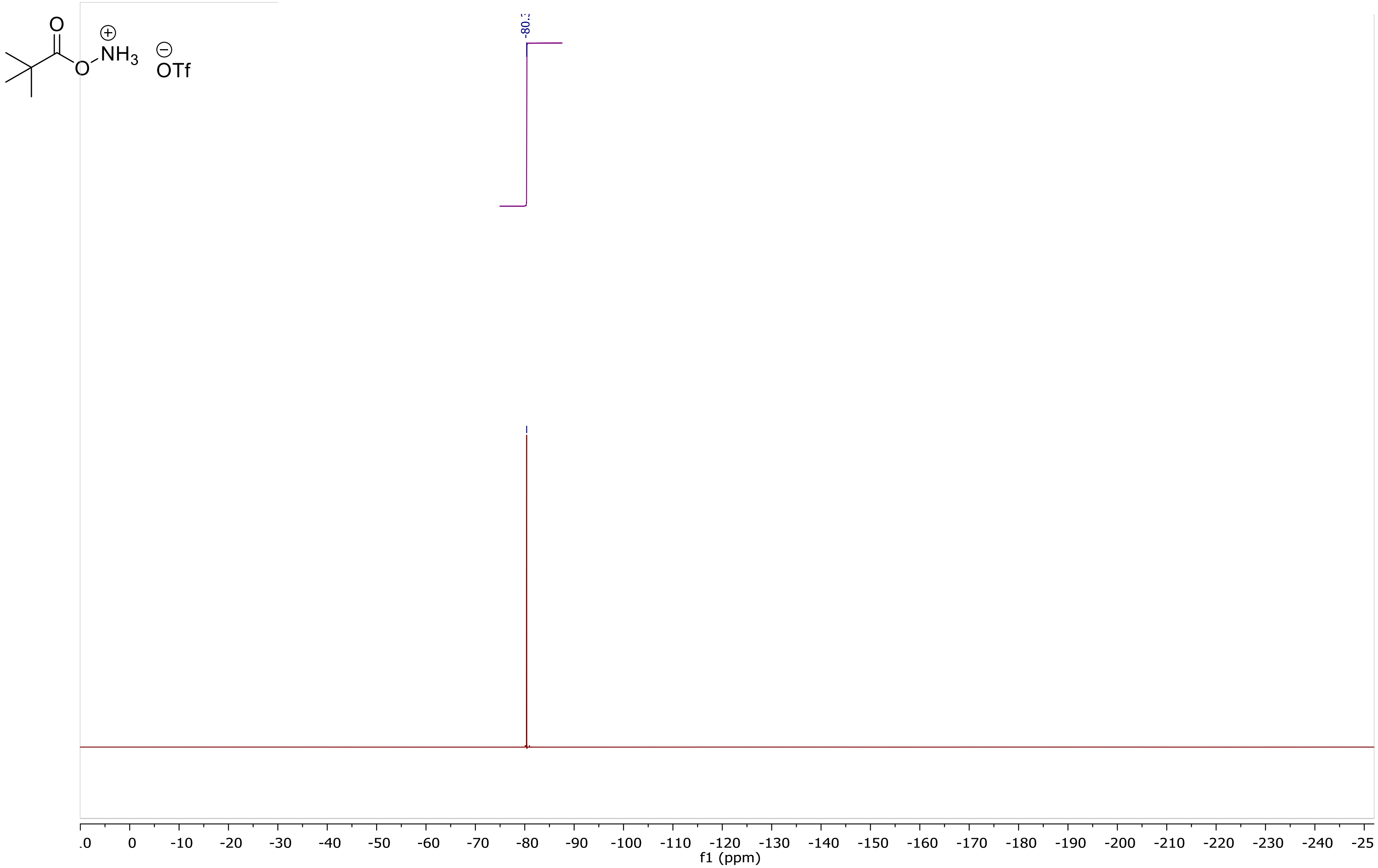

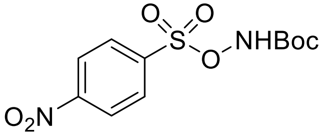

^{1}H NMR of *tert*-butyl (((4-nitrophenyl)sulfonyl)oxy)carbamate in $CDCl_3$

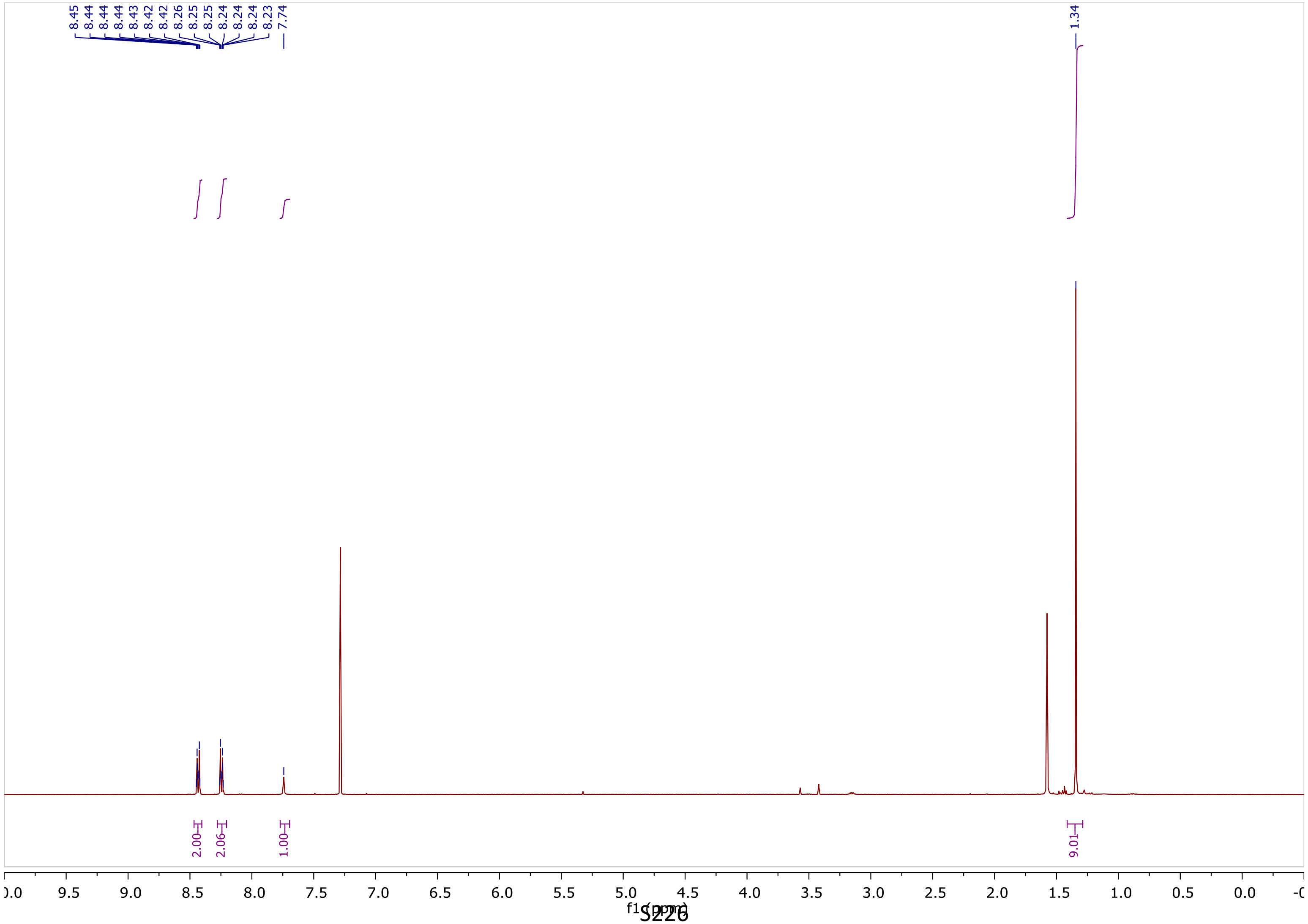

^{13}C NMR of *tert*-butyl (((4-nitrophenyl)sulfonyl)oxy)carbamate in $CDCl_3$

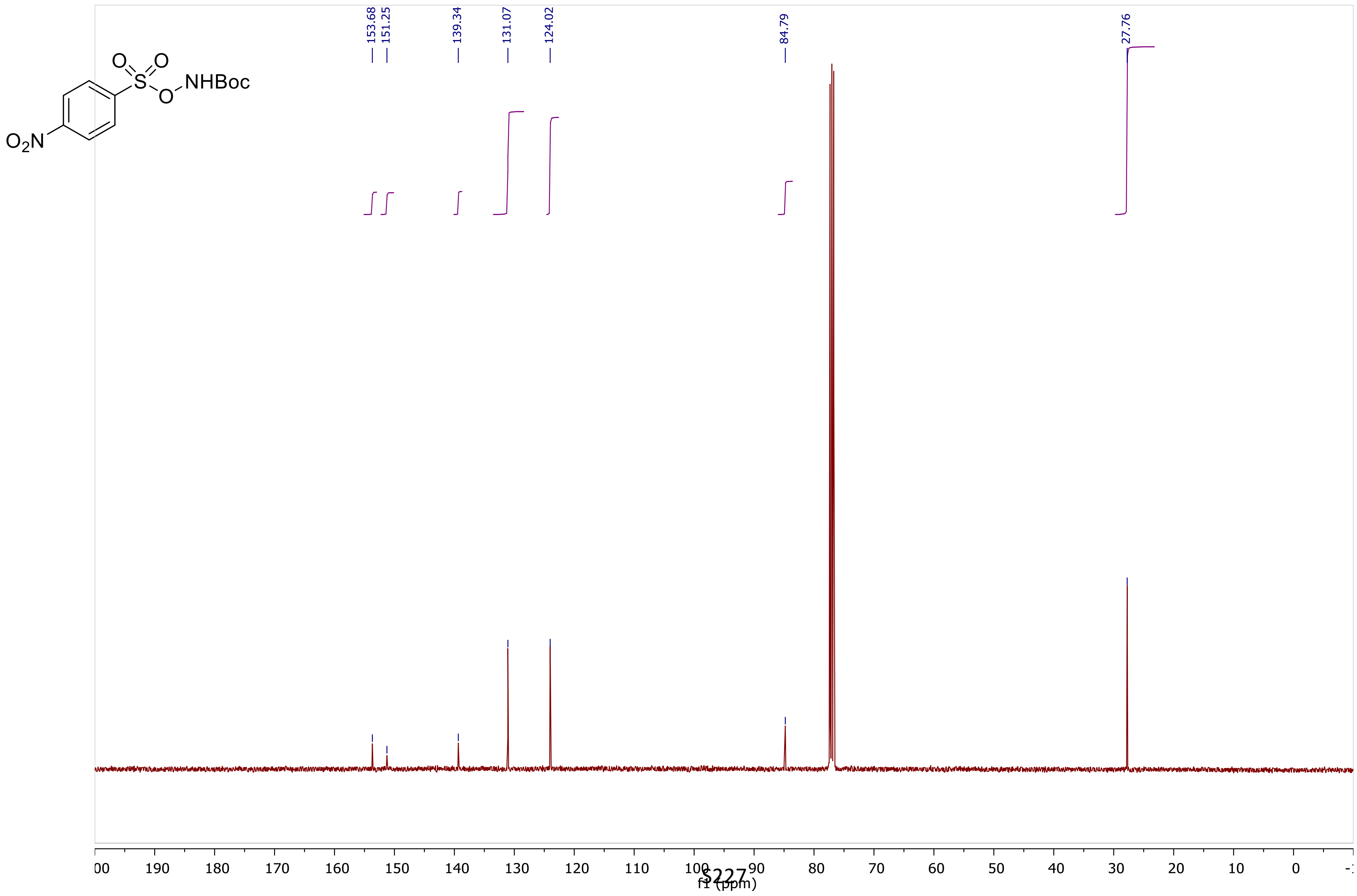

^{1}H NMR of O-((4-nitrophenyl)sulfonyl)hydroxylammonium trifluoromethanesulfonate **2g** in DMSO-d6

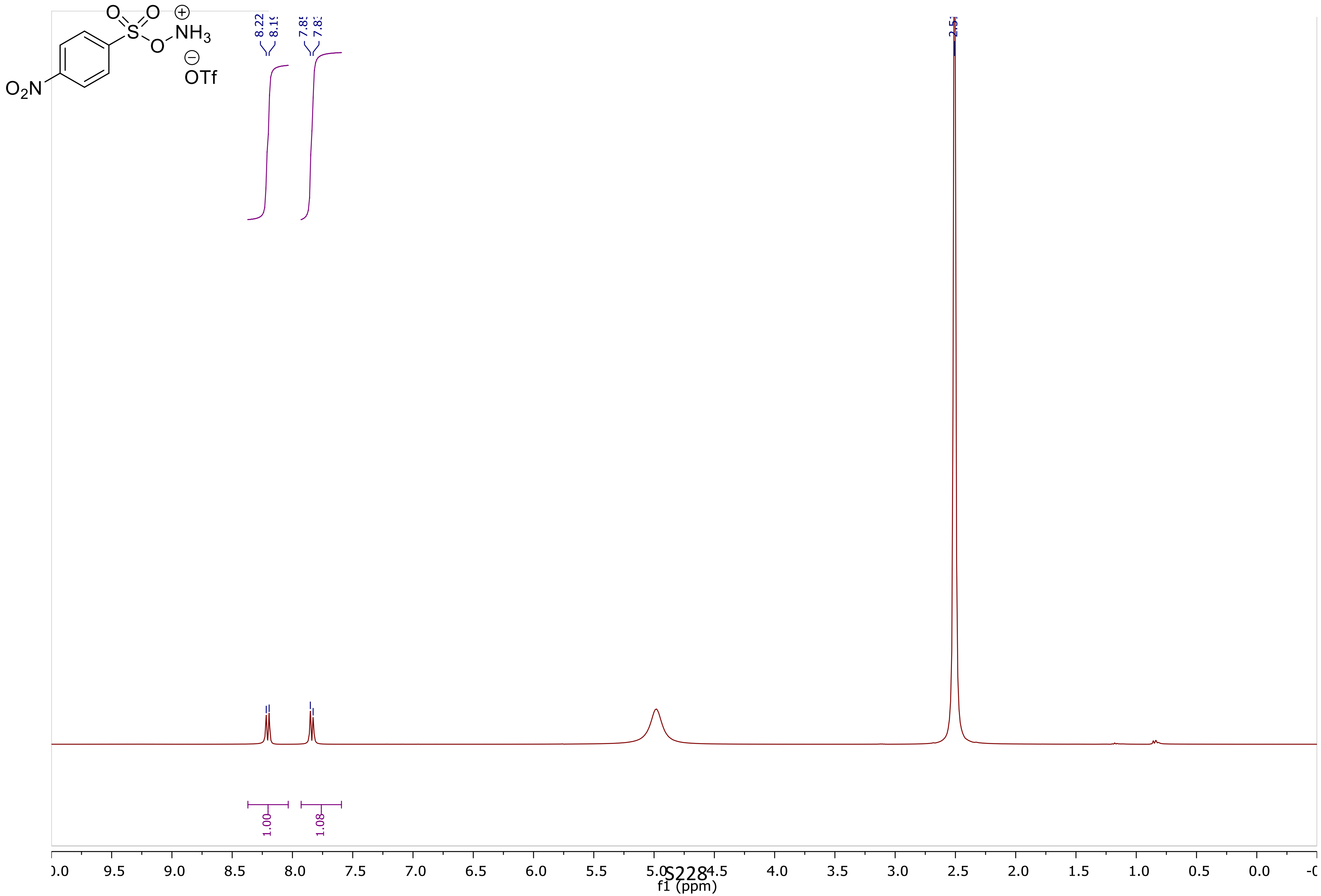

^{13}C NMR of O-((4-nitrophenyl)sulfonyl)hydroxylammonium trifluoromethanesulfonate **2g** in DMSO-d6

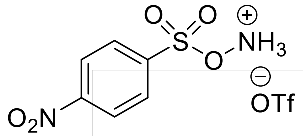

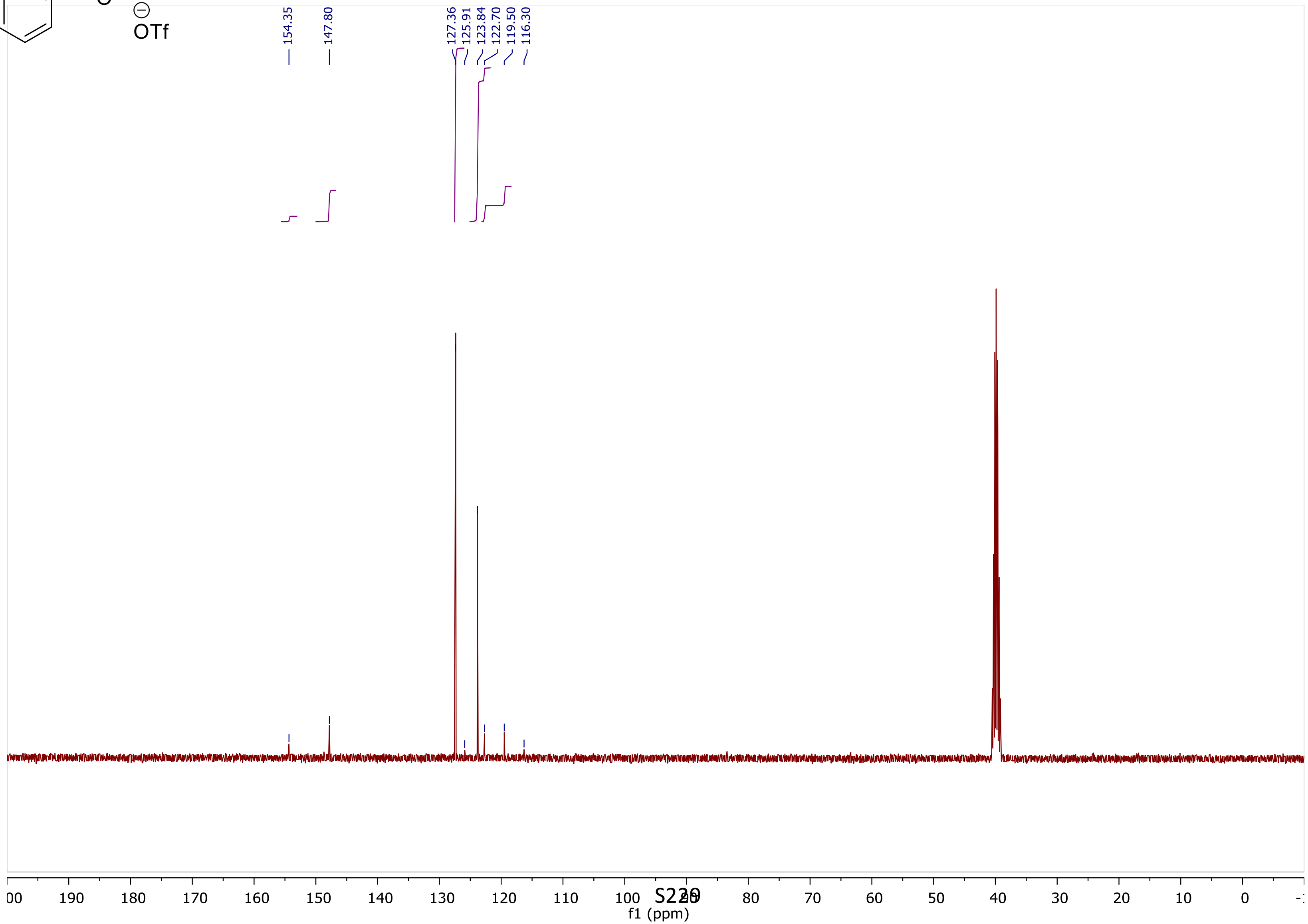

^{19}F NMR of O-((4-nitrophenyl)sulfonyl)hydroxylammonium trifluoromethanesulfonate **2g** in DMSO-d6

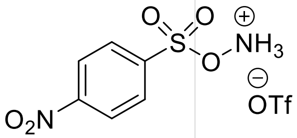

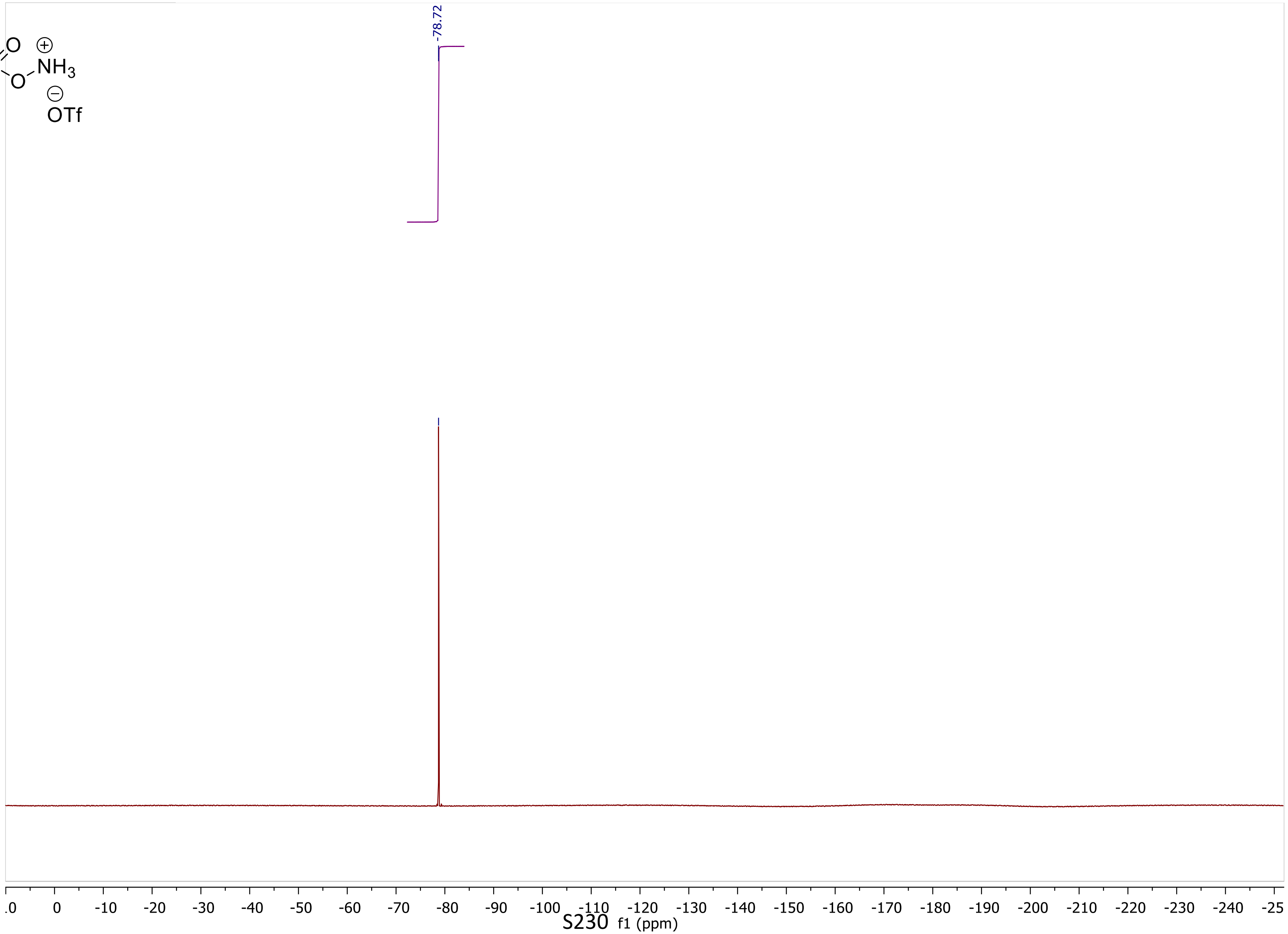

^{1}H NMR of *tert*-butyl (4-methoxybenzoyl)oxy)(methyl)carbamate in $CDCl_3$

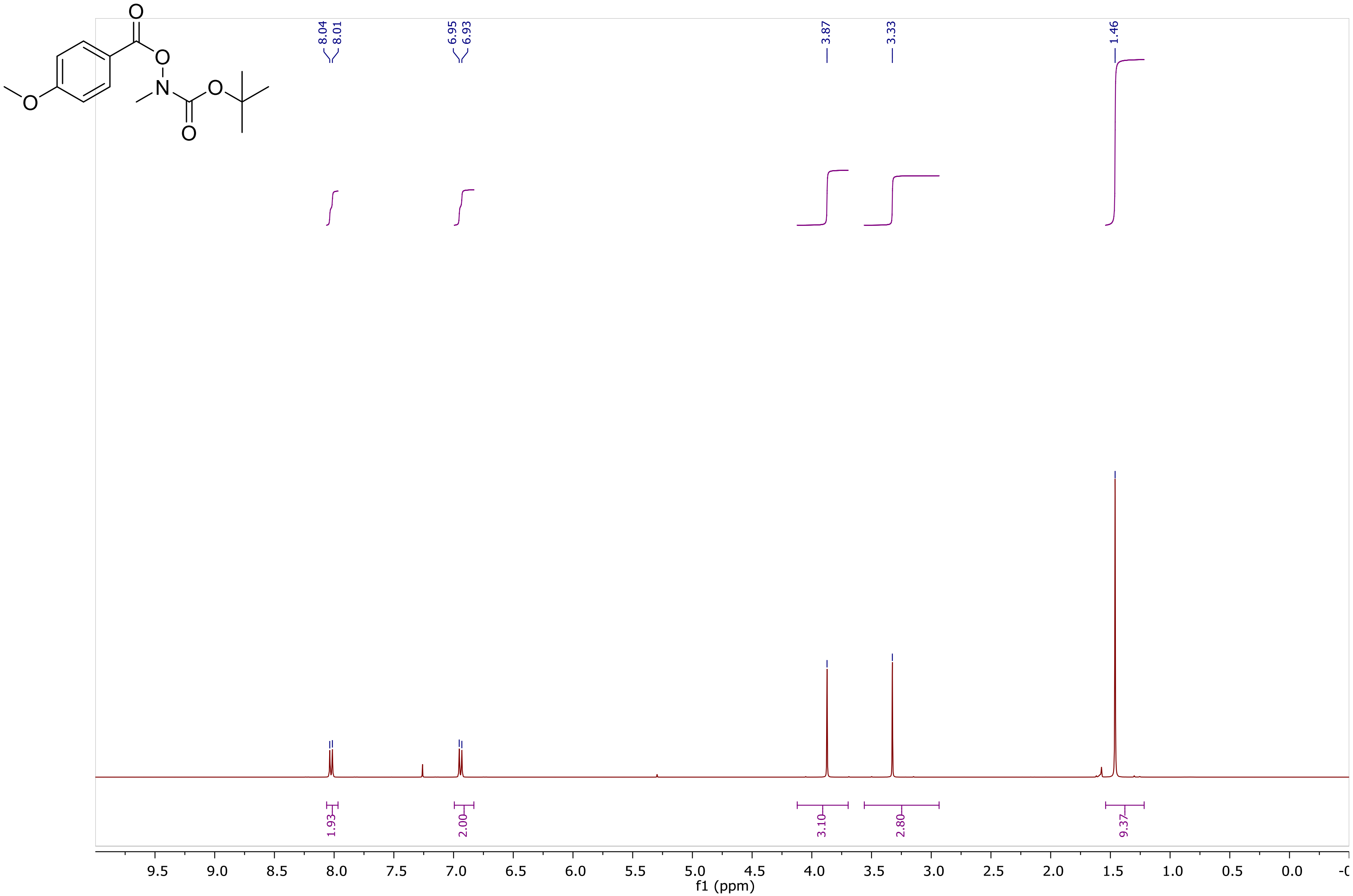

^{13}C NMR of *tert*-butyl (4-methoxybenzoyl)oxy)(methyl)carbamate in $CDCl_3$

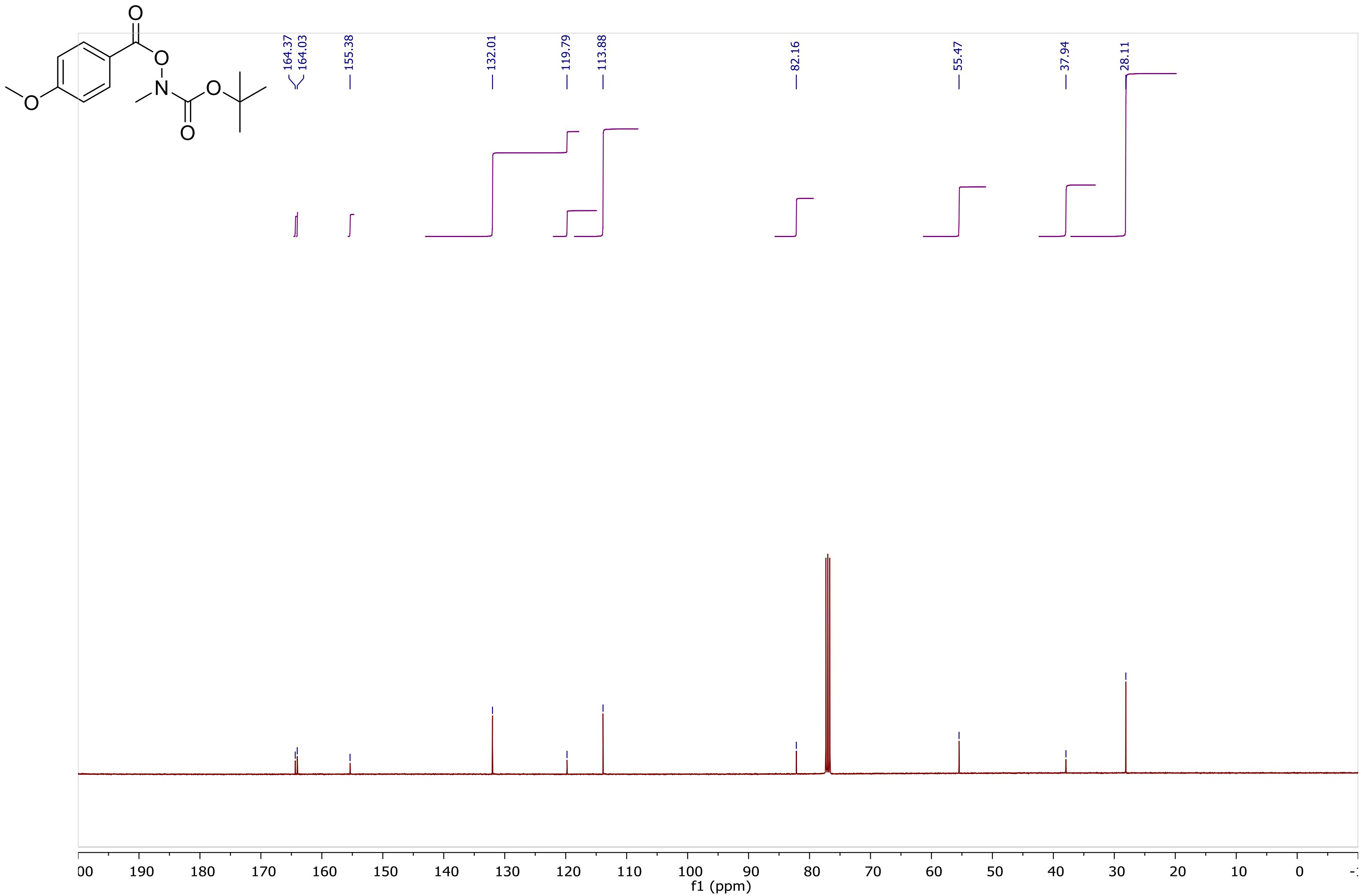

^{1}H NMR of *O*-(4-methoxybenzoyl)-*N*-methylhydroxylammonium trifluoromethanesulfonate **3d** in CD_3CN

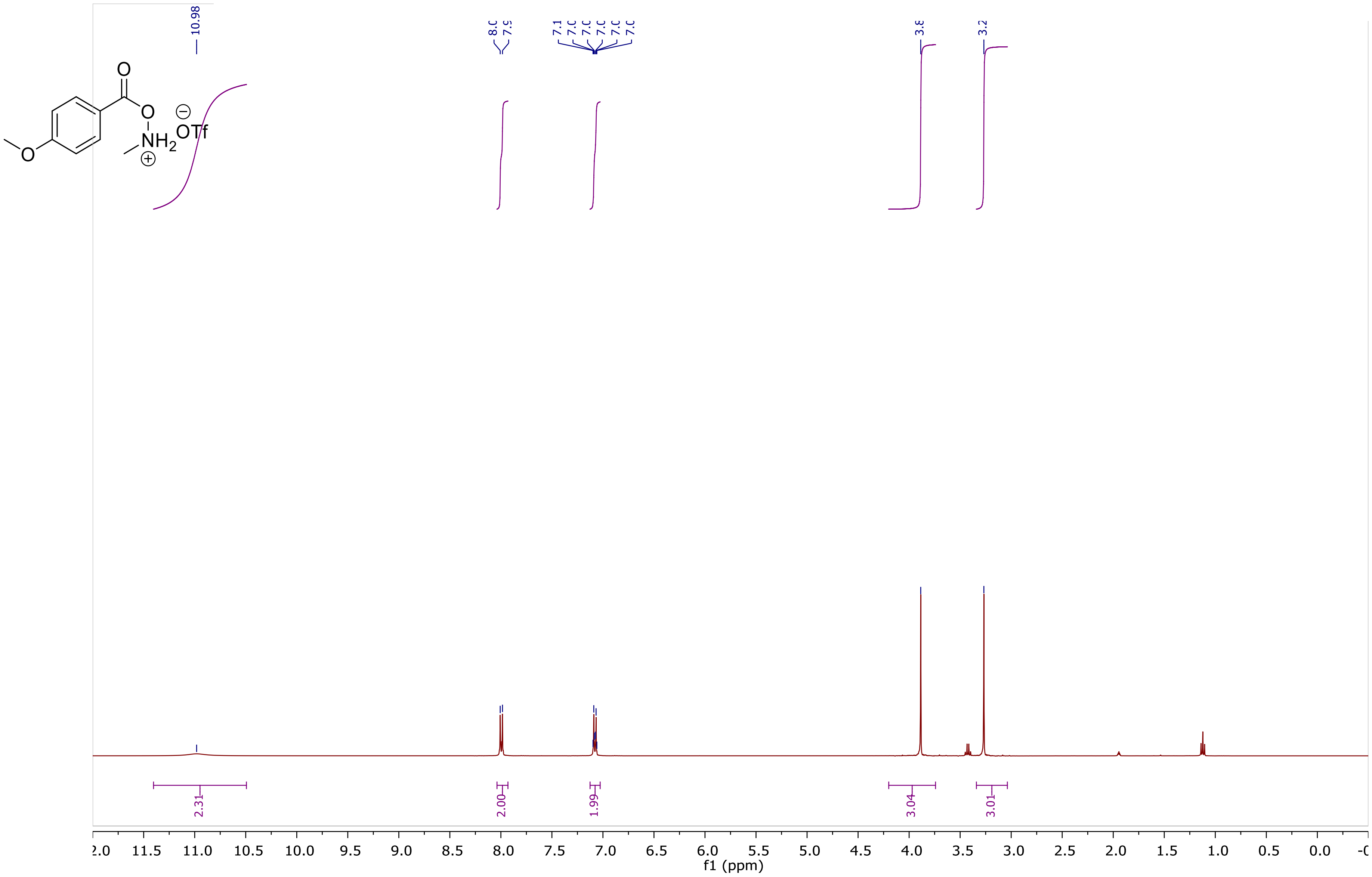

^{13}C NMR of *O*-(4-methoxybenzoyl)-*N*-methylhydroxylammonium trifluoromethanesulfonate **3d** in CD_3CN

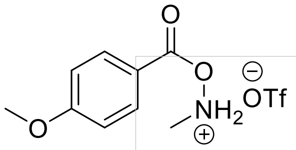

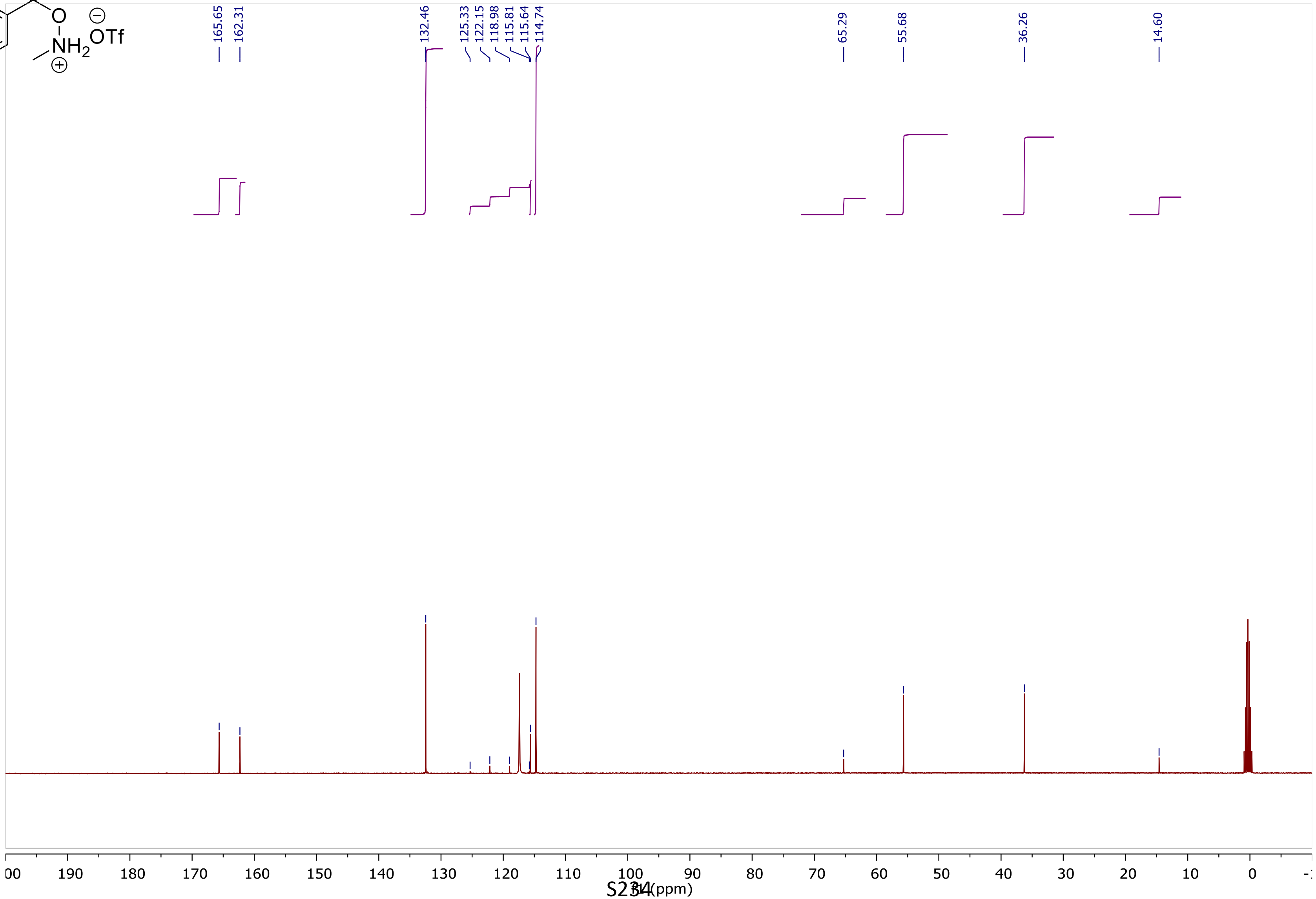

^{19}F NMR of *O*-(4-methoxybenzoyl)-*N*-methylhydroxylammonium trifluoromethanesulfonate **3d** in CD_3CN

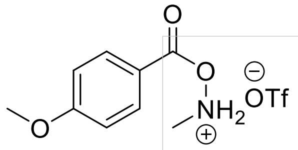

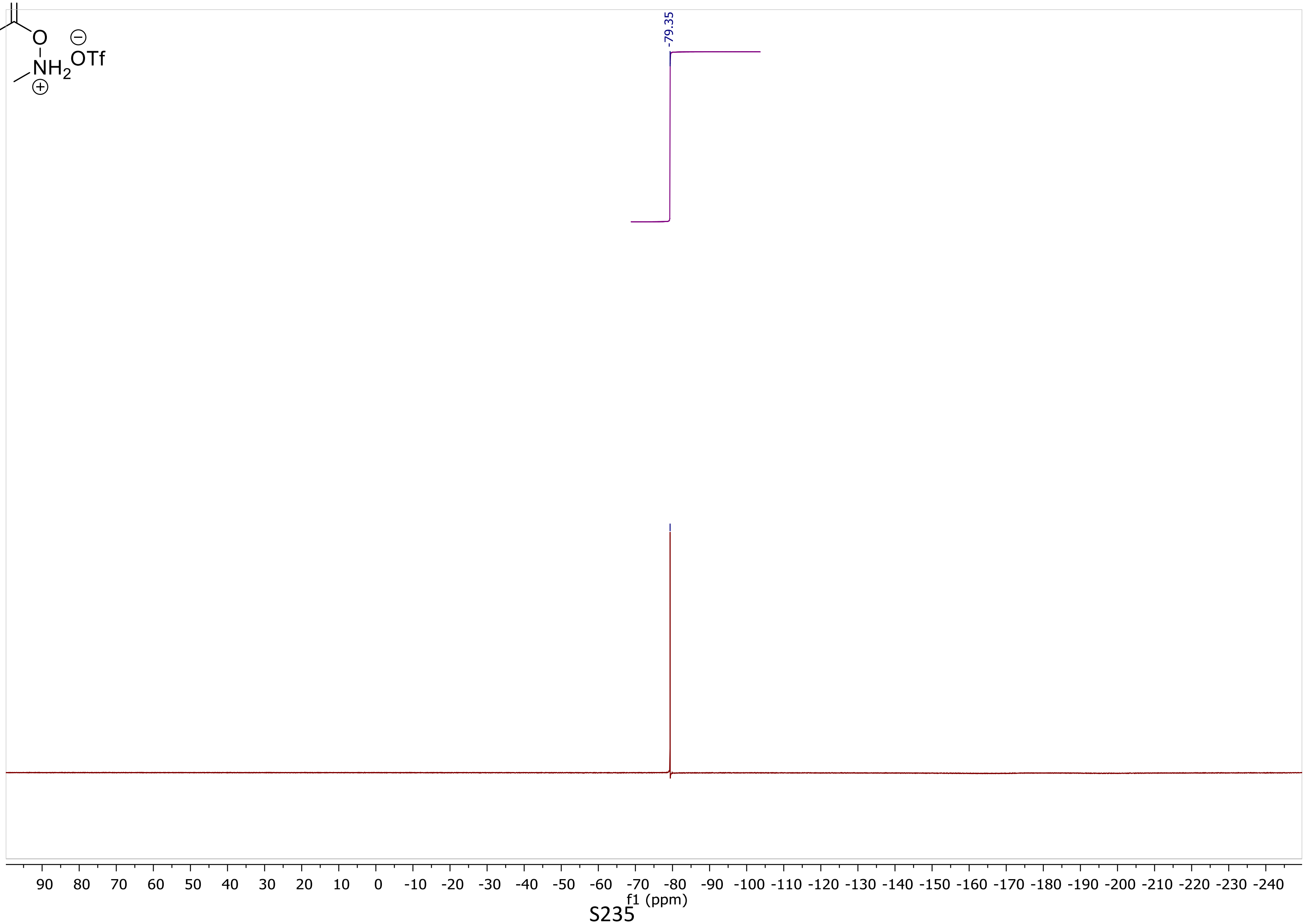

^{1}H NMR of *tert*-butyl (4-nitrobenzoyl)oxy)(methyl)carbamate in $CDCl_3$

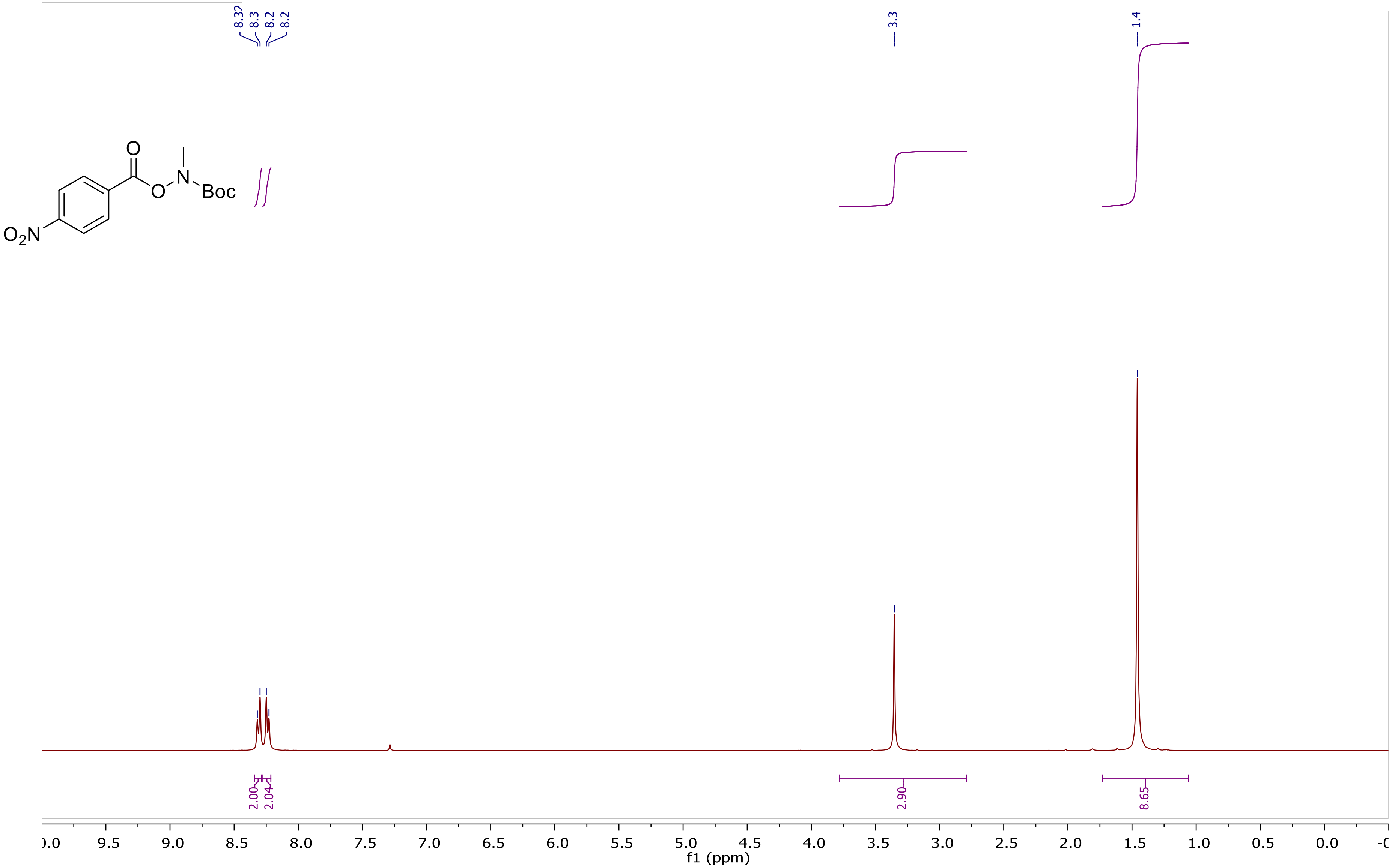

^{13}C NMR of *tert*-butyl (4-nitrobenzoyl)oxy)(methyl)carbamate in $CDCl_3$

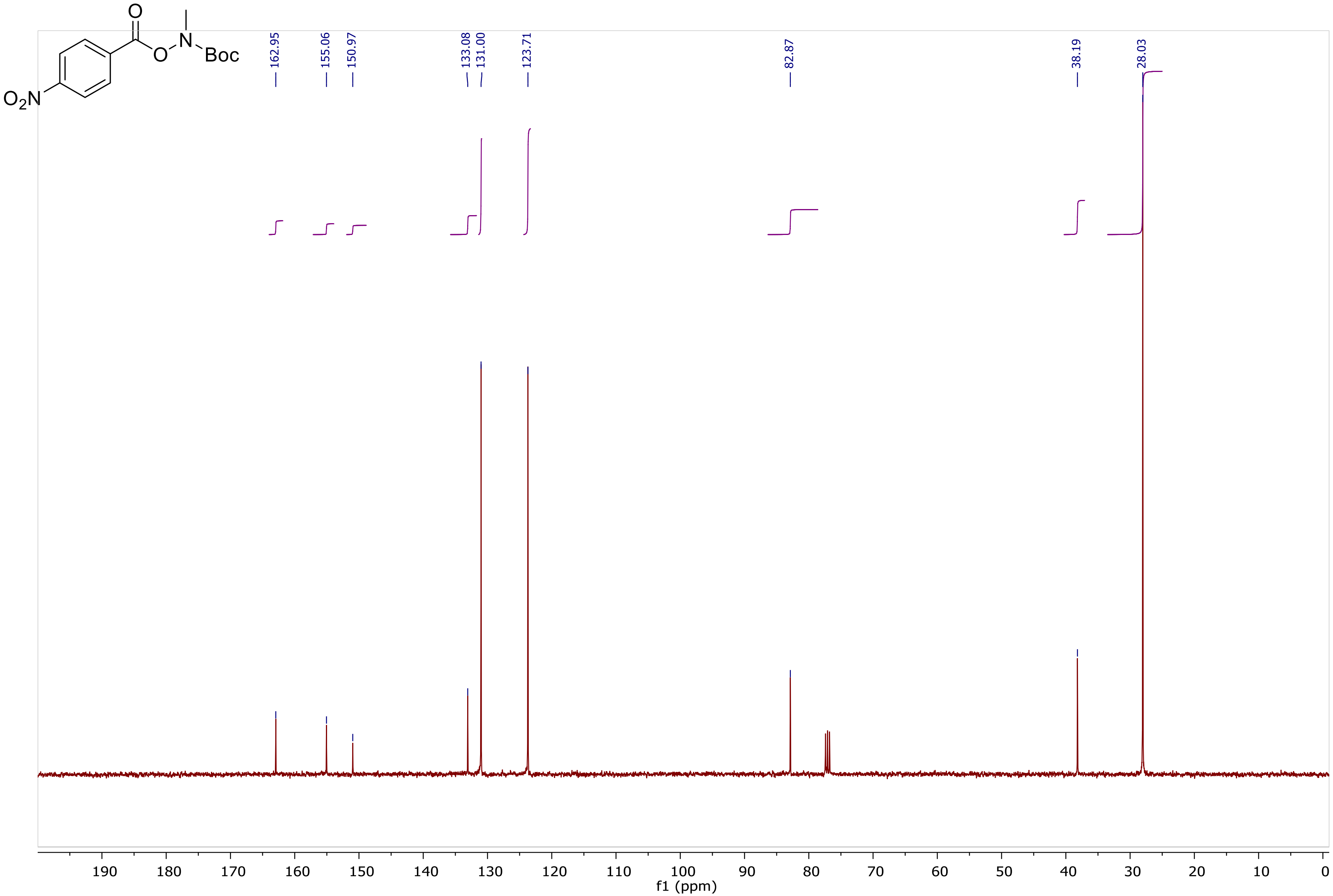

^{1}H NMR of *O*-(4-nitrobenzoyl)-*N*-methylhydroxylammonium trifluoromethanesulfonate **3b** in CD_3CN

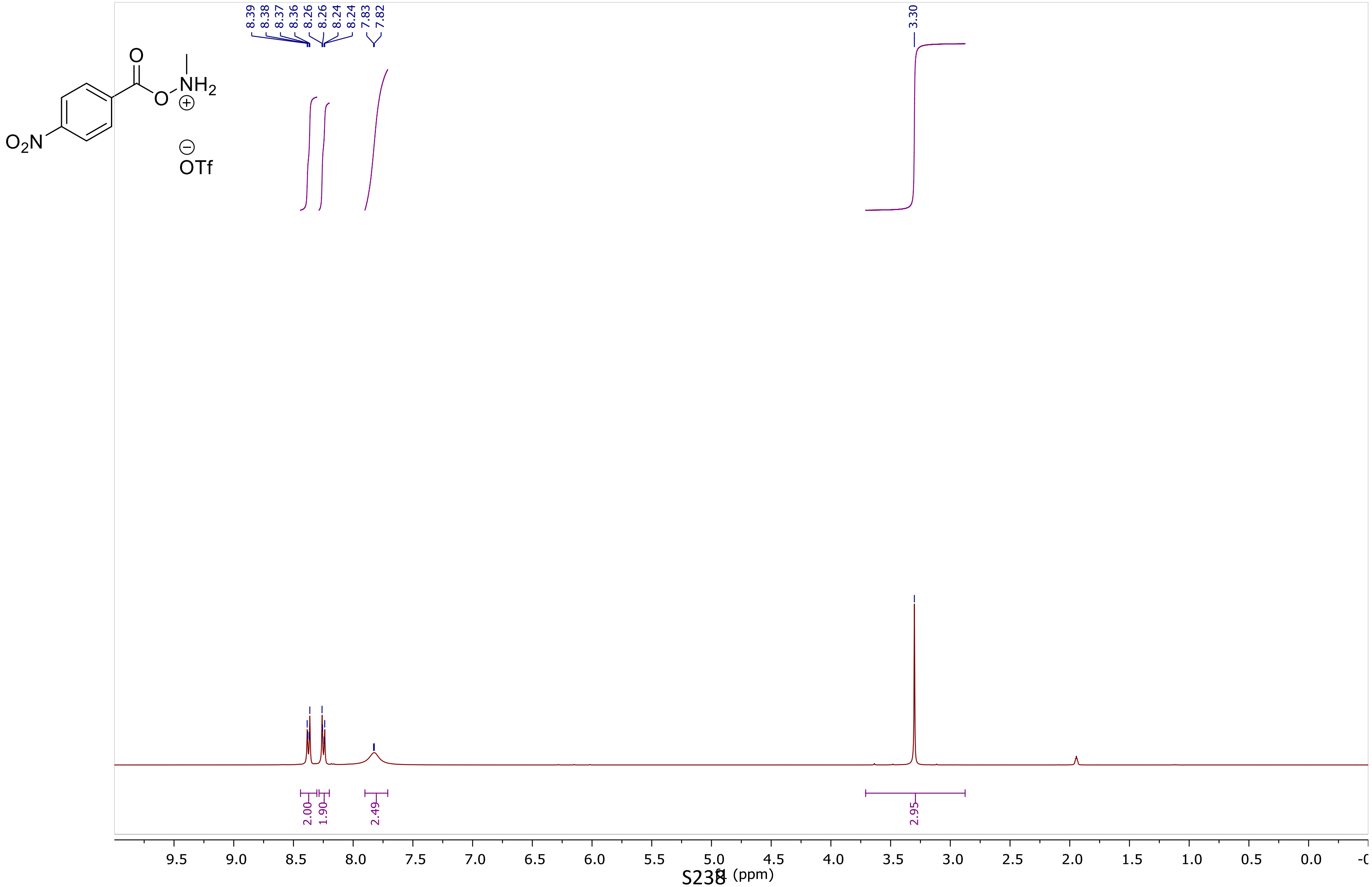

^{13}C NMR of *O*-(4-nitrobenzoyl)-*N*-methylhydroxylammonium trifluoromethanesulfonate **3b** in CD_3CN

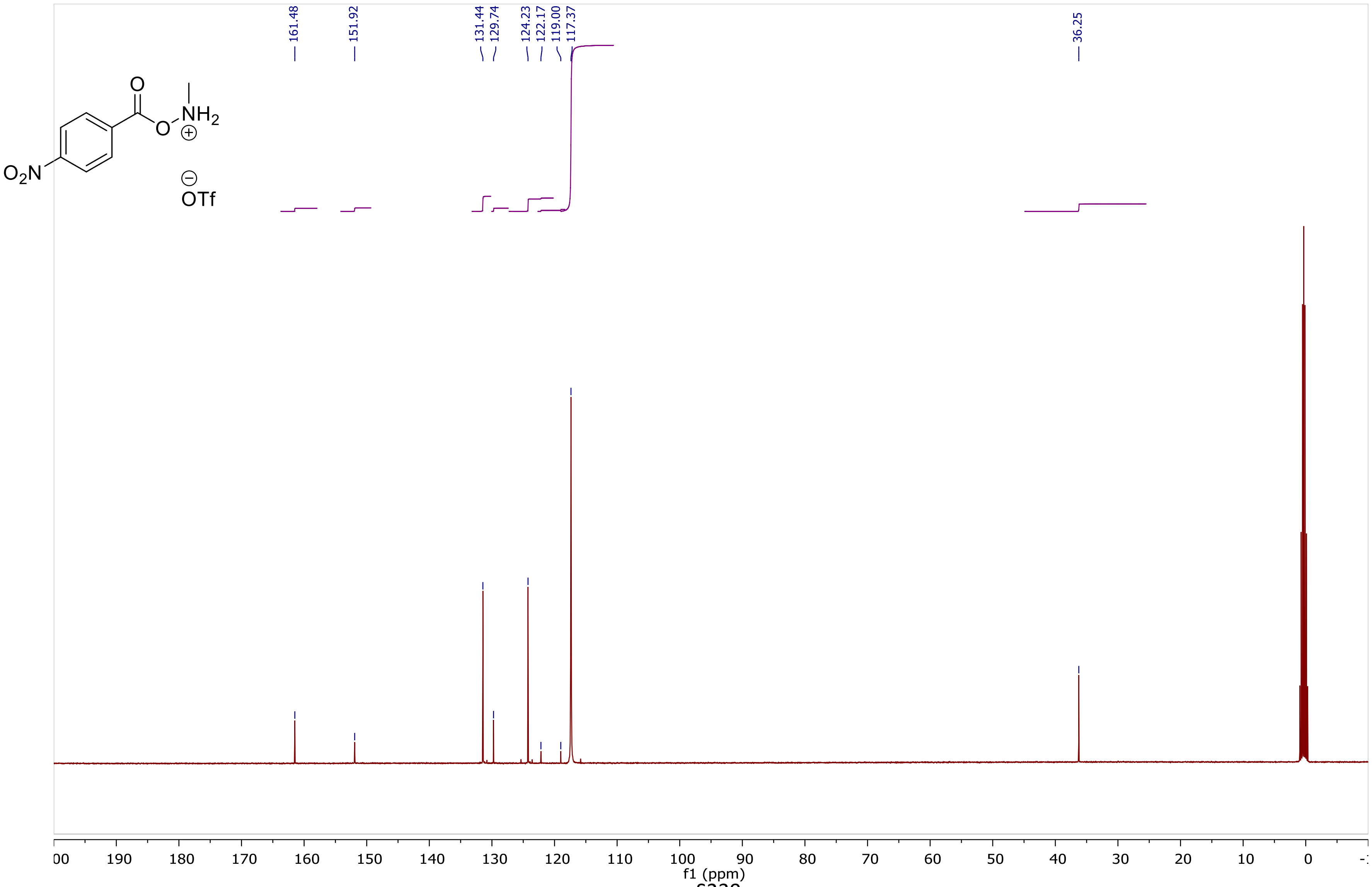

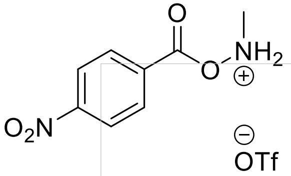

^{19}F NMR of *O*-(4-nitrobenzoyl)-*N*-methylhydroxylammonium trifluoromethanesulfonate **3b** in CD_3CN

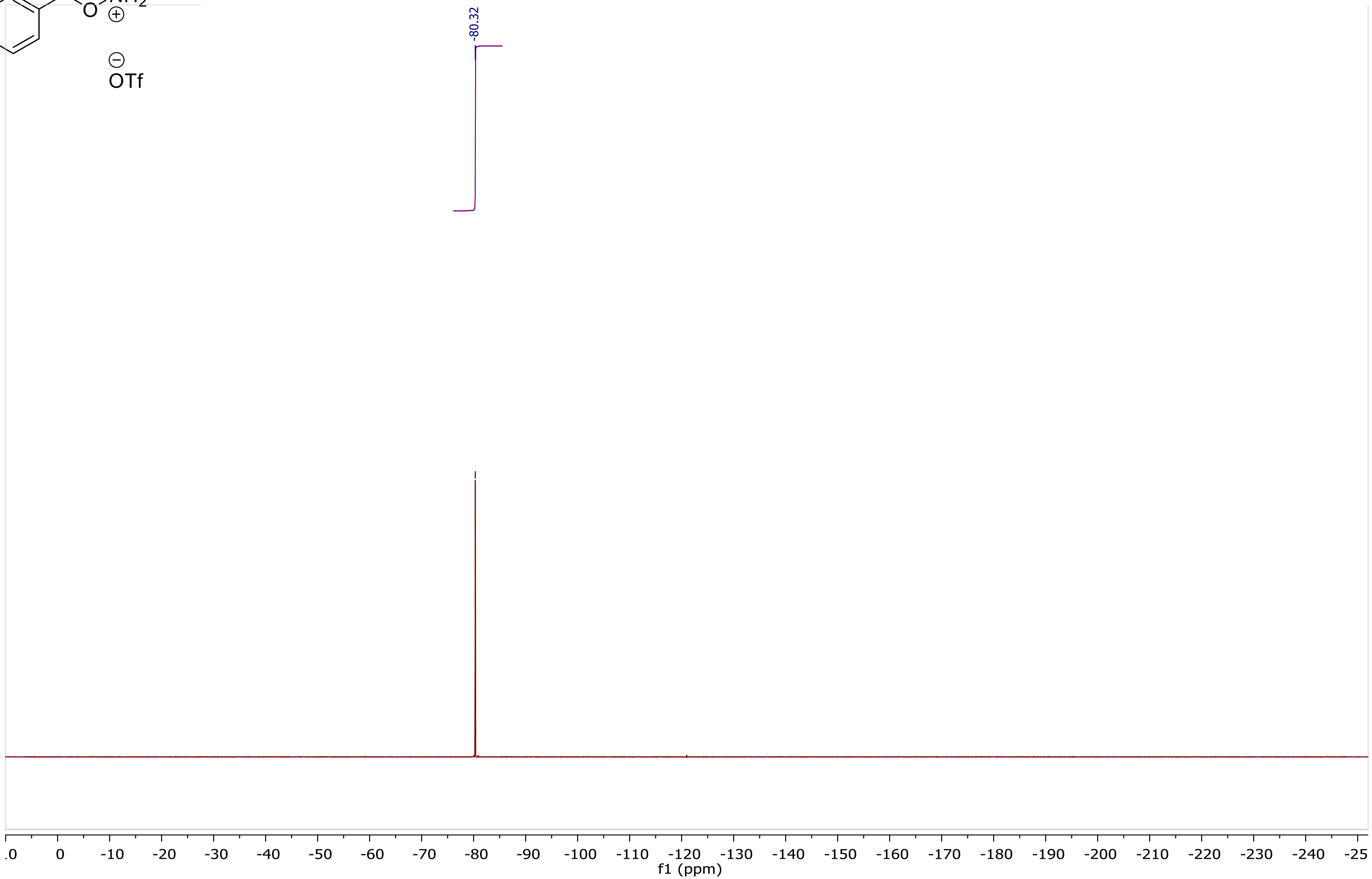

^{1}H NMR of *tert*-butyl ((3,5-bis(trifluoromethyl)benzoyl)oxy)(methyl)carbamate in $CDCl_3$

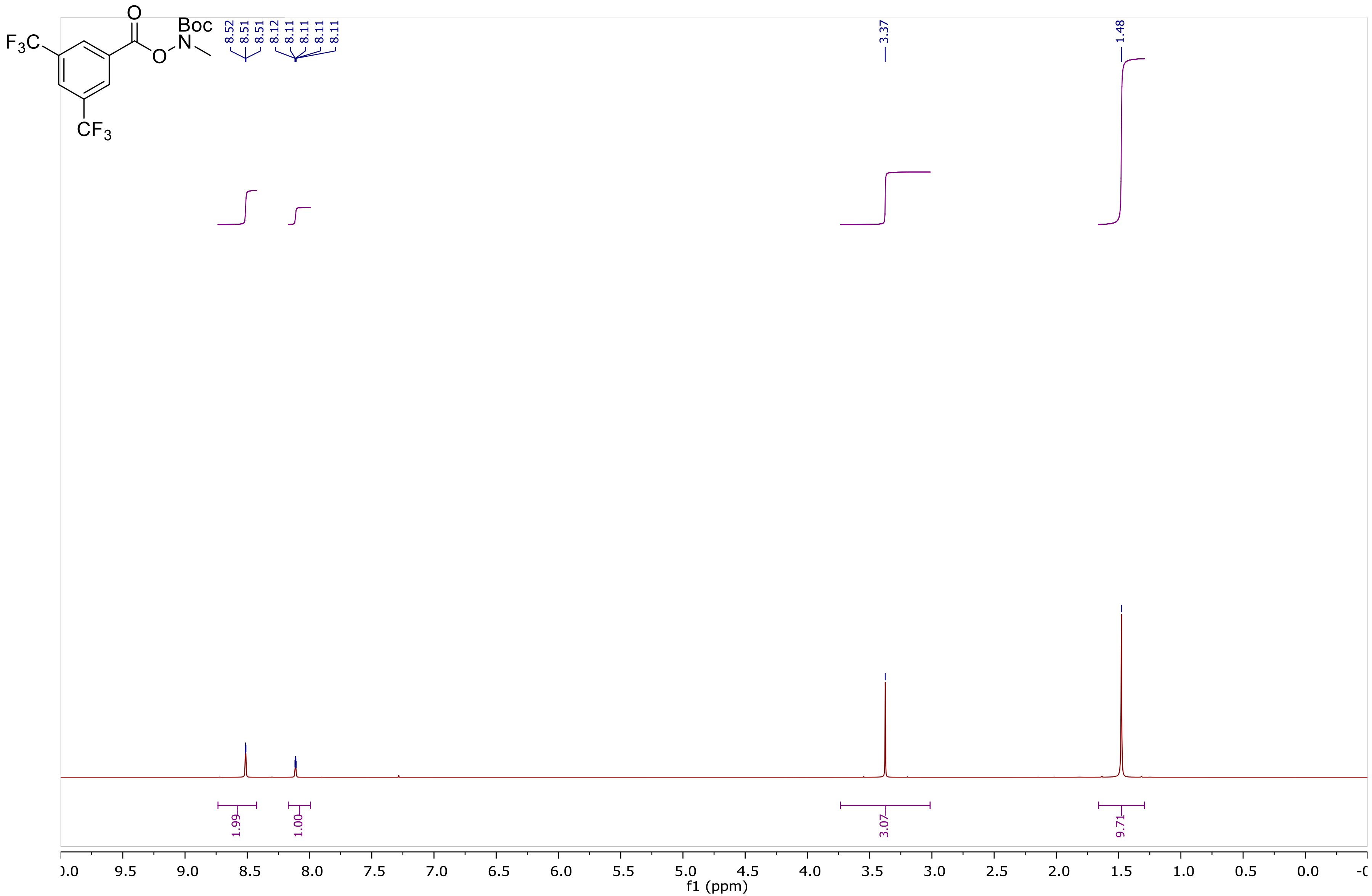

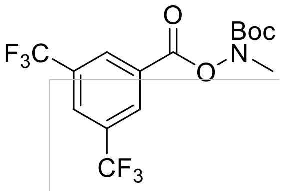

^{13}C NMR of *tert*-butyl ((3,5-bis(trifluoromethyl)benzoyl)oxy)(methyl)carbamate in $CDCl_3$

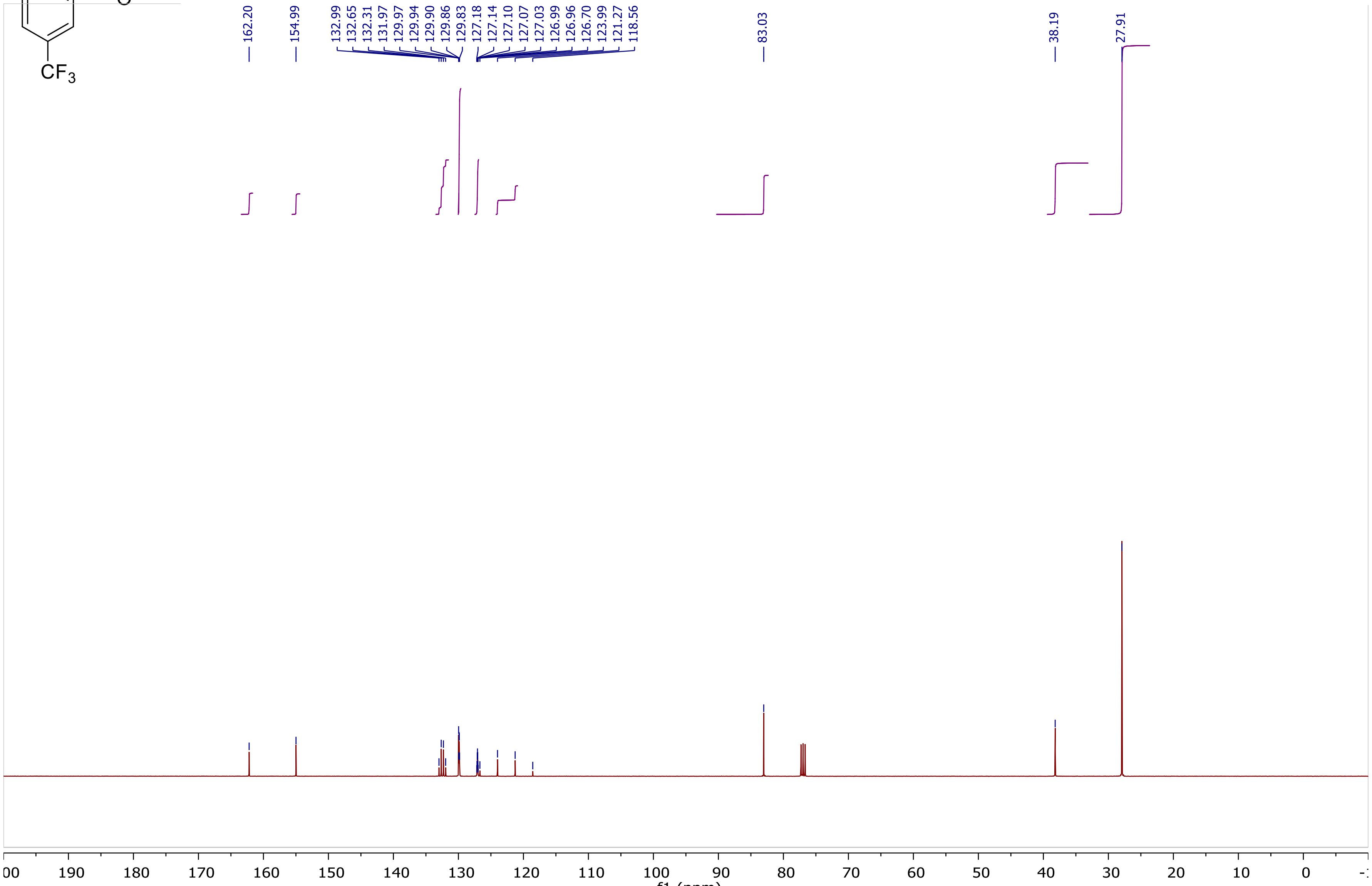

^{19}F NMR of *tert*-butyl ((3,5-bis(trifluoromethyl)benzoyl)oxy)(methyl)carbamate in $CDCl_3$

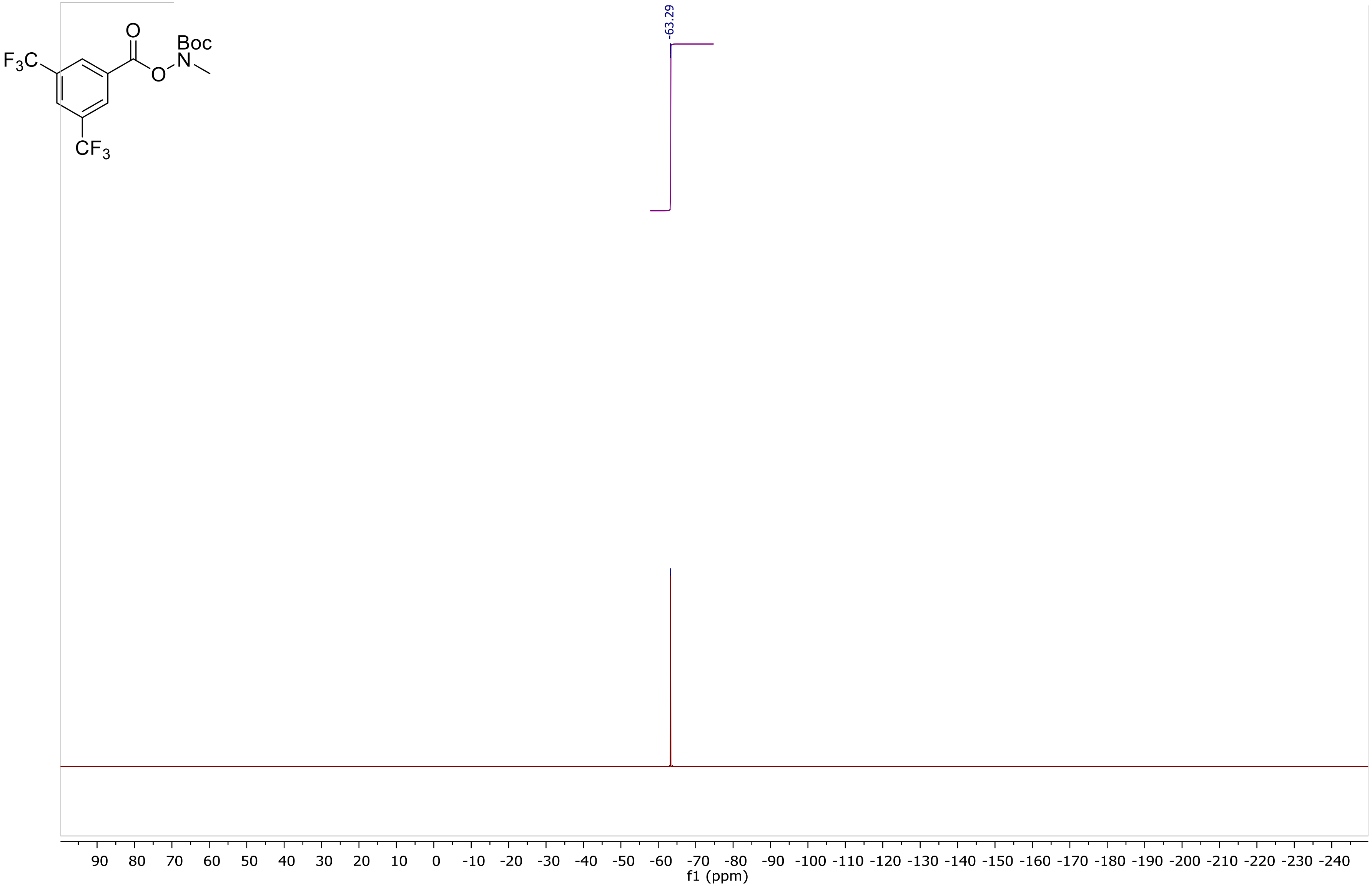

^{1}H NMR of *O*-(3,5-bis(trifluoromethyl)benzoyl)-*N*-methylhydroxylammonium trifluoromethanesulfonate **3c** in CD_3CN

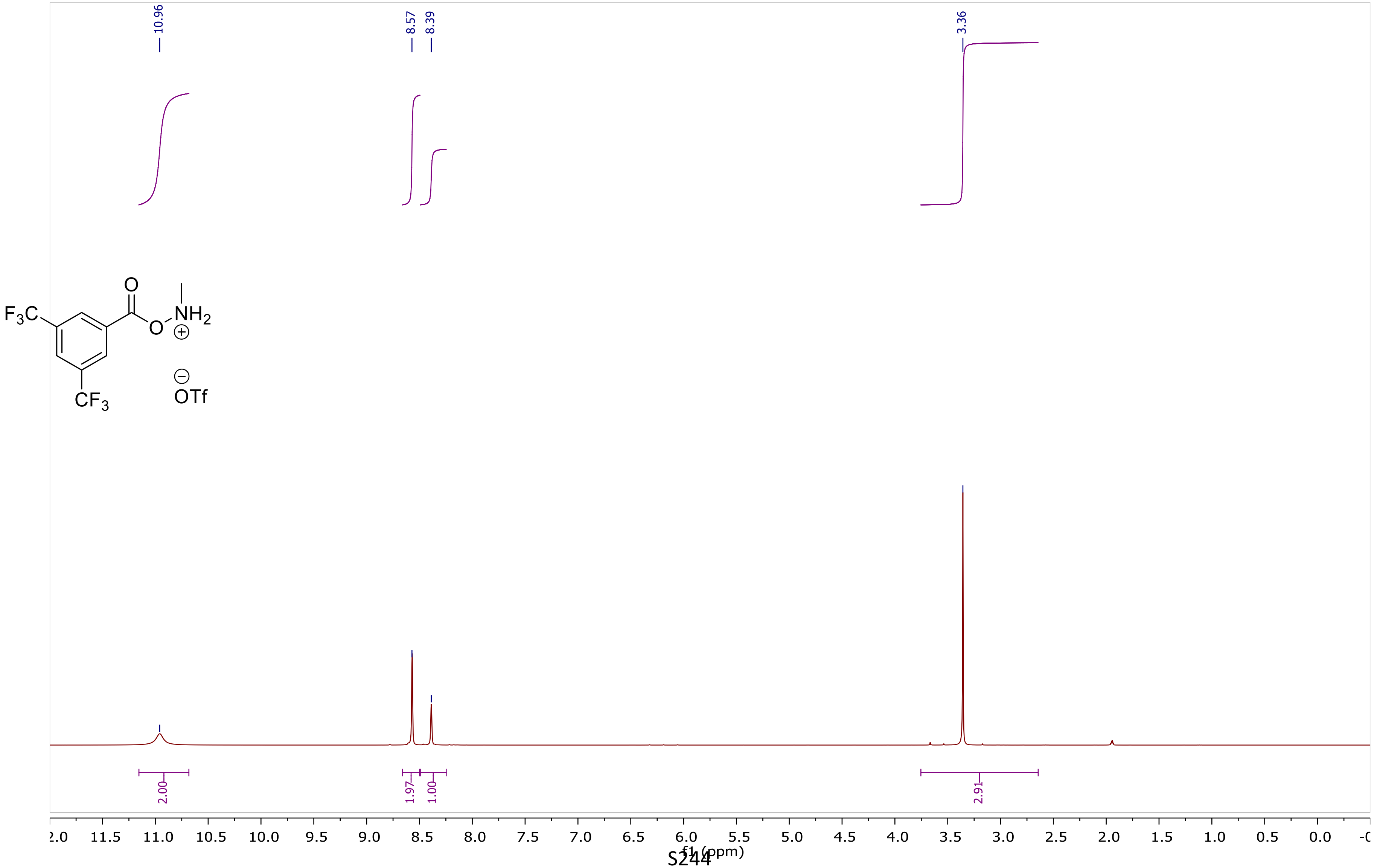

^{13}C NMR of *O*-(3,5-bis(trifluoromethyl)benzoyl)-*N*-methylhydroxylammonium trifluoromethanesulfoate **3c** in CD_3CN

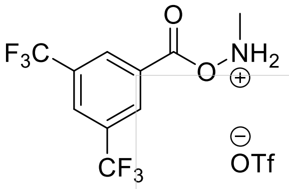

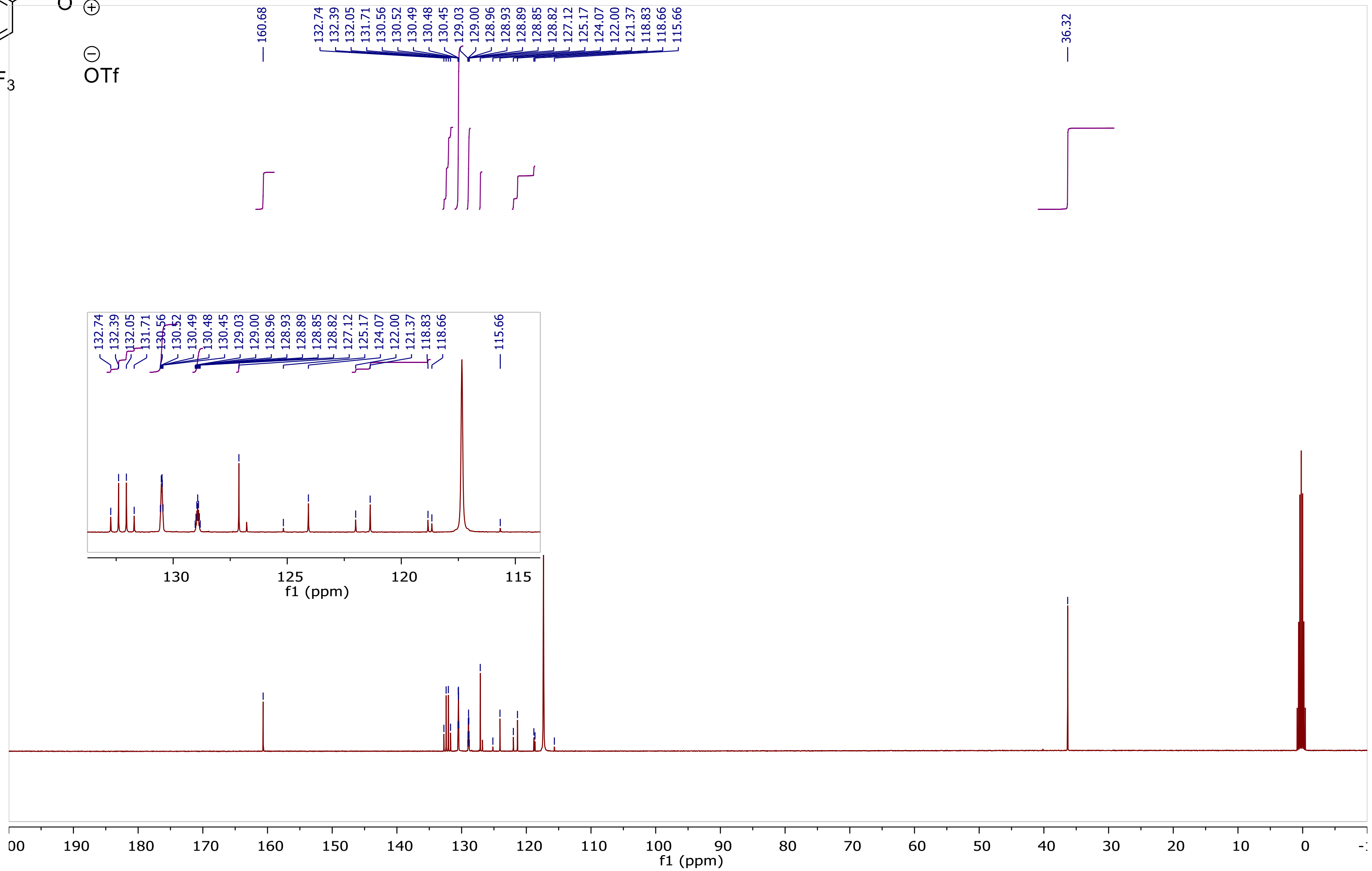

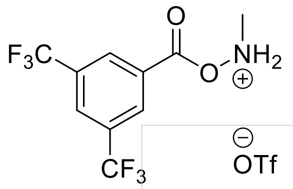

^{19}F NMR of *O*-(3,5-bis(trifluoromethyl)benzoyl)-*N*-methylhydroxylammonium trifluoromethanesulfonate **3c** in CD_3CN

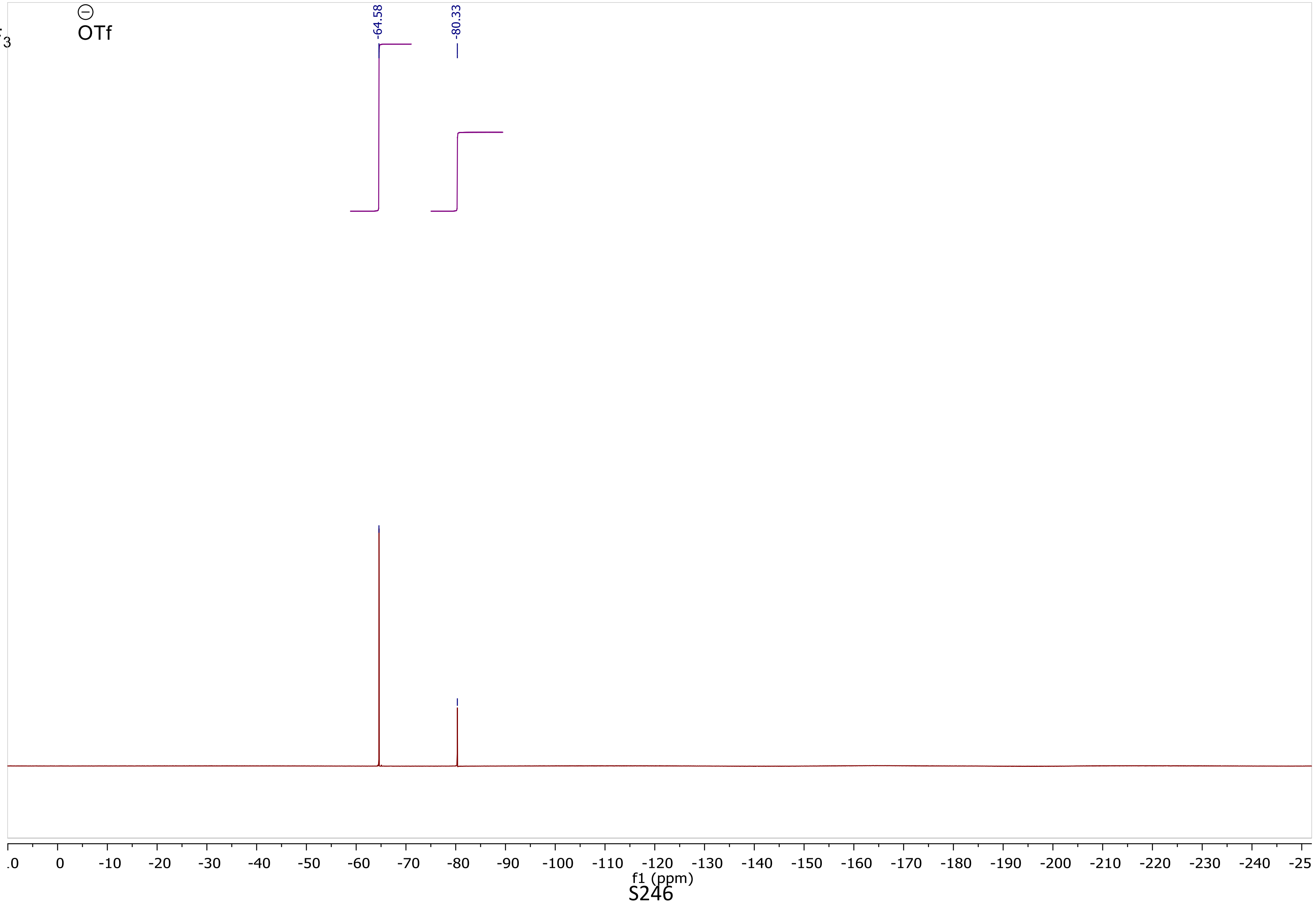

^{1}H NMR of *tert*-butyl (4-methoxybenzoyl)oxy)carbamate in $CDCl_3$

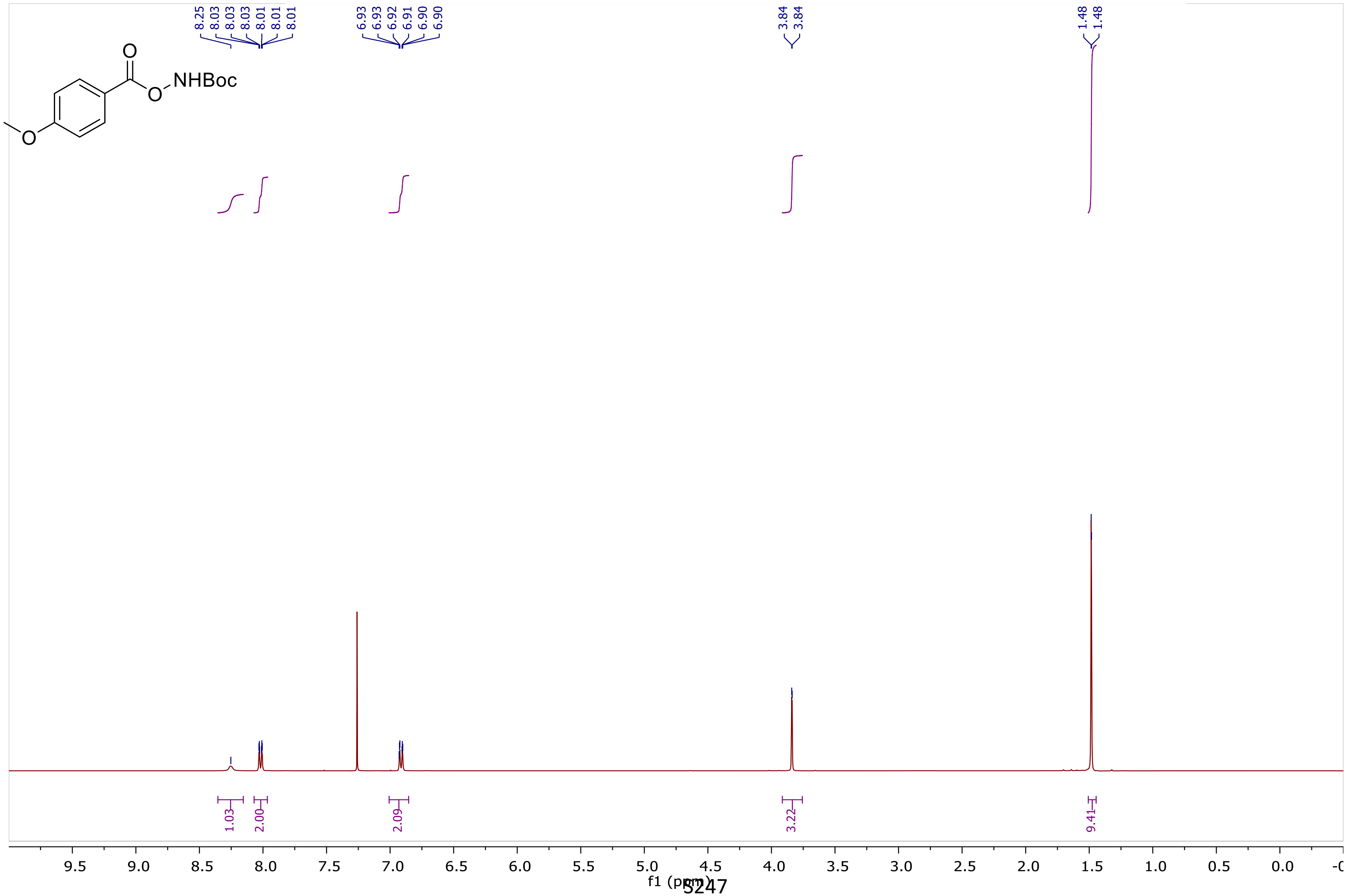

^{13}C NMR of *tert*-butyl (4-methoxybenzoyl)oxy)carbamate in $CDCl_3$

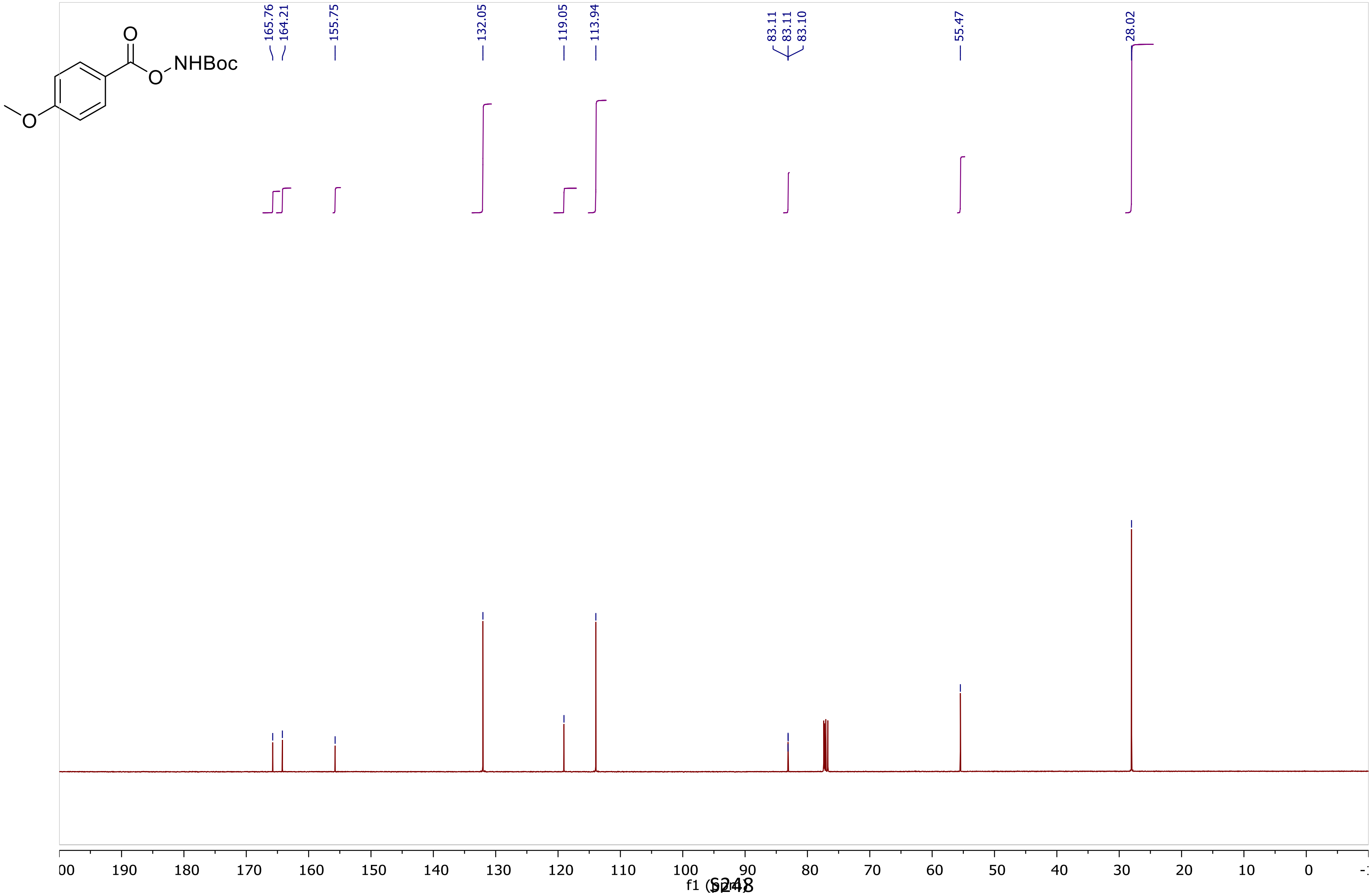

^{1}H NMR of *tert*-butyl ethyl((4-methoxybenzoyl)oxy)carbamate in $CDCl_3$

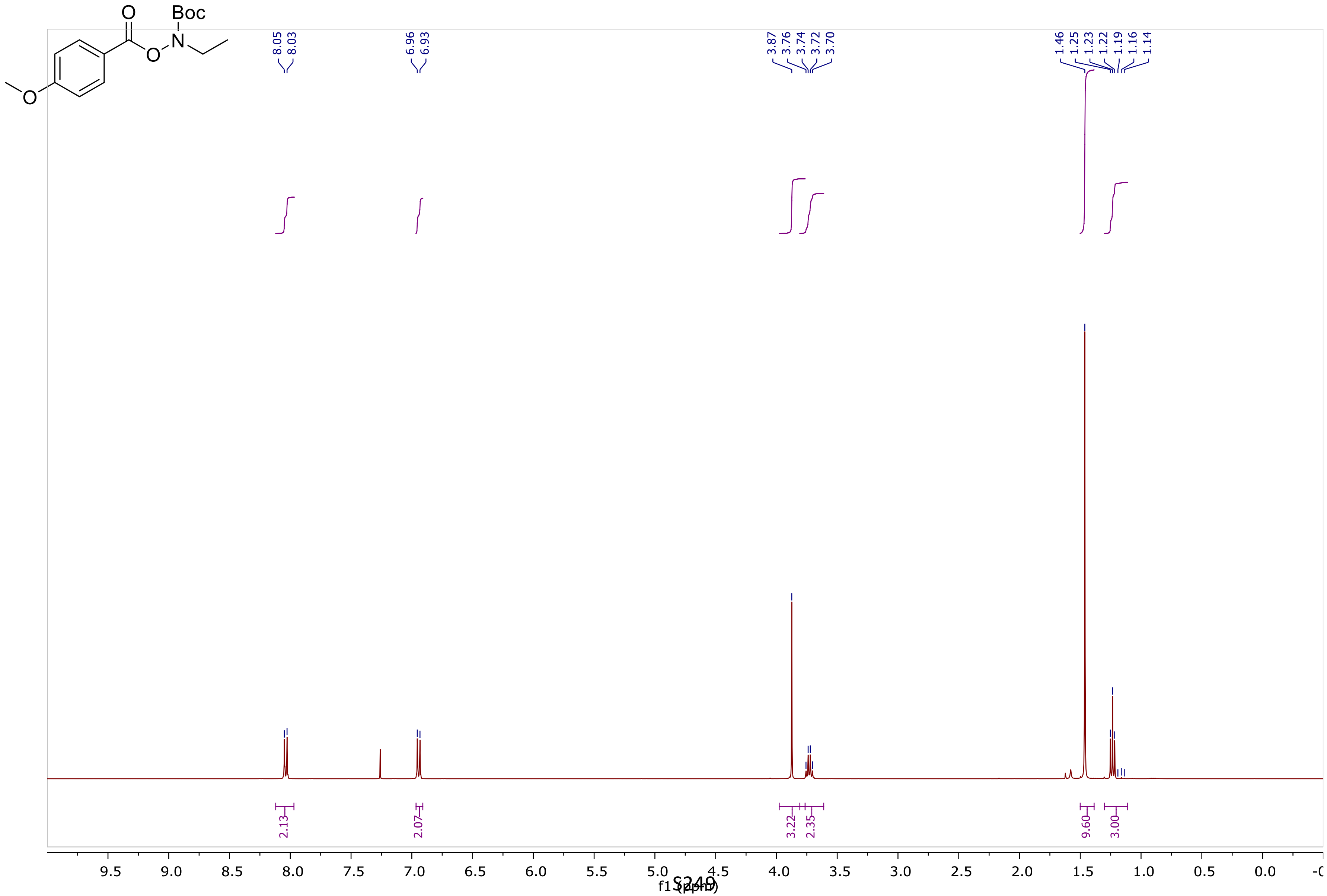

^{13}C NMR of *tert*-butyl ethyl((4-methoxybenzoyl)oxy)carbamate in $CDCl_3$

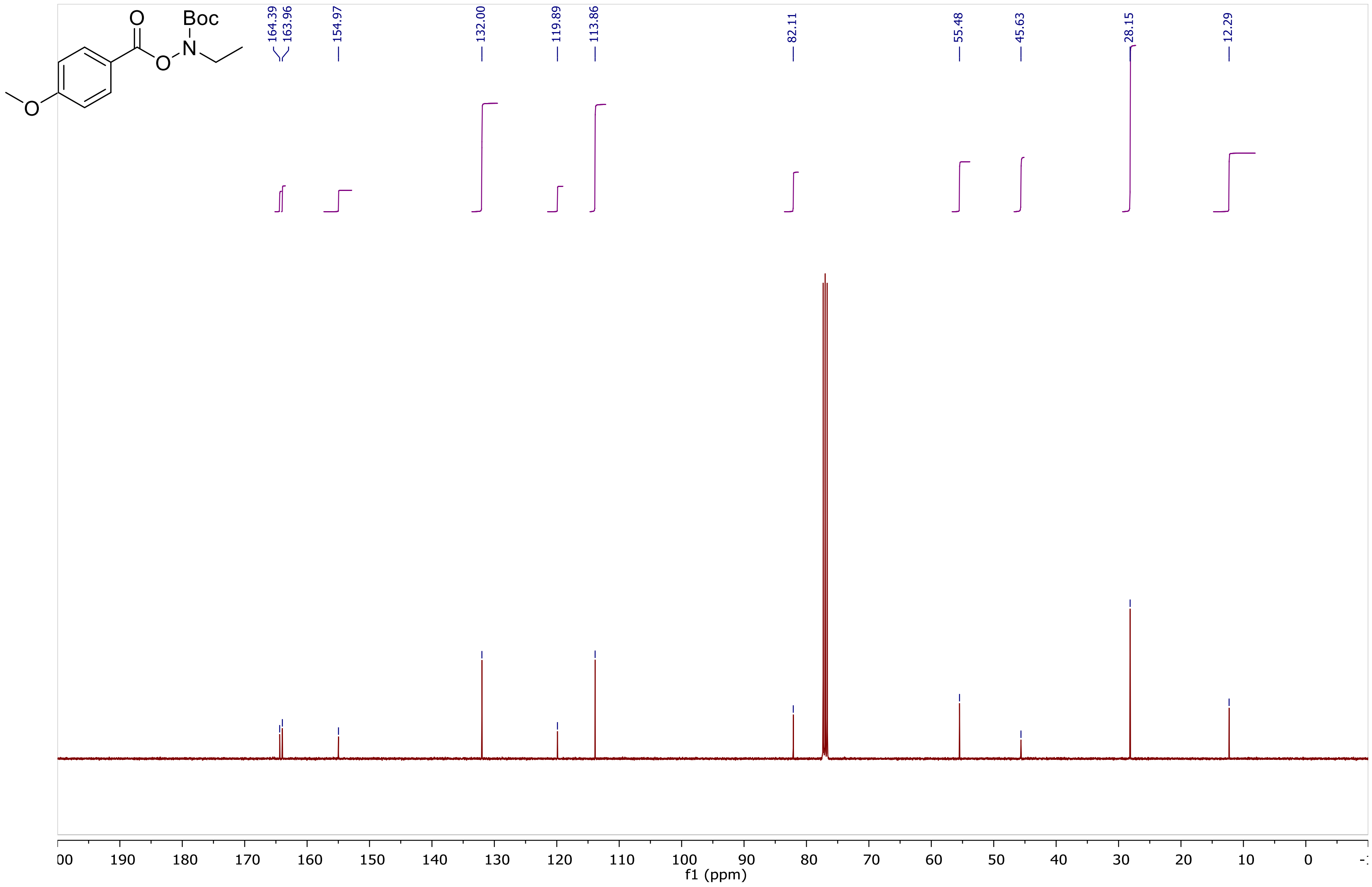

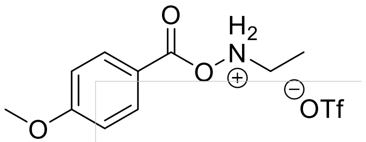

^{1}H NMR of *O*-(4-methoxybenzoyl)-*N*-ethylhydroxylammonium trifluoromethane sulfonate **3e** in CD_3CN

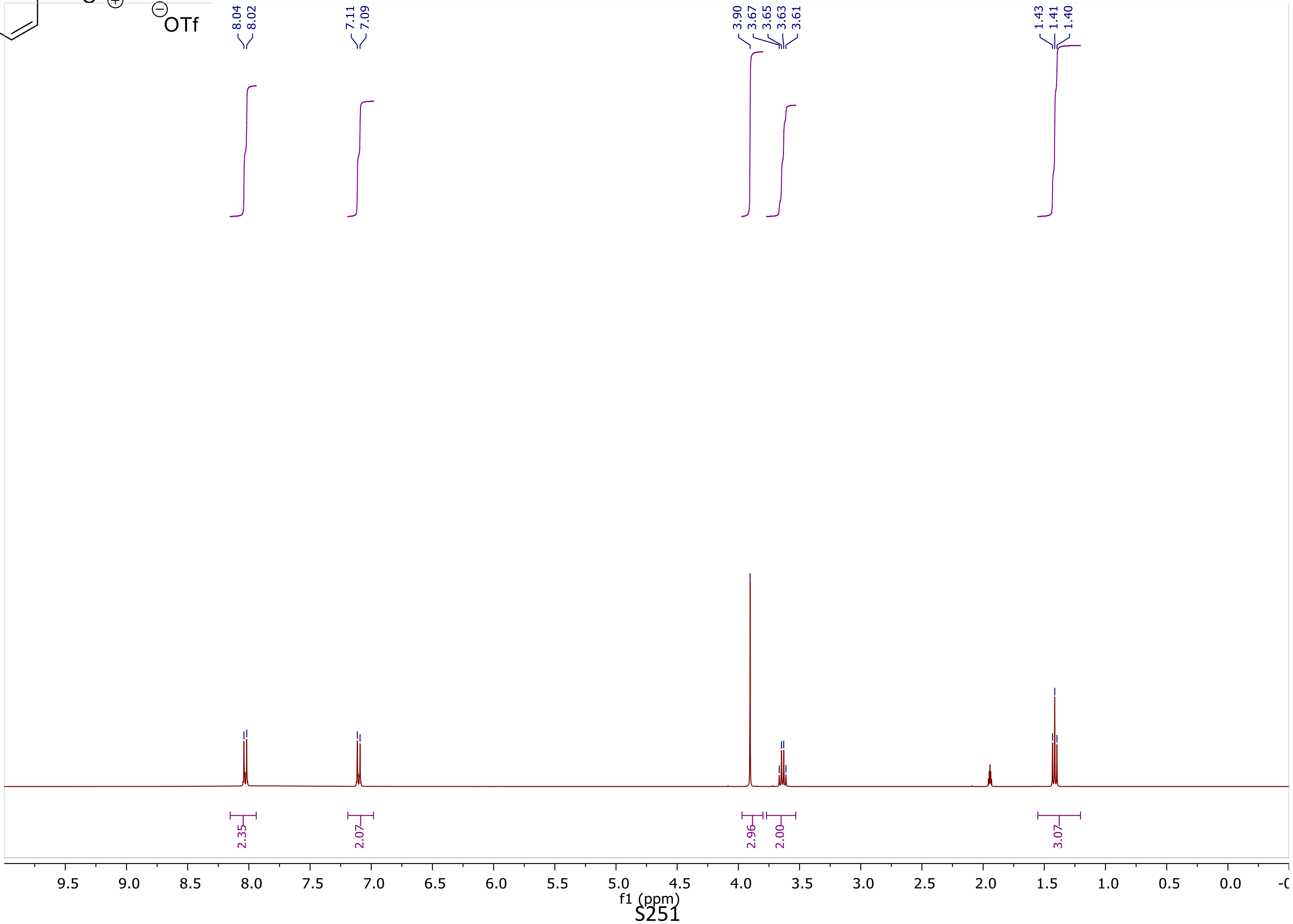

^{13}C NMR of *O*-(4-methoxybenzoyl)-*N*-ethylhydroxylammonium trifluoromethanesulfonate **3e** in CD_3CN

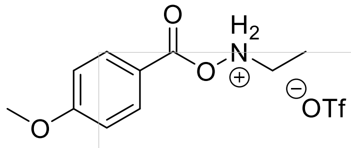

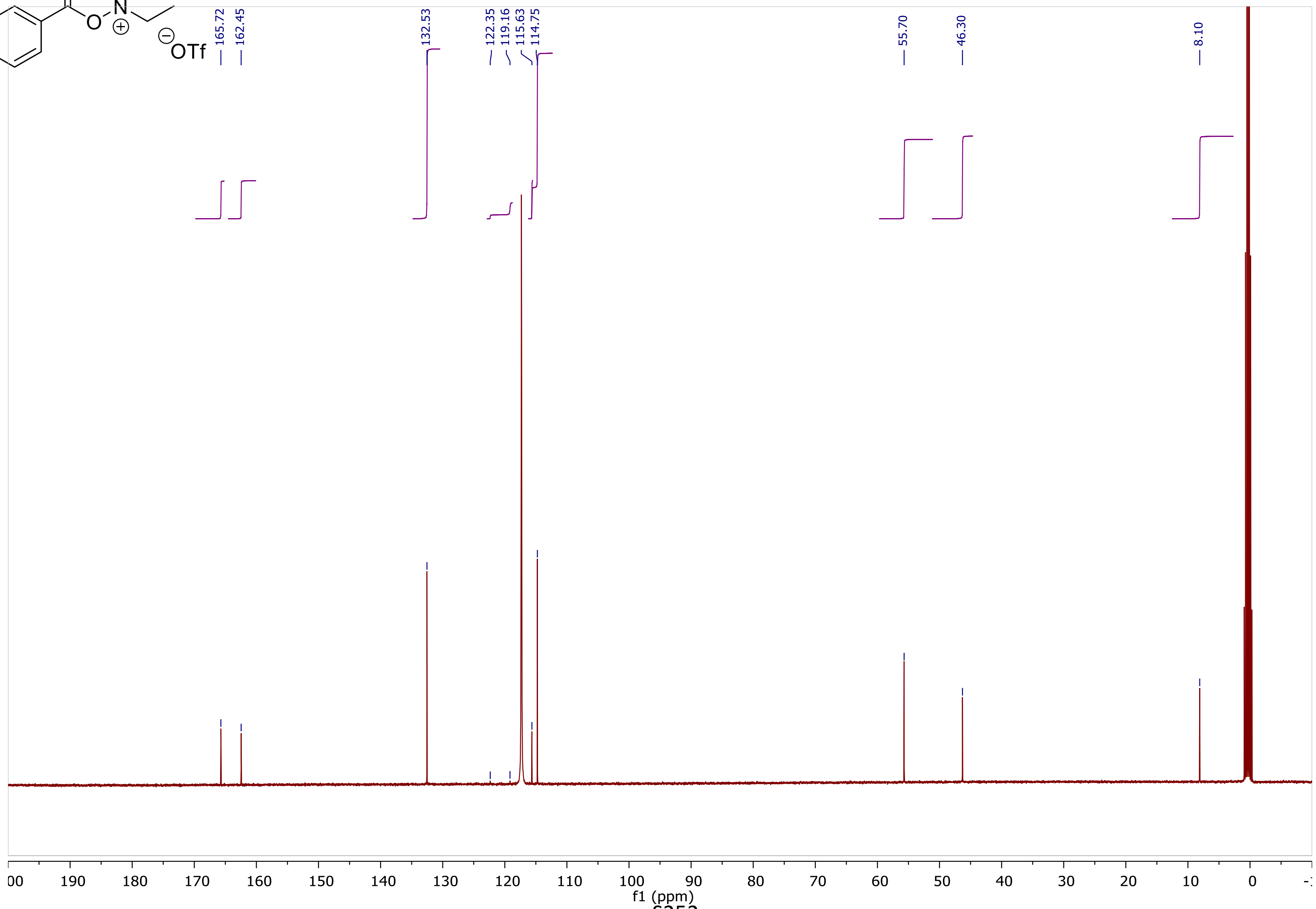

^{19}F NMR of *O*-(4-methoxybenzoyl)-*N*-ethylhydroxylammonium trifluoromethanesulfonate **3e** in CD_3CN

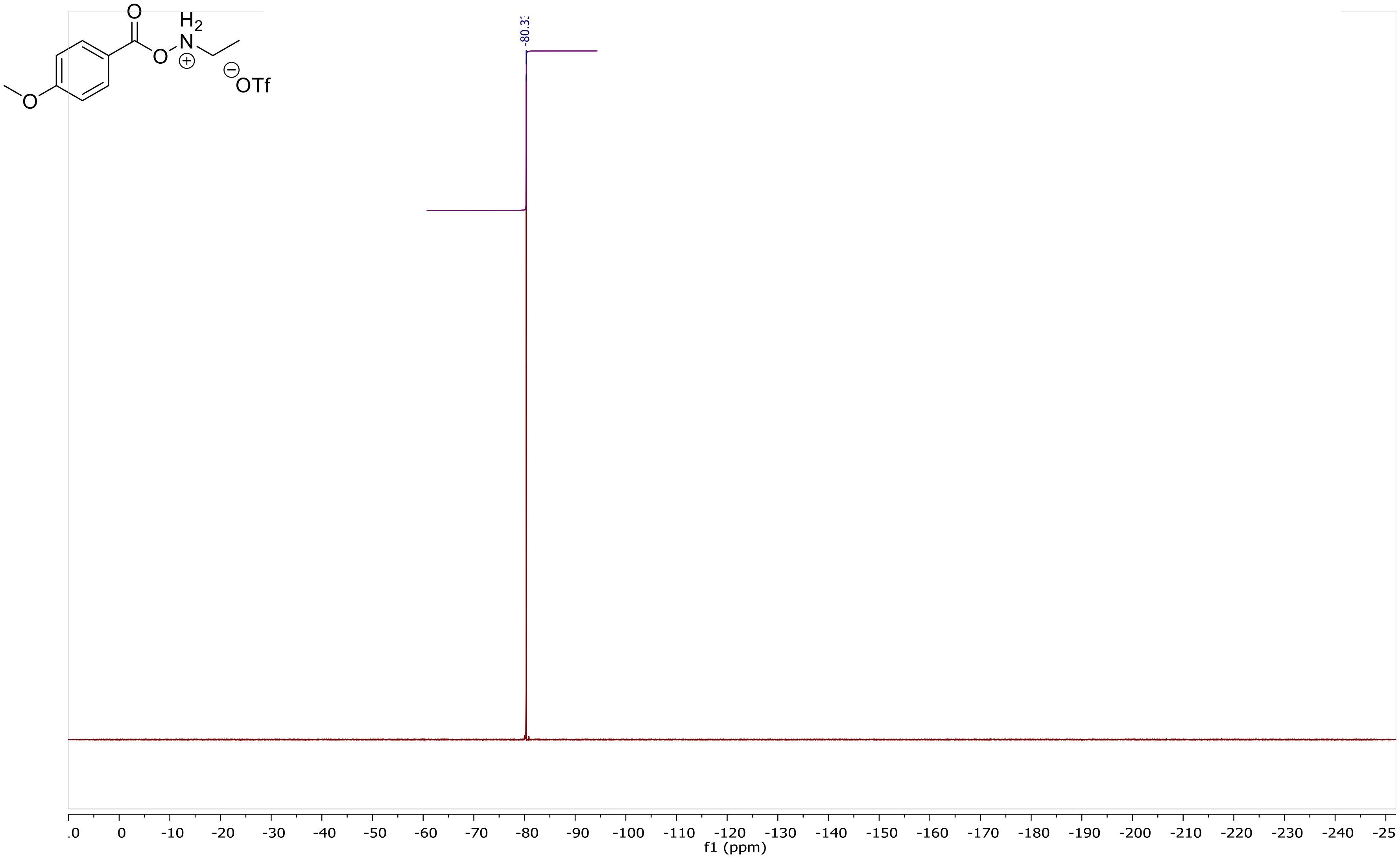

^{1}H NMR of *tert*-butyl propyl((4-methoxybenzoyl)oxy)carbamate in $CDCl_3$

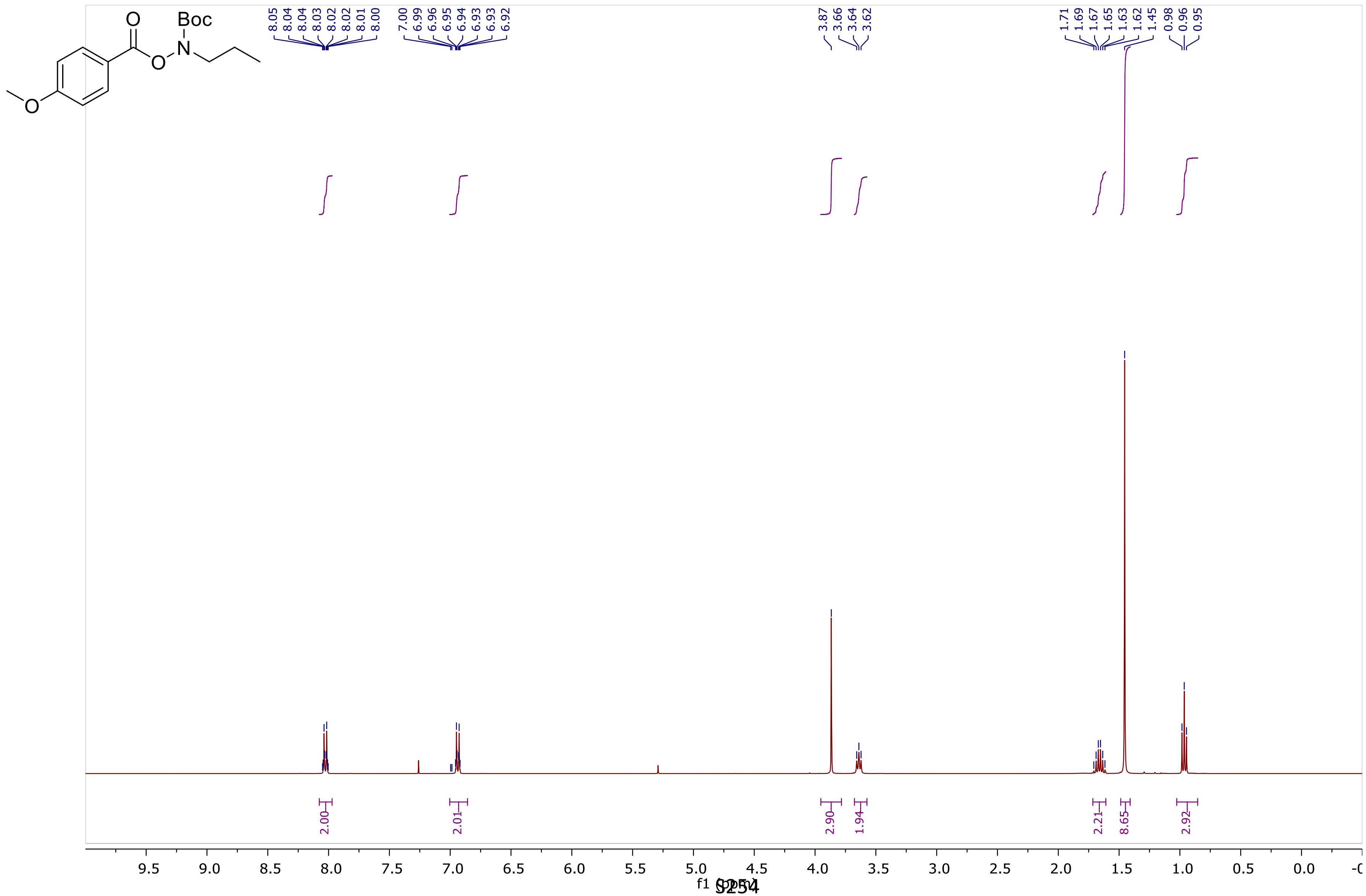

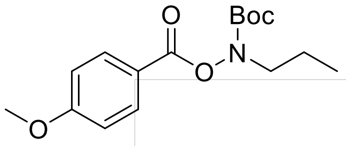

^{13}C NMR of *tert*-butyl propyl((4-methoxybenzoyl)oxy)carbamate in $CDCl_3$

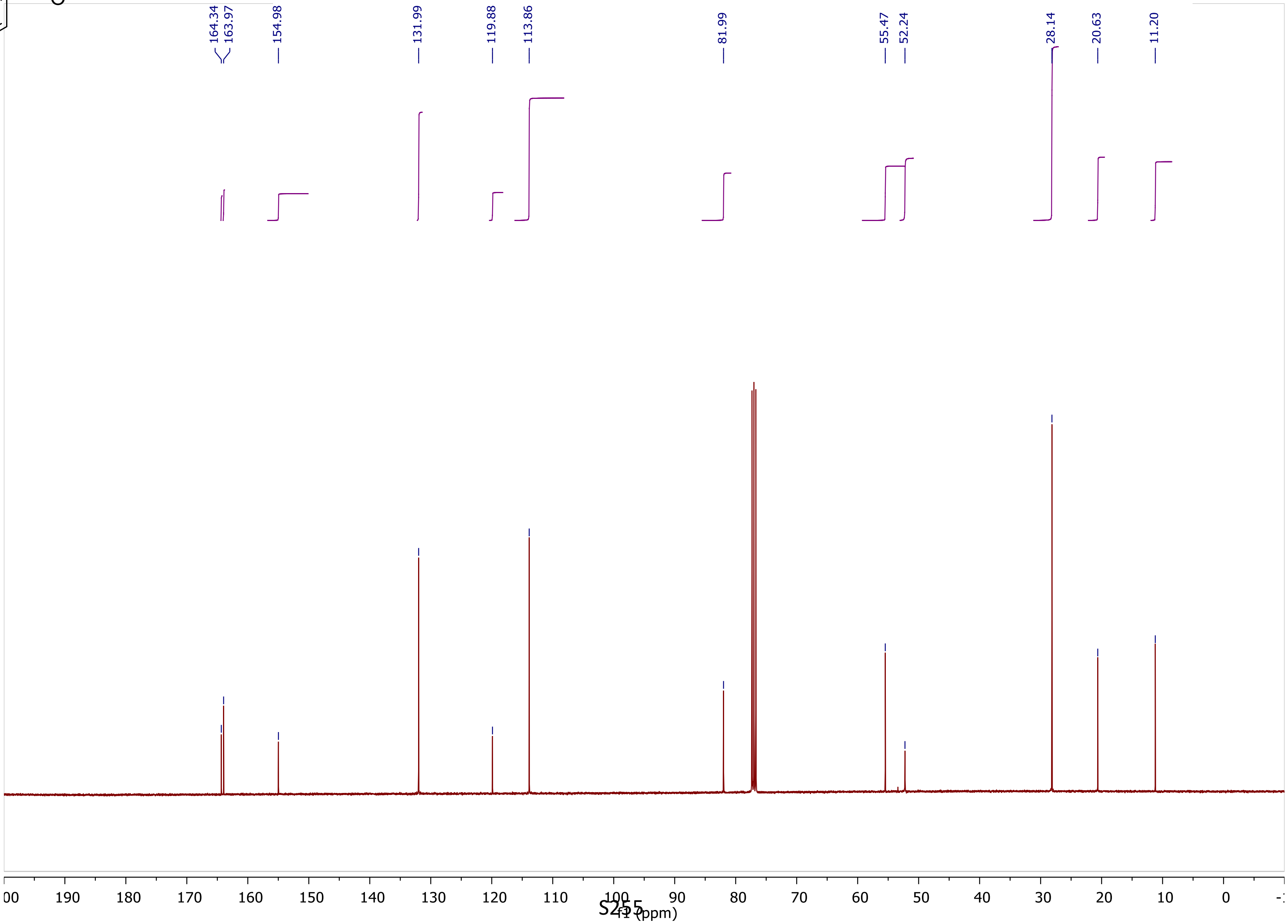

^{1}H NMR of *O*-(4-methoxybenzoyl)-*N*-propylhydroxylammonium trifluoromethanesulfonate **3f** in CD_3CN

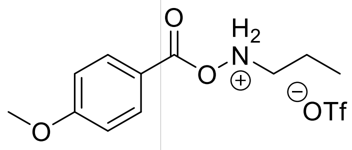

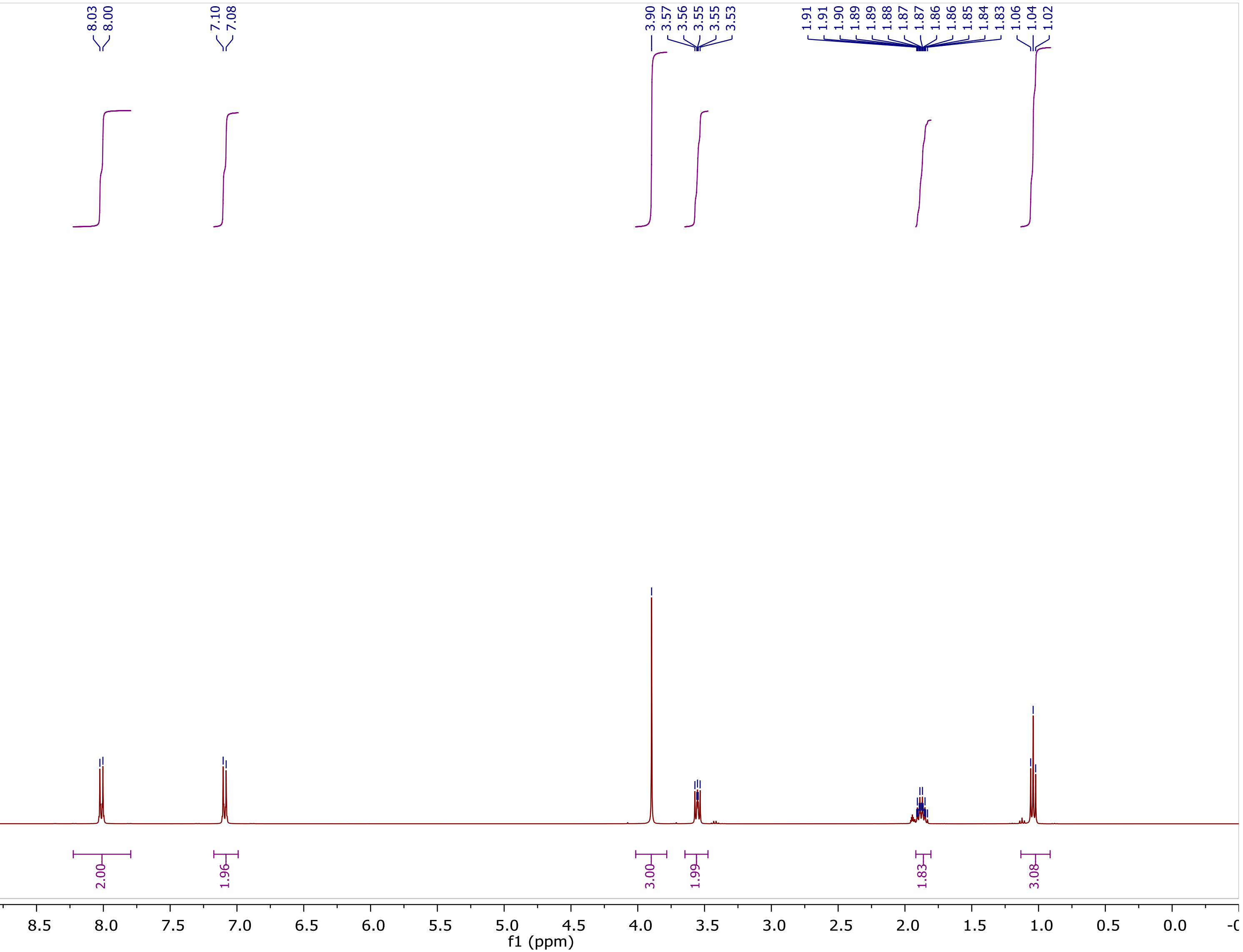

^{13}C NMR of *O*-(4-methoxybenzoyl)-*N*-propylhydroxylammonium trifluoromethanesulfonate **3f** in CD_3CN

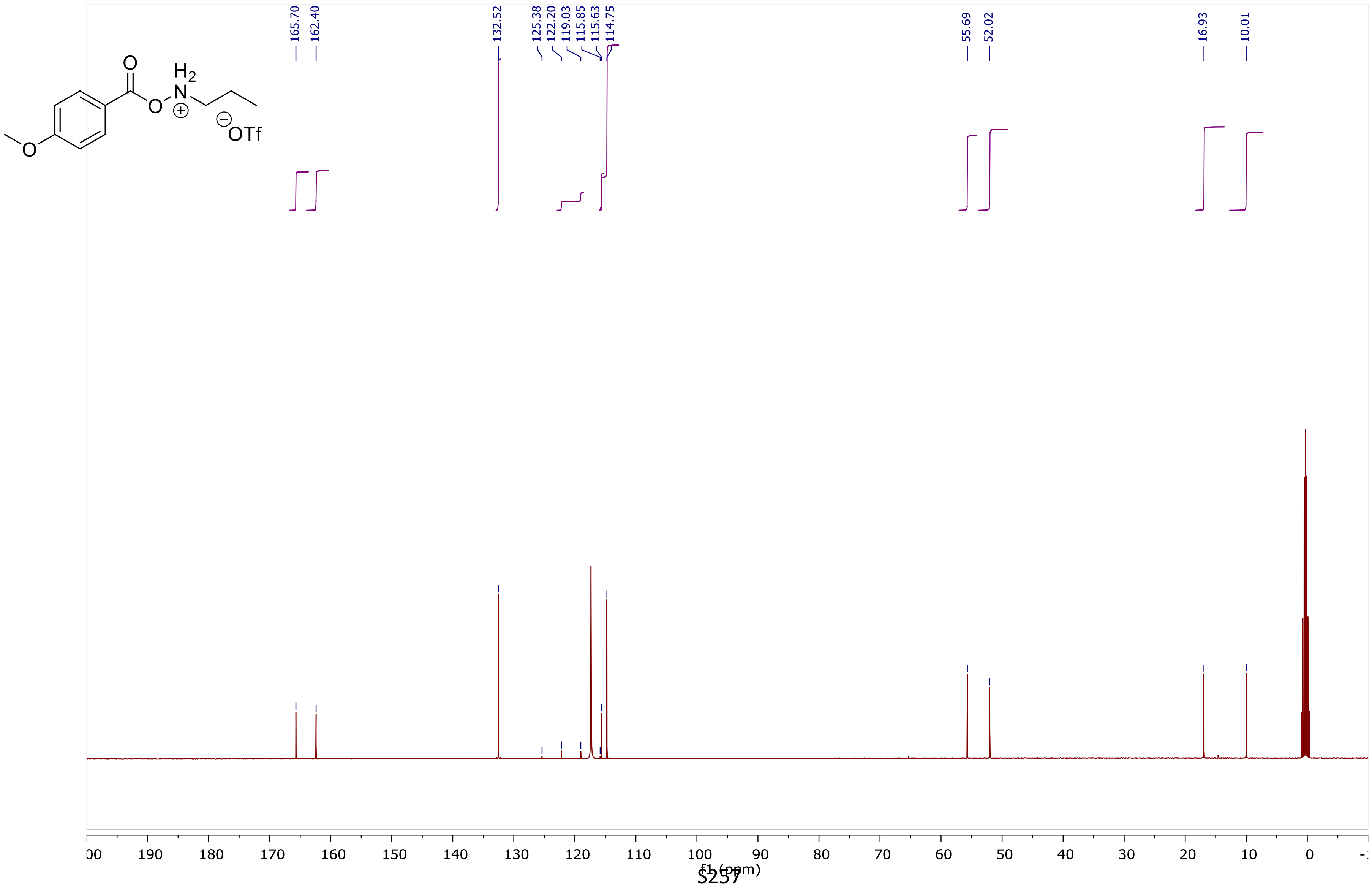

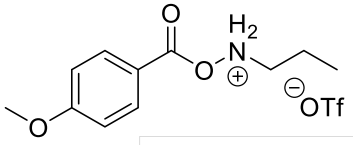

^{19}F NMR of *O*-(4-methoxybenzoyl)-*N*-propylhydroxylammonium trifluoromethanesulfonate **3f** in CD_3CN

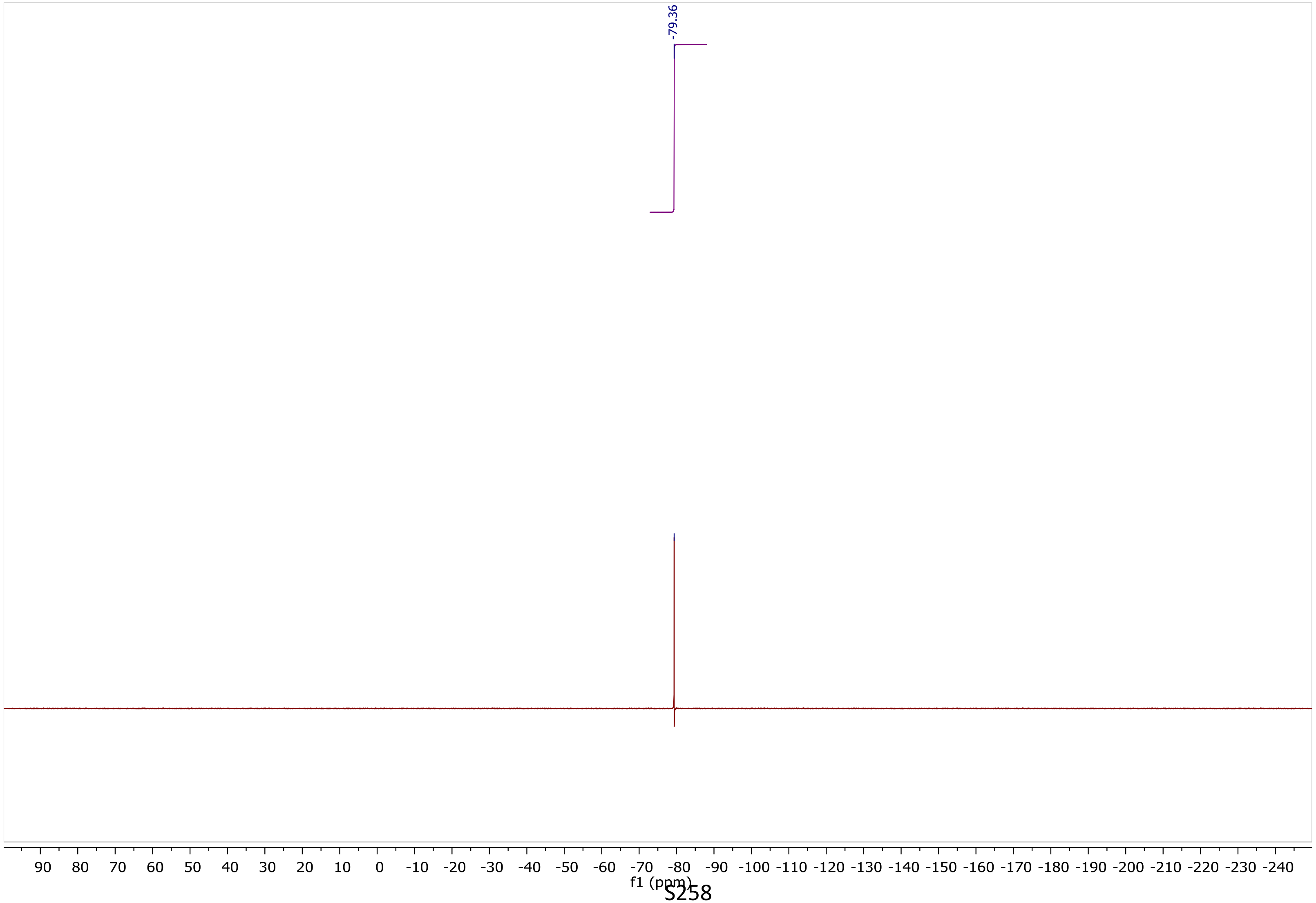

^{1}H NMR of *tert*-butyl hexyl((4-methoxybenzoyl)oxy)carbamate in $CDCl_3$

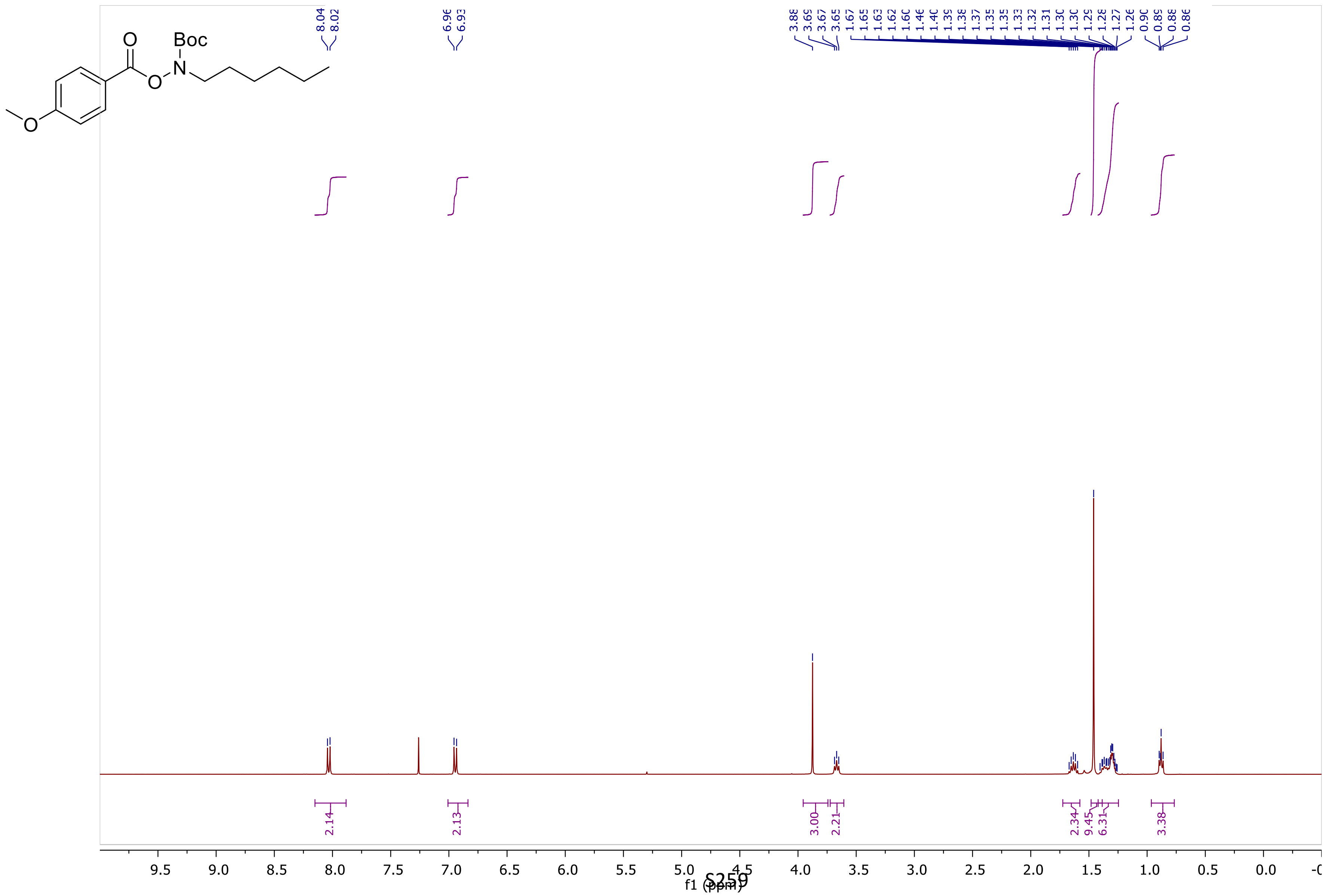

^{13}C NMR of *tert*-butyl hexyl((4-methoxybenzoyl)oxy)carbamate in $CDCl_3$

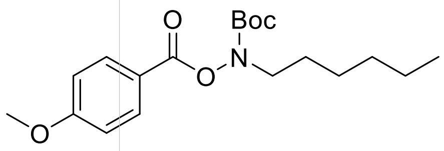

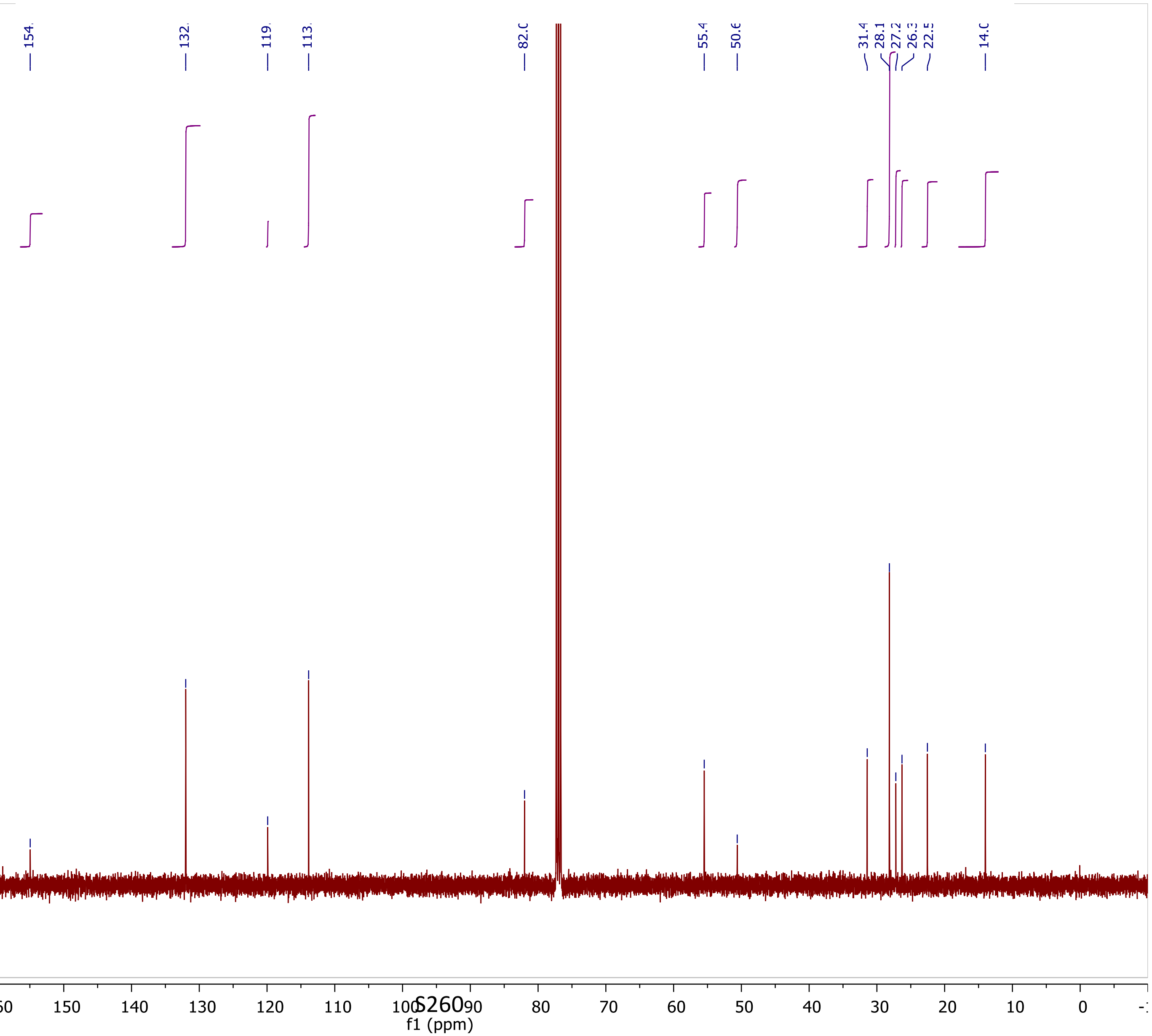

^{1}H NMR of *O*-(4-methoxybenzoyl)-*N*-hexylhydroxylammonium trifluoromethanesulfonate **3h** in CD_3CN

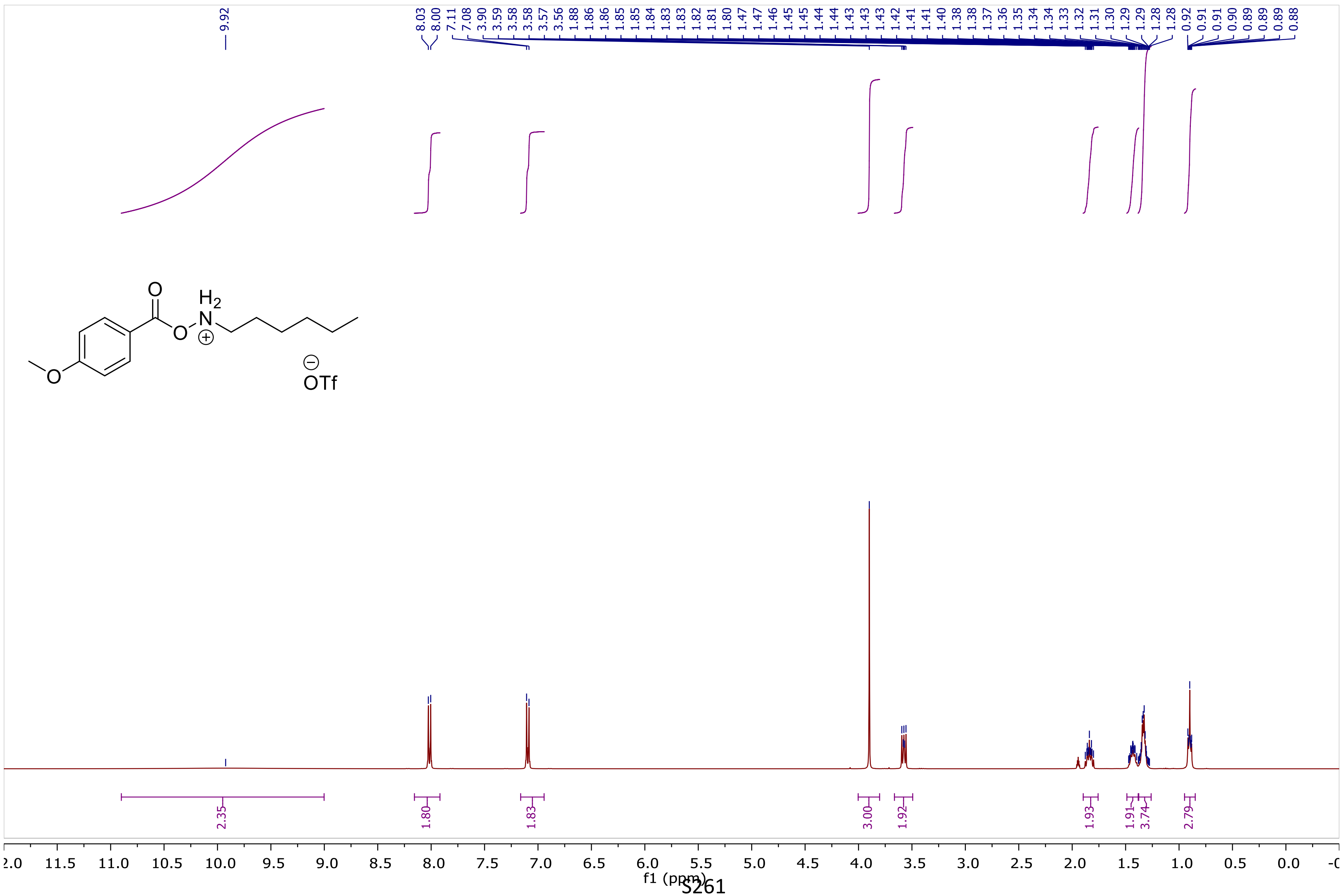

^{13}C NMR of *O*-(4-methoxybenzoyl)-*N*-hexylhydroxylammonium trifluoromethanesulfonate **3h** in CD_3CN

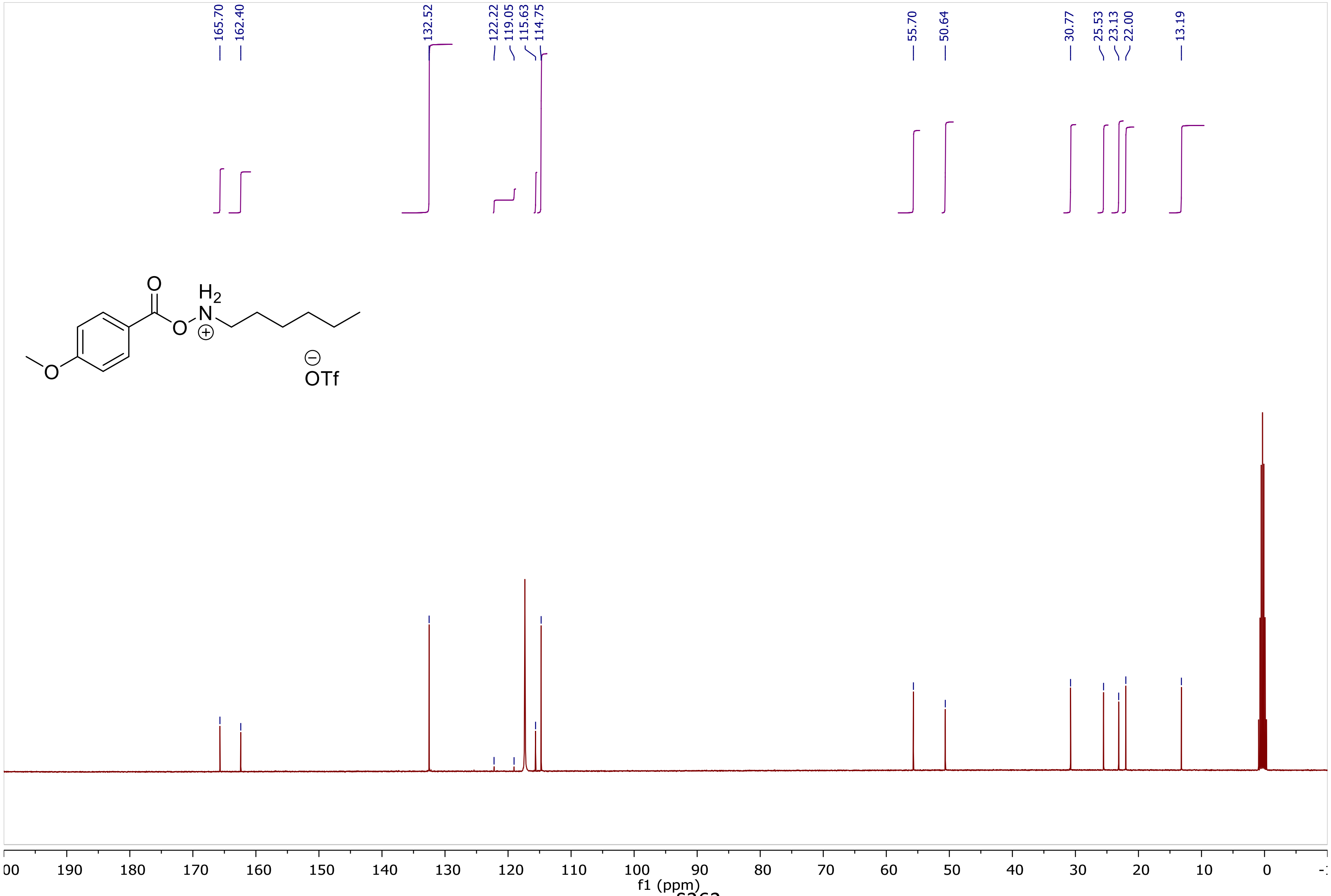

^{19}F NMR of *O*-(4-methoxybenzoyl)-*N*-hexylhydroxylammonium trifluoromethanesulfonate **3h** in CD_3CN

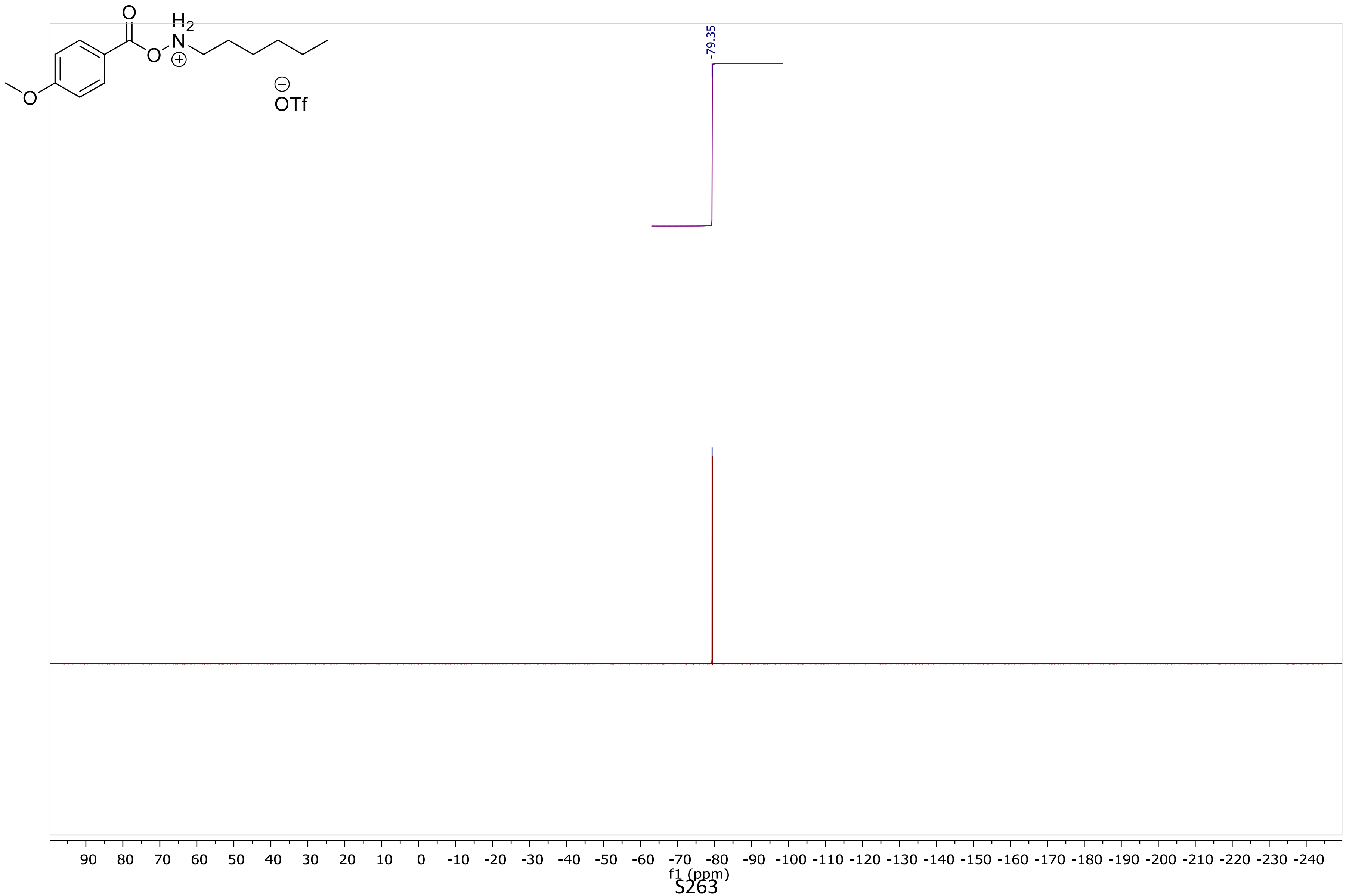

^{1}H NMR of *tert*-butyl (2-cyanoethyl)((4-methoxybenzoyl)oxy)carbamate in $CDCl_3$

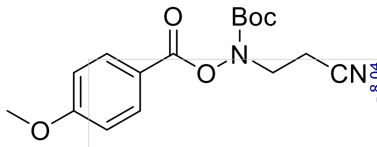

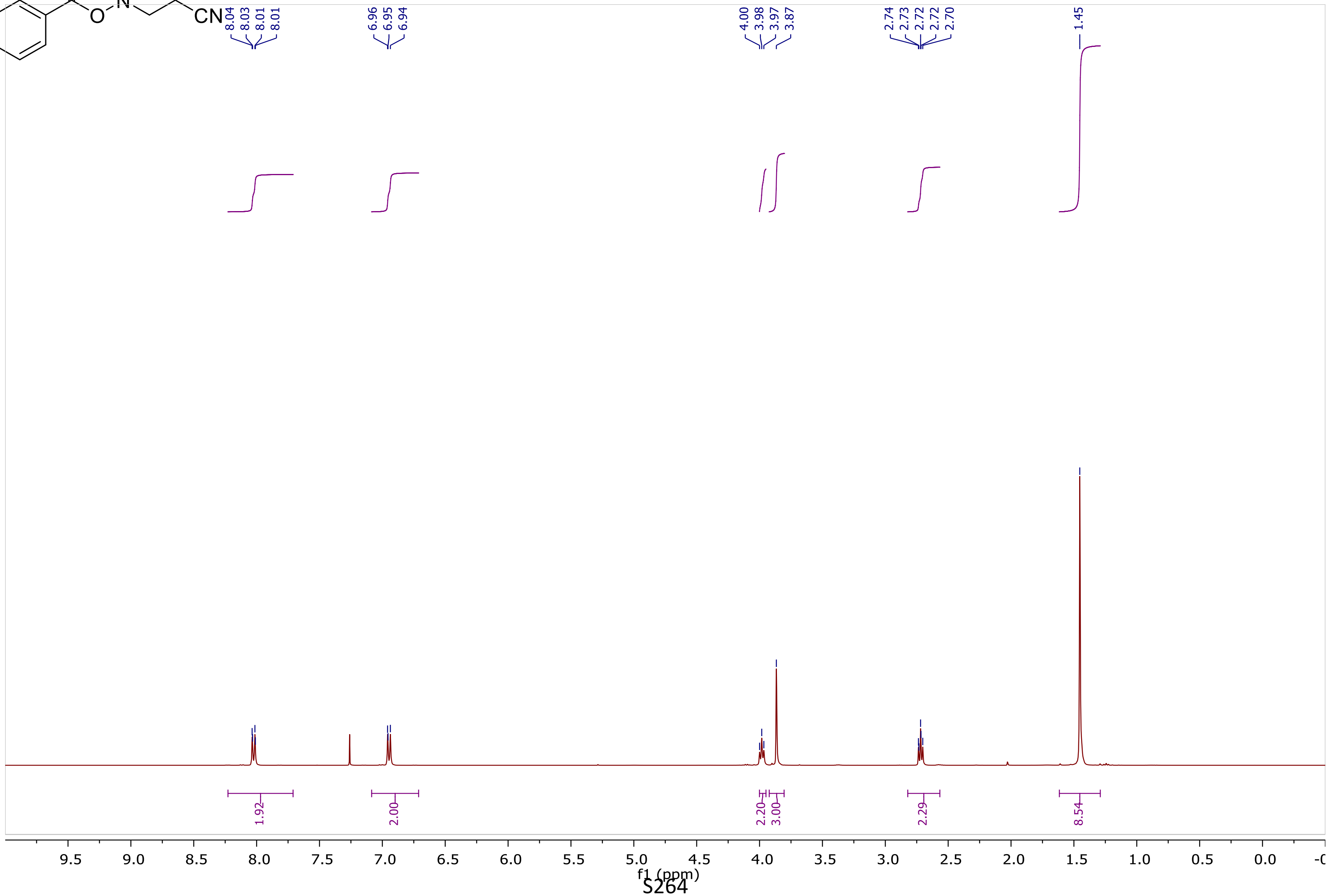

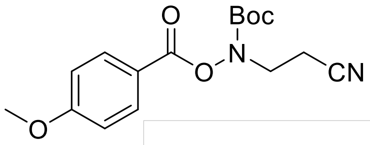

^{13}C NMR of *tert*-butyl (2-cyanoethyl)((4-methoxybenzoyl)oxy)carbamate in $CDCl_3$

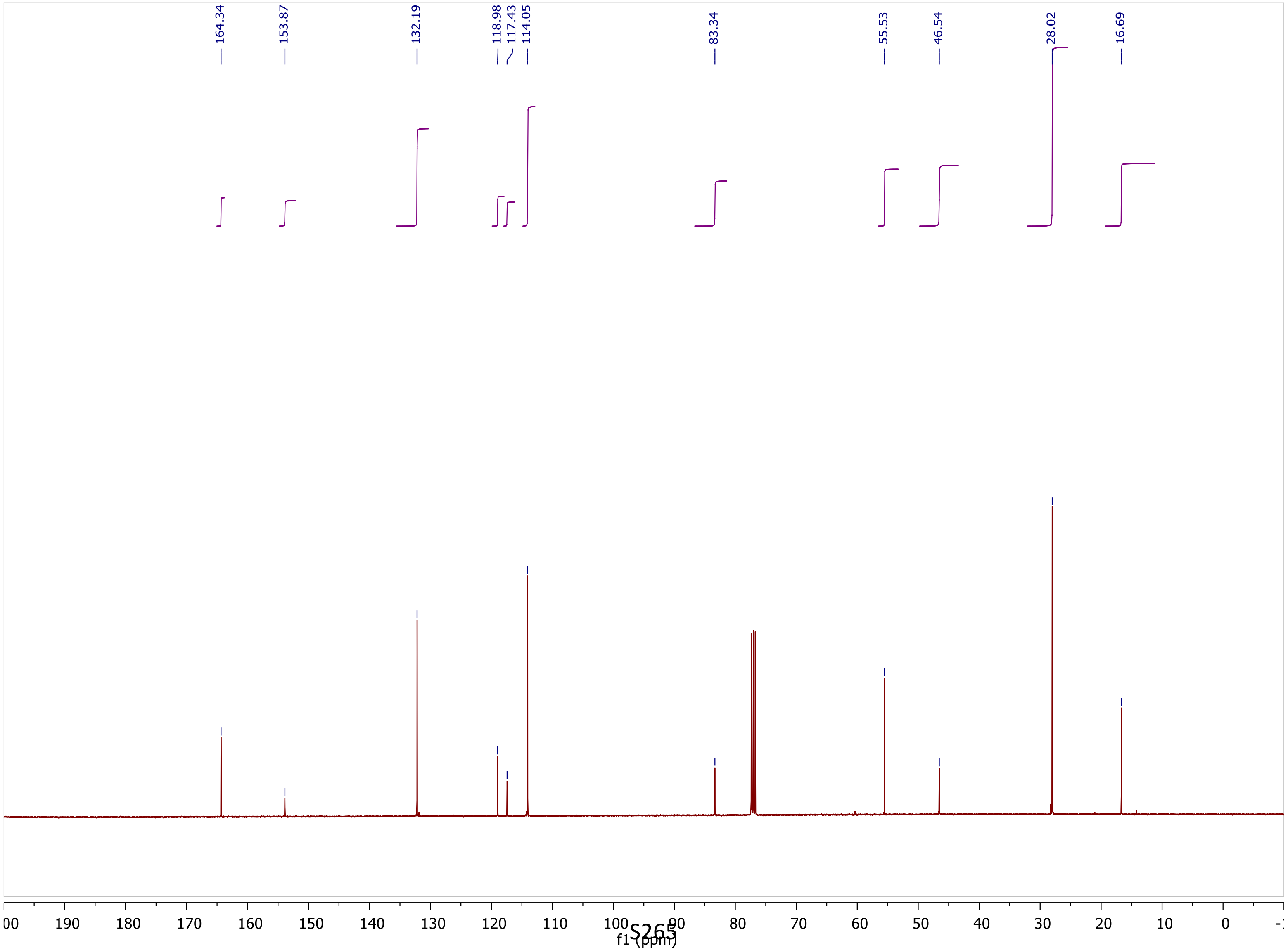

^{1}H NMR of *N*-(2-cyanoethyl)-*O*-(4-methoxybenzoyl)hydroxylammonium trifluoromethanesulfonate **3g** in CD_3CN

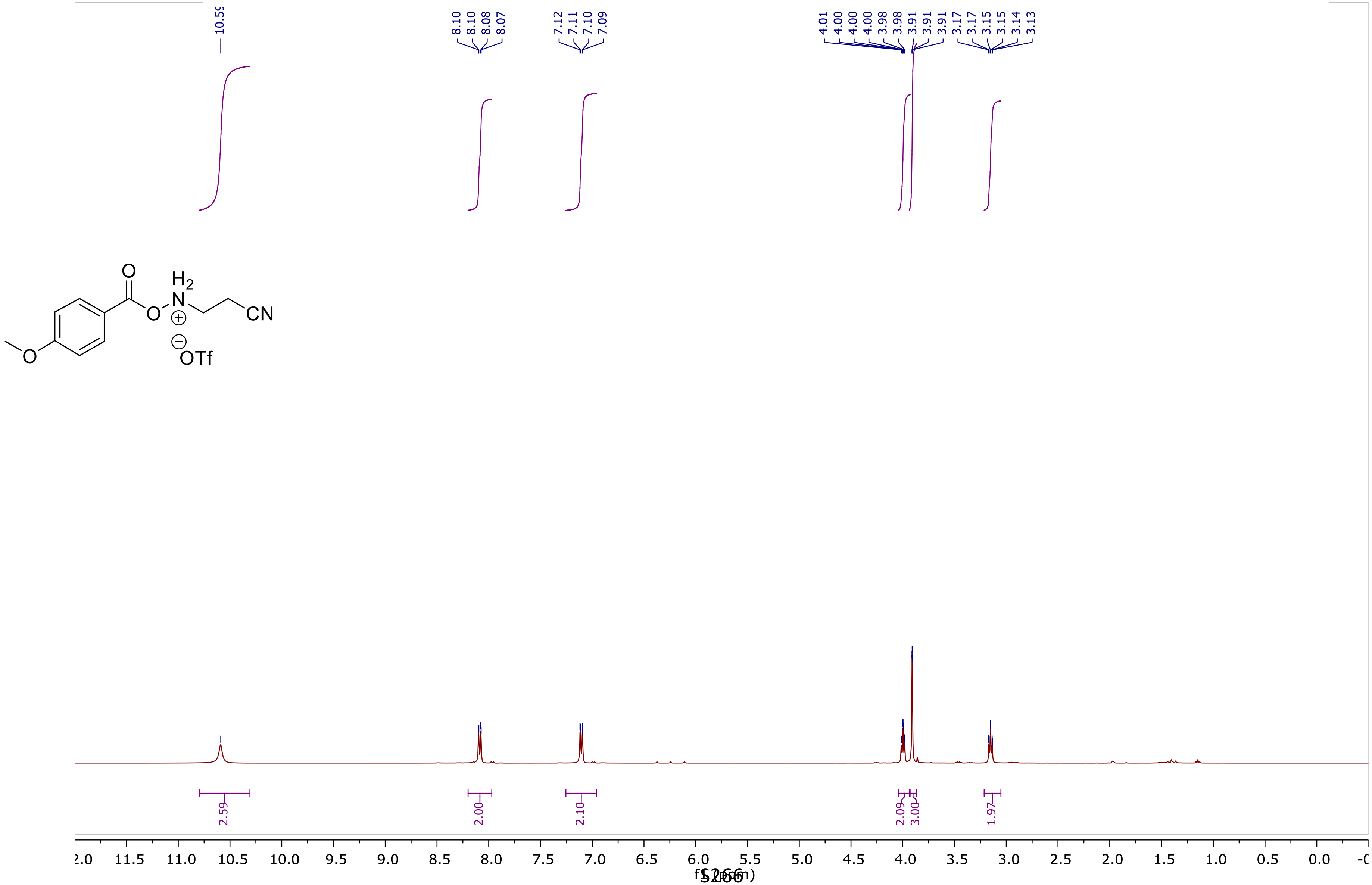

^{13}C NMR of *N*-(2-cyanoethyl)-*O*-(4-methoxybenzoyl)hydroxylammonium trifluoromethanesulfonate **3g** in CD_3CN

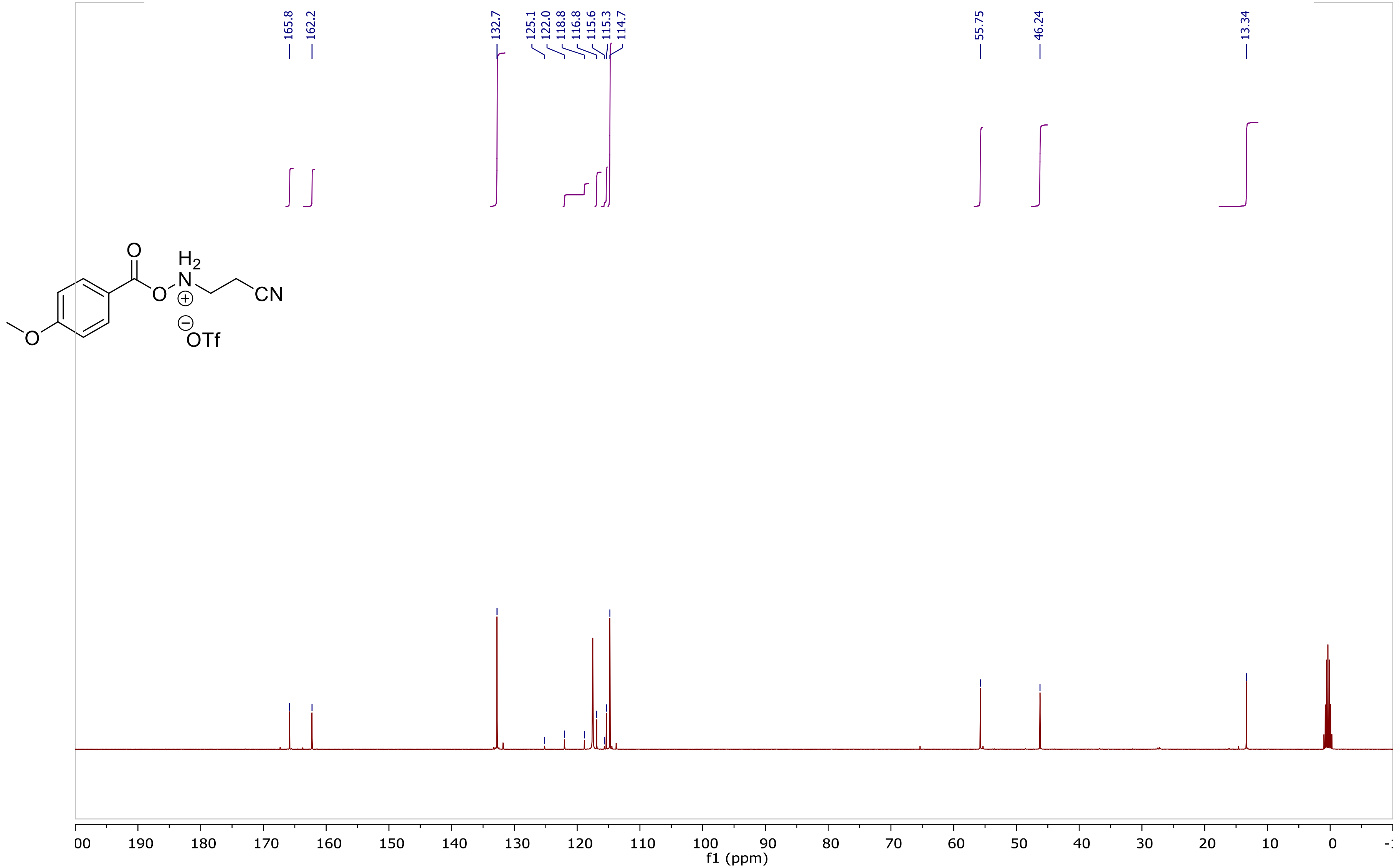

^{19}F NMR of *N*-(2-cyanoethyl)-*O*-(4-methoxybenzoyl)hydroxylammonium trifluoromethanesulfonate **3g** in CD_3CN

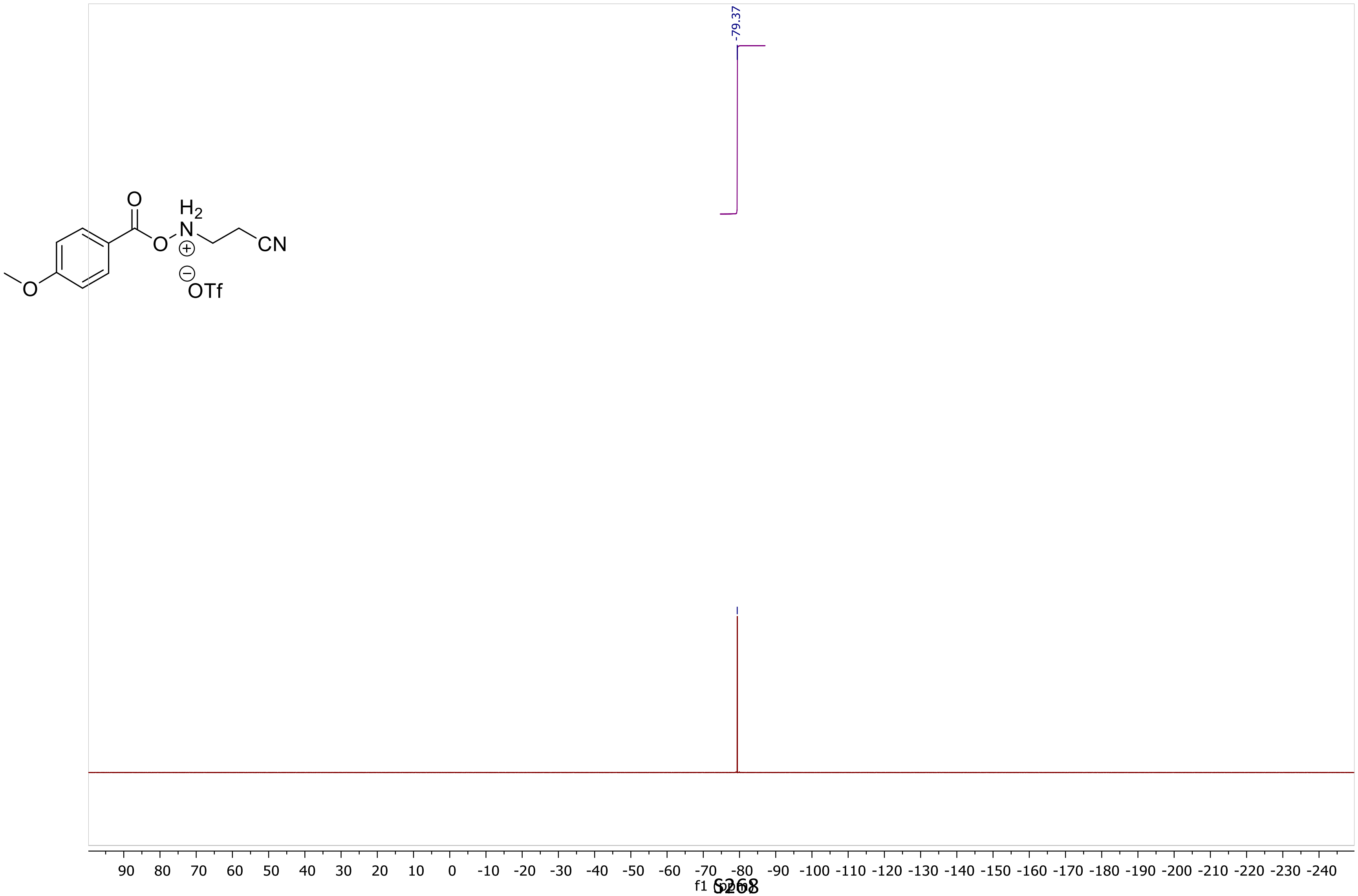

NHMe Transfer Products

^{1}H NMR of *N*1-methylbenzene-1,2-diamine **5a** in $CDCl_3$

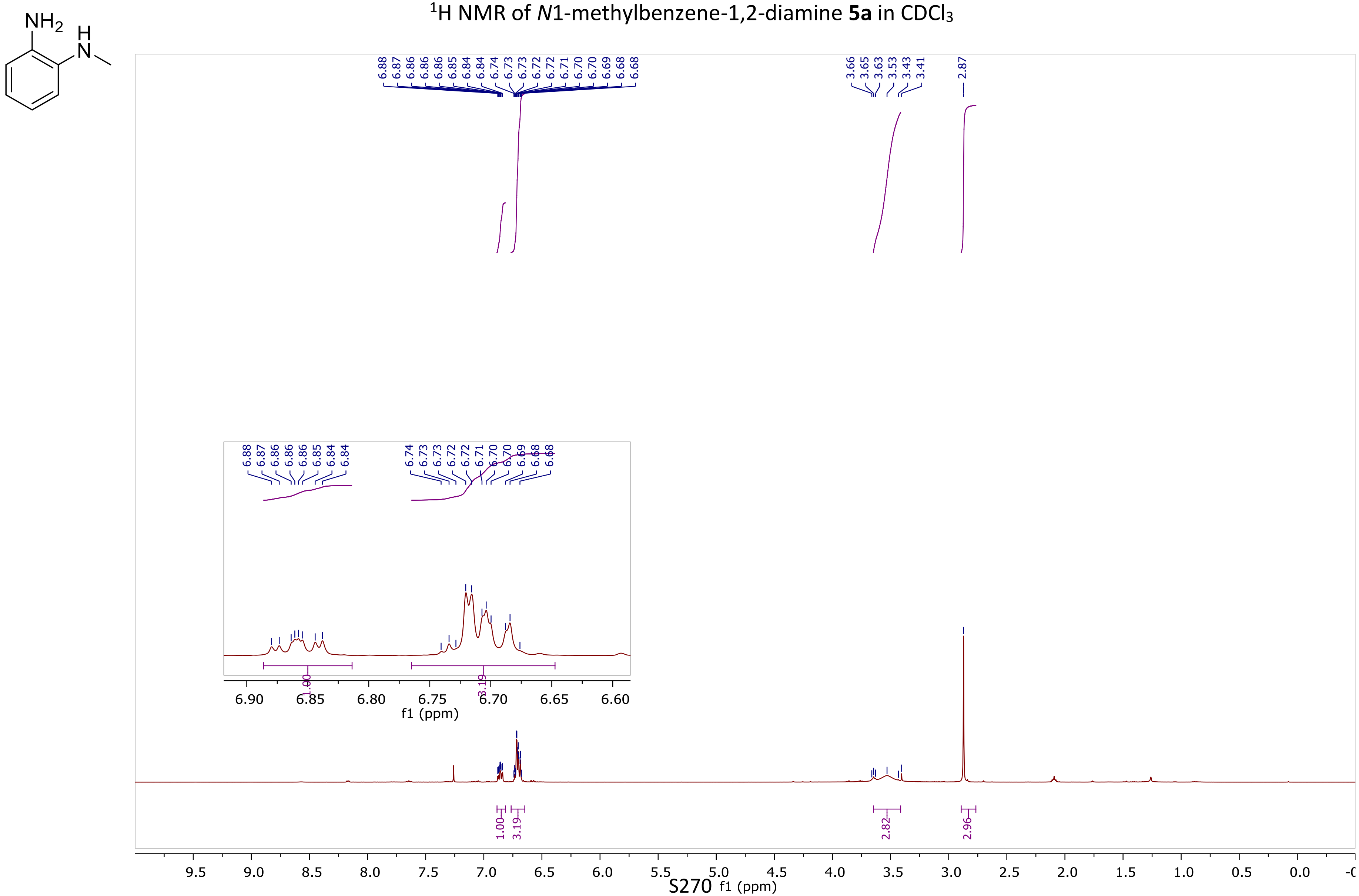

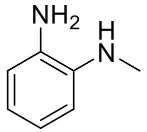

^{13}C NMR of *N*1-methylbenzene-1,2-diamine **5a** in $CDCl_3$

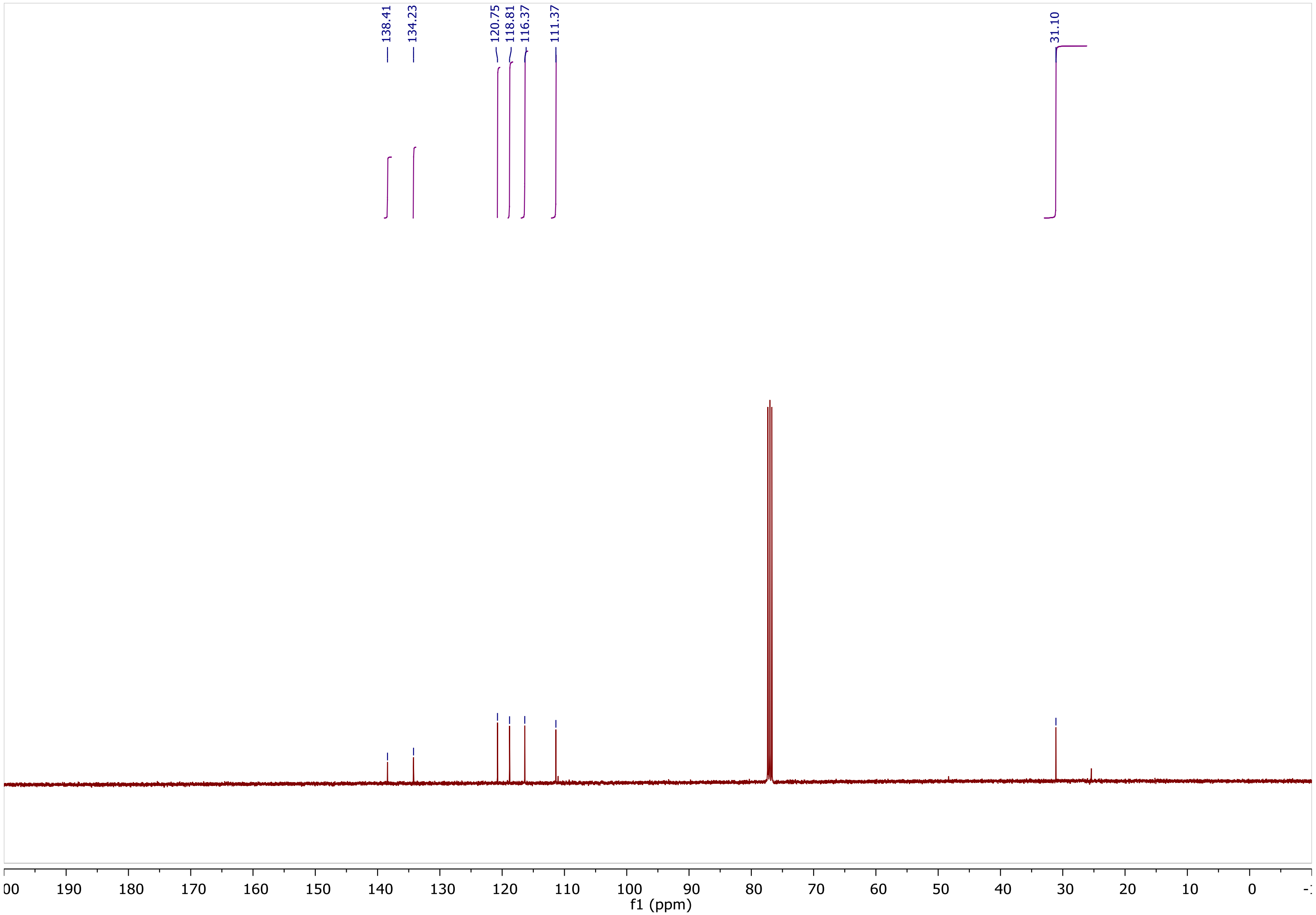

^{1}H NMR of *N*1,3-dimethylbenzene-1,2-diamine **5b** in $CDCl_3$

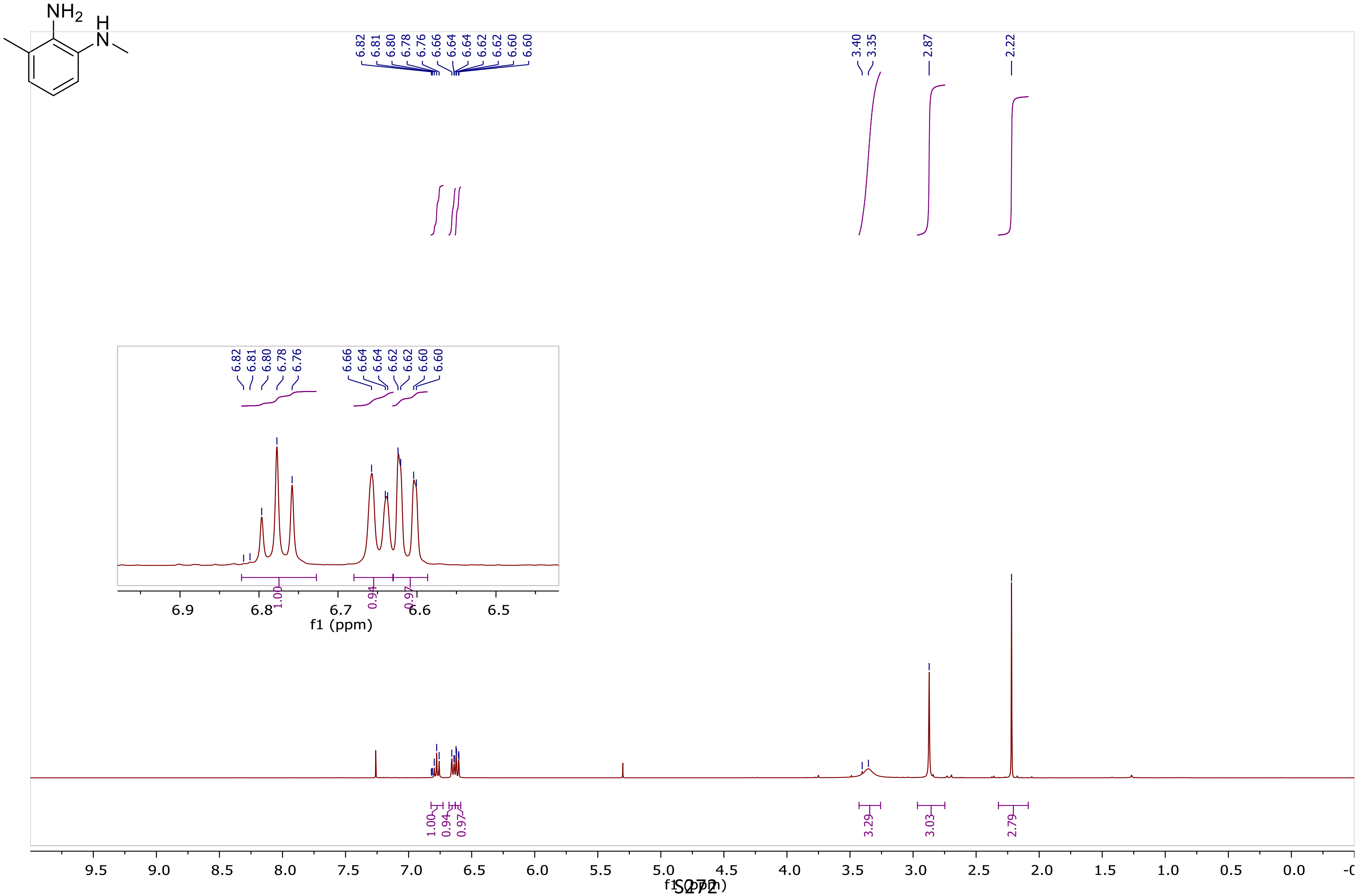

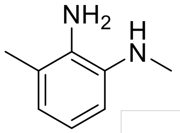

^{13}C NMR of *N*1,3-dimethylbenzene-1,2-diamine **5b** in $CDCl_3$

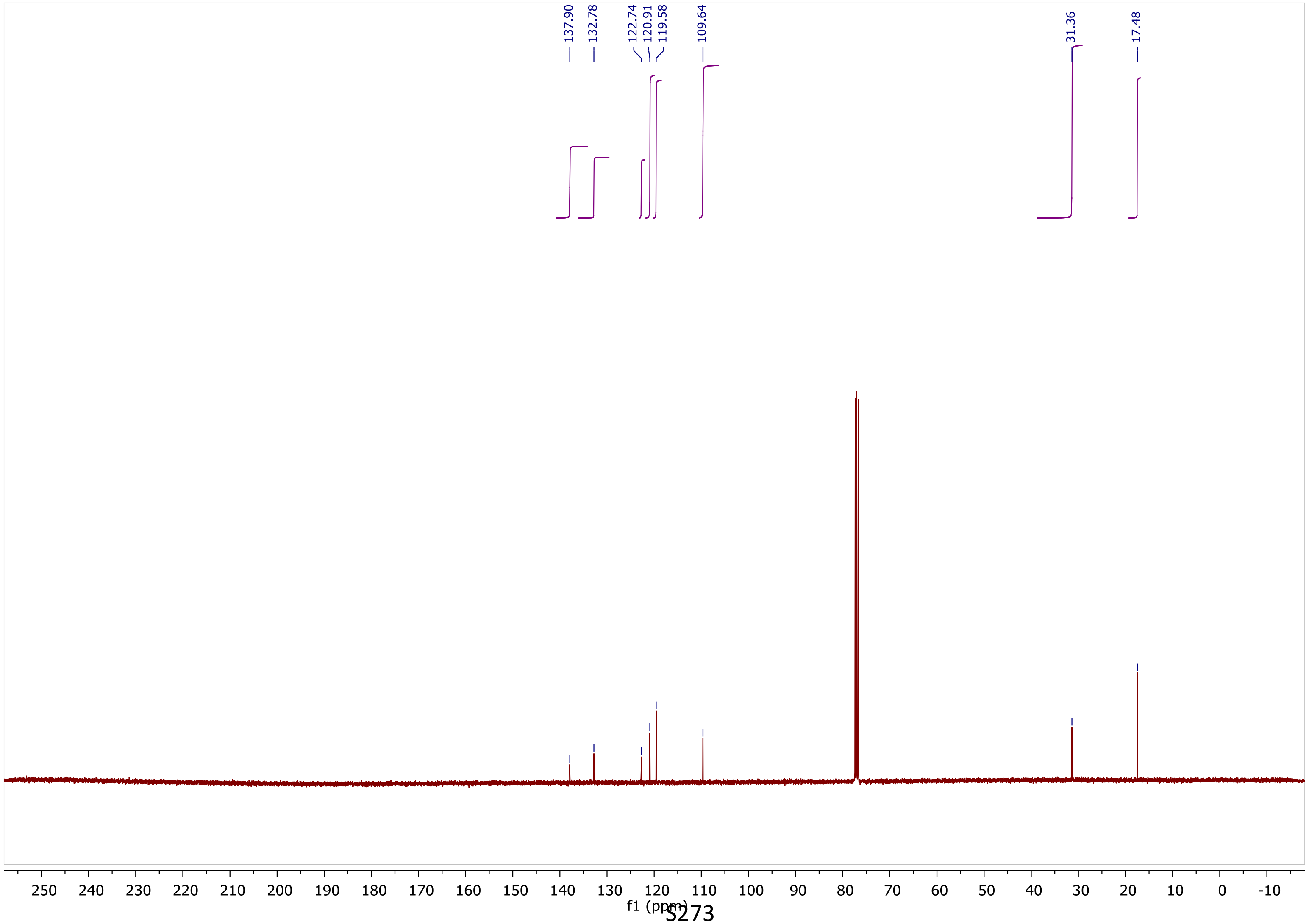

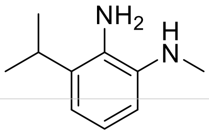

^{1}H NMR of 3-isopropyl-*N*1-methylbenzene-1,2-diamine **5c** in $CDCl_3$

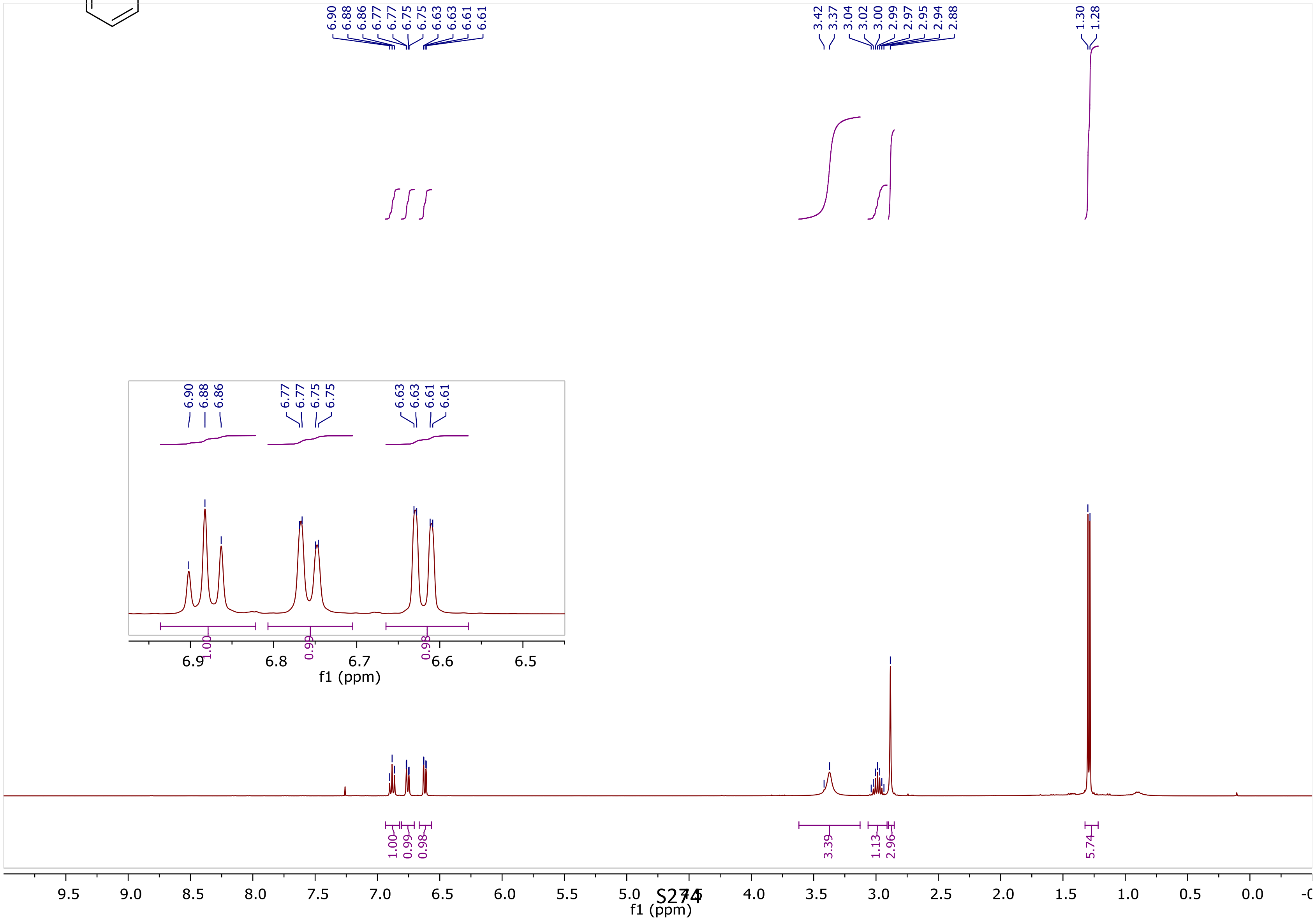

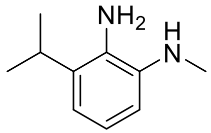

^{13}C NMR of 3-isopropyl-*N*1-methylbenzene-1,2-diamine **5c** in $CDCl_3$

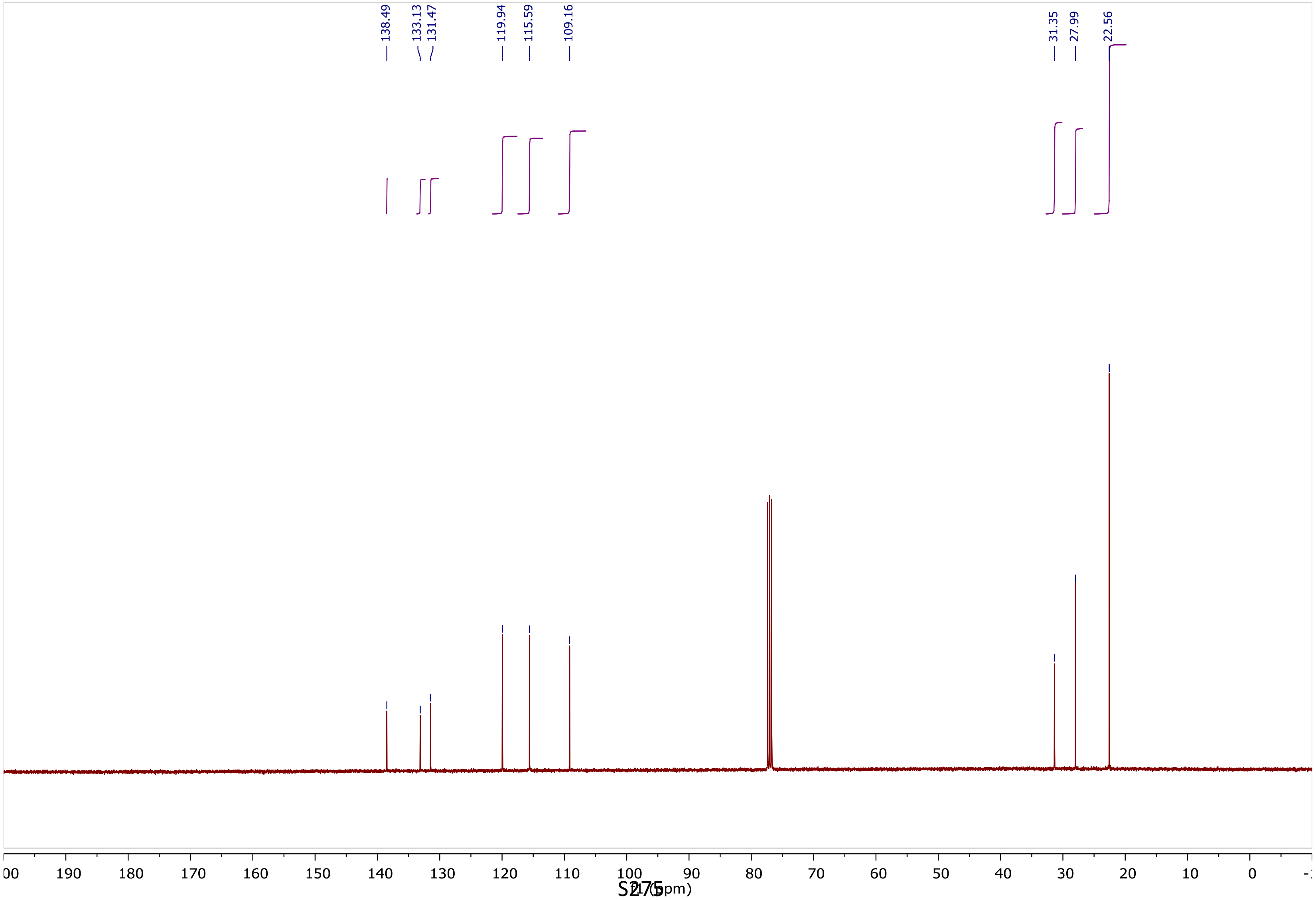

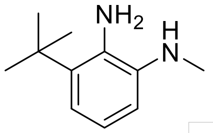

^{1}H NMR of 3-*tert*-butyl-*N*1-methylbenzene-1,2-diamine **5d** in $CDCl_3$

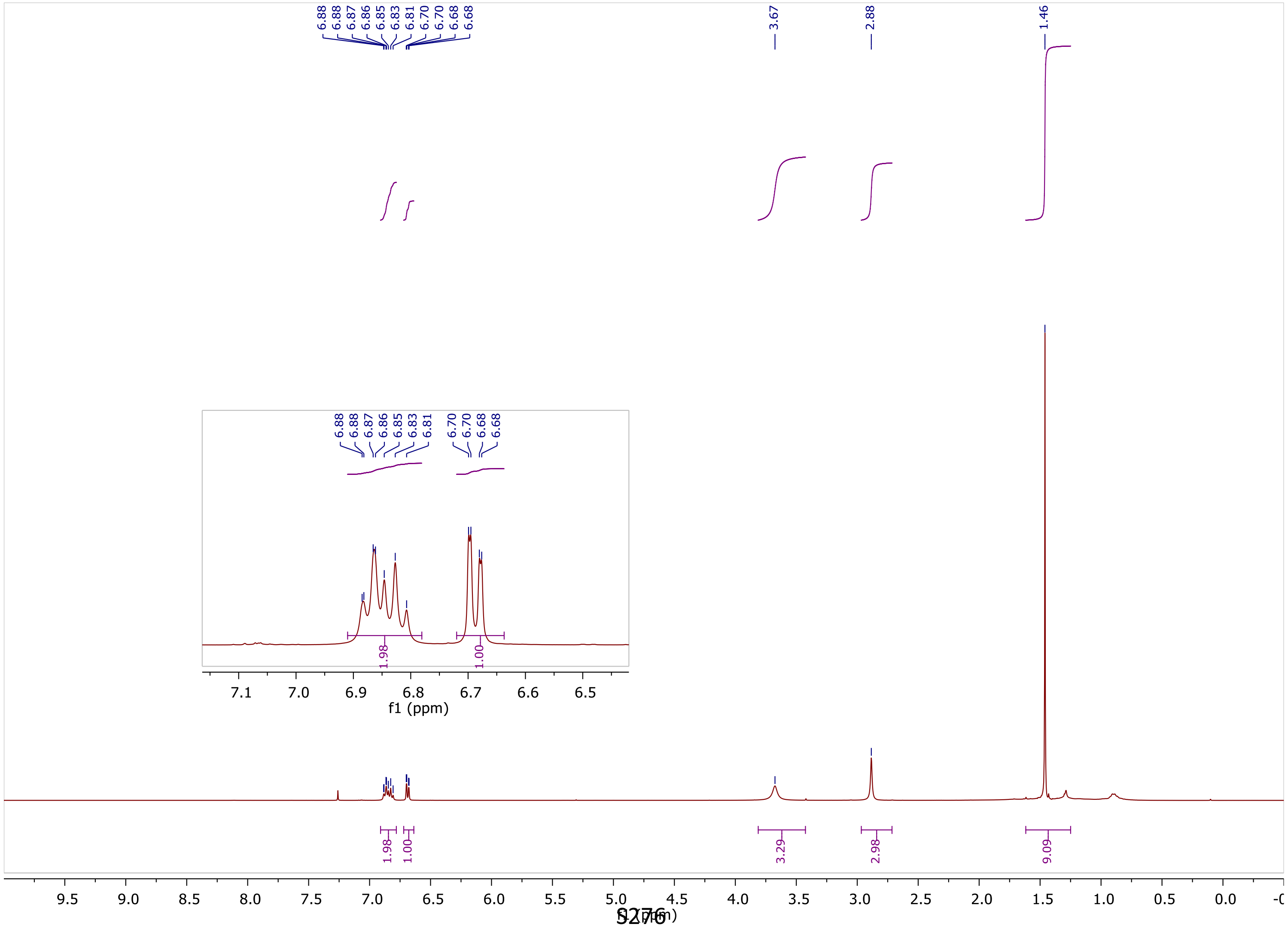

^{13}C NMR of 3-*tert*-butyl-*N*1-methylbenzene-1,2-diamine **5d** in $CDCl_3$

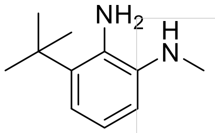

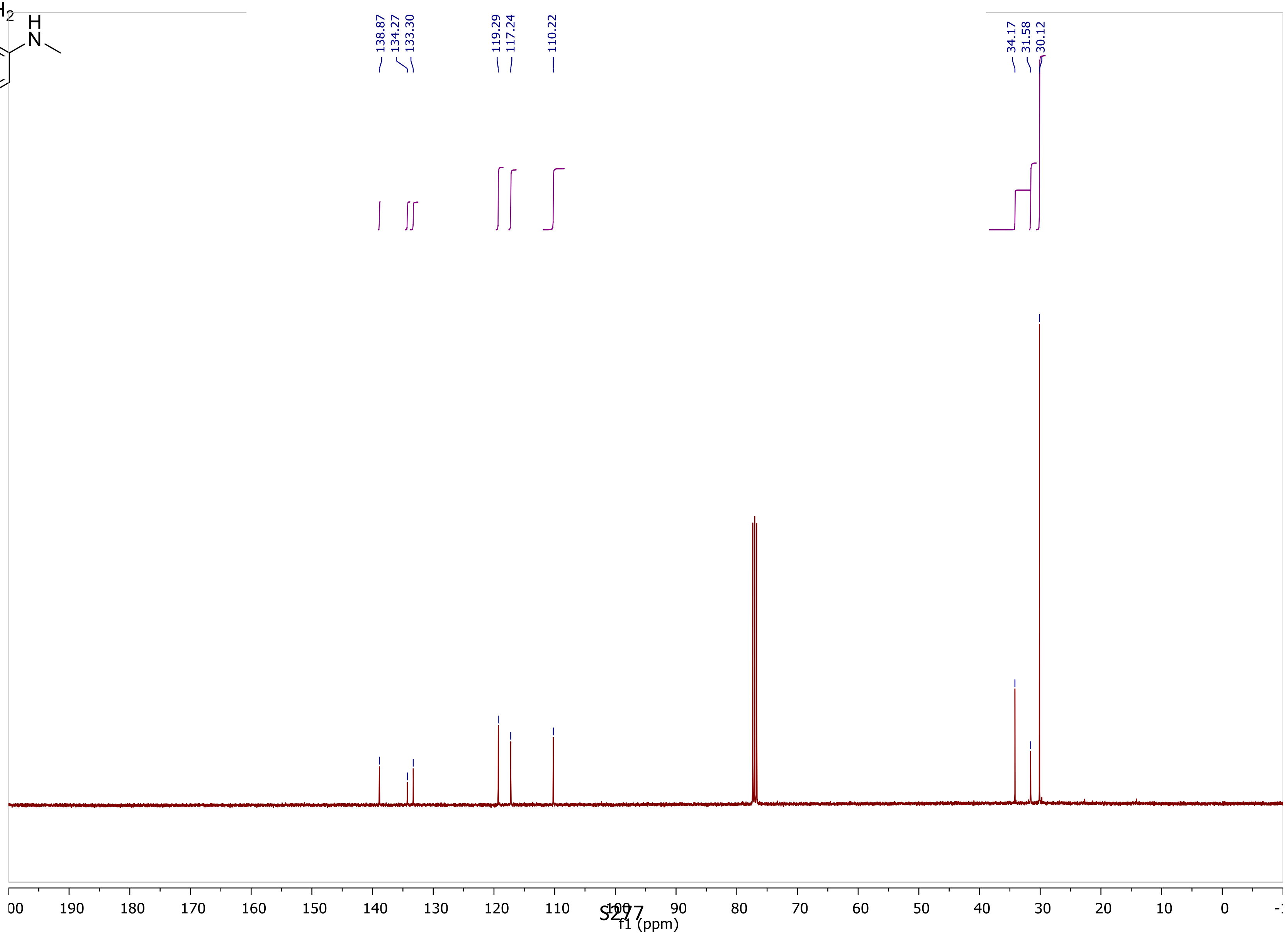

^{1}H NMR of *N*1,6-dimethylbenzene-1,2-diamine **5e-i** and *N*1,4-dimethylbenzene-1,2-diamine **5e-ii** in DMSO-d^6

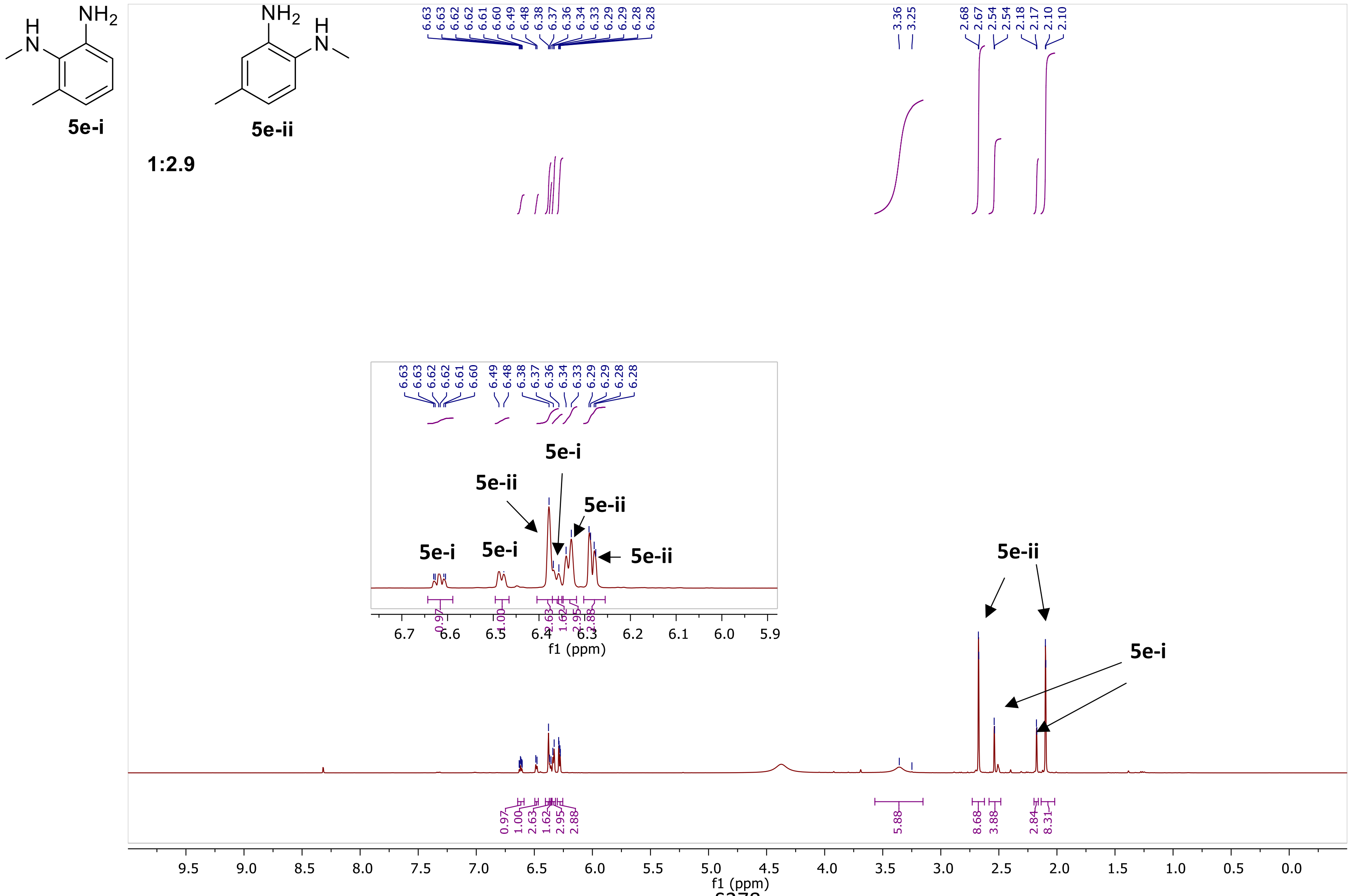

^{13}C NMR of *N*1,6-dimethylbenzene-1,2-diamine **5e-i** and *N*1,4-dimethylbenzene-1,2-diamine **5e-ii** in DMSO-d^6

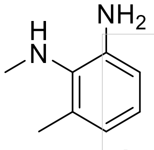

5e-i

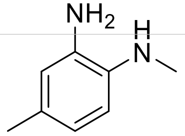

5e-ii

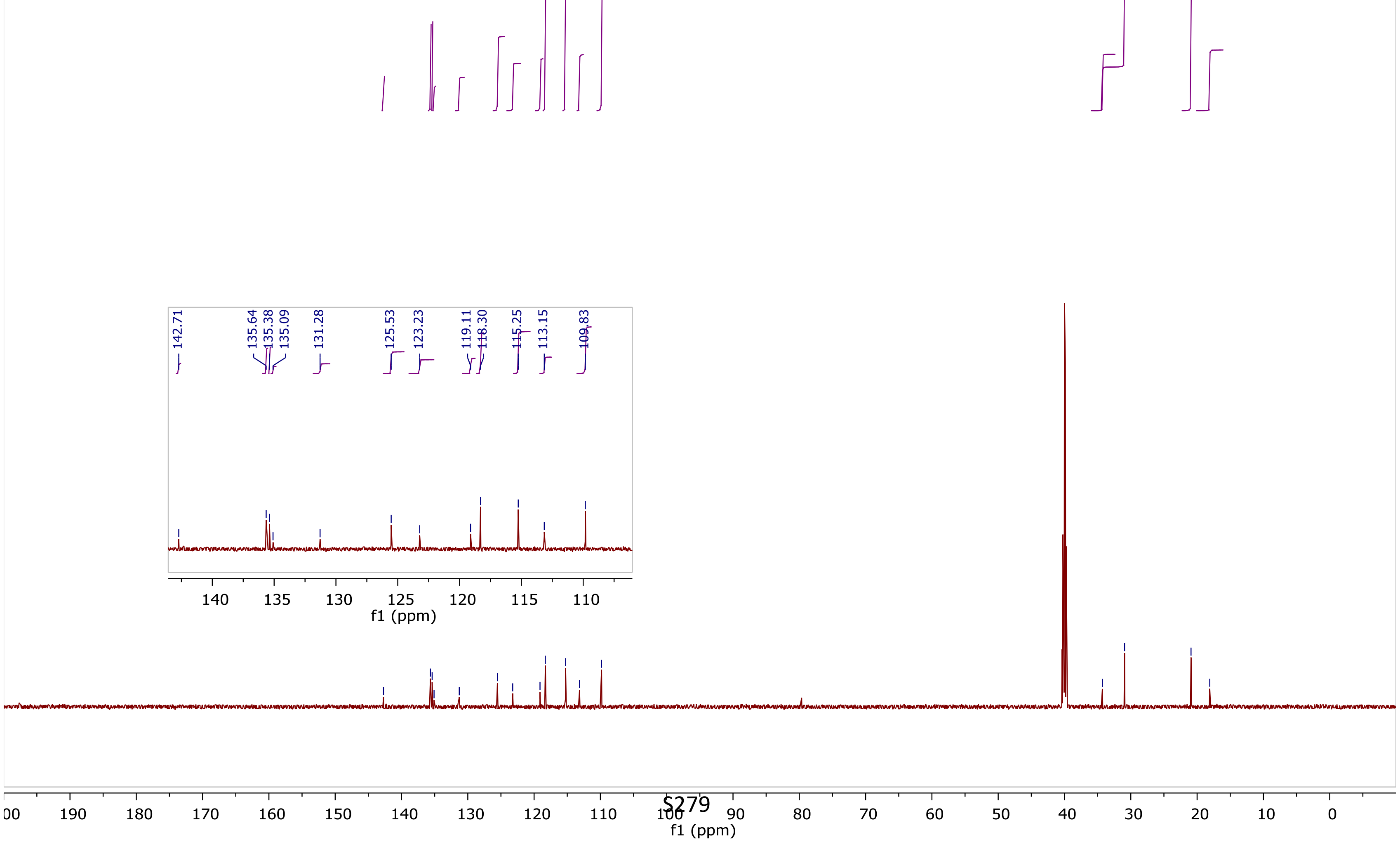

^1H NMR of 6-isopropyl-*N*1-methylbenzene-1,2-diamine **5f-i** and 4-isopropyl-*N*1-methylbenzene-1,2-diamine **5f-ii** in DMSO-d^6

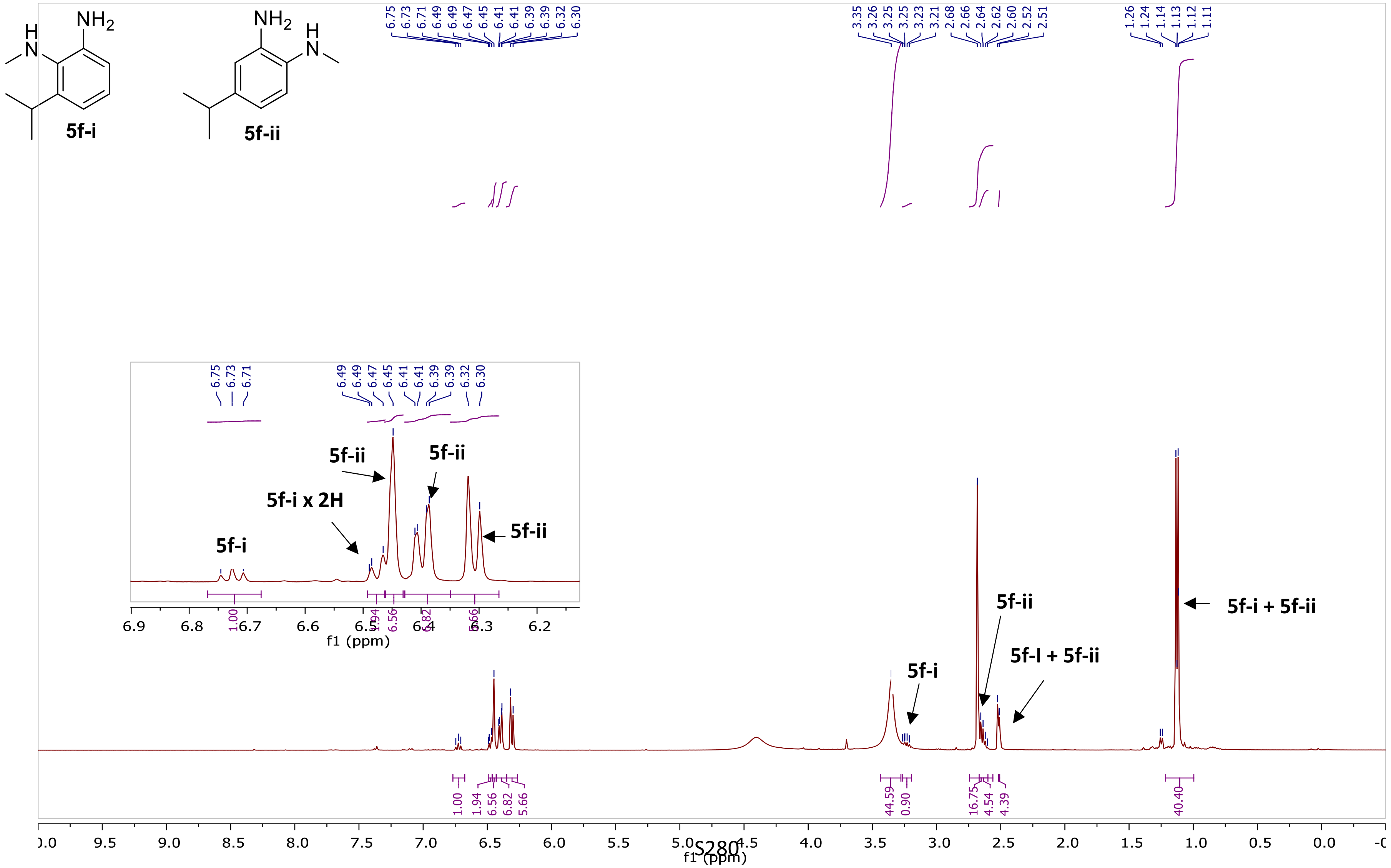

^{13}C NMR of 6-isopropyl-*N*1-methylbenzene-1,2-diamine **5f-i** and 4-isopropyl-*N*1-methylbenzene-1,2-diamine **5f-ii** in DMSO-d[6]

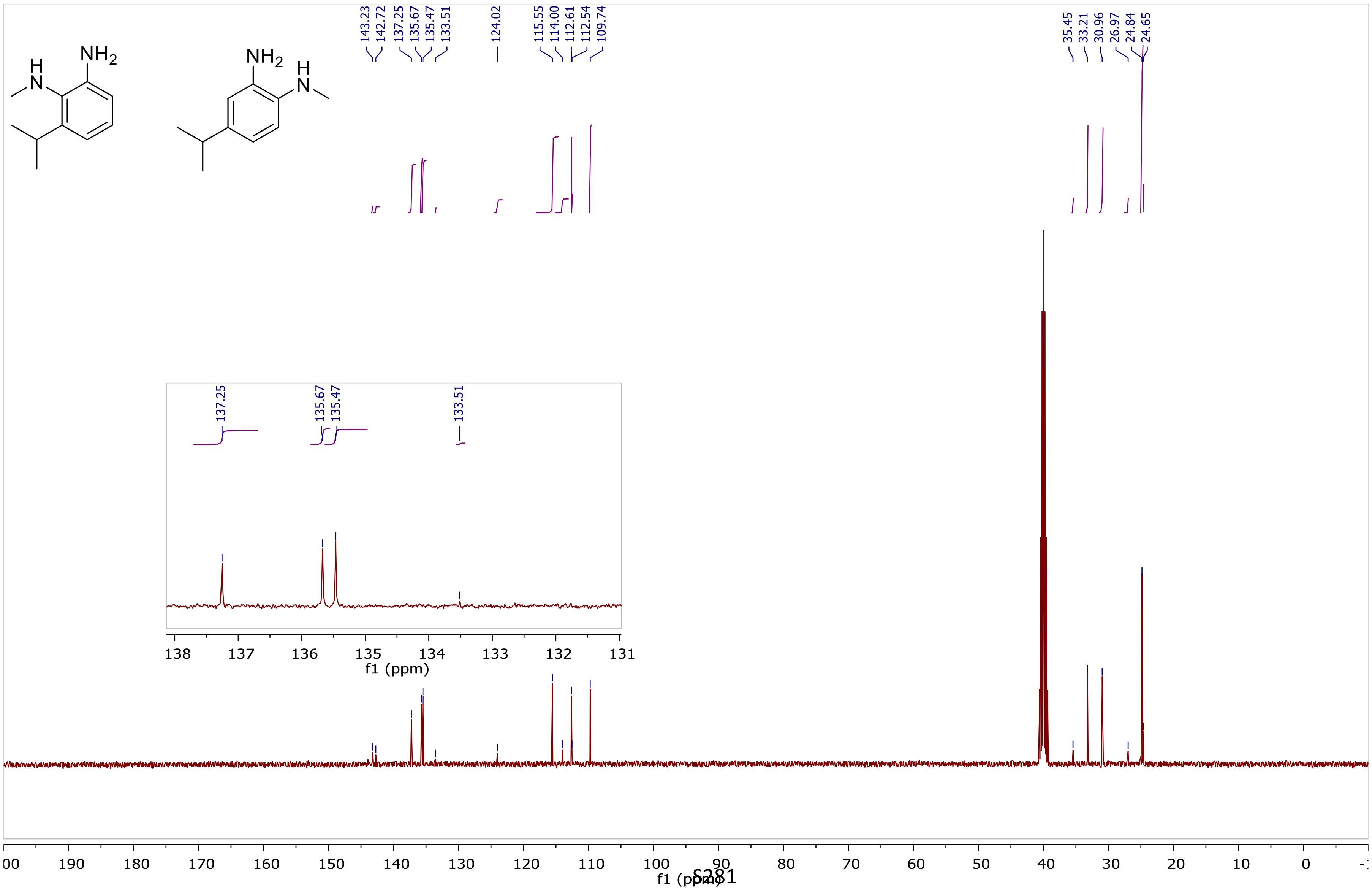

^{1}H NMR of 3-(hex-1-yn-1-yl)-N1-methylbenzene-1,2-diamine **5g** in $CDCl_3$

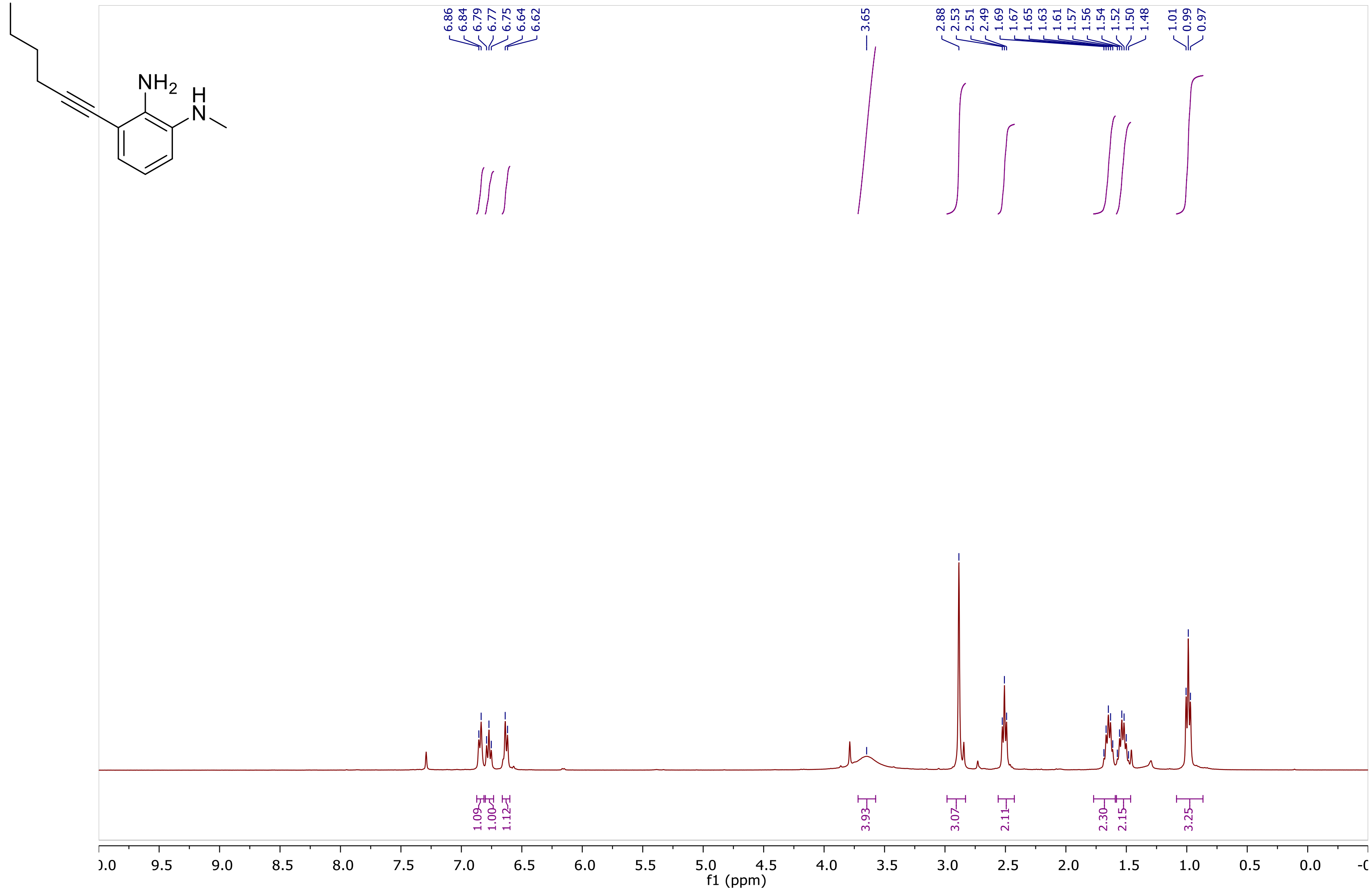

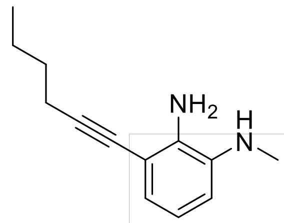

^{13}C NMR of 3-(hex-1-yn-1-yl)-N1-methylbenzene-1,2-diamine **5g** in $CDCl_3$

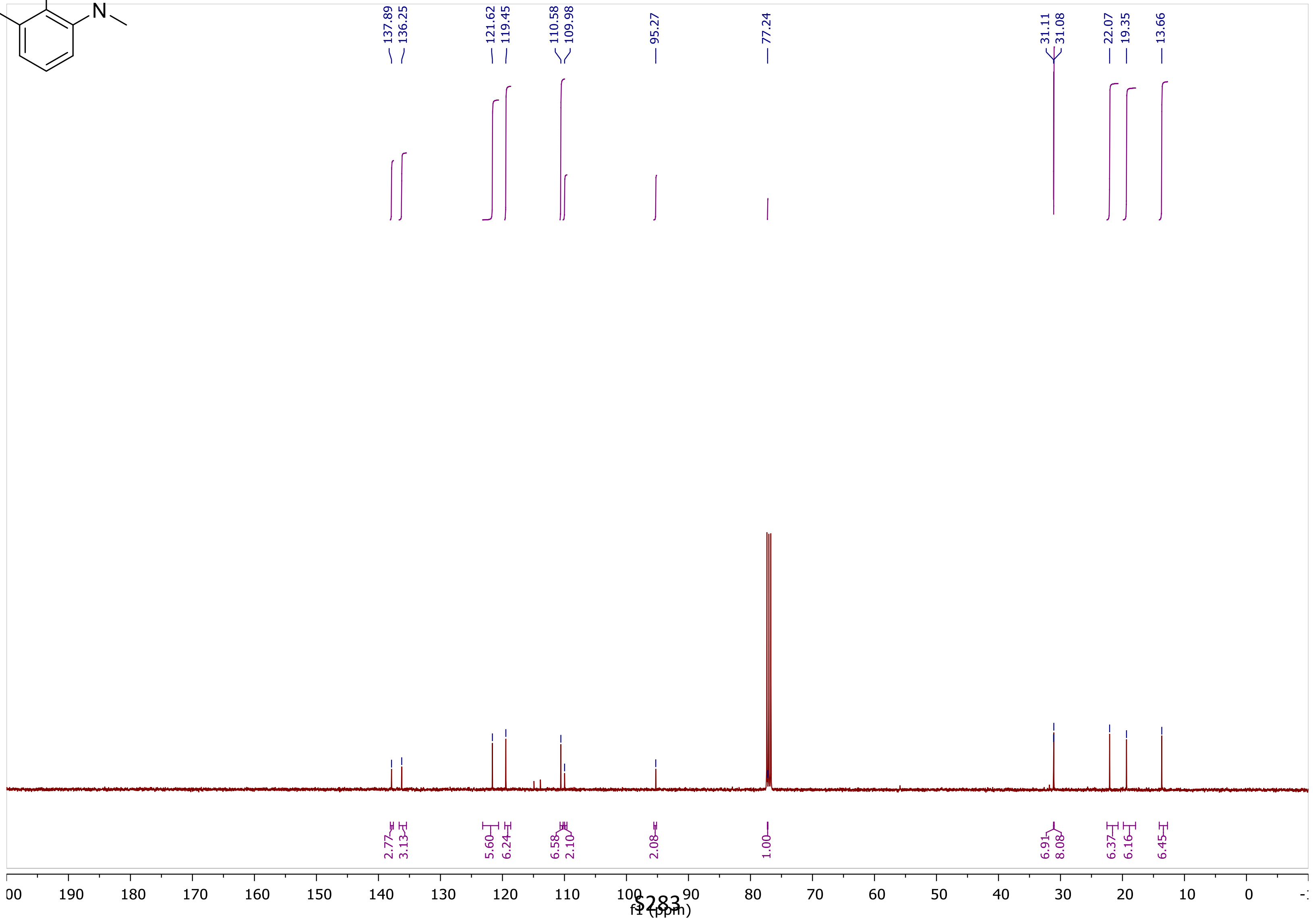

^{1}H NMR of *N*1,5-dimethylbenzene-1,2-diamine **5h** in $CDCl_3$

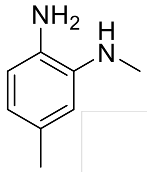

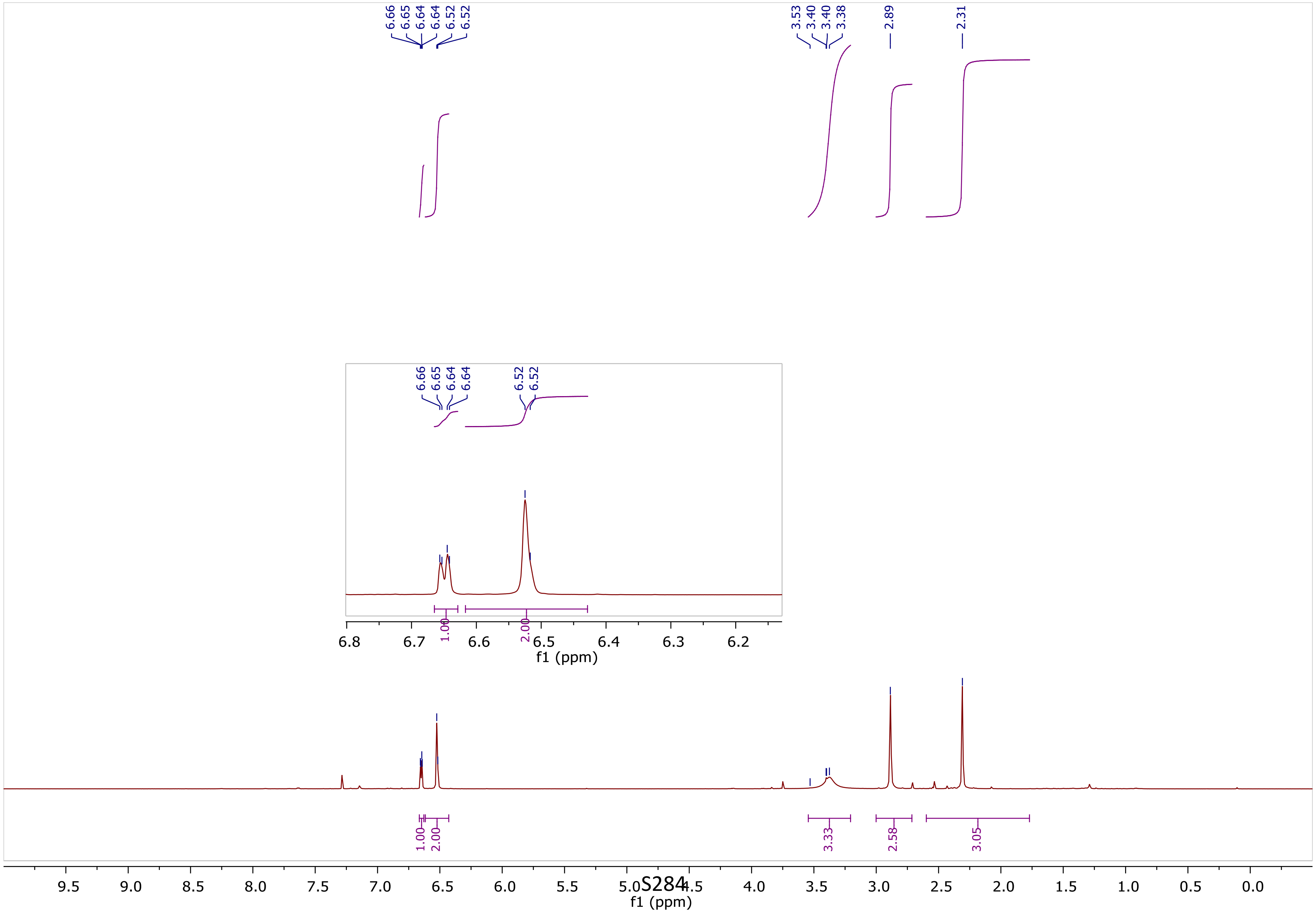

^{13}C NMR of *N*1,5-dimethylbenzene-1,2-diamine **5h** in $CDCl_3$

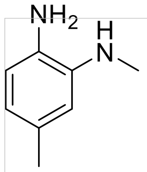

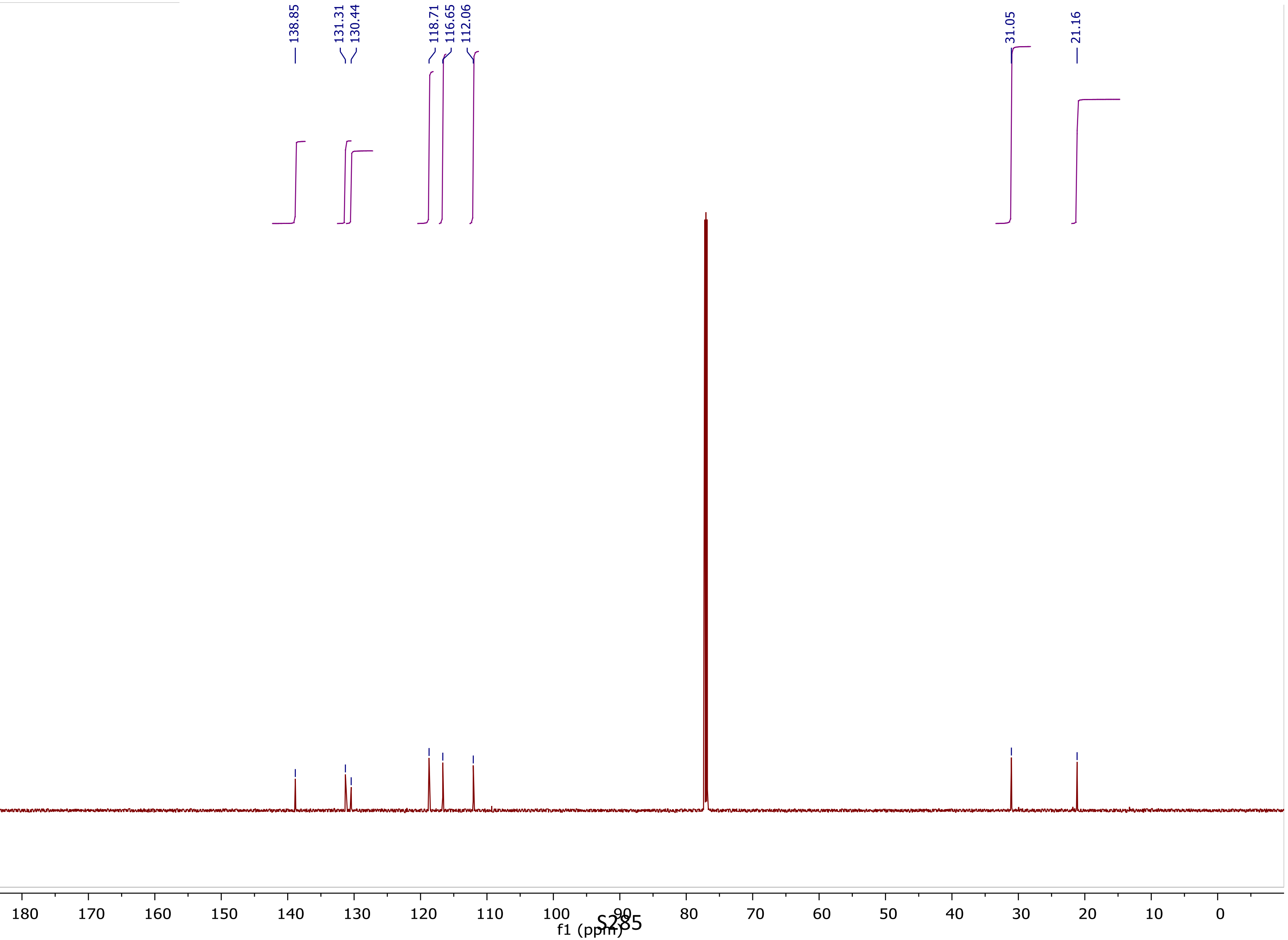

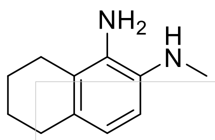

^{1}H NMR of *N*2-methyl-5,6,7,8-tetrahydronaphthalene-1,2-diamine **5i** in MeOD-d[4]

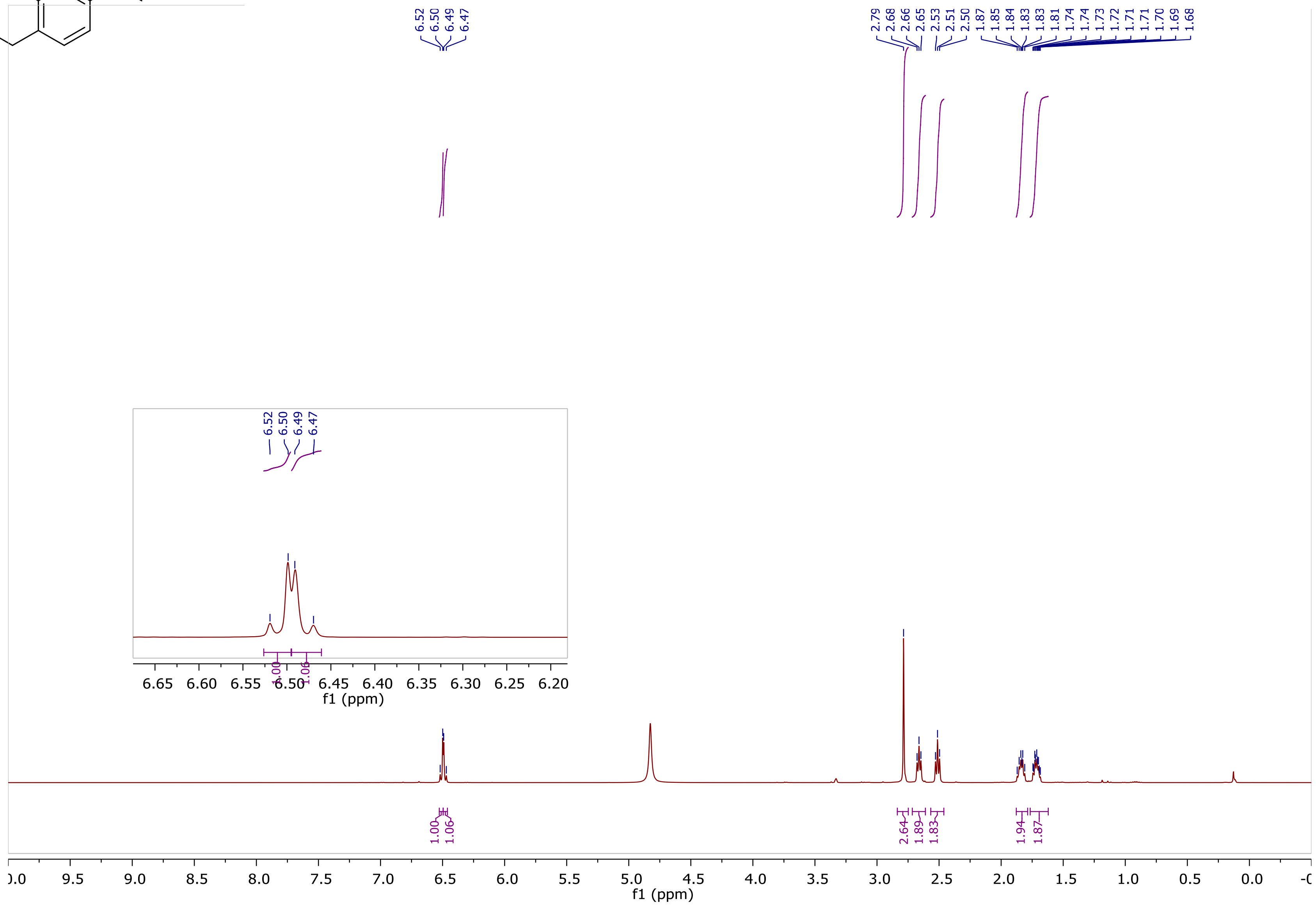

^{13}C NMR of *N*2-methyl-5,6,7,8-tetrahydronaphthalene-1,2-diamine **5i** in MeOD-d4

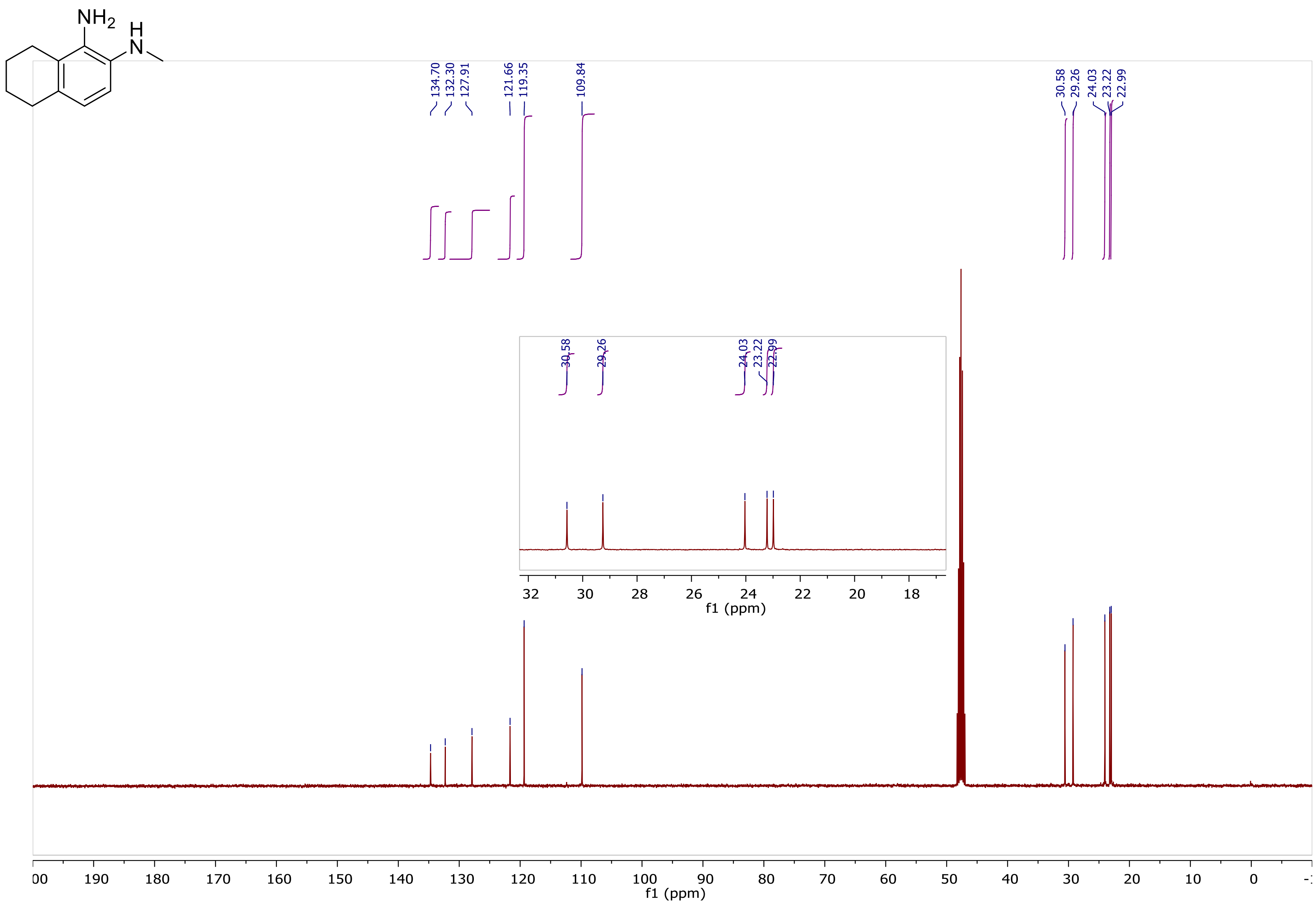

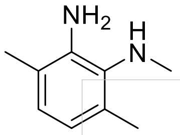

^{1}H NMR of *N*1,3,6-trimethylbenzene-1,2-diamine **5j** in $CDCl_3$

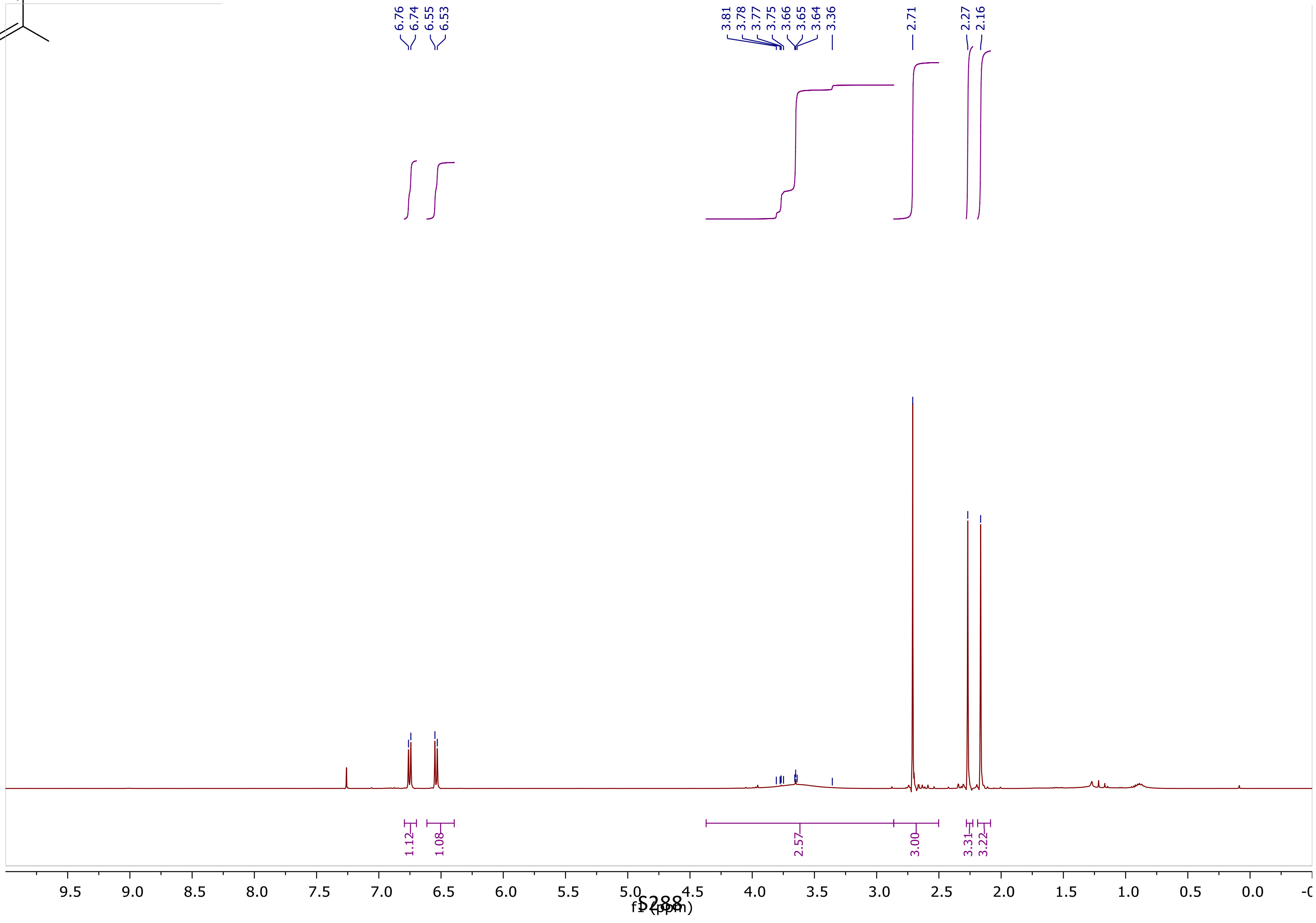

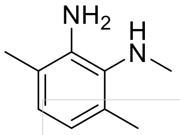

^{13}C NMR of *N*1,3,6-trimethylbenzene-1,2-diamine **5j** in $CDCl_3$

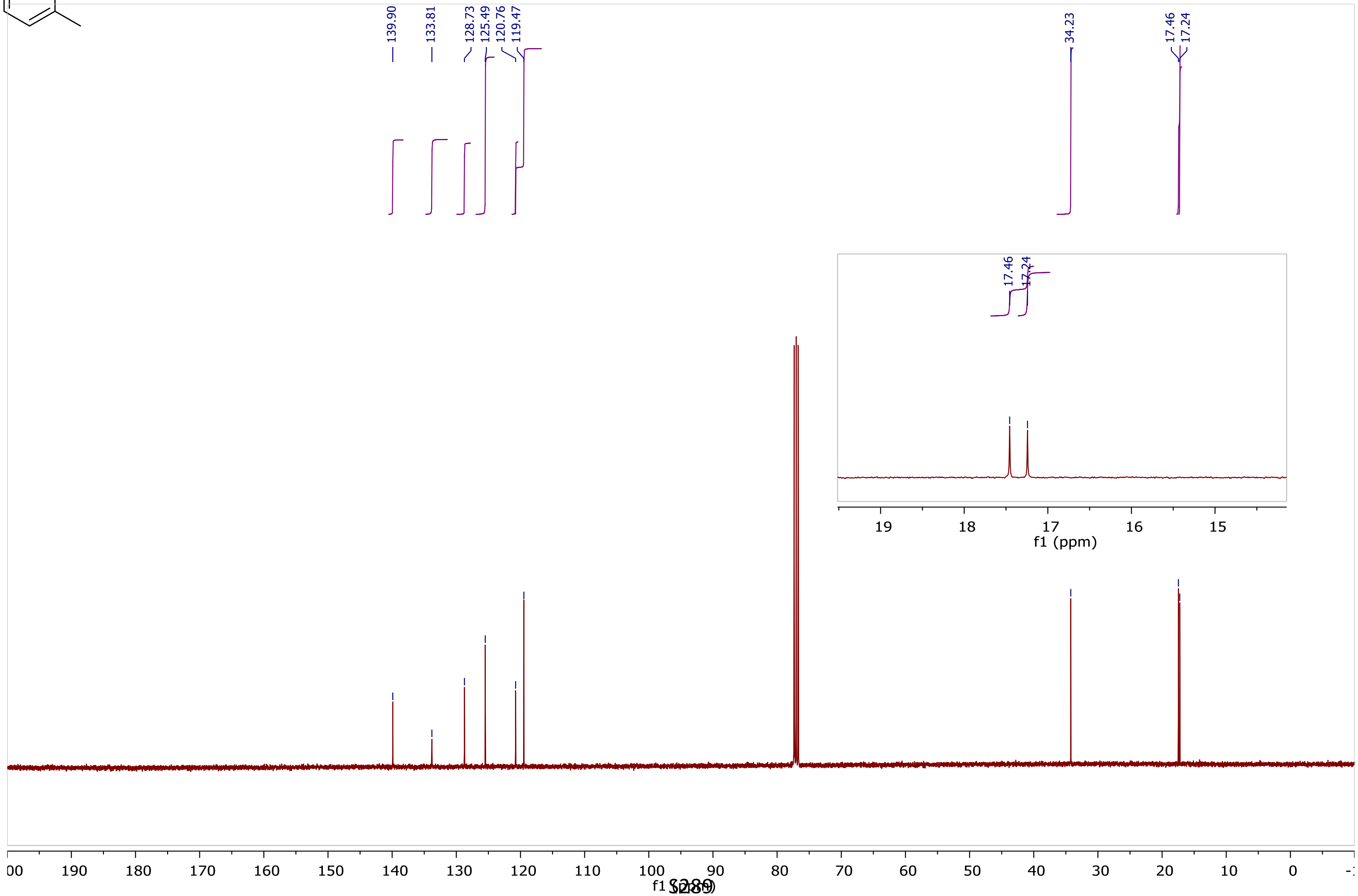

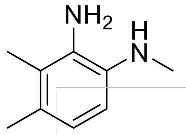

^{1}H NMR of *N*1,3,4-trimethylbenzene-1,2-diamine **5k** in $CDCl_3$

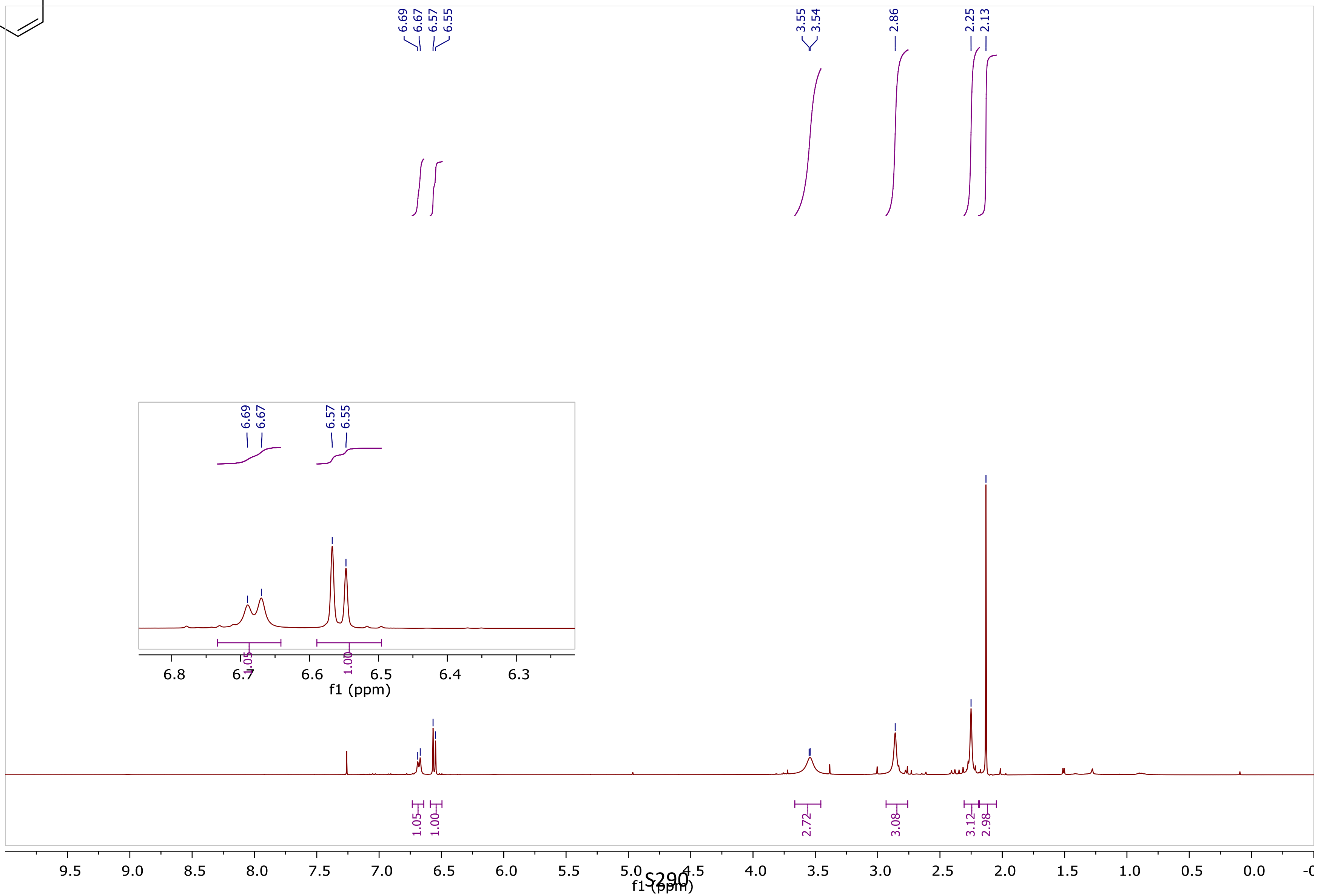

^{13}C NMR of *N*1,3,4-trimethylbenzene-1,2-diamine **5k** in $CDCl_3$

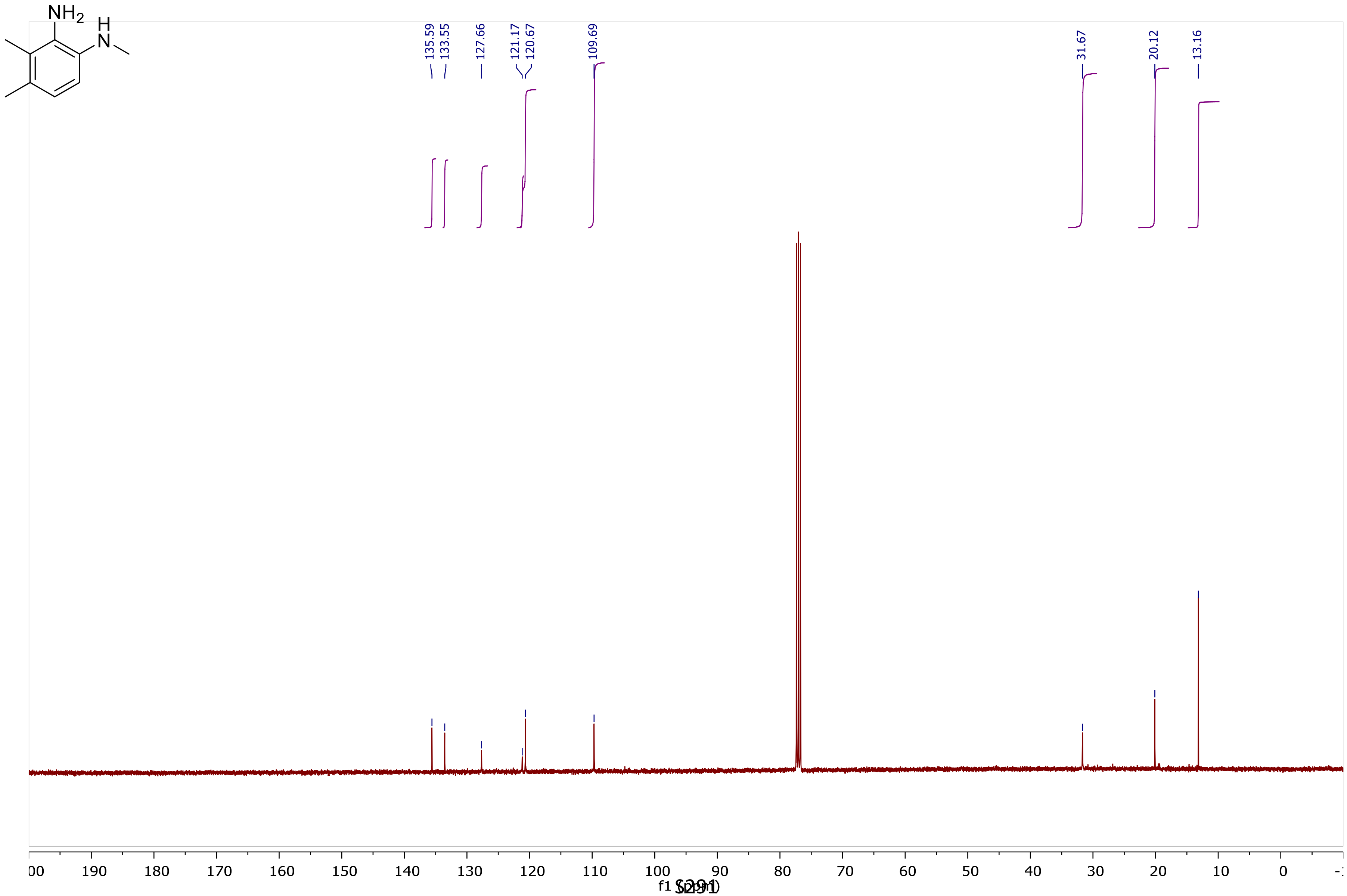

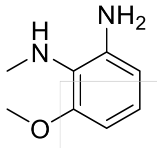

^1H NMR of 6-methoxy-*N*1-methylbenzene-1,2-diamine **5l-i** in $CDCl_3$

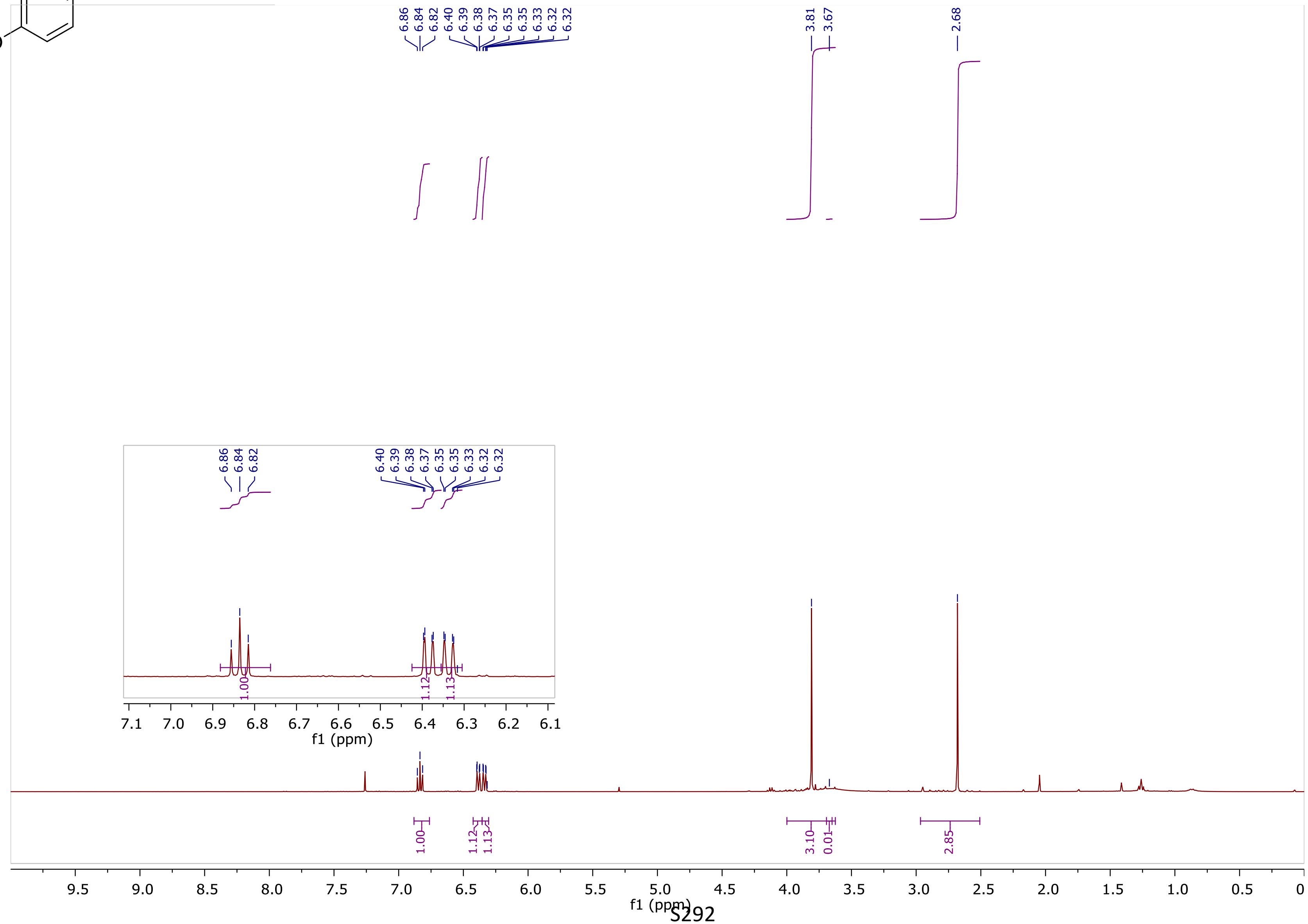

^{13}C NMR of 6-methoxy-*N*1-methylbenzene-1,2-diamine **5l-ii** in $CDCl_3$

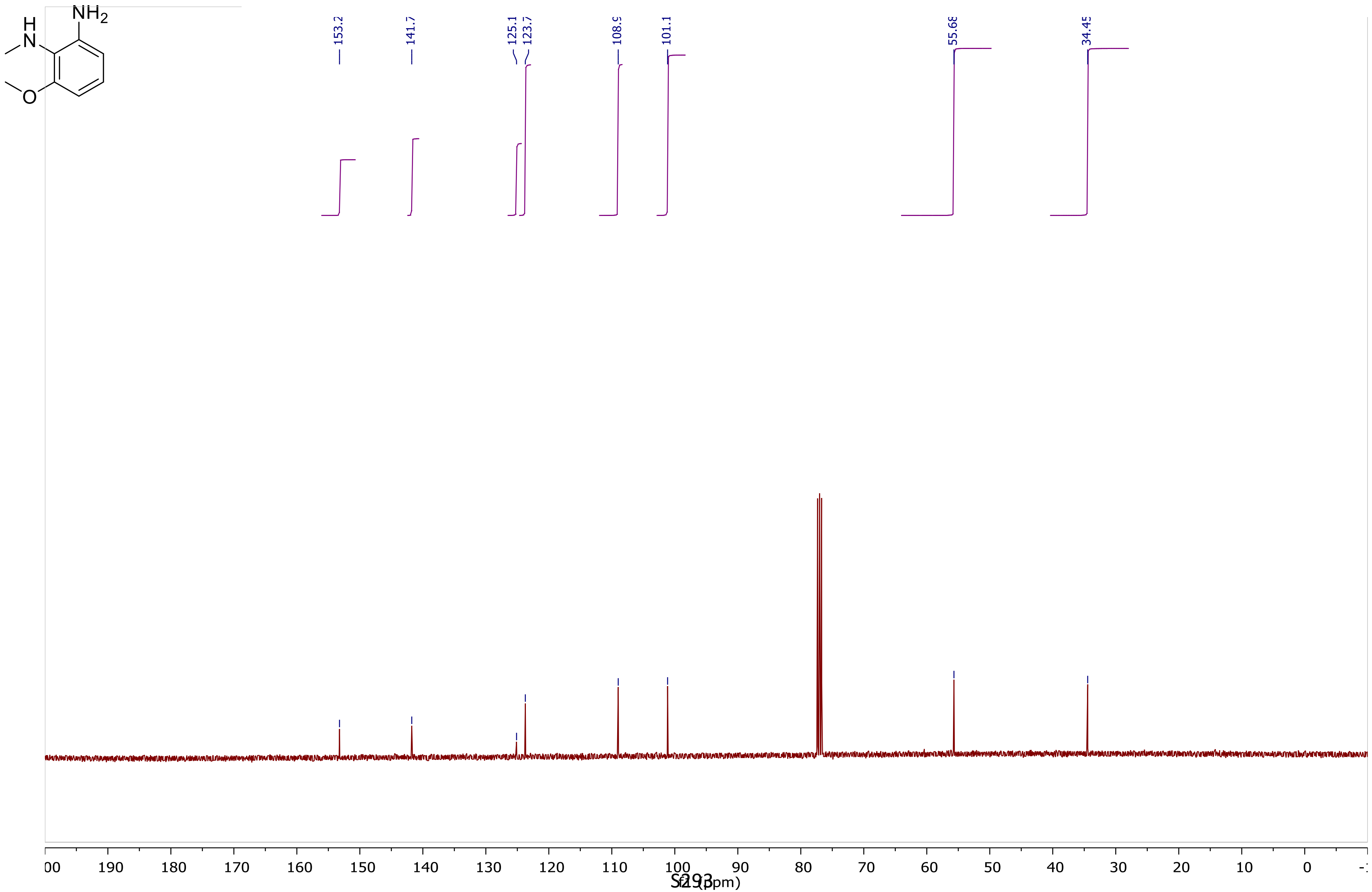

^{1}H NMR of 4-methoxy-*N*1-methylbenzene-1,2-diamine **5l-ii** in MeOD-d[4]

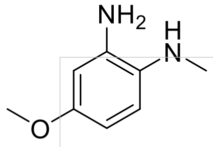

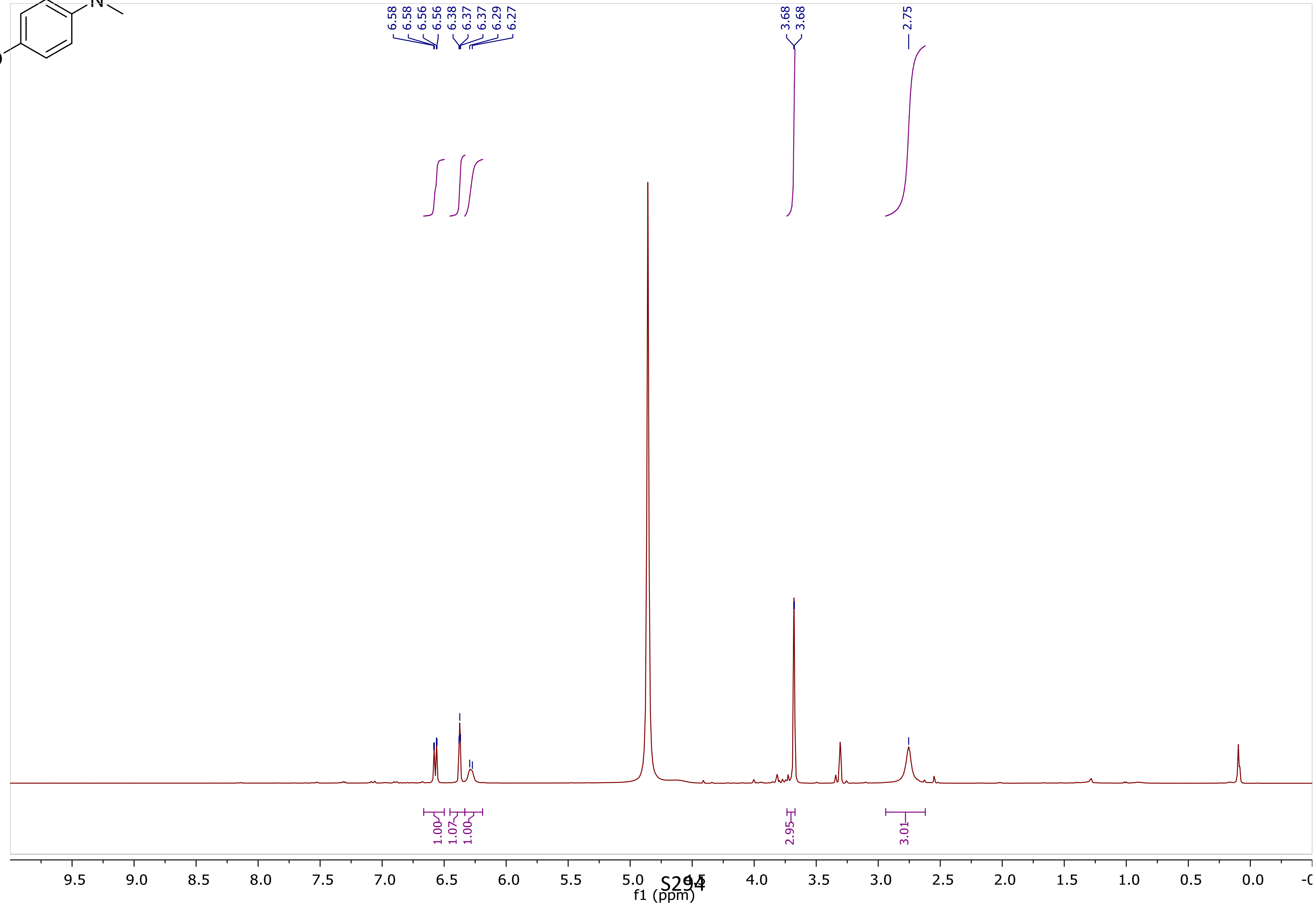

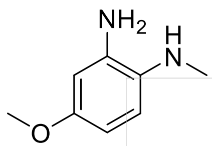

^{13}C NMR of 4-methoxy-*N*1-methylbenzene-1,2-diamine **5l-ii** in MeOD-d[4]

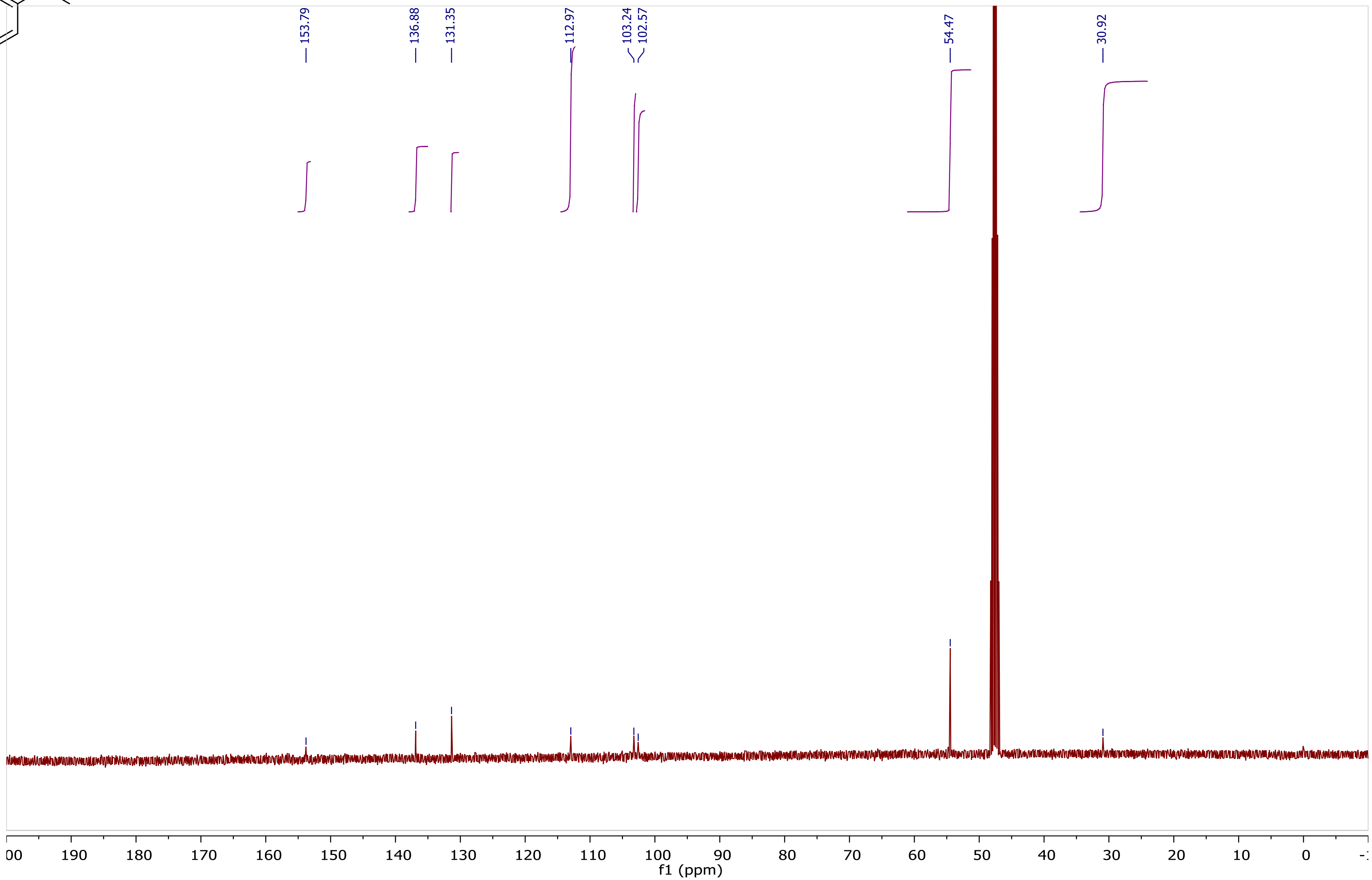

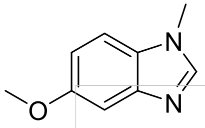

^{1}H NMR of 5-methoxy-1-methyl-1*H*-benzo[d]imidazole **5l-iii** in $CDCl_3$

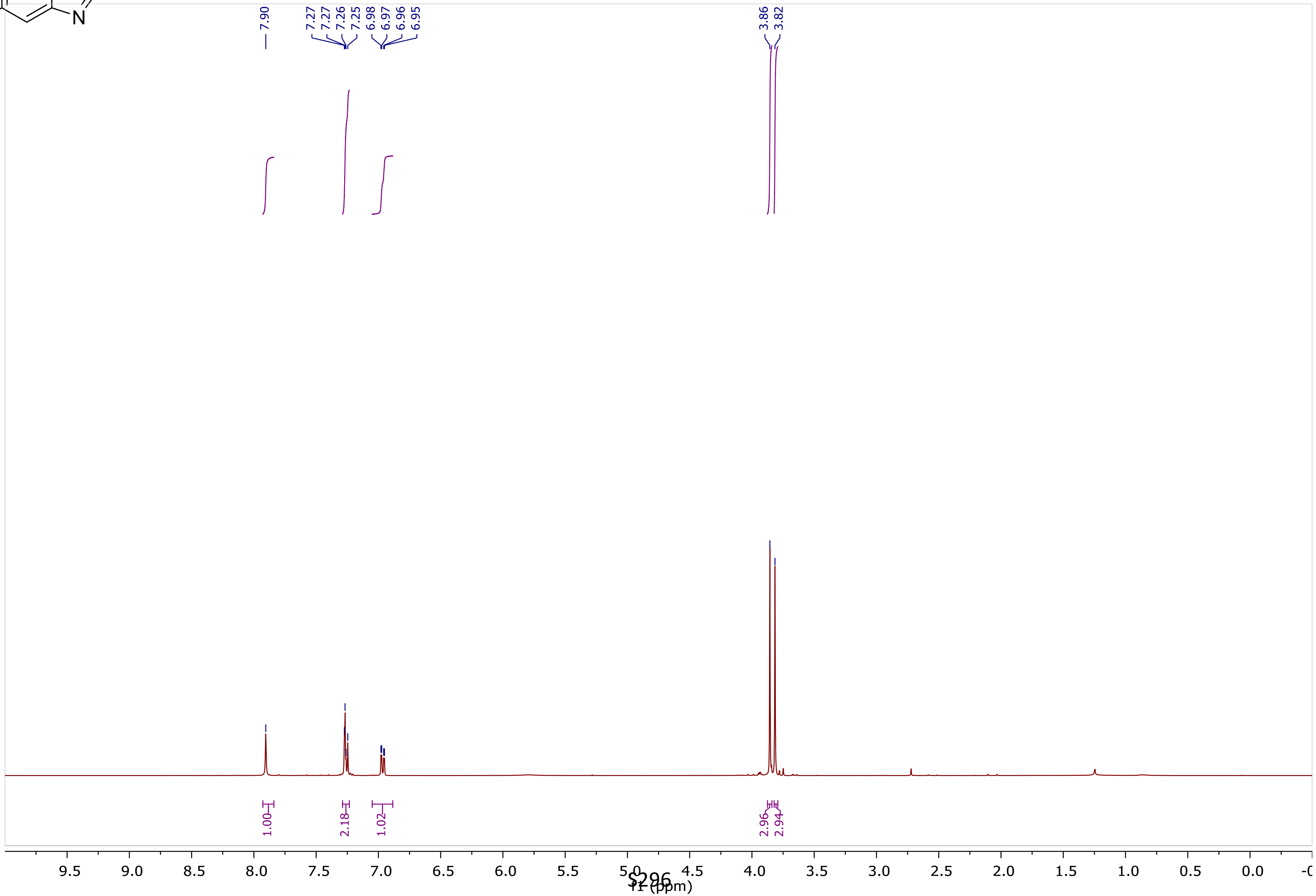

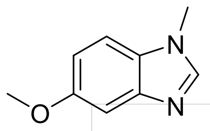

^{13}C NMR of 5-methoxy-1-methyl-1*H*-benzo[d]imidazole **5l-iii** in $CDCl_3$

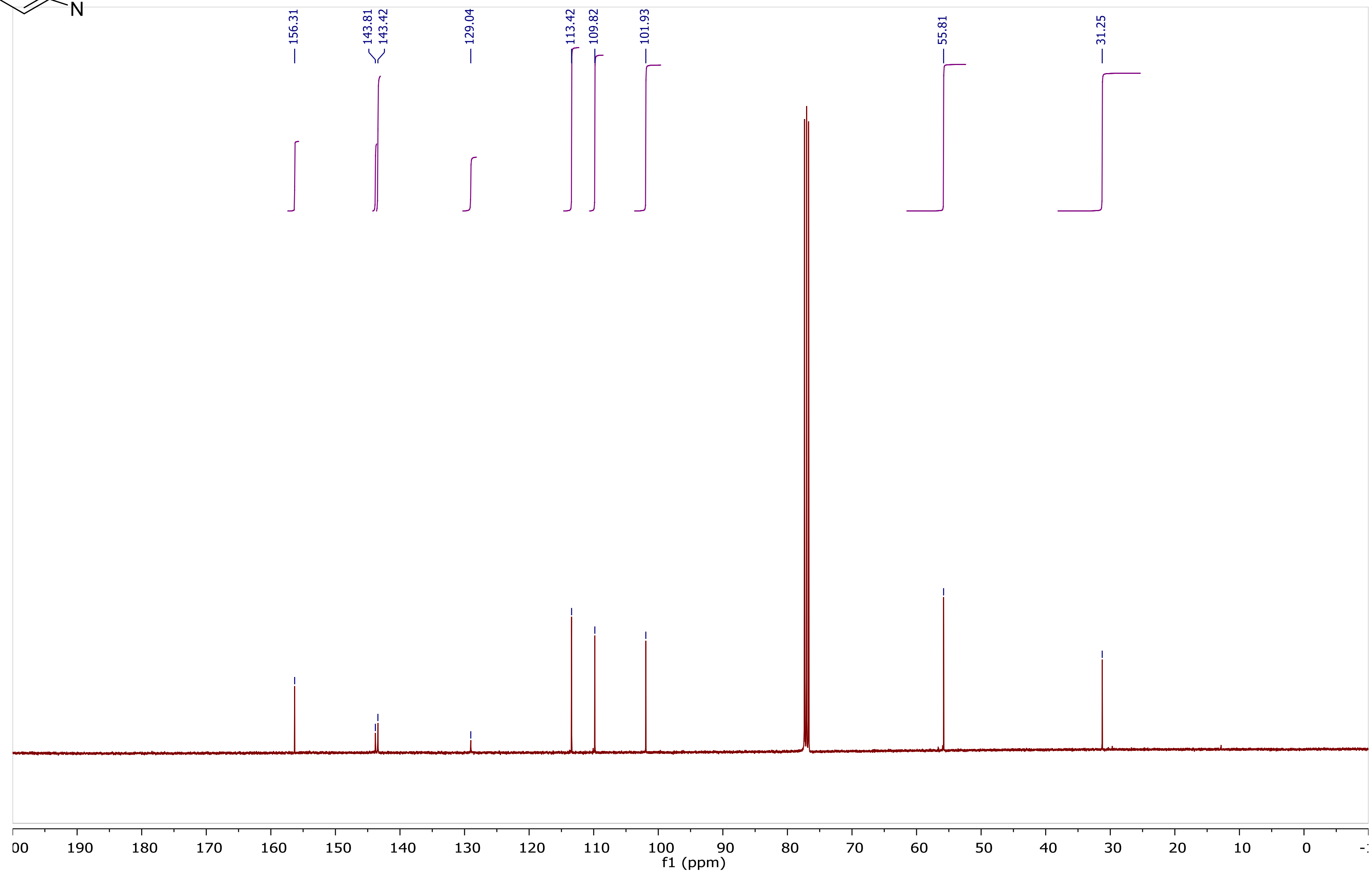

^{1}H NMR of 4-methoxy-*N*1,3-dimethylbenzene-1,2-diamine **5m-i** in $CDCl_3$

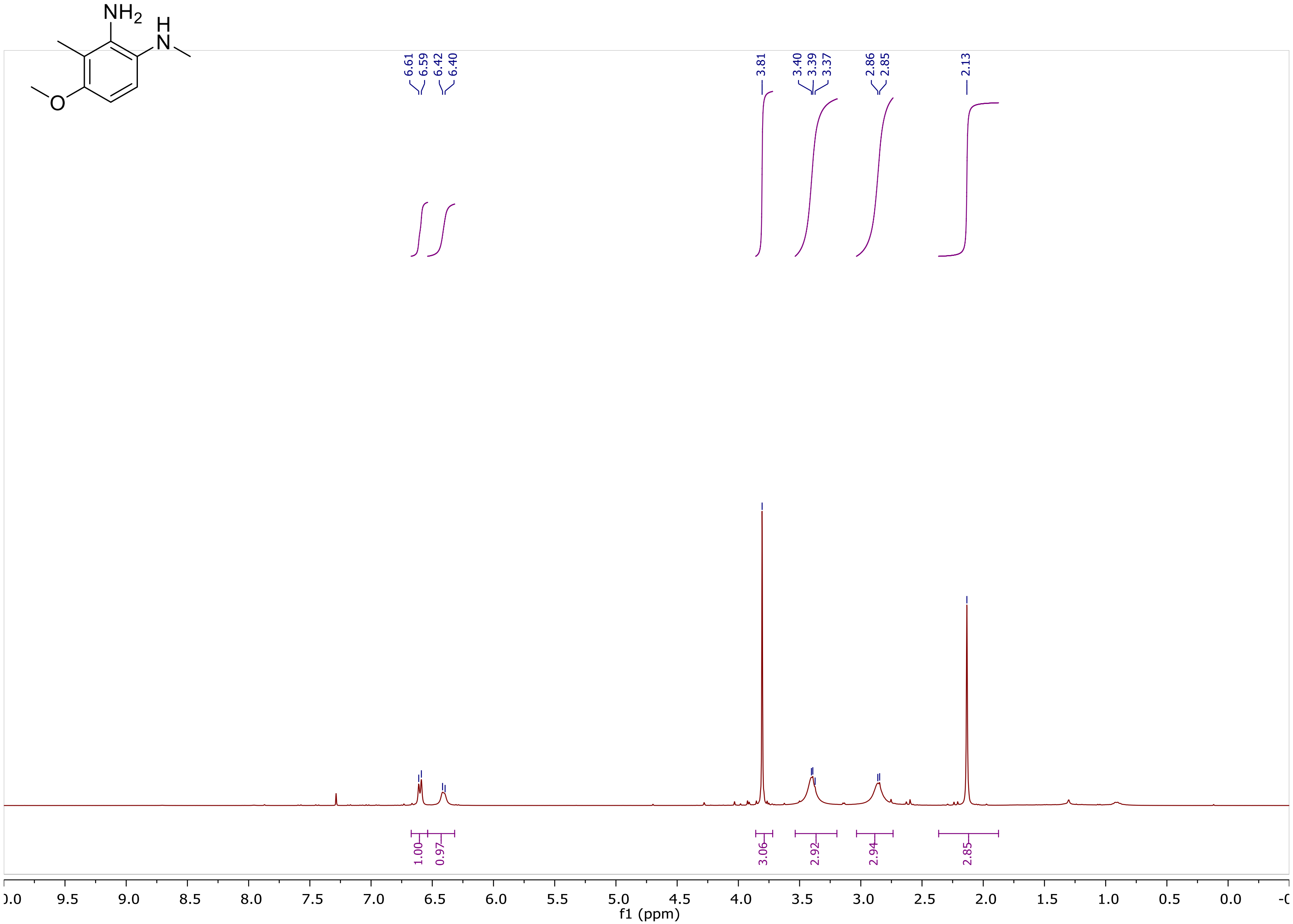

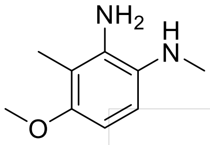

^{13}C NMR of 4-methoxy-*N*1,3-dimethylbenzene-1,2-diamine **5m-i** in $CDCl_3$

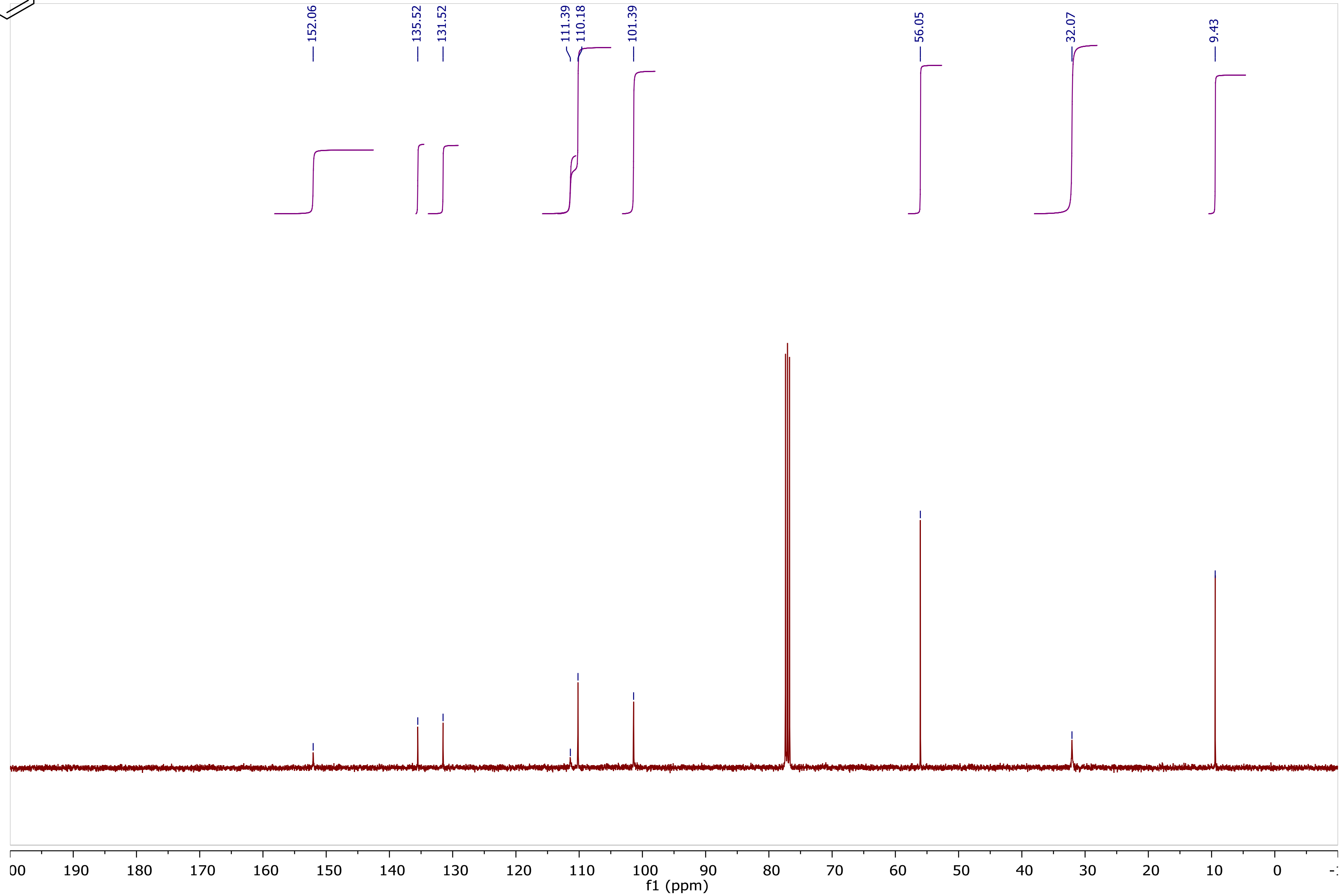

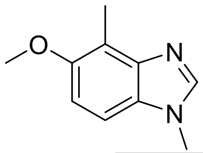

^{1}H NMR of 5-methoxy-1,4-dimethyl-1*H*-benzo[d]imidazole **5m-ii** in $CDCl_3$

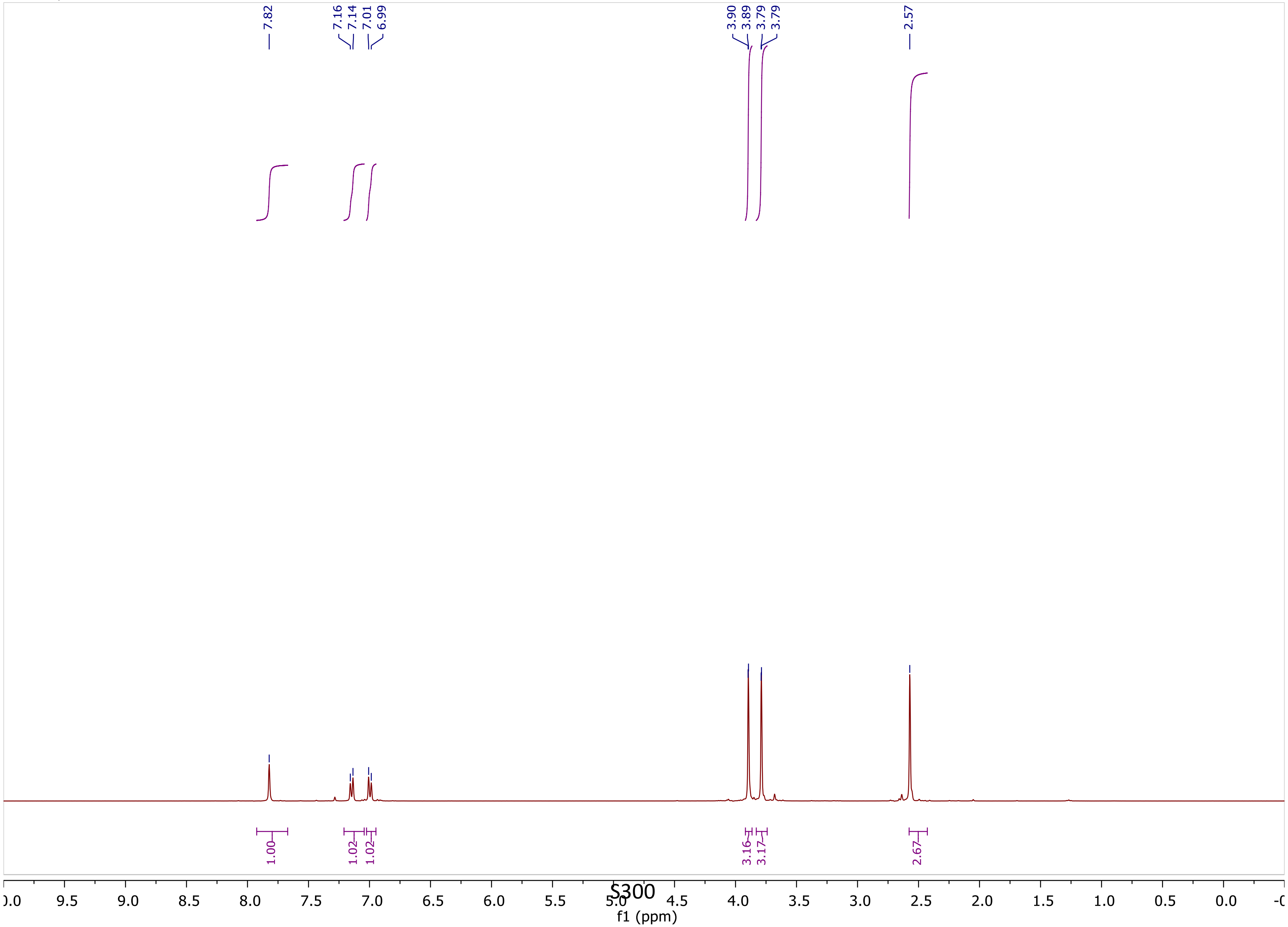

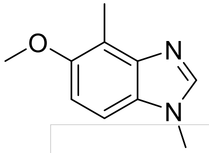

^{13}C NMR of 5-methoxy-1,4-dimethyl-1*H*-benzo[d]imidazole **5m-ii** in $CDCl_3$

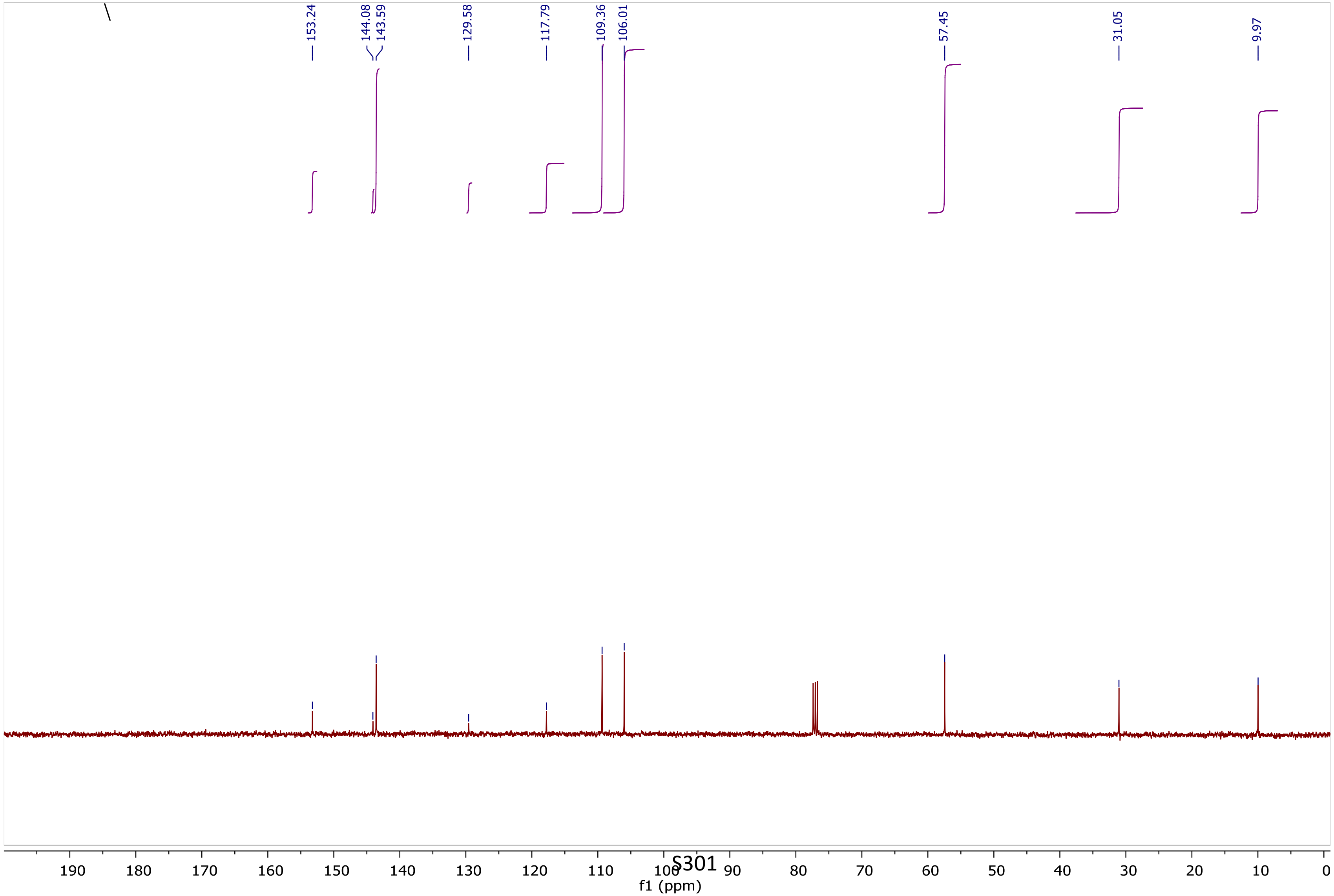

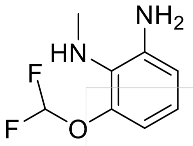

^{1}H NMR of 6-(difluoromethoxy)-*N*1-methylbenzene-1,2-diamine **5n-i** in $CDCl_3$

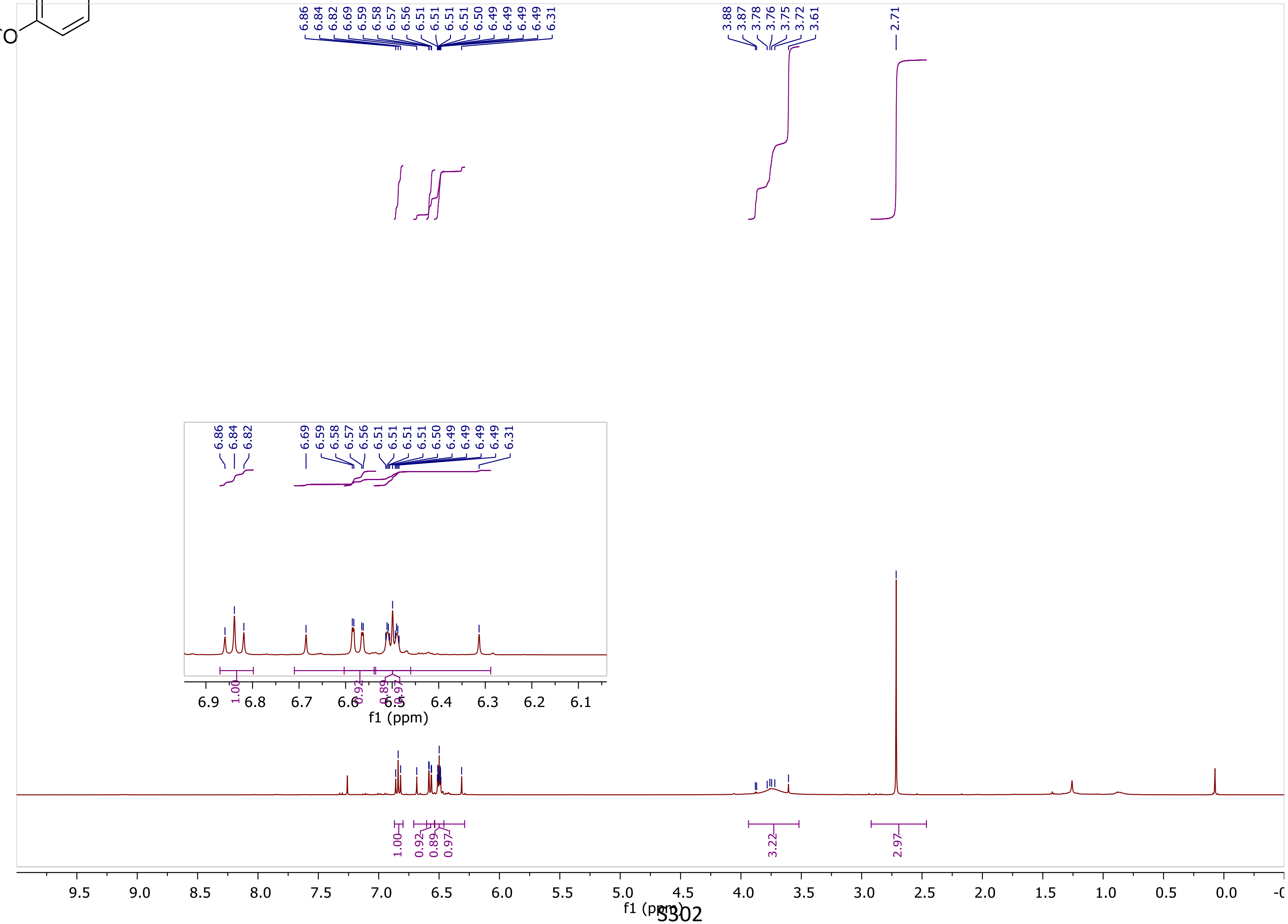

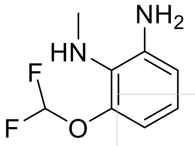

^{13}C NMR of 6-(difluoromethoxy)-*N*1-methylbenzene-1,2-diamine **5n-i** in $CDCl_3$

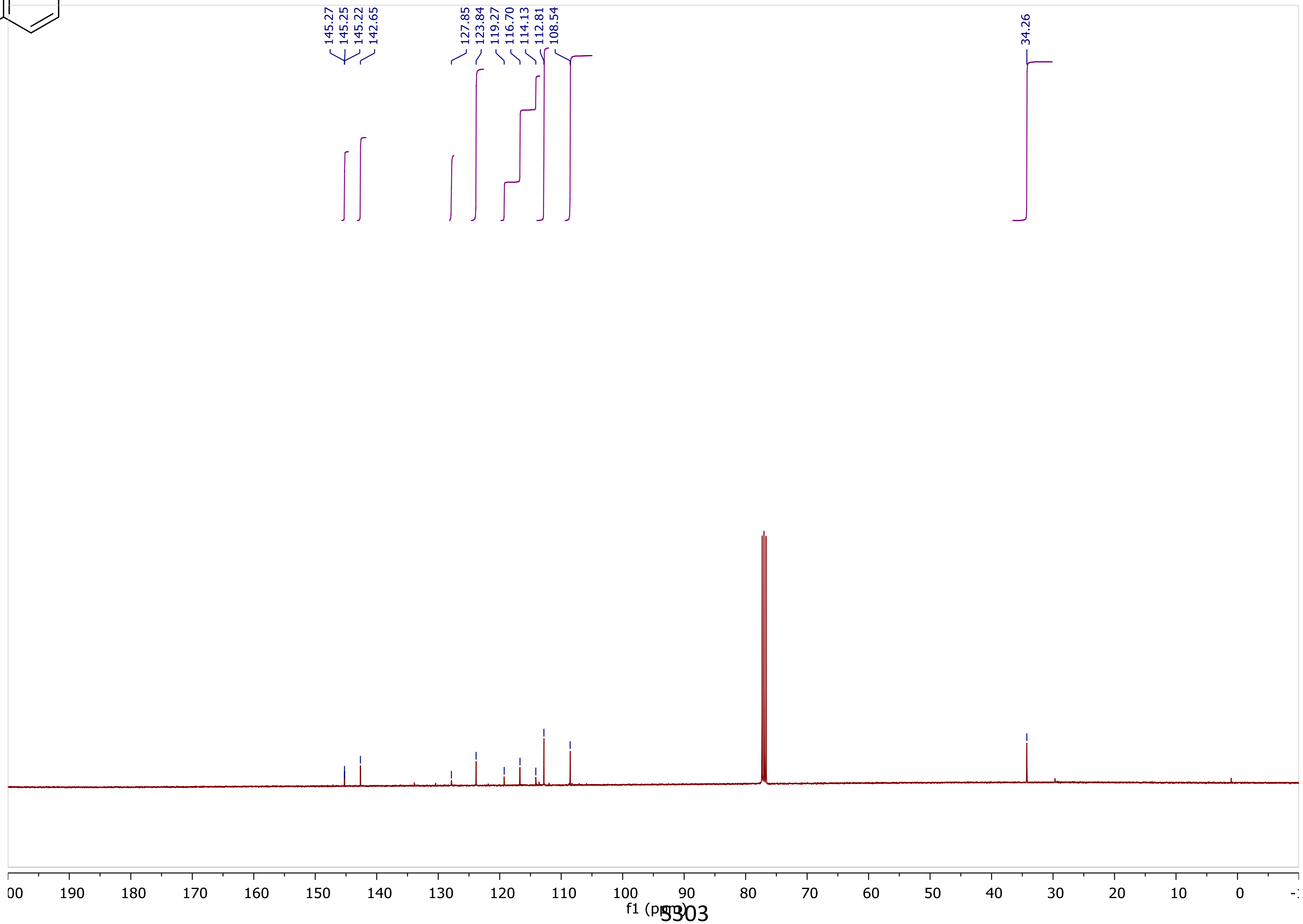

^{19}F NMR of 6-(difluoromethoxy)-*N*1-methylbenzene-1,2-diamine **5n-i** in $CDCl_3$

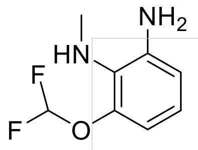

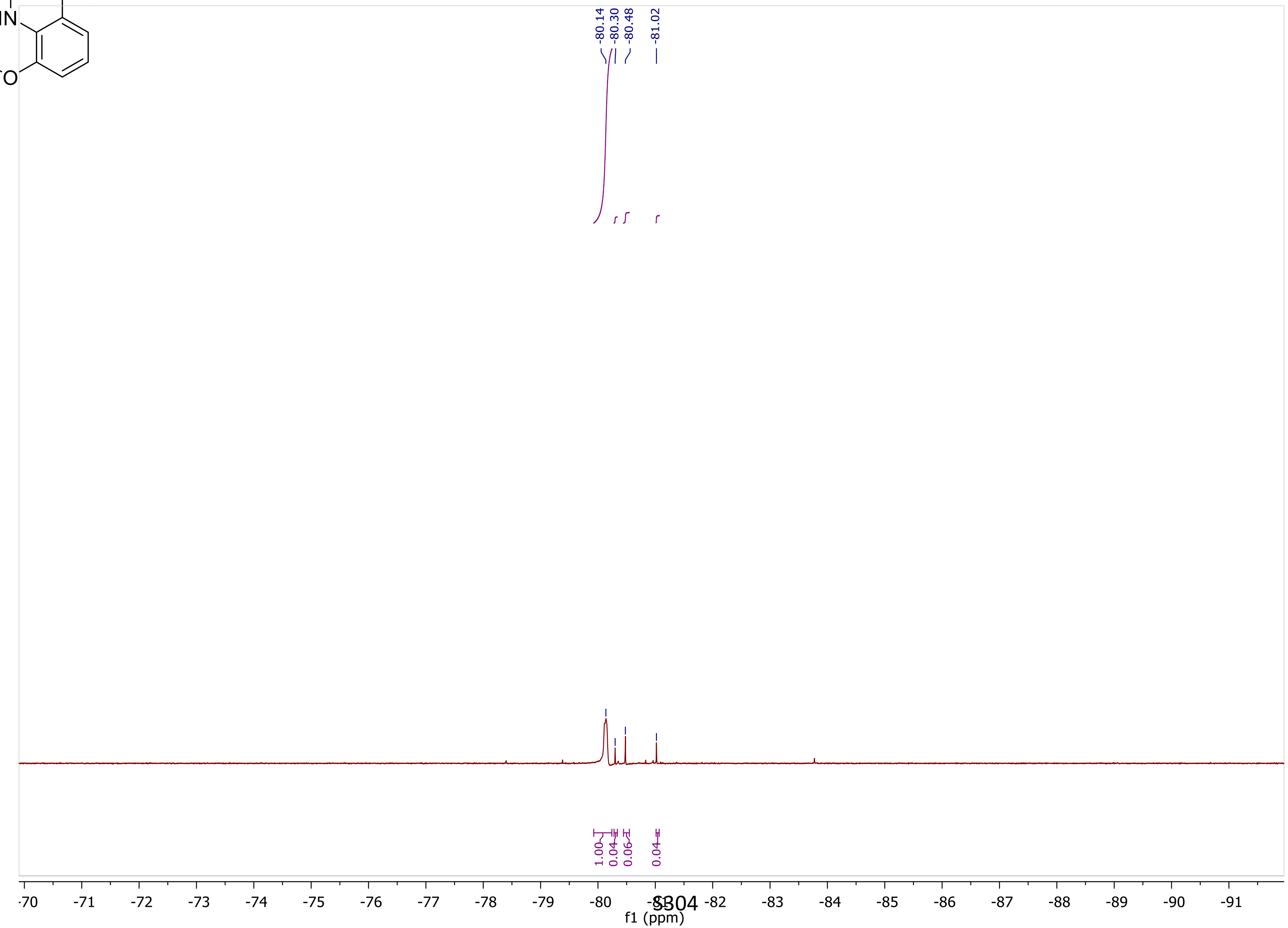

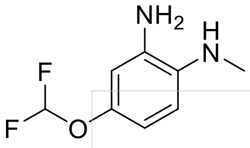

^{1}H NMR of 4-(difluoromethoxy)-*N*1-methylbenzene-1,2-diamine **5n-ii** in $CDCl_3$

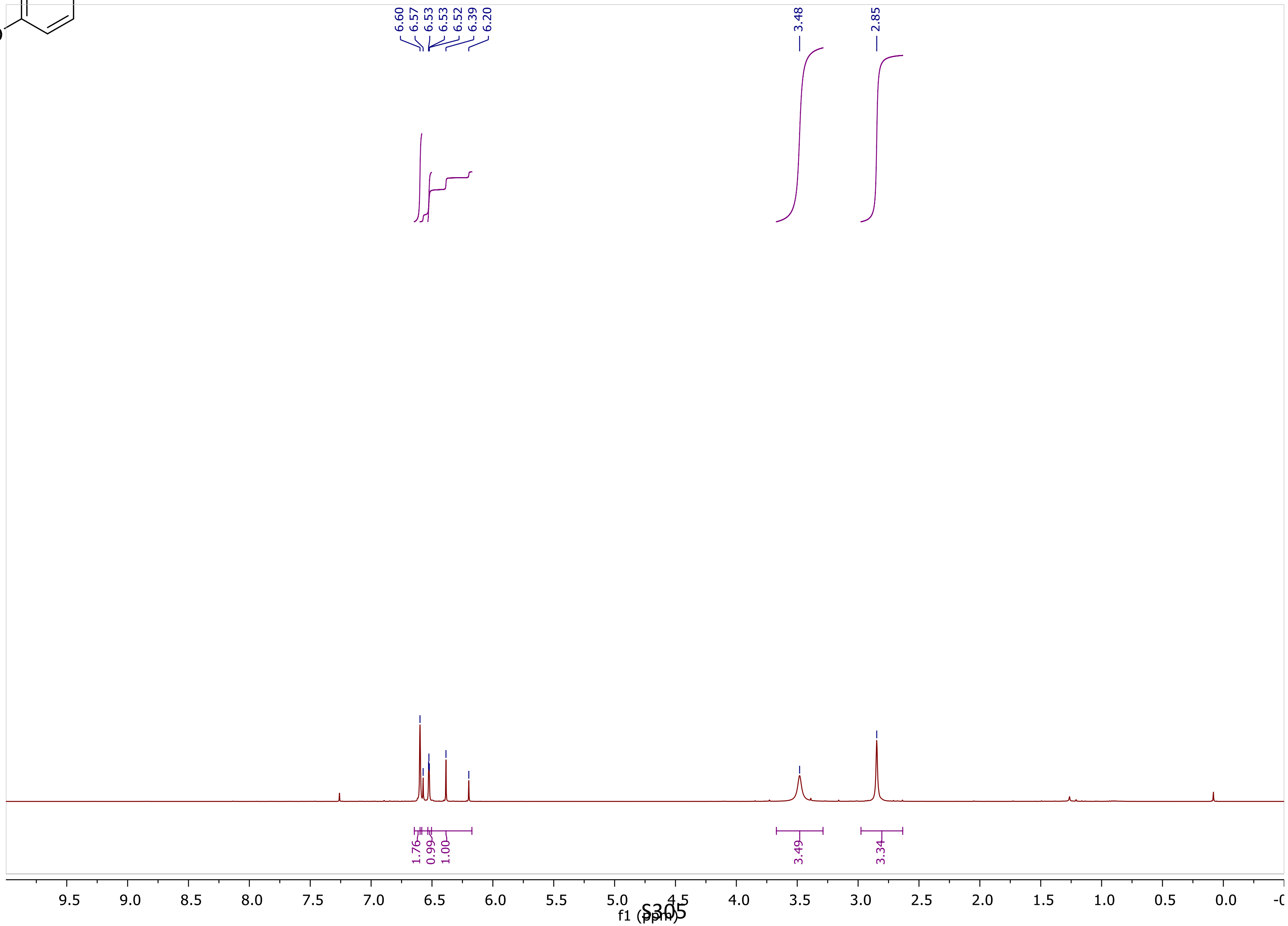

^{13}C NMR of 4-(difluoromethoxy)-*N*1-methylbenzene-1,2-diamine **5n-ii** in $CDCl_3$

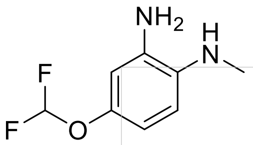

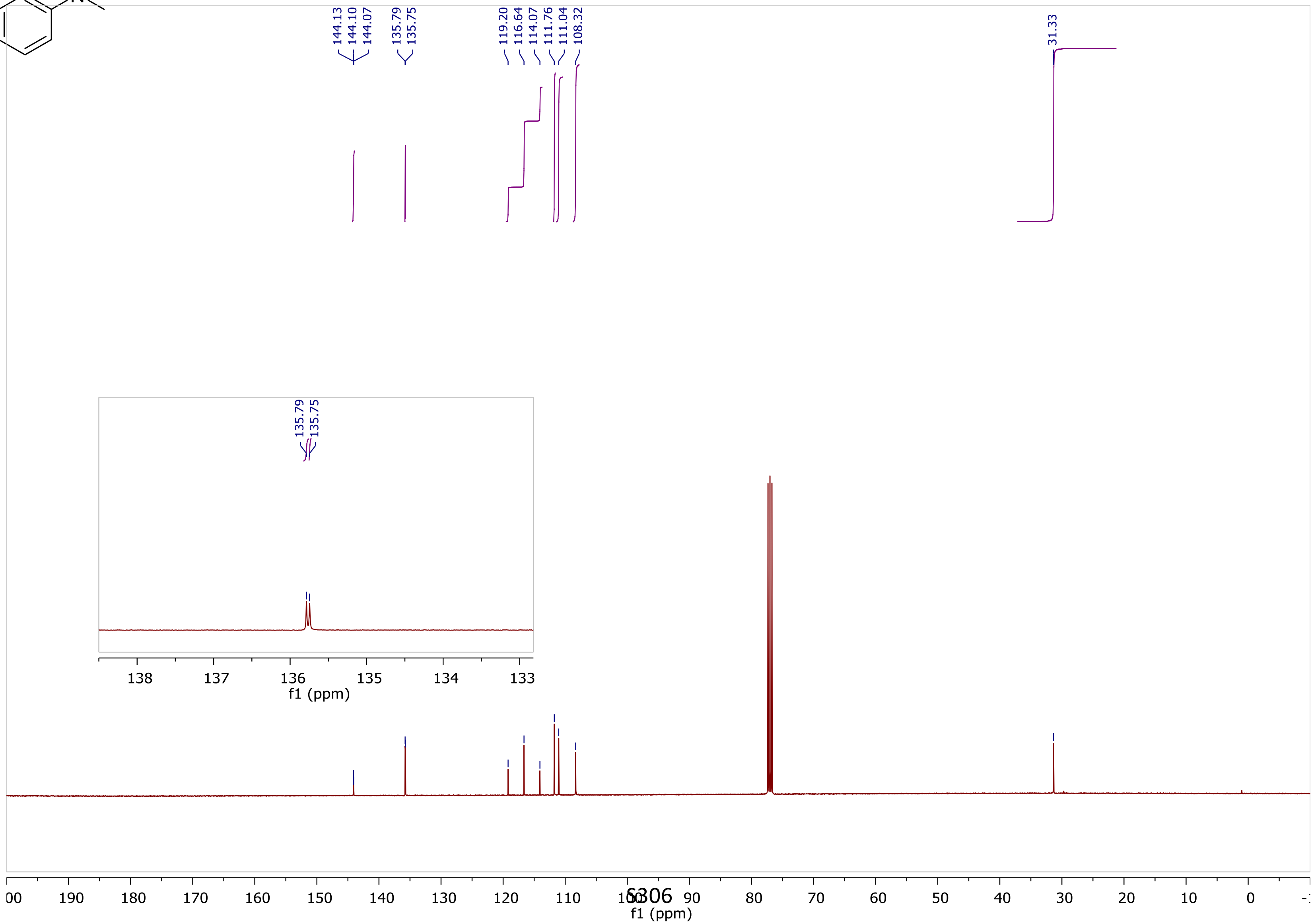

^{19}F NMR of 4-(difluoromethoxy)-*N*1-methylbenzene-1,2-diamine **5n-ii** in $CDCl_3$

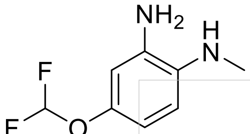

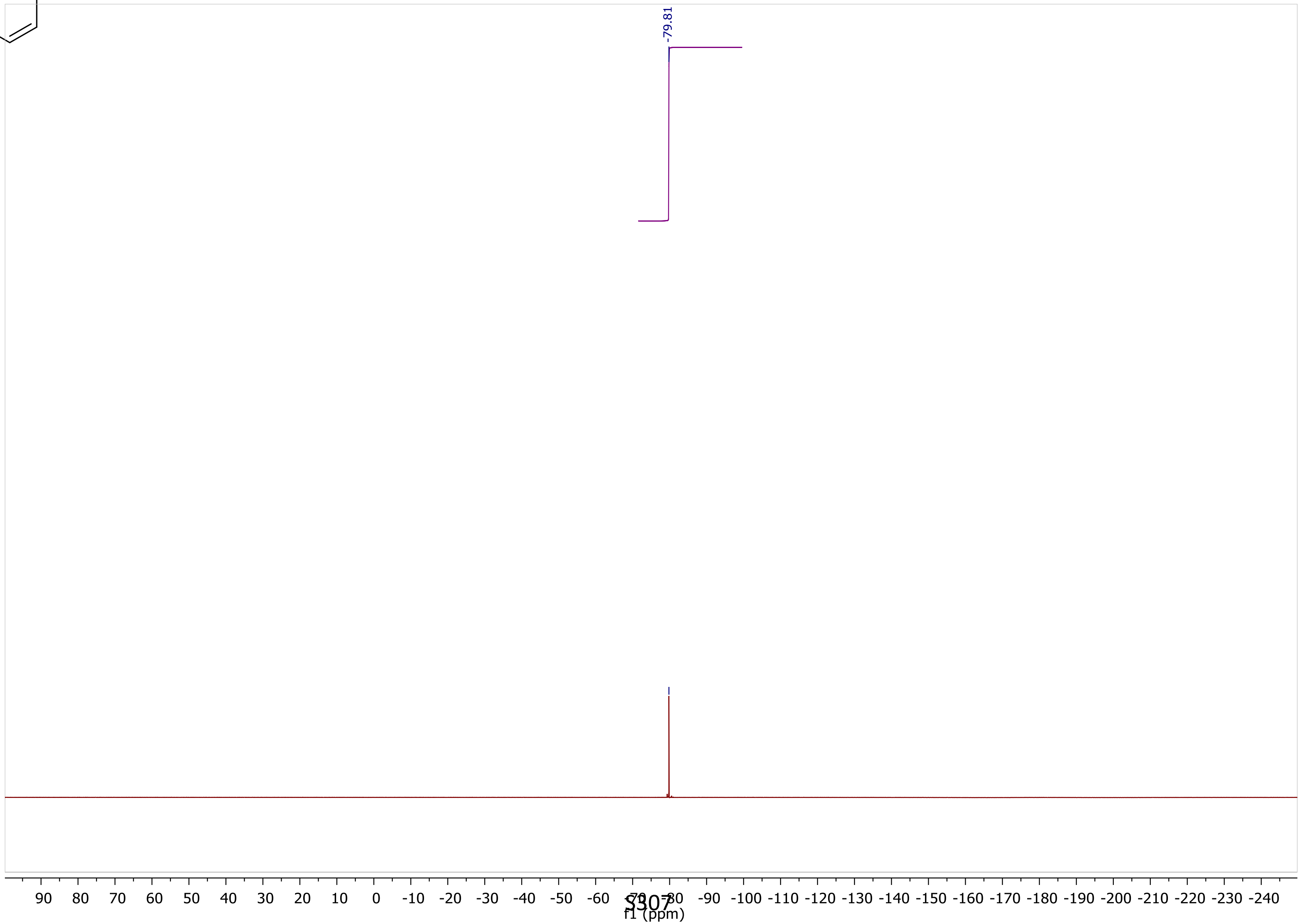

^{1}H NMR of 5-(difluoromethoxy)-1-methyl-1*H*-benzo[d]imidazole **5n-iii** in $CDCl_3$

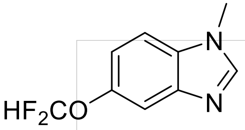

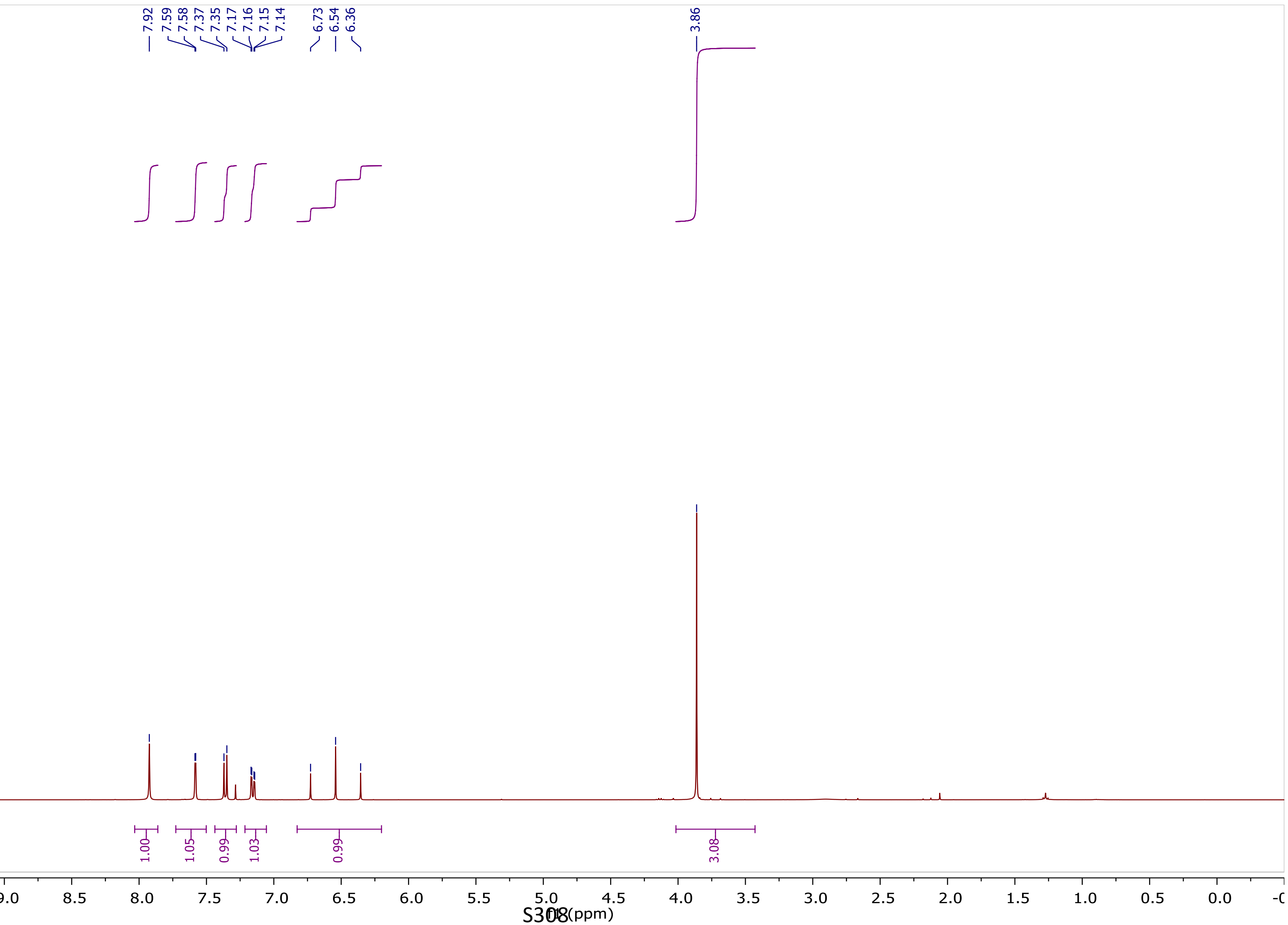

^{13}C NMR of 5-(difluoromethoxy)-1-methyl-1*H*-benzo[d]imidazole **5n-iii** in $CDCl_3$

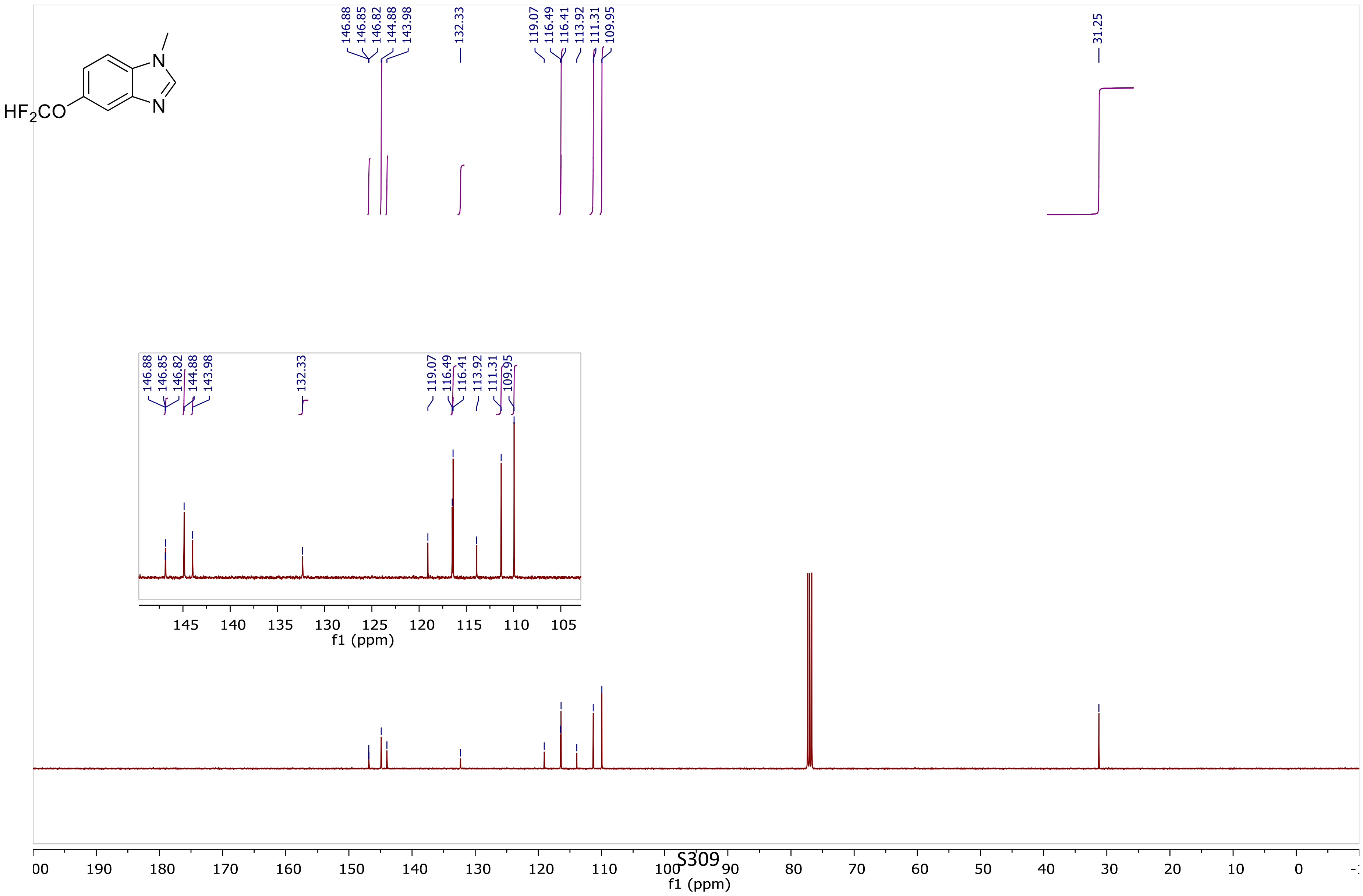

^{19}F NMR of 5-(difluoromethoxy)-1-methyl-1*H*-benzo[d]imidazole **5n-iii** in $CDCl_3$

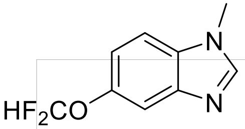

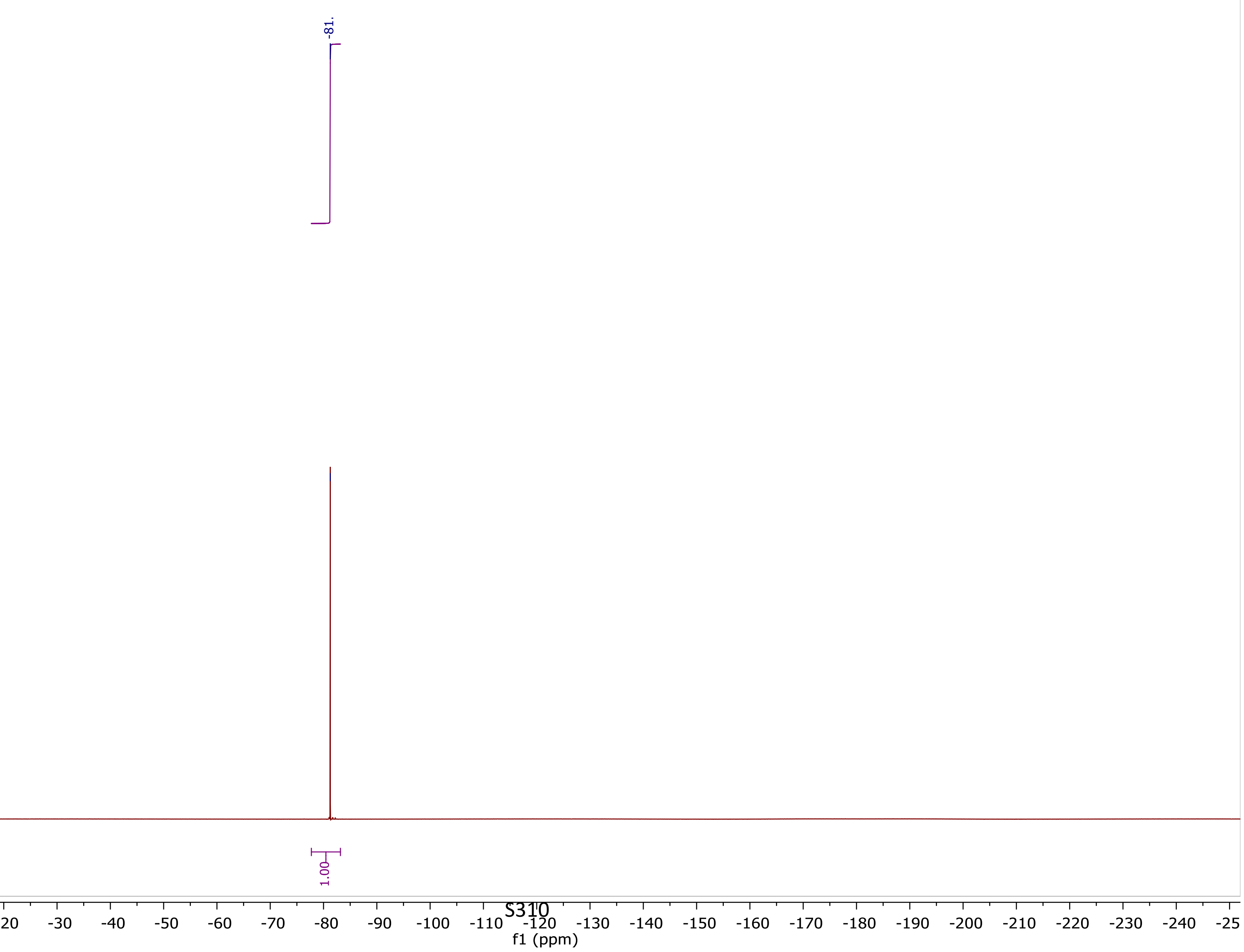

^1H NMR of 6-bromo-*N*1,3-dimethylbenzene-1,2-diamine **5o** in $CDCl_3$

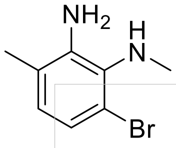

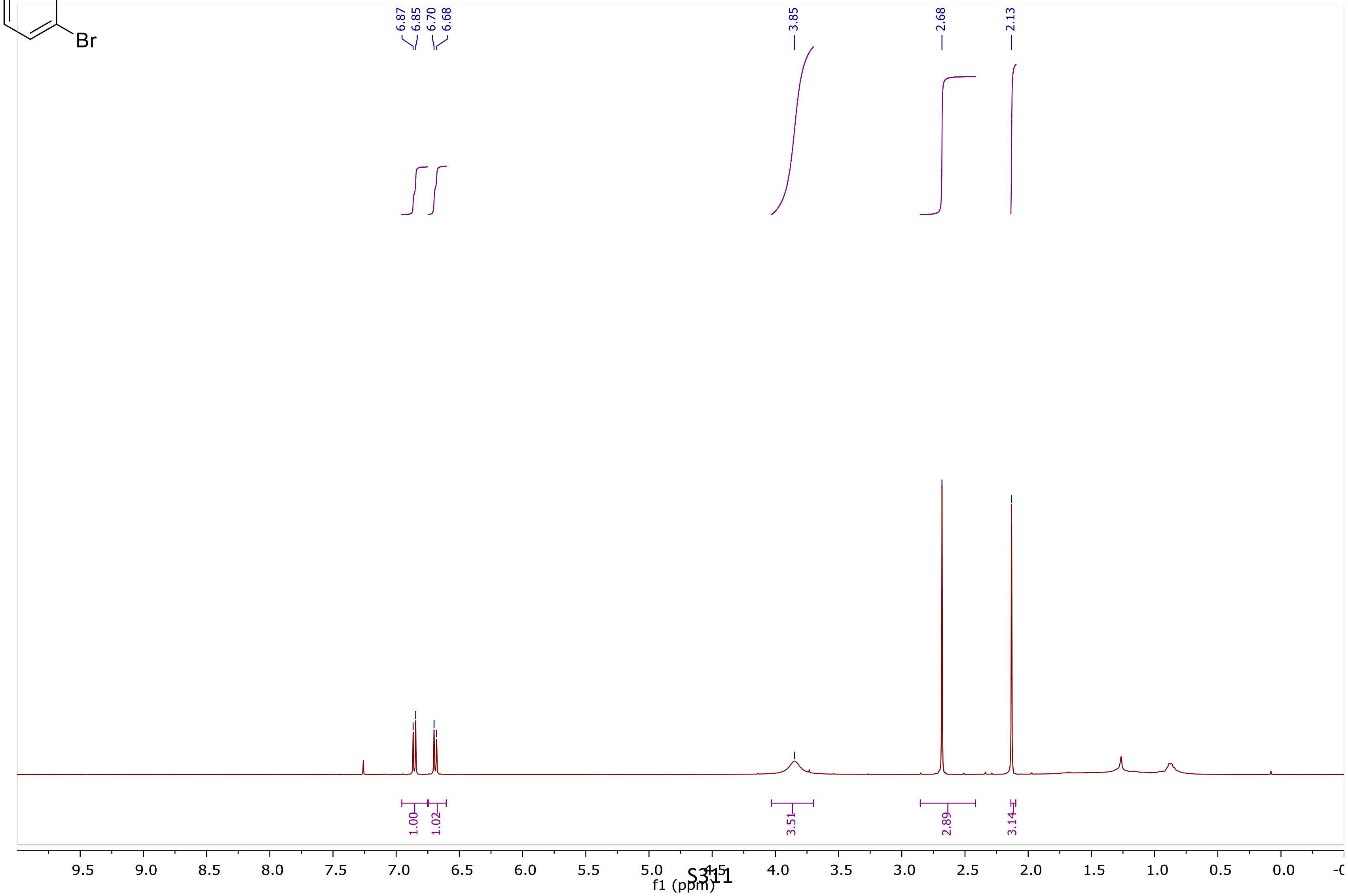

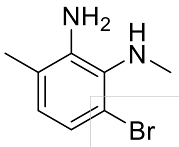

^{13}C NMR of 6-bromo-*N*1,3-dimethylbenzene-1,2-diamine **5o** in $CDCl_3$

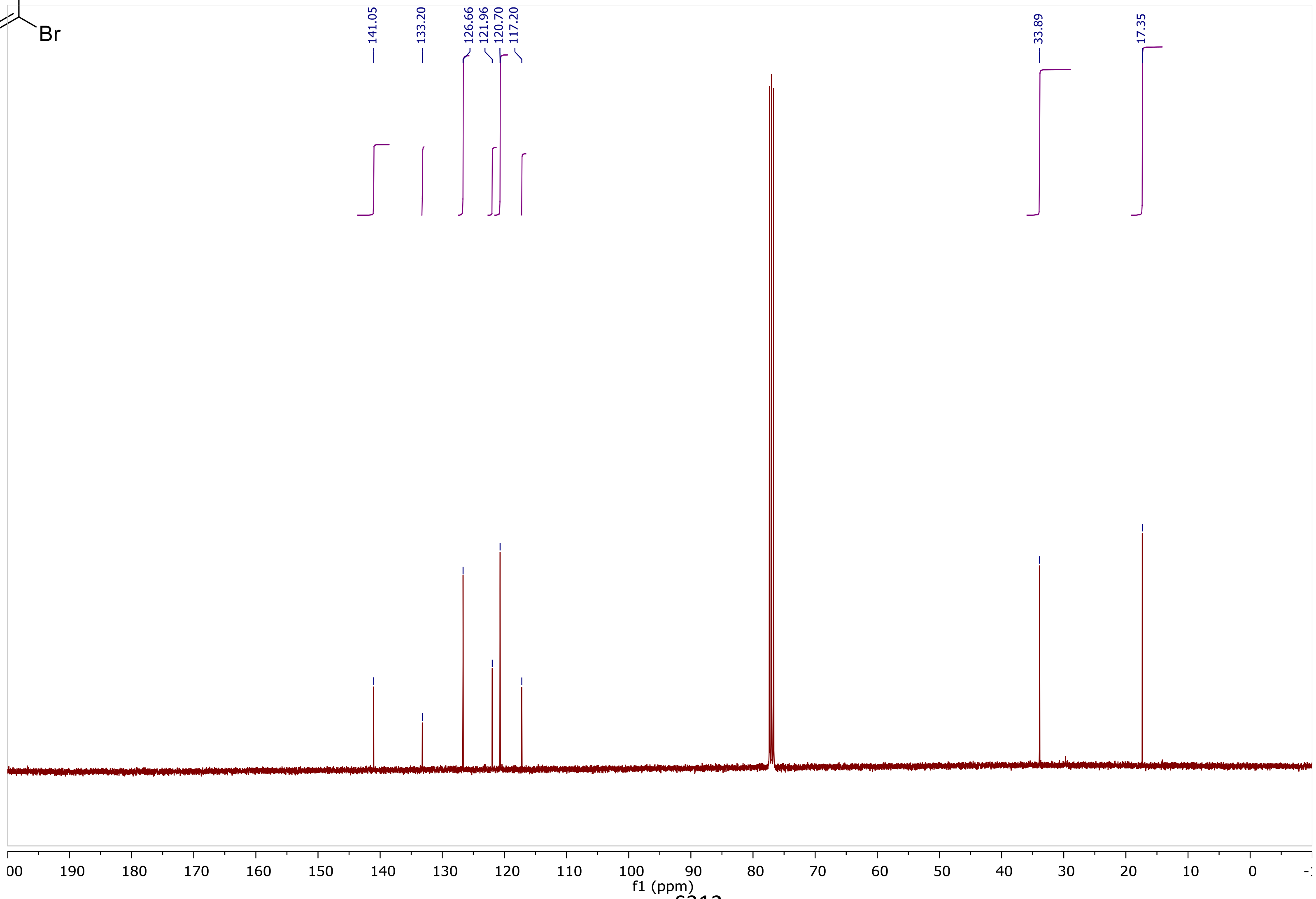

^{1}H NMR of 4-chloro-*N*1,3-dimethylbenzene-1,2-diamine **5p-i** in $CDCl_3$

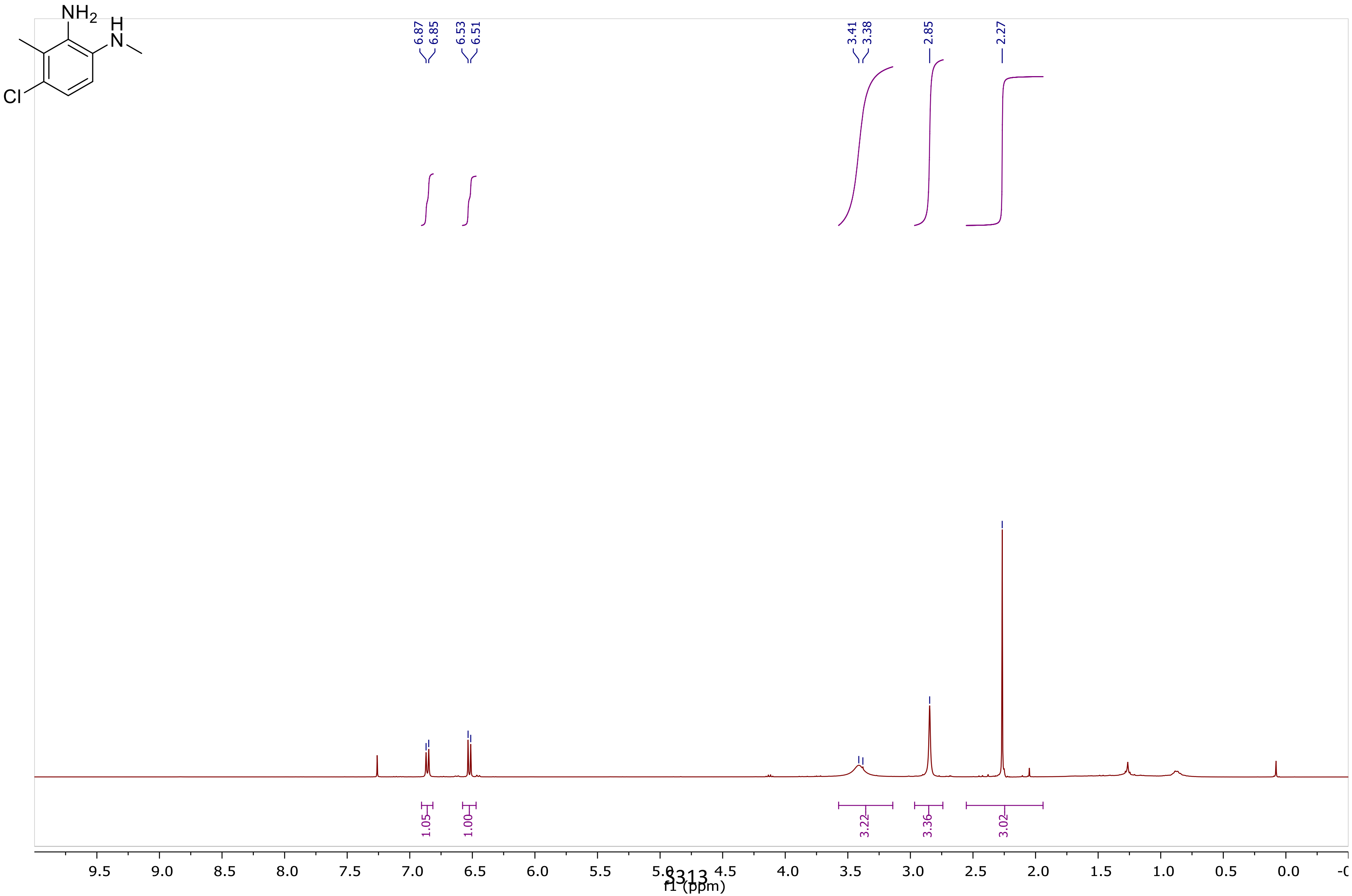

^{13}C NMR of 4-chloro-*N*1,3-dimethylbenzene-1,2-diamine **5p-i** in $CDCl_3$

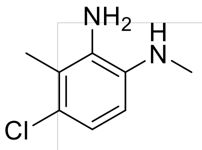

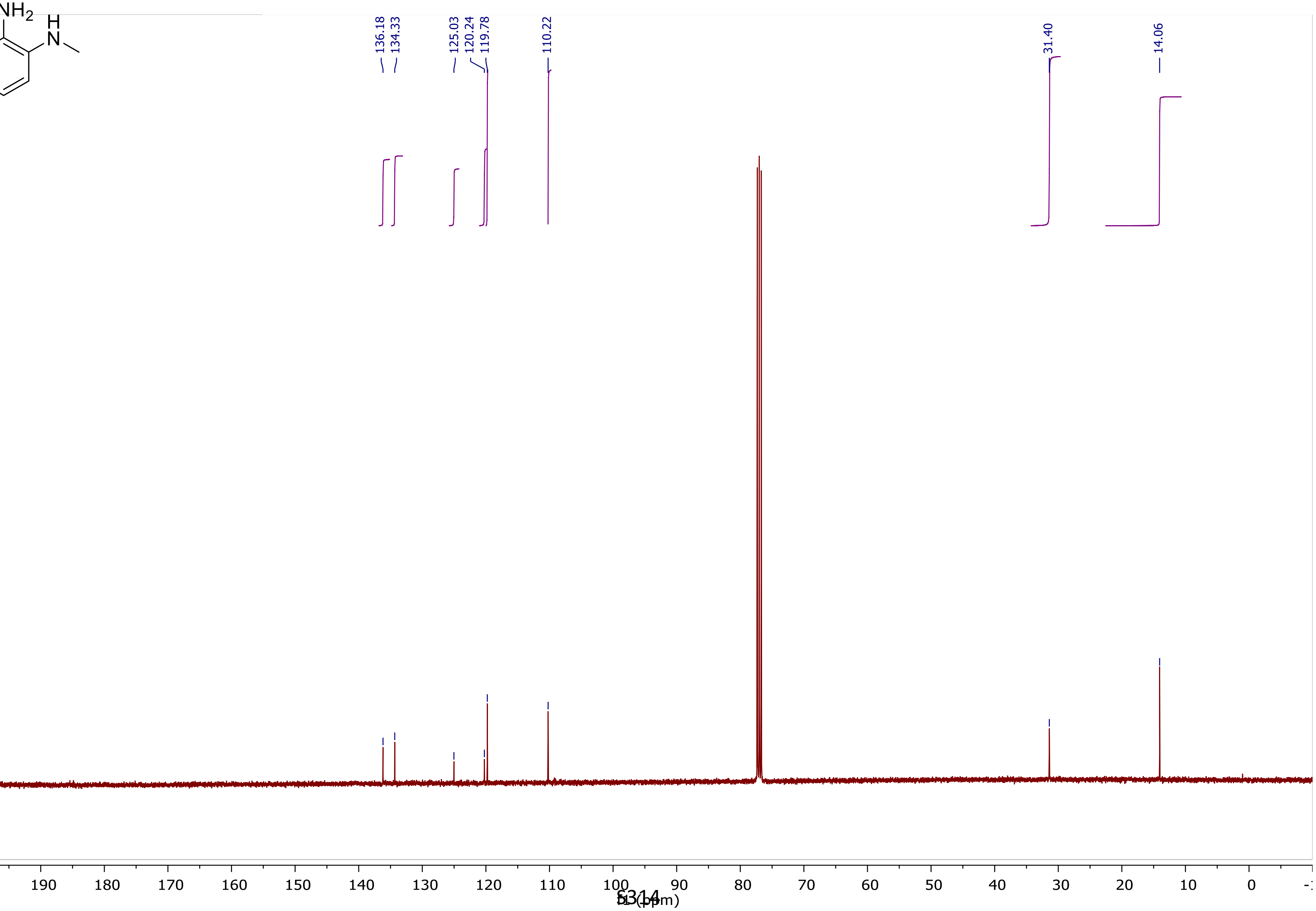

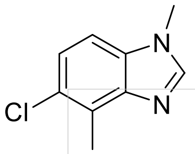

^{1}H NMR of 5-chloro-1,4-dimethyl-1*H*-benzo[d]imidazole **5p-ii** in $CDCl_3$

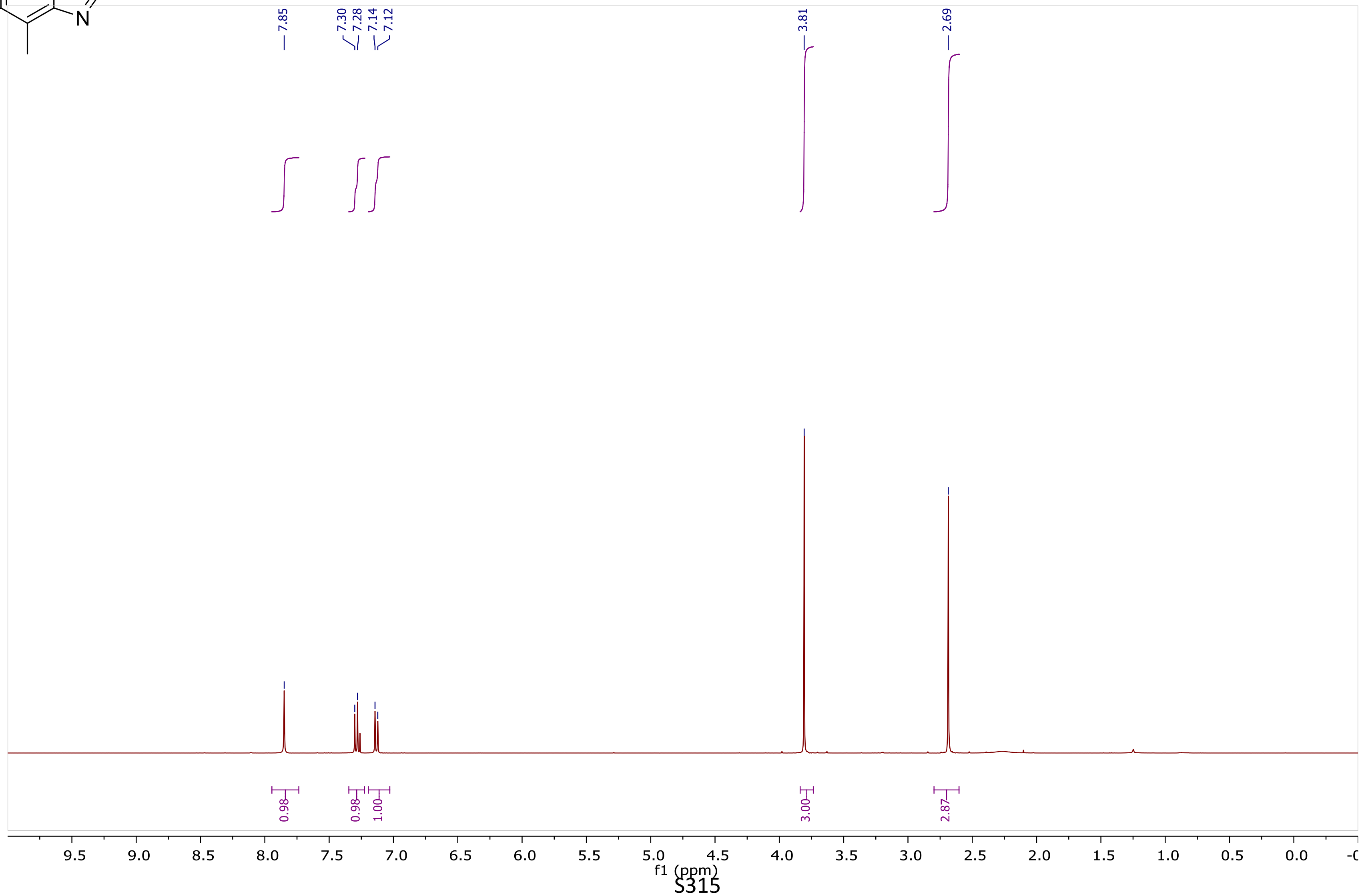

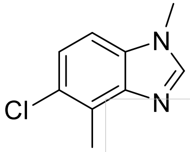

^{13}C NMR of 5-chloro-1,4-dimethyl-1*H*-benzo[d]imidazole **5p-ii** in $CDCl_3$

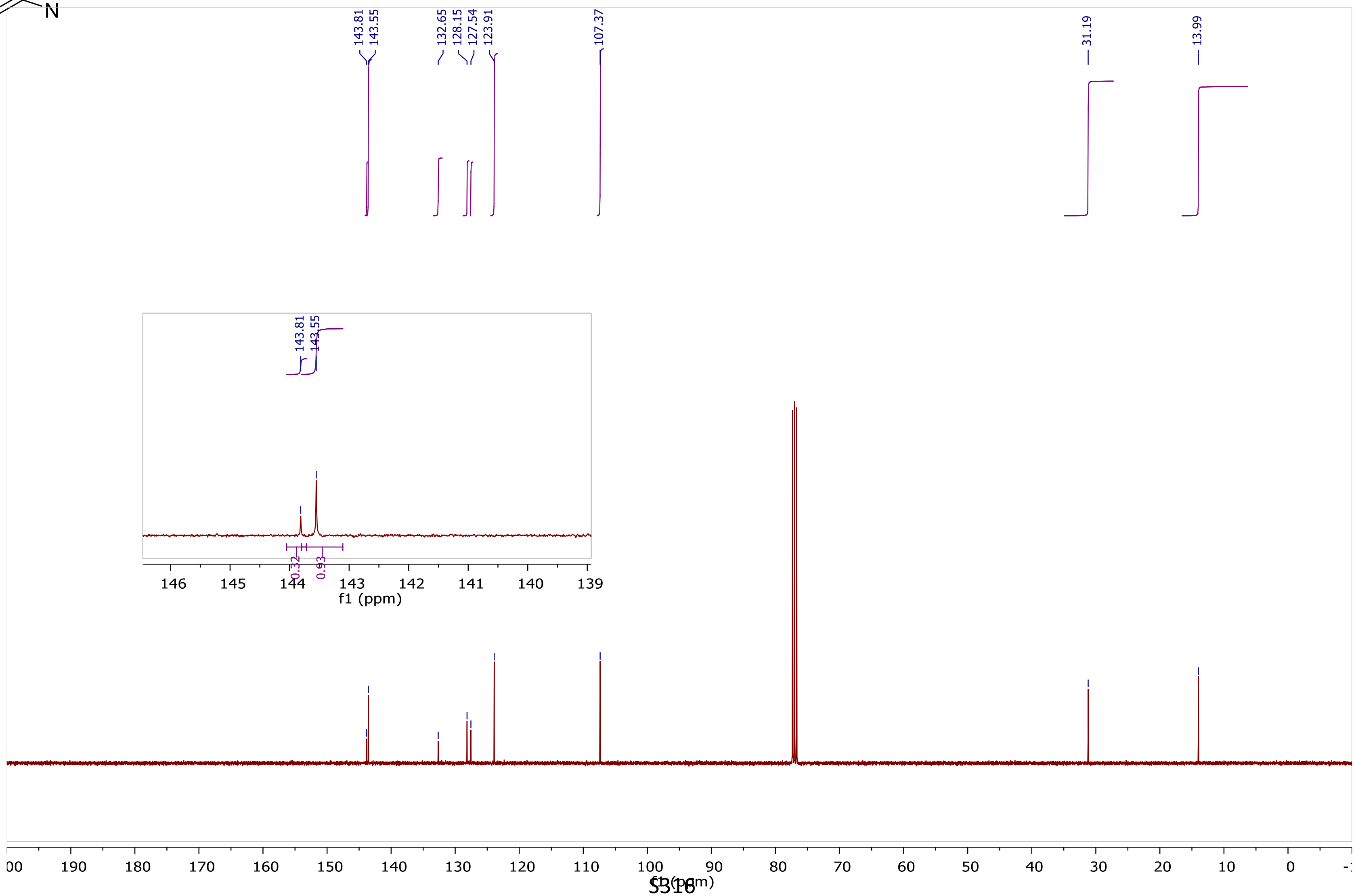

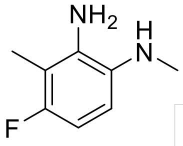

^{1}H NMR of 4-fluoro-N1,3-dimethylbenzene-1,2-diamine **5q** in MeOD-d[4]

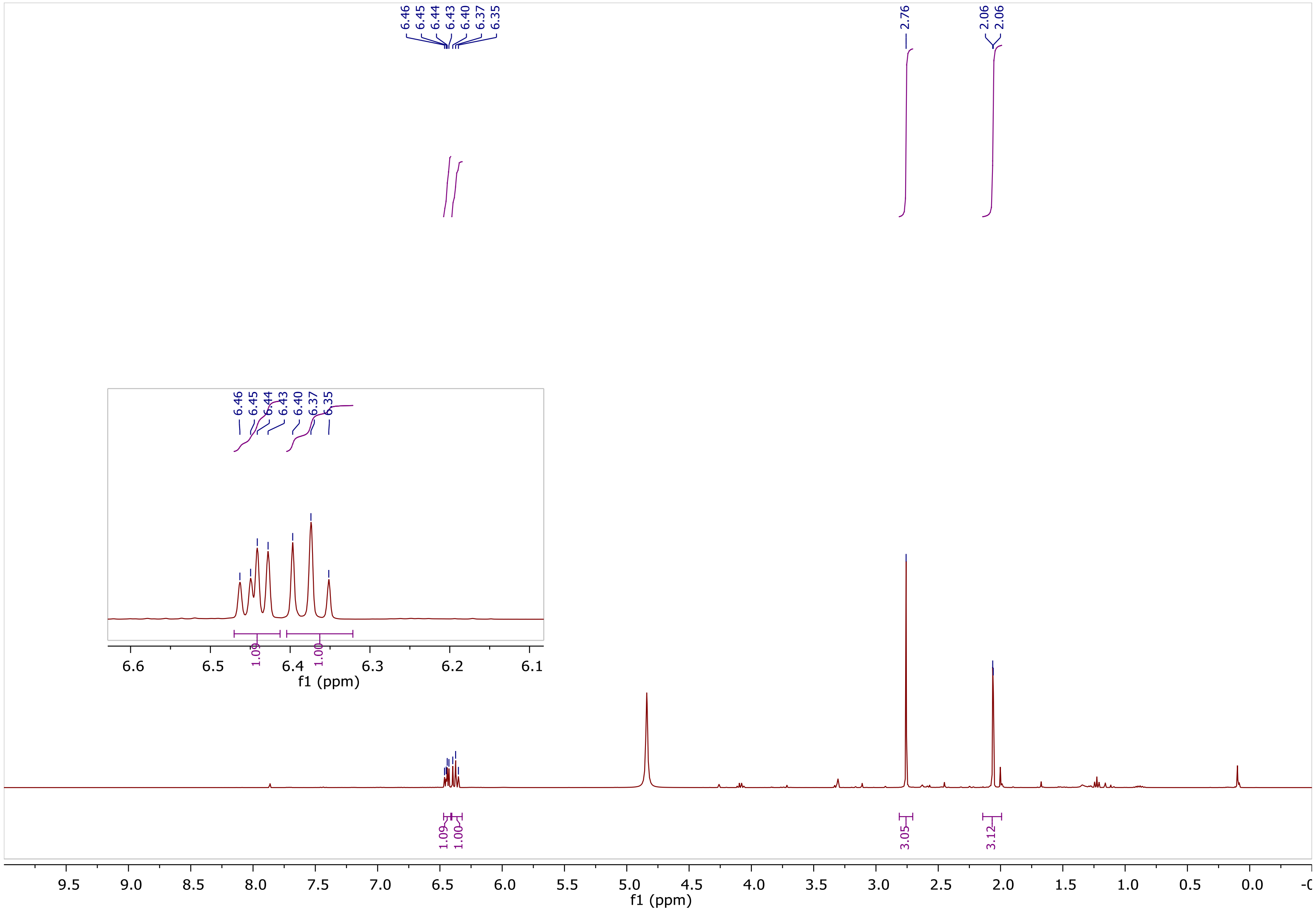

^{13}C NMR of 4-fluoro-N1,3-dimethylbenzene-1,2-diamine **5q** in MeOD-d[4]

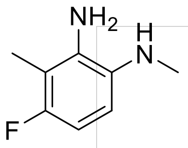

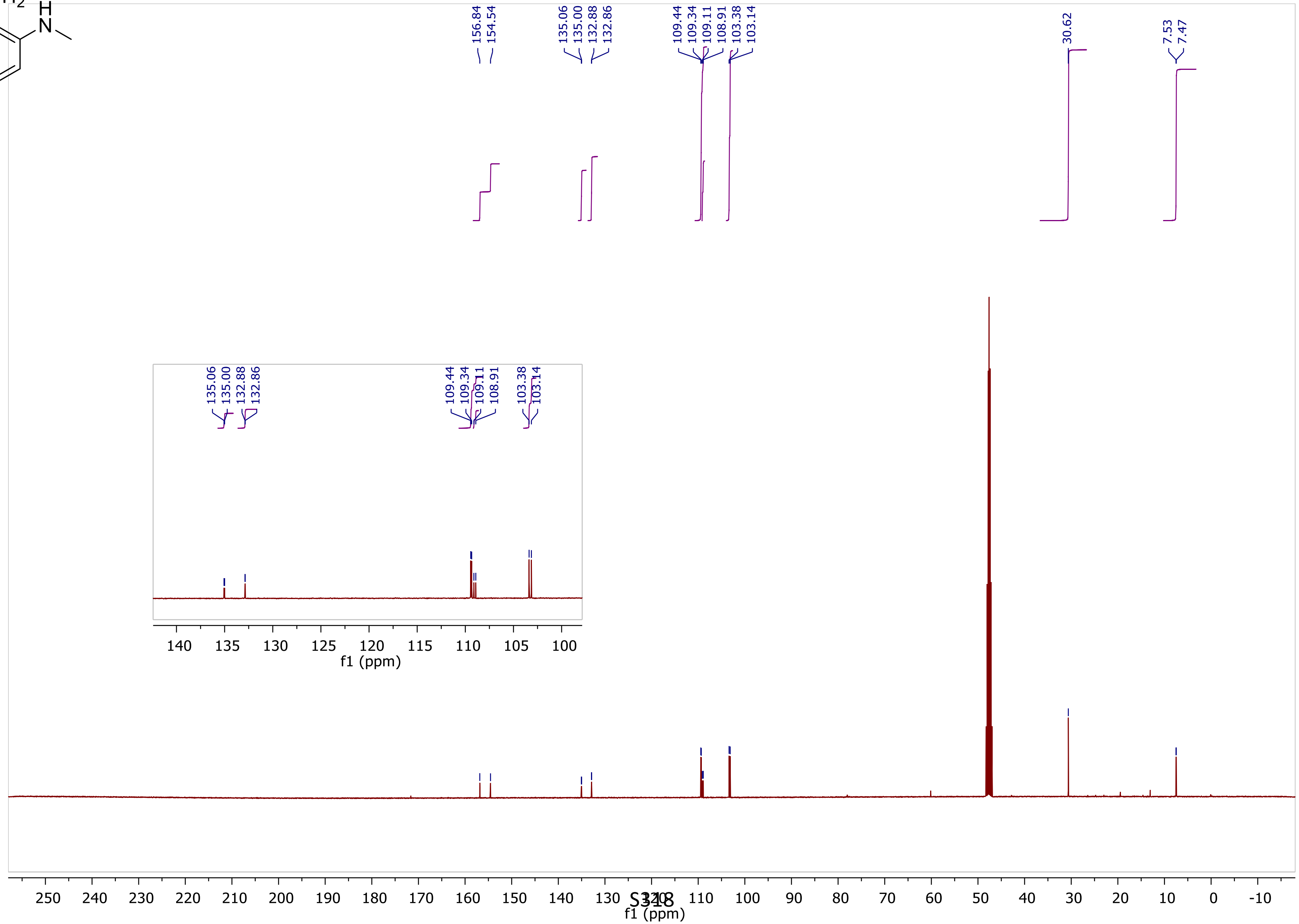

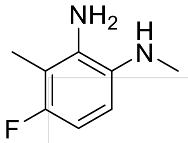

^{19}F NMR of 4-fluoro-N1,3-dimethylbenzene-1,2-diamine **5q** in MeOD-d[4]

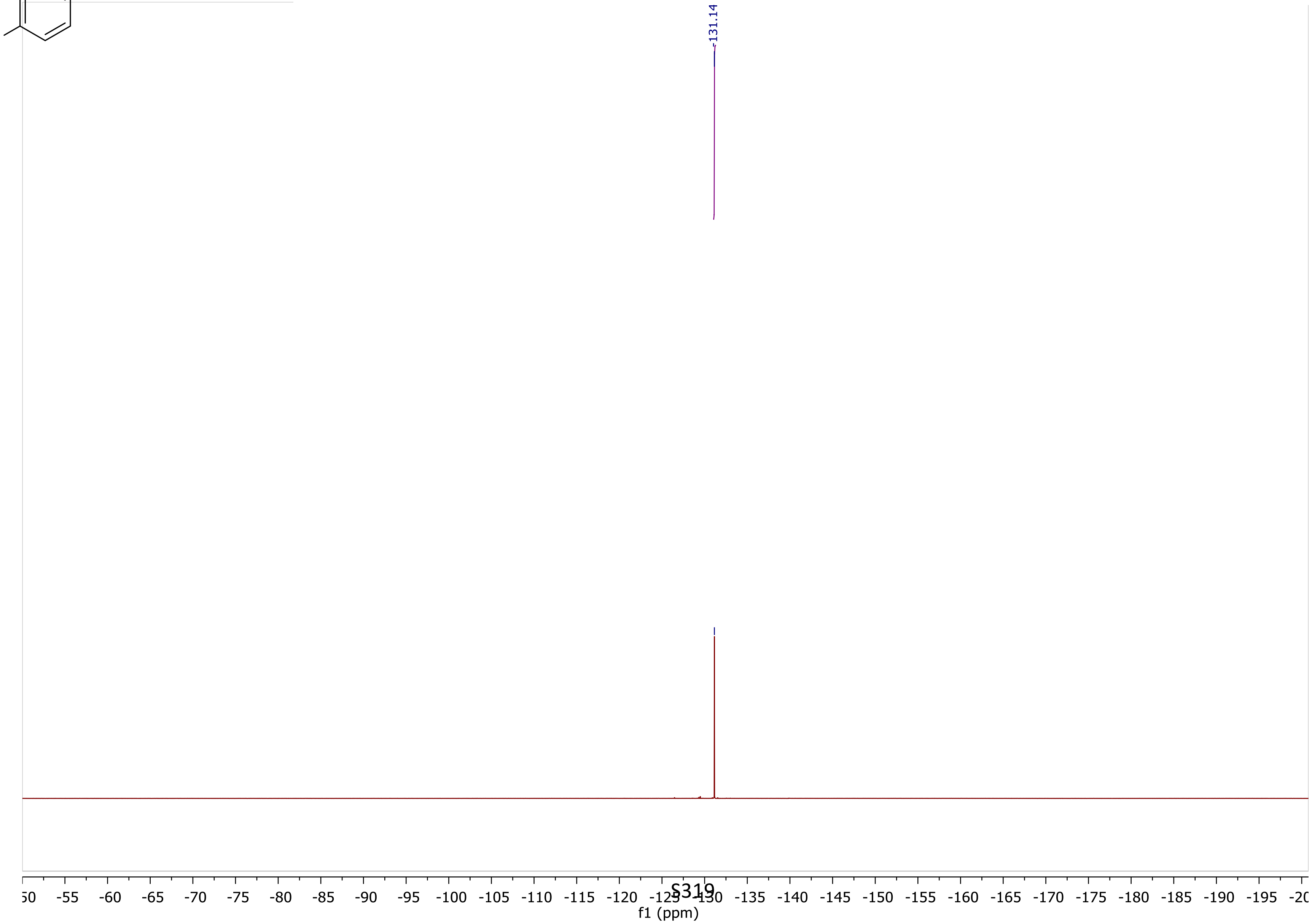

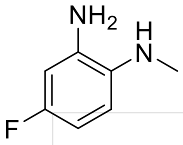

^{1}H NMR of 4-fluoro-*N*1-methylbenzene-1,2-diamine **5r** in $CDCl_3$

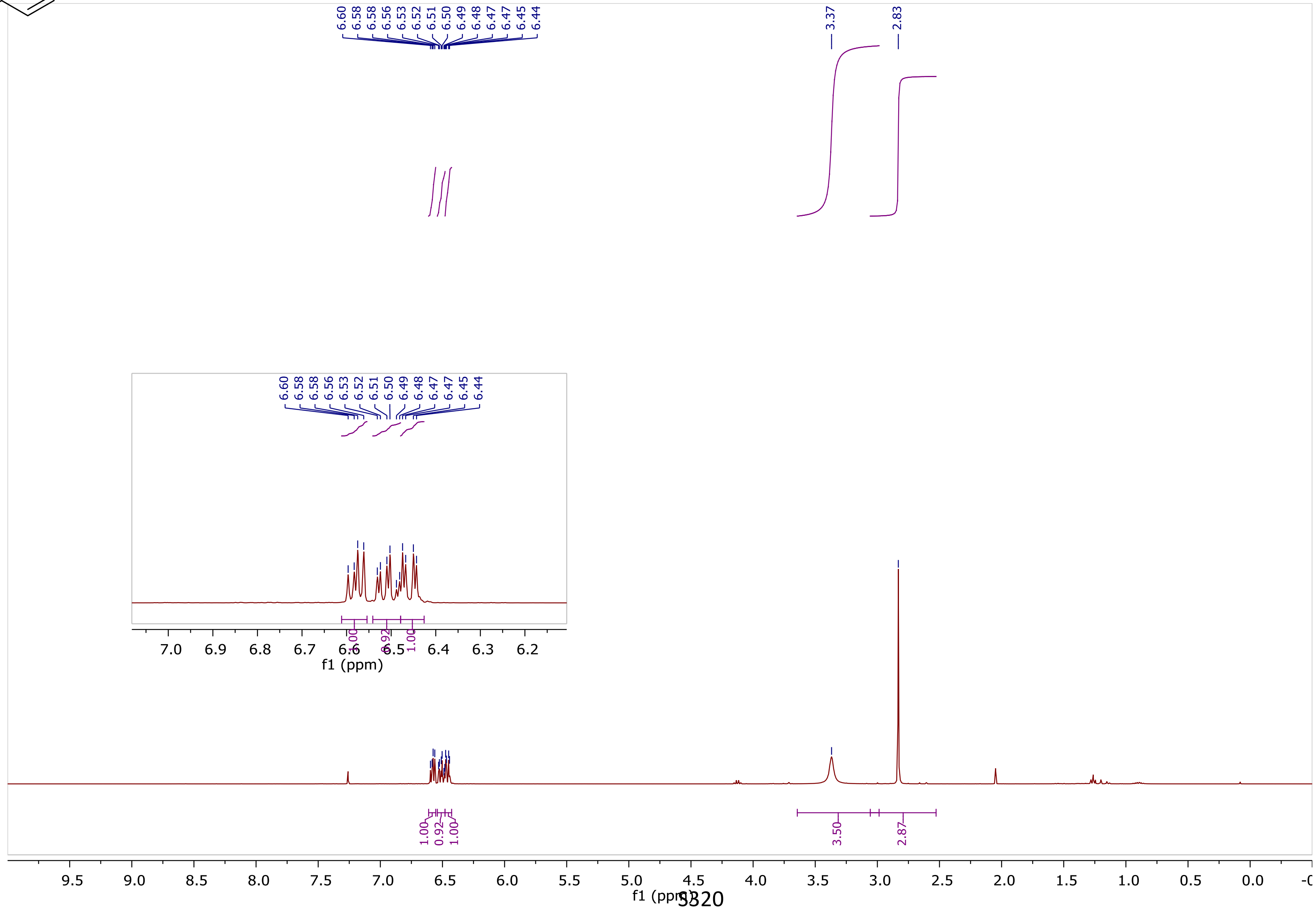

^{13}C NMR of 4-fluoro-*N*1-methylbenzene-1,2-diamine **5r** in $CDCl_3$

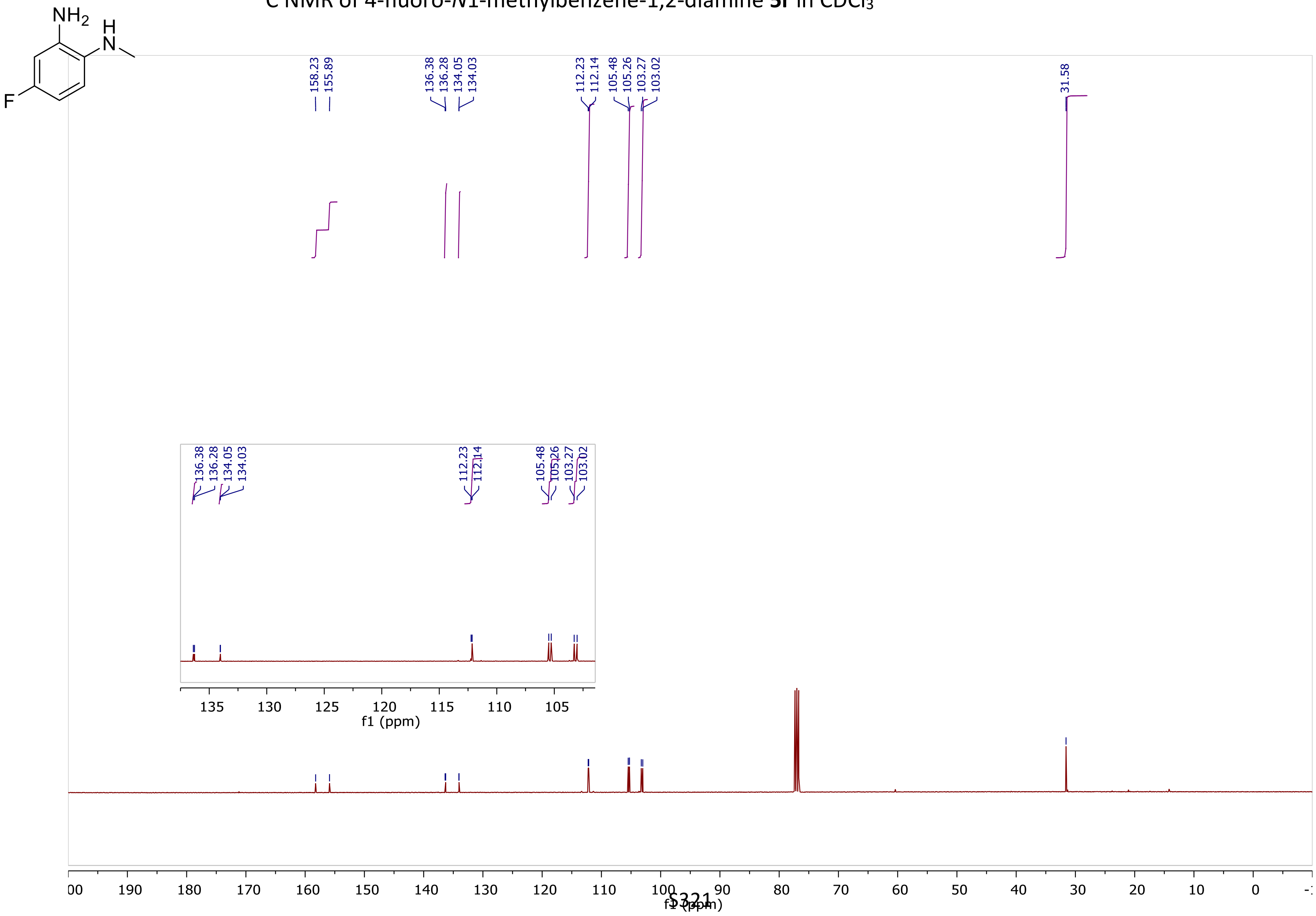

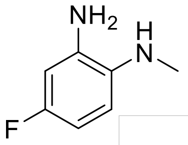

^{19}F NMR of 4-fluoro-*N*1-methylbenzene-1,2-diamine **5r** in $CDCl_3$

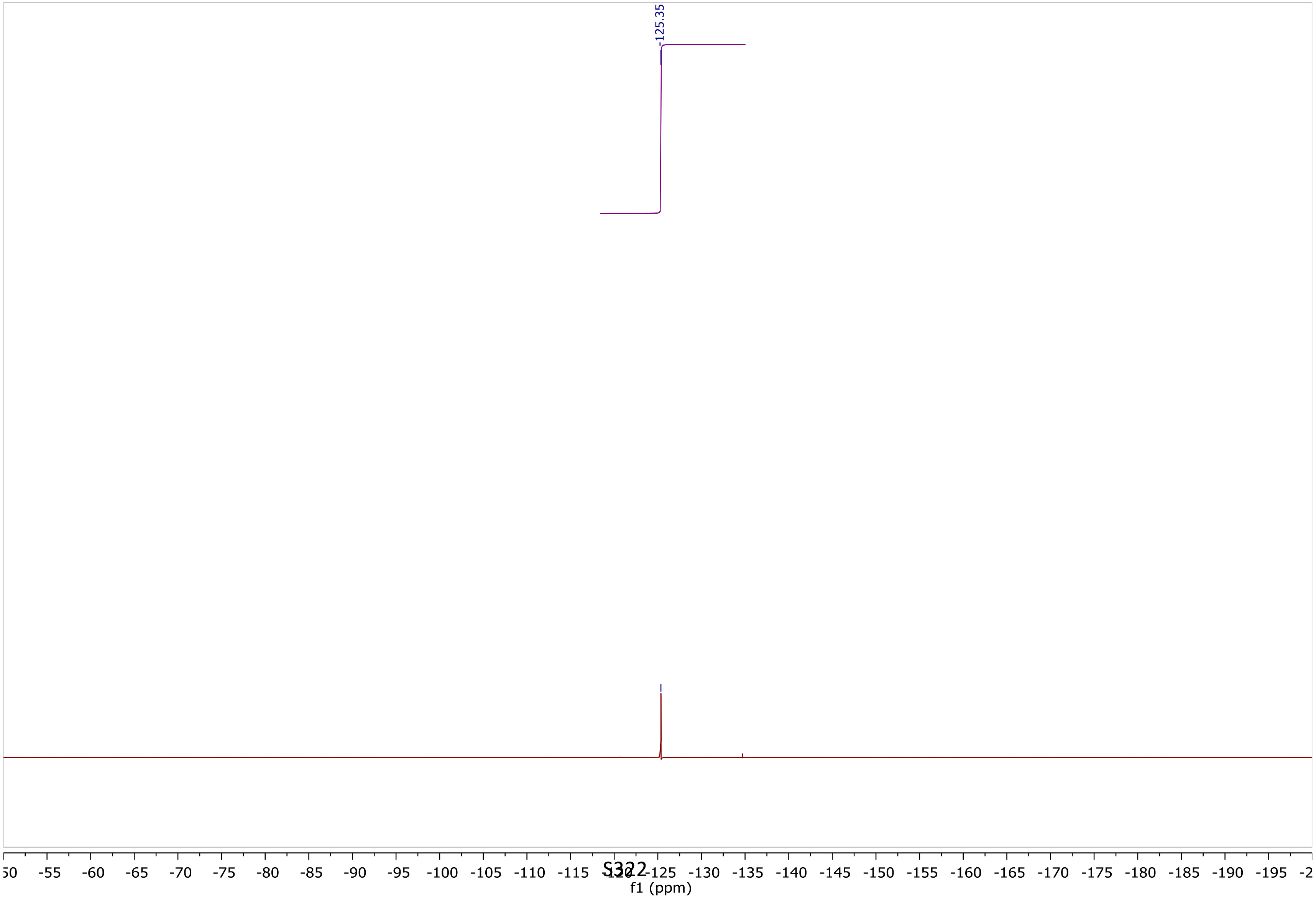

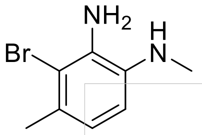

^{1}H NMR of 4-bromo-*N*1,3-dimethylbenzene-1,2-diamine **5s** in $CDCl_3$

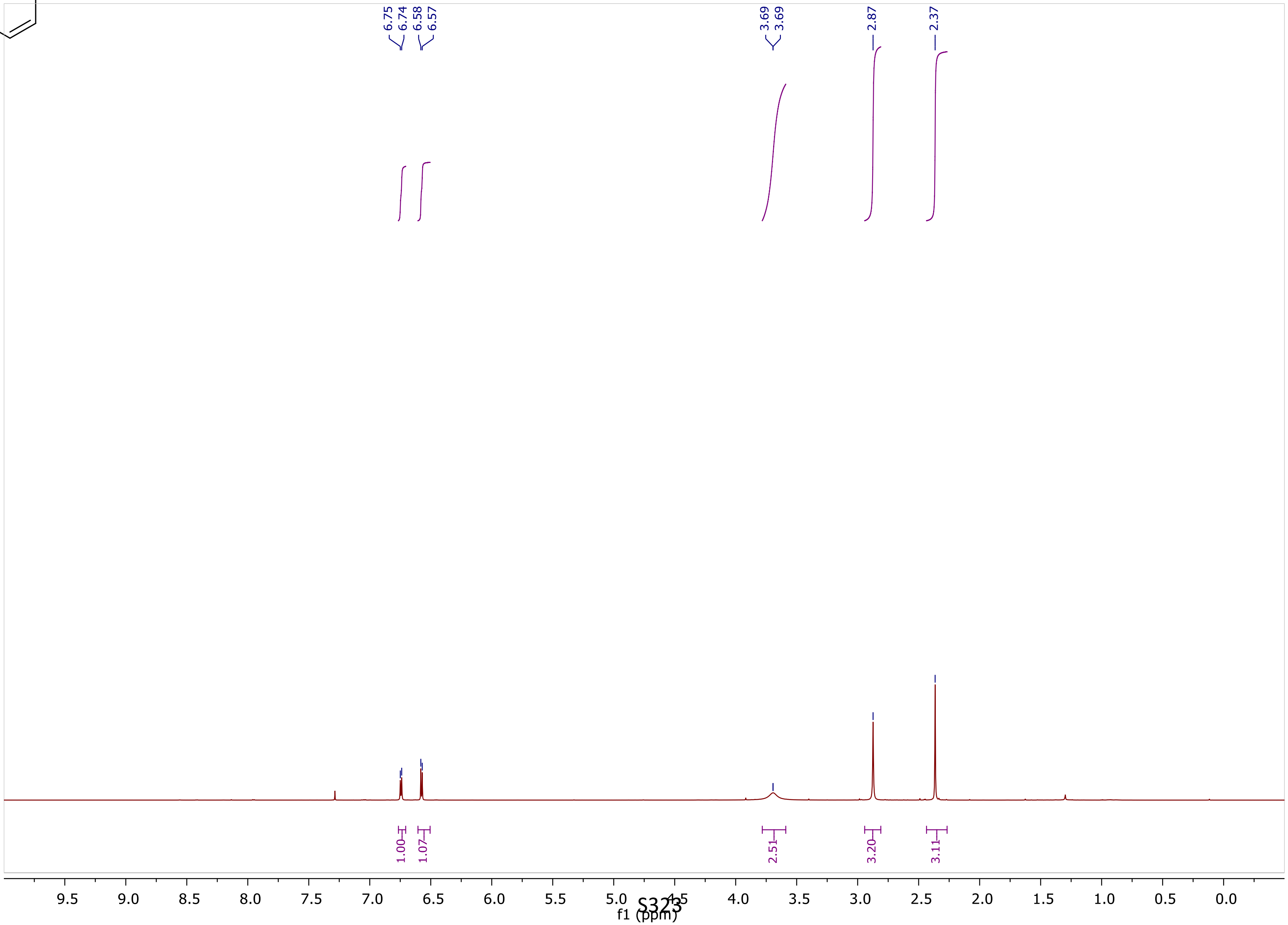

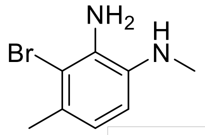

^{13}C NMR of 4-bromo-*N*1,3-dimethylbenzene-1,2-diamine **5s** in $CDCl_3$

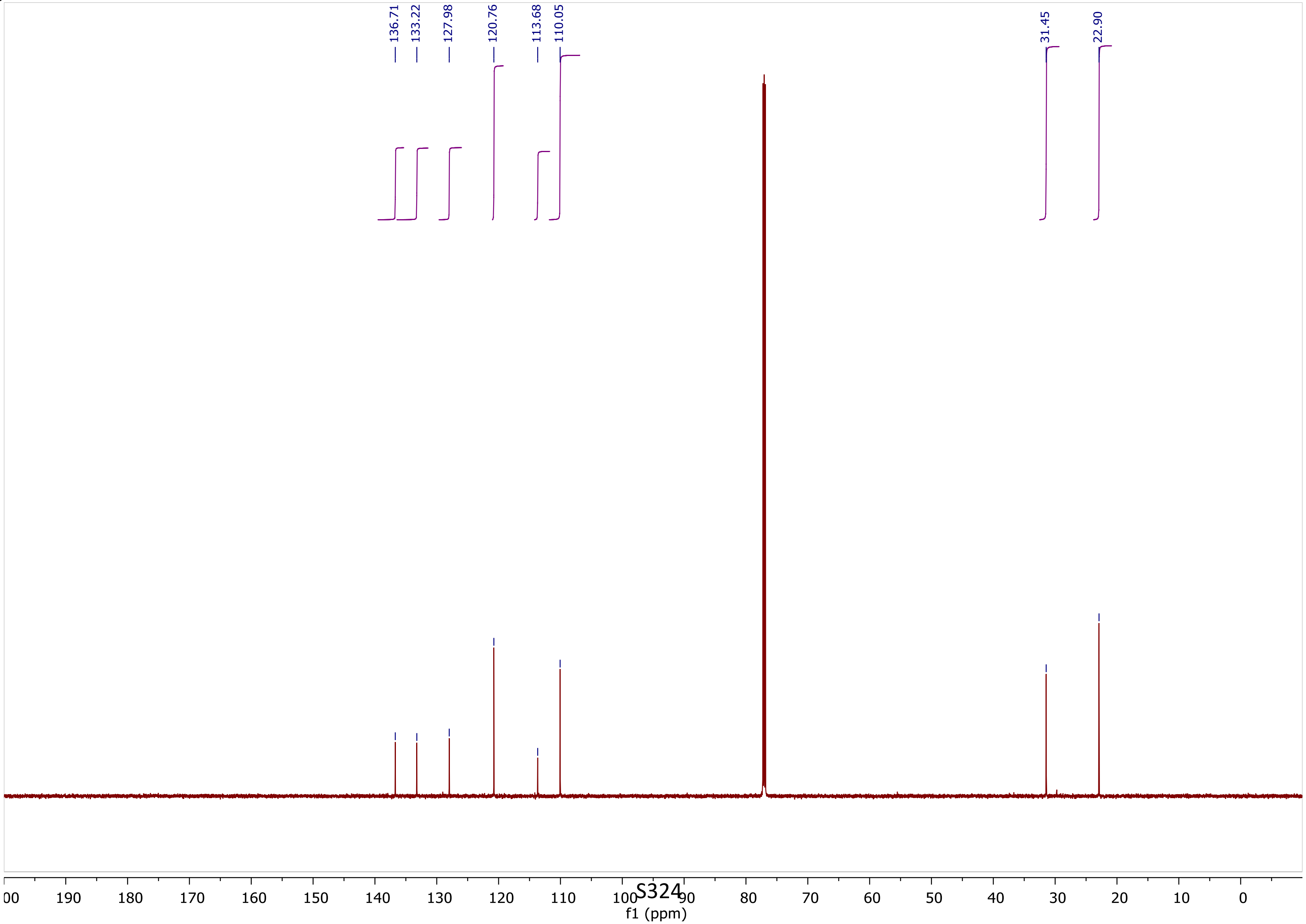

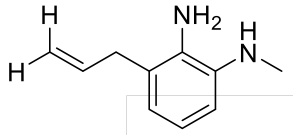

^{1}H NMR of 3-allyl-*N*1-methylbenzene-1,2-diamine **5t** in $CDCl_3$

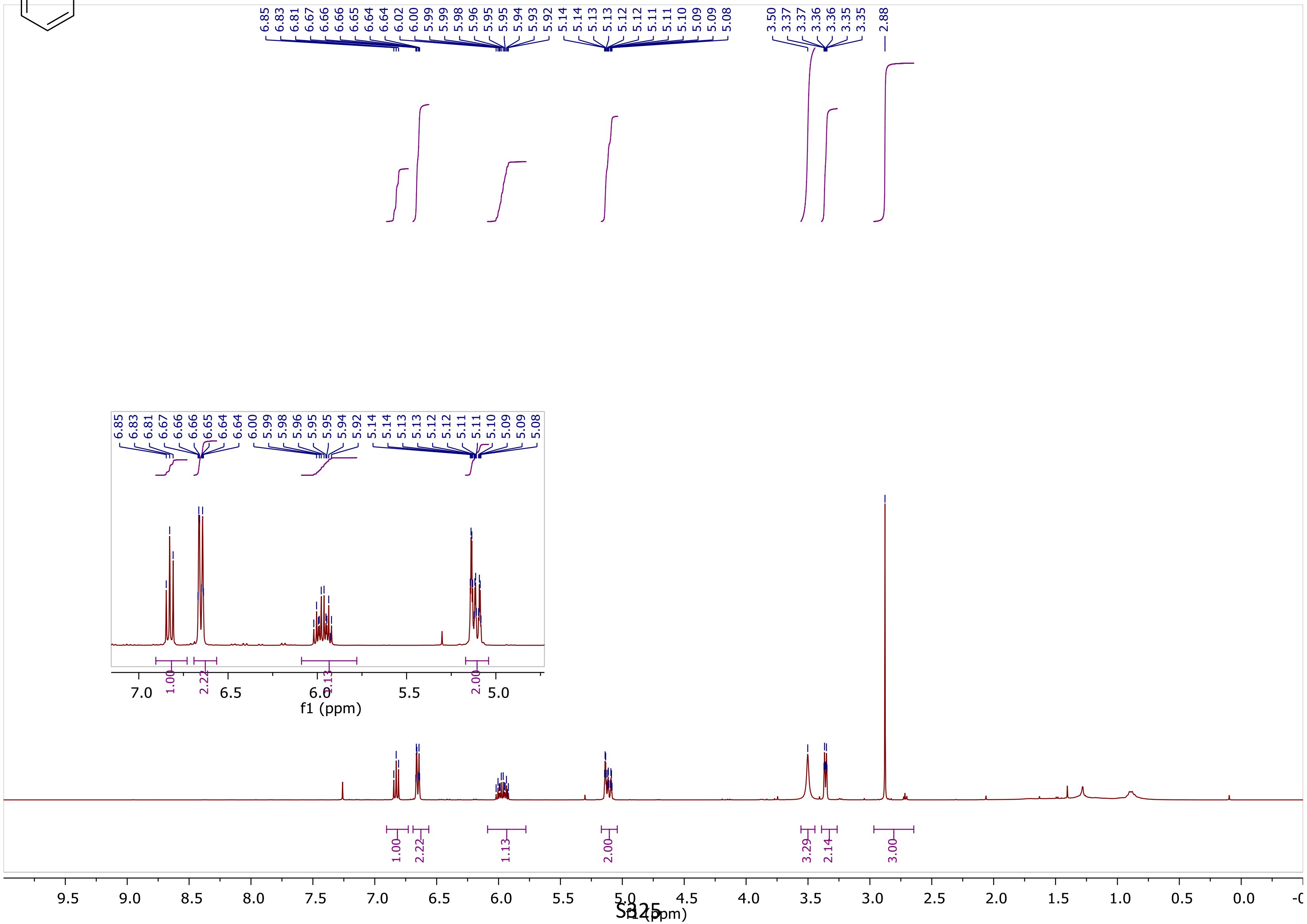

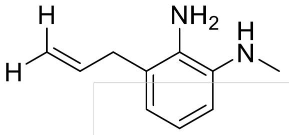

^{1}H NMR of 3-allyl-*N*1-methylbenzene-1,2-diamine **5t** in $CDCl_3$

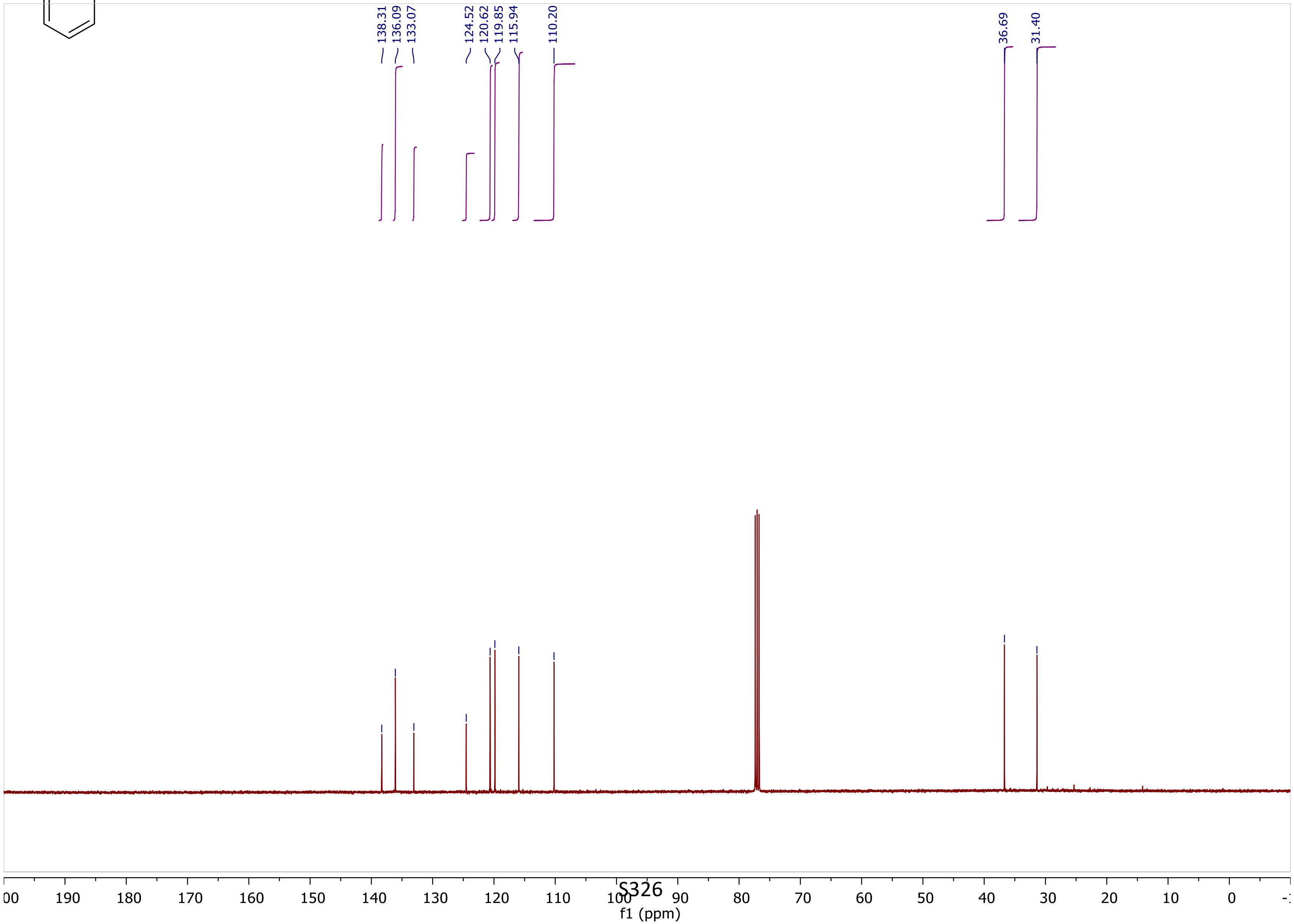

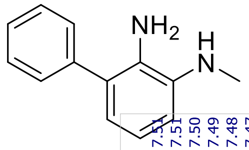

^{1}H NMR of 3-phenyl-*N*1-methylbenzene-1,2-diamine **5u** in $CDCl_3$

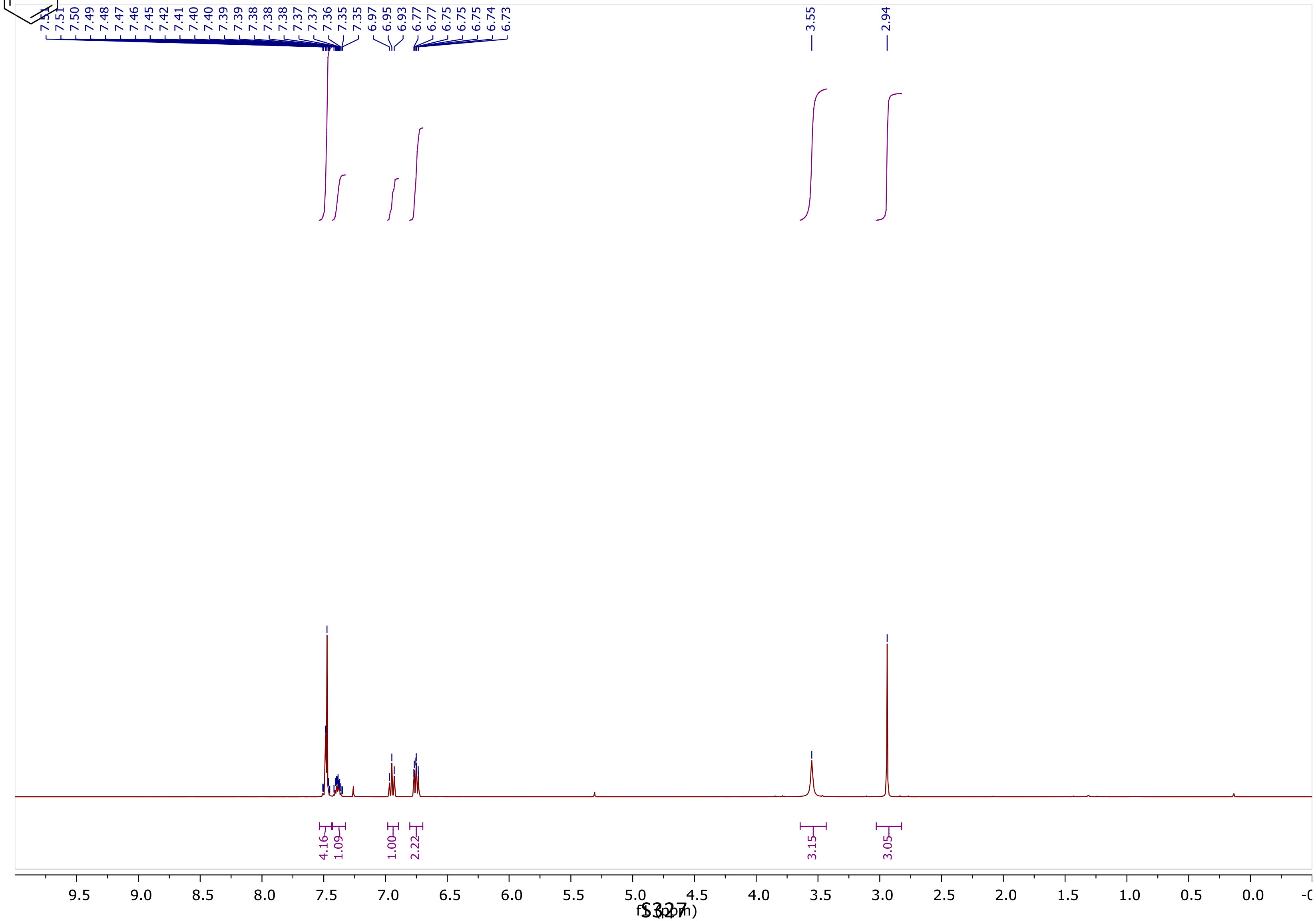

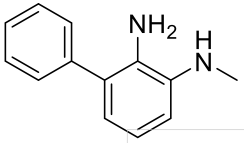

^{13}C NMR of 3-phenyl-*N*1-methylbenzene-1,2-diamine **5u** in $CDCl_3$

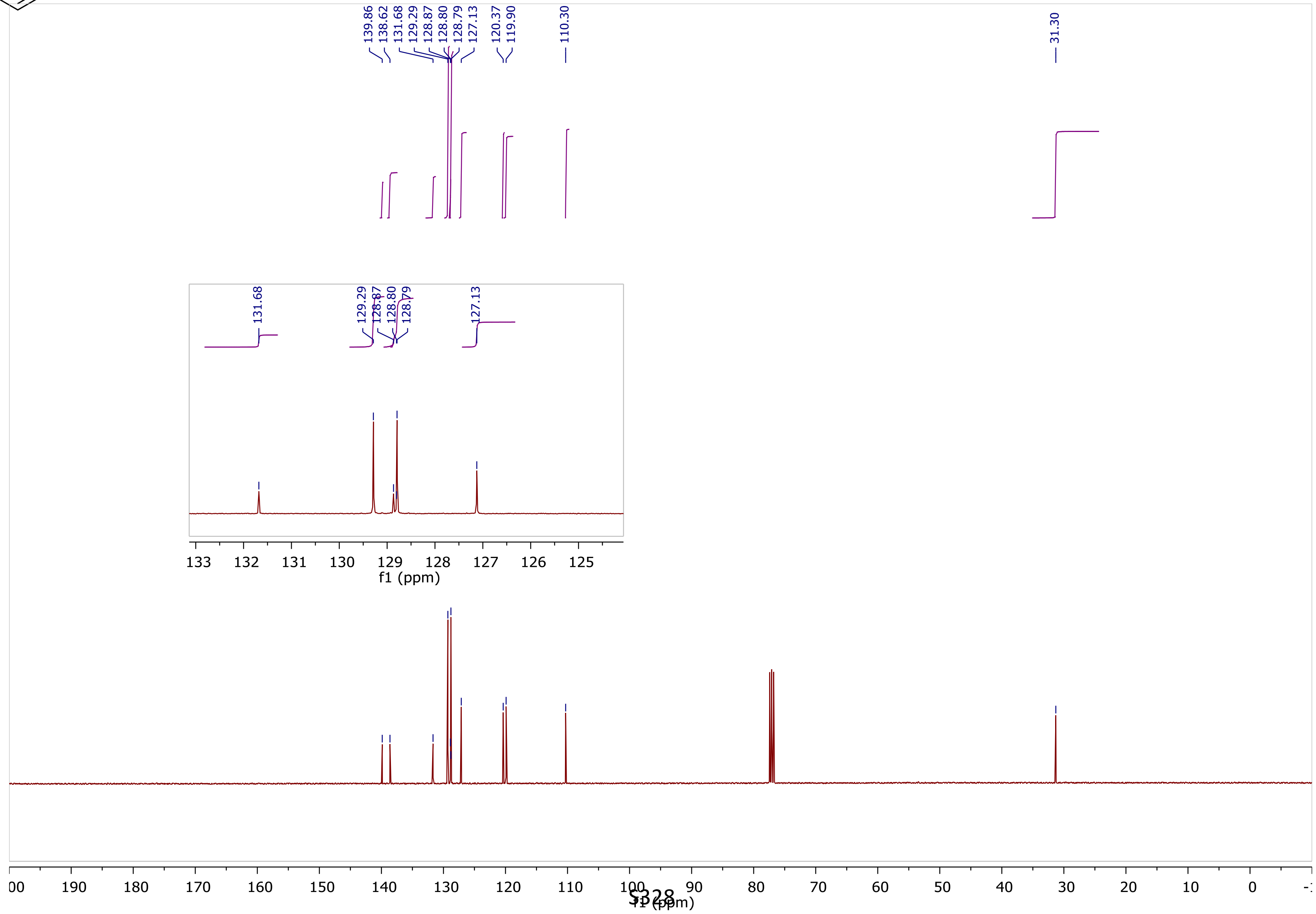

^{1}H NMR of 3-(4-chlorophenyl)-*N*1-methylbenzene-1,2-diamine **5v** in $CDCl_3$

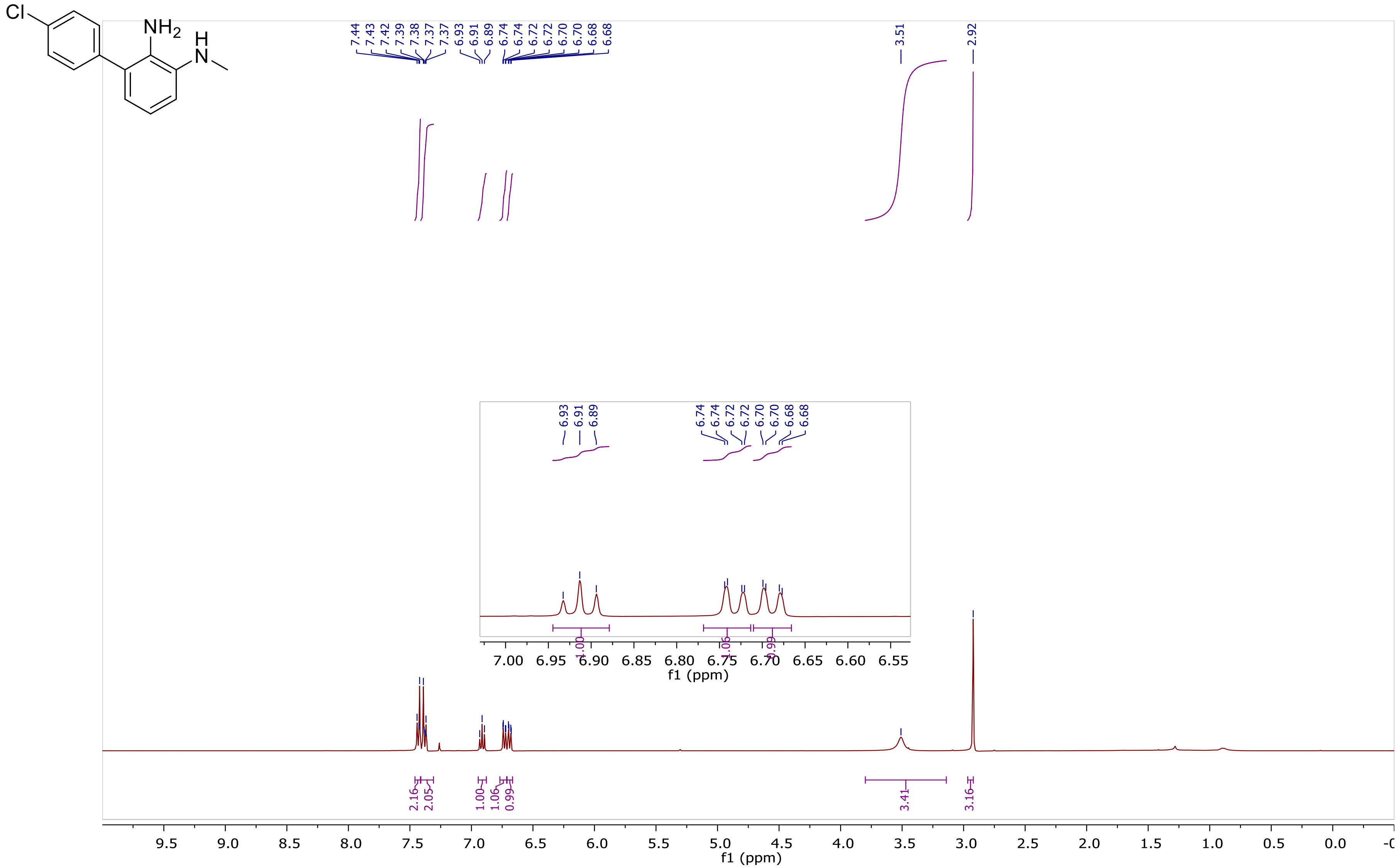

^{13}C NMR of 3-(4-chlorophenyl)-*N*1-methylbenzene-1,2-diamine **5v** in $CDCl_3$

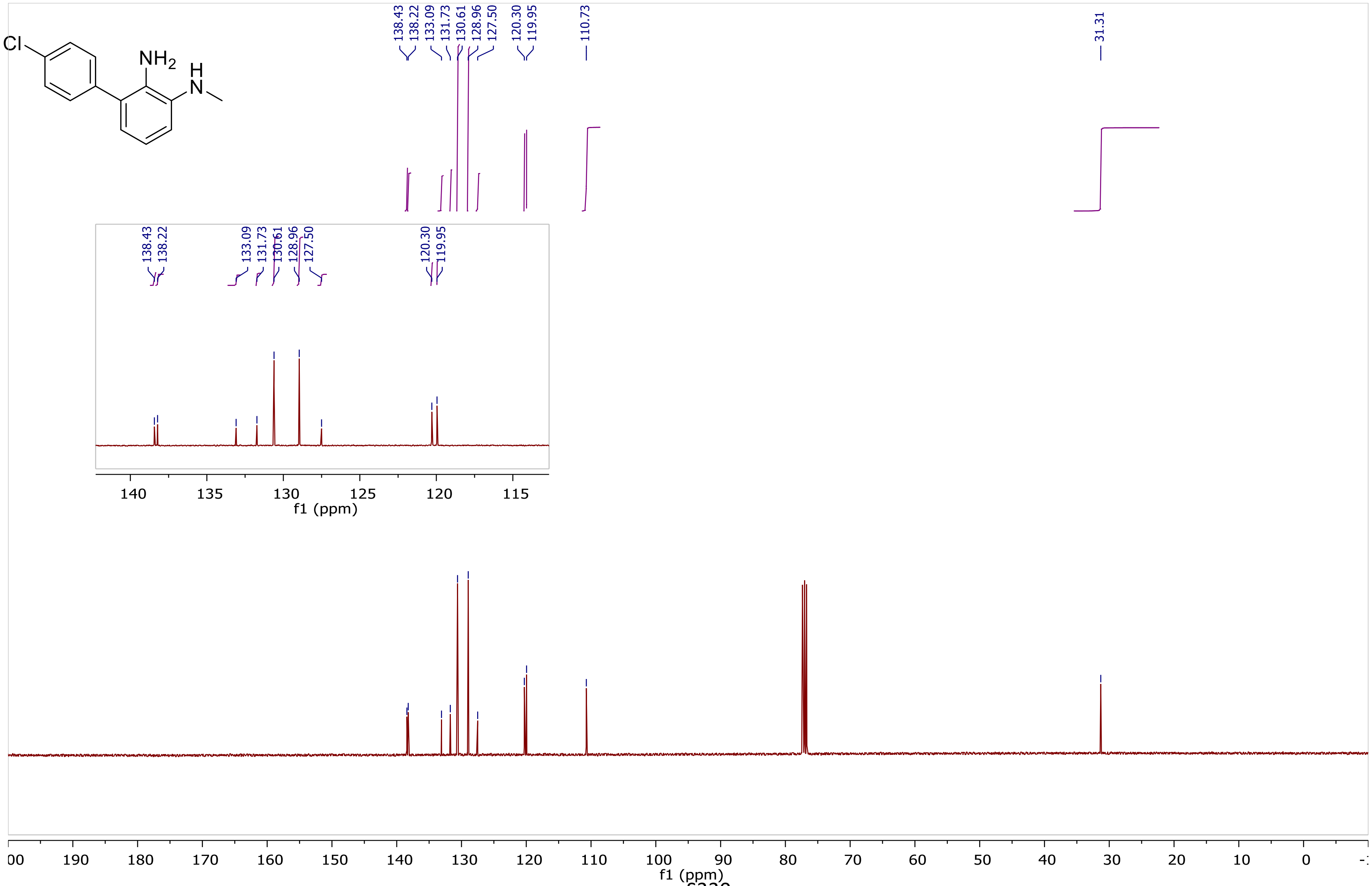

^{1}H NMR of 3-(4-methylphenyl)-*N*1-methylbenzene-1,2-diamine **5w** in $CDCl_3$

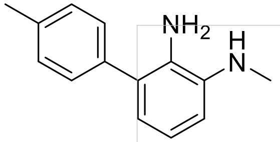

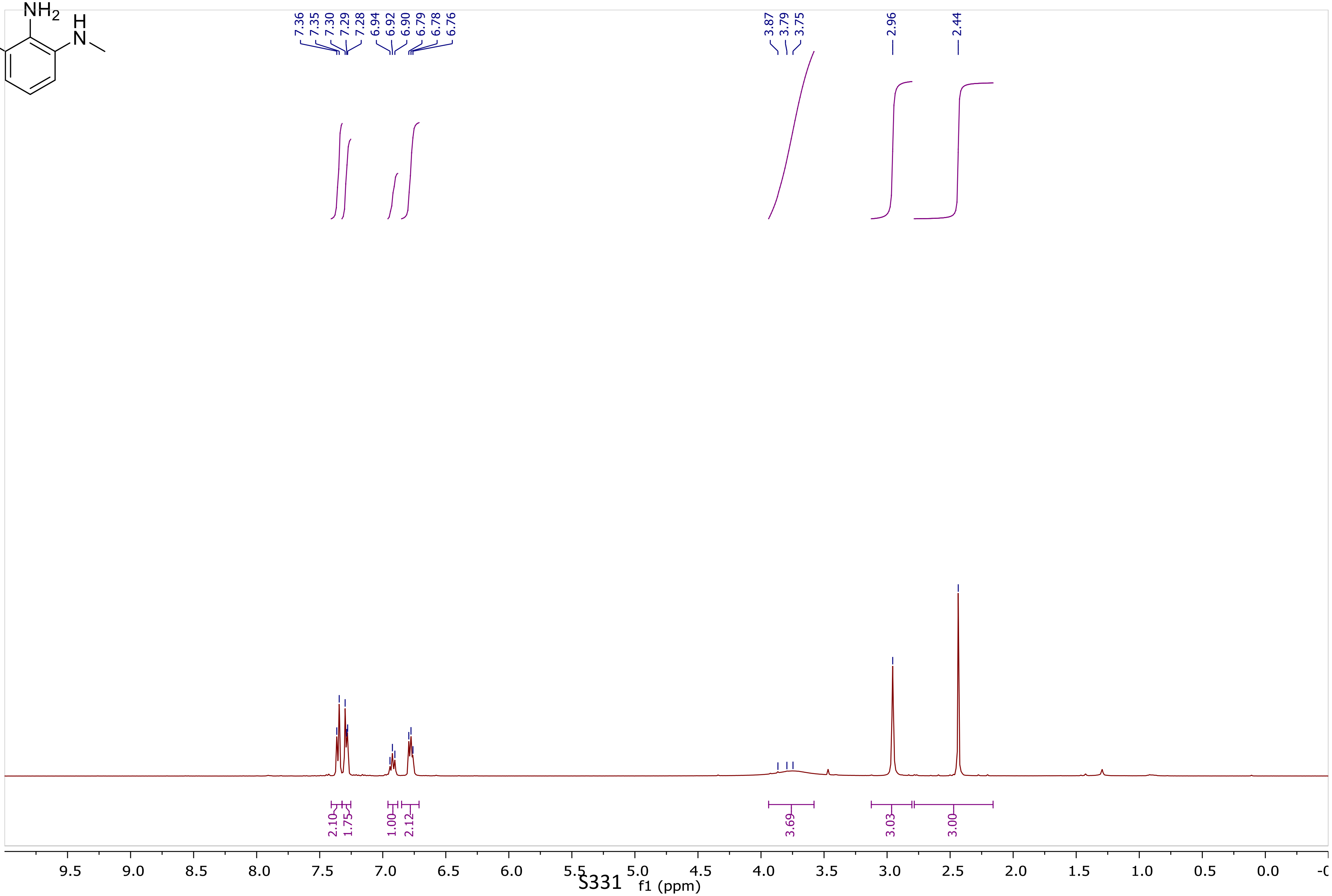

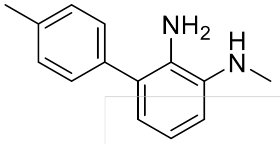

^{13}C NMR of 3-(4-methylphenyl)-*N*1-methylbenzene-1,2-diamine **5w** in $CDCl_3$

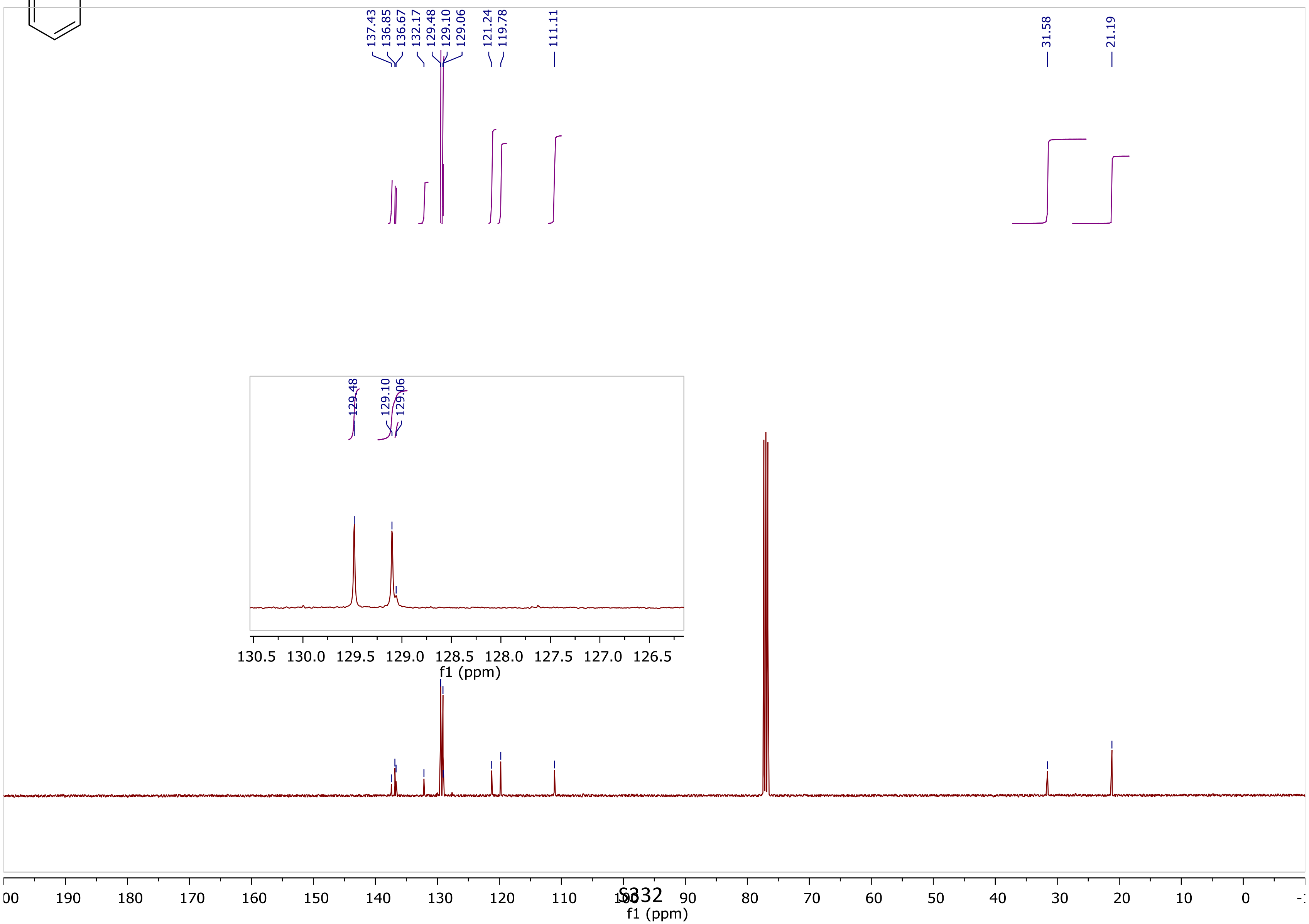

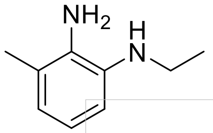

^{1}H NMR of *N*1-ethyl-3-methylbenzene-1,2-diamine **5x** in $CDCl_3$

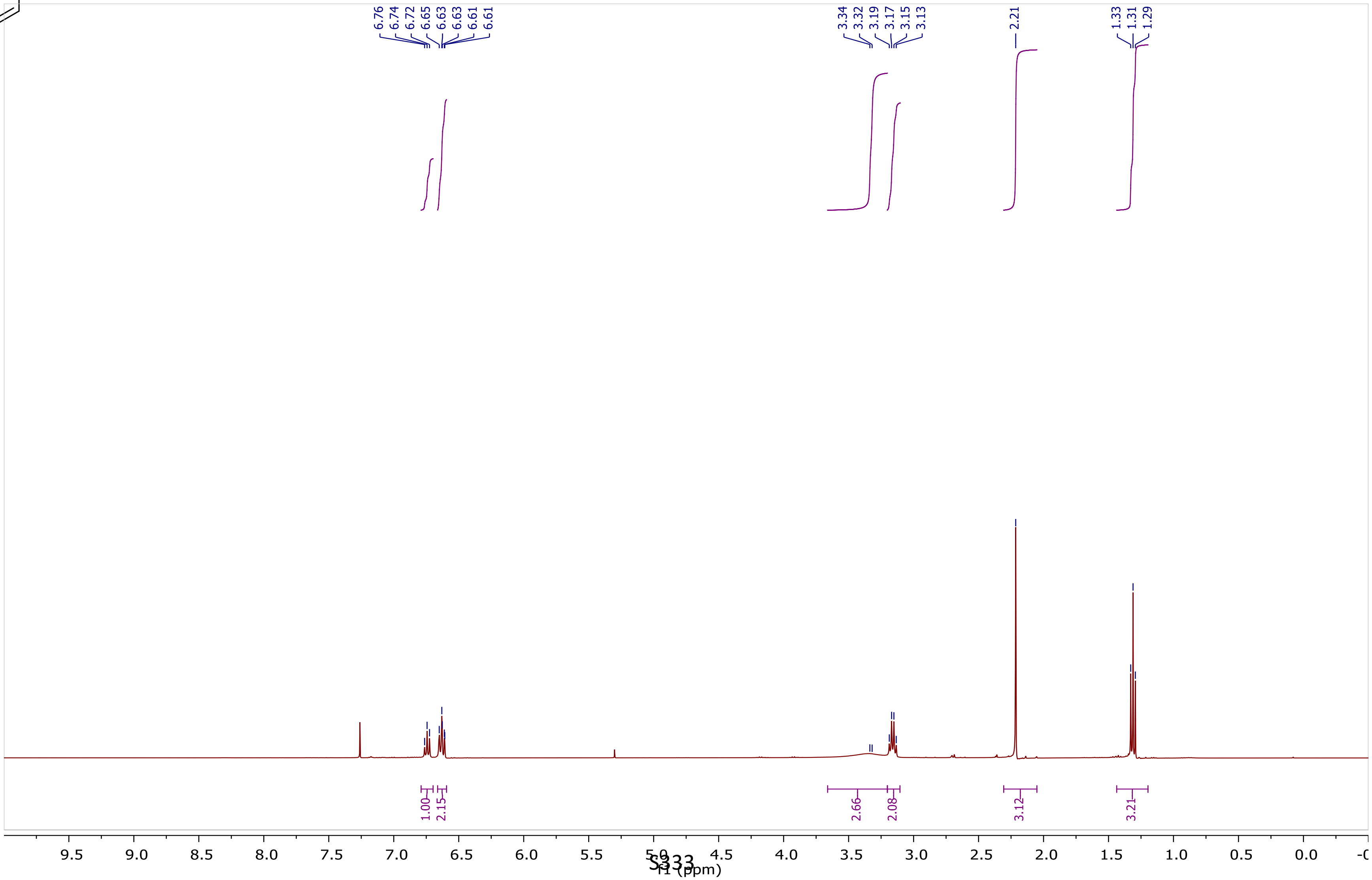

^{13}C NMR of *N*1-ethyl-3-methylbenzene-1,2-diamine **5x** in $CDCl_3$

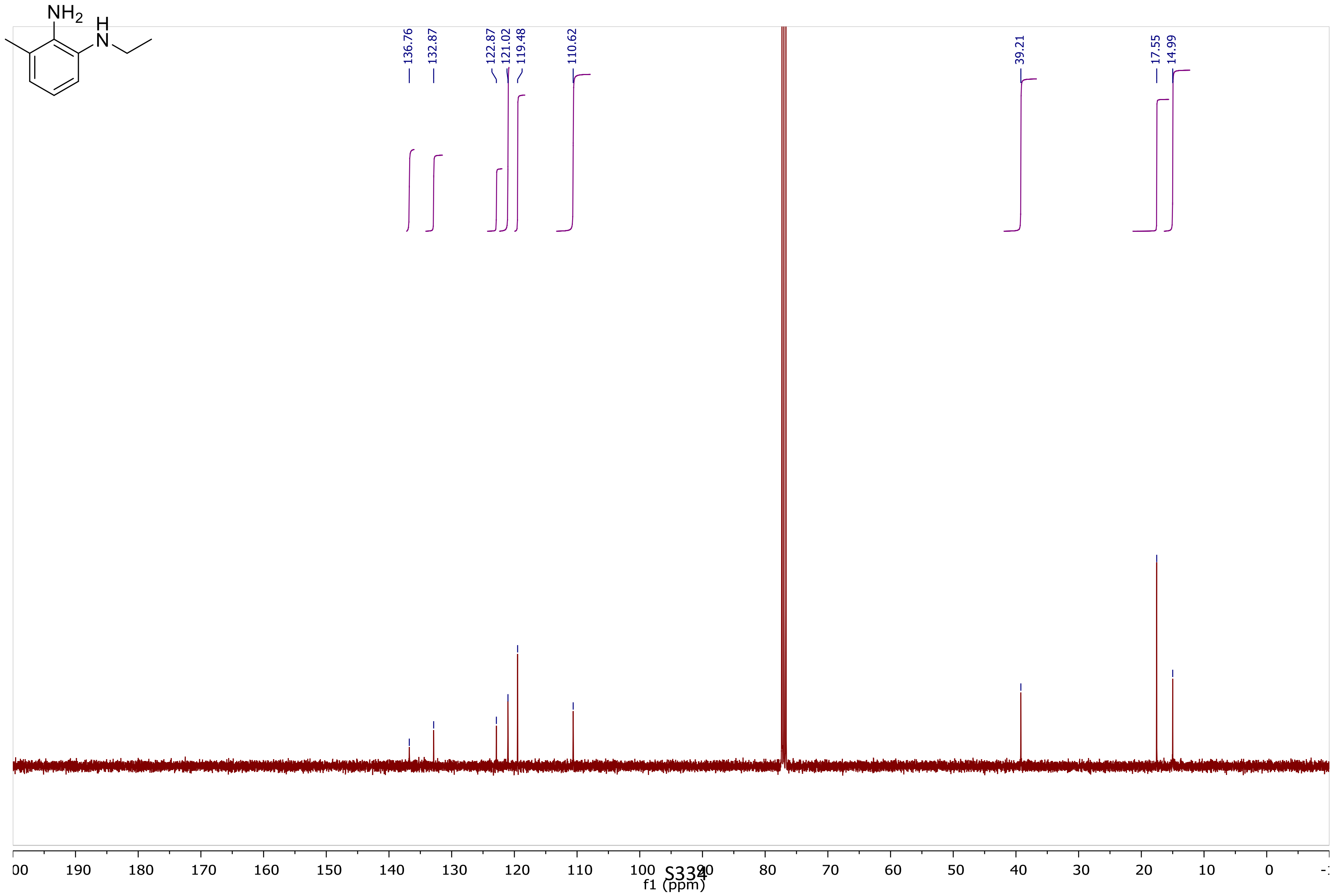

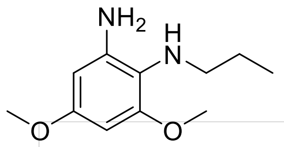

^{1}H NMR of 4,6-dimethoxy-*N*1-propylbenzene-1,2-diamine **5y** in $CDCl_3$

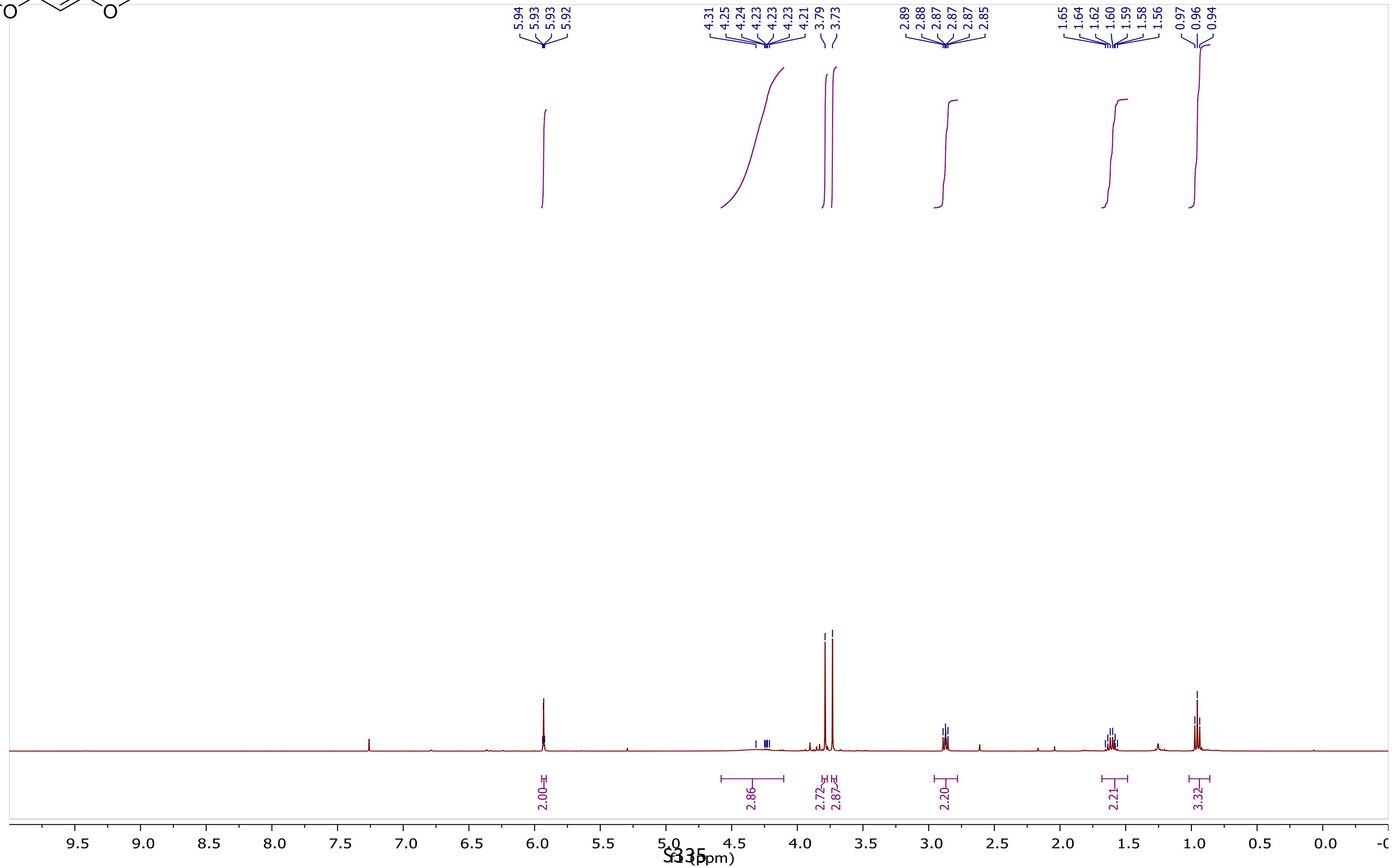

^{13}C NMR of 4,6-dimethoxy-*N*1-propylbenzene-1,2-diamine **5y** in $CDCl_3$

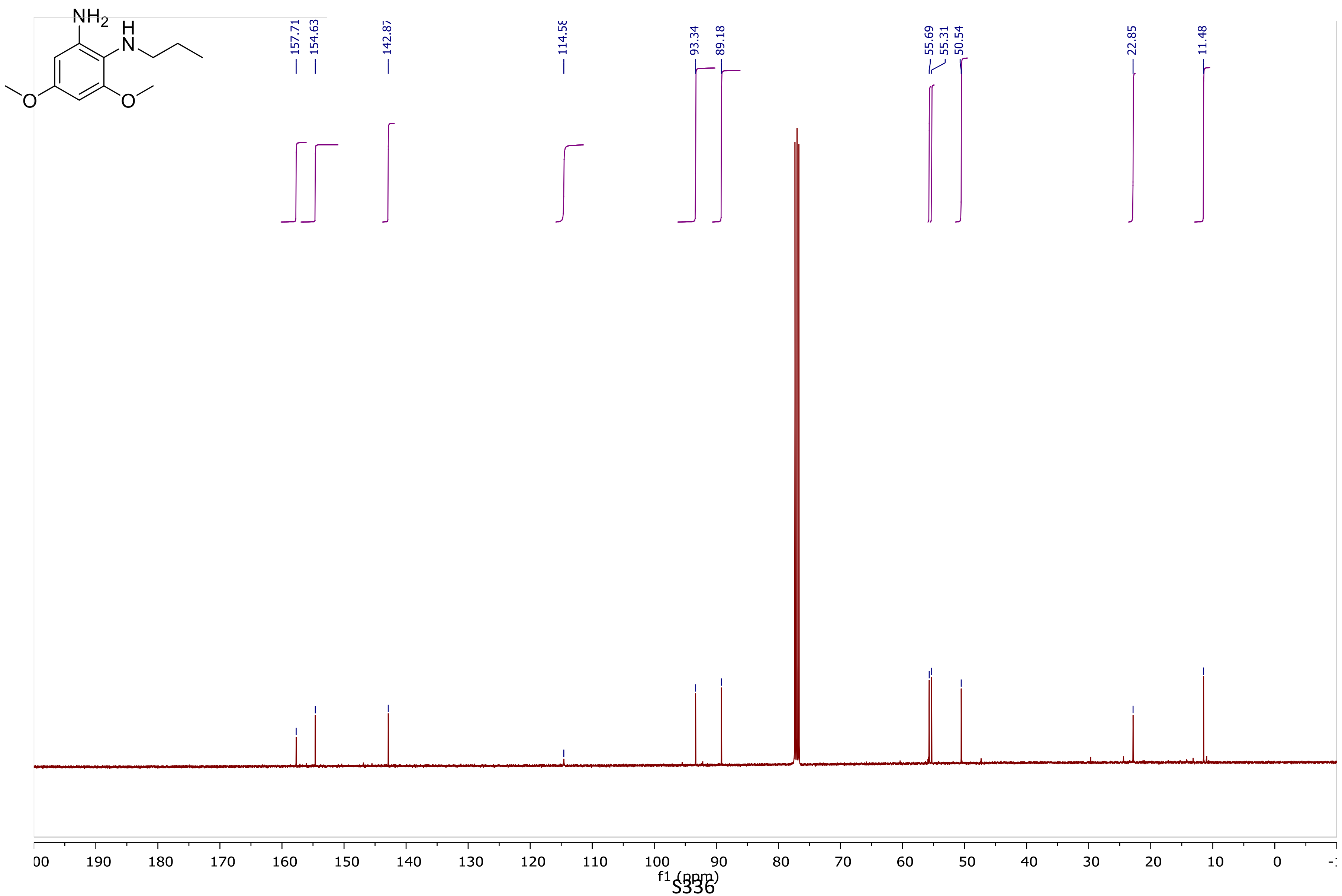

^1H NMR of 3-((2-amino-4-methoxy-3-methylphenyl)amino)propanenitrile **5z-i** in $CDCl_3$

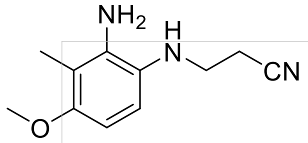

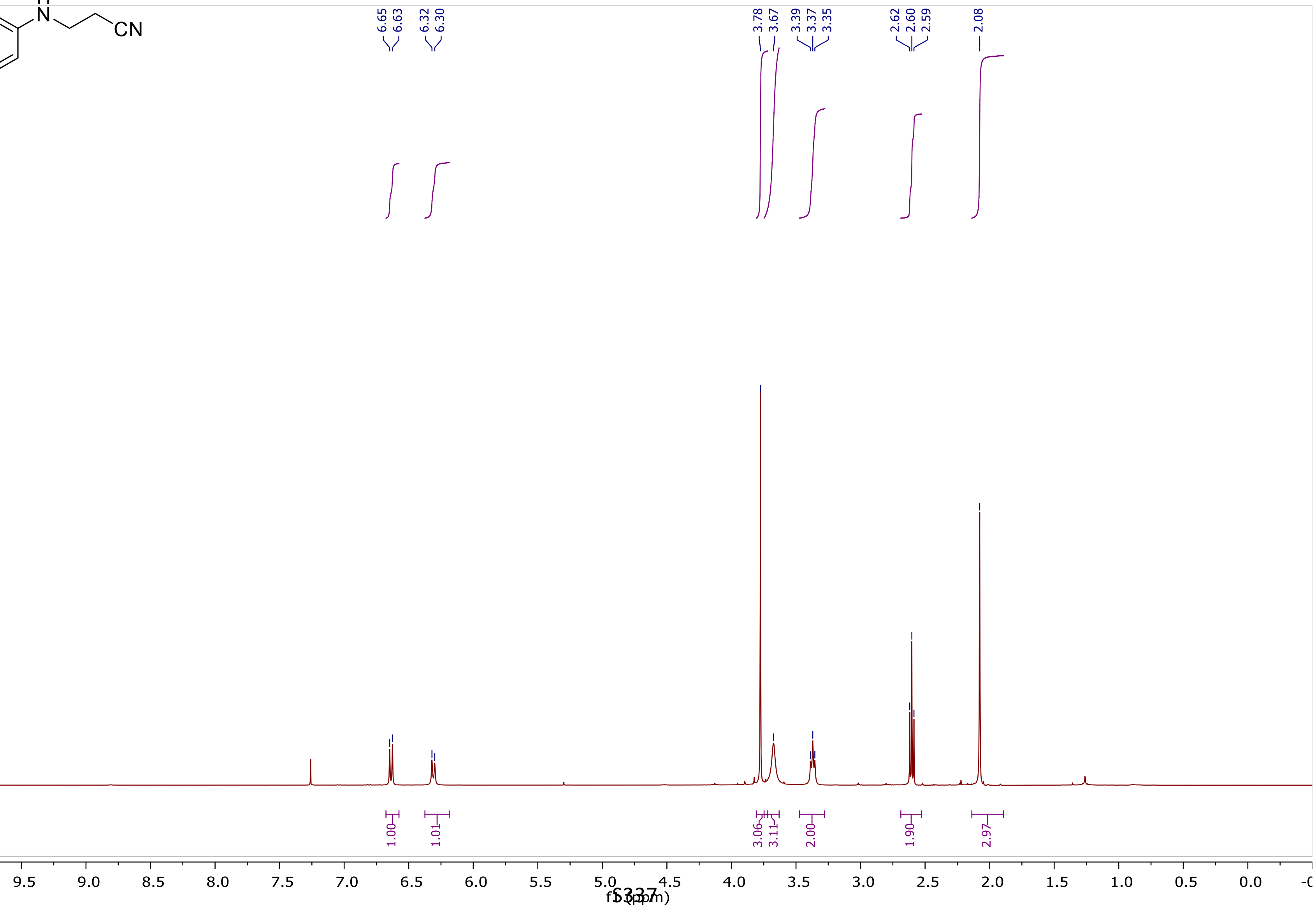

^{13}C NMR of 3-((2-amino-4-methoxy-3-methylphenyl)amino)propanenitrile **5z-i** in $CDCl_3$

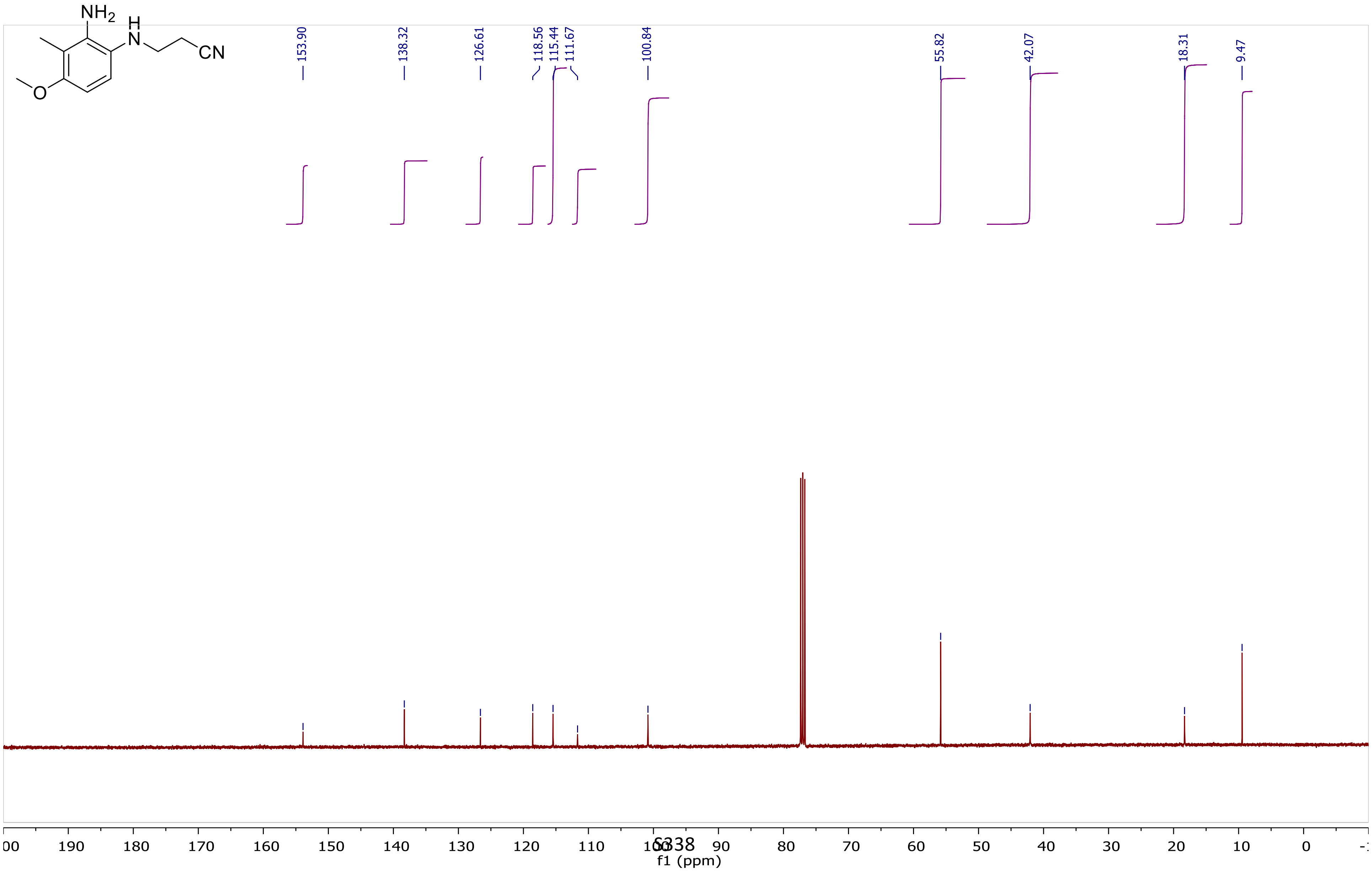

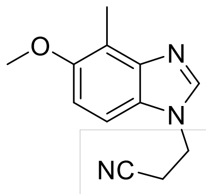

^{1}H NMR of 3-(5-methoxy-4-methyl-1H-benzo[d]imidazol-1-yl)propanenitrile **5z-ii** in $CDCl_3$

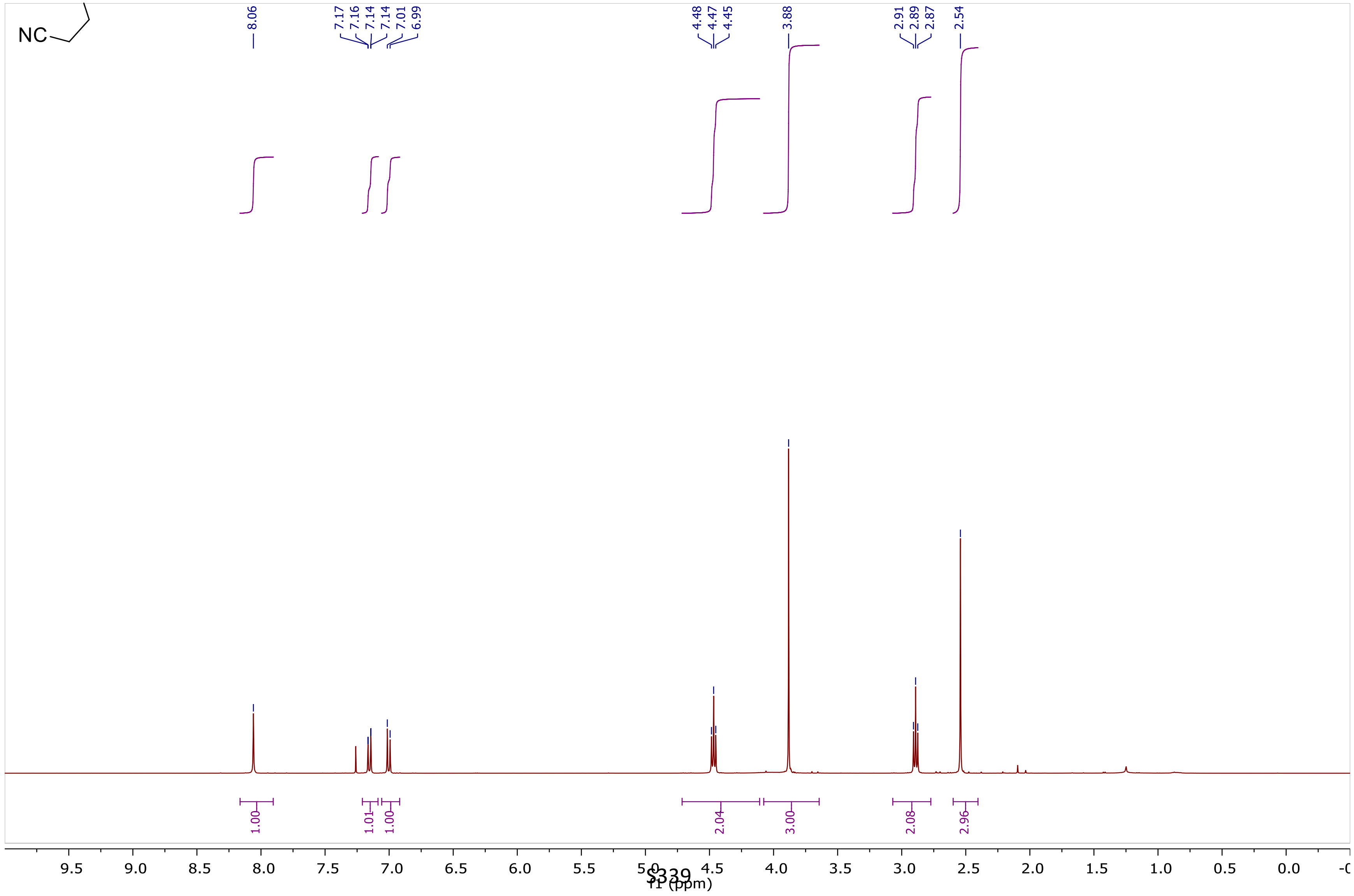

^13^C NMR of 3-(5-methoxy-4-methyl-1H-benzo[d]imidazol-1-yl)propanenitrile **5z-ii** in $CDCl_3$

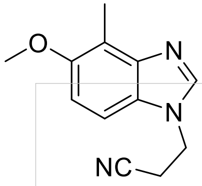

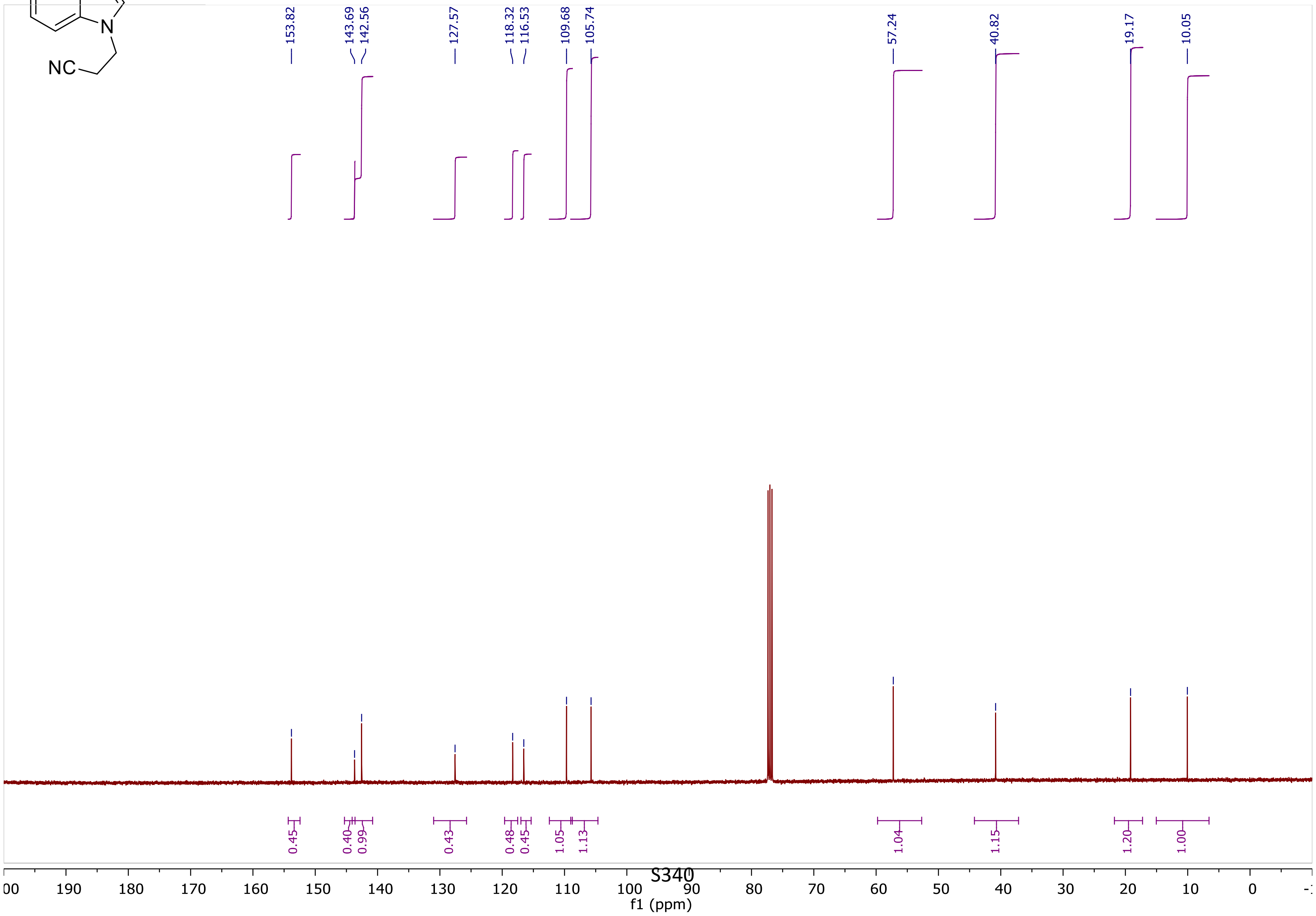

^{1}H NMR of 1-hexyl-5-methoxy-4-methyl-1H-benzo[d]imidazole **5aa** in $CDCl_3$

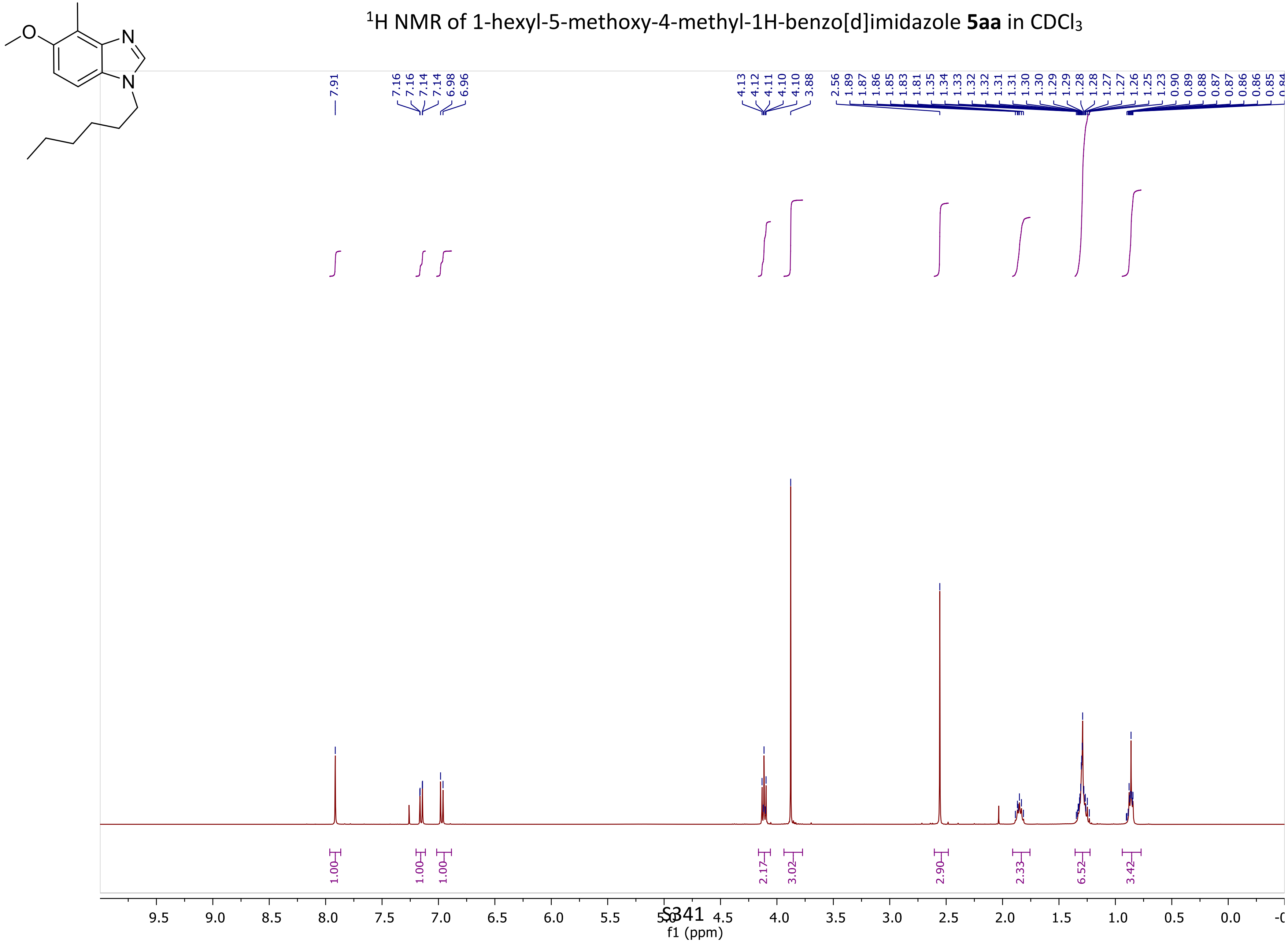

^{13}C NMR of 1-hexyl-5-methoxy-4-methyl-1H-benzo[d]imidazole **5aa** in $CDCl_3$

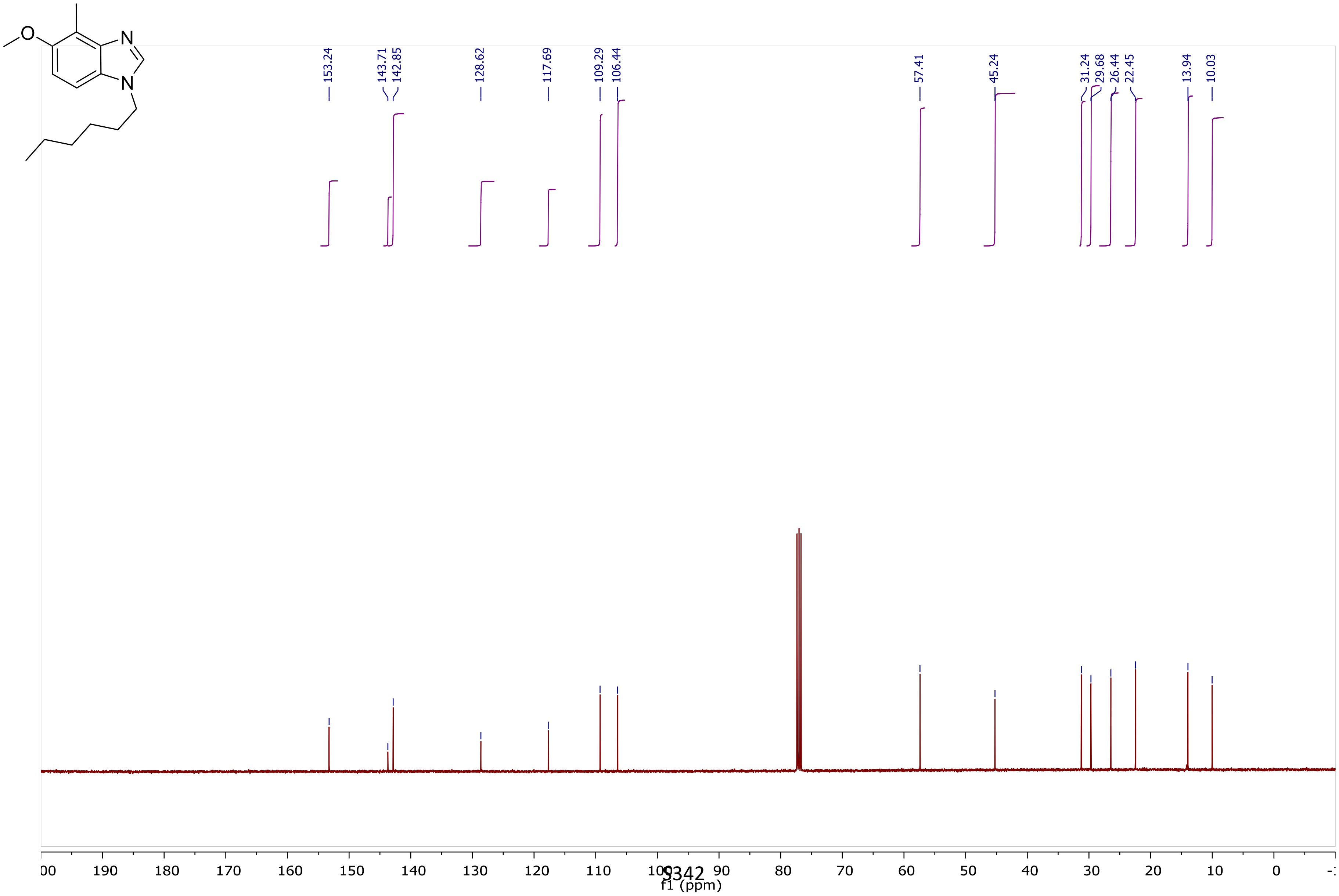

NH_2 Transfer Products

^{1}H NMR of benzene-1,2-diamine **4a** in $CDCl_3$

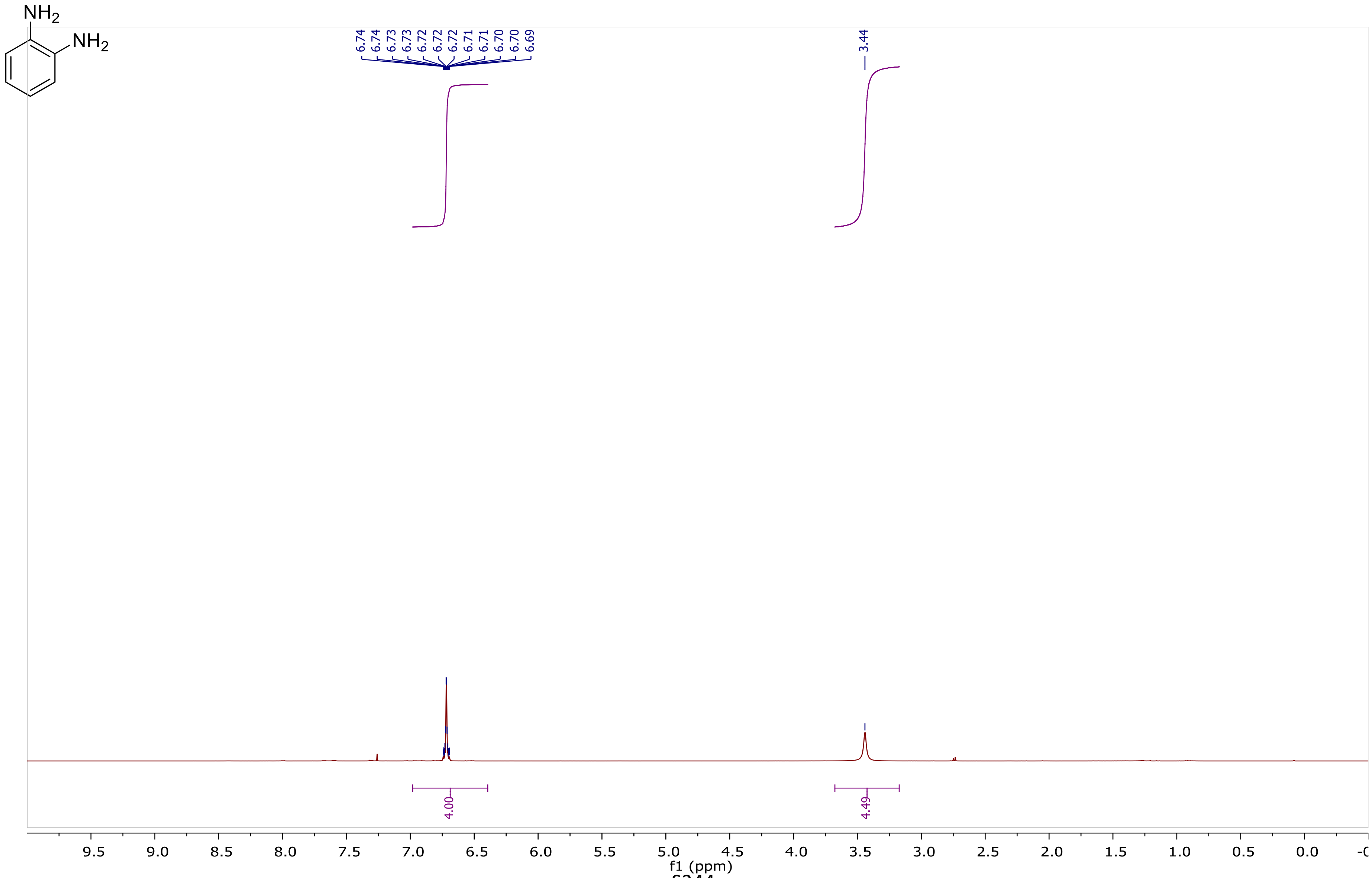

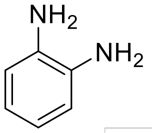

^{13}C NMR of benzene-1,2-diamine **4a** in $CDCl_3$

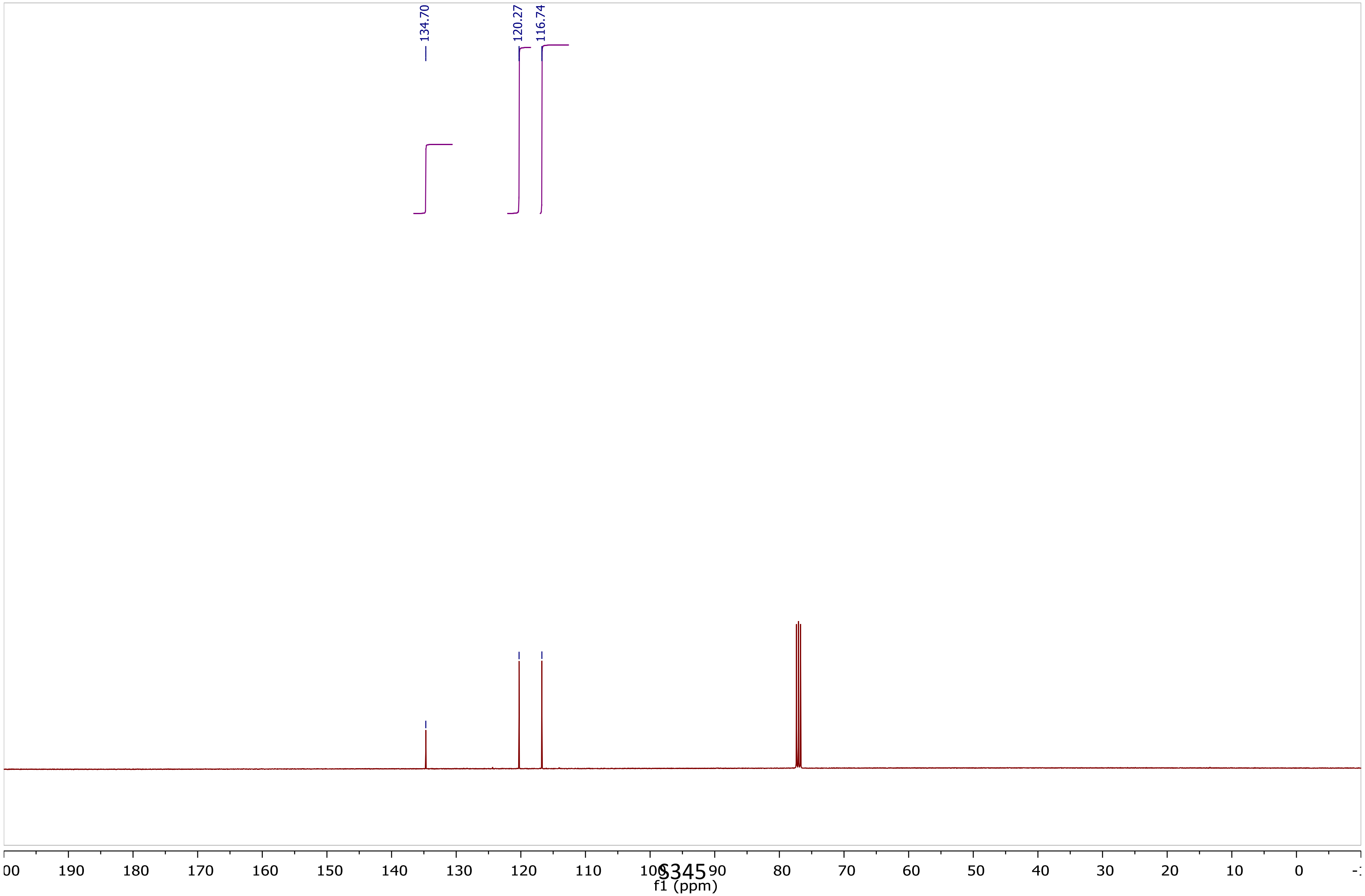

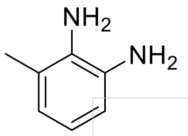

^{1}H NMR of 3-methylbenzene-1,2-diamine **4b** in $CDCl_3$

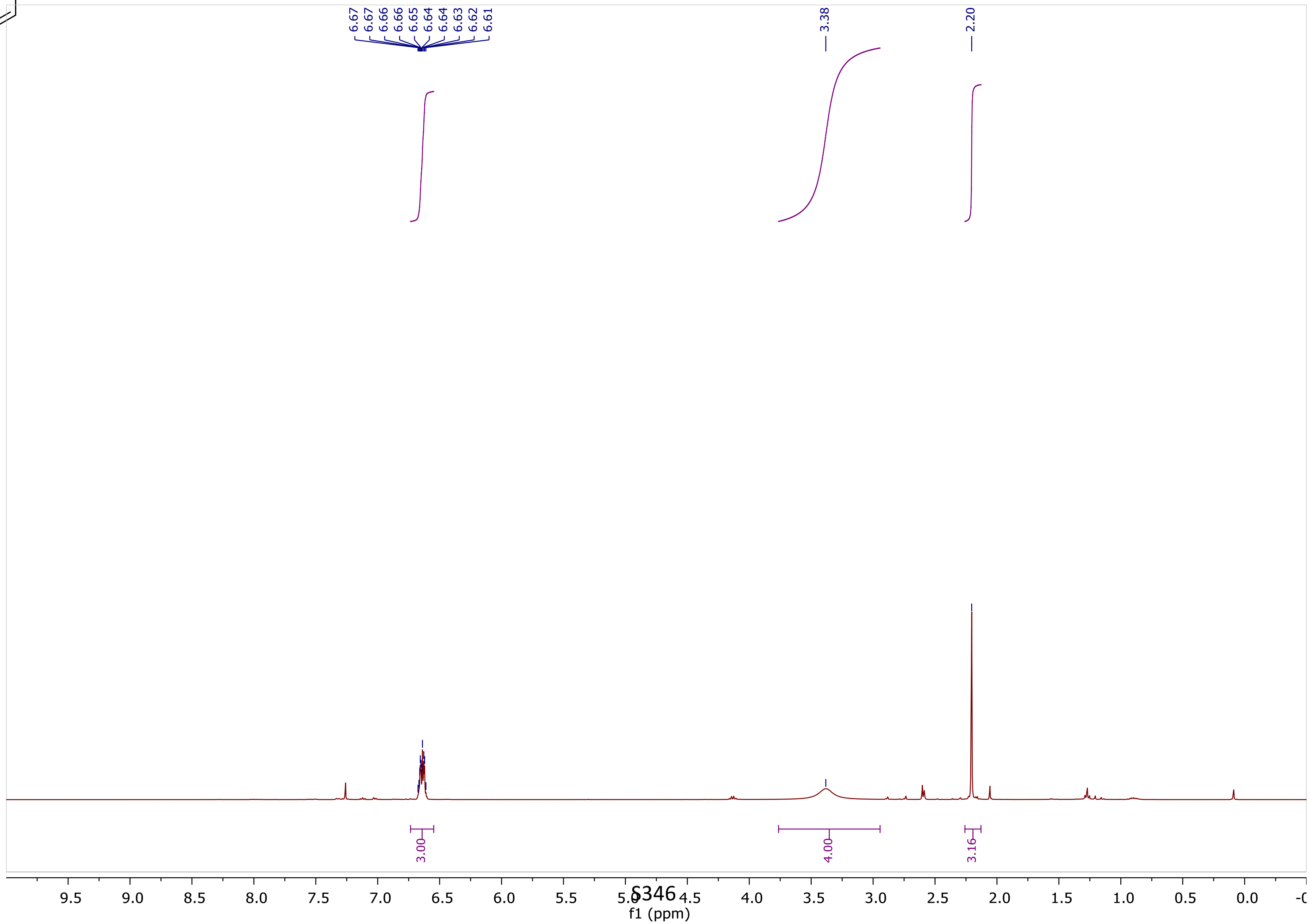

^{13}C NMR of 3-methylbenzene-1,2-diamine **4b** in $CDCl_3$

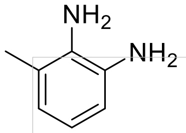

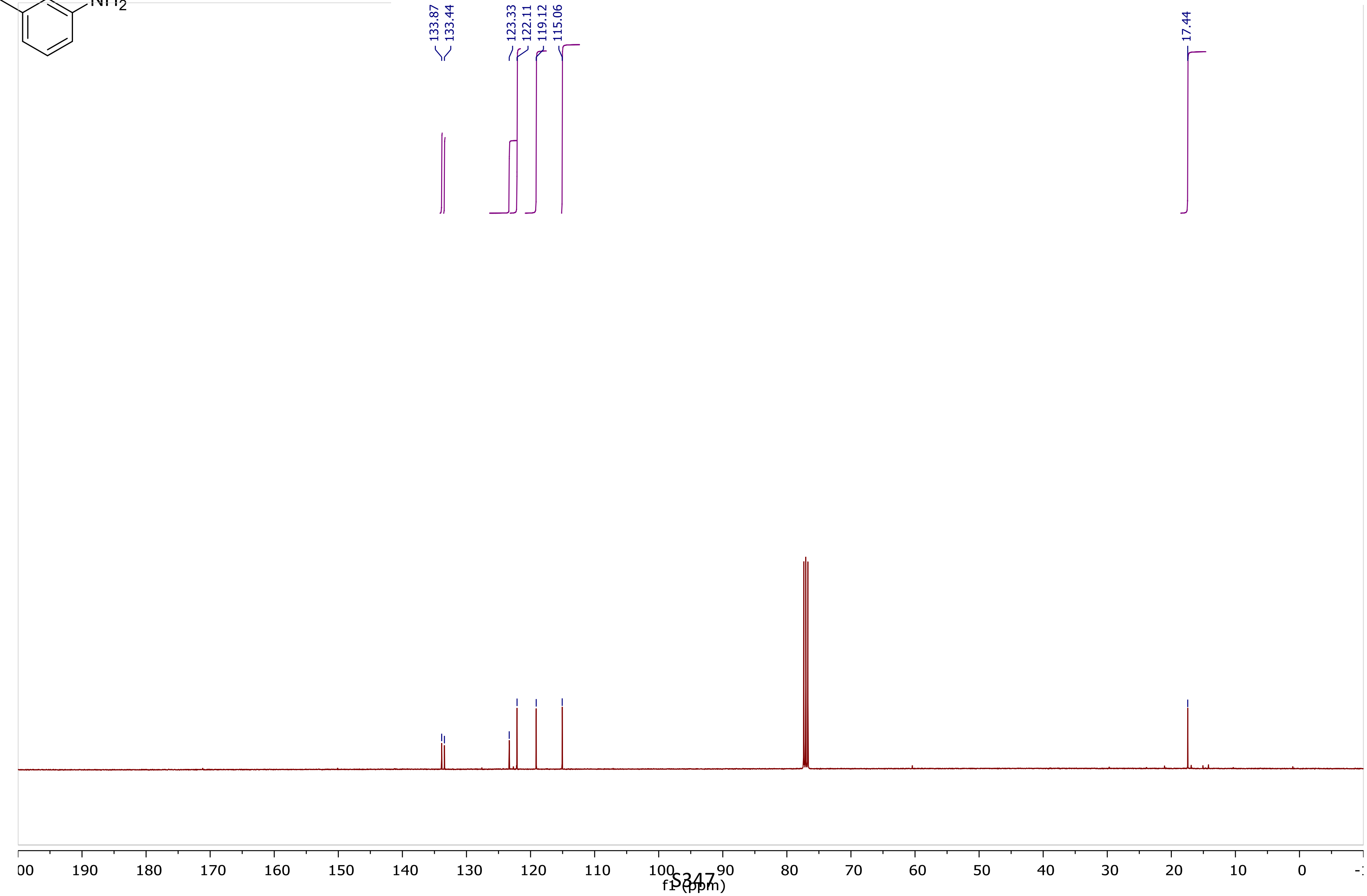

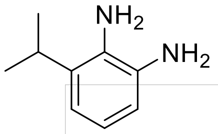

^{1}H NMR of 3-isopropylbenzene-1,2-diamine **4c-i** in MeOD-d[4]

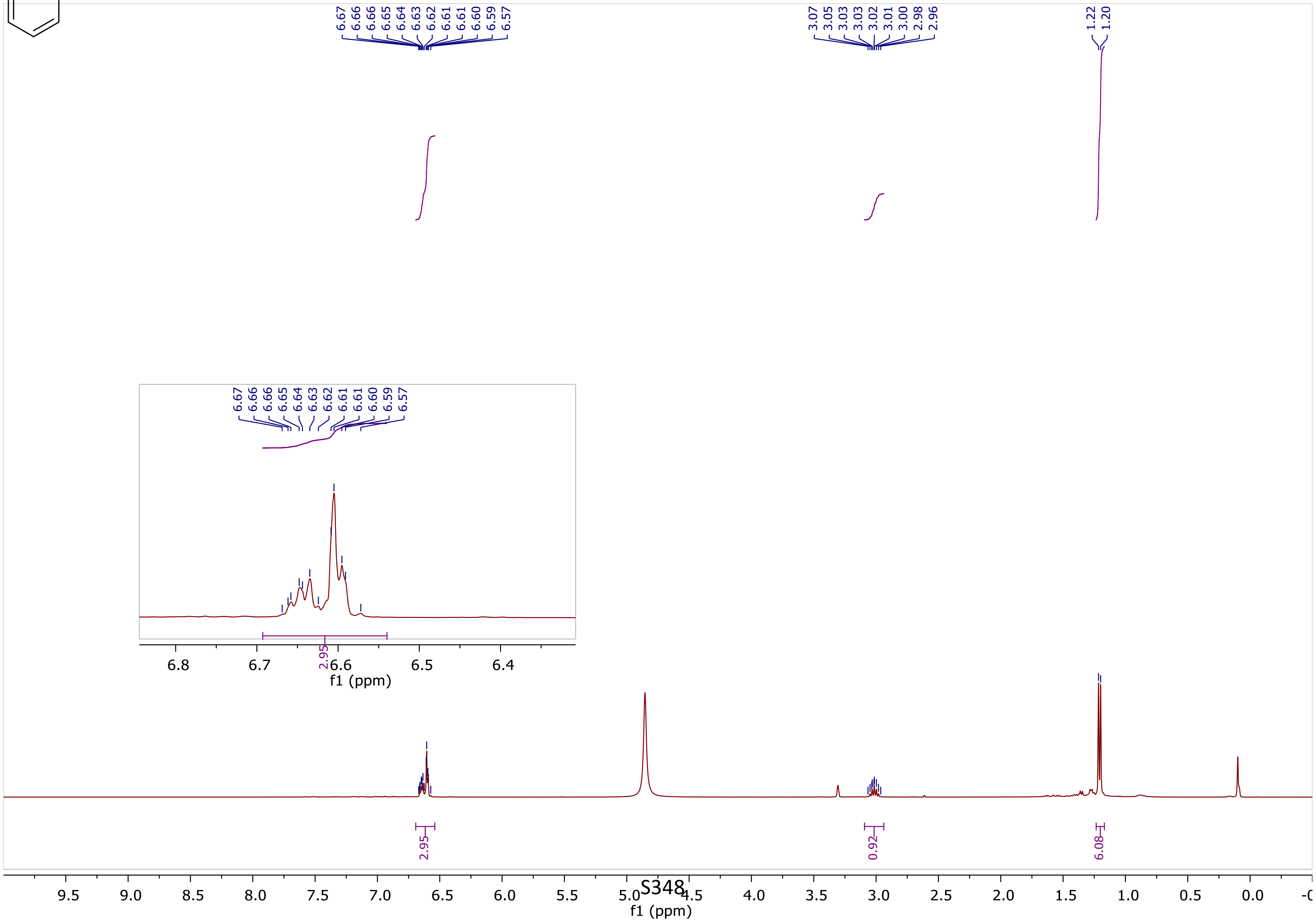

^{13}C NMR of 3-isopropylbenzene-1,2-diamine **4c-i** in MeOD-d[4]

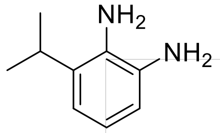

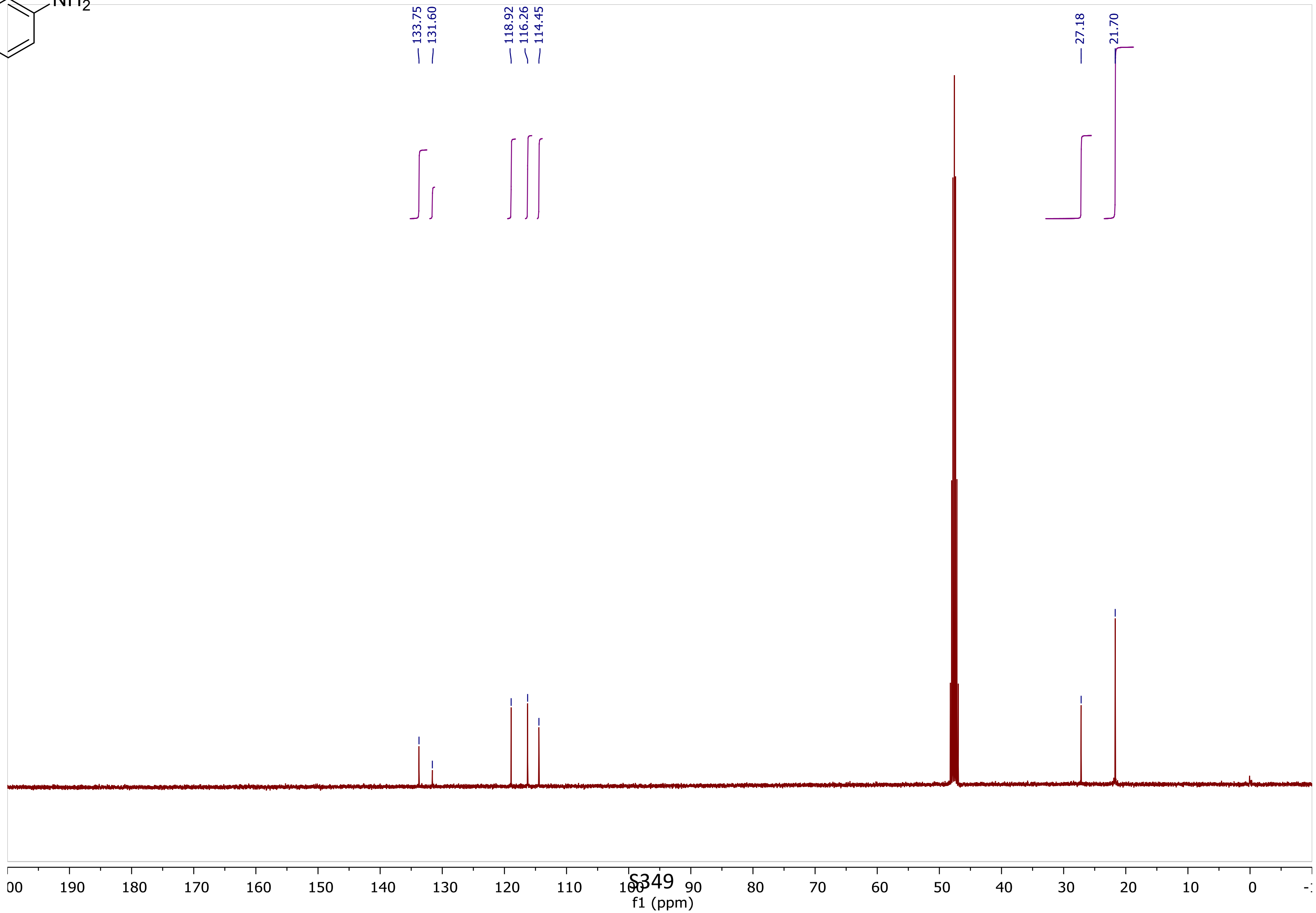

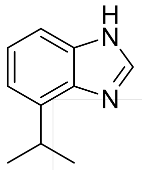

^{1}H NMR of 4-isopropyl-1*H*-benzo[d]imidazole **4c-ii** in $CDCl_3$

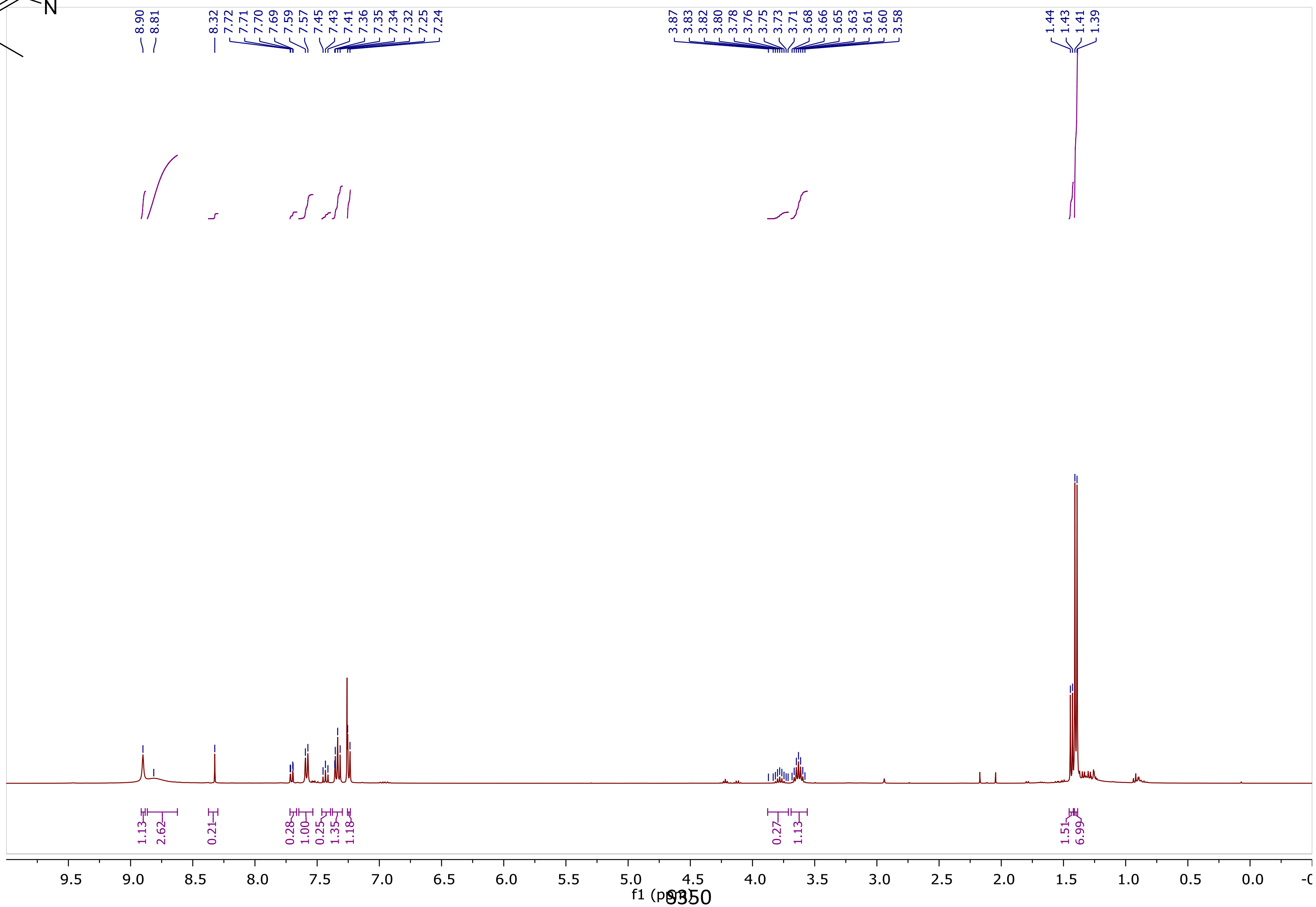

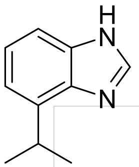

^{13}C NMR of 4-isopropyl-1*H*-benzo[d]imidazole **4c-ii** in $CDCl_3$

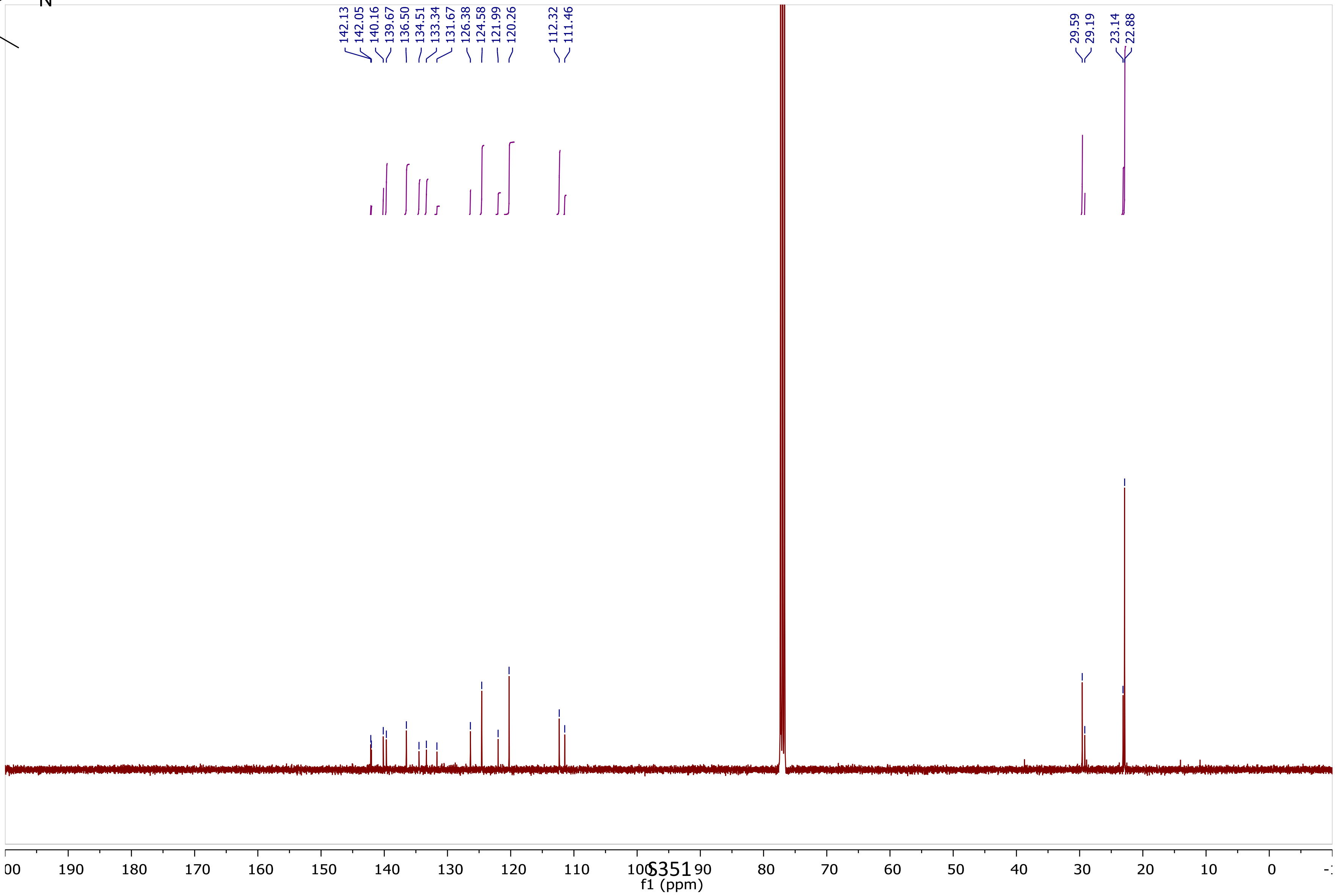

^{1}H NMR of 3-chlorobenzene-1,2-diamine **4d-i** in $CDCl_3$

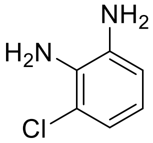

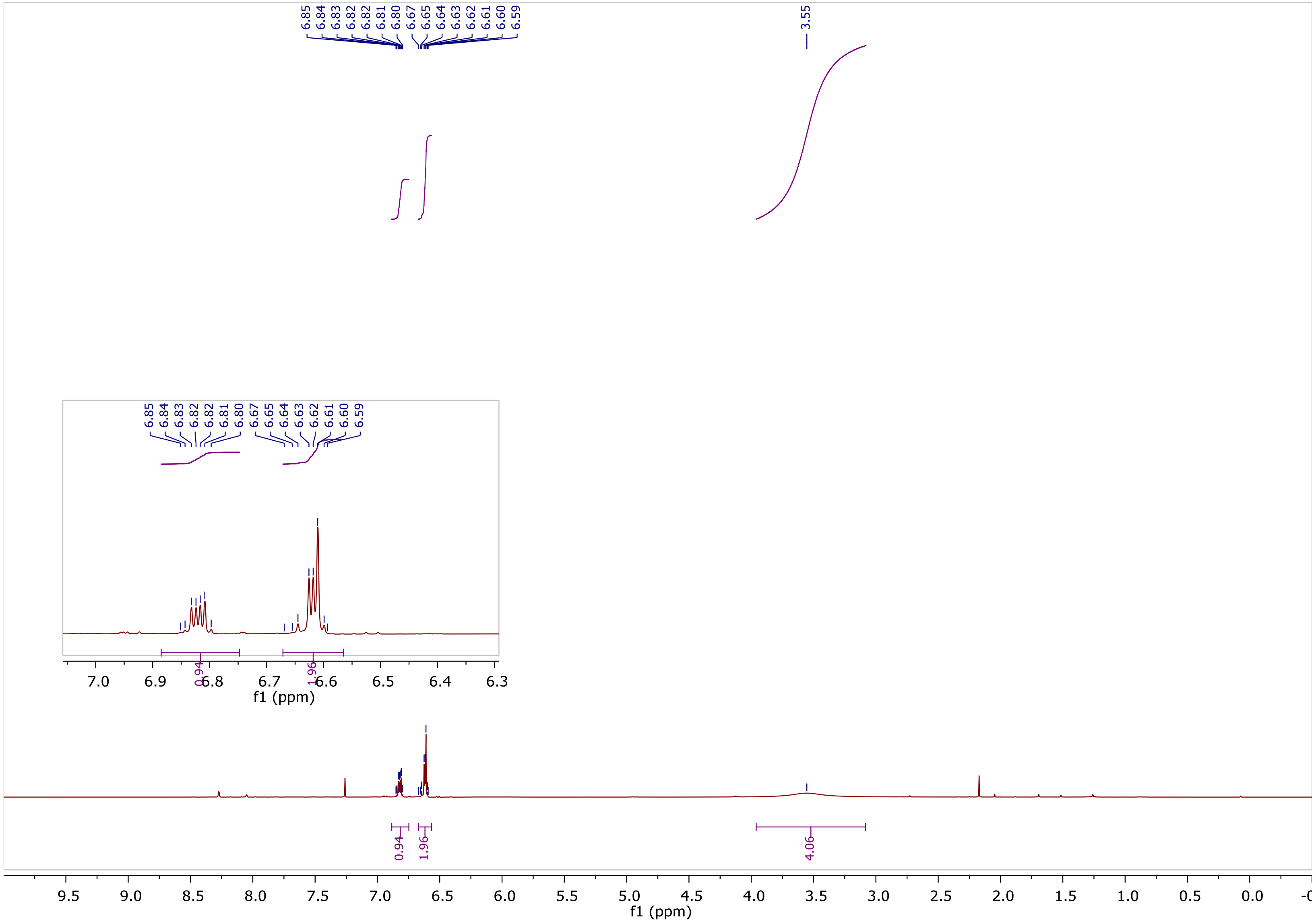

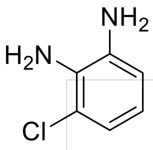

^{13}C NMR of 3-chlorobenzene-1,2-diamine **4d-i** in $CDCl_3$

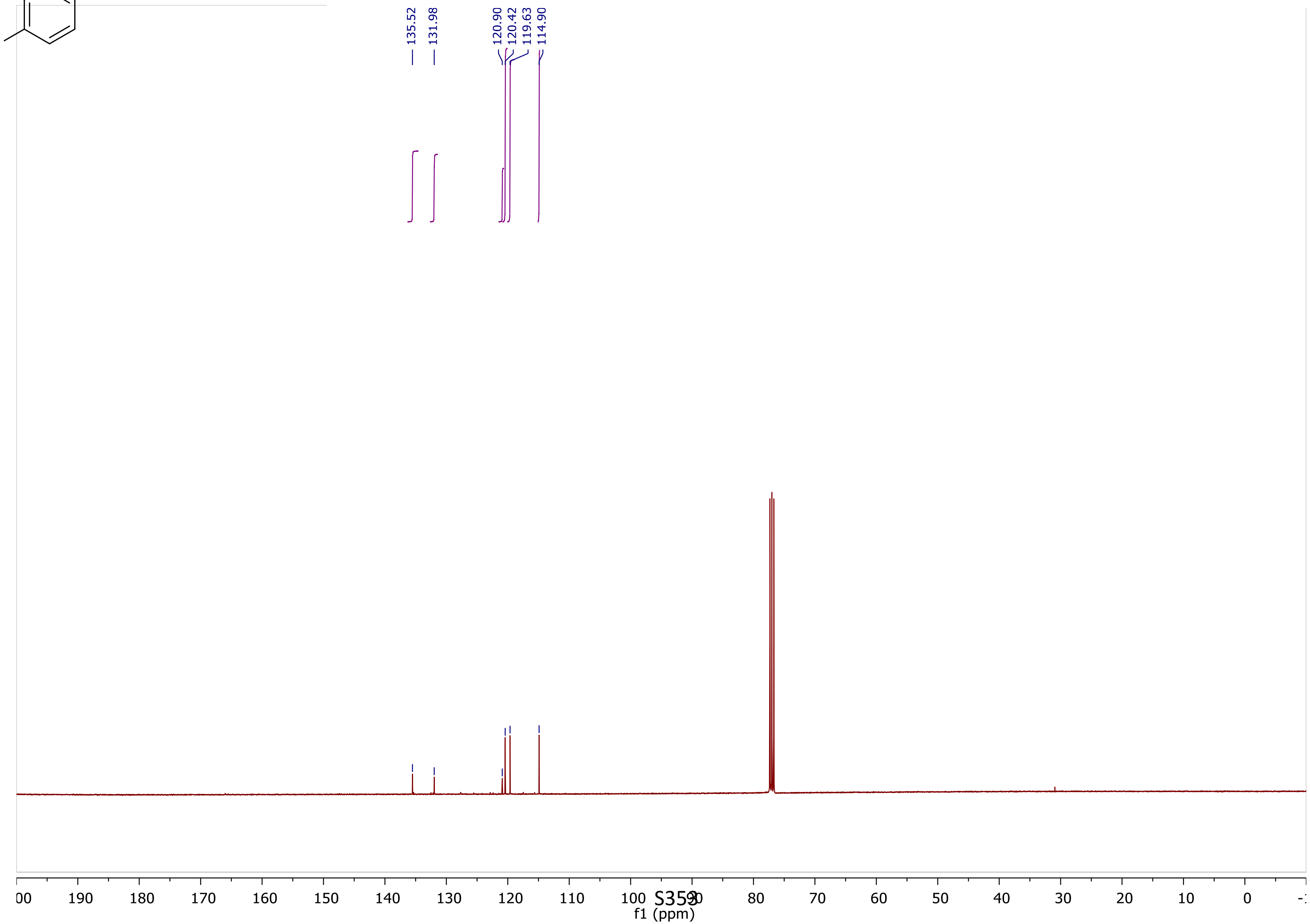

^{1}H NMR of 4-chlorobenzene-1,2-diamine **4d-ii** in $CDCl_3$

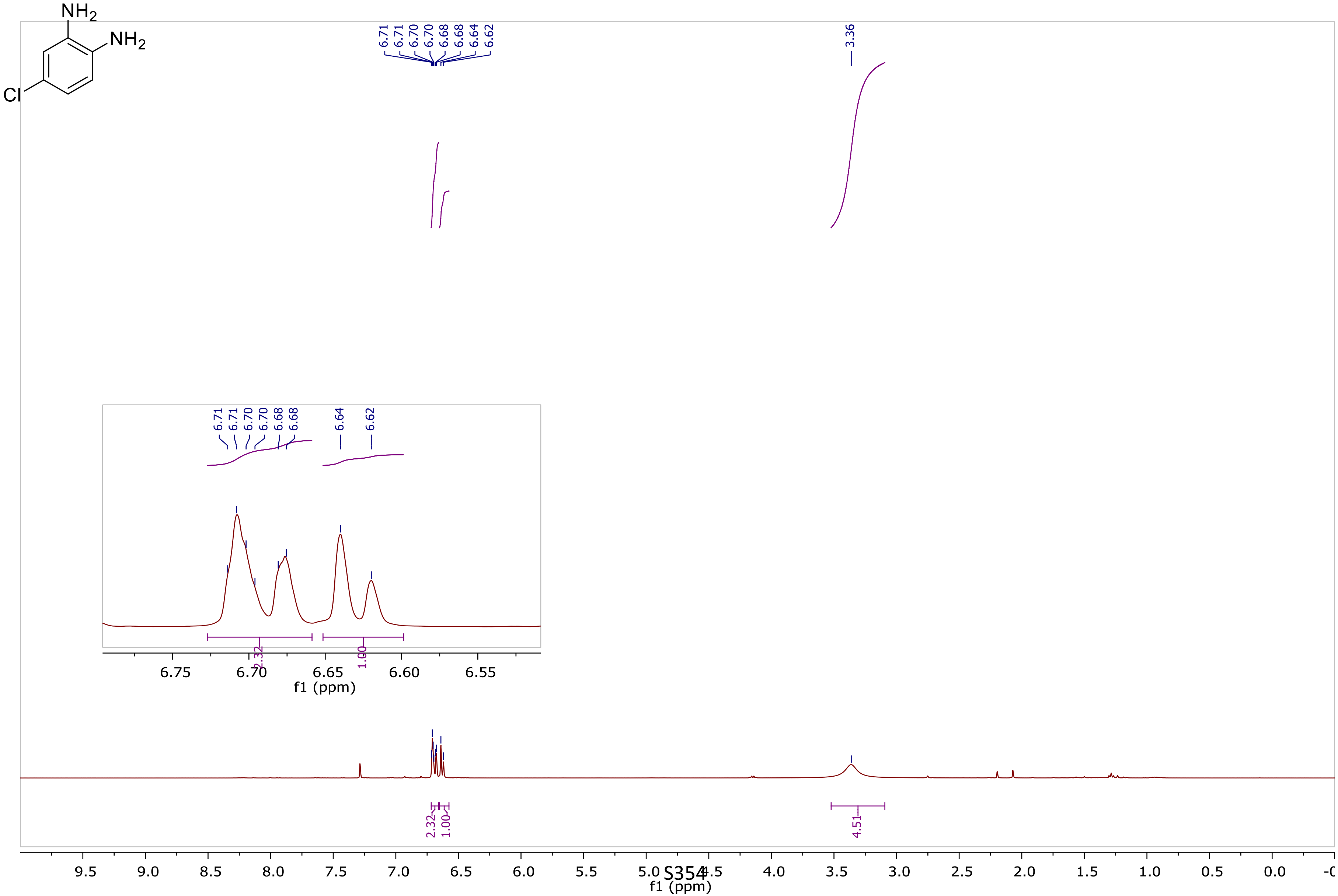

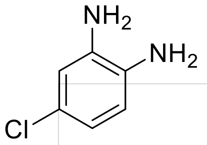

^{13}C NMR of 4-chlorobenzene-1,2-diamine **4d-ii** in $CDCl_3$

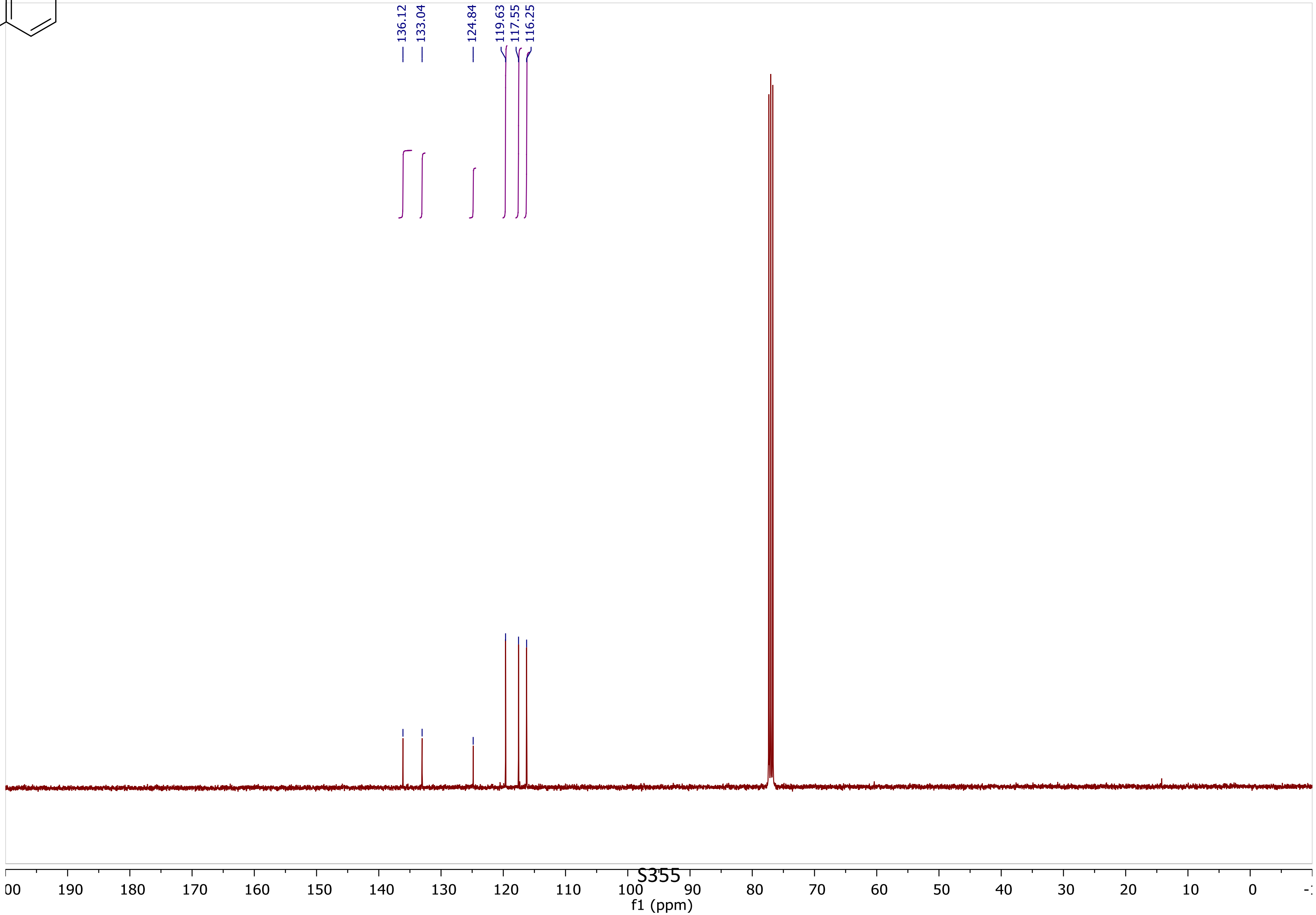

^{1}H NMR of 3-fluorobenzene-1,2-diamine **4e-i** in $CDCl_3$

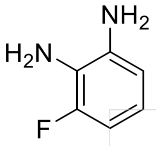

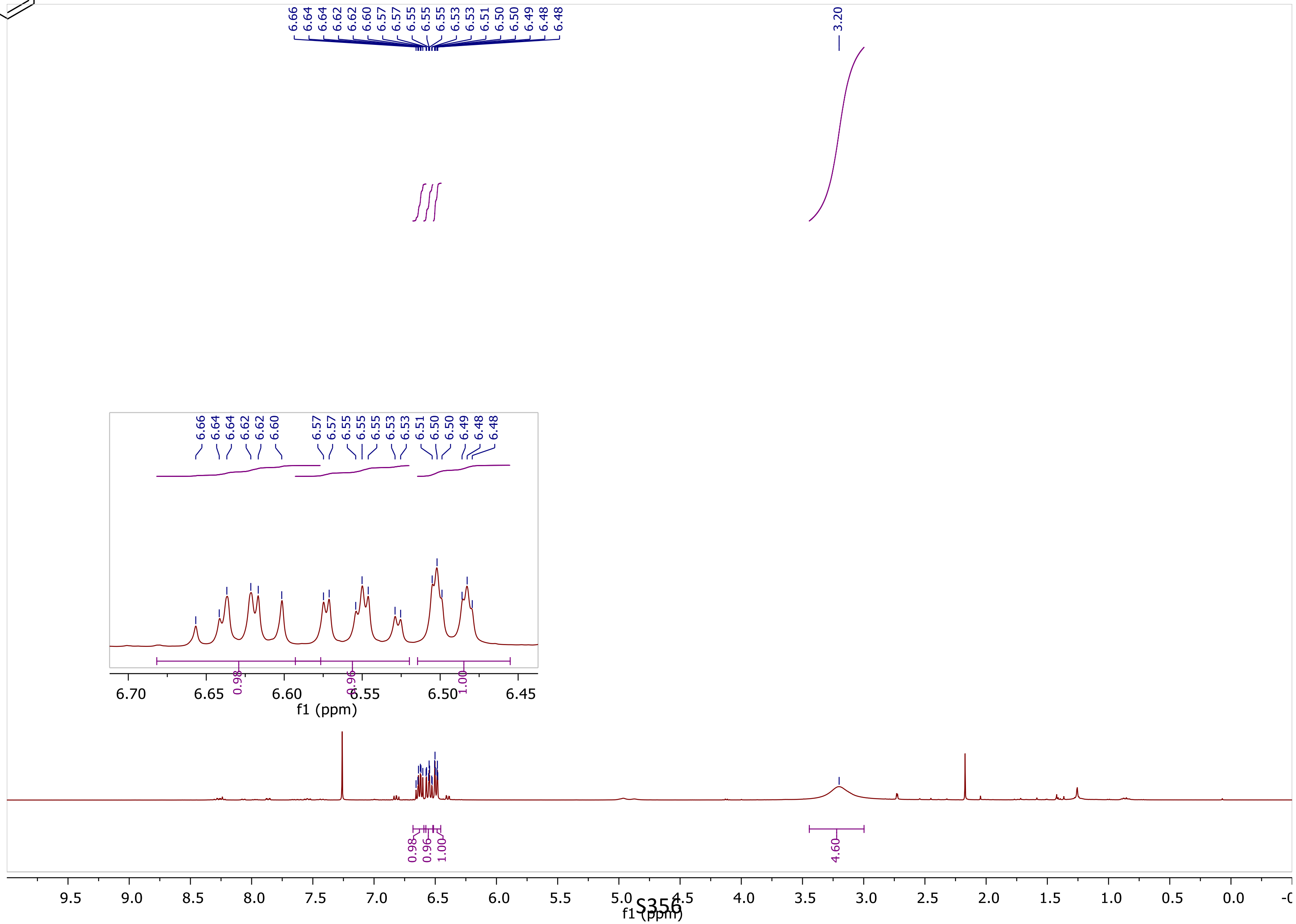

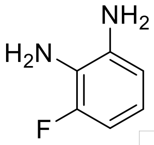

^{13}C NMR of 3-fluorobenzene-1,2-diamine **4e-i** in $CDCl_3$

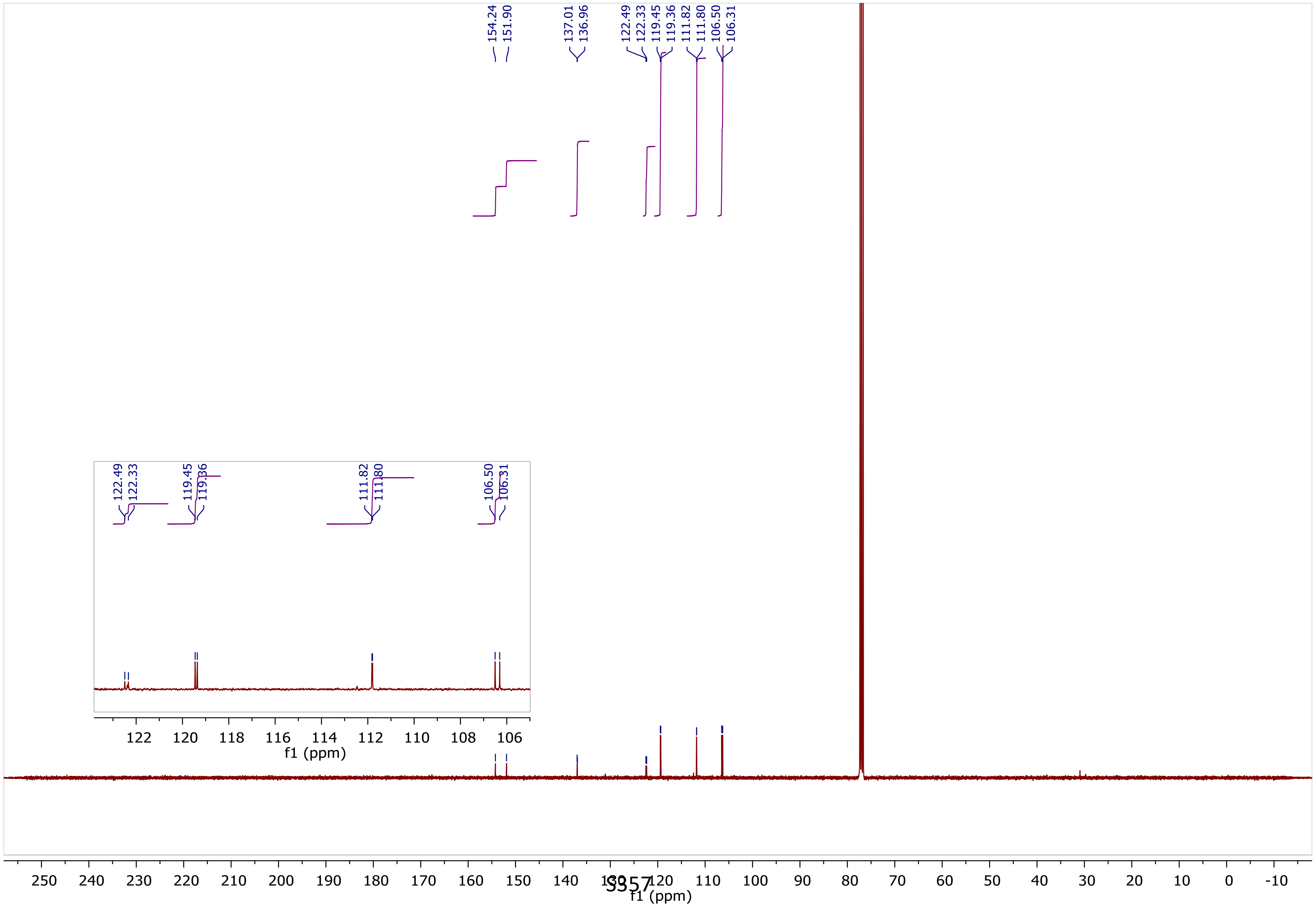

^{19}F NMR of 3-fluorobenzene-1,2-diamine **4e-i** in $CDCl_3$

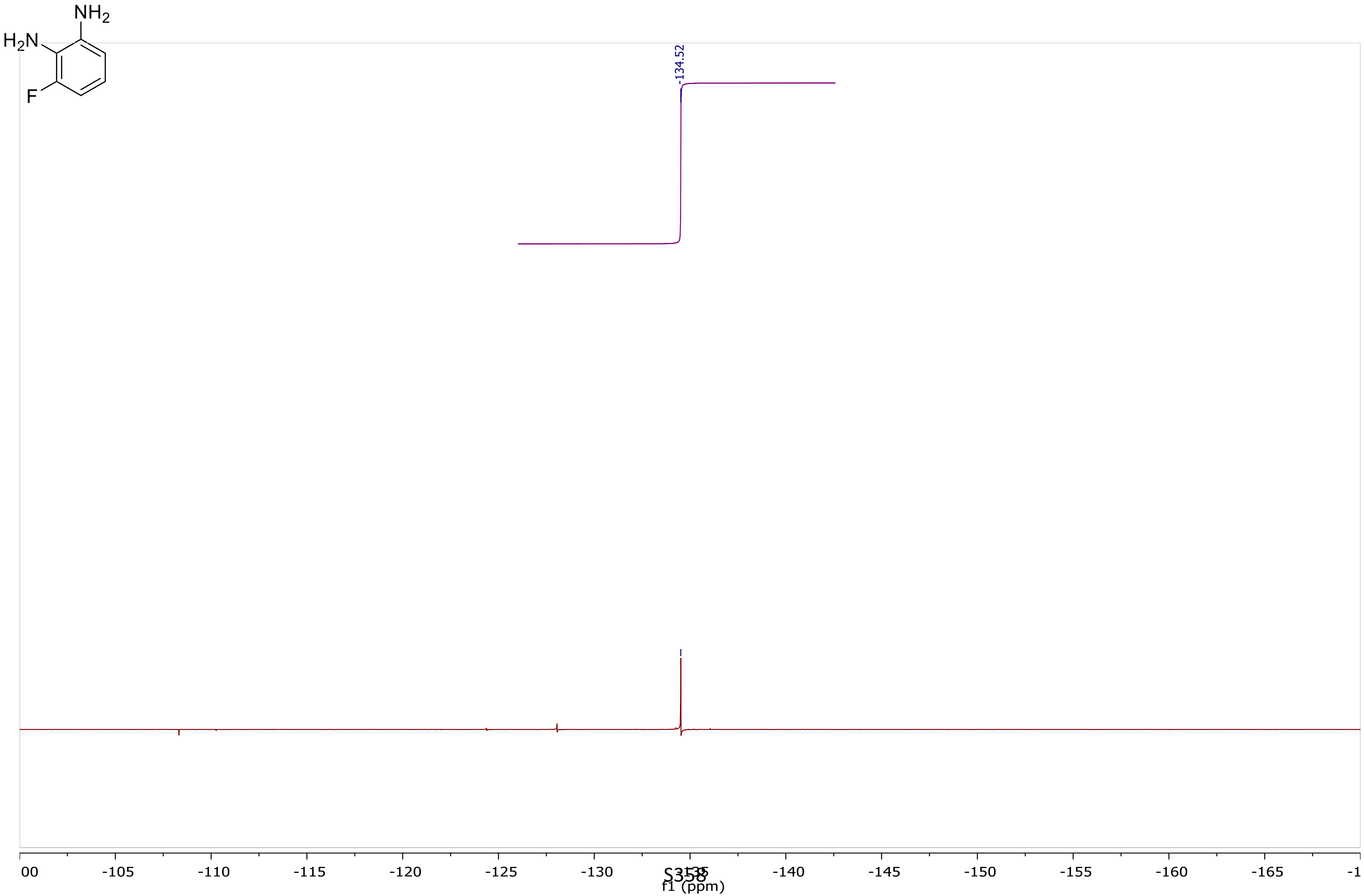

^{1}H NMR of 4-fluorobenzene-1,2-diamine **4e-ii** in $CDCl_3$

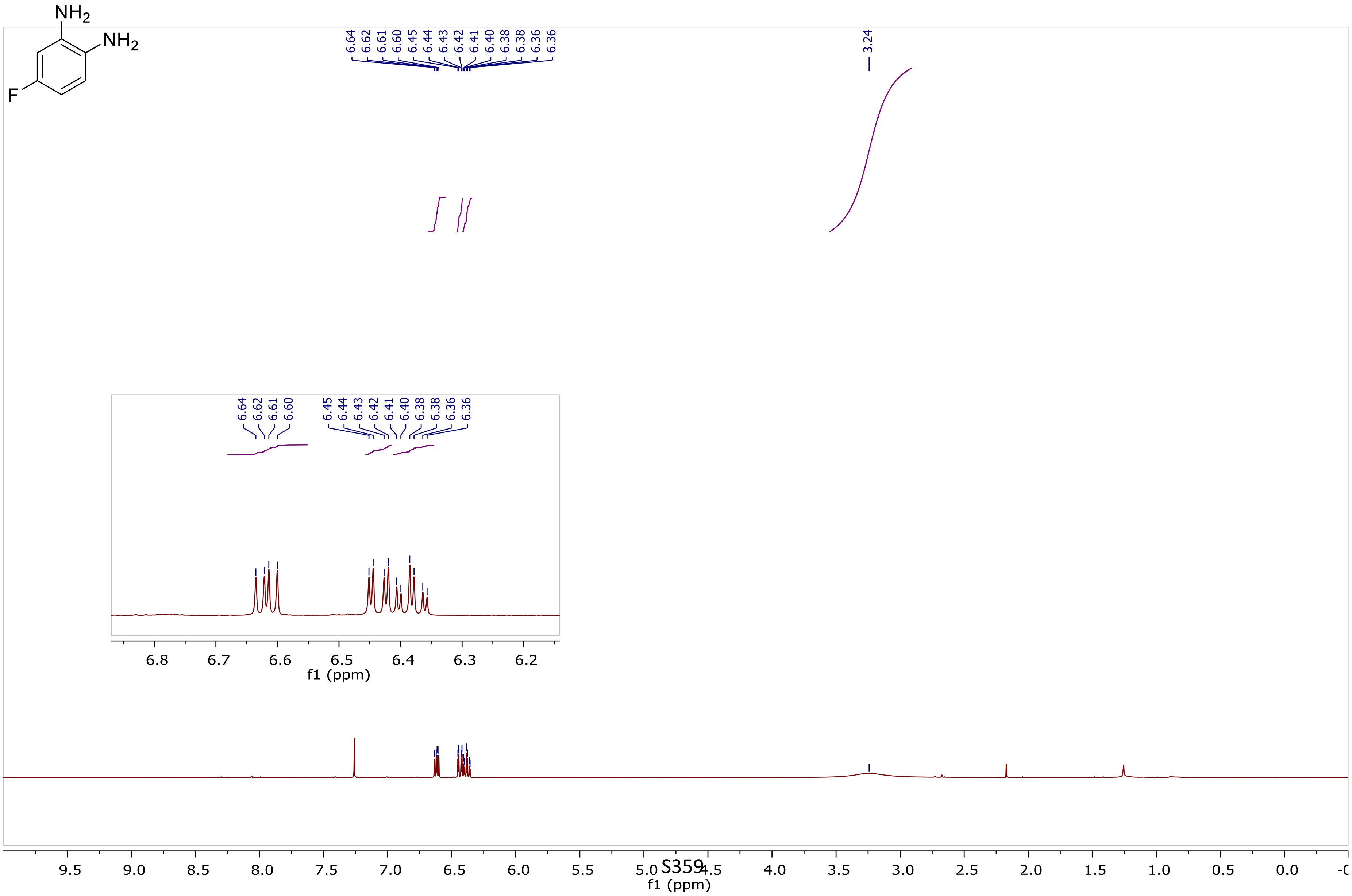

^{13}C NMR of 4-fluorobenzene-1,2-diamine **4e-ii** in $CDCl_3$

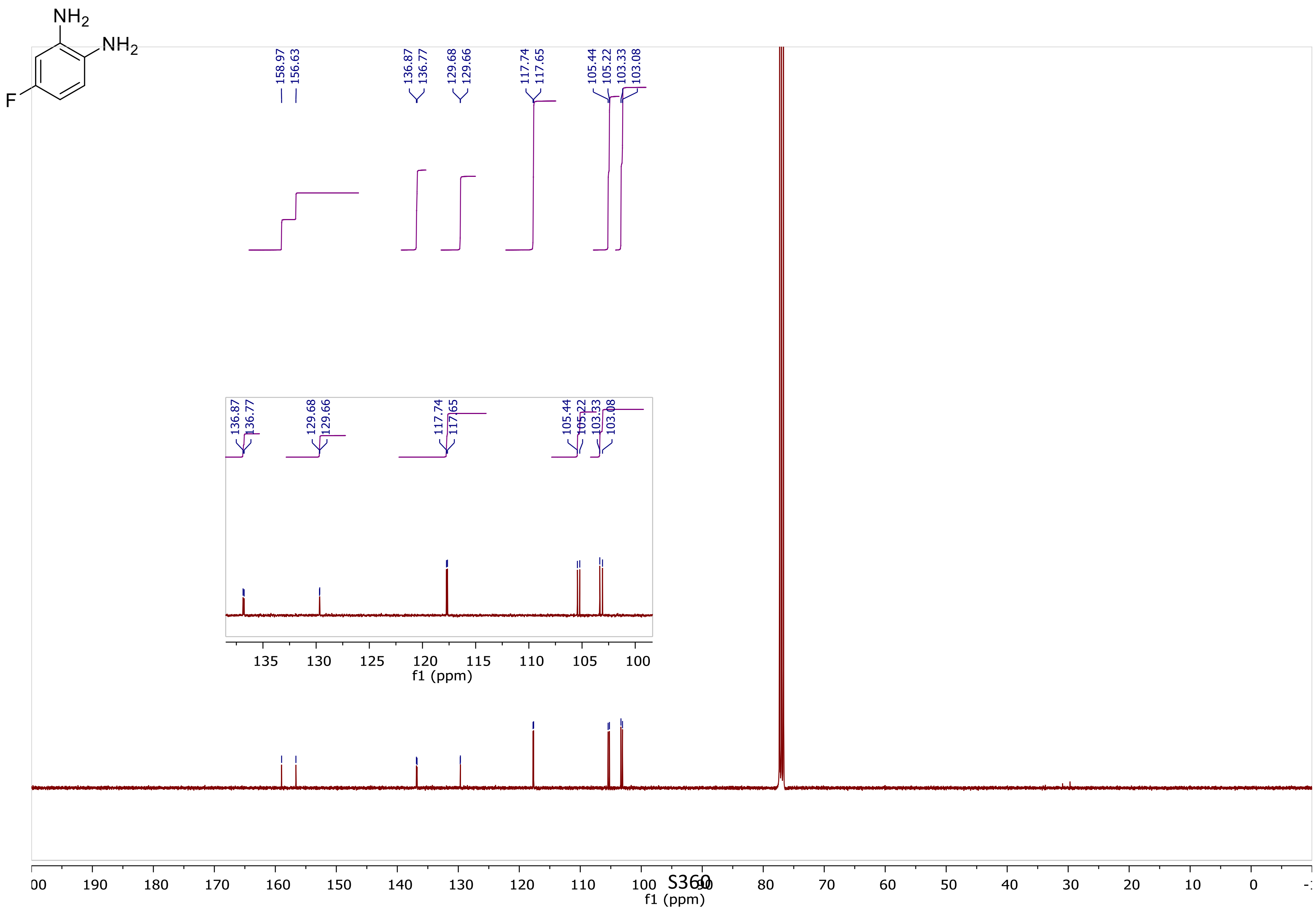

^{19}F NMR of 4-fluorobenzene-1,2-diamine **4e-ii** in $CDCl_3$

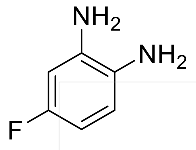

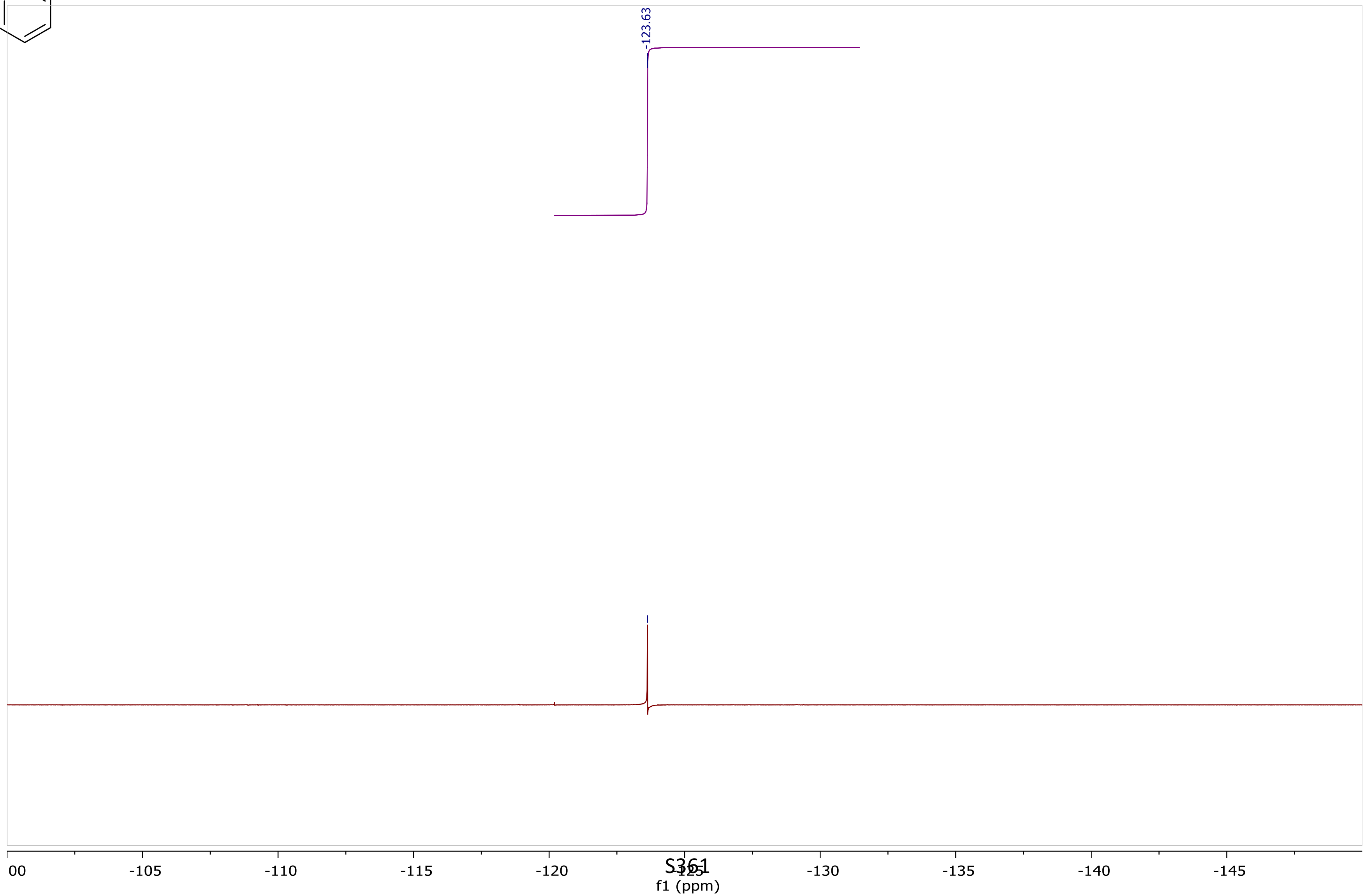

^{1}H NMR of 3-bromobenzene-1,2-diamine **4f-i** in $CDCl_3$

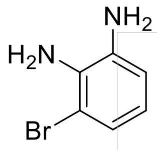

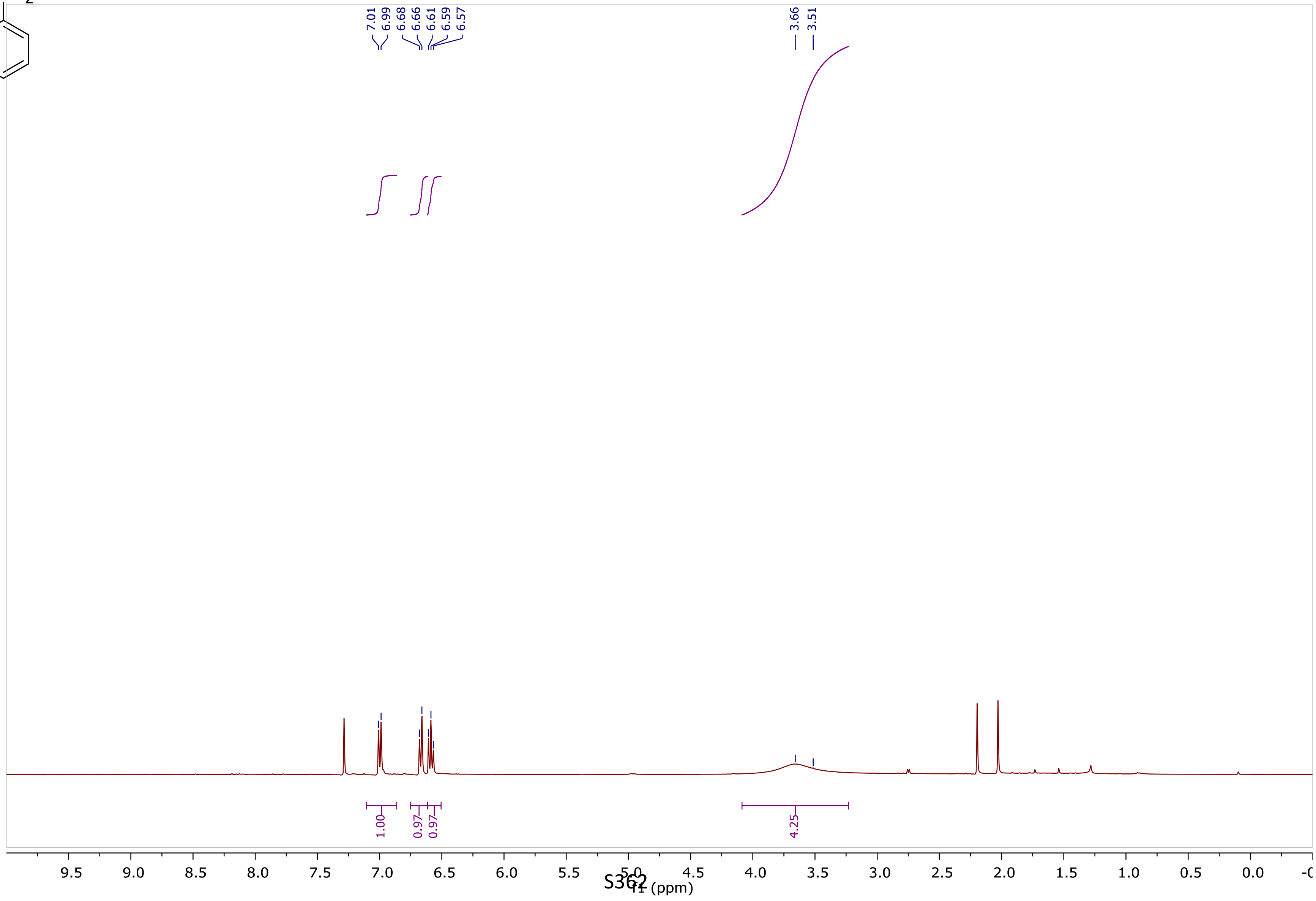

^{13}C NMR of 3-bromobenzene-1,2-diamine **4f-i** in $CDCl_3$

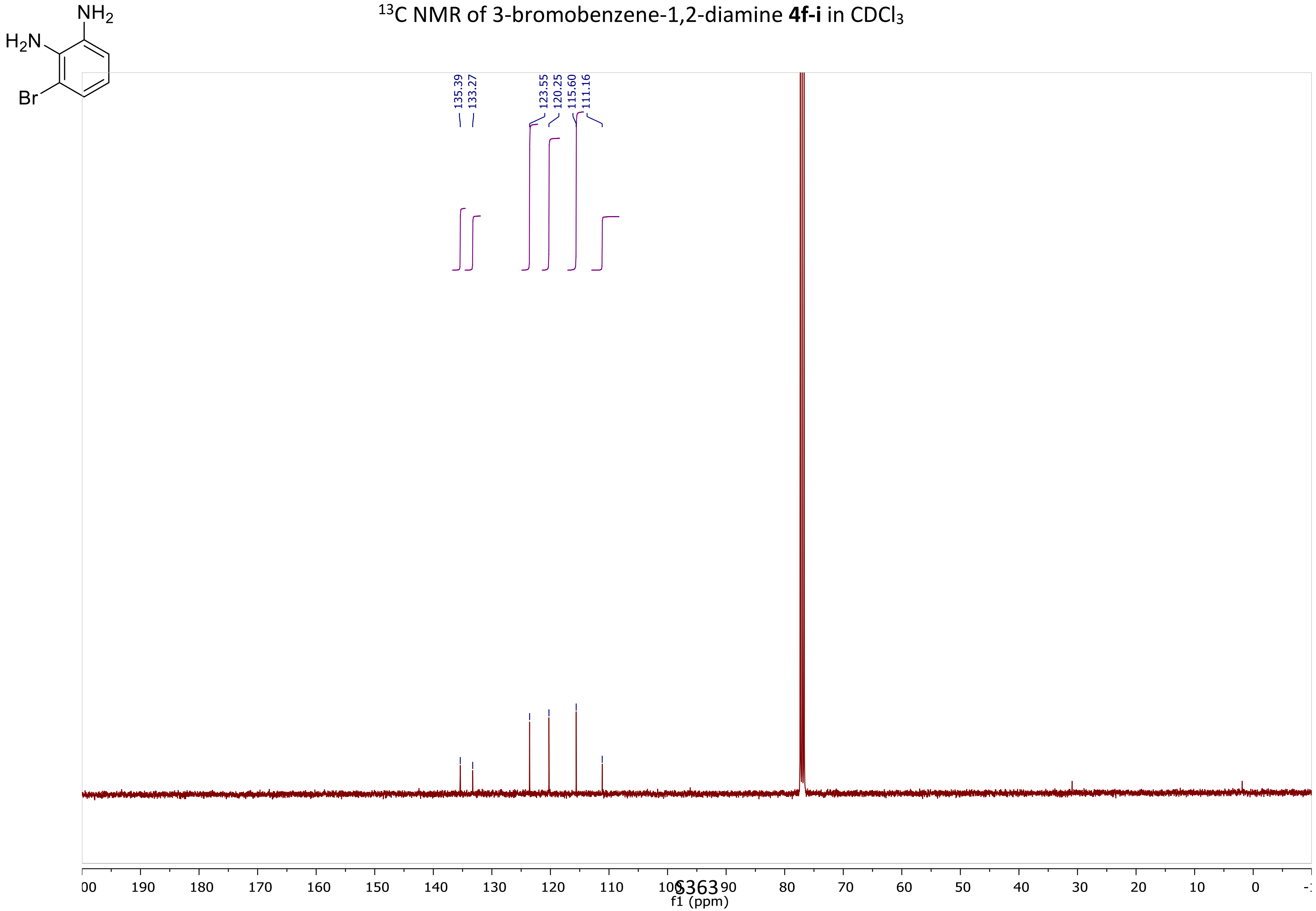

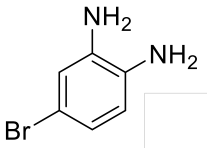

^{1}H NMR of 4-bromobenzene-1,2-diamine **4f-ii** in $CDCl_3$

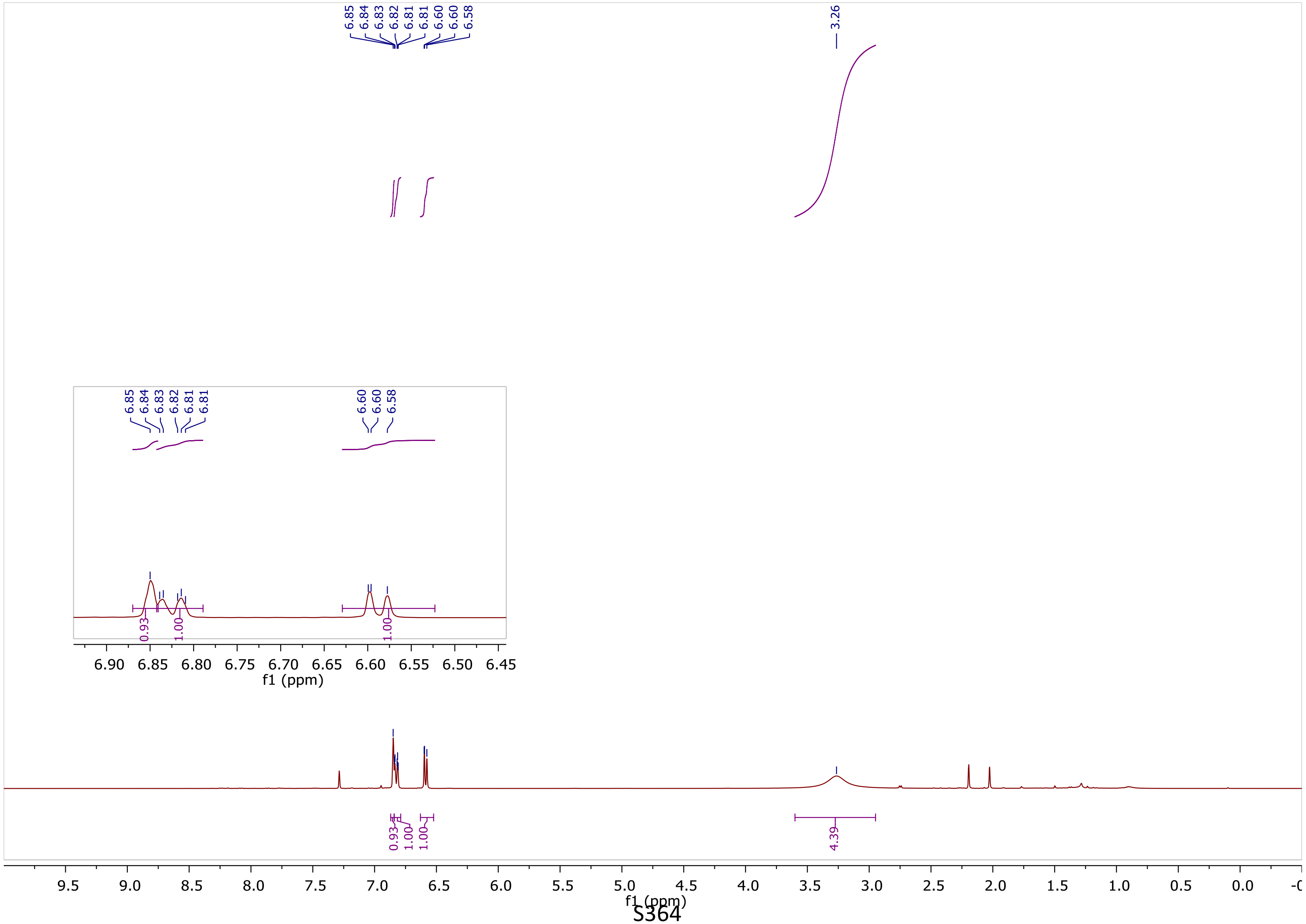

^{13}C NMR of 4-bromobenzene-1,2-diamine **4f-ii** in $CDCl_3$

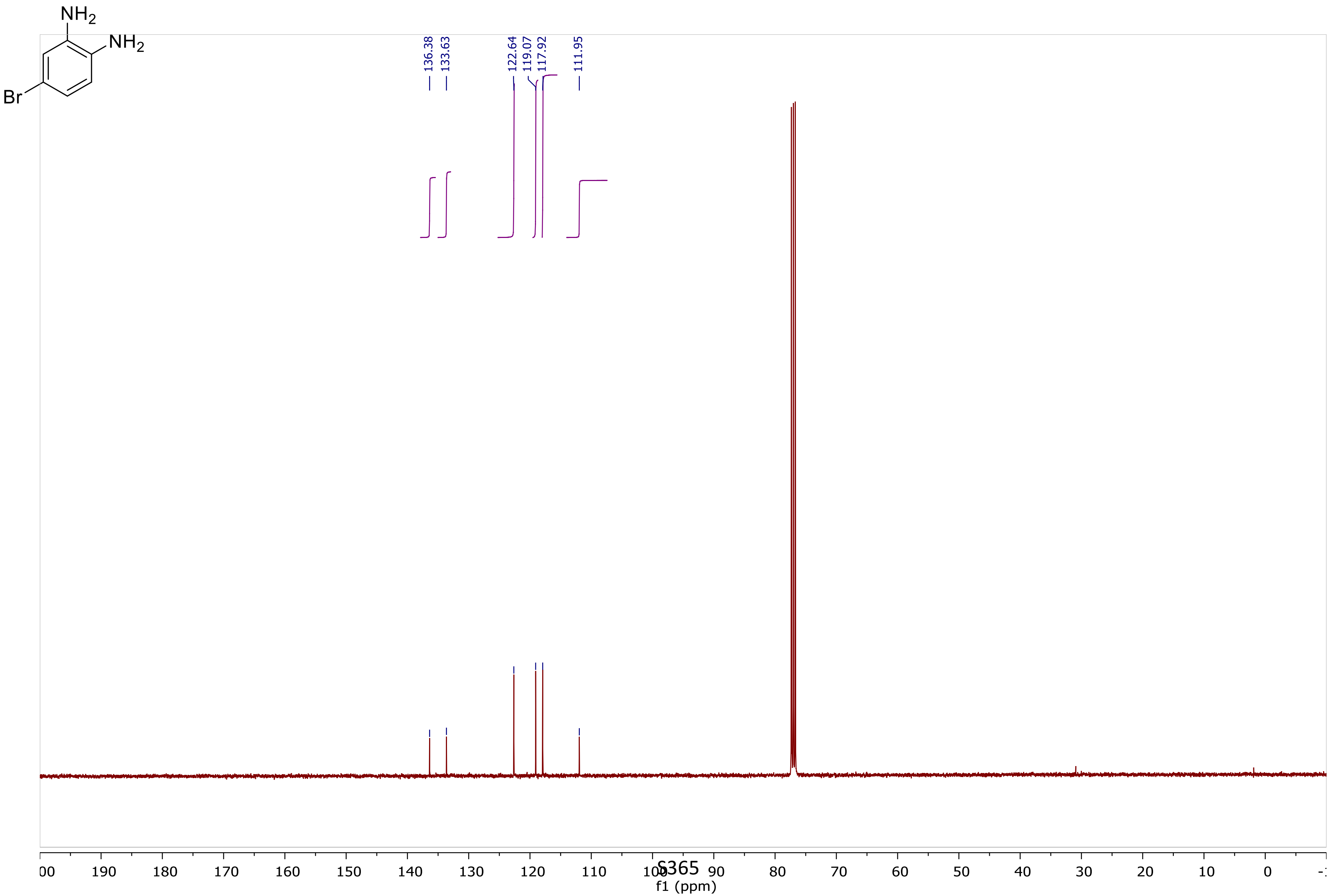

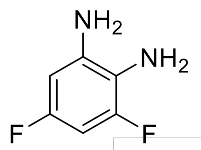

^{1}H NMR of 3,5-difluorobenzene-1,2-diamine **4g** in $CDCl_3$

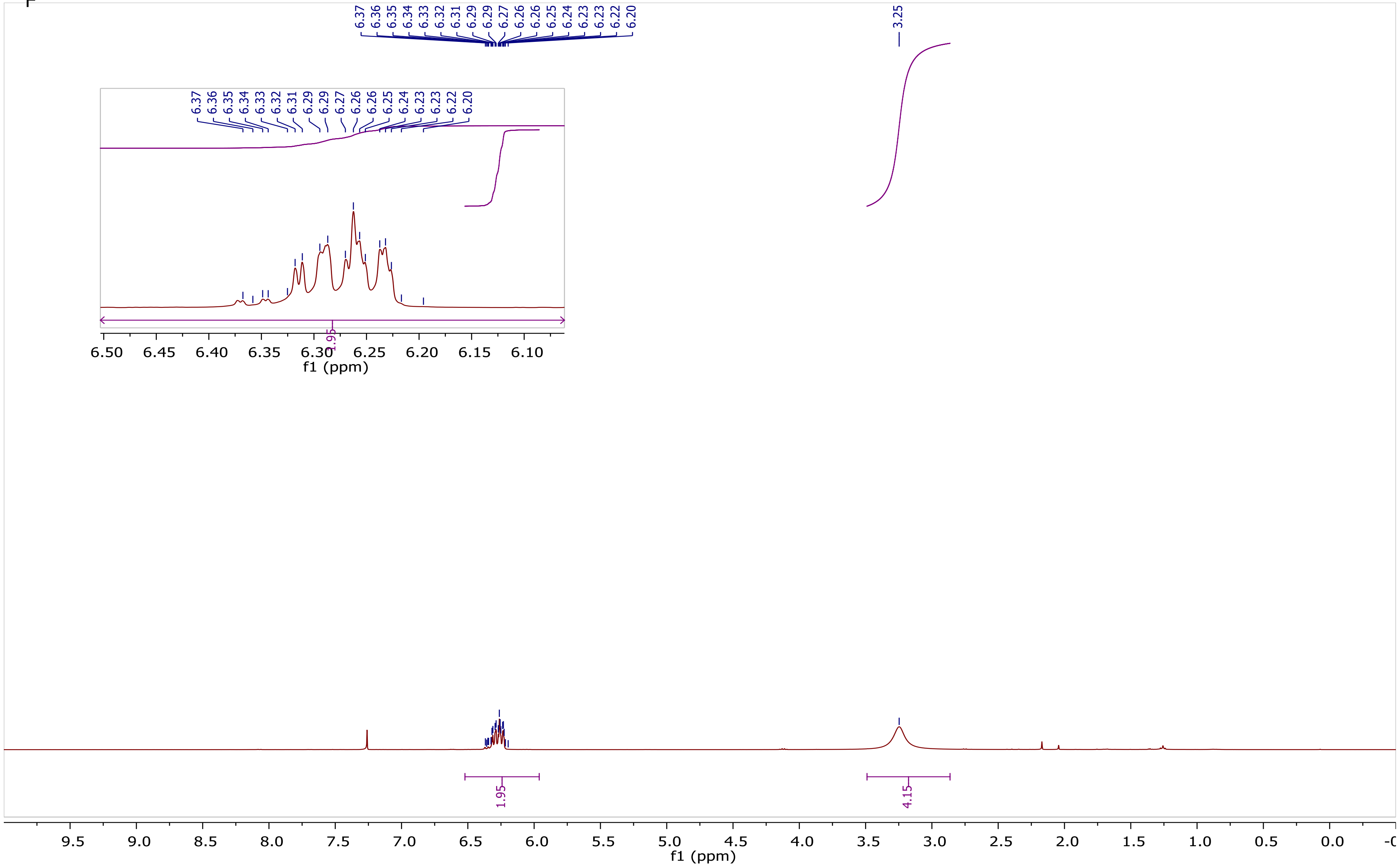

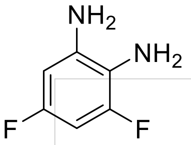

^{13}C NMR of 3,5-difluorobenzene-1,2-diamine **4g** in $CDCl_3$

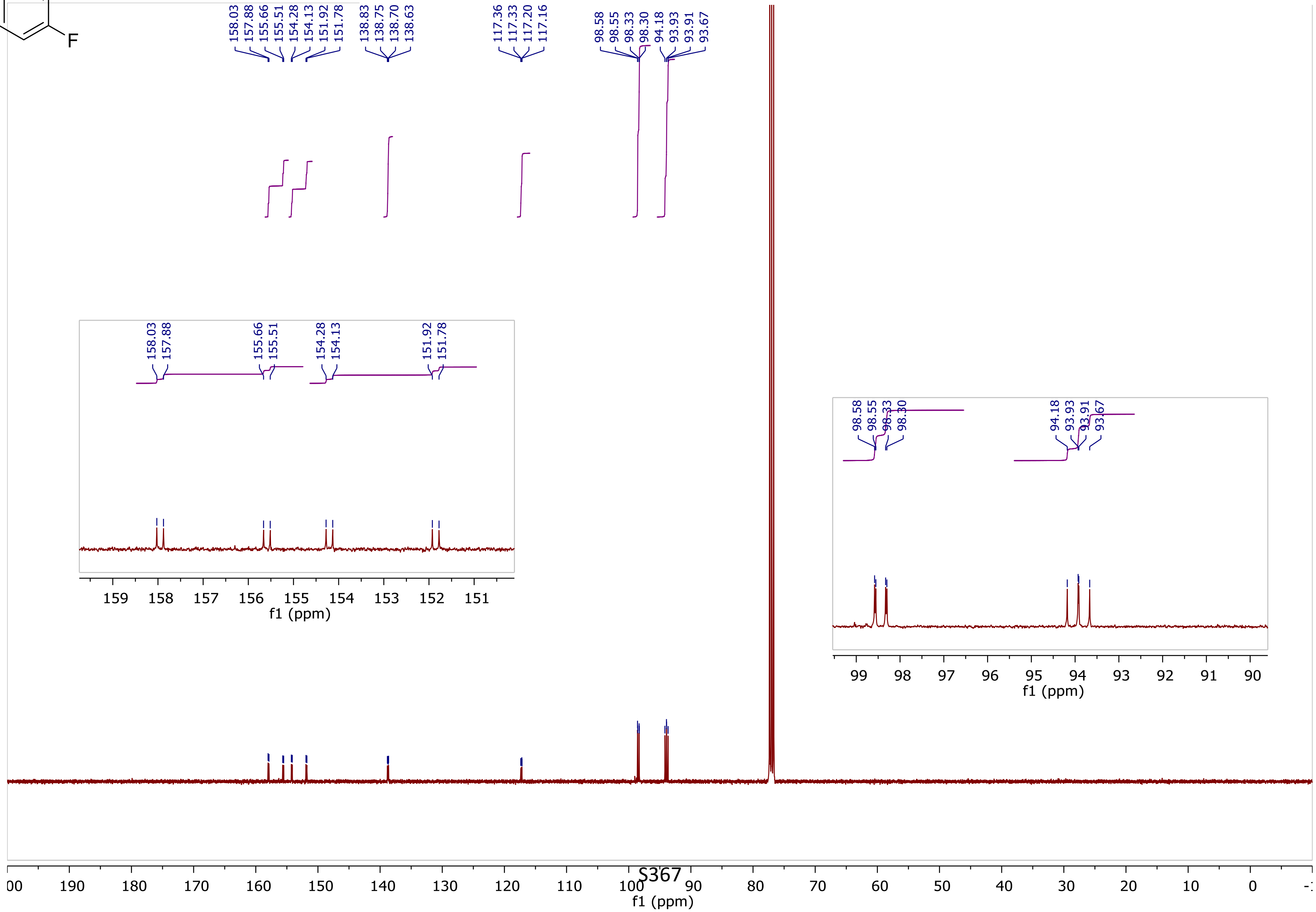

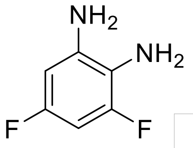

^{19}F NMR of 3,5-difluorobenzene-1,2-diamine **4g** in $CDCl_3$

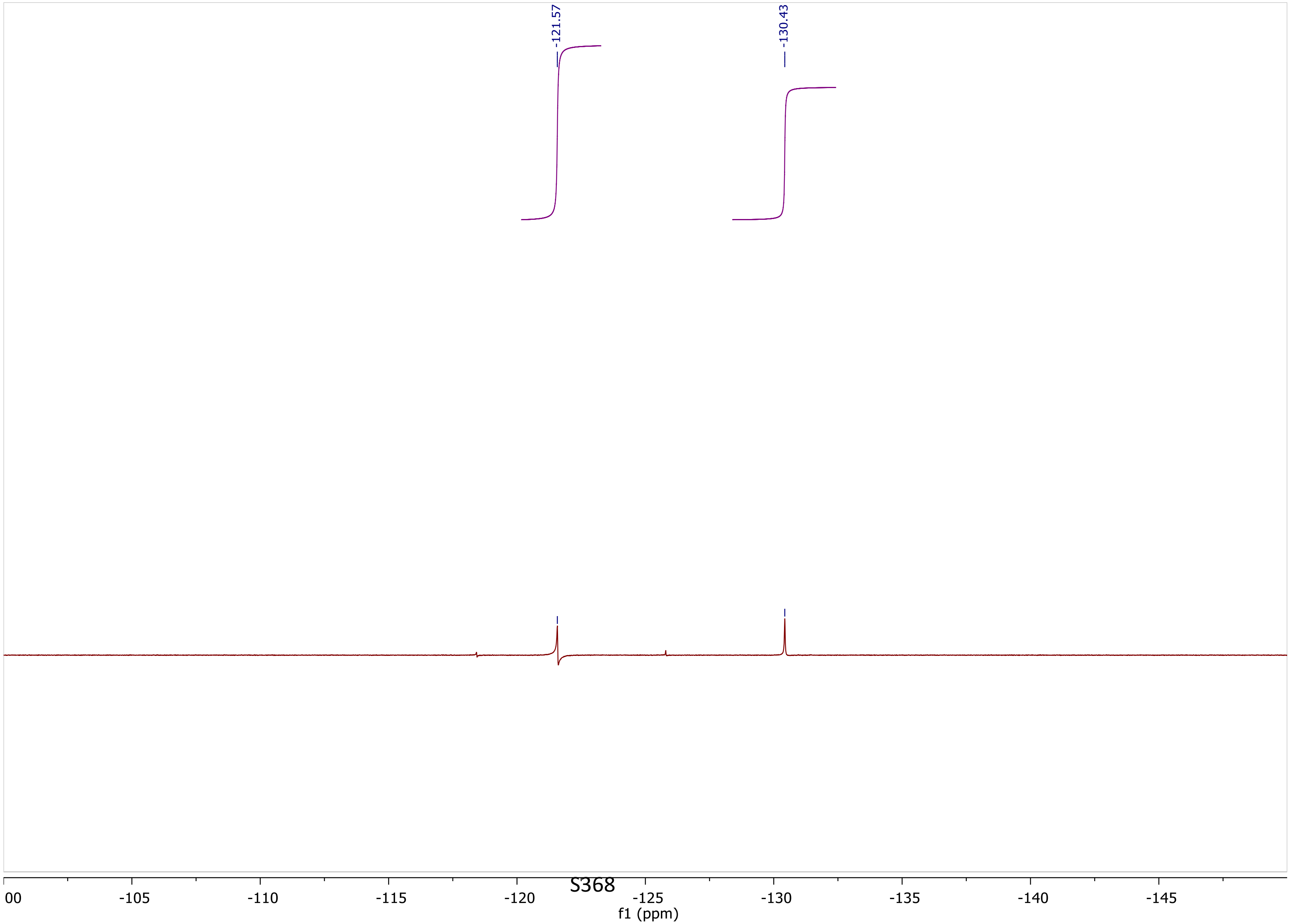

^{1}H NMR of 4-methoxy-3-methylbenzene-1,2-diamine **4h-i** in $CDCl_3$

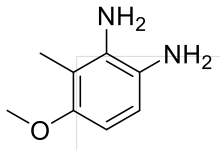

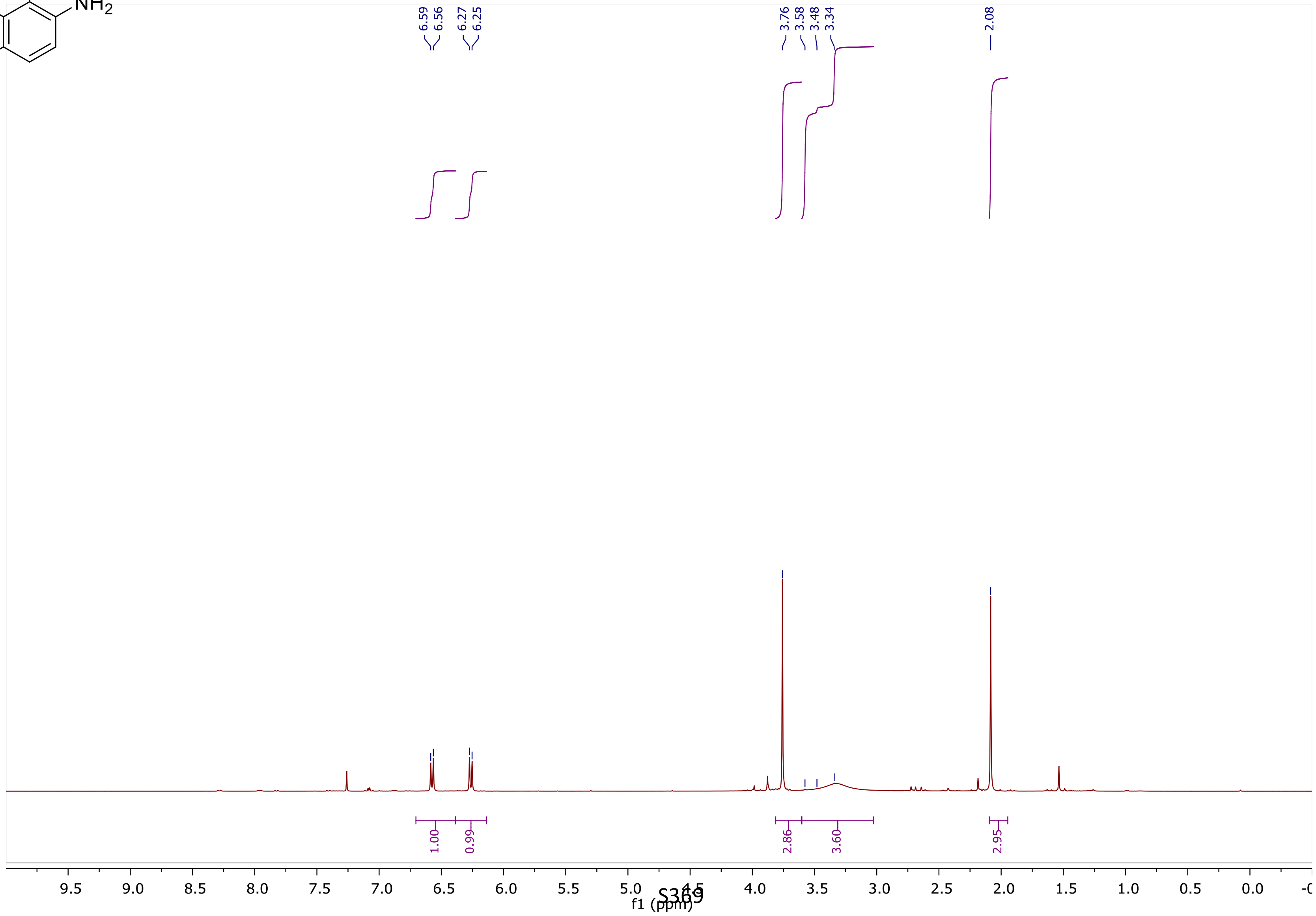

^{13}C NMR of 4-methoxy-3-methylbenzene-1,2-diamine **4h-i** in $CDCl_3$

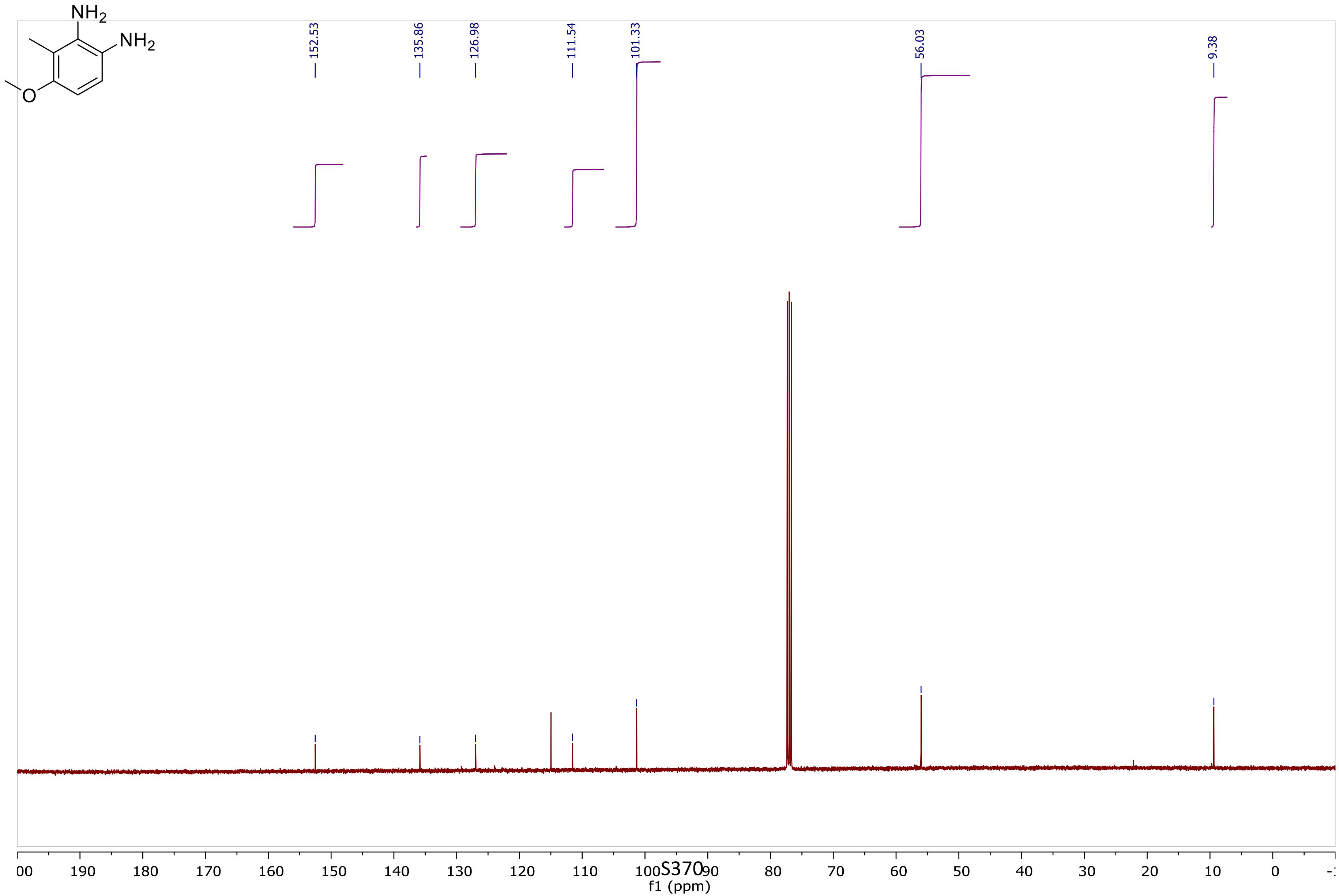

^{1}H NMR of 5-methoxy-4-methyl-1*H*-benzo[d]imidazole **4h-ii** in $CDCl_3$

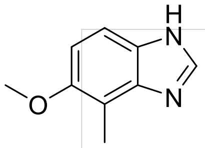

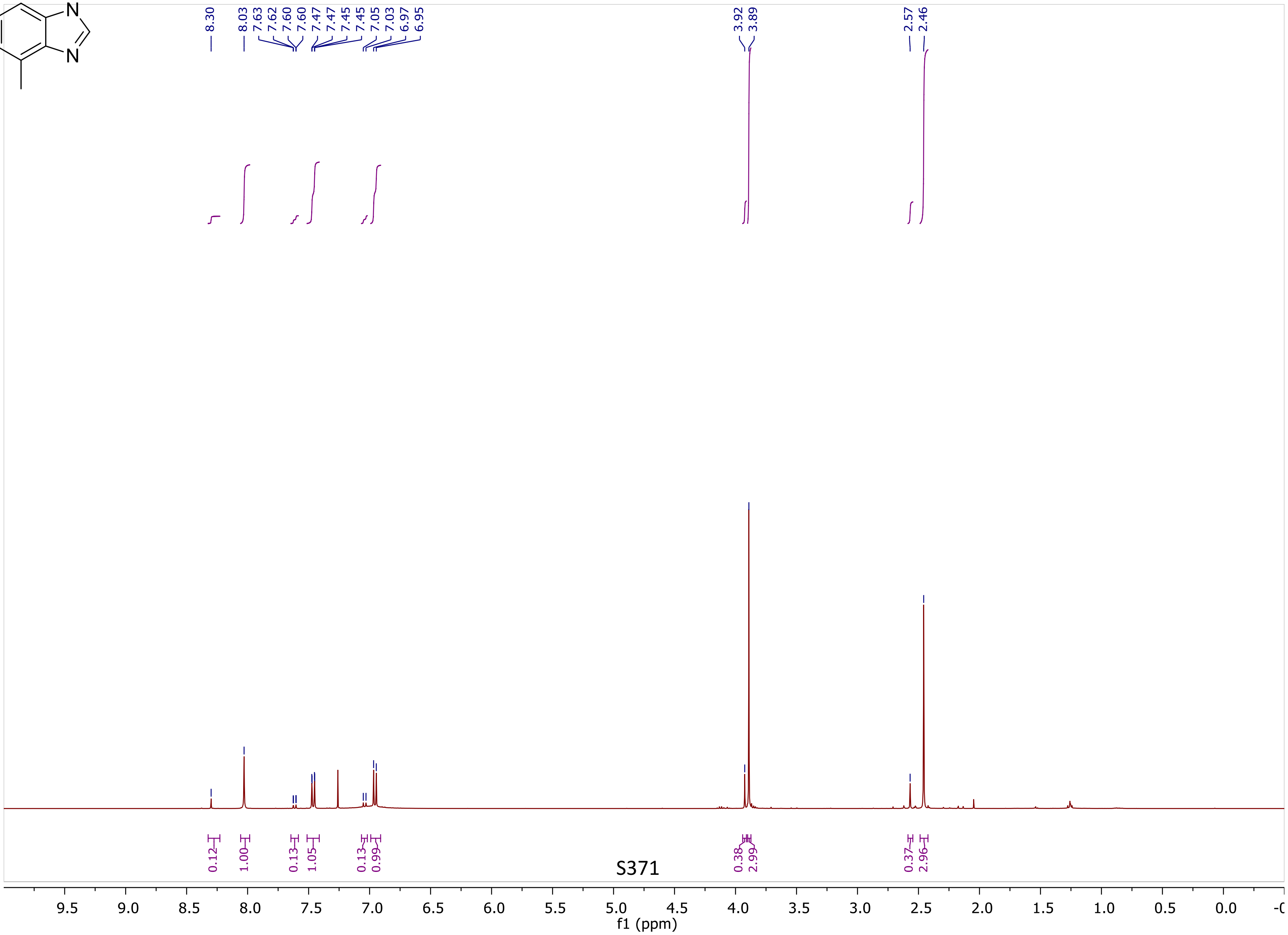

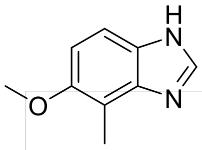

^{13}C NMR of 5-methoxy-4-methyl-1*H*-benzo[d]imidazole **4h-ii** in $CDCl_3$

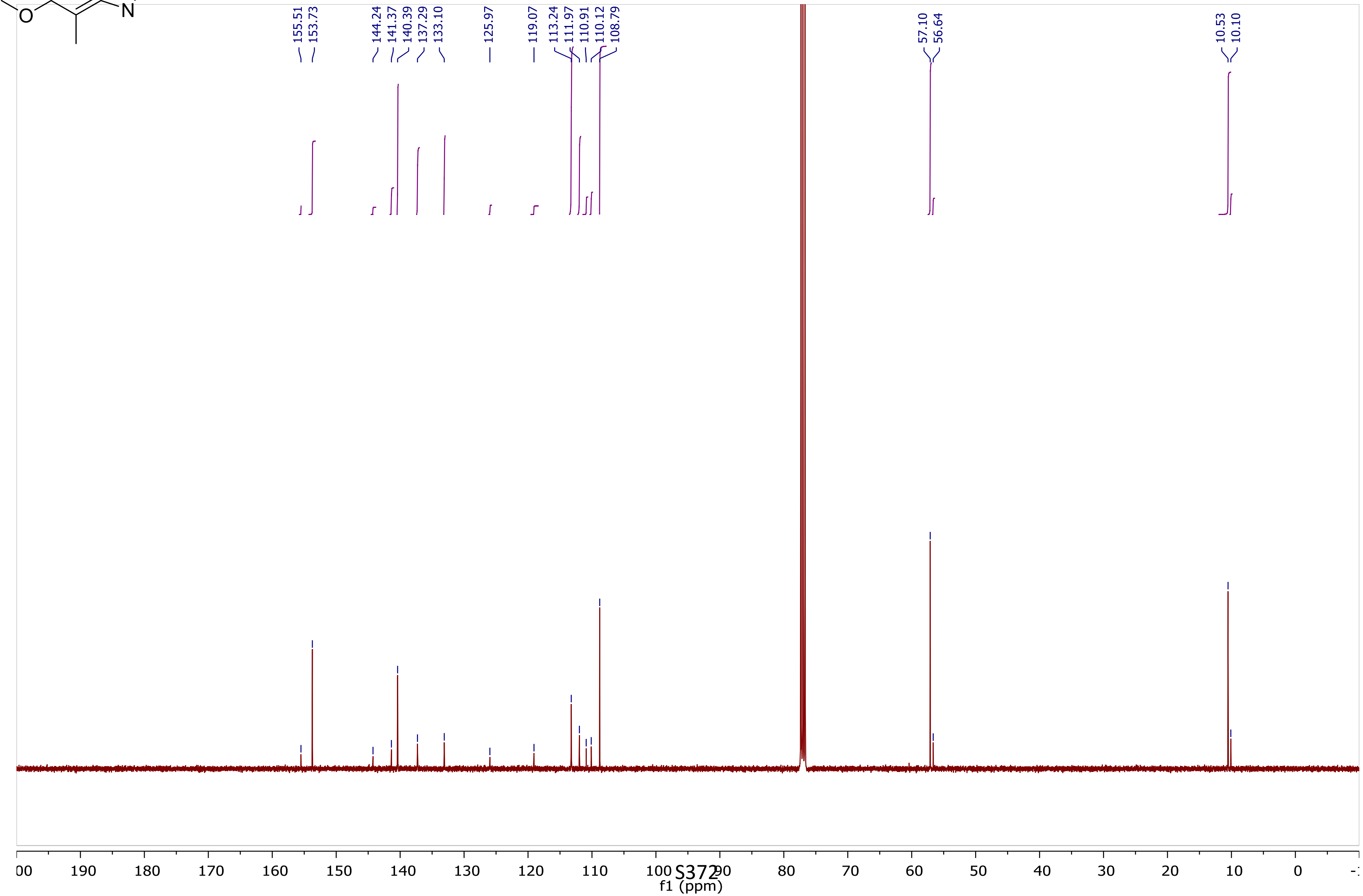

^{1}H NMR of 3-bromo-4-methylbenzene-1,2-diamine **4i** in MeOD-d[4]

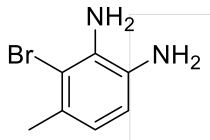

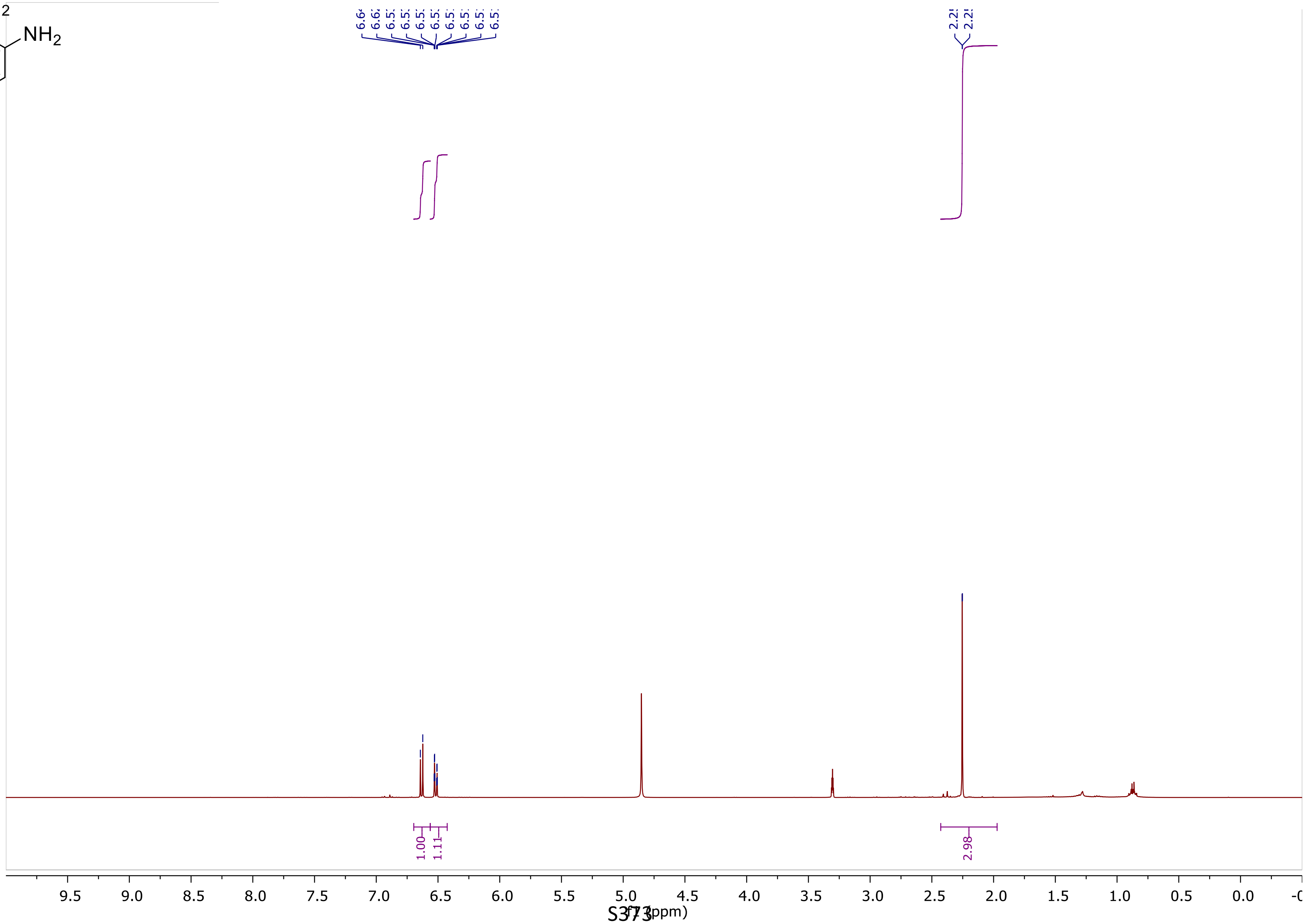

^{1}H NMR of 3-bromo-4-methylbenzene-1,2-diamine **4i** in MeOD-d[4]

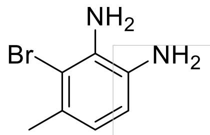

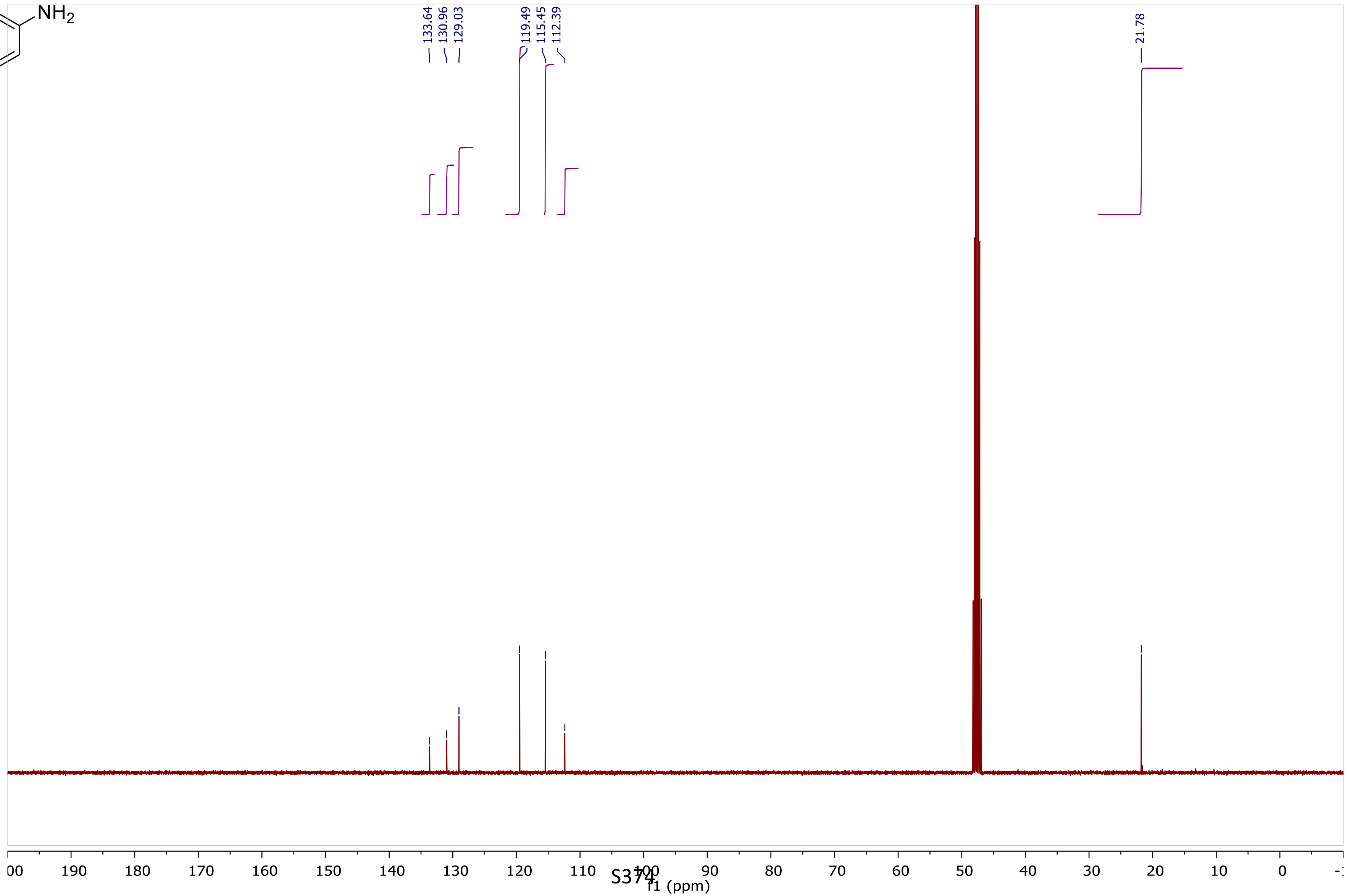

^{1}H NMR of *N*1-benzylbenzene-1,2-diamine **4j** in $CDCl_3$

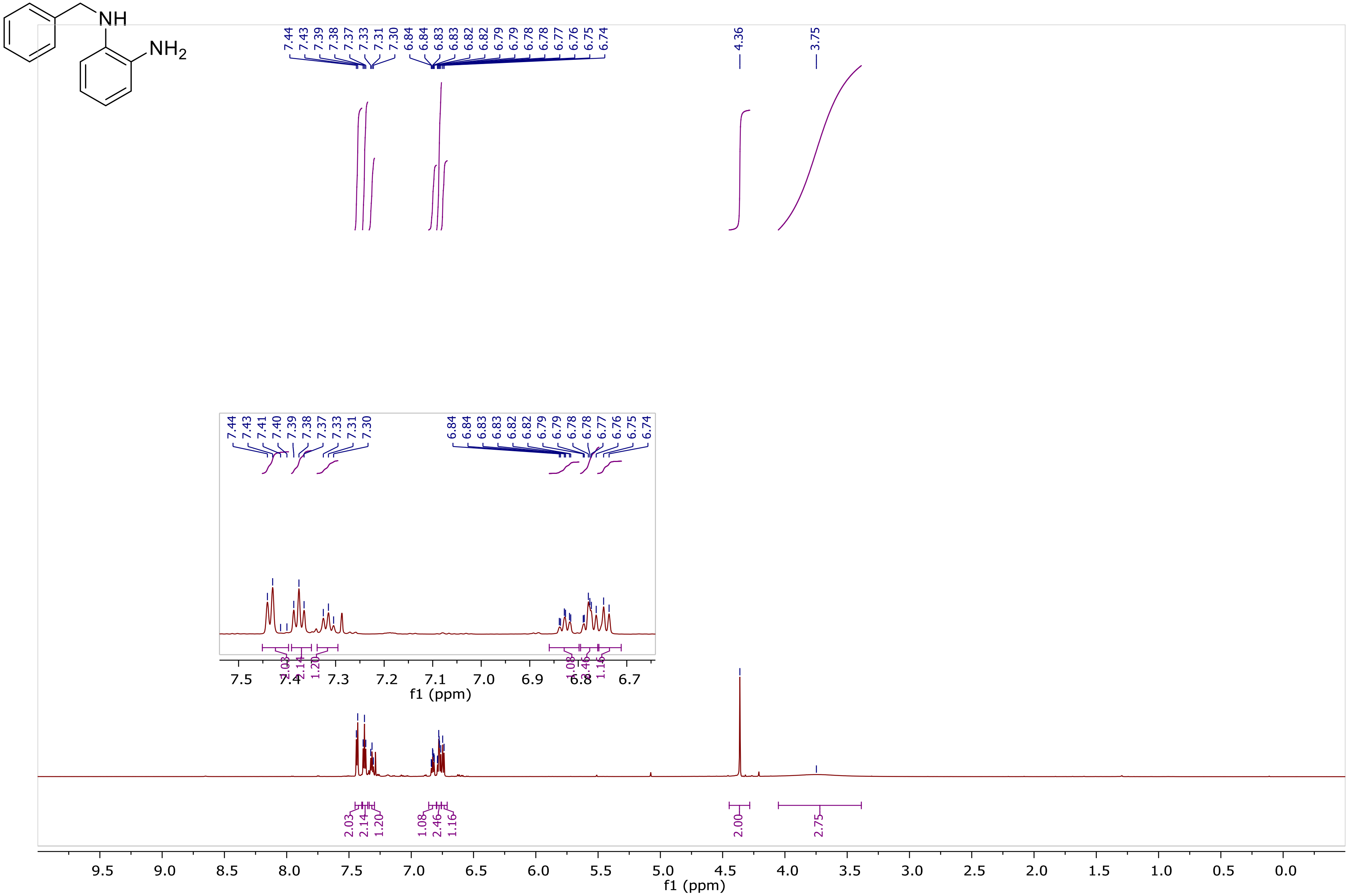

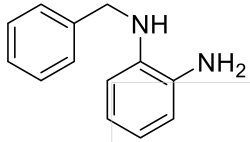

^{13}C NMR of *N*1-benzylbenzene-1,2-diamine **4j** in $CDCl_3$

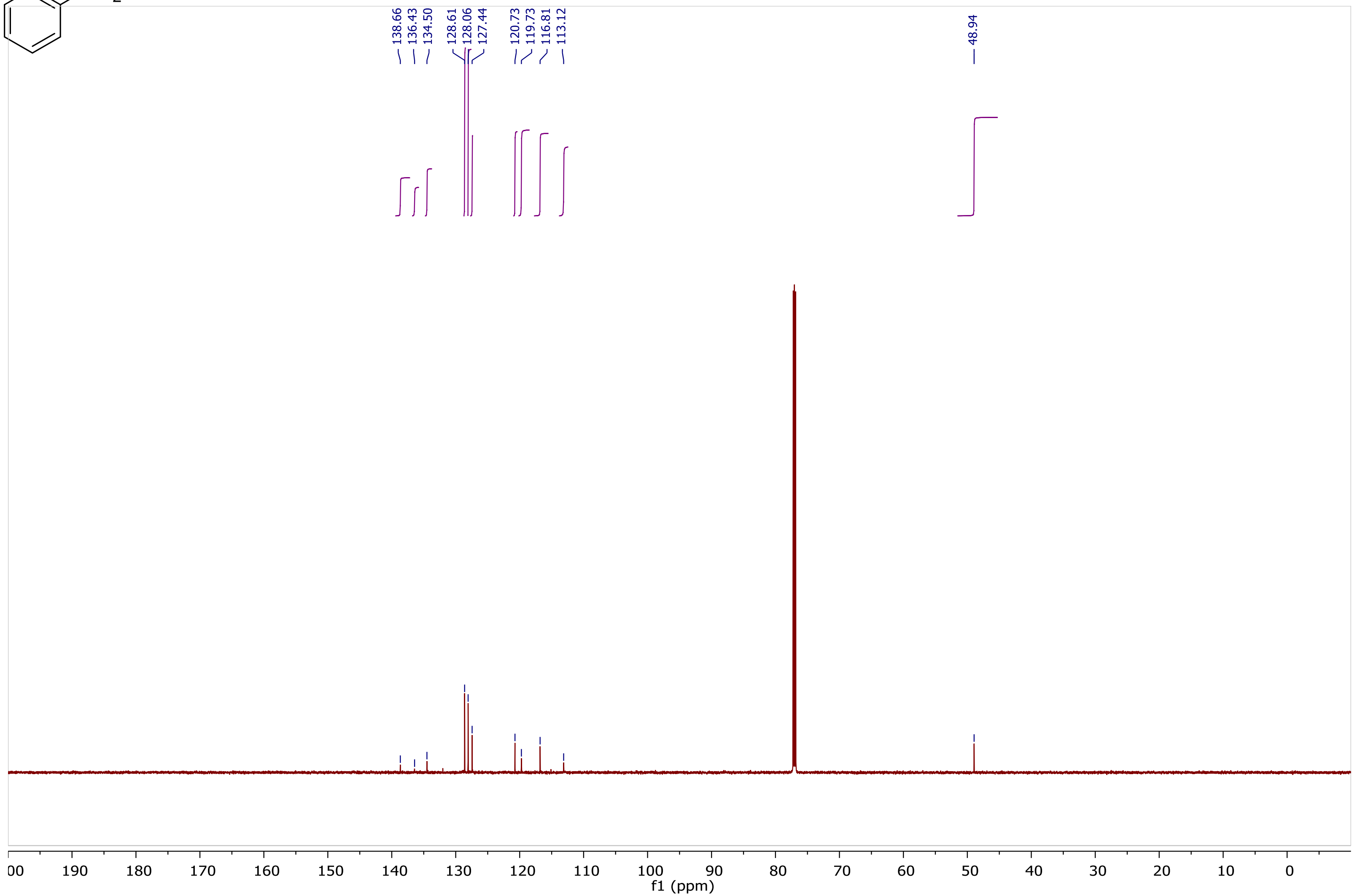

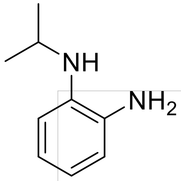

^{1}H NMR of *N*1-isopropylbenzene-1,2-diamine **4k** in $CDCl_3$

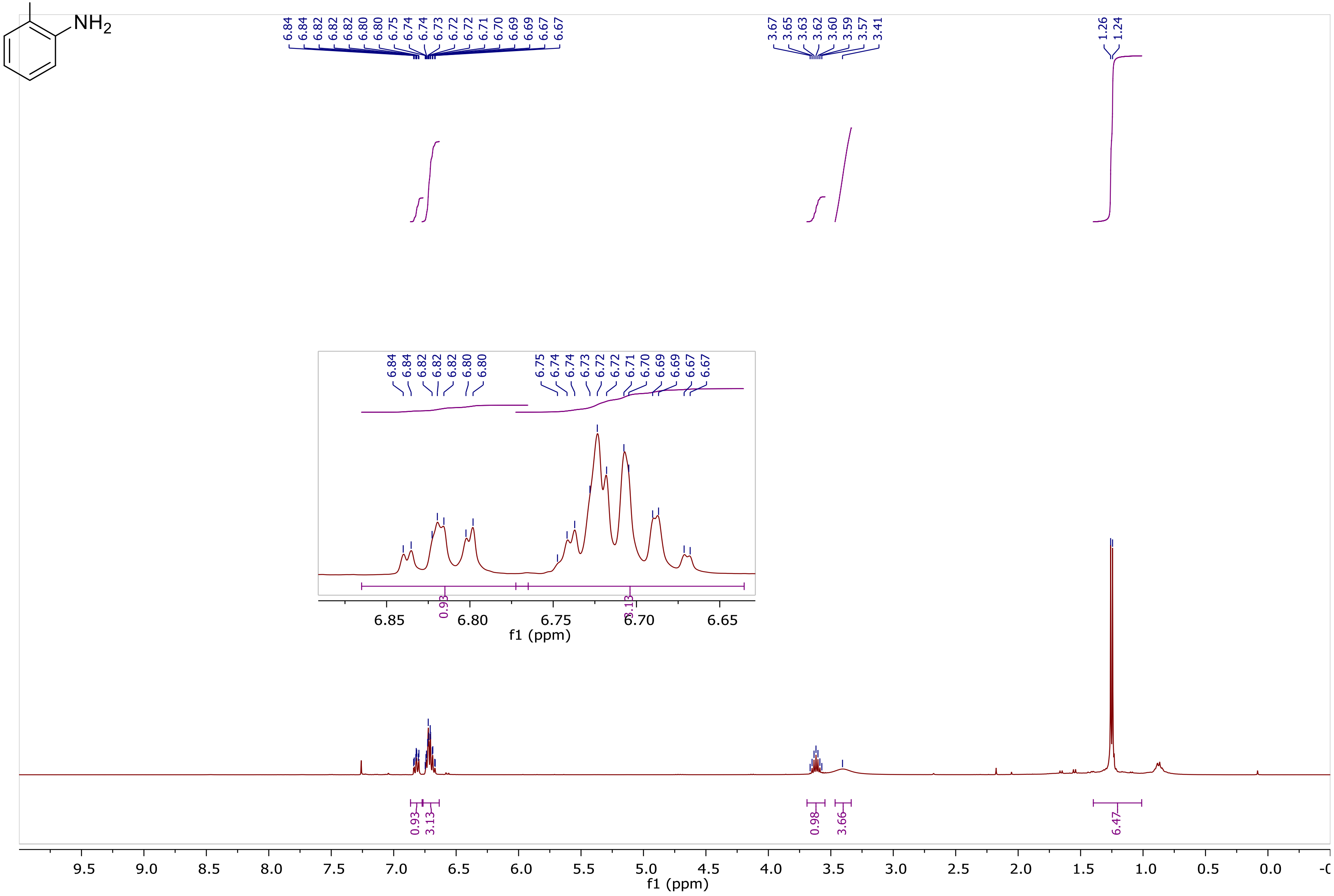

^{13}C NMR of *N*1-benzylbenzene-1,2-diamine **4k** in $CDCl_3$

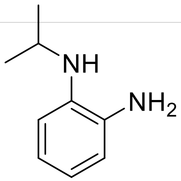

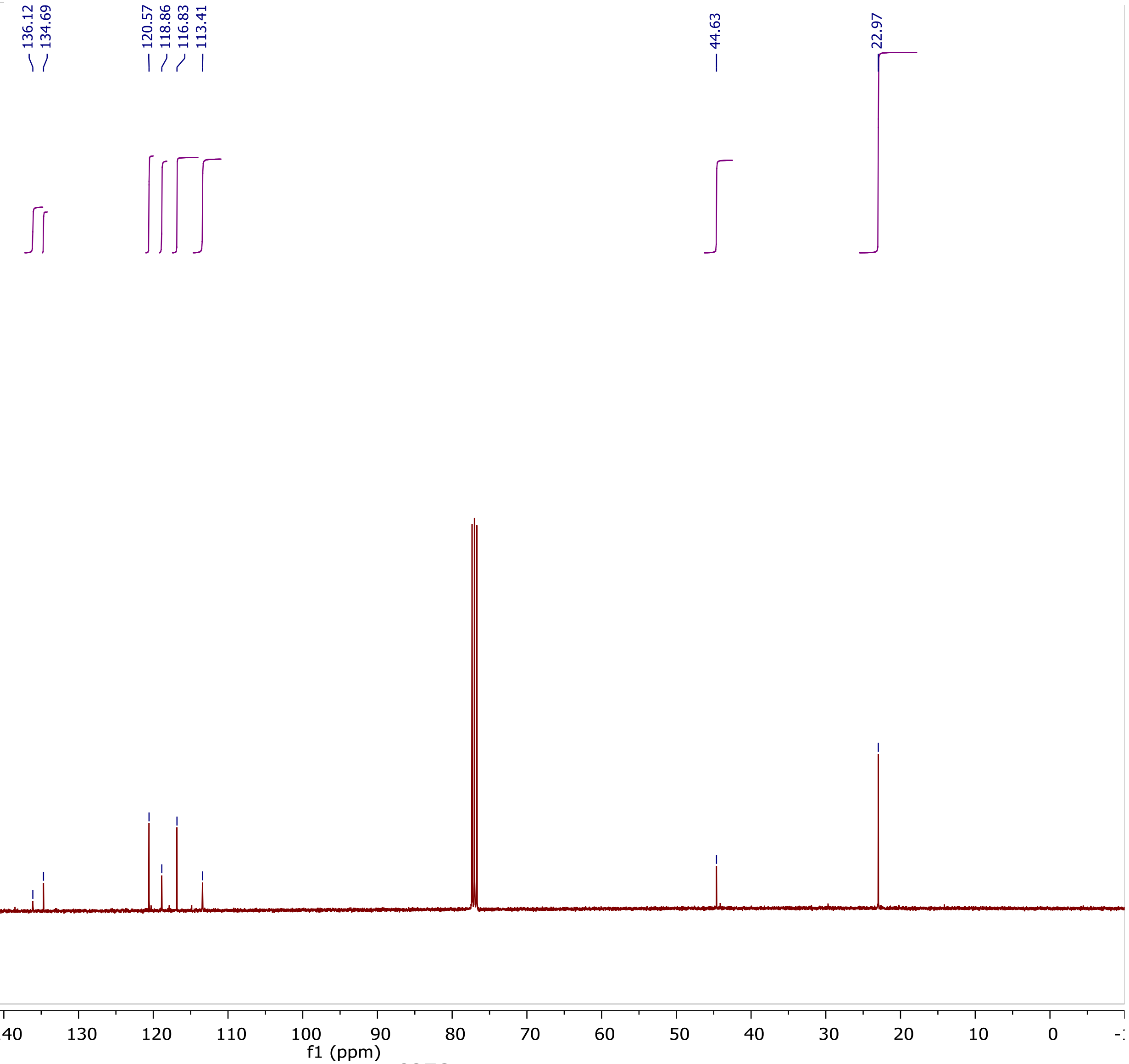

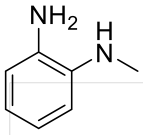

^{1}H NMR of *N*1-methylbenzene-1,2-diamine **4l** in $CDCl_3$

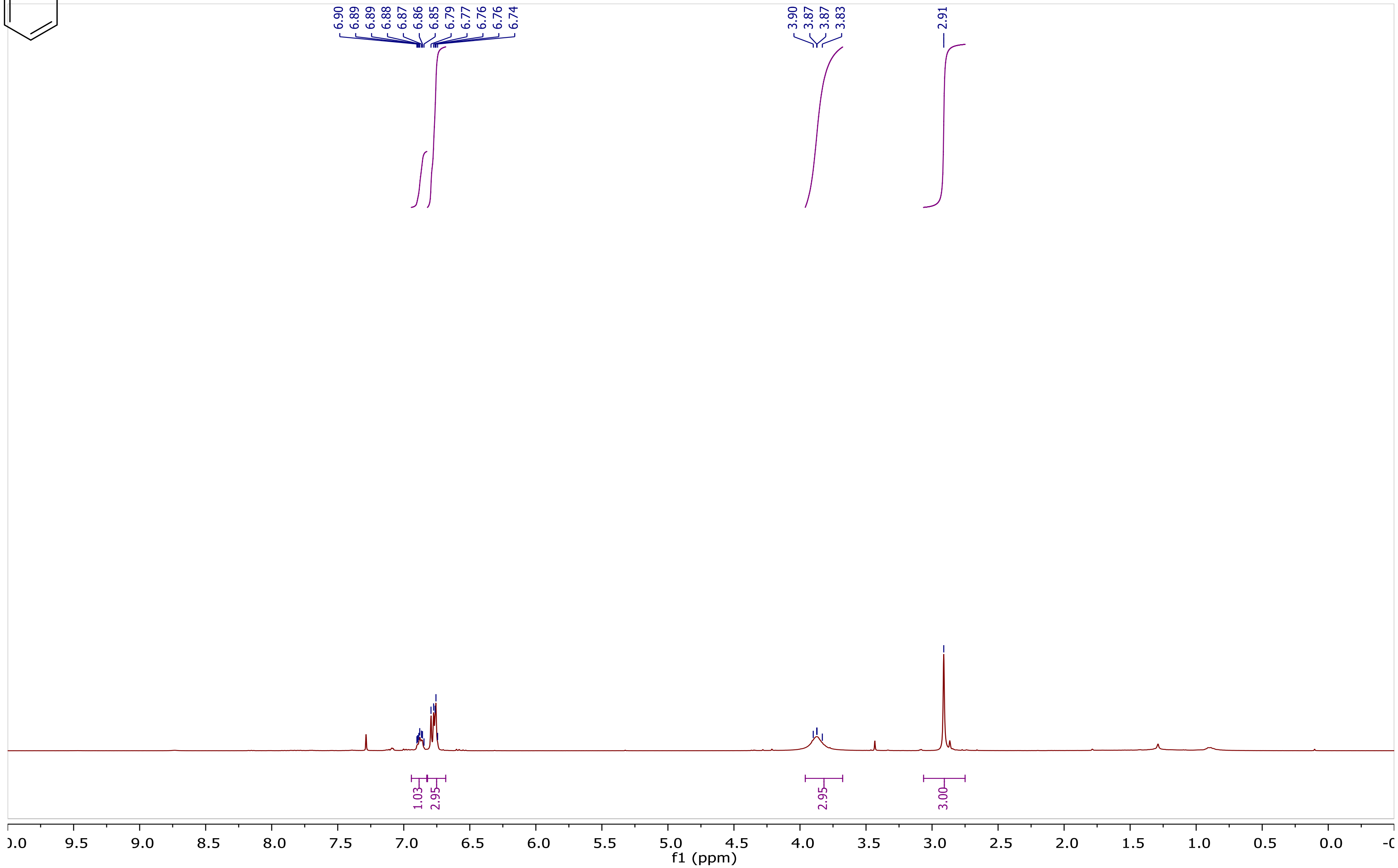

^{13}C NMR of *N*1-methylbenzene-1,2-diamine **4l** in $CDCl_3$

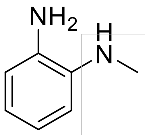

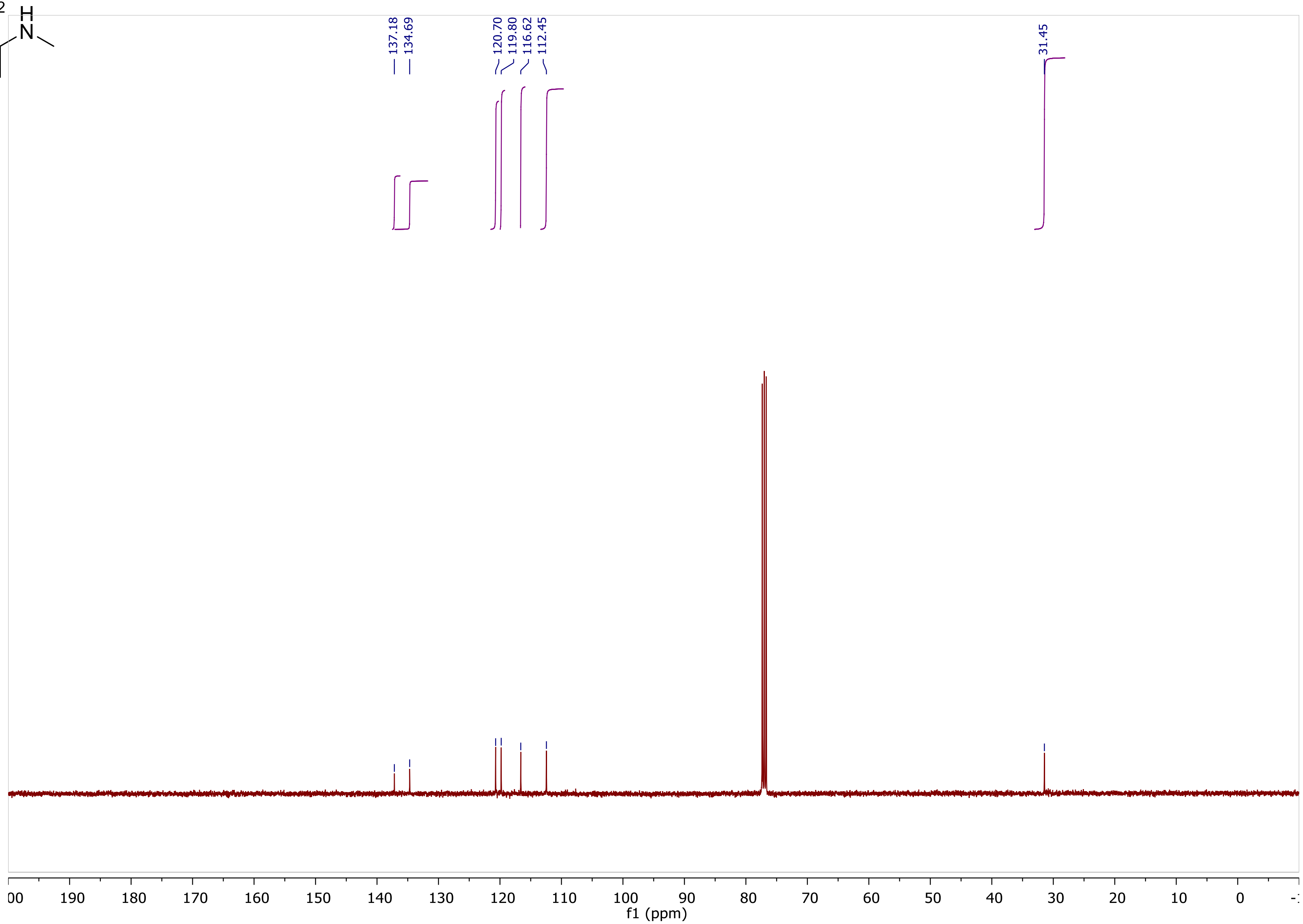

Product Functionalisations

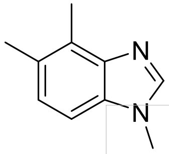

^{1}H NMR of 1,4,5-trimethyl-1*H*-benzo[d]imidazole **6a** in CD_3CN

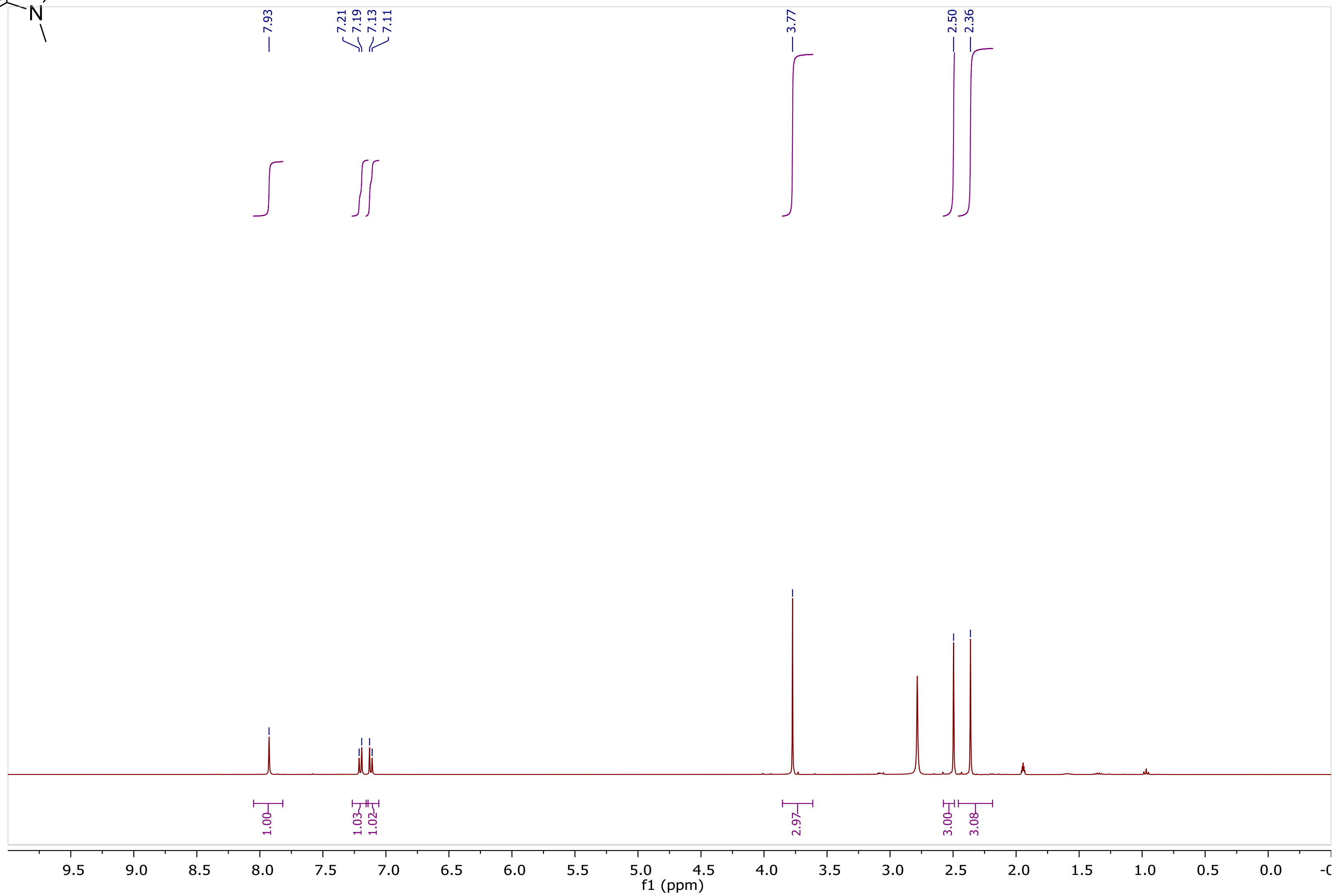

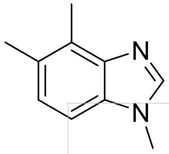

^{13}C NMR of 1,4,5-trimethyl-1*H*-benzo[d]imidazole **6a** in CD_3CN

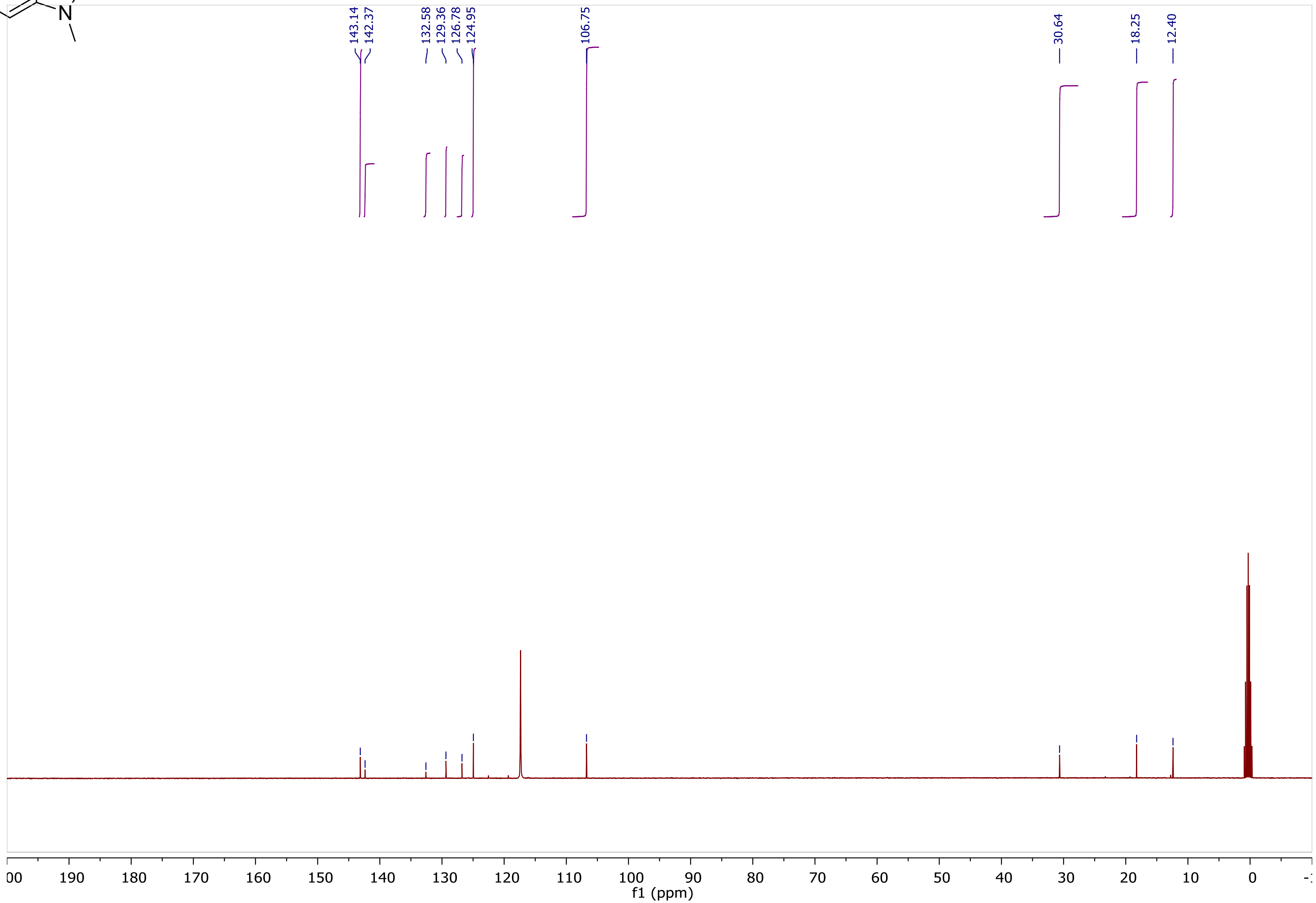

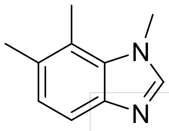

^{1}H NMR of 1,6,7-trimethyl-1*H*-benzo[d]imidazole **6b** in $CDCl_3$

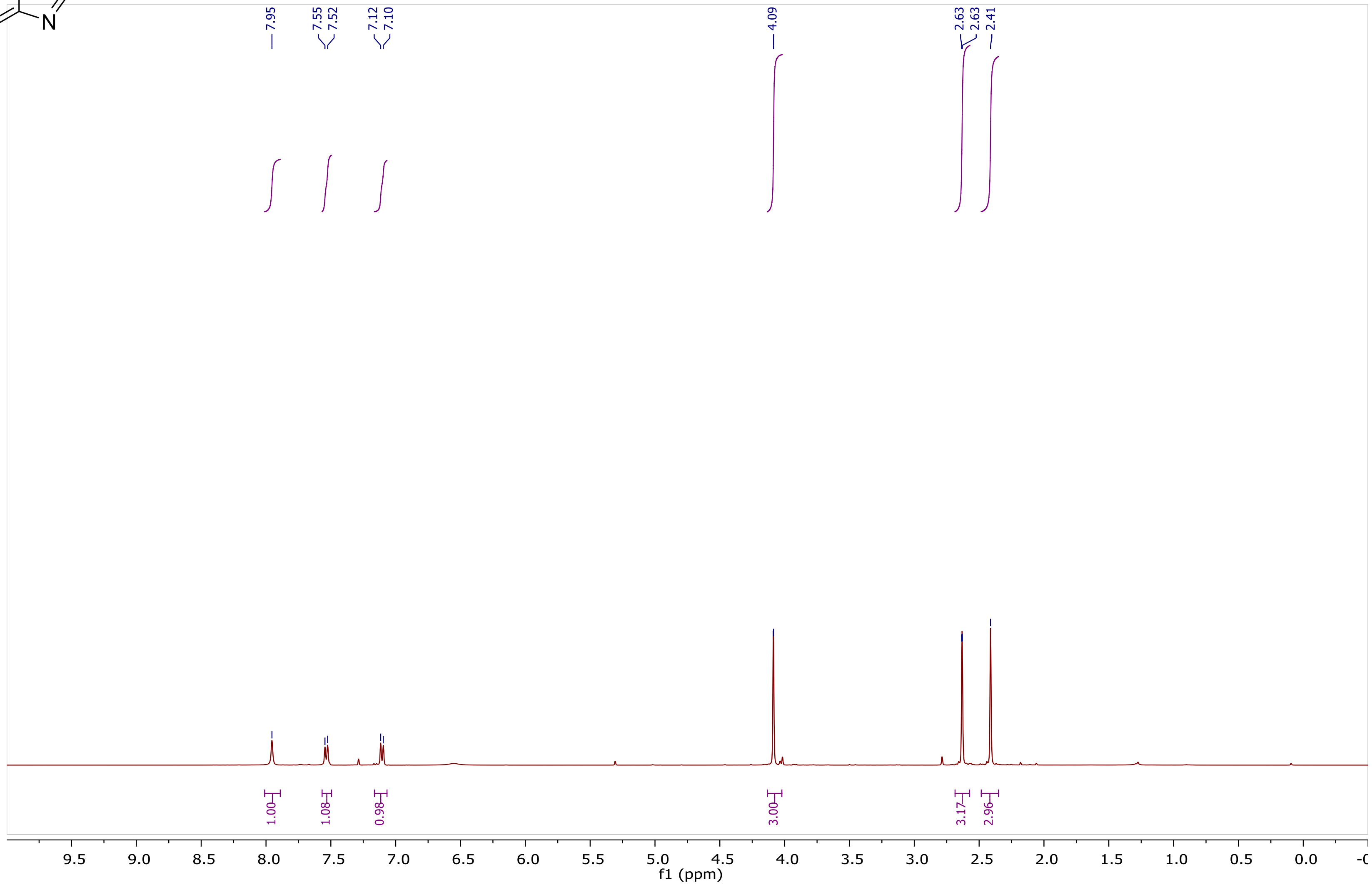

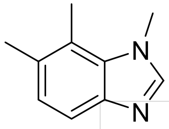

^{13}C NMR of 1,6,7-trimethyl-1*H*-benzo[d]imidazole **6b** in $CDCl_3$

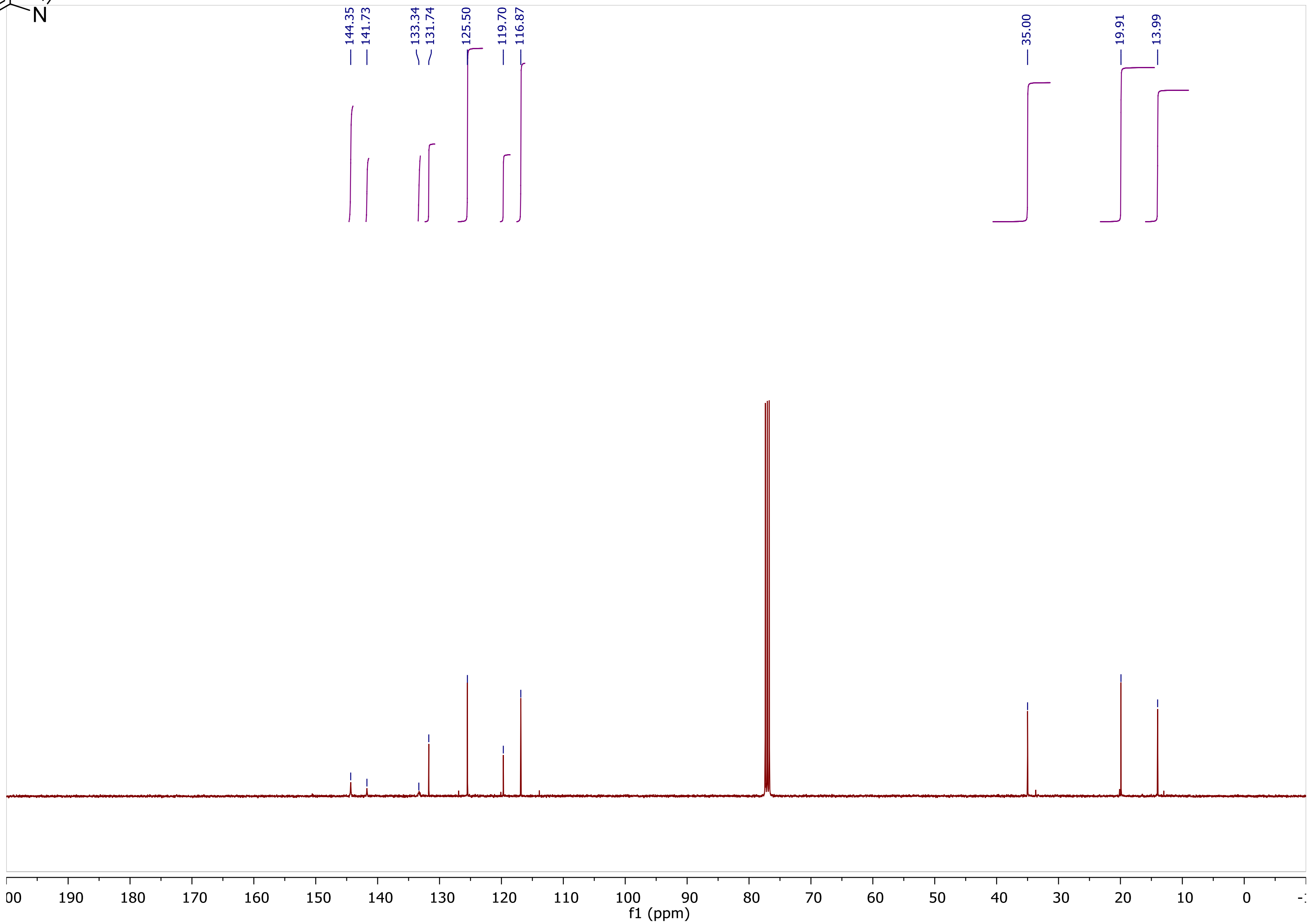

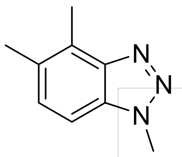

^{1}H NMR of 1,4,5-trimethyl-1*H*-benzo[d][1,2,3]triazole **6c** in $CDCl_3$

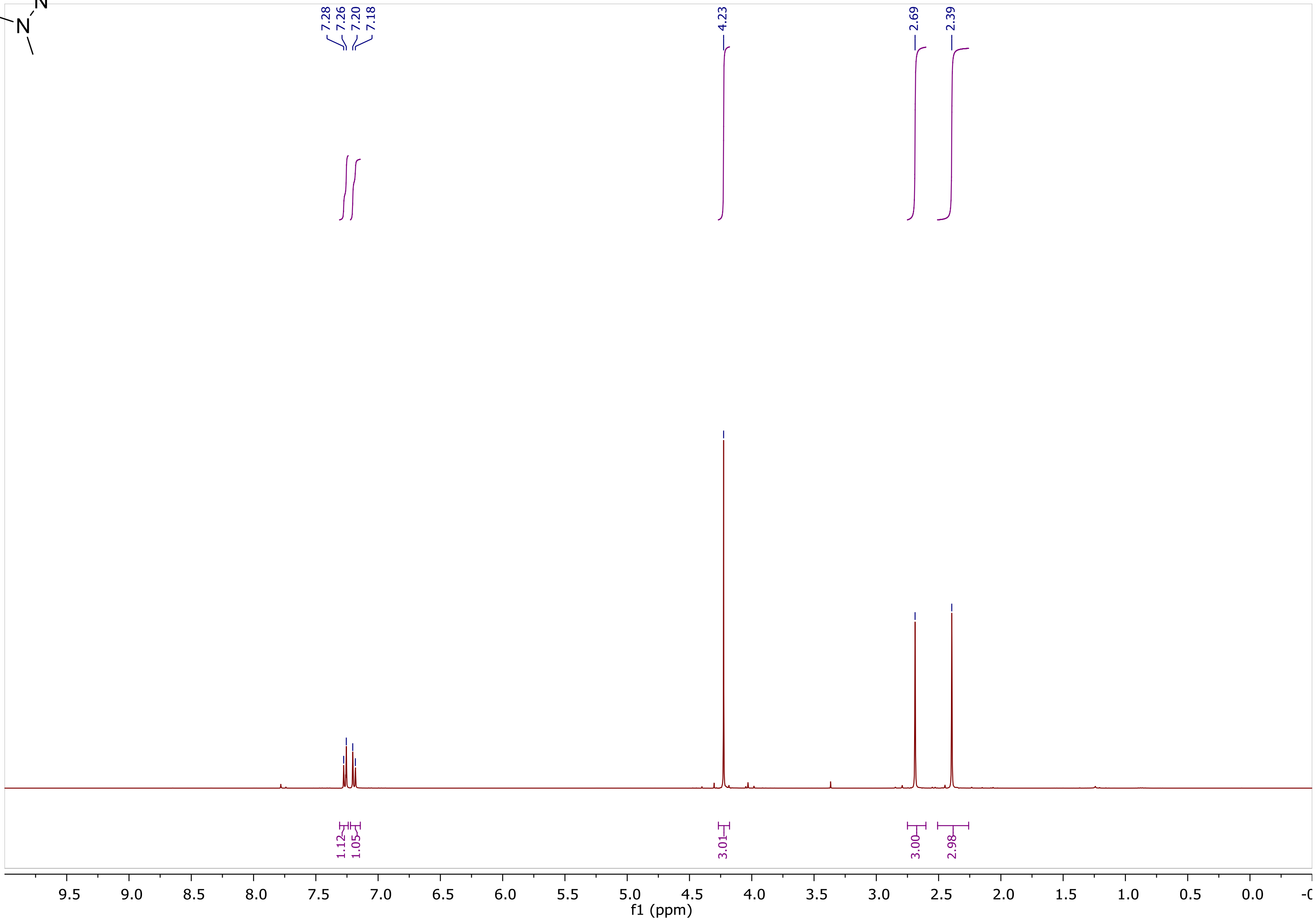

^{13}C NMR of 1,4,5-trimethyl-1*H*-benzo[d][1,2,3]triazole **6c** in $CDCl_3$

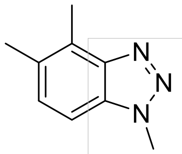

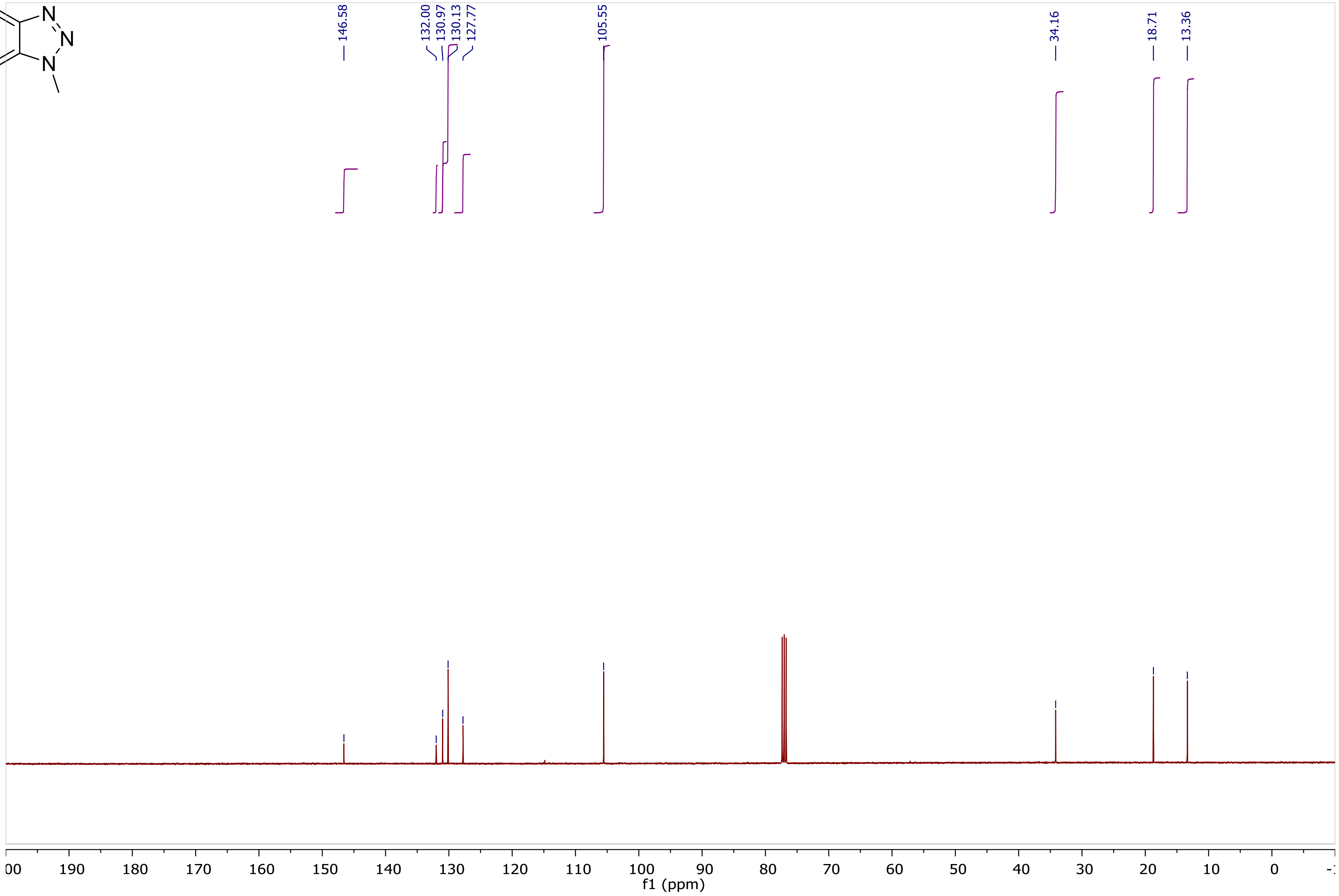

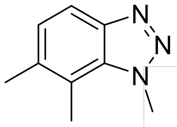

^{1}H NMR of 1,6,7-trimethyl-1*H*-benzo[d][1,2,3]triazole **6d** in $CDCl_3$

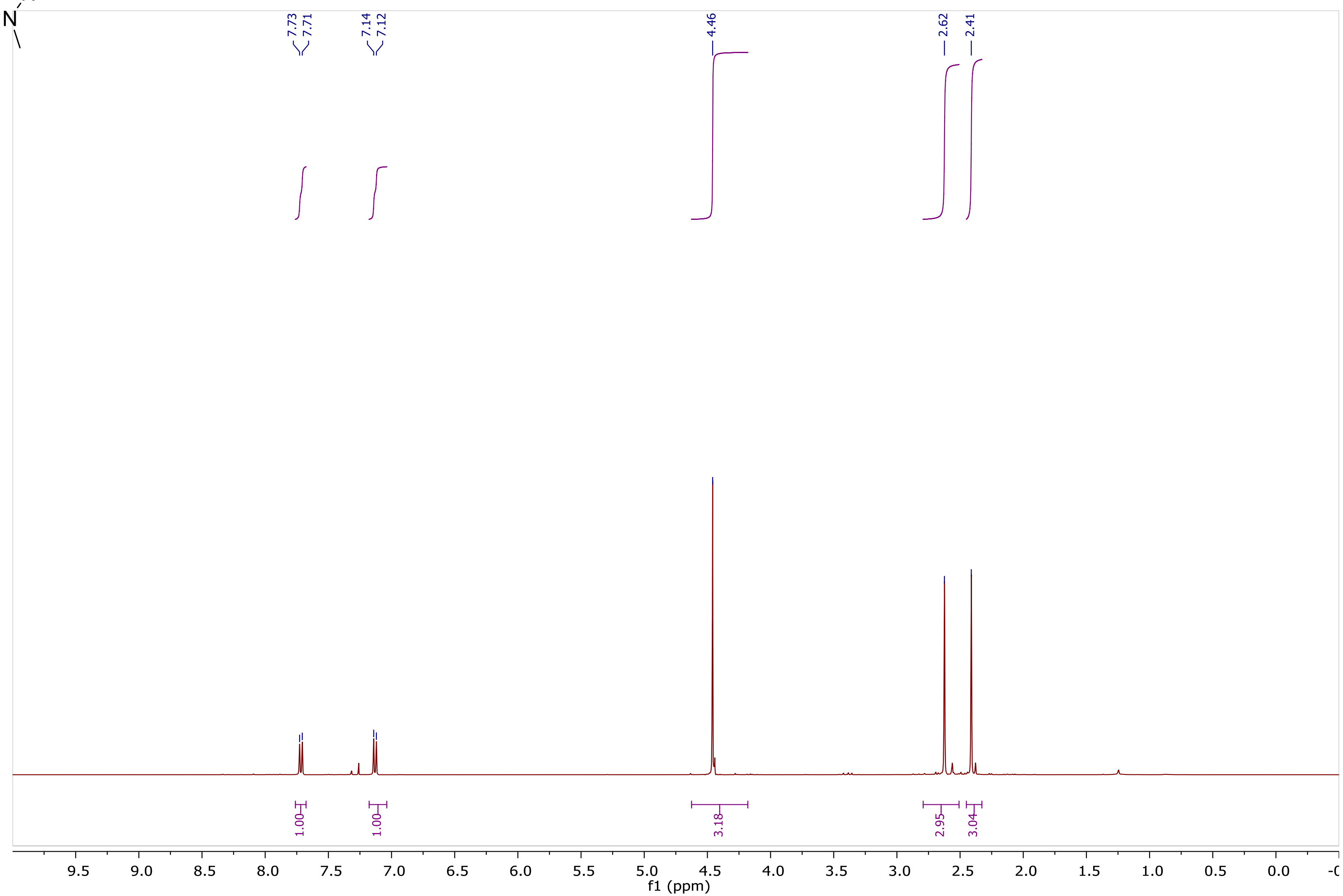

^{13}C NMR of 1,6,7-trimethyl-1*H*-benzo[d][1,2,3]triazole **6d** in $CDCl_3$

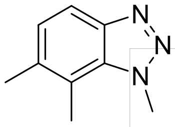

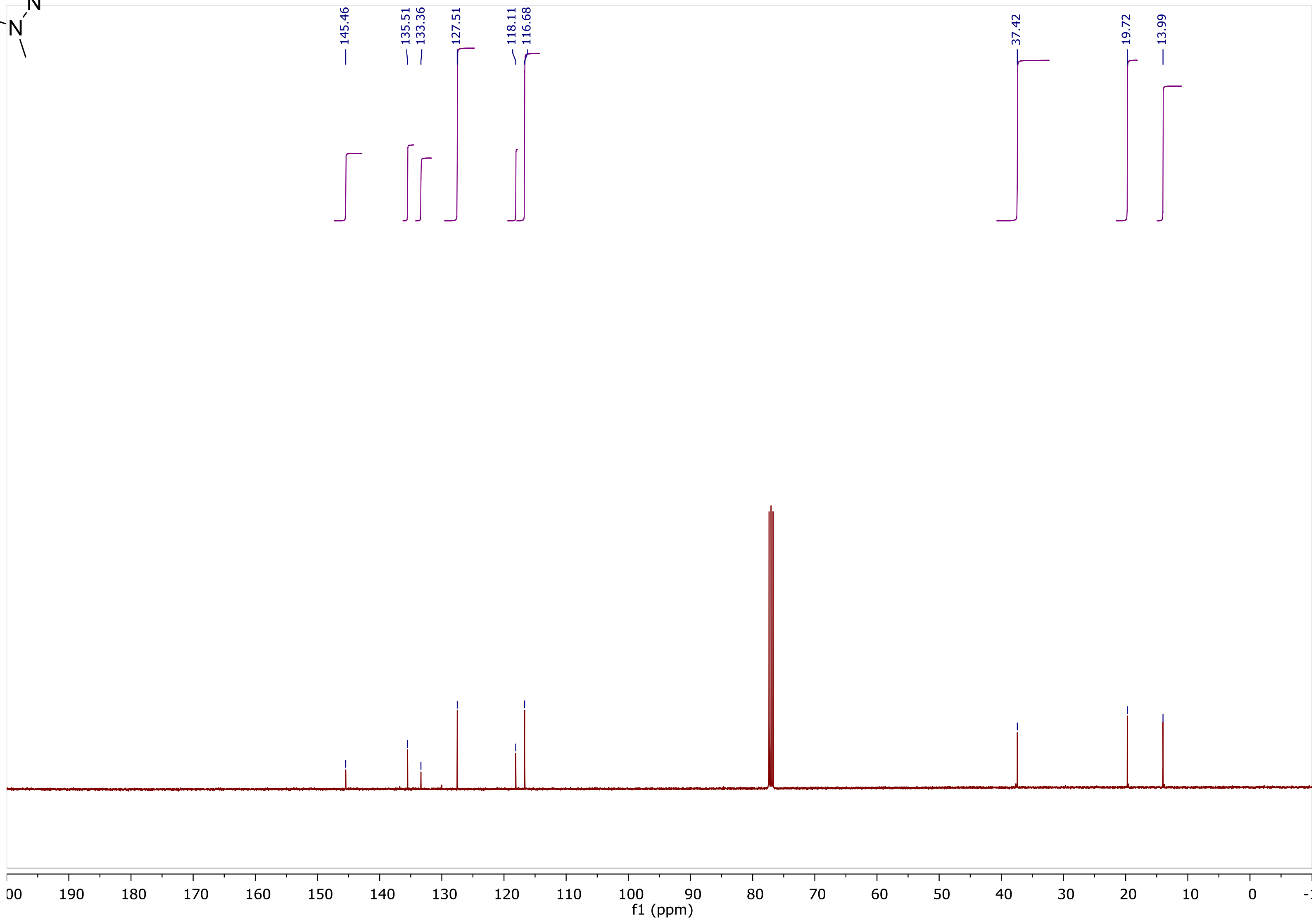

^{1}H NMR of 2,3-diphenylquinoxaline **6e** in $CDCl_3$

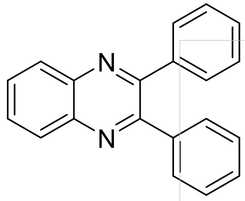

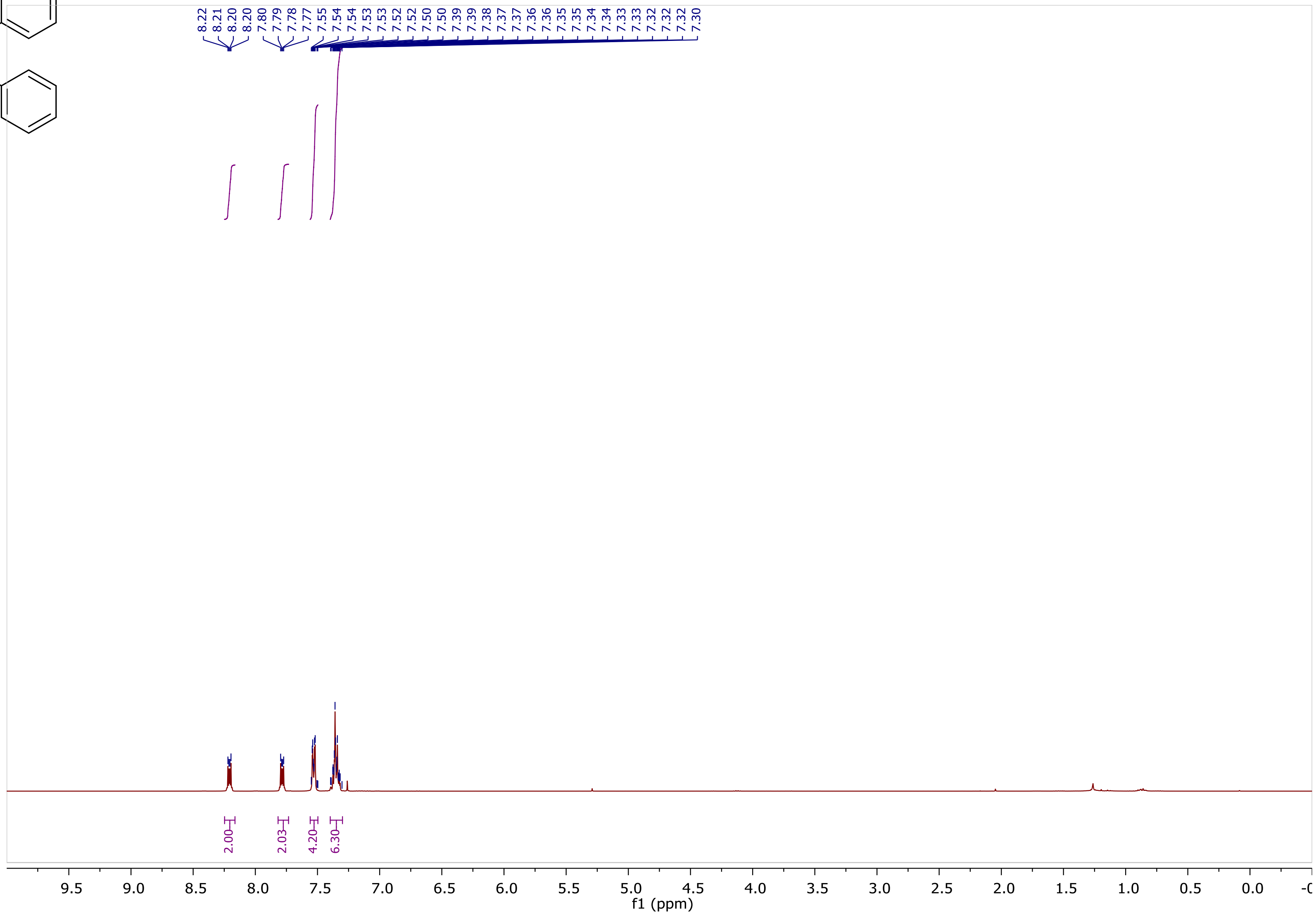

^{13}C NMR of 2,3-diphenylquinoxaline **6e** in $CDCl_3$

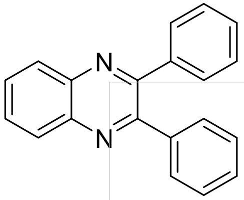

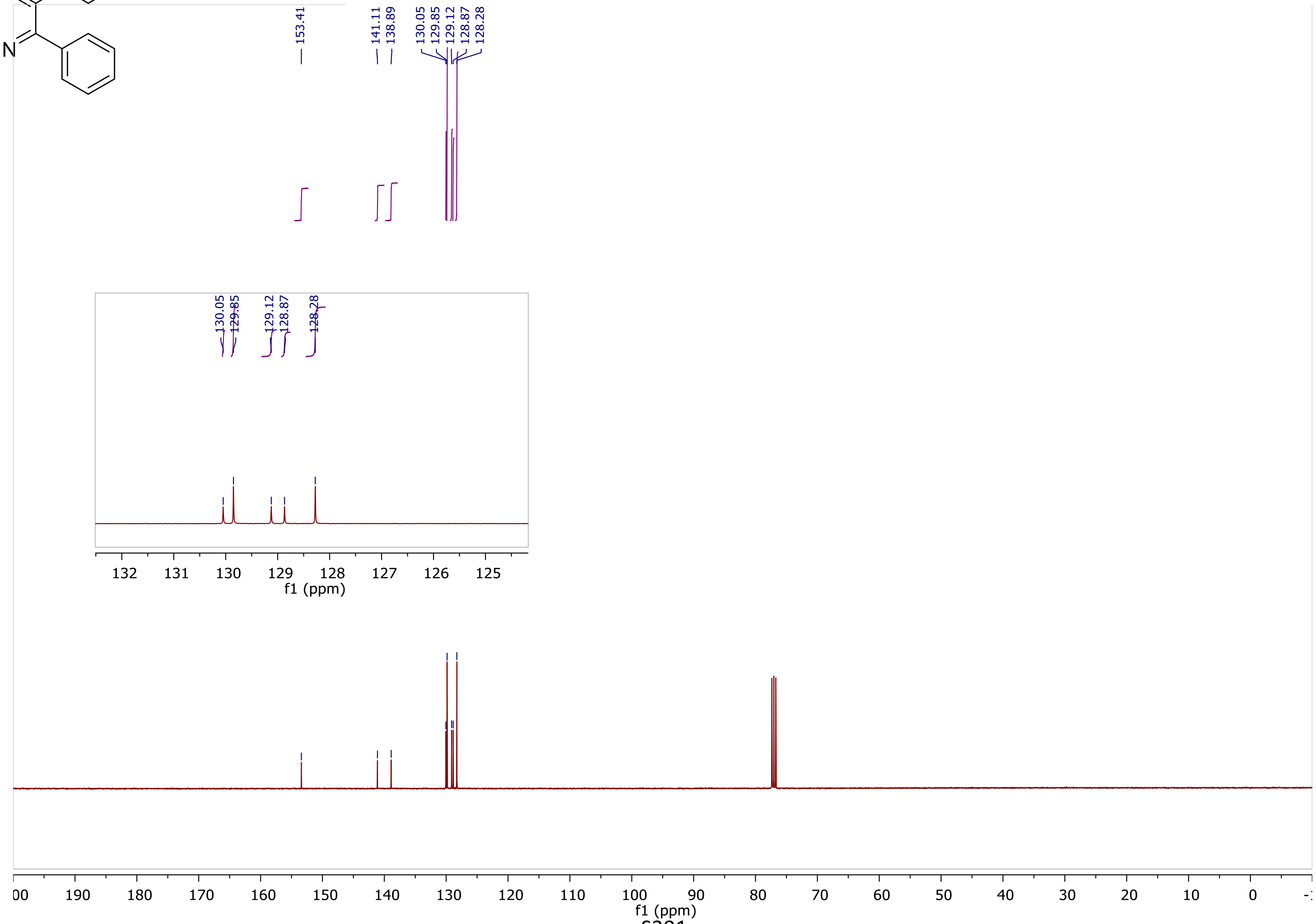

^{1}H NMR of 2,2,4-trimethyl-2,3-dihydro-1*H*-benzo[b][1,4]diazepine **6f** in $CDCl_3$

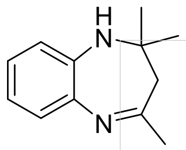

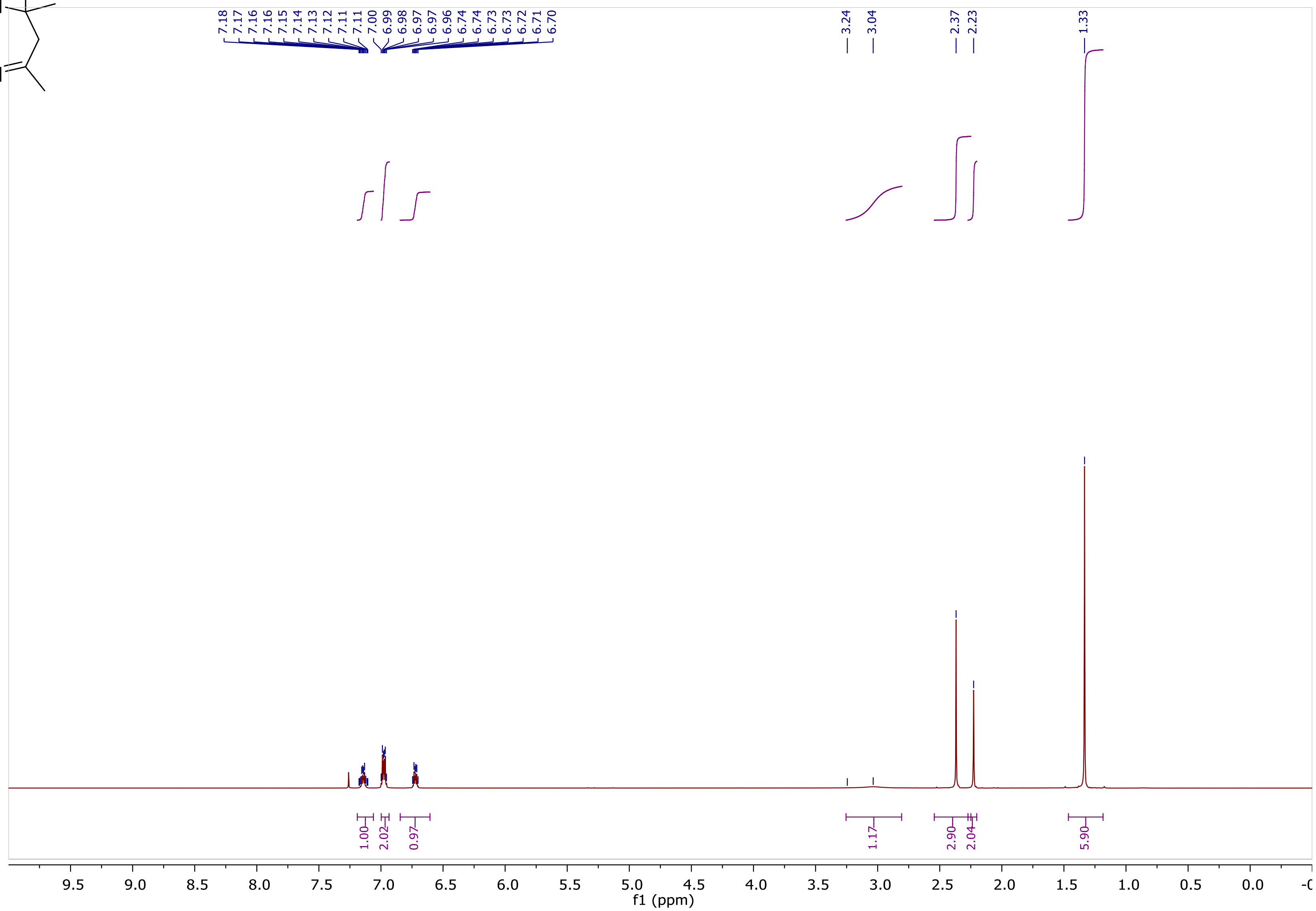

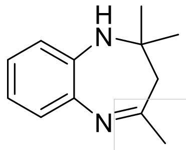

^{13}C NMR of 2,2,4-trimethyl-2,3-dihydro-1*H*-benzo[b][1,4]diazepine **6f** in $CDCl_3$

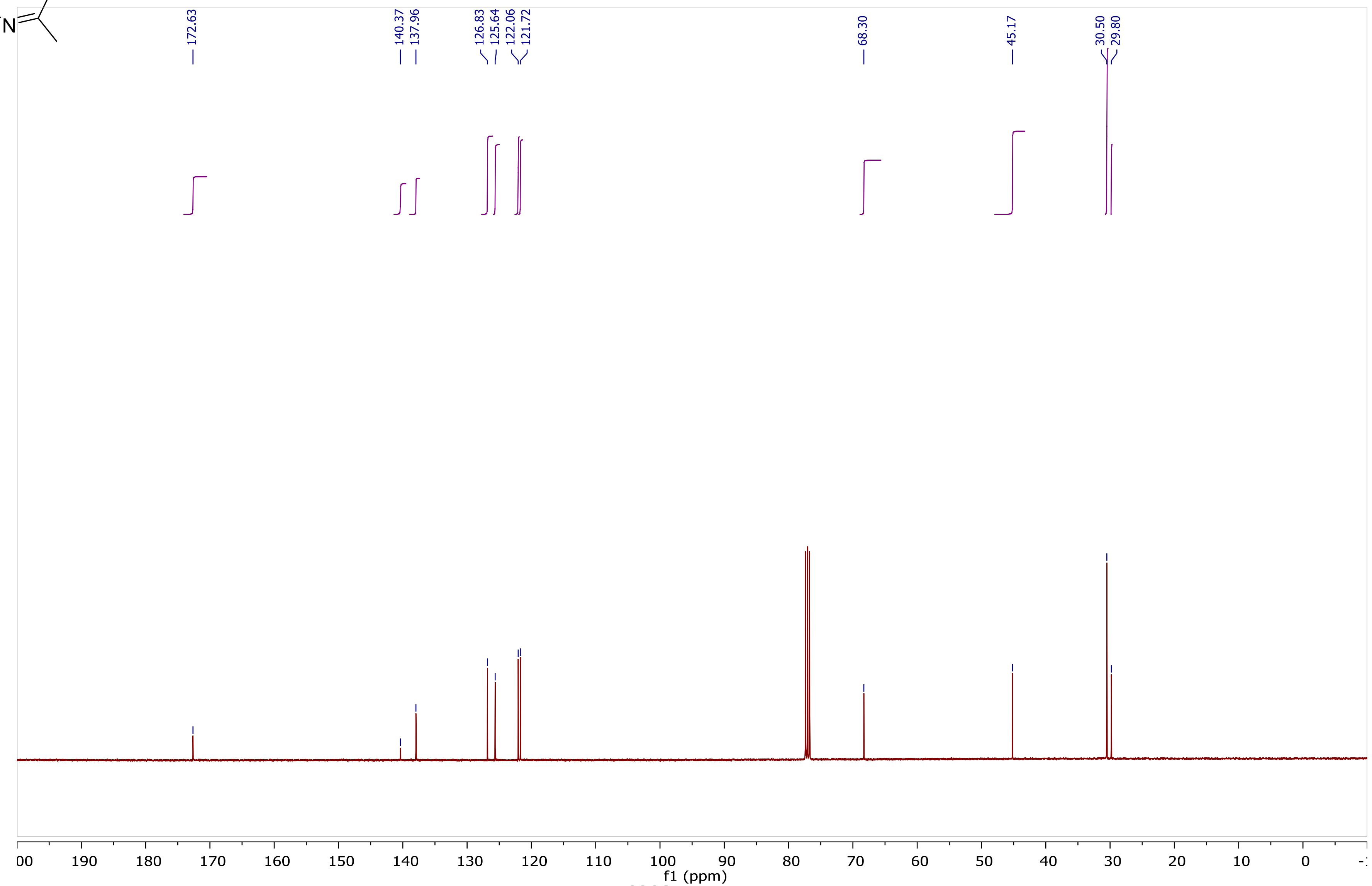

Control Reaction on a Neutral Substrate

^{1}H NMR of sodium phenylsulfamate in D_2O

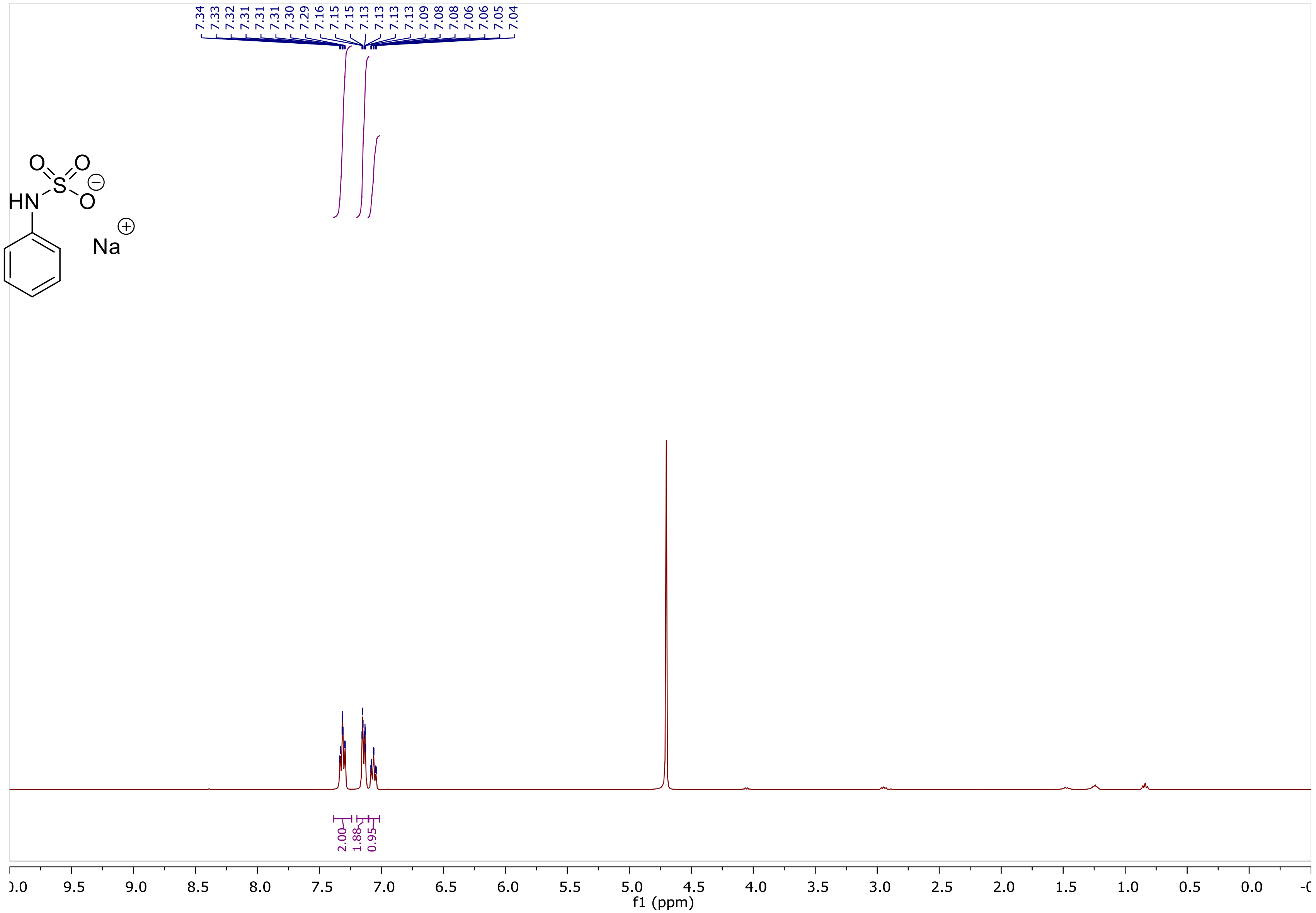

^{1}H NMR of sodium phenylsulfamate in D_2O

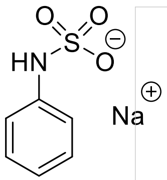

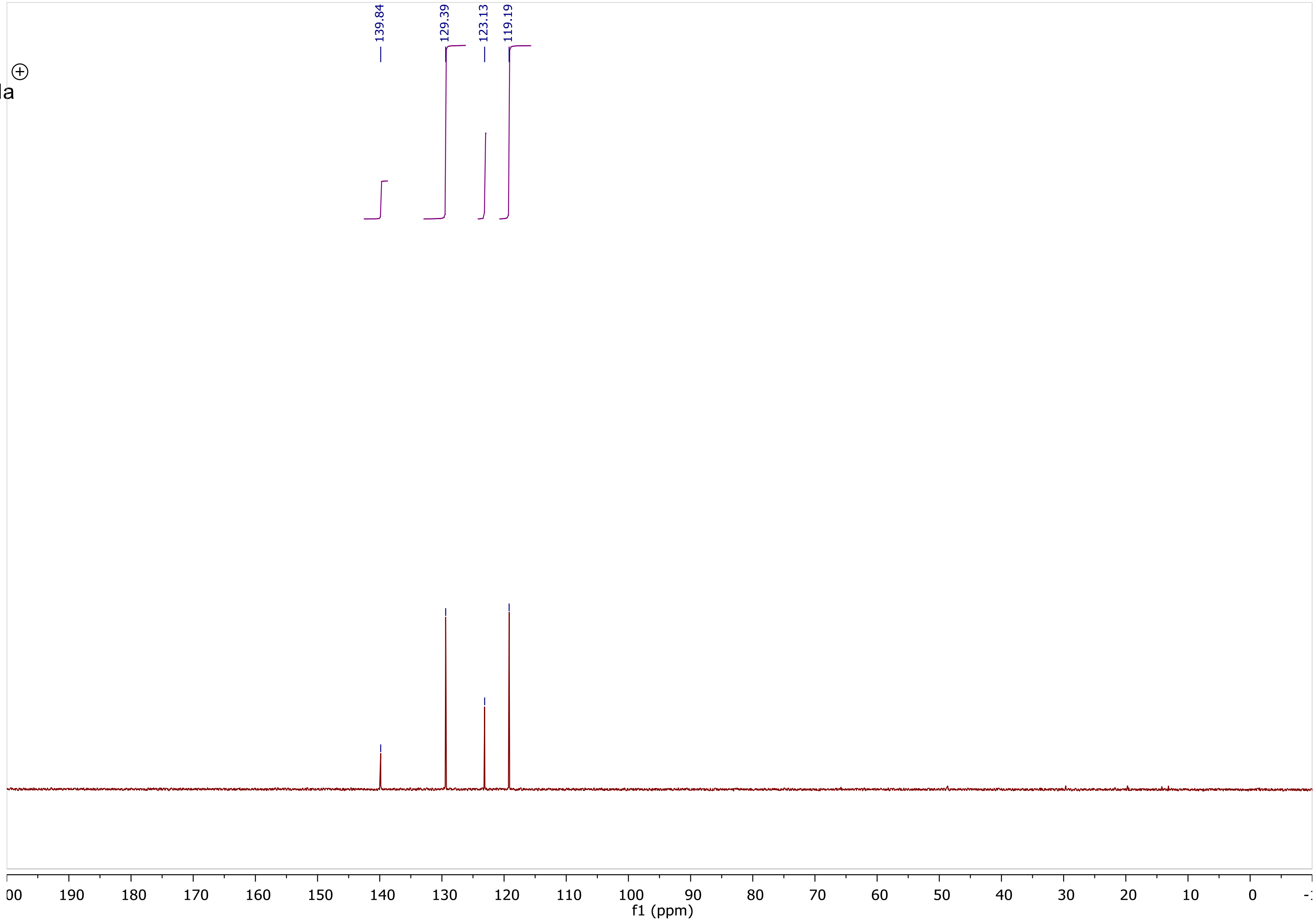

^{1}H NMR of neopentyl phenylsulfamate **7** in $CDCl_3$

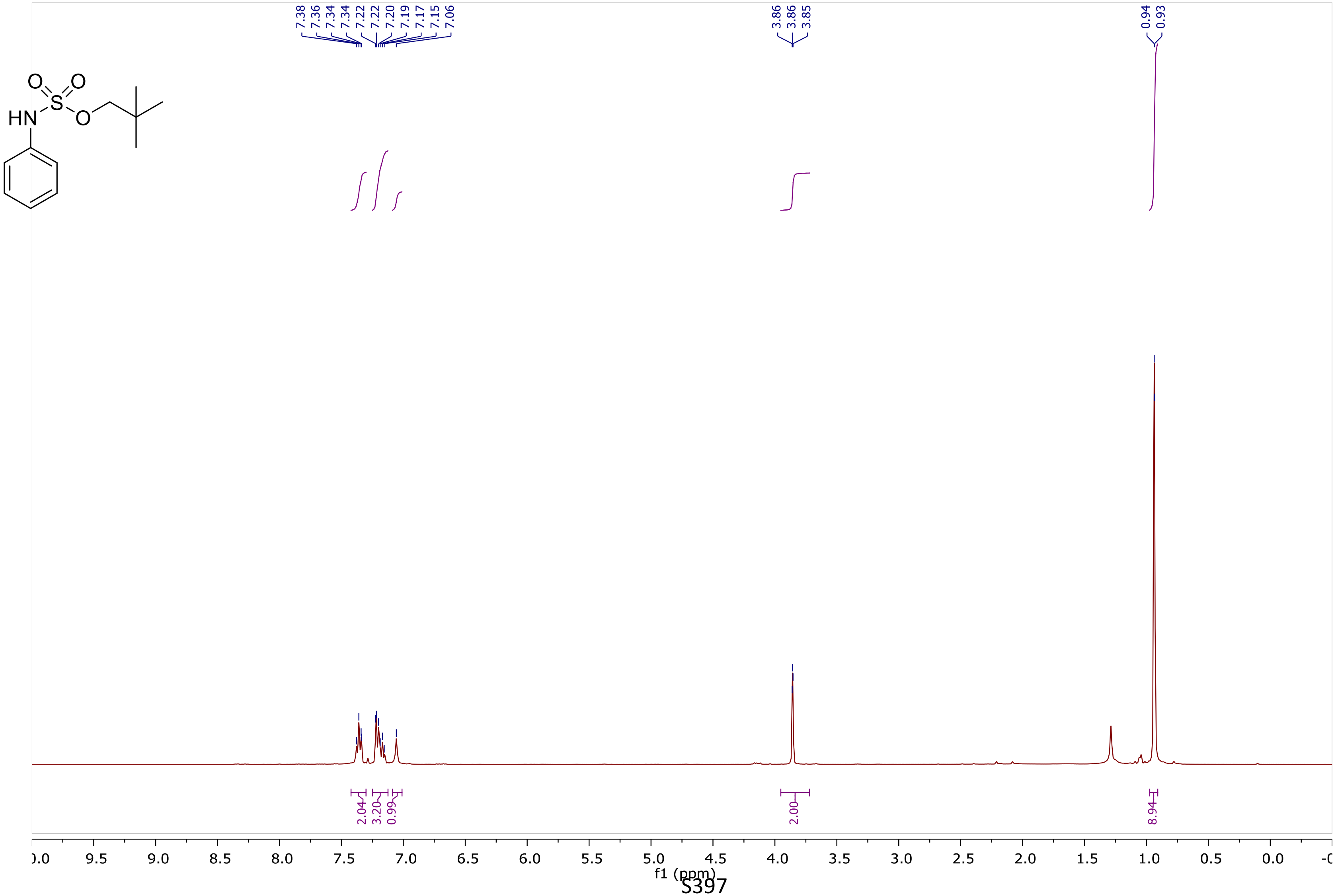

^{13}C NMR of neopentyl phenylsulfamate **7** in $CDCl_3$

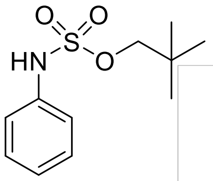

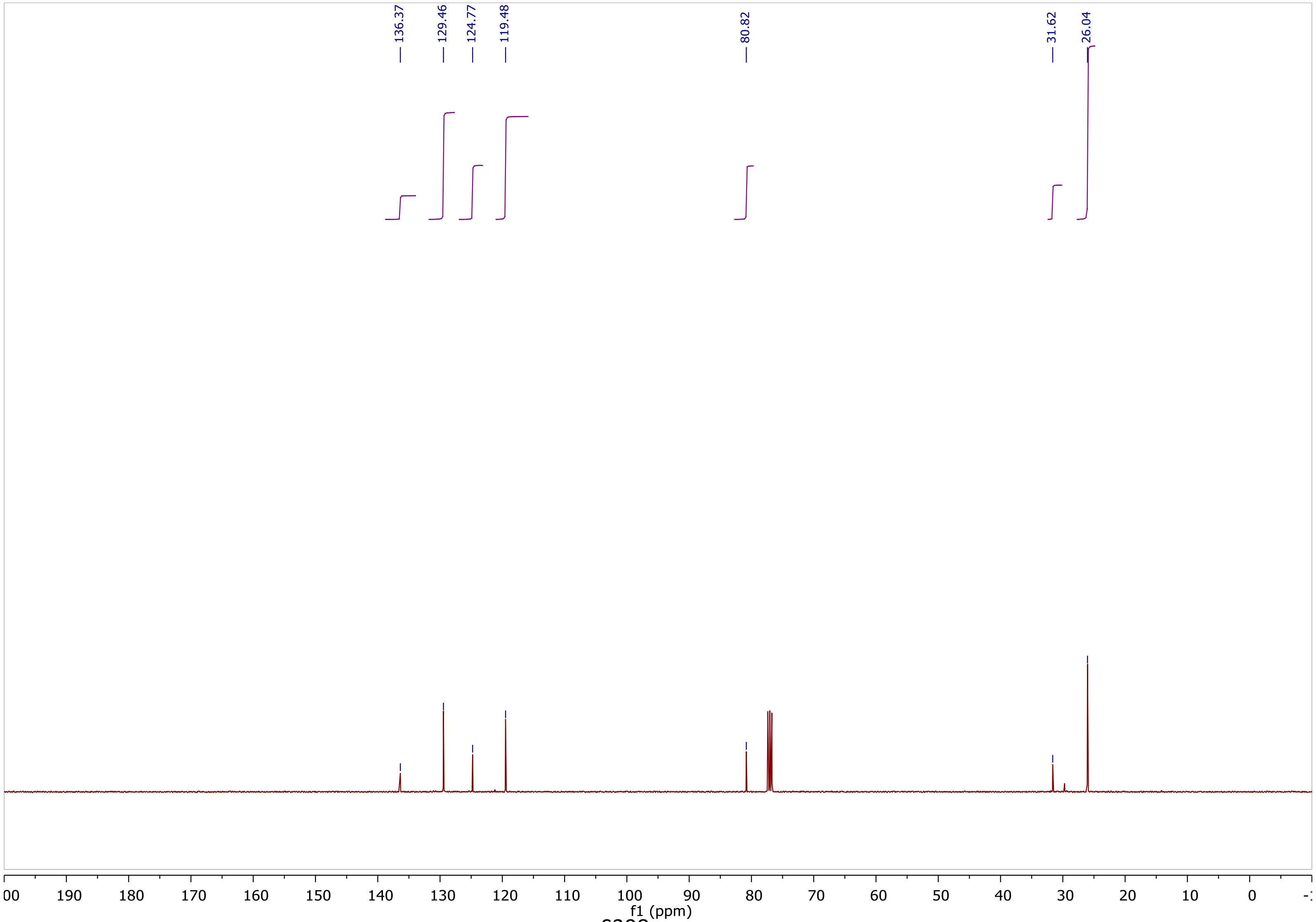

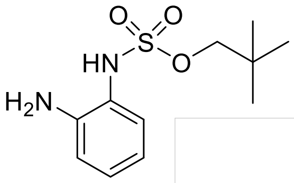

^1H NMR of neopentyl (2-aminophenyl)sulfamate in $CDCl_3$

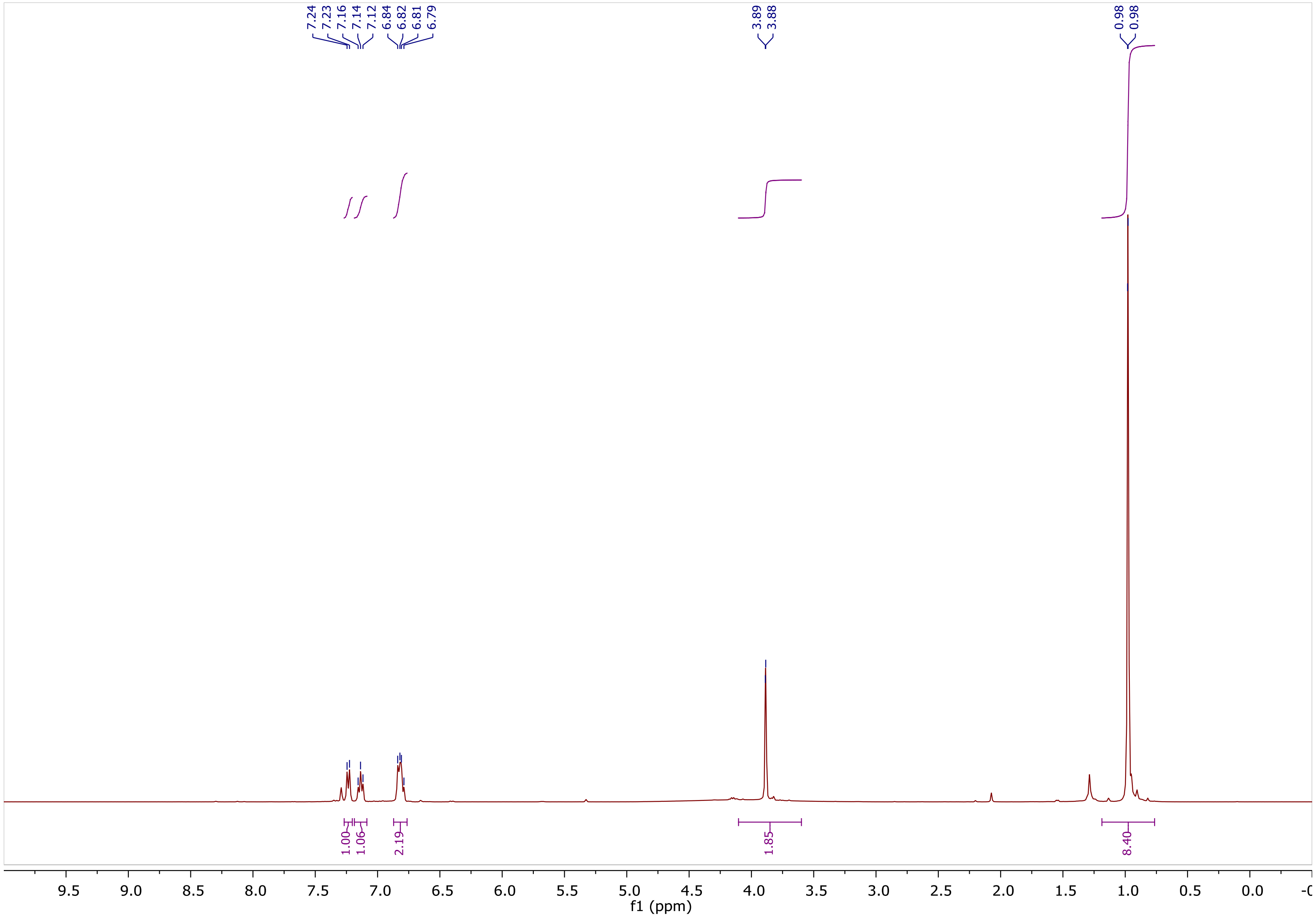

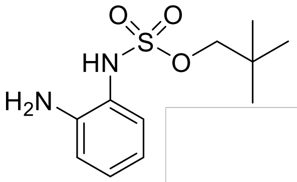

^{13}C NMR of neopentyl (2-aminophenyl)sulfamate in $CDCl_3$

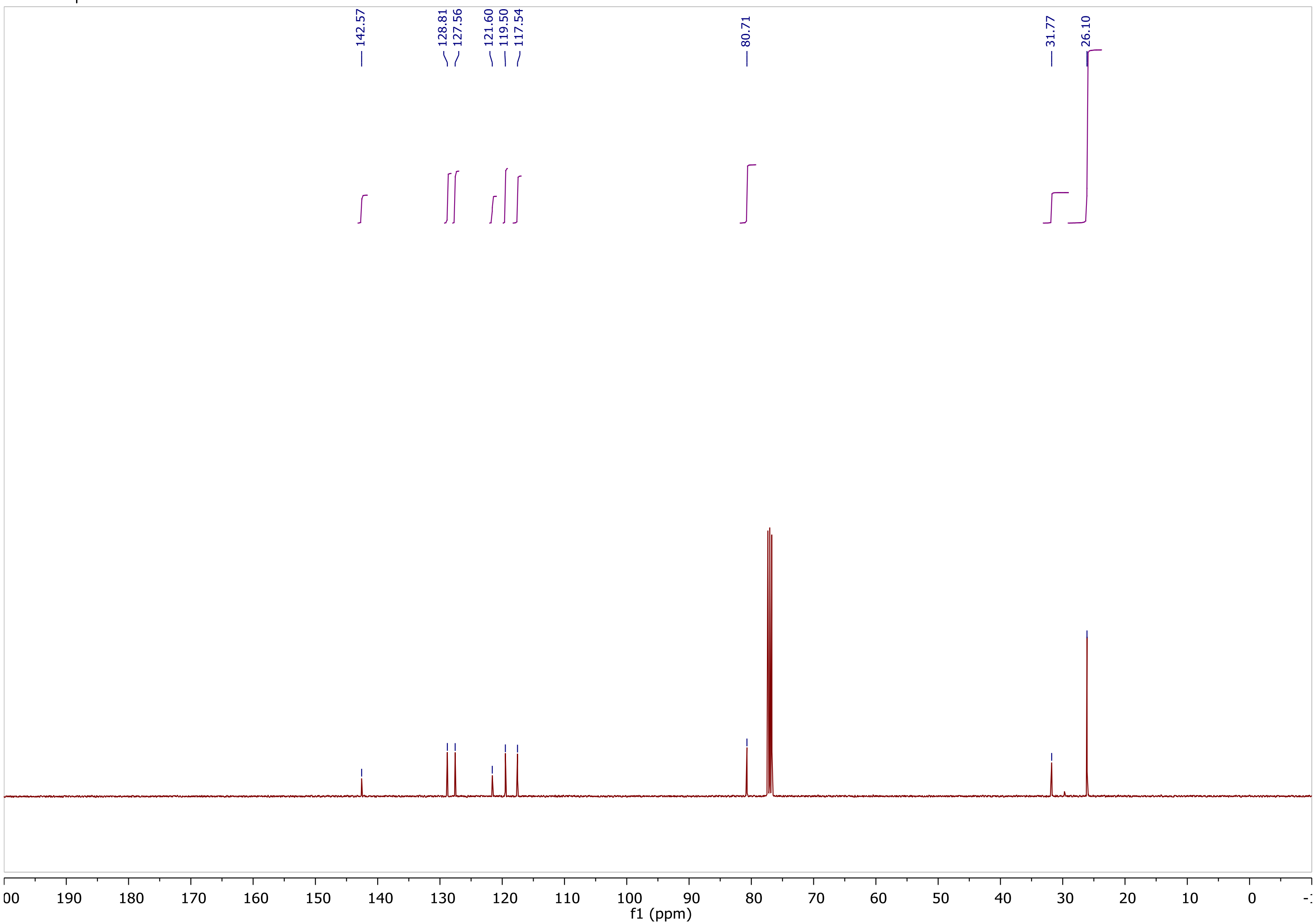

^{1}H NMR of neopentyl (4-aminophenyl)sulfamate in $CDCl_3$

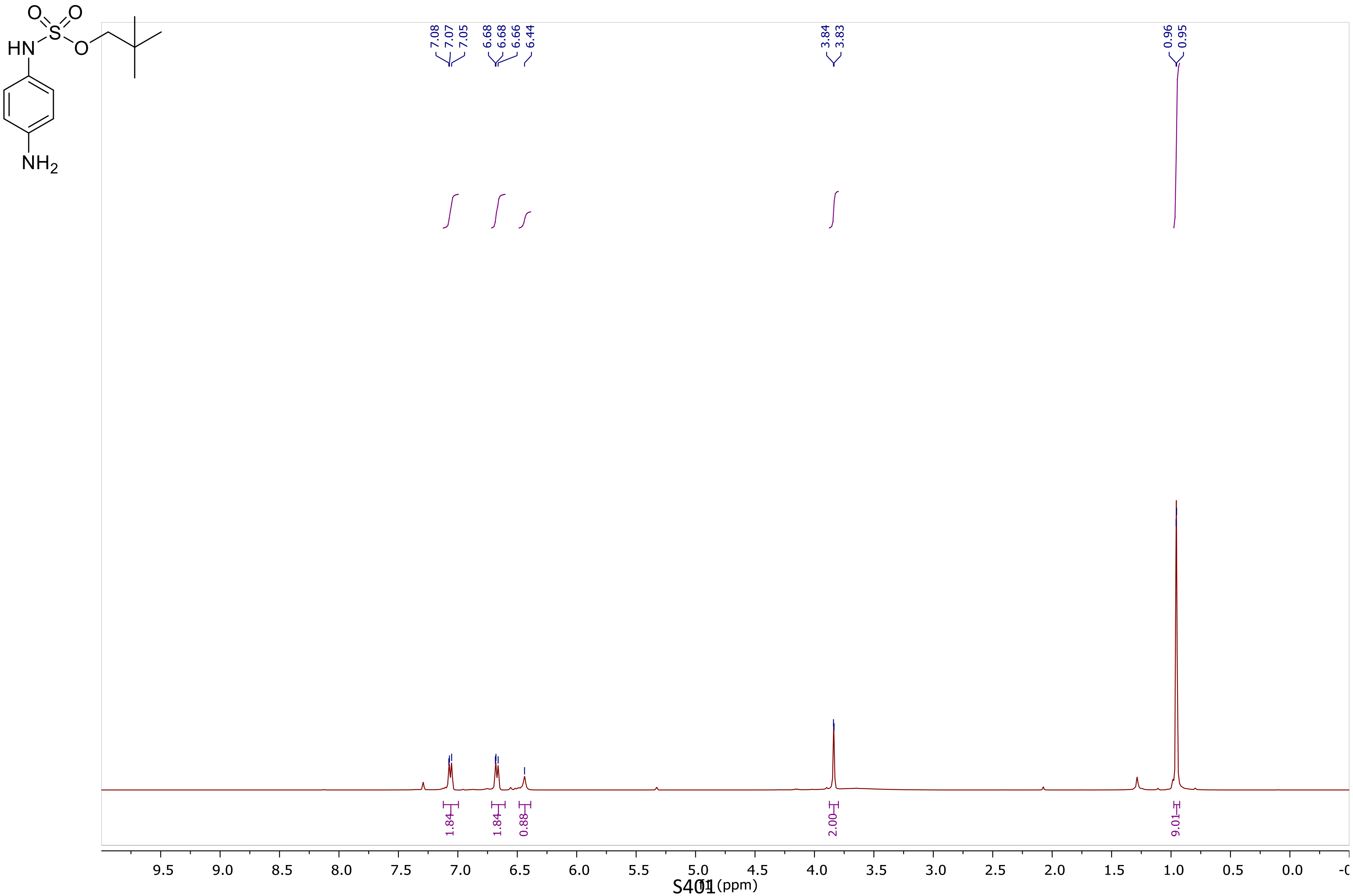

^{13}C NMR of neopentyl (4-aminophenyl)sulfamate in $CDCl_3$

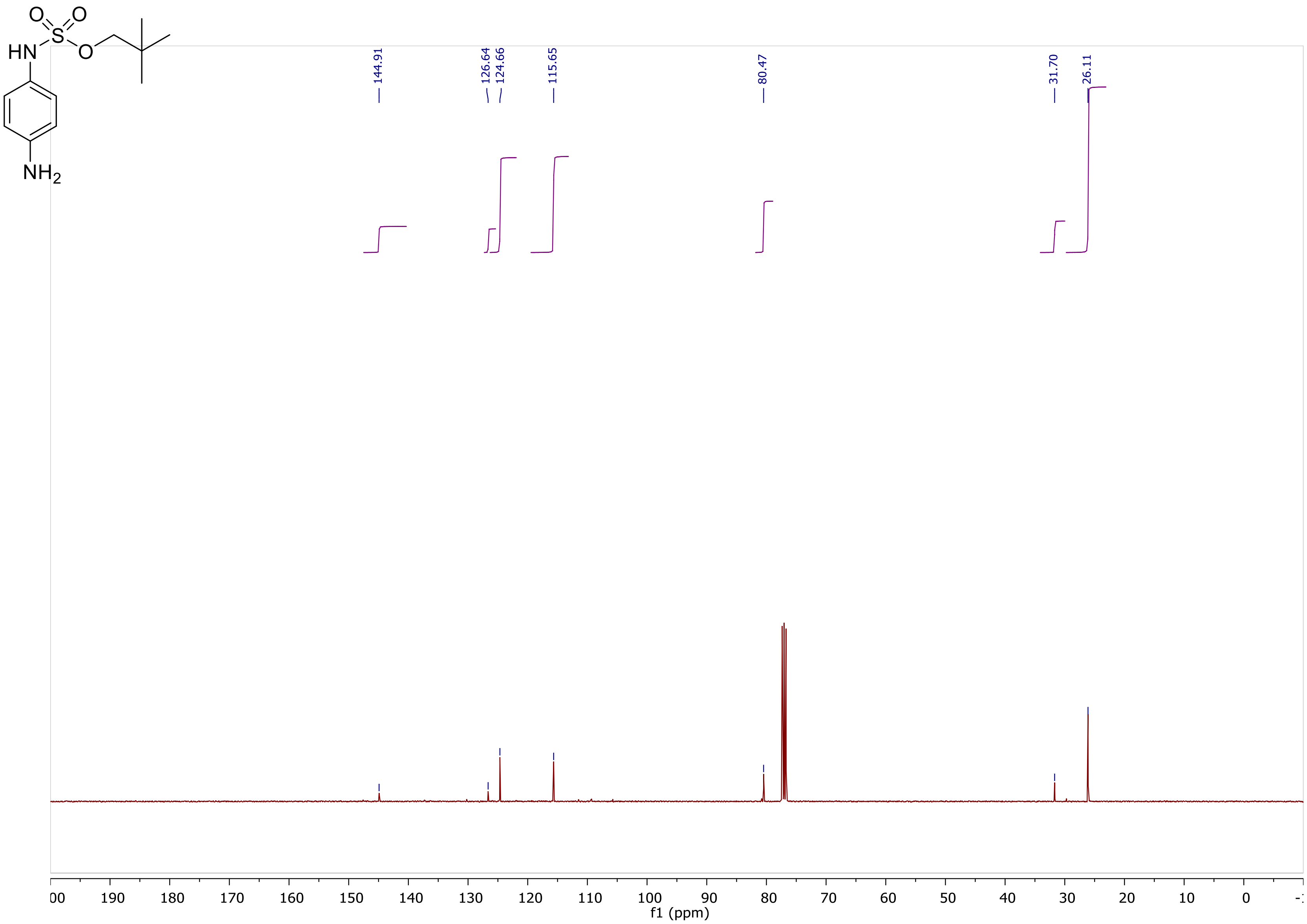

^{1}H NMR of tetrabutylammonium phenylsulfate **8** in $CDCl_3$

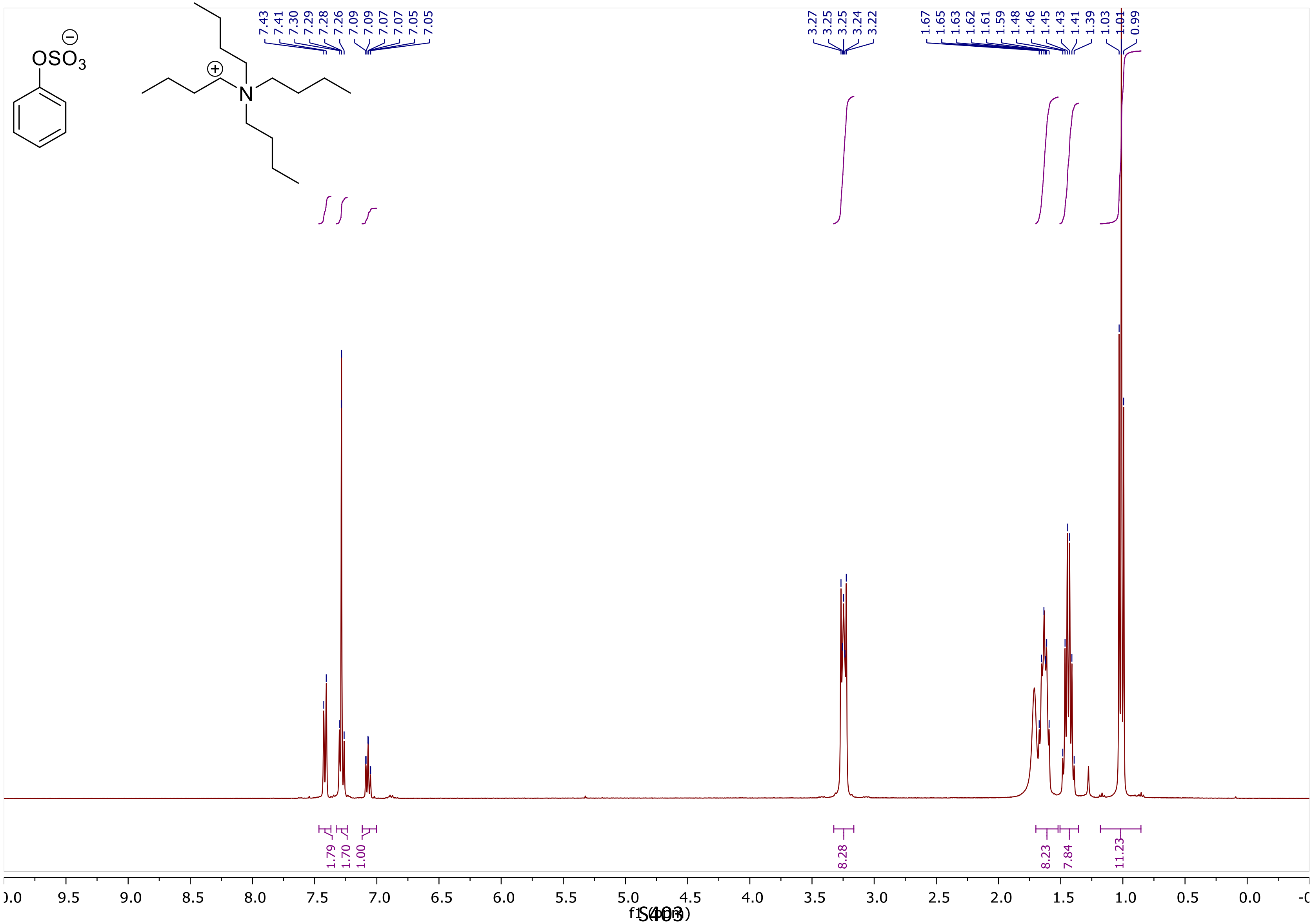

^{13}C NMR of tetrabutylammonium phenylsulfate **8** in $CDCl_3$

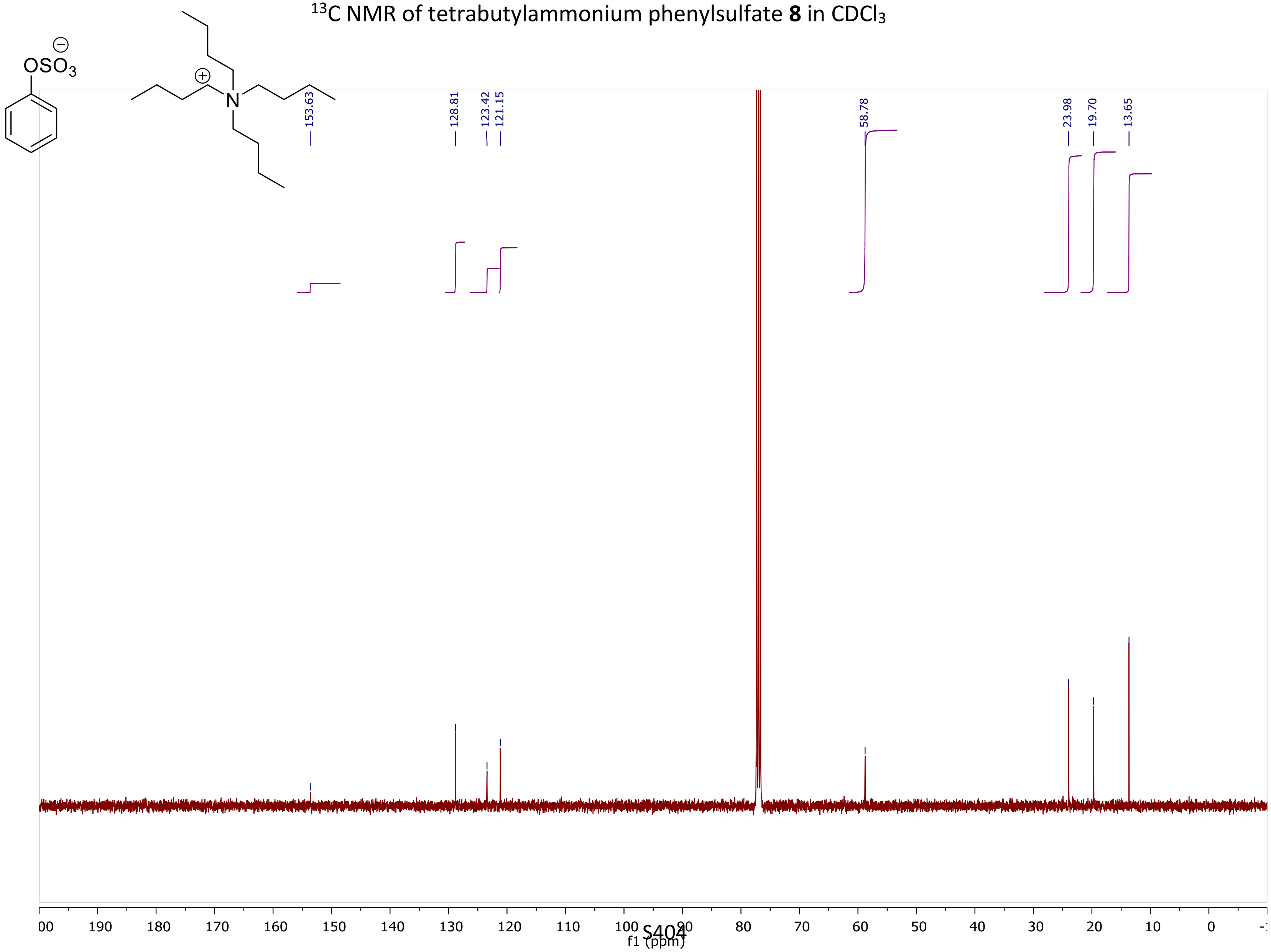

^1H NMR of 2-aminophenol **9** in DMSO-d6

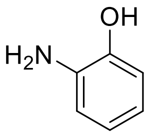

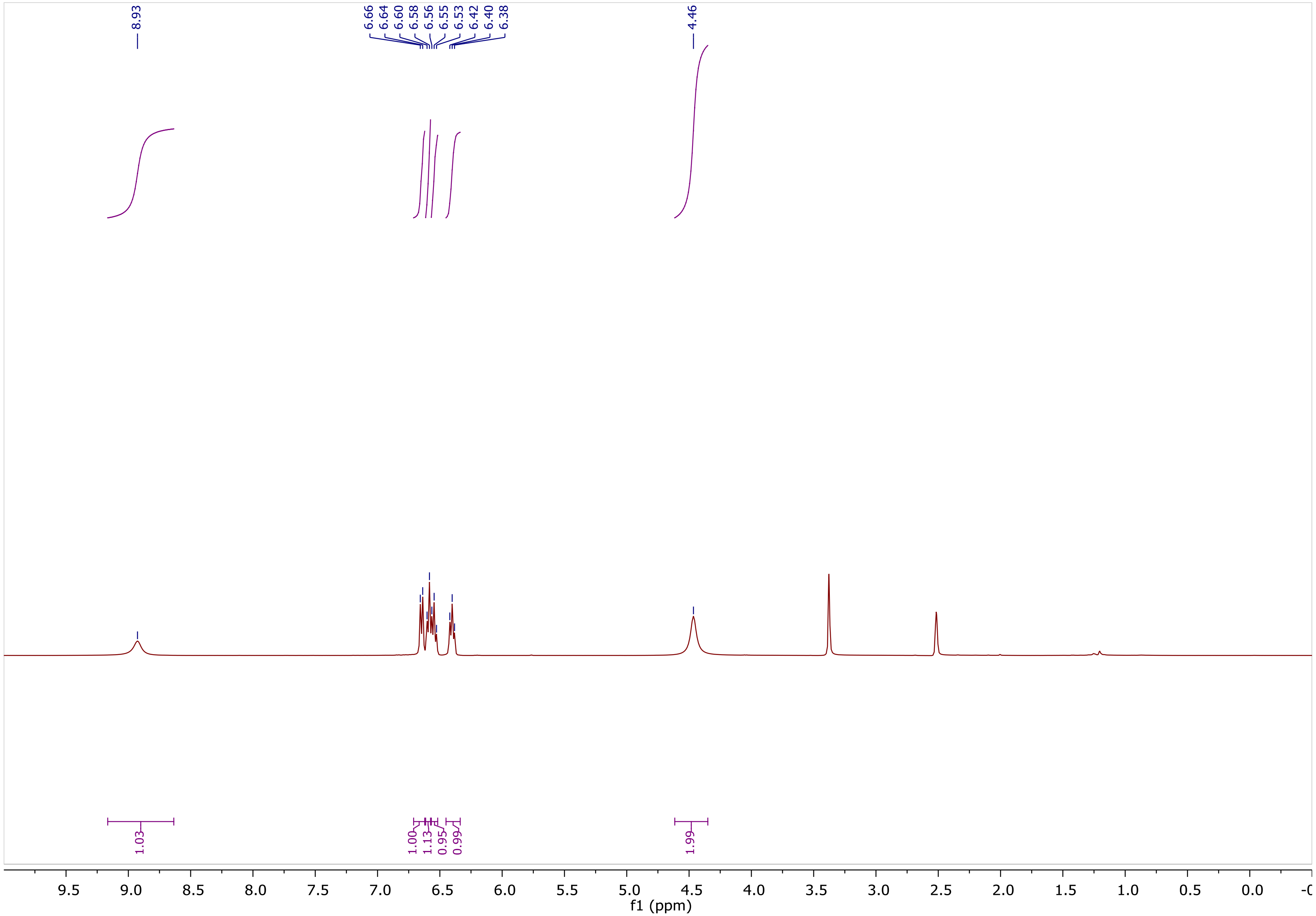

^{13}C NMR of 2-aminophenol **9** in DMSO-d6

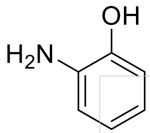

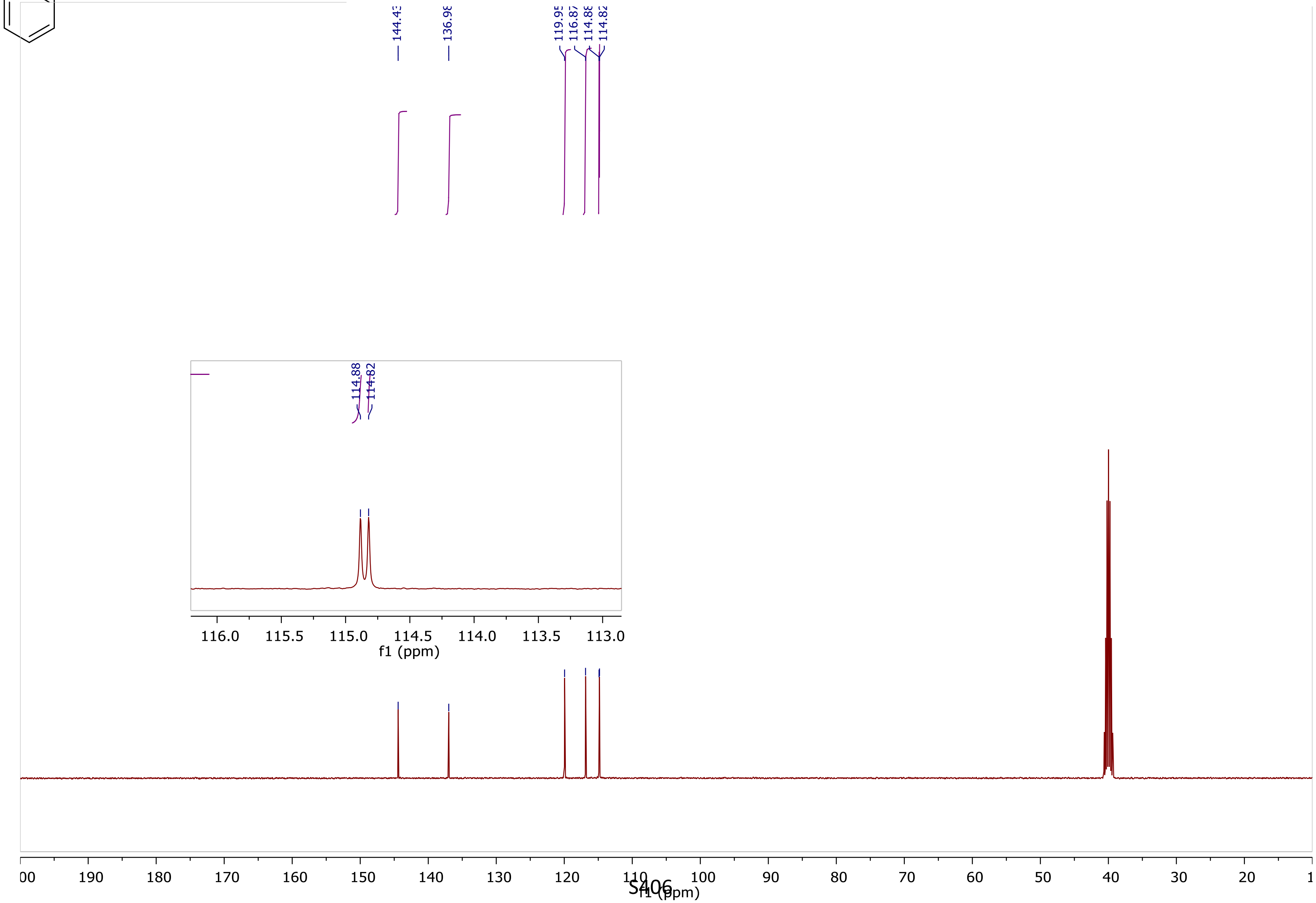

^{1}H NMR of 4-aminophenol in DMSO-d6

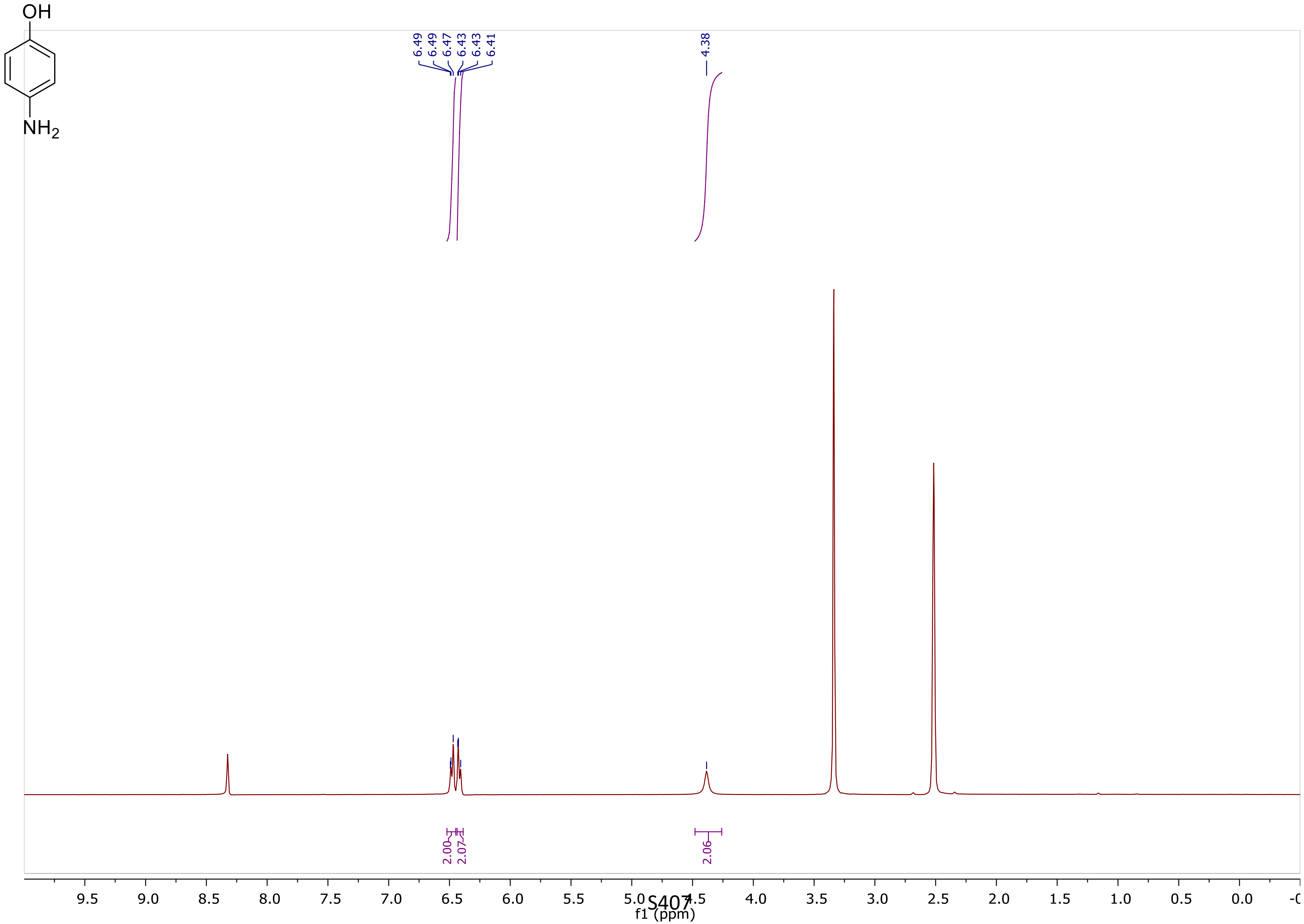

^{13}C NMR of 4-aminophenol in DMSO-d6

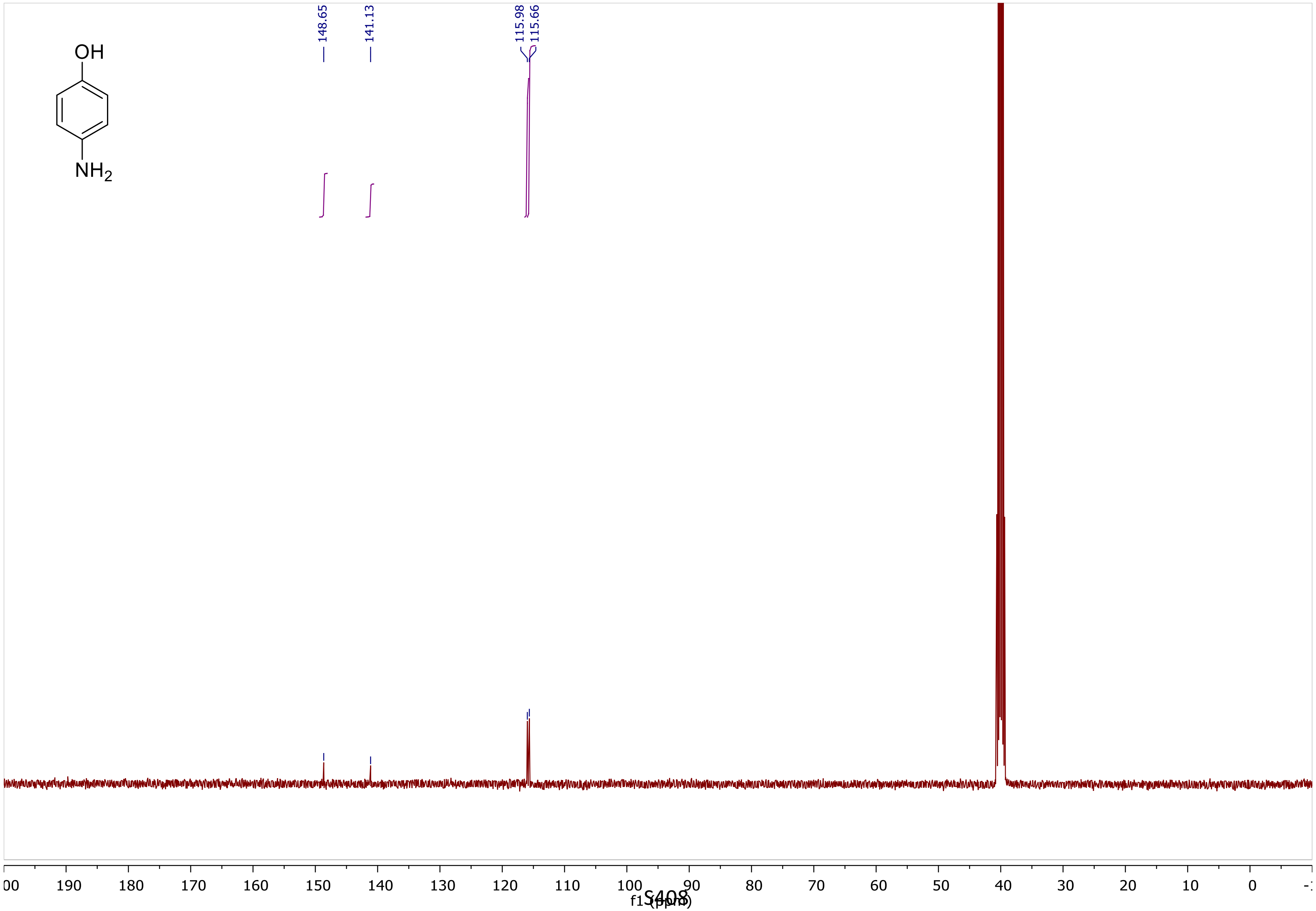

Supplement: Supplementary file 1 — ja1c05531_si_001.pdf [file ja1c05531_si_001.pdf]
